# Supplementary material for: Iron photocatalysis via Brønsted acid-unlocked ligand-to-metal charge transfer
Source: Nat Commun. 2024 Jul 20;15:6115. doi: 10.1038/s41467-024-50507-6 (PMC11271273; doi:10.1038/s41467-024-50507-6)
Supplement: Supplementary file 1 — Supplementary lnformation [file 41467_2024_50507_MOESM1_ESM.pdf]

Supplementary Information

Title: Iron photocatalysis via Brønsted acid-unlocked ligand-to-metal charge transfer

Authors: Xiaoyu Jiang[1], Yu Lan[1,2,3,*], Yudong Hao[1], Kui Jiang[1], Jing He[1], Jiali Zhu[1], Shiqi Jia[1], Jinshuai Song[1], Shi-Jun Li[1,2,*] and Linbin Niu[1,2,*]

Affiliations:

[1]College of Chemistry, and Pingyuan Laboratory, Zhengzhou University, Zhengzhou 450001, Henan, P. R. China.

[2]State Key Laboratory of Antiviral Drugs, Pingyuan Laboratory, Henan Normal University, Xinxiang, Henan, 453007, P. R. China.

[3]School of Chemistry and Chemical Engineering, Chongqing Key Laboratory of Chemical Theory and Mechanism, Chongqing University, Chongqing 401331, P. R. China.

[*]Corresponding author. E-mail: lanyu@cqu.edu.cn; lishijunzong@zzu.edu.cn; nlb@zzu.edu.cn

Table of Contents

1. Supplementary Methods 3
2. Supplementary Discussion.. 7
3. Supplementary Notes... 32
4. Supplementary References..155

1. Supplementary Methods

1.1 General information

All manipulations were carried out by standard Schlenk techniques. Unless otherwise stated, analytical grade solvents and commercially available reagents were used to conduct the reactions. LED irradiation was accomplished using the photochemical reactors (Supplementary Figure 1), whose wavelength is 404 – 499 nm (Supplementary Figure 2). Thin layer chromatography (TLC) employed glass 0.20 – 0.25 mm silica gel plates. Purification of products was accomplished using flash chromatography on silica gel. All the new compounds were characterized by ^{1}H NMR, ^{13}C NMR, ^{19}F NMR and ESI-HRMS. The known compounds were characterized by ^{1}H NMR, ^{13}C NMR, ^{19}F NMR and ESI-HRMS. NMR spectra were recorded on a Bruker 400 instrument operating at 400, 101, and 376 MHz for ^{1}H NMR, ^{13}C NMR and ^{19}F NMR respectively. Chemical shifts (δ) were reported in ppm, and coupling constants (*J*) are in Hertz (Hz). Data was reported in ppm using $CDCl_3$ (^{1}H NMR δ 7.26, ^{13}C NMR δ 77.16) or DMSO-d_6 (^{1}H NMR δ 2.50, ^{13}C NMR δ 39.52) as solvent unless otherwise specified. The following abbreviations were used to explain the multiplicities: s = singlet, d = doublet, t = triplet, q = quartet, m = multiplet. High-resolution mass data was recorded on mass spectrometers in the ESI-HRMS mode.

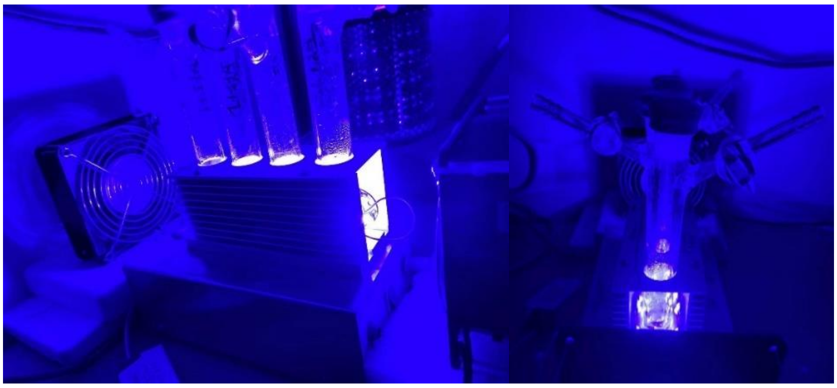

Supplementary Figure 1. Photochemical reactors.

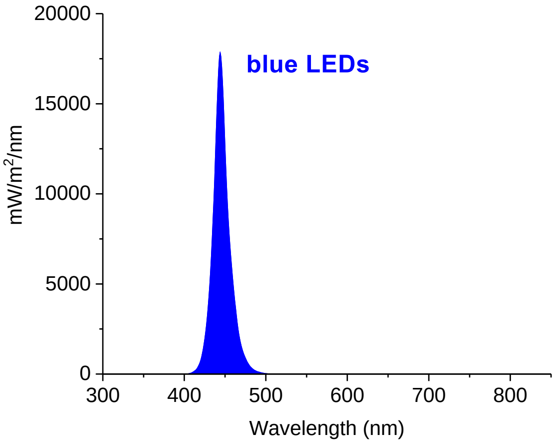

Supplementary Figure 2. Wavelength spectrum of photochemical reactors.

1.2 Preparation of substrates and catalysts

Methods of structural synthesis

To a 50 mL Schlenk tube, 4-dimethylaminopyridine (0.5 mmol), Et_3N (10.0 mmol) and alcohol (5.0 mmol) were dissolved in dichloromethane (12.0 mL). Acyl chloride (7.5 mmol) was added dropwise at 0 °C. The solution was stirred for 2 h at room temperature. Sat. $NaHCO_3$ aq. was poured into the reaction mixture. The organic layer was separated and extracted from water with EtOAc. The combined organic phase was washed with brine, dried over Na_2SO_4, filtered, and concentrated under vacuum. The residue was purified by silica gel column chromatography to afford the products[1].

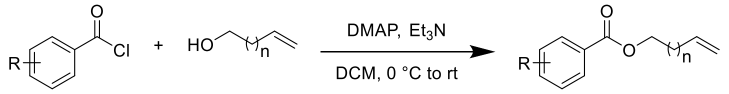

Supplementary Figure 3. Method A for the synthesis of non-activated alkenes.

To a 50 mL Schlenk tube, 4-dimethylaminopyridine (0.5 mmol), Et_3N (10.0 mmol) and alcohol (5.0 mmol) were dissolved in dichloromethane (12.0 mL). Sulfonyl chloride (7.5 mmol) was dropwise added at 0 °C. The reaction mixture was stirred for 2 h at room temperature. Sat. $NaHCO_3$ aq. was poured into the reaction mixture. The organic layer was separated and extracted from water with EtOAc. The combined organic phase was washed with brine, dried over Na_2SO_4, filtered, and concentrated under vacuum. The residue was purified by silica gel column chromatography to afford the products[1].

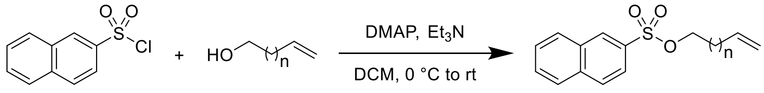

Supplementary Figure 4. Method B for the synthesis of non-activated alkenes.

To a 50 mL Schlenk tube, tetrabutylammonium iodide (0.5 mmol), Cs_2CO_3 (6.5 mmol) and phenol (6.0 mmol) were dissolved in dimethylformamide (12.0 mL). Alkenyl halide (5.0 mmol) was added and the mixture was stirred for 16 h at 50 °C. Diluted NaOH aq. was poured into the reaction mixture. The organic layer was separated and extracted from water with *tert*-Butyl methyl ether. The combined organic phase was washed with brine, dried over Na_2SO_4, filtered, and concentrated under vacuum. The residue was purified by silica gel column chromatography to afford the products[1].

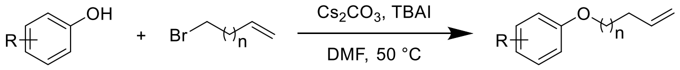

Supplementary Figure 5. Method C for the synthesis of non-activated alkenes.

To a 100 mL Schlenk tube, isoindoline-1, 3-dione (10.0 mmol), alkenyl halide (13.0 mmol) and K_2CO_3 (13.0 mmol) were dissolved in dimethylformamide (30.0 mL). The reaction mixture was stirred at room temperature, and the progress of the reaction was monitored by the TLC analysis. After 6 h the reaction was completed, as judged by TLC. The reaction mixture was slowly poured into water (100.0 mL). The aqueous phase was extracted with CH_2Cl_2 (5 × 20.0 mL). The organic extract was washed with water (3 × 40.0 mL), dried over Na_2SO_4, filtered, and concentrated under vacuum. The residue was purified by silica gel column chromatography to afford the products[2].

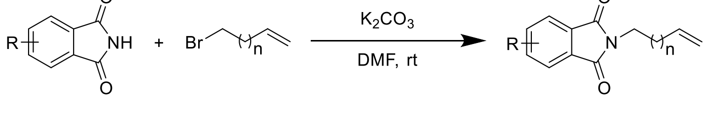

Supplementary Figure 6. Method D for the synthesis of non-activated alkenes.

The synthesis of $Fe(CF_3COO)_3$: To a 50 mL Schlenk tube, iron(III) chloride (5.0 mmol) and silver trifluoroacetate (15.0 mmol) were dissolved and reacted in dichloromethane (15.0 mL) for 3 h at room temperature. The precipitate AgCl was filtered and the residue was washed with acetone. The corresponding filtrate was concentrated under reduced pressure and the resulting product was dried

at 80 °C for 1 h. Iron(III) tri-fluoroacetate was obtained as red solid[3]. **ESI-HRMS** for $C_6F_9FeO_6Na^+$: 417.8793; found: 417.8811.

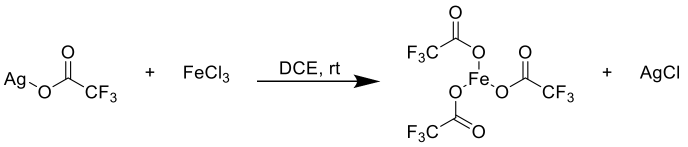

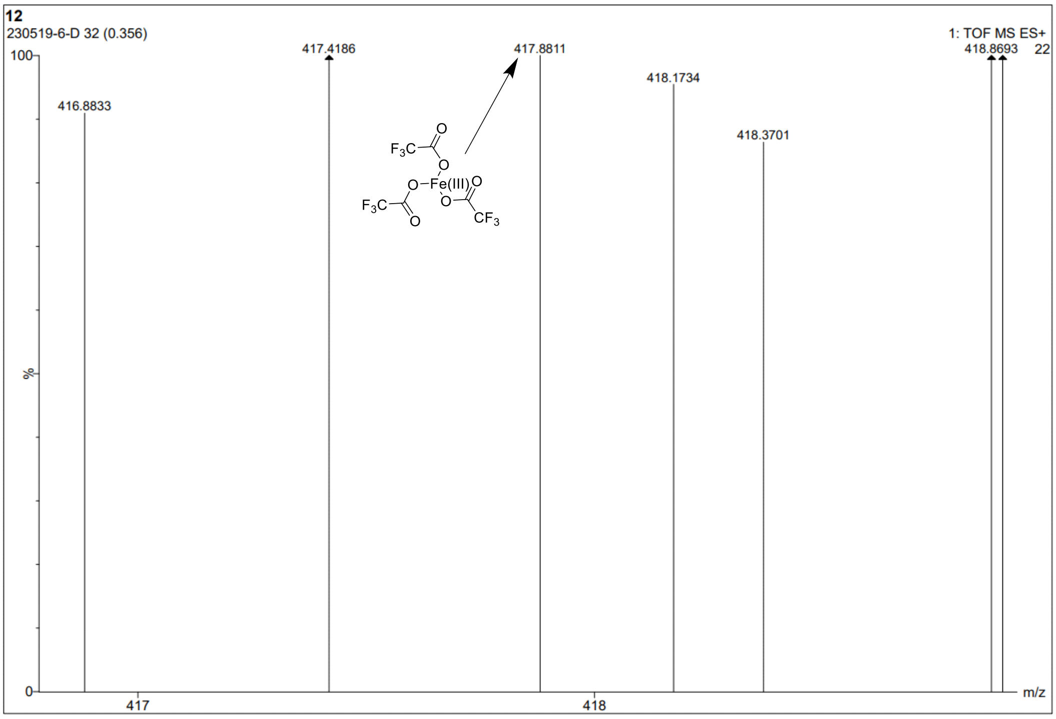

Supplementary Figure 7. Synthesis of $Fe(CF_3COO)_3$ and **ESI-HRMS** detection of $Fe(CF_3COO)_3$.

1.3 Experimental procedures for fluoro-polyhaloalkylation

General procedure for fluoro-polyhaloalkylation of alkenes: A 25 mL Schlenk flask equipped with a magnetic bar was charged with Fe(acac)$_3$ (7.1 mg, 0.02 mmol) and Selectfluor (106.3 mg, 0.3 mmol). The flask was evacuated and refilled with N_2 for three times. The vessel was then charged with extra dry CH_3CN (2.0 mL), alkene (0.2 mmol), $(C_nX_mCO)_2O$ (X: F or Cl) (1.0 or 1.4 mmol), oxydibenzene (17.0 mg, 0.1 mmol) and isopropanol (24.0 mg, 0.4 mmol). The reaction mixture was stirred under nitrogen atmosphere and irradiated by blue LEDs for 12 or 24 or 36 h. After completion of the reaction, CO_2 was detected by TCD-GC. Then the reaction system was quenched by triethylamine, diluted with EtOAc. After concentrated under vacuum, the resulting residue was purified by silica gel flash column chromatography to give the products.

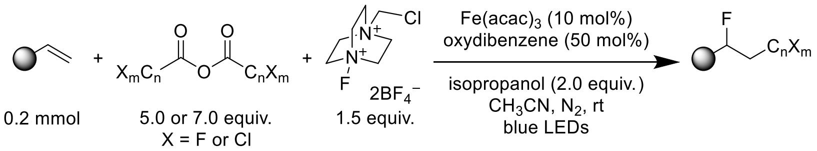

Supplementary Figure 8. Brønsted acid-unlocked iron LMCT photocatalysis for radical fluoro-polyhaloalkylation of alkenes.

General procedure for gram scale synthesis: A 100 mL Schlenk flask equipped with a magnetic bar was charged with Fe(acac)$_3$ (0.1766 g, 0.5 mmol) and Selectfluor (2.6570 g, 7.5 mmol). The flask was evacuated and refilled with N_2 for three times. The vessel was then charged with extra dry CH_3CN (50.0 mL), alkene (5.0 mmol), TFAA (5.2508 g, 25.0 mmol), oxydibenzene (0.4255 g, 2.5 mmol) and isopropanol (0.6010 g, 10.0 mmol). The reaction mixture was stirred under nitrogen atmosphere and irradiated by blue LEDs for 72 h. After completion of the reaction. Then the reaction system was quenched by triethylamine, diluted with EtOAc. After concentrated under vacuum, the resulting residue was purified by silica gel flash column chromatography to give the product.

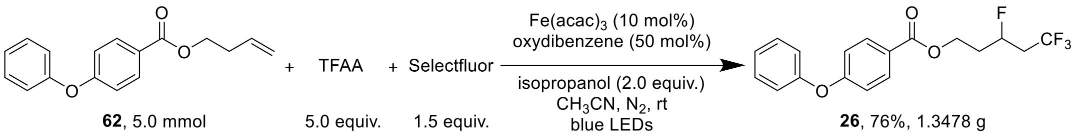

Supplementary Figure 9. Scale up reaction.

General procedure for TON experiment: A 100 mL Schlenk flask equipped with a magnetic bar was charged with Fe(acac)$_3$ (0.0021 g, 0.006 mmol) and Selectfluor (1.0628 g, 3.0 mmol). The flask was evacuated and refilled with N_2 for three times. The vessel was then charged with extra dry CH_3CN (20.0 mL), alkene (2.0 mmol), TFAA (2.1003 g, 10.0 mmol), oxydibenzene (0.1702 g, 1.0 mmol) and isopropanol (0.2406 g, 4.0 mmol). The reaction mixture was stirred under nitrogen atmosphere and irradiated by blue LEDs for 144 h. After completion of the reaction. Then the reaction system was quenched by triethylamine, diluted with EtOAc. After concentrated under vacuum, the resulting residue was purified by silica gel flash column chromatography to give the product.

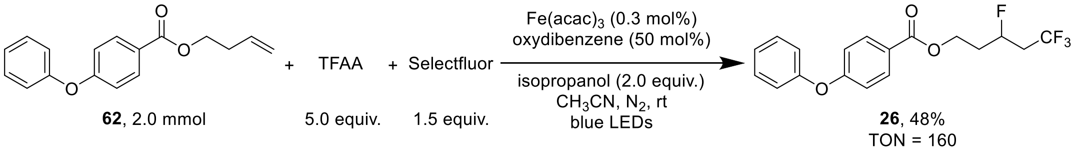

Supplementary Figure 10. TON experiment.

2. Supplementary Discussion

2.1 Investigation of conditions

Investigation of anisoles

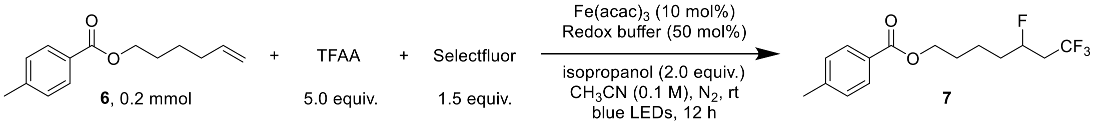

Entry	Redox buffer	**7**, Yield
1	anisole	47%
2	methyl 4-methoxybenzoate	43%
3	1-methoxynaphthalene	Trace
4	*tert*-butoxybenzene	15%

Supplementary Table 1. Results of investigation of anisoles. Yields were determined by GC analysis of the crude reaction mixture, using biphenyl as an internal standard.

Investigation of iron salts

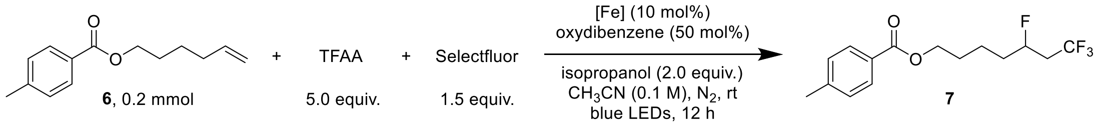

Entry	[Fe]	**7**, Yield
1	$FeCl_3$	53%
2	$Fe(CF_3COO)_3$	67%
3	$Fe(OH)(OAc)_2$	55%
4	$Fe(NO_3)_3{\cdot}9H_2O$	43%
5	$Fe(acac)_2$	64%

Supplementary Table 2. Results of investigation of iron salts. Yields were determined by GC analysis of the crude reaction mixture, using biphenyl as an internal standard.

Investigation of solvents

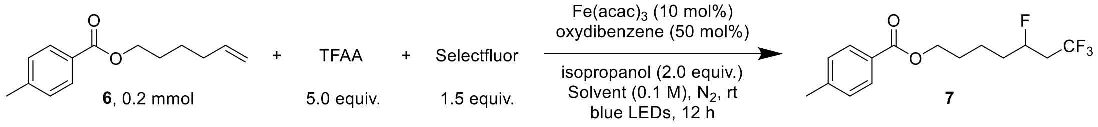

Entry	Solvent	**7**, Yield
1	CH_3CN	70%
2	Acetone	N.D.
3	DCE	N.D.
4	DCM	N.D.
5	EA	42%
6	DMF	N.D.
7	DMSO	N.D.
8	THF	N.D.
9	CCl_3CN	N.D.
10	$(CH_3)_3CCN$	16%
11	N-methyl-2-pyrrolidone	N.D.
12	1,4-Dioxane	N.D.
13	1,2-Dimethoxyethane	N.D.
14	2-Methoxy-2-methylpropane	N.D.

Supplementary Table 3. Results of investigation of solvents. Yields were determined by GC analysis of the crude reaction mixture, using biphenyl as an internal standard.

Deviation from standard condition

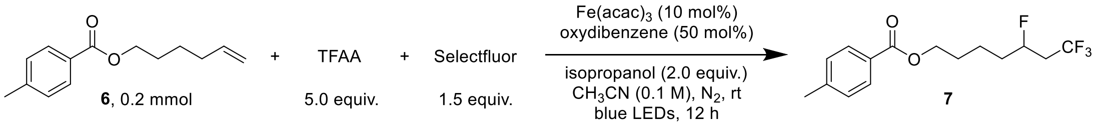

Entry	Deviation from standard condition	**7**, Yield
1	None	70%
2	CF_3COONa instead of TFAA	N.D.
3	TFA instead of TFAA	31%
4	NFSI as [F] source	28%
5	NFPY-OTf as [F] source	N.D.
6	Green LEDs	N.D.
7	Heat to 60 °C or 80 °C (w/o light)	N.D.

Supplementary Table 4. Results of deviation from standard condition. Yields were determined by GC analysis of the crude reaction mixture, using biphenyl as an internal standard.

Controlled experiments for the fluorotrifluoromethylation of alkenes

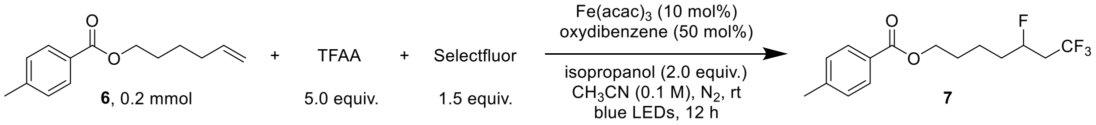

Entry	Deviation from the standard condition	**6**, Recovery	**7**, Yield
1	none	trace	70%
2	w/o light	85%	N.D.
3	w/o $Fe(acac)_3$	65%	N.D.
4	w/o oxydibenzene	0	15%
5	w/o light & oxydibenzene	90%	N.D.
6	w/o $Fe(acac)_3$ & oxydibenzene	87%	N.D.

Supplementary Table 5. Results of controlled experiments. Yields were determined by GC analysis of the crude reaction mixture, using biphenyl as an internal standard.

Control experiments for the stoichiometric experiments

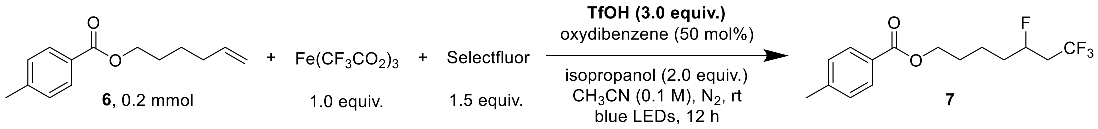

Entry	Deviation from the standard conditions	**6**, Recovery	**7**, Yield
1	/	10%	41%
2	w/o isopropanol	12%	18%
3	w/o oxydibenzene	0	21%
4	w/o oxydibenzene & isopropanol	0	11%
5	lithium isopropoxide instead of isopropanol	69%	N.D.

Supplementary Table 6. Results of stoichiometric experiments. Yields were determined by GC analysis of the crude reaction mixture, using biphenyl as an internal standard.

2.2 Mechanistic studies

General procedure for gas experimental analysis: A 25 mL Schlenk flask equipped with a magnetic bar was charged with $Fe(acac)_3$ (7.1 mg, 0.02 mmol) and Selectfluor (106.3 mg, 0.3 mmol). The flask was evacuated and refilled with N_2 for three times. The vessel was then charged with extra dry CH_3CN (2.0 mL), alkene (0.2 mmol), TFAA (210.0 mg, 1.0 mmol), oxydibenzene (17.0 mg, 0.1 mmol) and isopropanol (24.0 mg, 0.4 mmol). The reaction mixture was stirred under nitrogen atmosphere and irradiated by blue LEDs for 12 h. After completion of the reaction, CO_2 was detected by TCD-GC.

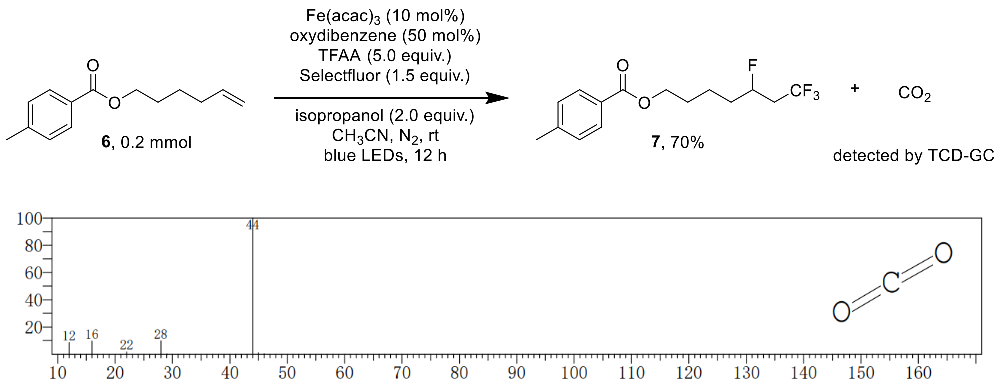

Supplementary Figure 11. Detection of CO_2.

General procedure for radical trapping experiments: A 25 mL Schlenk flask equipped with a magnetic bar was charged with $Fe(acac)_3$ (7.1 mg, 0.02 mmol), Selectfluor (106.3 mg, 0.3 mmol) and 2,2,6,6-tetramethyl-1-piperidinyloxy (**TEMPO**) (62.5 mg, 0.4 mmol) or butylated hydroxytoluene (**BHT**) (88.1 mg, 0.4 mmol). The flask was evacuated and refilled with N_2 for three times. The vessel was then charged with extra dry CH_3CN (2.0 mL), alkene (0.2 mmol), TFAA (210.0 mg, 1.0 mmol), oxydibenzene (17.0 mg, 0.1 mmol) and isopropanol (24.0 mg, 0.4 mmol). The reaction mixture was stirred under nitrogen atmosphere and irradiated by blue LEDs for 12 h. After completion of the reaction. Then the reaction system was quenched by triethylamine, diluted with EtOAc. The reaction results were monitored by TLC and GC. Yields were determined by GC analysis of the crude reaction mixture, using biphenyl as an internal standard. No desired product **7** was detected.

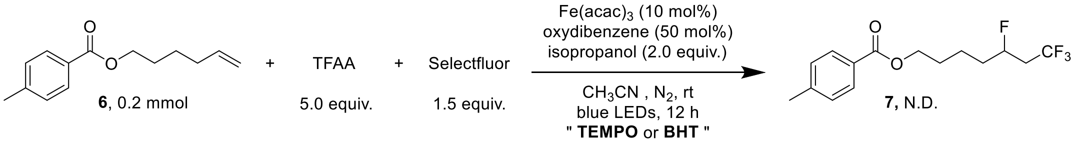

Supplementary Figure 12. Radical inhibition experiments.

General Procedure for ^{19}F NMR experiments: TFAA (0.5 mmol, 105.0 mg), isopropanol (0.4 mmol, 24.0 mg), trifluoroacetophenone (0.1 mmol, 17.4 mg) as internal standard, and CH_3CN (0.5 mL) were added in a vial, which was generated esters **1** and TFA **2**, the corresponding product was detected by ^{19}F NMR.

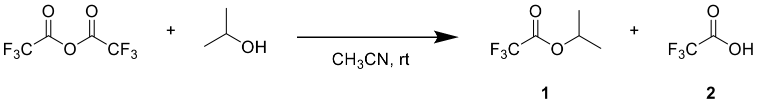

Supplementary Figure 13. TFAA and alcohol *in situ* generating Brønsted acid.

Reaction conditions:

(1) TFAA (0.5 mmol) + internal standard (0.1 mmol) + CH_3CN (0.5 mL).

(2) TFAA (0.5 mmol) + isopropanol (0.4 mmol) + internal standard (0.1 mmol) + CH_3CN (0.5 mL).

(3) isopropyl 2,2,2-trifluoroacetate (0.5 mmol) + internal standard (0.1 mmol) + CH_3CN (0.5 mL).

(4) TFA (0.5 mmol) + internal standard (0.1 mmol) + CH_3CN (0.5 mL).

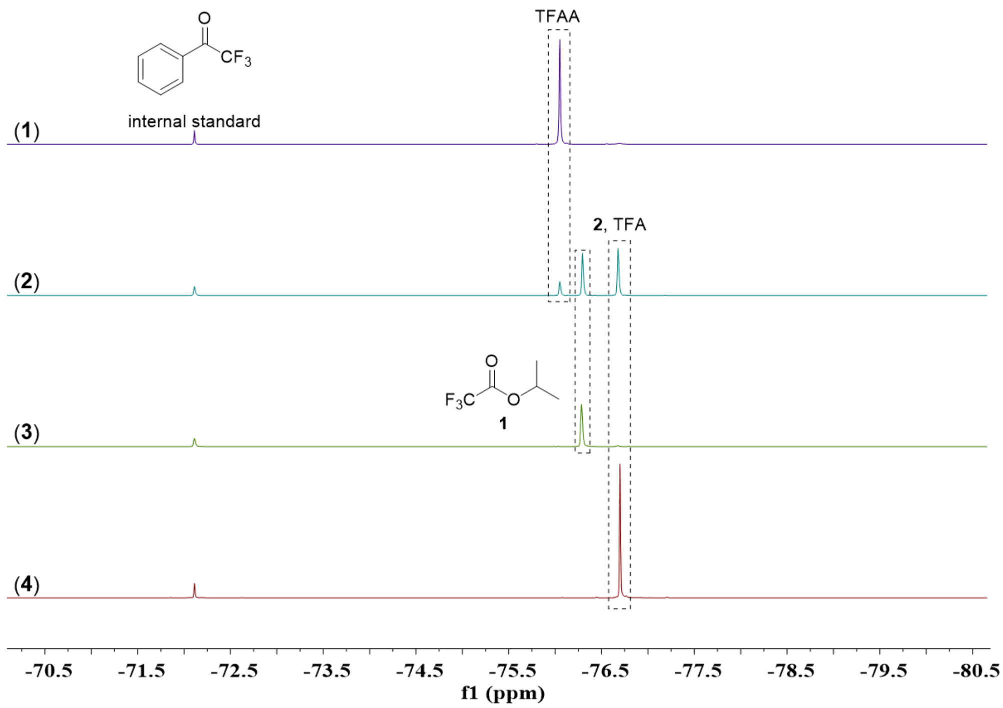

Supplementary Figure 14. ^{19}F NMR analysis of TFAA and alcohol.

The advantage of using ($(CF_3CO)_2O$) and alcohol to *in situ* generate Brønsted acid: A 25 mL Schlenk flask equipped with a magnetic bar was charged with $Fe(acac)_3$ (7.1 mg, 0.02 mmol) and Selectfluor (106.3 mg, 0.3 mmol). The flask was evacuated and refilled with N_2 for three times. The vessel was then charged with extra dry CH_3CN (2.0 mL), alkene (0.2 mmol), TFAA (210.0 mg, 1.0 mmol), oxydibenzene (17.0 mg, 0.1 mmol) and isopropanol (24.0 mg, 0.4 mmol). The reaction mixture was stirred under nitrogen atmosphere and irradiated by blue LEDs. After completion of the reaction. Then the reaction system was quenched by triethylamine, diluted with EtOAc. Yields were determined by GC analysis of the crude reaction mixture, using biphenyl as an internal standard (Supplementary Figure 15 line **i**). In the absence of isopaopanol under the standard condition (Supplementary Figure 15 line **ii**). When TFAA and isopropanol was replaced into TFA (Supplementary Figure 15 line **iii**). We found that line **i** works better than line **ii** and line **iii**.

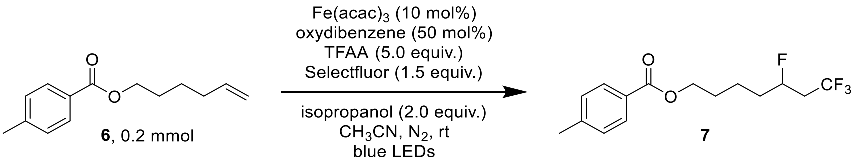

Line	Deviation from the standard condition
i	/ (standard condition)
ii	w/o isopropanol
iii	TFA instead of TFAA and isopropanol

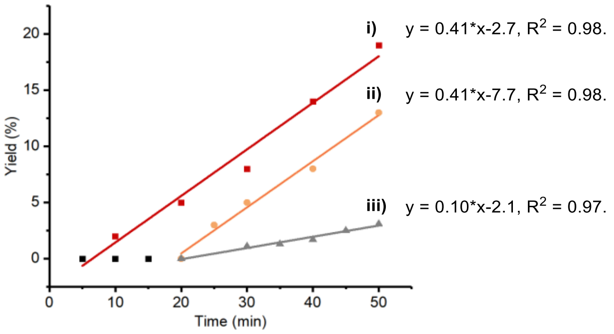

Supplementary Figure 15. Initial reaction rates with TFAA or TFA as CF_3 source. Yields were determined by GC analysis of the crude reaction mixture, using biphenyl as an internal standard.

General procedure for aminotrifluoromethylation and fluoroamination of alkenes: A 25 mL Schlenk flask equipped with a magnetic bar was charged with $Fe(acac)_3$ (7.1 mg, 0.02 mmol) and

Selectfluor (106.3 mg, 0.3 mmol). The flask was evacuated and refilled with N_2 for three times. The vessel was then charged with extra dry CH_3CN (2.0 mL), alkene (0.2 mmol), TFAA (1.0 mmol) and isopropanol (24.0 mg, 0.4 mmol). The reaction mixture was stirred under nitrogen atmosphere and irradiated by blue LEDs for 12 h. After completion of the reaction. Then the reaction system was quenched by triethylamine, diluted with EtOAc. After concentrated under vacuum, the resulting residue was purified by silica gel flash column chromatography to give the product and by-products. Based on previous reports[4], the role of oxydibenzene thus might serve as the redox buffer to timely quench the electrophilic *N* radical cation from the Selectfluor.

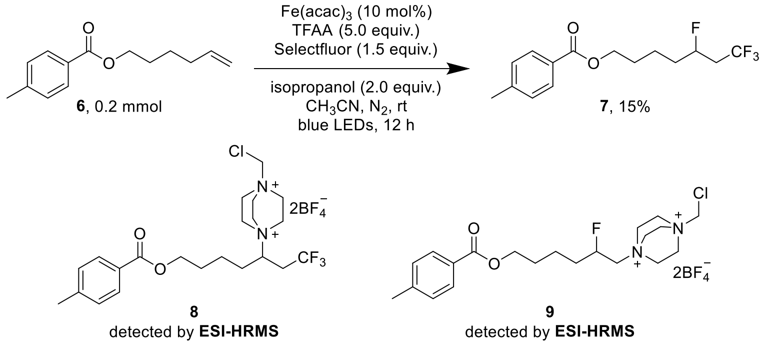

Supplementary Figure 16. Study of aminotrifluoromethylation and fluoroamination products.

The role of the oxydibenzene in the reaction system: A 25 mL Schlenk flask equipped with a magnetic bar was charged with $Fe(acac)_3$ (7.1 mg, 0.02 mmol) and NFSI (94.6 mg, 0.3 mmol). The flask was evacuated and refilled with N_2 for three times. The vessel was then charged with extra dry CH_3CN (2.0 mL), alkene (0.2 mmol), TFAA (1.0 mmol), oxydibenzene (17.0 mg, 0.1 mmol) and isopropanol (24.0 mg, 0.4 mmol). The reaction mixture was stirred under nitrogen atmosphere and irradiated by blue LEDs for 12 h. After completion of the reaction. Then the reaction system was quenched by triethylamine, diluted with EtOAc. After concentrated under vacuum, the resulting residue was purified by silica gel flash column chromatography to give the amination product, detected by **NMR**.

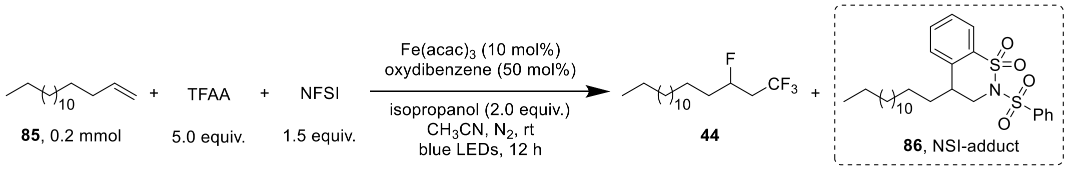

Entry	Deviation from standard conditions	**85,** Recovery	**44**, Yield	**86**, NSI-adduct
1	/	57%	13%	3%
2	w/o oxydibenzene	26%	7%	25%

Supplementary Figure 17. Study of NFSI as fluorine source.

ESI-HRMS detection

ESI-HRMS for $C_{16}H_{10}F_6O_4Na^+$ (**3**): 403.0375; found: 403.0388.

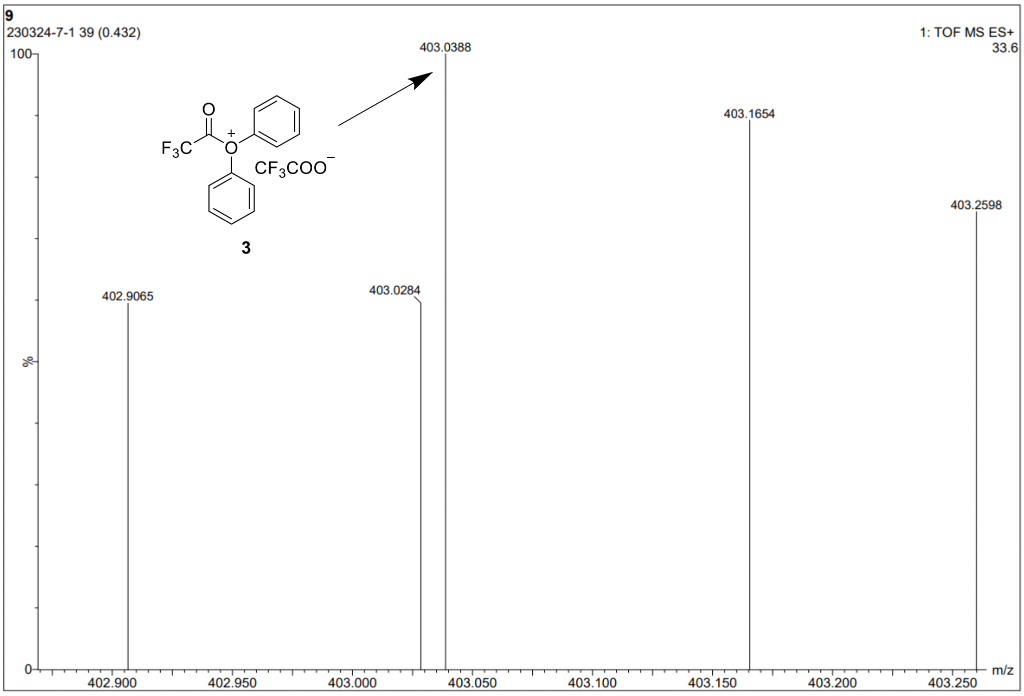

Supplementary Figure 18. **ESI-HRMS** detection of **3**.

ESI-HRMS for $C_6H_6F_6NO_3Na^+$ (**4**): 273.9909; found: 273.9912.

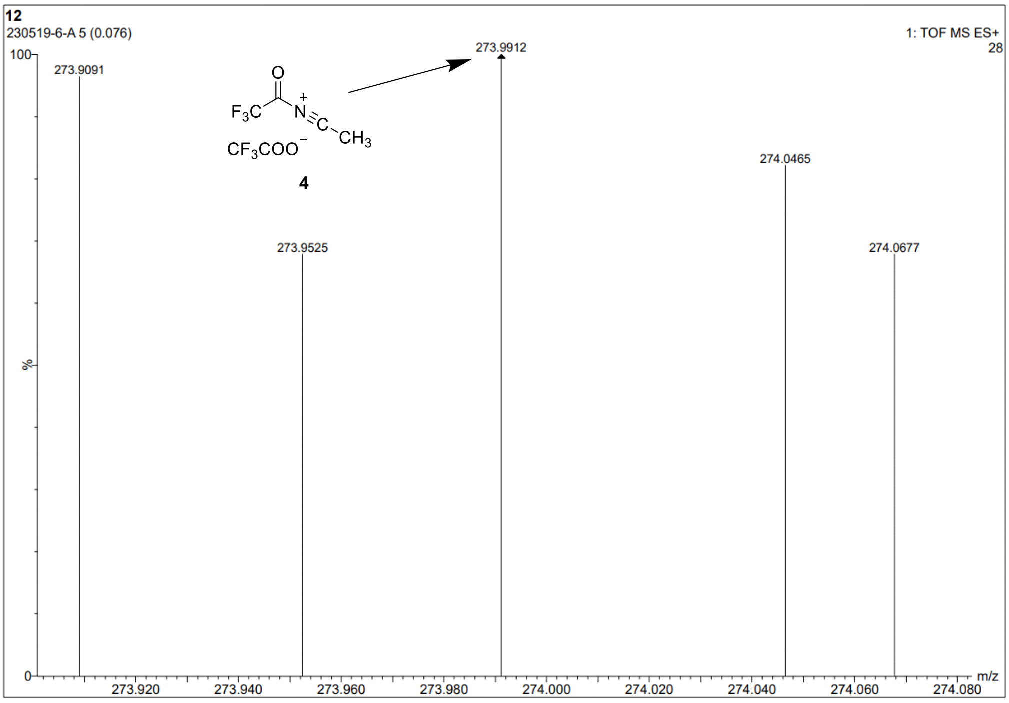

Supplementary Figure 19. **ESI-HRMS** detection of **4**.

ESI-HRMS for $C_4H_2F_3NOH^+$ (**5**): 138.0161; found: 138.0169.

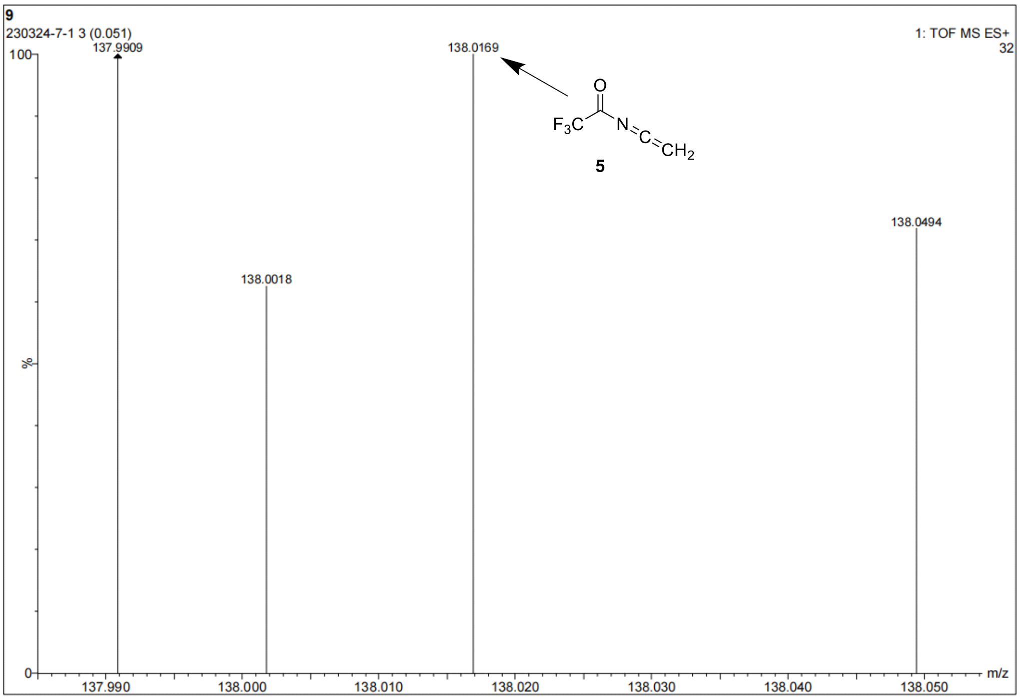

Supplementary Figure 20. **ESI-HRMS** detection of **5**.

ESI-HRMS for $C_{22}H_{32}B_2ClF_{11}N_2O_2H^+$ (**8**): 623.2236; found: 623.2227.

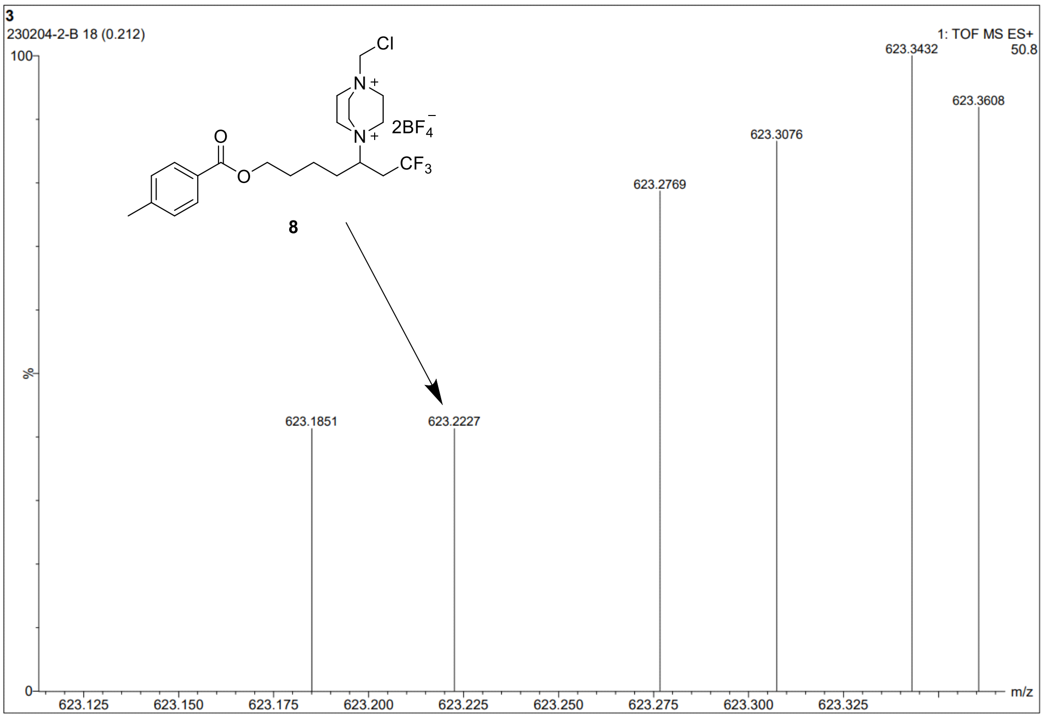

Supplementary Figure 21. **ESI-HRMS** detection of **8**.

ESI-HRMS for $C_{21}H_{32}B_2ClF_9N_2O_2H^+$ (**9**): 573.2267; found: 573.2256.

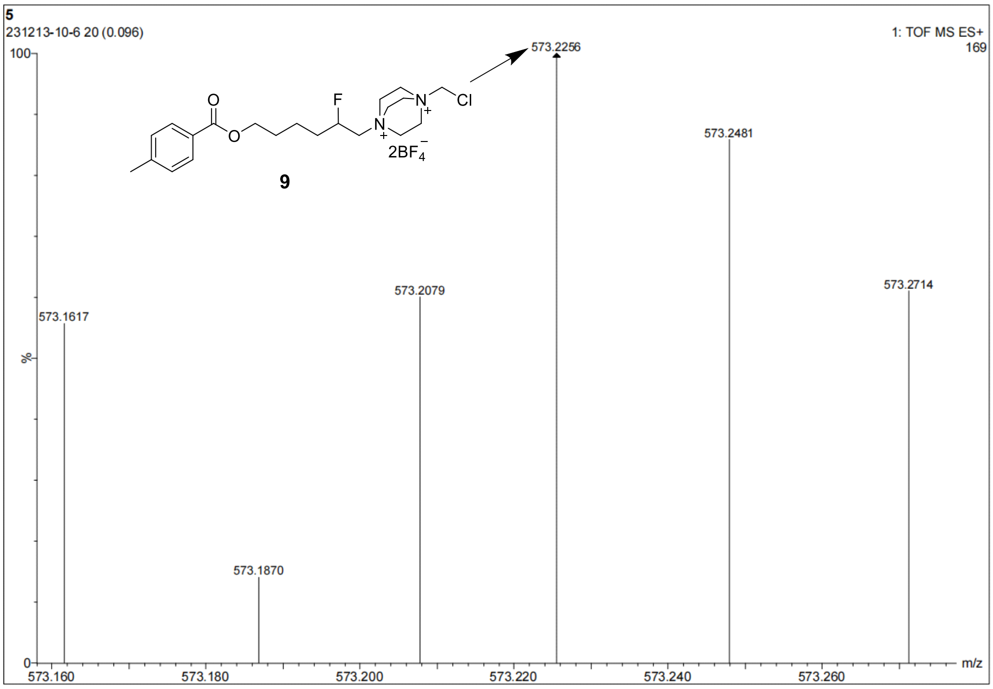

Supplementary Figure 22. **ESI-HRMS** detection of **9**.

ESI-HRMS for $C_{28}H_{41}NO_4S_2Na^+$ (**86**): 542.2369; found: 542.2381.

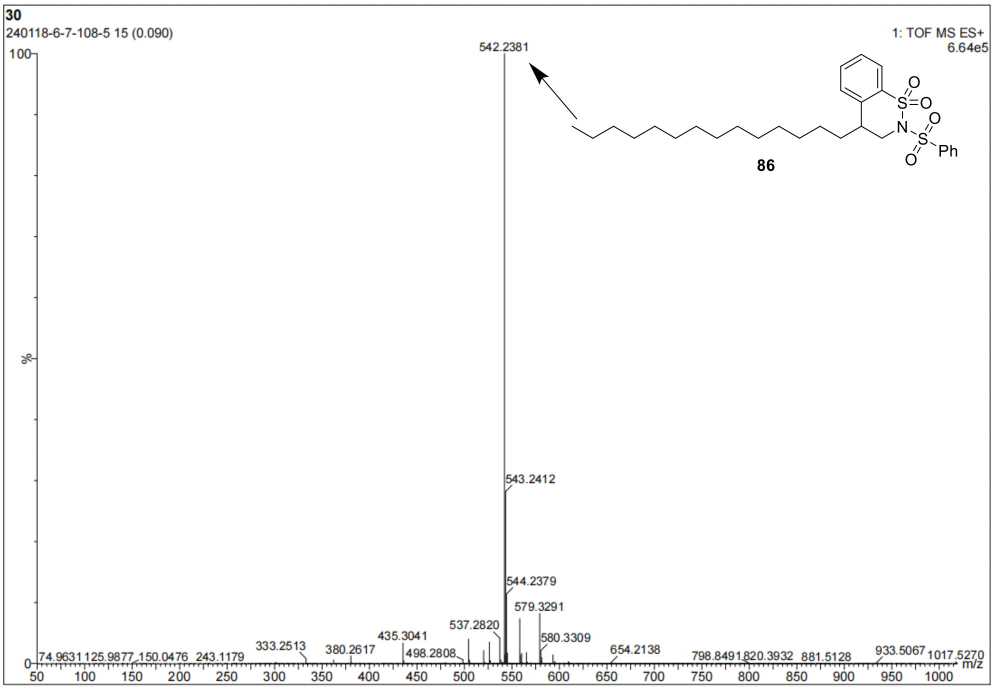

Supplementary Figure 23. **ESI-HRMS** detection of **86**.

The study of the photoactive species (12)

a) $Fe(CF_3CO_2)_3$ + TFA (1:3) in CH_3CN, detected by **ESI-HRMS**, found: 477.9503, and 591.9427, consistent with theoretically exact mass of **12**: 477.9504, and **12** + [$CF_3CO_2^-$] + [H^+]: 591.9433, respectively.

b) $Fe(CF_3CO_2)_3$ + TfOH (1:3) in CH_3CN, detected by **ESI-HRMS**, found: 477.9493, and 613.9250, consistent with theoretically exact mass of **12**: 477.9504, and **12** + [$CF_3CO_2^-$] + [Na^+]: 613.9252,

respectively.

c) Fe(acac)$_3$ + TFAA + isopropanol (1:5:2) in CH_3CN, detected by **ESI-HRMS**, found: 477.9508, and 613.9256, consistent with theoretically exact mass of **12**: 477.9504, and **12** + [$CF_3CO_2^-$] + [Na^+]: 613.9252, respectively.

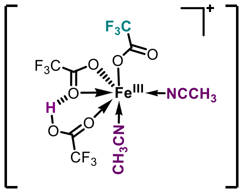

12: $C_{10}H_7F_9FeN_2O_6^+$, Exact mass: 477.9504.

Supplementary Figure 24. Study of the photoactive species.

The study of the Fe-F species (87)

a) $Fe(CF_3CO_2)_3$ + TFA + TBAF (1:3:3) in CH_3CN, detected by **ESI-HRMS**, found: 383.9360, and 519.9398, consistent with theoretically exact mass of **87**: 383.9638, and **87** + [$CF_3CO_2^-$] + [Na^+]: 519.9386, respectively.

b) $Fe(CF_3CO_2)_3$ + TfOH + TBAF (1:3:3) in CH_3CN, detected by **ESI-HRMS**, found: 383.9305, and 497.9581, consistent with theoretically exact mass of **87**: 383.9638, and **87** + [$CF_3CO_2^-$] + [H^+]: 497.9567, respectively.

c) Fe(acac)$_3$ + TFAA + isopropanol + TBAF (1:5:2:3) in CH_3CN, detected by **ESI-HRMS**, found: 519.9399, consistent with theoretically exact mass of **87** + [$CF_3CO_2^-$] + [Na^+]: 519.9386.

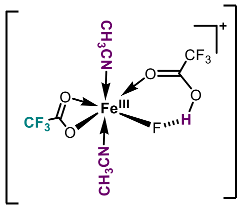

87: $C_8H_7F_7FeN_2O_4^+$, Exact mass: 383.9638.

Supplementary Figure 25. Study of the photoactive Fe-F species.

General procedure for UV-vis experiments:

1. (1) $FeCl_3$:TFAA:isopropanol:dibenzene (1:5:2:0.5).

(2) $Fe(CF_3COO)_3$ [0.25 mM].

(3) $Fe(CF_3COO)_3$:TfOH (1:30).

CH_3CN as solvent, the mixture was stirred 30 min in darkness, [iron] salt 0.25 mM.

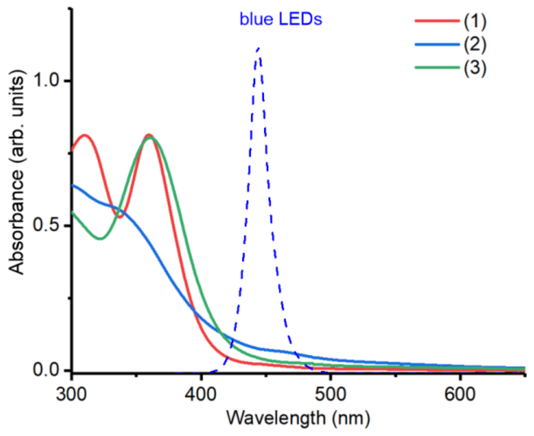

Supplementary Figure 26. Absorbance profiles of different [iron] salt.

2. (1) $Fe(CF_3CO_2)_3$ under darkness [0.25 mM].

(2) $Fe(acac)_3$ under darkness [0.25 mM].

(3) $Fe(acac)_3$:TFA (1:3) under darkness.

(4) $Fe(acac)_3$:TFA (1:3) under blue LEDs.

CH_3CN as solvent, the mixture was stirred 30 min, [iron] salt 0.25 mM.

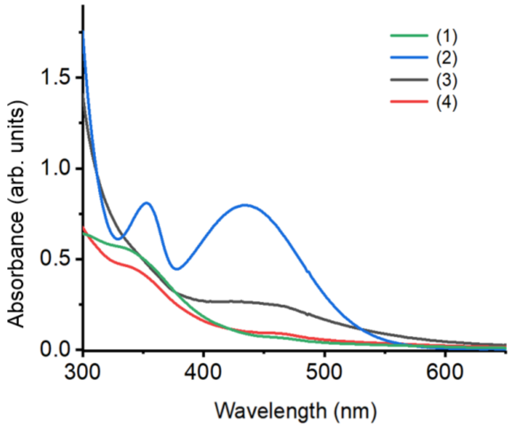

Supplementary Figure 27. Absorbance profiles of [iron] salt and Brønsted acid.

3. (1) $Fe(CF_3CO_2)_3$ under darkness [0.25 mM].

(2) $Fe(CF_3CO_2)_3$:CF_3CO_2Na (1:3) under darkness.

(3) $Fe(CF_3CO_2)_3$:CF_3CO_2Na:TfOH (1:3:6) under darkness.

(4) $Fe(CF_3CO_2)_3$:CF_3CO_2Na:TfOH (1:3:6) under blue LEDs irradiation.

CH_3CN as solvent, the mixture was stirred 30 min, [iron] salt 0.25 mM.

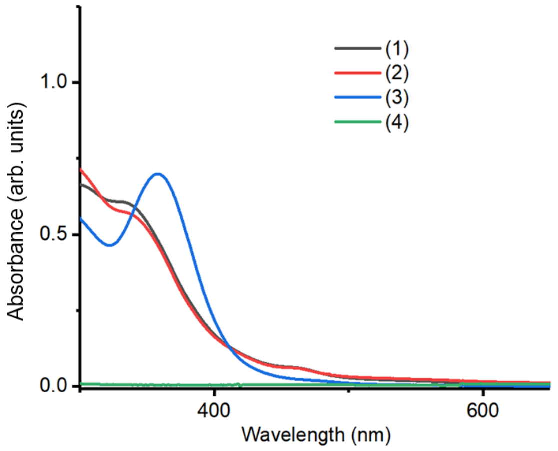

Supplementary Figure 28. Absorbance profiles of $Fe(CF_3CO_2)_3$ and CF_3CO_2Na.

4. The UV-vis monitorization of the oxidation of Fe(II) to Fe(III)

(1) $Fe(CF_3CO_2)_3$ [0.25 mM].

(2) $Fe(CF_3CO_2)_3$ + TfOH (1:30).

(3) $Fe(CF_3CO_2)_3$ + TfOH (1:30) under blue LEDs irradiation.

(4) $Fe(CF_3CO_2)_3$ + TfOH under blue LEDs irradiation, then, Selectfluor (1:30:15) added.

(5) $Fe(CF_3CO_2)_3$ + TfOH under blue LEDs irradiation, then, Selectfluor + oxydibenzene (1:30:15:5) added.

(6) $Fe(CF_3CO_2)_3$ + TfOH under blue LEDs irradiation, then, CF_3CO_2Na + Selectfluor + oxydibenzene (1:30:10:15:5) added.

CH_3CN as solvent, the mixture was stirred 30 min, [iron] salt 0.25 mM.

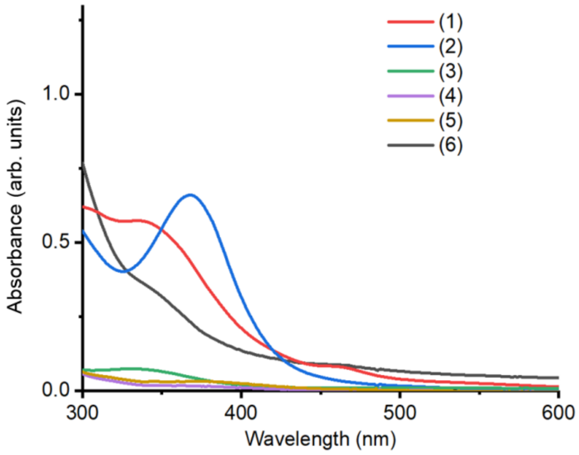

Supplementary Figure 29. UV-vis monitorization of the oxidation of Fe(II) to Fe(III).

General procedure for Brønsted acid loading experiments: A 25 mL Schlenk flask equipped with a magnetic bar was charged with $Fe(CF_3CO_2)_3$ (78.8 mg, 0.2 mmol) and Selectfluor (106.3 mg, 0.3 mmol). The flask was evacuated and refilled with N_2 for three times. The vessel was then charged with extra dry CH_3CN (2.0 mL), alkene (0.2 mmol), oxydibenzene (17.0 mg, 0.1 mmol), isopropanol (24.0 mg, 0.4 mmol) and TfOH (0.2 mmol, 0.4 mmol, 0.6 mmol, 0.8 mmol, 1.0 mmol). The reaction mixture was stirred under nitrogen atmosphere and irradiated by blue LEDs. After completion of the reaction. Then the reaction system was quenched by triethylamine, diluted with EtOAc. Yields were determined by GC analysis of the crude reaction mixture, using biphenyl as an internal standard.

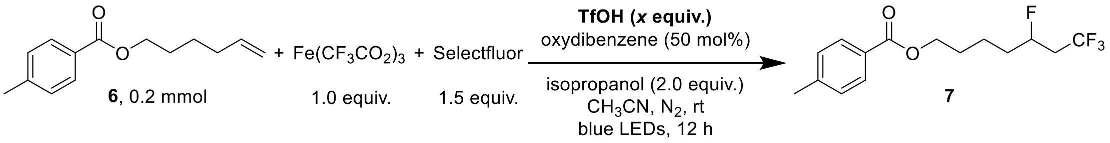

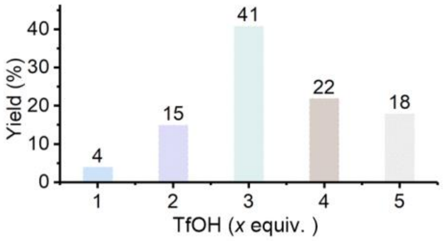

Supplementary Figure 30. Brønsted acid loading experiments.

General procedure for light on/off experiments: A 25 mL Schlenk flask equipped with a magnetic bar was charged with $Fe(acac)_3$ (7.1 mg, 0.02 mmol) and Selectfluor (106.3 mg, 0.3 mmol). The flask was evacuated and refilled with N_2 for three times. The vessel was then charged with extra dry CH_3CN (2.0 mL), alkene (0.2 mmol), TFAA (210.0 mg, 1.0 mmol), oxydibenzene (17.0 mg, 0.1 mmol) and isopropanol (24.0 mg, 0.4 mmol). The reaction mixture was stirred under nitrogen atmosphere and irradiated by blue LEDs. After completion of the reaction. Then the reaction system was quenched by triethylamine, diluted with EtOAc. Yields were determined by GC analysis of the crude reaction mixture, using biphenyl as an internal standard. Observed product **7** formation occurred only during periods of light irradiation, which ruled out a radical chain mechanism.

General procedure for kinetic curve of $Fe(acac)_3$: A 25 mL Schlenk flask equipped with a magnetic bar was charged with $Fe(acac)_3$ (0.001 mmol, 0.002 mmol, 0.003 mmol, 0.004 mmol, 0.005 mmol, 0.006 mmol) and Selectfluor (106.3 mg, 0.3 mmol). The flask was evacuated and

refilled with N_2 for three times. The vessel was then charged with extra dry CH_3CN (2.0 mL), alkene (0.2 mmol), TFAA (210.0 mg, 1.0 mmol), oxydibenzene (17.0 mg, 0.1 mmol) and isopropanol (24.0 mg, 0.4 mmol). The reaction mixture was stirred under nitrogen atmosphere and irradiated by blue LEDs for 1.5 h. After completion of the reaction. Then the reaction system was quenched by triethylamine, diluted with EtOAc. Yields were determined by GC analysis of the crude reaction mixture, using biphenyl as an internal standard.

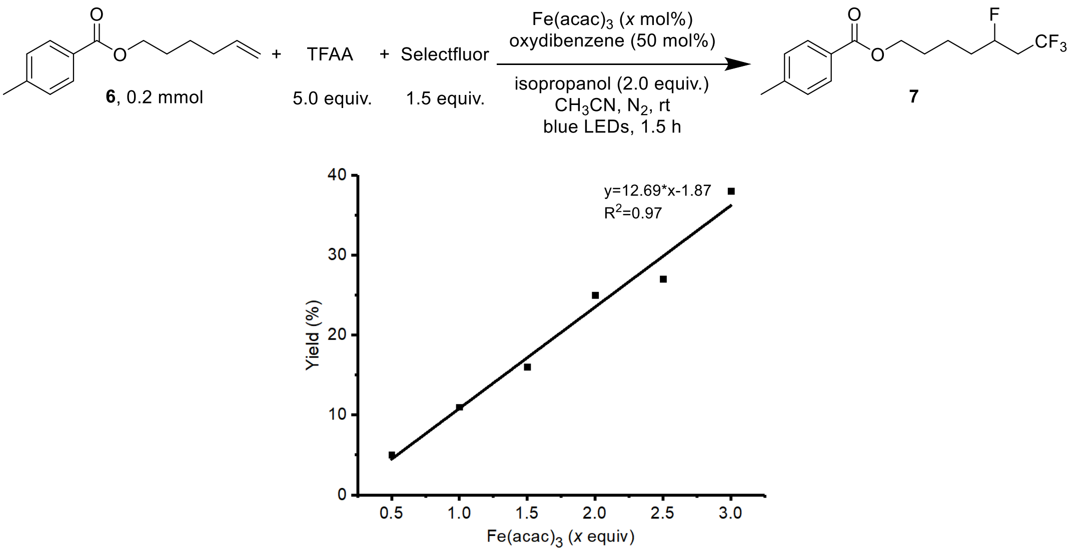

Supplementary Figure 31. Kinetic studies for $Fe(acac)_3$.

General procedure for kinetic curve of TFAA: A 25 mL Schlenk flask equipped with a magnetic bar was charged with $Fe(acac)_3$ (7.1 mg, 0.02 mmol) and Selectfluor (106.3 mg, 0.3 mmol). The flask was evacuated and refilled with N_2 for three times. The vessel was then charged with extra dry CH_3CN (2.0 mL), alkene (0.2 mmol), TFAA (0.4 mmol, 0.45 mmol, 0.5 mmol, 0.55 mmol, 0.6 mmol), oxydibenzene (17.0 mg, 0.1 mmol) and isopropanol (24.0 mg, 0.4 mmol). The reaction mixture was stirred under nitrogen atmosphere and irradiated by blue LEDs for 1.5 h. After completion of the reaction. Then the reaction system was quenched by triethylamine, diluted with EtOAc. Yields were determined by GC analysis of the crude reaction mixture, using biphenyl as an internal standard.

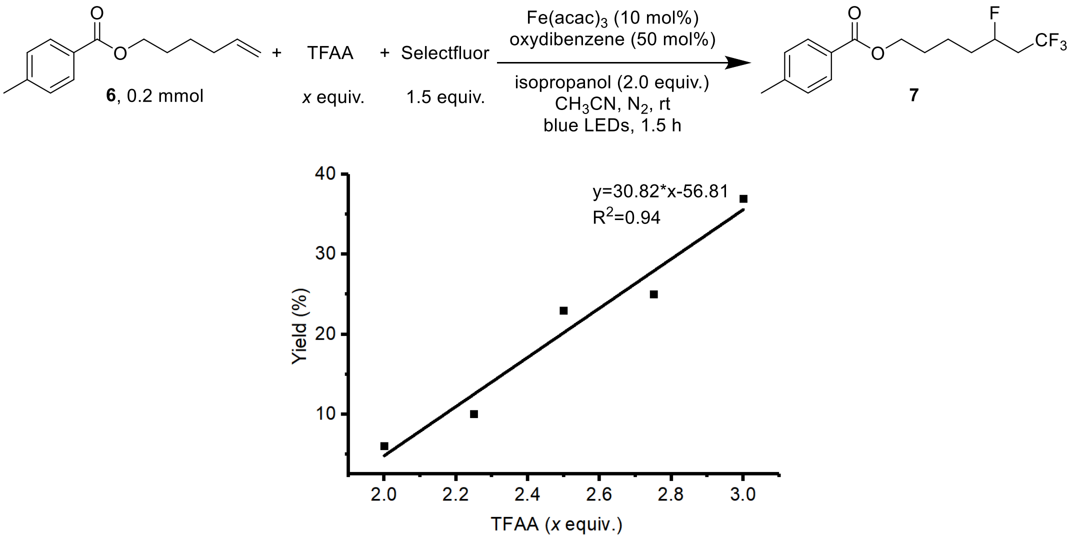

Supplementary Figure 32. Kinetic studies for TFAA.

General procedure for acetonitrile loading experiments: A 25 mL Schlenk flask equipped with a magnetic bar was charged with $Fe(acac)_3$ (7.1 mg, 0.02 mmol) and Selectfluor (106.3 mg, 0.3 mmol). The flask was evacuated and refilled with N_2 for three times. The vessel was then charged

with extra dry DCE (2.0 mL), alkene (0.2 mmol), TFAA (1.0 mmol), oxydibenzene (17.0 mg, 0.1 mmol), CH_3CN (0 mmol, 0.4 mmol, 0.12 mmol, 2.0 mmol) and isopropanol (24.0 mg, 0.4 mmol). The reaction mixture was stirred under nitrogen atmosphere and irradiated by blue LEDs for 12 h. After completion of the reaction. Then the reaction system was quenched by triethylamine, diluted with EtOAc. Yields were determined by GC analysis of the crude reaction mixture, using biphenyl as an internal standard.

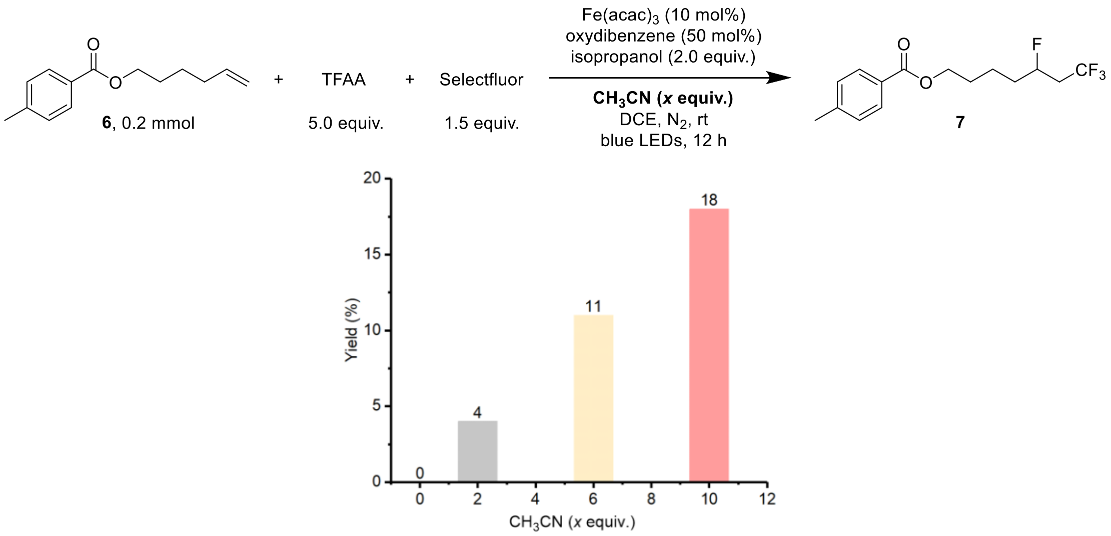

Supplementary Figure 33. Acetonitrile loading experiments

2.3 Density Functionl Theory calculation

Computational Details

All the calculation have been performed using the Gaussian Program (Gaussian 16)[5]. The geometry optimization was conducted with the B3LYP functional and standard 6-31G(d, p) basis set. The solvent effect was considered by IEFPCM[6,7]. Harmonic vibrational frequency calculations were performed for all stationary points to confirm whether they are local minima or transition state structures and to derive the thermochemical corrections for the enthalpies and free energies. Time-dependent density functional theory (TD-DFT) calculations were conducted at same level within the adiabatic approximation to predict the excitation energies[8,9].

The possible light-harvesting species in the reaction

According to the experimental observations, the possible light-harvesting species was calculated by DFT. The free energy for **12** generation is lower than that of other possible structures in Supplementary Figure 34.

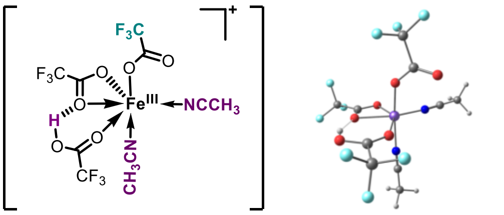

12 (0.0 kcal/mol)

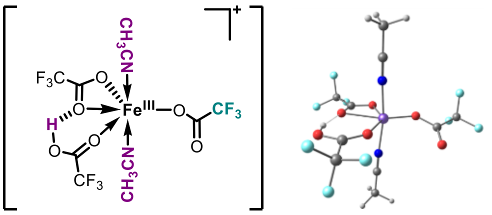

88 (0.3 kcal/mol)

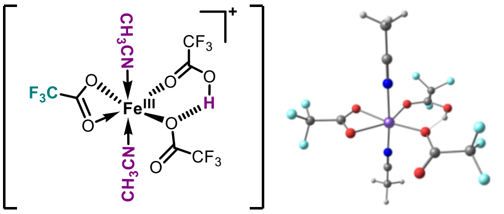

89 (1.2 kcal/mol)

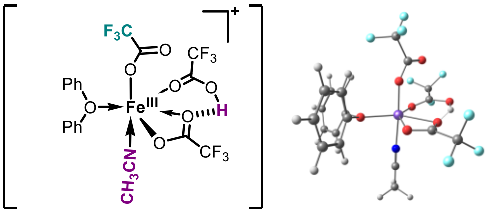

90 (11.2 kcal/mol)

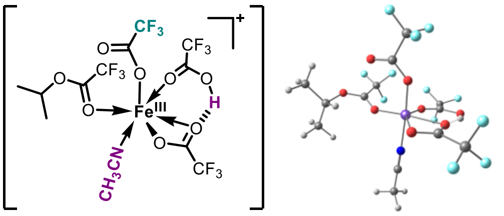

91 (7.0 kcal/mol)

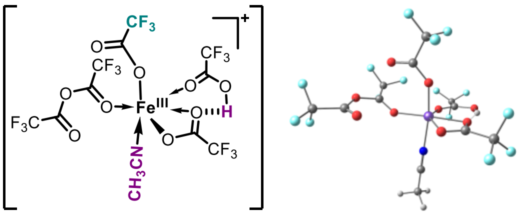

92 (11.0 kcal/mol)

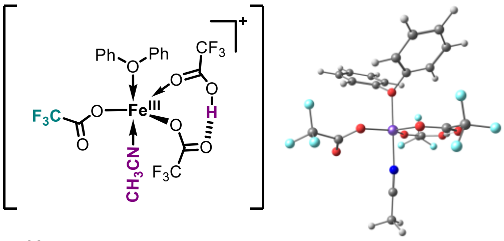

93 (7.7 kcal/mol)

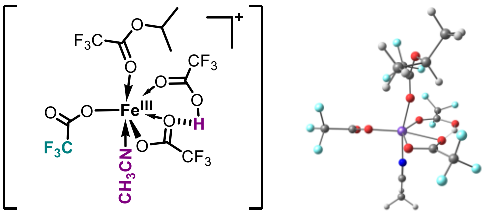

94 (7.6 kcal/mol)

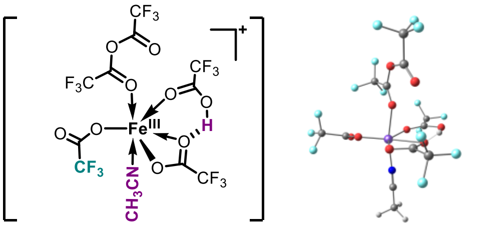

95 (12.5 kcal/mol)

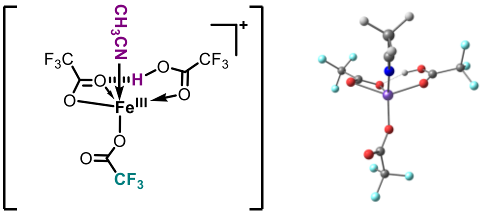

96 (3.3 kcal/mol)

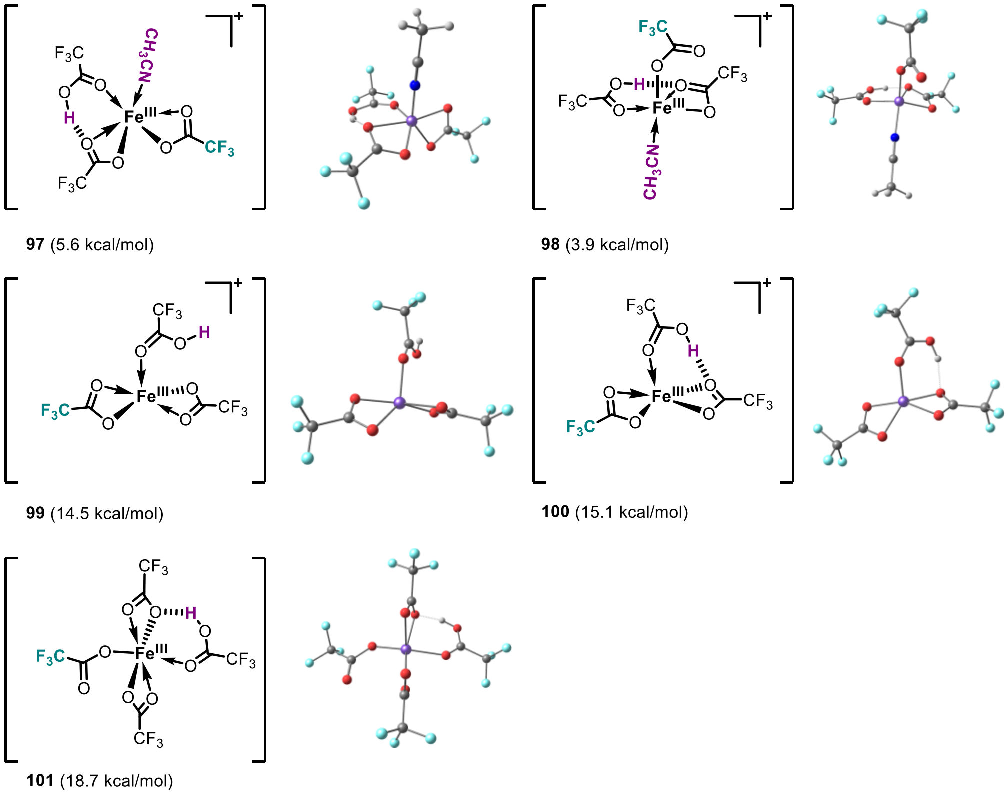

Supplementary Figure 34. Computational investigations of iron and CF_3COO^--based complexes.

Then, the TD-DFT calculations for the **12** were conducted to reveal the light-induced electron transfer process. As shown in the Supplementary Figure 35, 36, and 37, the absorption peak in 450.02 nm can be excited by our blue LEDs (λ: 404 – 499 nm).

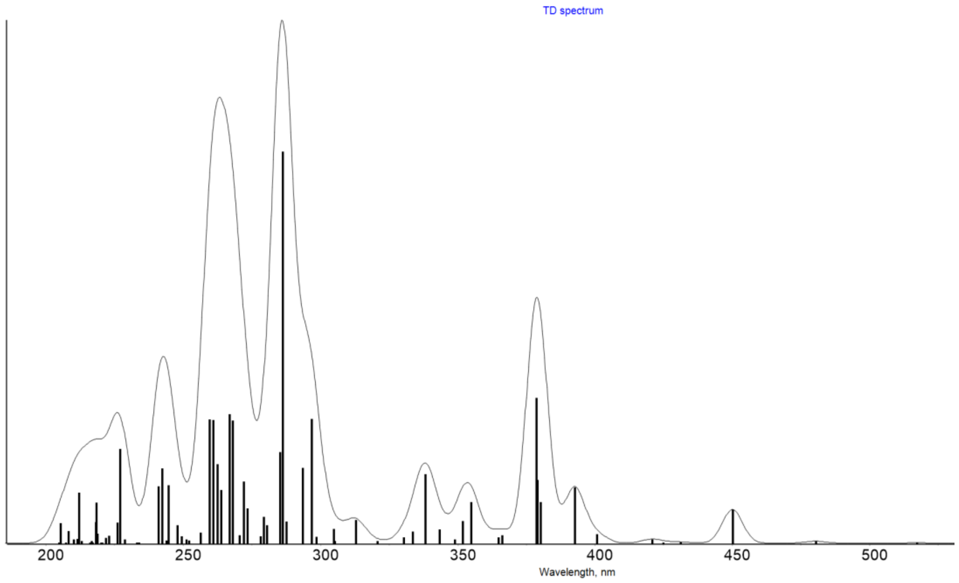

Supplementary Figure 35. UV-vis absorption predicted by the TD-DFT (**12**).

transition	coefficient
112-Beta→117-Beta	0.10650

113-Beta→116-Beta	-0.50854
113-Beta→118-Beta	0.23940
114-Beta→117-Beta	0.75302
115-Beta→116-Beta	-0.21293
115-Beta→118-Beta	0.10251

Supplementary Figure 36. Major transition modes and coefficients (**12**).

The orbital analysis shows that the electron in the trifluoroacetate of **12** could be excited to the iron center to achieve LMCT under our blue LEDs irradiation.

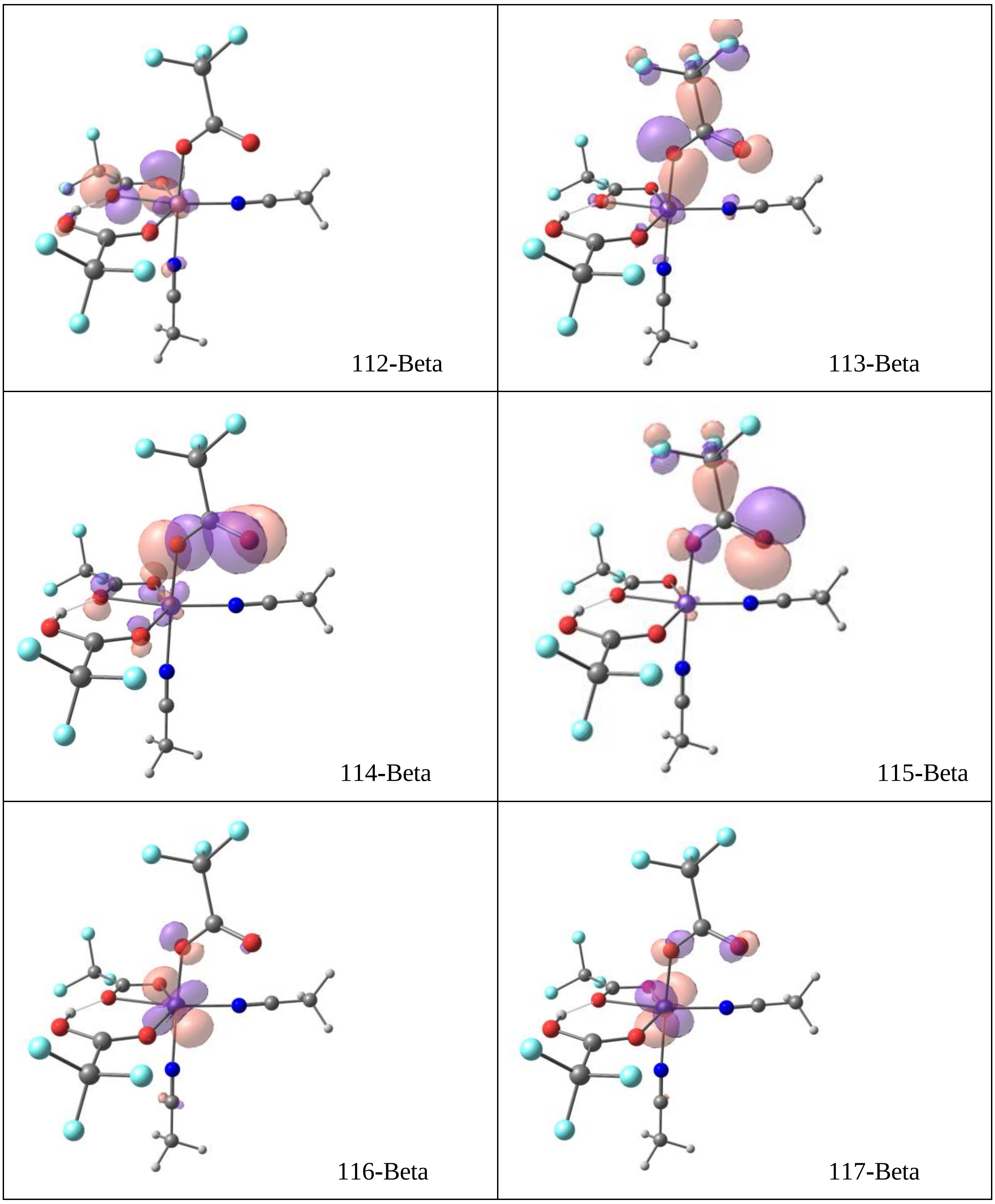

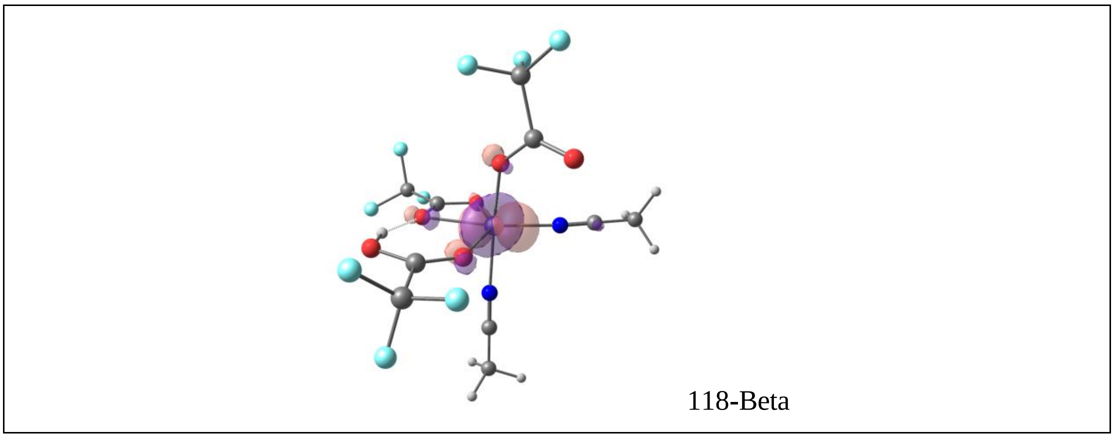

Supplementary Figure 37. Mentioned orbitals for the peak in 450.02 nm.

Light-harvesting species without H+

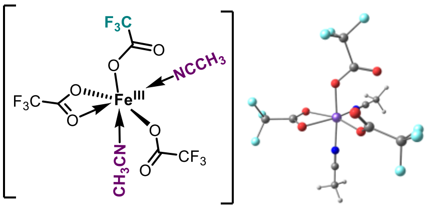

Supplementary Figure 38. 3D structure of **12'**.

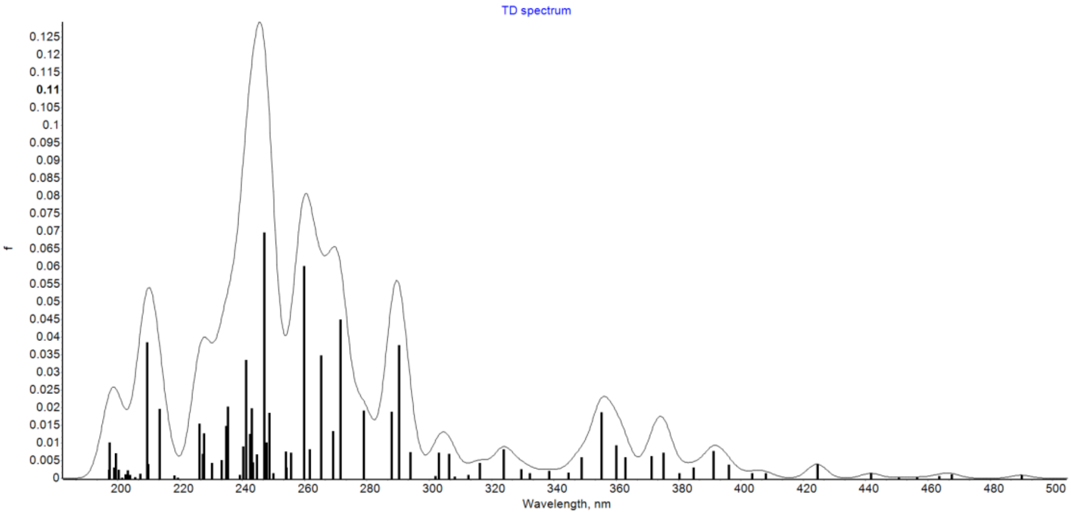

Supplementary Figure 39. UV-vis spectra predicted by the TD-DFT (**12'**).

transition	coefficient
108-Beta→116-Beta	0.10802
112-Beta→116-Beta	-0.12540
113-Beta→116-Beta	0.91231
114-Beta→116-Beta	-0.14856

Supplementary Figure 40. Major transition modes and coefficients (**12'**).

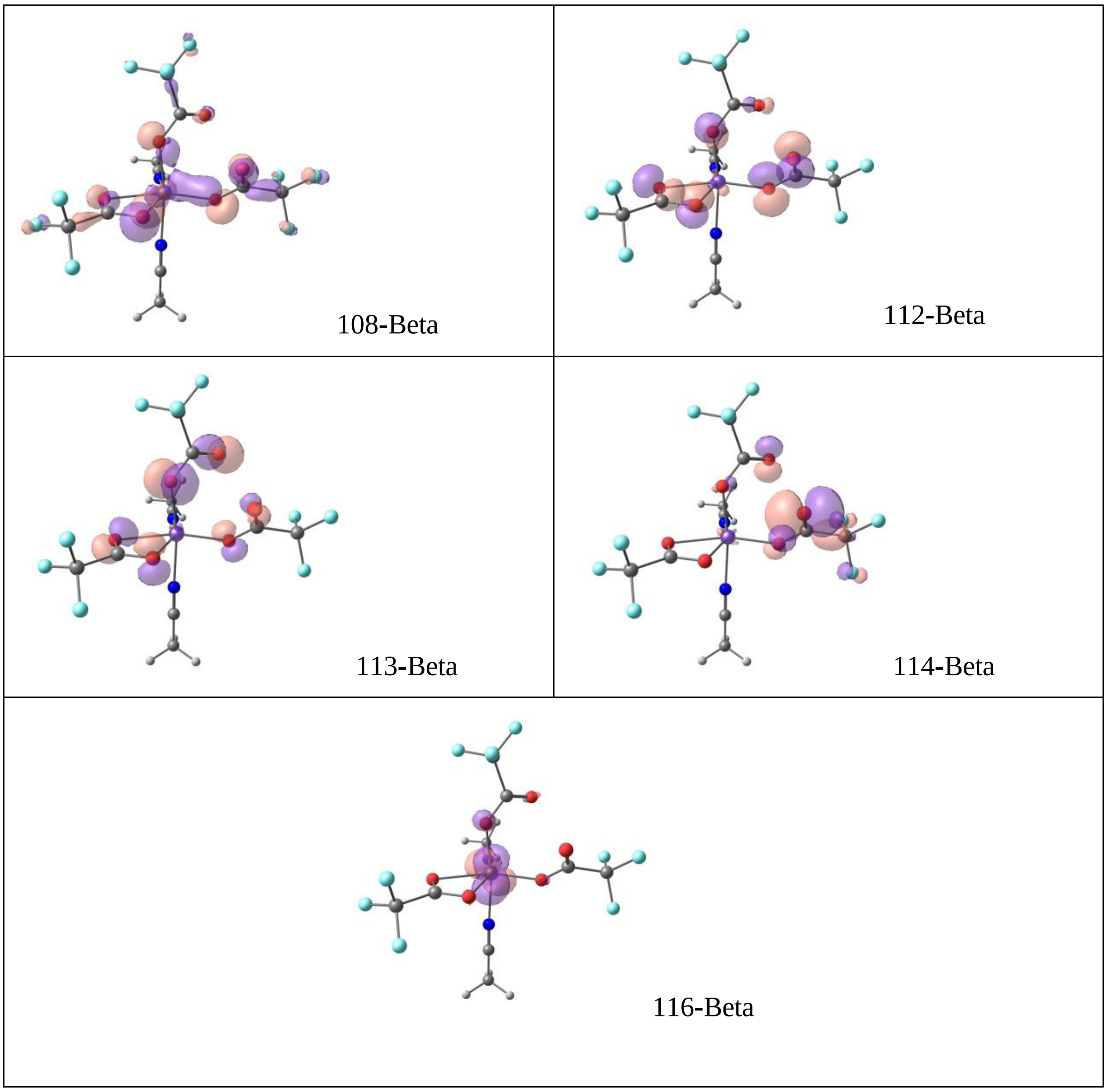

Supplementary Figure 41. Key orbitals of the most possible absorption peak (**12'**).

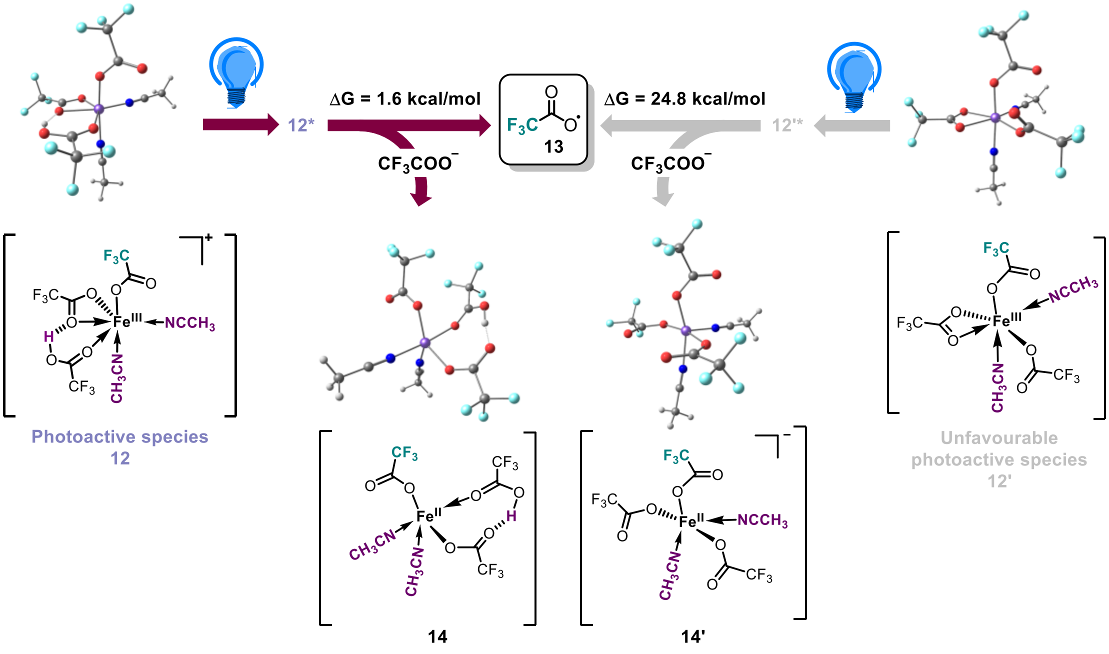

Supplementary Figure 42. Comparison between the **12** and **12'** under blue LEDs irradiation.

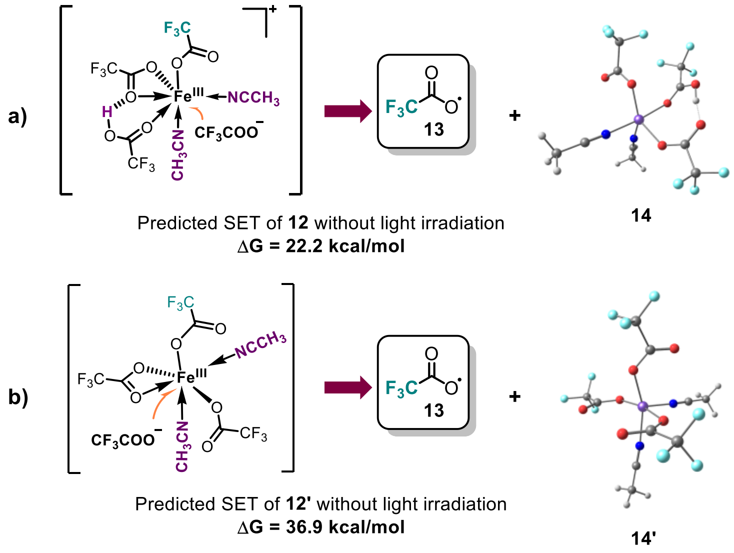

Supplementary Figure 43. SET comparison between **12** and **12'** without light irradiation.

We also compared the orbital energies of **12** and **12'** (Supplementary Figure 44). In the presence of intramolecular hydrogen bonding, the LUMO energy of iron significantly decreased by 0.86 eV, which is beneficial to the LMCT process.

Species	E (arb. units)	Electron volt (eV)
**12** (LUMO)	-0.1892	-5.1462
**12'** (LUMO)	-0.1575	-4.2840

Supplementary Figure 44. LUMO energy comparison between **12** and **12'**.

Species	G (arb. units)
**12** (doublet)	-3108.130262
**12** (quartet)	-3108.137950
**12 (hextet)**	**-3108.161111**
**14** (singlet)	-3108.325434
**14** (triplet)	-3108.350736
**14 (quintet)**	**-3108.376685**

Supplementary Figure 45. Spin states determination of **12** and **14**.

Light-harvesting species involved F^-

The formation of **87** requires the F^- generation from Selectfluor, while Selectfluor plays the role as an electrophilic fluorination reagent to capture the alkyl radical intermediate **15** in this system. Considering the fact that exogenous F^- shows the suppression effect for alkene fluorotrifluoromethylation (Supplementary Figure 51), we proposed that the concentration of F^- under the standard condition should be low. Although **87** was detected by ESI-HRMS spectrometry and DFT calculations also demonstrated its feasibility of generating CF_3 radical under blue light irradiation (Supplementary Figure 46), the low concentration of F^- in the process of reaction may indicate that **87** is not the first choice in comparison to **12**, but alternative. To further understand the role of possible species **87**, this protocol was extended to the trifluoromethylation of arene though directly replacing Selectfluor into sodium persulfate. As shown in Supplementary Figure 50, even

in the absence of fluorine source, this protocol is still feasible to produce CF_3 radical, while the exogenous F^- do not promote the trifluoromethylation of arene. It indicates that **87** is not necessary for this protocol and **12** is the first choice for effective iron-based light-harvesting species under this standard condition.

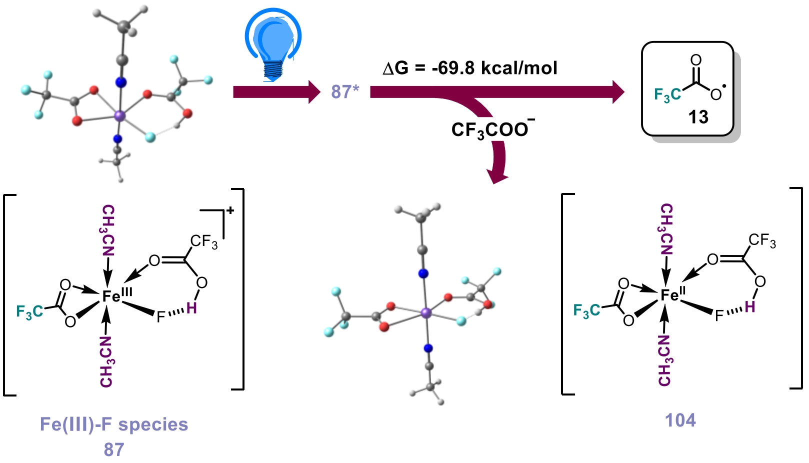

Supplementary Figure 46. DFT calculation of **87**.

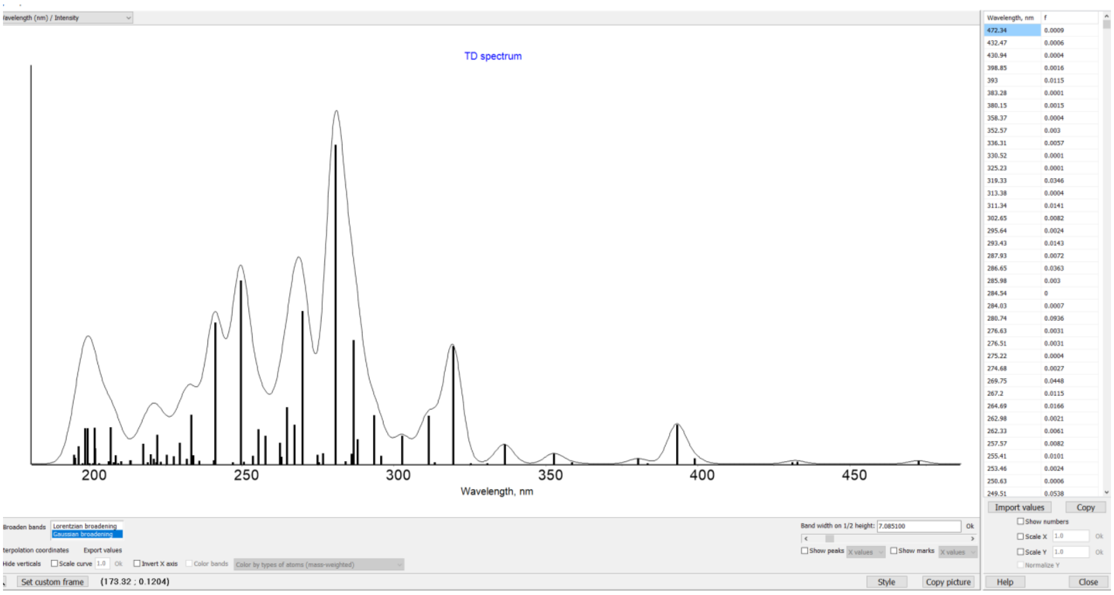

Supplementary Figure 47. UV-vis spectra predicted by the TD-DFT (**87**).

transition	coefficient
89-Beta→93-Beta	-0.18964
90-Beta→93-Beta	-0.15313
91-Beta→93-Beta	-0.26813
92-Beta→93-Beta	0.92180

Supplementary Figure 48. Major transition modes and coefficients (**87**).

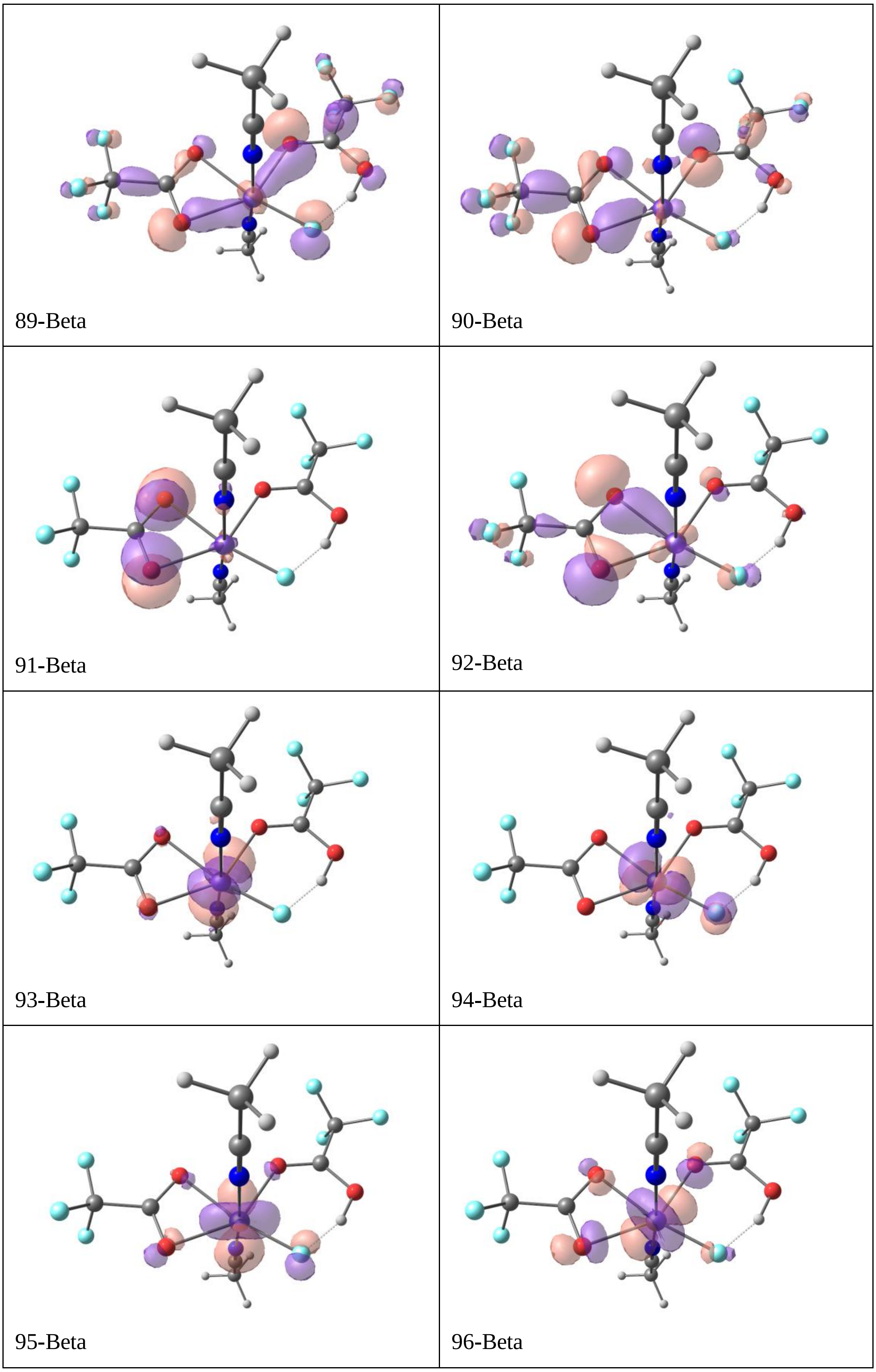

Supplementary Figure 49. Key orbitals of the most possible absorption peak (**87**).

General procedure for trifluoromethylation of arenes: A 25 mL Schlenk flask equipped with a magnetic bar was charged with Fe(acac)$_3$ (0.02 mmol), $Na_2S_2O_8$ (0.4 mmol) and methyl anisate (102, 0.2 mmol). The flask was evacuated and refilled with N_2 for three times. The vessel was then

charged with extra dry CH_3CN (2.0 mL), oxydibenzene (17.0 mg, 0.1 mmol), isopropanol (24.0 mg, 0.4 mmol), TBAF (0 mmol, 0.08 mmol, 0.2 mmol) and TFAA (2.0 mmol). The reaction mixture was stirred under nitrogen atmosphere and irradiated by blue LEDs 12 h. After completion of the reaction. Then the reaction system was quenched by triethylamine, diluted with EtOAc. Yields were determined by GC analysis of the crude reaction mixture, using naphthalene as an internal standard.

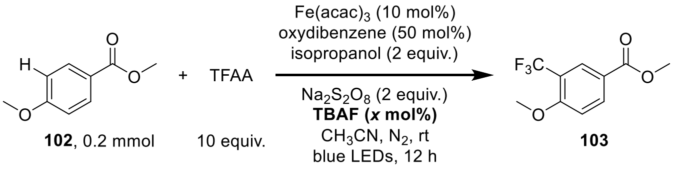

Entry	TBAF (*x* mol%)	**103**, Yield
1	0	22%
2	40	9%
3	100	6%

Supplementary Table 7. Investigation of exogenous F^- effect for trifluoromethylation of arenes.

General procedure for the fluorotrifluoromethylation of alkenes involved fluorion: A 25 mL Schlenk flask equipped with a magnetic bar was charged with $Fe(acac)_3$ (0.02 mmol), Selectfluor (0.3 mmol). The flask was evacuated and refilled with N_2 for three times. The vessel was then charged with extra dry CH_3CN (2.0 mL), oxydibenzene (17.0 mg, 0.1 mmol), isopropanol (24.0 mg, 0.4 mmol), TBAF (0 mmol, 0.1 mmol, 0.2 mmol) and TFAA (1.0 mmol). The reaction mixture was stirred under nitrogen atmosphere and irradiated by blue LEDs 12 h. After completion of the reaction. Then the reaction system was quenched by triethylamine, diluted with EtOAc. Yields were determined by GC analysis of the crude reaction mixture, using biphenyl as an internal standard.

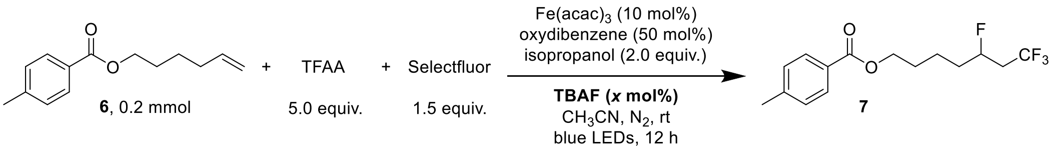

Entry	TBAF (*x* mol%)	**7**, Yield
1	/	70%
2	50	29%
3	100	N.D.

Supplementary Table 8. Investigation of exogenous F^- effect for fluorotrifluoromethylation of alkenes.

Species	E (arb. units)
Selectfluor	-844.45939
[Selectfluor]$^{-}$	-844.70564
Ph_2O	-538.53302
**17**	-538.31759
**12**	-3108.27344
**14**	-3108.48845

**12'**	-3107.85594
**14'**	-3108.02774

Supplementary Figure 50. Calculation for the possible single electron transfer.

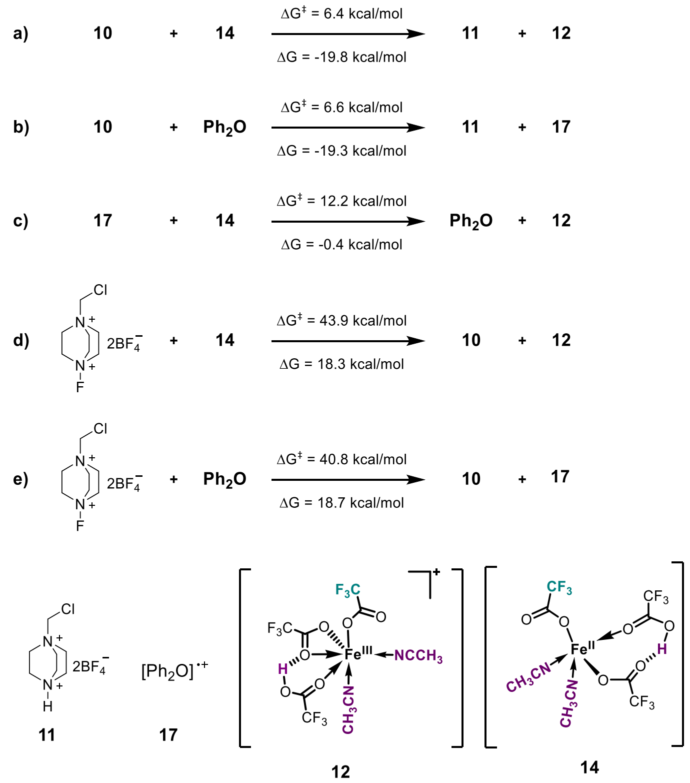

Supplementary Figure 51. DFT calculation for SET process.

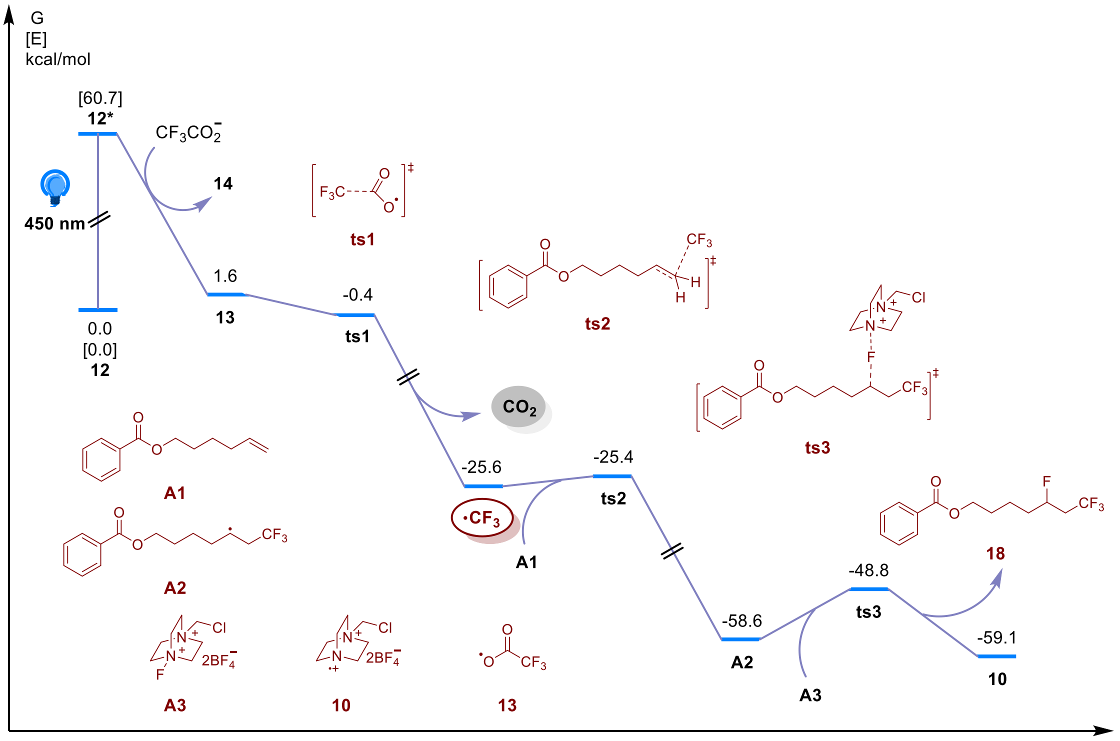

Supplementary Figure 52. Free energy profile for the generation of the product.

Species	Gibbs free energies (arb. units)	Species	Gibbs free energies (arb. units)
**12**	-3108.161111	**14**	-3108.376568
**12'**	-3107.757221	**14'**	-3107.935688
**87**	-2681.839651	**104**	-2682.16889
**88**	-3108.160518	**89**	-3108.159142
**90**	-3513.783164	**91**	-3620.063222
**92**	-3952.512065	**93**	-3513.788757
**94**	-3620.062247	**95**	-3952.509614
**96**	-2975.409546	**97**	-2975.410447
**98**	-2975.406744	**99**	-2842.58805
**100**	-2842.64628	**101**	-3369.04626
**$CF_3CO_2^-$**	-526.333578	**13**	-526.115524
**MeCN**	-132.745395	**$CF_3CO_2{}^iPr$**	-644.658704
**Ph_2O**	-538.385279	**$(CF_3CO)_2O$**	-977.113857
**ts1**	-526.118672		

Supplementary Figure 53. Corresponding free energies of the key species.

3. Supplementary Notes

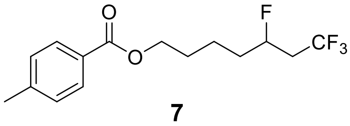

7

5,7,7,7-Tetrafluoroheptyl 4-methylbenzoate (**7**): The compound was purified by columnchromatography (petroleum ether/EtOAc = 60/1), resulting in a colorless liquid (42.7 mg) at 70% yield. **^{1}H NMR** (400 MHz, Chloroform-*d*) δ 7.92 (d, *J* = 8.2 Hz, 2H), 7.23 (d, *J* = 8.0 Hz, 2H), 4.82 (dtt, *J* = 48.7, 8.2, 3.3 Hz, 1H), 4.32 (t, *J* = 6.4 Hz, 2H), 2.62 – 2.19 (m, 2H), 2.40 (s, 3H), 1.88 – 1.48 (m, 6H). **^{13}C NMR** (101 MHz, Chloroform-d) δ 166.75, 143.73, 129.65, 129.19, 127.64, 125.54 (dq, *J* = 3.4, 276.8 Hz), 87.51 (dq, *J* = 172.4, 3.2 Hz), 64.39, 39.46 (qd, *J* = 28.5, 22.9 Hz), 34.71 (d, *J* = 20.8 Hz), 28.45, 21.71, 21.47 (d, *J* = 4.1 Hz). **^{19}F NMR** (376 MHz, Chloroform-*d*) δ -64.12 (d, *J* = 7.6 Hz, 3F), -182.40 (q, *J* = 7.8 Hz, 1F). **ESI-HRMS** exact mass calculated for [$C_{15}H_{18}F_4O_2Na^+$]: 329.1135, found 329.1139.

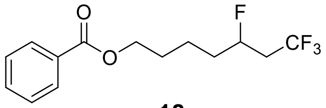

18

5,7,7,7-Tetrafluoroheptyl benzoate (**18**): The compound was purified by columnchromatography (petroleum ether/EtOAc = 60/1), resulting in a colorless liquid (29.2 mg) at 50% yield. **^{1}H NMR** (400 MHz, Chloroform-*d*) δ 8.06 – 8.02 (m, 2H), 7.59 – 7.41 (m, 3H), 4.83 (dtt, *J* = 48.8, 8.1, 3.5 Hz, 1H), 4.34 (t, *J* = 6.4 Hz, 2H), 2.63 – 2.20 (m, 2H), 1.88 – 1.54 (m, 6H). **^{13}C NMR** (101 MHz, Chloroform-d) δ 166.71, 133.07, 130.42, 129.65, 128.50, 125.54(dq, *J* = 3.6, 276.8 Hz), 87.53 (dq, *J* = 172.5, 3.2 Hz), 64.60, 39.54 (qd, *J* = 28.5, 23.0 Hz), 34.75 (d, *J* = 20.9 Hz), 28.47, 21.50 (d, *J* = 4.1 Hz). **^{19}F NMR** (376 MHz, Chloroform-d) δ -64.11 (d, *J* = 7.4 Hz, 3F), -182.39 (q, *J* = 7.4 Hz, 1F). **ESI-HRMS** exact mass calculated for [$C_{14}H_{16}F_4O_2Na^+$]: 315.0979, found 315.0981.

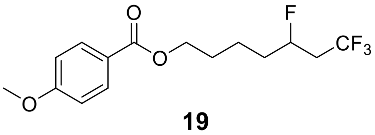

19

5,7,7,7-Tetrafluoroheptyl 4-methoxybenzoate (**19**): The compound was purified by columnchromatography (petroleum ether/EtOAc = 60/1), resulting in a colorless liquid (43.8 mg) at 68% yield. **^{1}H NMR** (400 MHz, Chloroform-*d*) δ 7.99 – 7.95 (m, 2H), 6.93 – 6.80 (m, 2H), 4.82 (dtt, *J* = 49.0, 8.2, 3.5 Hz, 1H), 4.30 (t, *J* = 6.4 Hz, 2H), 3.84 (s, 3H), 2.62 – 2.19 (m, 2H), 1.89 – 1.47 (m, 6H). **^{13}C NMR** (101 MHz, Chloroform-*d*) δ 166.40, 163.47, 131.62, 125.55 (dq, *J* = 3.4, 276.8 Hz), 122.80, 113.71, 87.51 (dq, *J* = 172.4, 3.2 Hz), 64.24, 55.47, 39.45 (qd, *J* = 28.5, 22.9 Hz), 34.70 (d, *J* = 20.9 Hz), 28.48, 21.46 (d, *J* = 4.1 Hz). **^{19}F NMR** (376 MHz, Chloroform-*d*) δ -64.14 (d, *J* = 7.5 Hz, 3F), -182.44 (q, *J* = 7.4 Hz, 1F). **ESI-HRMS** exact mass calculated for [$C_{15}H_{18}F_4O_3Na^+$] :345.1084, found 345.1092.

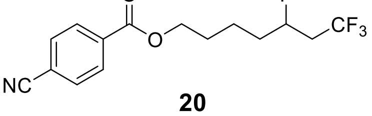

20

5,7,7,7-Tetrafluoroheptyl 4-cyanobenzoate (**20**): The compound was purified by columnchromatography (petroleum ether/EtOAc = 30/1), resulting in a colorless liquid (39.3 mg) at 62% yield. **^{1}H NMR** (400 MHz, DMSO-*d*$_6$) δ 8.11 – 7.95 (m, 4H), 4.95 – 4.73 (m, 1H), 4.30 (t, *J* = 6.4 Hz, 2H), 2.78 – 2.57 (m, 2H), 1.81 – 1.63 (m, 4H), 1.62 – 1.43 (m, 2H). **^{13}C NMR** (101 MHz, DMSO-*d*$_6$) δ 164.51, 133.72, 132.75, 129.76, 126.18 (q, *J* = 277.0 Hz), 118.03, 115.48, 87.90 (dq, *J* = 169.9, 3.2 Hz), 65.15, 37.91 (qd, *J* = 27.3, 21.1 Hz), 33.61 (d, *J* = 20.2 Hz), 27.69, 20.73 (d, *J* = 4.3 Hz). **^{19}F NMR** (376 MHz, DMSO-*d*$_6$) δ -62.62 (d, *J* = 7.6 Hz, 3F), -182.36 (q, *J* = 7.6 Hz, 1F). **ESI-HRMS** exact mass calculated for [$C_{15}H_{15}F_4NO_2Na^+$]: 340.0931, found 340.0943.

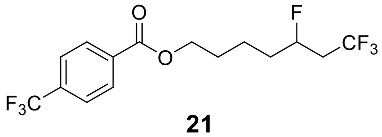

21

5,7,7,7-Tetrafluoroheptyl 4-(trifluoromethyl)benzoate (**21**): The compound was purified by columnchromatography (petroleum ether/EtOAc = 60/1), resulting in a colorless liquid (43.2 mg) at 60% yield. **^{1}H NMR** (400 MHz, DMSO-d_6) δ 8.17 – 7.85 (m, 4H), 4.94 – 4.73 (m, 1H), 4.32 (t, *J* = 6.4 Hz, 2H), 2.78 – 2.58 (m, 2H), 1.82 – 1.63 (m, 4H), 1.62 – 1.43 (m, 2H). **^{13}C NMR** (101 MHz, DMSO-d_6) δ 164.67, 133.55 (d, *J* = 0.9 Hz), 132.86 (q, *J* = 32.0 Hz), 129.96, 126.19 (q, *J* = 277.0 Hz), 125.73 (q, *J* = 3.7 Hz), 123.72 (q, *J* = 272.7 Hz), 87.91 (dq, *J* = 169.8, 3.3 Hz), 65.03, 37.90 (qd, *J* = 27.3, 21.0 Hz), 33.60 (d, *J* = 20.0 Hz), 27.71, 20.73 (d, *J* = 4.2 Hz). **^{19}F NMR** (376 MHz, DMSO-d_6) δ -61.84 (s, 3F), -62.61 (d, *J* = 7.7 Hz, 3F), -182.40 (q, *J* = 7.6 Hz, 1F). **ESI-HRMS** exact mass calculated for [$C_{15}H_{15}F_7O_2Na^+$]: 383.0852, found 383.0859.

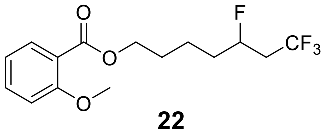

22

5,7,7,7-Tetrafluoroheptyl 2-methoxybenzoate (**22**): The compound was purified by columnchromatography (petroleum ether/EtOAc = 60/1), resulting in a colorless liquid (43.8 mg) at 68% yield. **^{1}H NMR** (400 MHz, Chloroform-*d*) δ 7.79 – 7.75 (m, 1H), 7.5 – 7.43 (m, 1H), 7.01 – 6.94 (m, 2H), 4.92 – 4.72 (m, 1H), 4.31 (t, *J* = 6.3 Hz, 2H), 3.89 (s, 3H), 2.61 – 2.21 (m, 2H), 1.88 – 1.51 (m, 6H). **^{13}C NMR** (101 MHz, Chloroform-*d*) δ 166.44, 159.26, 133.60, 131.63, 125.55 (dq, *J* = 3.5, 276.9 Hz), 120.39, 120.26, 112.16, 87.57 (dq, *J* = 172.4, 3.2 Hz), 64.41, 56.04, 39.54 (qd, *J* = 28.7, 23.3 Hz), 34.73 (d, *J* = 20.8 Hz), 28.44, 21.46 (d, *J* = 4.2 Hz). **^{19}F NMR** (376 MHz, Chloroform-*d*) δ -64.13 (d, *J* = 7.6 Hz, 3F), -182.23 – -182.41 (m, 1F). **ESI-HRMS** exact mass calculated for [$C_{15}H_{18}F_4O_3Na^+$]: 345.1084, found 345.1081.

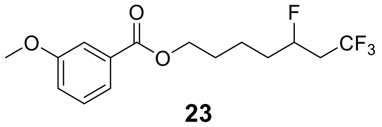

23

5,7,7,7-Tetrafluoroheptyl 3-methoxybenzoate (**23**): The compound was purified by columnchromatography (petroleum ether/EtOAc = 60/1), resulting in a colorless liquid (42.5 mg) at 66% yield. **^{1}H NMR** (400 MHz, Chloroform-*d*) δ 7.64 – 7.53 (m, 2H), 7.36 – 7.30 (m, 1H), 7.11 – 7.06 (m, 1H), 4.91 – 4.71 (m, 1H), 4.32 (t, *J* = 6.5 Hz, 2H), 3.82 (s, 3H), 2.59 – 2.19 (m, 2H), 1.87 – 1.50 (m, 6H). **^{13}C NMR** (101 MHz, Chloroform-*d*) δ 166.45, 159.65, 131.67, 129.45, 125.54 (dq, *J* = 3.3, 276.8 Hz), 121.91, 119.27, 114.23, 87.45 (dq, *J* = 172.5, 3.2 Hz), 64.60, 55.38, 39.36 (qd, *J* = 28.5, 22.8 Hz), 34.62 (d, *J* = 20.9 Hz), 28.35, 21.38 (d, *J* = 4.1 Hz). **^{19}F NMR** (376 MHz, Chloroform-*d*) δ -64.15 (d, *J* = 6.9 Hz, 3F), -182.50 (q, *J* = 7.5 Hz, 1F). **ESI-HRMS** exact mass calculated for [$C_{15}H_{18}F_4O_3Na^+$]: 345.1084, found 383.1089.

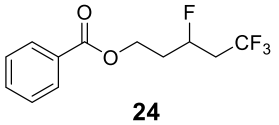

24

3,5,5,5-Tetrafluoropentyl benzoate (**24**): The compound was purified by columnchromatography (petroleum ether/EtOAc = 60/1), resulting in a colorless liquid (27.5 mg) at 52% yield. **^{1}H NMR** (400 MHz, DMSO-d_6) δ 7.98 (d, *J* = 7.4 Hz, 2H), 7.69 – 7.62 (m, 1H), 7.56 – 7.50 (m, 2H), 5.16 – 4.94 (m, 1H), 4.48 – 4.34 (m, 2H), 2.92 – 2.69 (m, 2H), 2.22 – 2.07 (m, 2H). **^{13}C NMR** (101 MHz, DMSO-d_6) δ 165.65, 133.41, 129.66, 129.19, 128.78, 126.15 (q, *J* = 277.0 Hz), 85.76 (dq, *J* = 170.4, 3.4 Hz), 60.49 (d, *J* = 4.7 Hz), 37.85 (qd, *J* = 27.5, 20.6 Hz), 33.25 (d, *J* = 20.2 Hz). **^{19}F NMR** (376 MHz, DMSO-d_6) δ -62.54 (d, *J* = 7.8 Hz, 3F), -184.51 (q, *J* = 8.2 Hz, 1F). **ESI-HRMS** exact mass calculated for [$C_{12}H_{12}F_4O_2Na^+$]: 287.0666, found 287.0661.

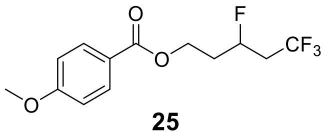

25

3,5,5,5-Tetrafluoropentyl 4-methoxybenzoate (**25**): The compound was purified by columnchromatography (petroleum ether/EtOAc = 60/1), resulting in a colorless liquid (41.2 mg) at 70% yield. **^{1}H NMR** (400 MHz, Chloroform-*d*) δ 8.01 – 7.95 (m, 2H), 6.95 – 6.90 (m, 2H), 5.15 – 4.94 (m, 1H), 4.54 – 4.37 (m, 2H), 3.85 (s, 3H), 2.72 – 2.29 (m, 2H), 2.21 – 2.01 (m, 2H). **^{13}C NMR** (101 MHz, Chloroform-*d*) δ 166.17, 163.69, 131.73, 125.37 (dq, *J* = 3.6, 276.8 Hz), 122.36, 113.85, 85.02 (dq, *J* = 173.3, 3.3 Hz), 59.98 (d, *J* = 4.8 Hz), 55.56, 39.57 (qd, *J* = 28.8, 22.7 Hz), 34.44 (d, *J* = 20.9 Hz). **^{19}F NMR** (376 MHz, Chloroform-*d*) δ -64.02 (d, *J* = 7.4 Hz, 3F), -184.46 (q, *J* = 7.4 Hz, 1F). **ESI-HRMS** exact mass calculated for [$C_{13}H_{14}F_4O_3Na^+$]: 317.0771, found 317.0775.

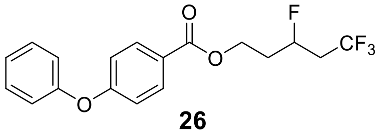

26

3,5,5,5-Tetrafluoropentyl 4-phenoxybenzoate (**26**): The compound was purified by columnchromatography (petroleum ether/EtOAc = 60/1), resulting in a colorless liquid (58.4 mg) at 82% yield. **^{1}H NMR** (400 MHz, Chloroform-*d*) δ 8.00 (d, *J* = 8.8 Hz, 2H), 7.42 – 7.36 (m, 2H), 7.23 – 6.97 (m, 5H), 5.04 (dtt, *J* = 48.8, 8.2, 3.6 Hz, 1H), 4.54 – 4.41 (m, 2H), 2.71 – 2.31 (m, 2H), 2.20 – 2.03 (m, 2H). **^{13}C NMR** (101 MHz, Chloroform-*d*) δ 165.91, 162.21, 155.64, 131.81, 130.17, 125.35 (dq, *J* = 3.6, 276.8 Hz), 124.72, 124.19, 120.27, 117.45, 84.98 (dq, *J* = 173.5, 3.3 Hz), 60.16 (d, *J* = 4.7 Hz), 39.56 (qd, *J* = 28.8, 22.7 Hz), 34.40 (d, *J* = 20.9 Hz). **^{19}F NMR** (376 MHz, Chloroform-*d*) δ -63.98 (d, *J* = 7.5 Hz, 3F), -184.48 (q, *J* = 7.7 Hz, 1F). **ESI-HRMS** exact mass calculated for [$C_{18}H_{16}F_4O_3H^+$]: 357.1108, found 357.1111.

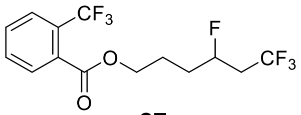

27

4,6,6,6-Tetrafluorohexyl 2-(trifluoromethyl)benzoate (**27**): The compound was purified by columnchromatography (petroleum ether/EtOAc = 60/1), resulting in a colorless liquid (44.5 mg) at 65% yield. **^{1}H NMR** (400 MHz, DMSO-*d*$_6$) δ 7.89 – 7.74 (m, 4H), 5.00 – 4.78 (m, 1H), 4.32 (t, *J* = 5.5 Hz, 2H), 2.81 – 2.59 (m, 2H), 1.92 – 1.68 (m, 4H). **^{13}C NMR** (101 MHz, DMSO-*d*$_6$) δ 166.27, 132.86, 131.86, 130.86 (q, *J* = 2.0 Hz), 129.99, 126.74 (q, *J* = 31.8 Hz), 126.71 (q, *J* = 5.2 Hz), 126.20 (q, *J* = 277.8 Hz), 124.85 (q, *J* = 273.7 Hz), 87.79 (dq, *J* = 169.9, 3.3 Hz), 65.34, 37.87 (qd, *J* = 27.4, 21.0 Hz), 30.63 (d, *J* = 20.4 Hz), 23.45 (d, *J* = 4.1 Hz). **^{19}F NMR** (376 MHz, DMSO-*d*$_6$) δ -58.28 (s, 3F), -62.60 (d, *J* = 7.7 Hz, 3F), -182.74 (q, *J* = 7.6 Hz, 1F). **ESI-HRMS** exact mass calculated for [$C_{14}H_{13}F_7O_2H^+$]: 369.0696, found 369.0706.

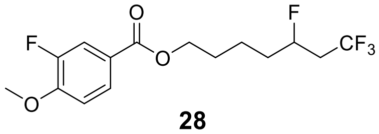

28

5,7,7,7-Tetrafluoroheptyl 3-fluoro-4-methoxybenzoate (**28**): The compound was purified by columnchromatography (petroleum ether/EtOAc = 60/1), resulting in a colorless liquid (41.5 mg) at 61% yield. **^{1}H NMR** (400 MHz, Chloroform-*d*) δ 7.78 – 7.75 (m, 1H), 7.70 – 7.65 (m, 1H), 6.97 – 6.91 (m, 1H), 4.89 – 4.69 (m, 1H), 4.27 (t, *J* = 6.4 Hz, 2H), 3.90 – 3.87 (m, 3H), 2.59 – 2.17 (m, 2H), 1.84 – 1.47 (m, 6H). **^{13}C NMR** (101 MHz, Chloroform-*d*) δ 165.57 (d, *J* = 2.8 Hz), 151.83 (d, *J* = 10.7 Hz), 151.80 (d, *J* = 246.8 Hz), 126.75 (d, *J* = 3.4 Hz), 125.52 (dq, *J* = 3.7, 276.8 Hz), 123.13 (d, *J* = 6.3 Hz), 117.27 (d, *J* = 19.6 Hz), 112.45 (d, *J* = 2.0 Hz), 87.52 (dq, *J* = 172.5, 3.1 Hz), 64.68, 56.37, 39.53 (qd, *J* = 28.6, 23.1 Hz), 34.74 (d, *J* = 20.9 Hz), 28.46, 21.48 (d, *J* = 4.1 Hz). **^{19}F NMR**

(376 MHz, Chloroform-*d*) δ -64.10 (d, *J* = 7.5 Hz, 3F), -134.65 (s, 1F), -182.40 (q, *J* = 7.9 Hz, 1F). **ESI-HRMS** exact mass calculated for [$C_{15}H_{17}F_5O_3Na^+$]: 363.0990, found 363.0997.

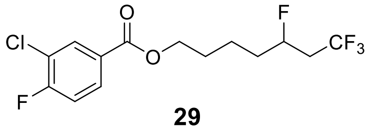

29

5,7,7,7-Tetrafluoroheptyl 3-chloro-4-fluorobenzoate (**29**): The compound was purified by columnchromatography (petroleum ether/EtOAc = 60/1), resulting in a colorless liquid (39.9 mg) at 58% yield. **^{1}H NMR** (400 MHz, Chloroform-*d*) δ 8.10 – 8.05 (m, 1H), 7.95 – 7.89 (m, 1H), 7.22 – 7.15 (m, 1H), 4.83 (dtt, *J* = 48.7, 8.3, 3.3 Hz, 1H), 4.33 (t, *J* = 6.5 Hz, 2H), 2.62 – 2.20 (m, 2H), 1.89 – 1.50 (m, 6H). **^{13}C NMR** (101 MHz, Chloroform-*d*) δ 164.67 (d, *J* = 0.5 Hz), 161.16 (d, *J* = 256.1 Hz), 132.42 (d, *J* = 1.1 Hz), 130.05 (d, *J* = 8.6 Hz), 127.61 (d, *J* = 3.9 Hz), 125.52 (dq, *J* = 3.7, 276.8 Hz), 121.55 (d, *J* = 18.3 Hz), 116.75 (d, *J* = 21.8 Hz), 87.48 (dq, *J* = 172.6, 3.5 Hz), 65.15, 39.49 (qd, *J* = 28.8, 22.8 Hz), 34.69 (d, *J* = 21.1 Hz), 28.37, 21.43 (d, *J* = 4.4 Hz). **^{19}F NMR** (376 MHz, Chloroform-*d*) δ -64.14 (d, *J* = 7.5 Hz, 3F), -108.13 (s, 1F), -182.54 (q, *J* = 7.8 Hz, 1F). **ESI-HRMS** exact mass calculated for [$C_{14}H_{14}ClF_5O_2Na^+$]: 367.0495, found 367.0502.

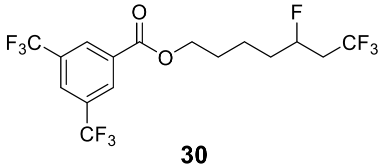

30

5,7,7,7-Tetrafluoroheptyl 3,4-bis(trifluoromethyl)benzoate (**30**): The compound was purified by columnchromatography (petroleum ether/EtOAc = 50/1), resulting in a colorless liquid (52.2 mg) at 61% yield. **^{1}H NMR** (400 MHz, Chloroform-*d*) δ 8.47 (s, 2H), 8.06 (s, 1H), 4.83 (dtt, *J* = 48.9, 8.0, 3.6 Hz, 1H), 4.42 (t, *J* = 6.6 Hz, 2H), 2.63 – 2.22 (m, 2H), 1.94 – 1.54 (m, 6H). **^{13}C NMR** (101 MHz, Chloroform-*d*) δ 164.06, 132.57, 132.38 (q, *J* = 34.0 Hz), 129.94 – 129.75 (m), 126.61 – 126.31 (m), 125.49 (dq, *J* = 4.0, 276.7 Hz), 123.02 (q, *J* = 272.9 Hz), 87.46 (dq, *J* = 172.6, 3.1 Hz), 65.90, 39.59 (qd, *J* = 28.6, 23.2 Hz), 34.72 (d, *J* = 20.9 Hz), 28.37, 21.45 (d, *J* = 4.0 Hz). **^{19}F NMR** (376 MHz, Chloroform-*d*) δ -63.03 (s, 3F), -63.04 (s, 3F), -64.16 (t, *J* = 7.1 Hz, 3F), -182.59 (q, *J* = 8.0 Hz, 1F). **ESI-HRMS** exact mass calculated for [$C_{16}H_{14}F_{10}O_2Na^+$]: 451.0726, found 451.0739.

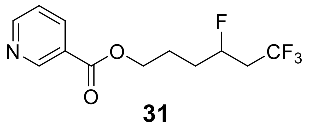

31

5,7,7,7-Tetrafluoroheptyl nicotinate (**31**): The compound was purified by columnchromatography (petroleum ether/EtOAc = 30/1), resulting in a colorless liquid (41.4 mg) at 74% yield. **^{1}H NMR** (400 MHz, Chloroform-*d*) δ 9.24 – 9.16 (m, 1H), 8.81 – 8.73 (m, 1H), 8.29 – 8.24 (m, 1H), 7.38 (dd, *J* = 8.0, 4.9 Hz, 1H), 4.86 (dtt, *J* = 49.1, 7.9, 3.7, Hz, 1H), 4.48 – 4.33 (m, 2H), 2.64 – 2.22 (m, 2H), 2.07 – 1.70 (m, 4H). **^{13}C NMR** (101 MHz, Chloroform-*d*) δ 165.25, 153.62, 150.93, 137.08, 126.07, 125.38 (dq, *J* = 3.9, 276.8 Hz), 123.42, 87.23 (dq, *J* = 173.0, 3.2 Hz), 64.64, 39.49 (qd, *J* = 28.6, 23.0 Hz), 31.74 (d, *J* = 21.2 Hz), 24.24 (d, *J* = 3.9 Hz). **^{19}F NMR** (376 MHz, Chloroform-*d*) δ -64.12 (d, *J* = 7.3 Hz, 3F), -182.90 (q, *J* = 7.5 Hz, 1F). **ESI-HRMS** exact mass calculated for [$C_{12}H_{13}F_4NO_2H^+$]: 280.0955, found 280.0956.

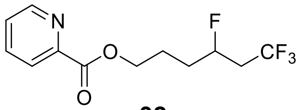

32

4,6,6,6-Tetrafluorohexyl picolinate (**32**): The compound was purified by columnchromatography (petroleum ether/EtOAc = 30/1), resulting in a colorless liquid (40.2 mg) at 72% yield. **^{1}H NMR** (400 MHz, Chloroform-*d*) δ 8.74 (d, *J* = 4.7 Hz, 1H), 8.10 (d, *J* = 7.8 Hz, 1H), 7.86 – 7.79 (m, 1H), 7.49 – 7.44 (m, 1H), 4.86 (dtt, *J* = 49.2, 8.3, 3.6 Hz, 1H), 4.51 – 4.39 (m, 2H), 2.62 – 2.21 (m, 2H), 2.09 – 1.70 (m, 4H). **^{13}C NMR** (101 MHz, Chloroform-*d*) δ 165.32, 150.07, 148.07, 137.18, 127.13,

125.44 (dq, *J* =3.4, 279.8 Hz), 125.31, 87.31 (dq, *J* = 173.0, 3.1 Hz), 65.18, 39.55 (qd, *J* = 28.6, 22.8 Hz), 31.80 (d, *J* = 21.2 Hz), 24.32 (d, *J* = 4.0 Hz). **^{19}F NMR** (376 MHz, Chloroform-*d*) δ -64.12 (d, *J* = 7.5 Hz, 3F), -182.81 (q, *J* = 7.7 Hz, 1F). **ESI-HRMS** exact mass calculated for [$C_{12}H_{13}F_4NO_2Na^+$]: 302.0775, found 302.0785.

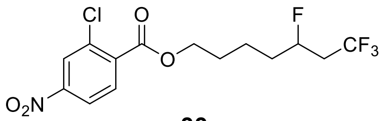

33

5,7,7,7-Tetrafluoroheptyl-2-chloro-4-nitrobenzoate (**33**): The compound was purified by columnchromatography (petroleum ether/EtOAc = 30/1), resulting in a colorless liquid (23.8 mg) at 32% yield. **^{1}H NMR** (400 MHz, Chloroform-*d*) δ 8.30 – 8.27 (m, 1H), 8.16 – 8.12 (m, 1H), 7.95 – 7.91 (m, 1H), 4.91 – 4.72 (m, 1H), 4.39 (t, *J* = 6.5 Hz, 2H), 2.63 – 2.21 (m, 2H), 1.90 – 1.50 (m, 6H). **^{13}C NMR** (101 MHz, Chloroform-*d*) δ 164.38, 149.50, 136.12, 134.70, 132.06, 126.06, 125.48 (dq, *J* = 3.6, 276.8 Hz), 121.58, 87.44 (dq, *J* = 172.5, 3.1 Hz), 66.06, 39.43 (qd, *J* = 28.6, 23.0 Hz), 34.56 (d, *J* = 20.9 Hz), 28.17, 21.38 (d, *J* = 4.0 Hz). **^{19}F NMR** (376 MHz, Chloroform-*d*) δ -64.12 (d, *J* = 7.1 Hz, 3F), -182.59 (q, *J* = 7.4 Hz, 1F). **ESI-HRMS** exact mass calculated for [$C_{14}H_{14}ClF_4O_4Na^+$]: 394.0440, found 394.0446.

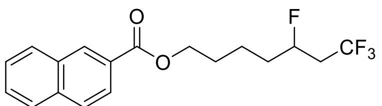

34

5,7,7,7-Tetrafluoroheptyl-2-naphthoate (**34**): The compound was purified by columnchromatography (petroleum ether/EtOAc = 60/1), resulting in a colorless liquid (32.8 mg) at 48% yield. **^{1}H NMR** (400 MHz, Chloroform-*d*) δ 8.62 (s, 1H), 8.10 – 8.04 (m, 1H), 7.96 (d, *J* = 8.0 Hz, 1H), 7.92 – 7.84 (m, 2H), 7.42 – 7.36 (m, 2H), 4.93 – 4.73 (m, 1H), 4.41 (t, *J* = 6.5 Hz, 2H), 2.64 – 2.22 (m, 2H), 1.92 – 1.55 (m, 6H). **^{13}C NMR** (101 MHz, Chloroform-*d*) δ 166.81, 135.62, 132.59, 131.07, 129.42, 128.35, 128.26, 127.85, 127.63, 126.76, 125.55 (dq, *J* = 3.4, 276.8 Hz), 125.27, 87.50 (dq, *J* = 172.5, 3.2 Hz), 64.72, 39.47 (qd, *J* = 28.5, 23.0 Hz), 34.71 (d, *J* = 20.8 Hz), 28.47, 21.48 (d, *J* = 4.2 Hz). **^{19}F NMR** (376 MHz, Chloroform-*d*) δ -64.04 (d, *J* = 7.6 Hz, 3F), -182.36 (q, *J* = 8.0 Hz, 1F). **ESI-HRMS** exact mass calculated for [$C_{18}H_{18}F_4O_2Na^+$]: 365.1135, found 365.1142.

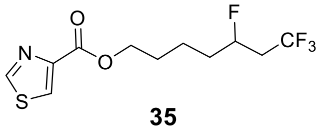

35

5,7,7,7-Tetrafluoroheptyl thiazole-4-carboxylate (**35**): The compound was purified by columnchromatography (petroleum ether/EtOAc = 30/1), resulting in a white solid (42.5 mg) at 71% yield. **^{1}H NMR** (400 MHz, Chloroform-*d*) δ 8.82 – 8.79 (m, 1H), 8.20 – 8.18 (m, 1H), 4.86 – 4.65 (m, 1H), 4.33 (t, *J* = 6.6 Hz, 2H), 2.56 – 2.14 (m, 2H), 1.85 – 1.44 (m, 6H). **^{13}C NMR** (101 MHz, Chloroform-*d*) δ 161.26, 153.53, 147.94, 127.35, 125.42 (dq, *J* = 3.2, 276.8 Hz), 87.36 (dq, *J* = 172.4, 3.2 Hz), 64.98, 39.30 (qd, *J* = 28.5, 22.8 Hz), 34.51 (d, *J* = 20.9 Hz), 28.22, 21.24 (d, *J* = 4.2 Hz). **^{19}F NMR** (376 MHz, DMSO-d_6) δ -62.42 (d, *J* = 8.0 Hz, 3F), -182.31 (q, *J* = 8.1 Hz, 1F). **ESI-HRMS** exact mass calculated for [$C_{11}H_{13}F_4NO_2SNa^+$]: 322.0495, found 322.0498.

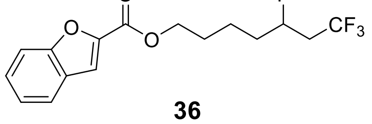

36

5,7,7,7-Tetrafluoroheptyl benzofuran-2-carboxylate (**36**): The compound was purified by columnchromatography (petroleum ether/EtOAc = 50/1), resulting in a colorless liquid (31.2 mg) at 47% yield. **^{1}H NMR** (400 MHz, Chloroform-*d*) δ 7.69 – 7.65 (m, 1H), 7.60 – 7.56 (m, 1H), 7.53 – 7.50 (m, 1H), 7.46 – 7.41 (m, 1H), 7.32 – 7.26 (m, 1H), 4.92 – 4.72 (m, 1H), 4.38 (t, *J* = 6.5 Hz, 2H), 2.62 – 2.20 (m, 2H), 1.88 – 1.50 (m, 6H). **^{13}C NMR** (101 MHz, Chloroform-*d*) δ 159.67,

155.85, 145.59, 127.77, 127.03, 125.52 (dq, *J* = 3.3, 276.6 Hz), 123.92, 122.93, 114.03, 112.45, 87.48 (dq, *J* = 172.5, 3.2 Hz), 65.05, 39.49 (qd, *J* = 28.6, 23.0 Hz), 34.66 (d, *J* = 20.9 Hz), 28.38, 21.37 (d, *J* = 4.1 Hz). ^{19}**F NMR** (376 MHz, Chloroform-*d*) δ -64.08 (d, *J* = 7.5 Hz, 3F), -182.44 (q, *J* = 7.9 Hz, 1F). **ESI-HRMS** exact mass calculated for [$C_{16}H_{16}F_4O_3Na^+$]: 355.0928, found 355.0930.

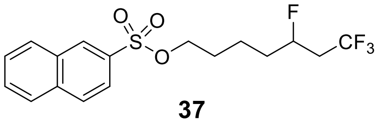

37

5,7,7,7-Tetrafluoroheptyl naphthalene-2-sulfonate (**37**): The compound was purified by columnchromatography (petroleum ether/EtOAc = 10/1), resulting in a colorless liquid (34.0 mg) at 45% yield. ^{1}**H NMR** (400 MHz, DMSO-*d*$_6$) δ 8.68 – 8.61 (m, 1H), 8.27 – 8.18 (m, 2H), 8.10 (d, *J* = 8.1 Hz, 1H), 7.89 – 7.85 (m, 1H), 7.83 – 7.67 (m, 2H), 4.72 (dtt, *J* = 50.1, 7.9, 4.2 Hz, 1H), 4.09 (t, *J* = 6.3 Hz, 2H), 2.66 – 2.51 (m, 2H), 1.65 – 1.28 (m, 4H), 1.43 – 1.26 (m, 2H). ^{13}**C NMR** (101 MHz, Chloroform-*d*) δ 135.42, 132.91, 132.08, 129.86, 129.85, 129.57, 129.46, 128.15, 128.03, 125.43 (dq, *J* = 3.7, 276.9 Hz), 122.62, 87.35 (dq, *J* = 172.8, 3.2 Hz), 70.38, 39.42 (qd, *J* = 28.6, 22.9 Hz), 34.34 (d, *J* = 20.9 Hz), 28.54, 20.91 (d, *J* = 4.1 Hz). ^{19}**F NMR** (376 MHz, Chloroform-*d*) δ -57.76 (d, *J* = 7.5 Hz, 3F), -177.66 (q, *J* = 7.6 Hz, 1F). **ESI-HRMS** exact mass calculated for [$C_{17}H_{18}F_4O_3SNa^+$]: 401.0805, found 401.0803.

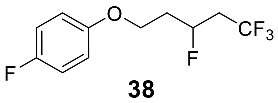

38

1-Fluoro-4-((3,5,5,5-tetrafluoropentyl)oxy)benzene (**38**): The compound was purified by columnchromatography (petroleum ether/EtOAc = 80/1), resulting in a colorless liquid (37.1 mg) at 73% yield. ^{1}**H NMR** (400 MHz, Chloroform-*d*) δ 7.02 – 6.96 (m, 2H), 6.88 – 6.83 (m, 2H), 5.22 – 5.01 (m, 1H), 4.13 – 4.03 (m, 2H), 2.71 – 2.34 (m, 2H), 2.20 – 2.08 (m, 2H). ^{13}**C NMR** (101 MHz, Chloroform-*d*) δ 157.59 (d, *J* = 238.5 Hz), 154.77 (d, *J* = 2.1 Hz), 125.53 (dq, *J* = 2.9, 276.8 Hz), 116.01 (d, *J* = 23.2 Hz), 115.63 (d, *J* = 8.0 Hz), 84.98 (dq, *J* = 172.5, 3.3 Hz), 63.62 (d, *J* = 4.9 Hz), 39.60 (qd, *J* = 28.7, 22.4 Hz), 34.88 (d, *J* = 20.7 Hz). ^{19}**F NMR** (376 MHz, Chloroform-*d*) δ -64.08 (d, *J* = 7.6 Hz, 3F), -123.62 (s, 1F), -184.82 (q, *J* = 8.3, 7.5 Hz, 1F). **ESI-HRMS** exact mass calculated for [$C_{11}H_{11}F_5ONa^+$]: 277.0622, found 277.0628.

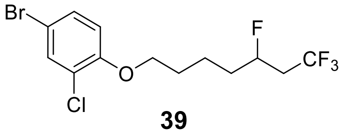

39

4-Bromo-2-chloro-1-((5,7,7,7-tetrafluoroheptyl)oxy)benzene (**39**): The compound was purified by columnchromatography (petroleum ether/EtOAc = 60/1), resulting in a colorless liquid (45.8 mg) at 61% yield. ^{1}**H NMR** (400 MHz, Chloroform-*d*) δ 7.51 – 7.48 (m, 1H), 7.33 – 7.28 (m, 1H), 6.77 (d, *J* = 8.8 Hz, 1H), 4.84 (dtt, *J* = 48.3, 7.9, 3.2 Hz, 1H), 4.01 (t, *J* = 6.1 Hz, 2H), 2.56 – 2.13 (m, 2H), 1.85 – 1.50 (m, 6H). ^{13}**C NMR** (101 MHz, Chloroform-*d*) δ 153.90, 132.82, 130.63, 125.57 (dq, *J* = 3.4, 276.8 Hz), 124.21, 114.62, 112.67, 87.59 (dq, *J* = 172.4, 3.2 Hz), 69.08, 39.52 (qd, *J* = 28.5, 22.9 Hz), 34.76 (d, *J* = 20.8 Hz), 28.67, 21.55 (d, *J* = 4.3 Hz). ^{19}**F NMR** (376 MHz, Chloroform-*d*) δ -64.09 (d, *J* = 7.5 Hz, 3F), -182.33 (q, *J* = 7.4 Hz, 1F). **ESI-HRMS** exact mass calculated for [$C_{13}H_{14}BrClF_4ONa^+$]: 398.9745, found 3989.9756.

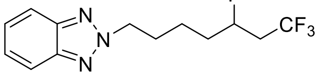

40

2-(5,7,7,7-Tetrafluoroheptyl)-2H-benzo[d][1,2,3]triazole (**40**): The compound was purified by columnchromatography (petroleum ether/EtOAc = 20/1), resulting in a colorless liquid (28.3 mg) at 49% yield. ^{1}**H NMR** (400 MHz, Chloroform-*d*) δ 7.88 – 7.32 (m, 4H), 4.77 (dtt, *J* = 12.1, 8.0, 3.5 Hz, 1H), 4.75 (t, *J* = 6.9 Hz, 2H), 2.65 – 2.08 (m, 2H), 2.22 – 2.12 (m, 2H), 1.87 – 1.38 (m, 4H). ^{13}**C NMR** (101 MHz, Chloroform-*d*) δ 144.43, 126.43, 125.45 (dq, *J* = 3.6, 276.9 Hz), 118.06, 87.34

(dq, J = 172.7, 3.2 Hz), 56.21, 39.43 (qd, J = 28.6, 22.9 Hz), 34.41 (d, J = 20.9 Hz), 29.58, 21.90 (d, J = 4.1 Hz). **^{19}F NMR** (376 MHz, Chloroform-*d*) δ -64.12 (d, J = 7.4 Hz, 3F), -182.43 (q, J = 7.4 Hz, 1F). **ESI-HRMS** exact mass calculated for [$C_{13}H_{15}F_4N_3Na^+$]: 312.1094, found 312.1100.

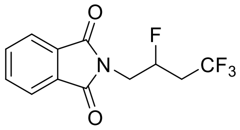

41

2-(1,3,3,3-Tetrafluoropropyl)isoindoline-1,3-dione (**41**): The compound was purified by columnchromatography (petroleum ether/EtOAc = 30/1), resulting in a white solid (31.9 mg) at 58% yield. **^{1}H NMR** (400 MHz, Chloroform-*d*) δ 7.87 – 7.70 (m, 4H), 7.72 (dd, J = 5.5, 3.1 Hz, 2H), 5.08 (dtt, J = 48.7, 7.3, 3.7 Hz, 1H), 4.08 – 3.96 (m, 1H), 3.92 – 3.77 (m, 1H), 2.65 – 2.38 (m, 2H). **^{13}C NMR** (101 MHz, Chloroform-*d*) δ 167.92, 134.44, 131.77, 125.17 (dq, J = 2.0, 276.8, Hz), 123.66, 84.83 (dq, J = 179.0, 3.2 Hz), 41.02 (d, J = 23.7 Hz), 37.49 (qd, J = 29.4, 21.7 Hz). **^{19}F NMR** (376 MHz, Chloroform-*d*) δ -63.95 (d, J = 7.3 Hz, 3F), -187.53 (q, J = 7.2 Hz, 1F). **ESI-HRMS** exact mass calculated for [$C_{12}H_9F_4NO_2Na^+$]: 298.0462, found 298.0469.

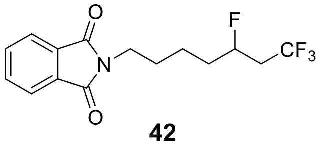

42

2-(5,7,7,7-Tetrafluoroheptyl)isoindoline-1,3-dione (**42**): The compound was purified by columnchromatography (petroleum ether/EtOAc = 30/1), resulting in a white solid (34.7 mg) at 55% yield. **^{1}H NMR** (400 MHz, Chloroform-*d*) δ 7.83 – 7.66 (m, 4H), 7.68 (dd, J = 5.5, 3.0 Hz, 2H), 4.76 (dtt, J = 49.1, 8.1, 3.6 Hz, 1H), 3.67 (t, J = 7.1 Hz, 2H), 2.57 – 2.19 (m, 2H), 1.78 – 1.38 (m, 6H). **^{13}C NMR** (101 MHz, DMSO-d_6) δ 167.94, 134.34, 131.61, 126.19 (q, J = 277.0 Hz), 122.98, 87.87 (dq, J = 169.8, 3.3 Hz), 37.83 (qd, J = 27.2, 21.0 Hz), 37.17, 33.52 (d, J = 20.2 Hz), 27.57, 21.44 (d, J = 4.3 Hz). **^{19}F NMR** (376 MHz, DMSO-d_6) δ -62.49 (d, J = 7.5 Hz, 3F), -182.16 (q, J = 7.6 Hz, 1F). **ESI-HRMS** exact mass calculated for [$C_{15}H_{15}F_4NO_2Na^+$]: 340.0931, found 340.0938.

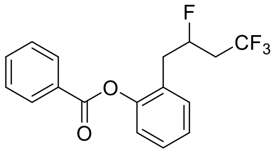

43

2-(2,4,4,4-Tetrafluorobutyl)phenyl benzoate (**43**): The compound was purified by columnchromatography (petroleum ether/EtOAc = 60/1), resulting in a colorless liquid (35.7 mg) at 55% yield. **^{1}H NMR** (400 MHz, Chloroform-*d*) δ 8.21 – 8.17 (m, 2H), 7.68 – 7.62 (m, 1H), 7.55 – 7.49 (m, 2H), 7.38 – 7.31 (m, 2H), 7.28 – 7.18 (m, 2H), 5.17 – 4.16 (m, 1H), 3.08 – 2.88 (m, 2H), 2.57 – 2.19 (m, 2H). **^{13}C NMR** (101 MHz, Chloroform-*d*) δ 165.07, 149.57, 134.06, 131.57, 130.24, 129.13, 128.91, 128.80, 127.88 (d, J = 4.5 Hz), 126.57, 125.45 (dq, J = 3.1, 276.9 Hz), 123.01, 87.11 (dq, J = 175.8, 3.0 Hz), 38.93 (qd, J = 28.7, 22.6 Hz), 35.81 (d, J = 22.1 Hz). **^{19}F NMR** (376 MHz, Chloroform-*d*) δ -63.98 (d, J = 7.5 Hz, 3F), -179.35 (q, J = 8.0 Hz, 1F). **ESI-HRMS** exact mass calculated for [$C_{17}H_{14}F_4O_2Na^+$]: 349.0822, found 349.0827.

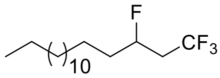

44

1,1,1,3-Tetrafluorooctane (**44**): The compound was purified by columnchromatography (petroleum ether/EtOAc = 80/1), resulting in a colorless liquid (40.5 mg) at 65% yield. **^{1}H NMR** (400 MHz, Chloroform-*d*) δ 4.79 (dtt, J = 49.0, 8.0, 3.7 Hz, 1H), 2.60 – 2.19 (m, 2H), 1.80 – 1.56 (m, 2H), 1.50 – 1.24 (m, 24H), 0.88 (t, J = 6.7 Hz, 3H). **^{13}C NMR** (101 MHz, Chloroform-*d*) δ 127.16 – 124.20 (m), 87.79 (dq, J = 172.1, 3.2 Hz), 39.56 (qd, J = 28.5, 23.0 Hz), 35.18 (d, J = 20.6

Hz), 32.09, 29.85, 29.83, 29.81, 29.81, 29.76, 29.66, 29.58, 29.52, 29.35, 24.73 (d, *J* = 4.3 Hz), 22.85, 14.27. **^{19}F NMR** (376 MHz, Chloroform-*d*) δ -64.17 (d, *J* = 7.3 Hz, 3F), -181.69 (q, *J* = 7.4 Hz, 1F). **ESI-HRMS** exact mass calculated for [$C_{17}H_{32}F_4H^+$]: 313.2513, found 313.2526.

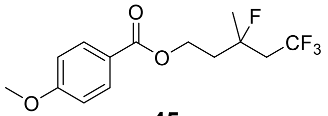

45

3,5,5,5-Tetrafluoro-3-methylpentyl 4-methoxybenzoate (**45**): The compound was purified by columnchromatography (petroleum ether/EtOAc = 60/1), resulting in a colorless liquid (22.2 mg) at 36% yield. **^{1}H NMR** (400 MHz, Chloroform-*d*) δ 8.00 – 7.94 (m, 2H), 6.94 – 6.90 (m, 2H), 4.52 – 4.41 (m, 2H), 3.85 (s, 3H), 2.68 – 2.50 (m, 2H), 2.32 – 2.12 (m, 2H), 1.57 (d, *J* = 22.1 Hz, 3H). **^{13}C NMR** (101 MHz, Chloroform-*d*) δ 166.19, 163.64, 131.69, 125.20 (dq, *J* = 8.4, 277.5 Hz), 122.43, 113.83, 92.39 (dq, *J* = 172.8, 2.2 Hz), 59.78 (d, *J* = 6.2 Hz), 55.54, 43.28 (qd, *J* = 27.9, 26.1 Hz), 38.74 (dd, *J* = 22.5, 1.6 Hz), 24.80 (dd, *J* = 24.2, 1.8 Hz). **^{19}F NMR** (376 MHz, Chloroform-*d*) δ -60.78 (d, *J* = 8.9 Hz, 3F), -143.12 (q, *J* = 8.9 Hz, 1F). **ESI-HRMS** exact mass calculated for [$C_{14}H_{16}F_4O_3Na^+$]: 331.0928, found 331.0938.

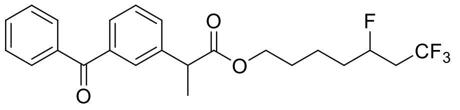

46

5,7,7,7-Tetrafluoroheptyl-2-(3-benzoylphenyl)propanoate (**46**): The compound was purified by columnchromatography (petroleum ether/EtOAc = 50/1), resulting in a colorless liquid (60.2 mg) at 71% yield. **^{1}H NMR** (400 MHz, Chloroform-*d*) δ 7.82 – 7.75 (m, 3H), 7.69 – 7.41 (m, 6H), 4.83 – 4.63 (m, 1H), 4.16 – 4.04 (m, 2H), 3.80 (q, *J* = 7.2 Hz, 1H), 2.55 – 2.37 (m, 1H), 2.35 – 2.15 (m, 1H), 1.75 – 1.57 (m, 4H), 1.54 (d, *J* = 7.2 Hz, 3H), 1.51 – 1.42 (m, 1H), 1.42 – 1.32 (m, 1H). **^{13}C NMR** (101 MHz, Chloroform-*d*) δ 196.53, 174.12, 141.07, 114.05, 138.06, 137.59, 132.63, 131.56, 130.15, 129.23, 129.22, 129.13, 128.59, 128.41, 125.51 (dq, *J* = 3.4, 276.8 Hz), 87.43 (dq, *J* = 172.6, 3.0 Hz), 64.52, 45.55, 39.41 (qd, *J* = 28.5, 22.9 Hz), 34.56 (d, *J* = 20.8 Hz), 28.22, 21.20 (d, *J* = 4.1 Hz), 18.47. **^{19}F NMR** (376 MHz, Chloroform-*d*) δ -64.08 (d, *J* = 7.4 Hz, 3F), -182.32 – -182.44 (m, 1F). **ESI-HRMS** exact mass calculated for [$C_{23}H_{24}F_4O_3Na^+$]: 447.1554, found 447.1565.

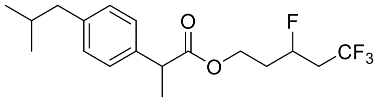

47

3,5,5,5-Tetrafluoropentyl-2-(4-isobutylphenyl)propanoate (**47**): The compound was purified by columnchromatography (petroleum ether/EtOAc = 80/1), resulting in a colorless liquid (36.9 mg) at 53% yield. **^{1}H NMR** (400 MHz, Chloroform-*d*) δ 7.18 (d, *J* = 8.1 Hz, 2H), 7.10 (d, *J* = 8.0 Hz, 2H), 4.76 (dtt, *J* = 48.5, 8.1, 4.0 Hz, 1H), 4.34 – 4.09 (m, 2H), 3.70 (q, *J* = 7.2 Hz, 1H), 2.53 – 2.33 (m, 3H), 2.30 – 2.10 (m, 1H), 2.01 – 1.78 (m, 3H), 1.50 (dd, *J* = 7.2, 1.3 Hz, 3H), 0.90 (d, *J* = 6.6 Hz, 6H). **^{13}C NMR** (101 MHz, Chloroform-*d*) δ 174.57, 174.57, 140.92, 140.91, 137.59, 137.55, 129.56, 129.55, 127.18, 127.17, 125.29 (dq, *J* = 3.1, 277.3 Hz), 85.76 – 83.78 (m), 59.92 – 59.72 (m), 45.30 – 45.17 (m), 45.13, 39.38 (qd, *J* = 28.8, 23.0 Hz), 34.12 (dd, *J* = 21.0, 5.0 Hz), 30.29, 22.49, 18.36. **^{19}F NMR** (376 MHz, Chloroform-*d*) δ -64.09 – -64.18 (m, 3F), -184.63 (dq, *J* = 68.4, 7.4 Hz, 1F); **ESI-HRMS** exact mass calculated for [$C_{18}H_{24}F_4O_2H^+$]: 349.1785, found 349.1793.

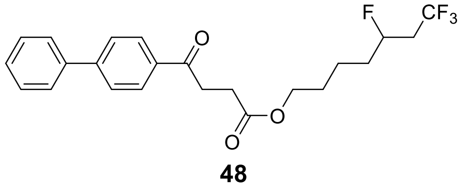

48

5,7,7,7-Tetrafluoroheptyl-4-([1,1'-biphenyl]-4-yl)-4-oxobutanoate (**48**): The compound was purified by columnchromatography (petroleum ether/EtOAc = 60/1), resulting in a solid with color

(36.5 mg) at 43% yield. **^{1}H NMR** (400 MHz, Chloroform-*d*) δ 8.04 (d, *J* = 8.4 Hz, 2H), 7.67 (d, *J* = 8.4 Hz, 2H), 7.63 – 7.59 (m, 2H), 7.48 – 7.42 (m, 2H), 7.41 – 7.36 (m, 1H), 4.80 (dtt, *J* = 49.2, 8.1, 3.5 Hz, 1H), 4.13 (t, *J* = 6.4 Hz, 2H), 3.31 (t, *J* = 6.5 Hz, 2H), 2.77 (t, *J* = 6.5 Hz, 2H), 2.57 – 2.22 (m, 2H), 1.80 – 1.41 (m, 6H). **^{13}C NMR** (101 MHz, Chloroform-*d*) δ 197.56, 172.80, 145.71, 139.67, 135.21, 128.92, 128.56, 128.22, 127.16, 127.13, 125.53 (dq, *J* = 2.8, 276.9 Hz), 87.39 (dq, *J* = 172.4, 3.1 Hz), 64.06, 39.16 (qd, *J* = 28.4, 22.7 Hz), 34.43 (d, *J* = 20.7 Hz), 33.30, 28.19, 28.14, 21.10 (d, *J* = 4.3 Hz). **^{19}F NMR** (376 MHz, Chloroform-*d*) δ -63.99 (d, *J* = 7.7 Hz, 3F), -182.25 (q, *J* = 7.9 Hz, 1F); **ESI-HRMS** exact mass calculated for [$C_{23}H_{24}F_4O_3Na^+$]: 477.1554, found 477.1561.

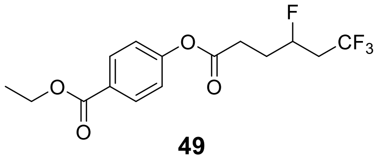

49

Ethyl 4-((4,6,6,6-tetrafluorohexanoyl)oxy)benzoate (**49**): The compound was purified by columnchromatography (petroleum ether/EtOAc = 60/1), resulting in a white solid (26.9 mg) at 40% yield. **^{1}H NMR** (400 MHz, Chloroform-*d*) δ 8.11 – 8.05 (m, 2H), 7.20 – 7.13 (m, 2H), 4.95 (dtt, *J* = 49.1, 8.1, 4.1 Hz, 1H), 4.38 (q, *J* = 7.2 Hz, 2H), 2.88 – 2.73 (m, 2H), 2.70 – 2.52 (m, 1H), 2.49 – 2.32 (m, 1H), 2.20 – 2.06 (m, 2H), 1.39 (t, *J* = 7.1 Hz, 3H). **^{13}C NMR** (101 MHz, Chloroform-*d*) δ 170.66, 165.89, 154.12, 131.29, 128.38, 125.29 (dq, *J* = 4.2, 276.9 Hz), 121.55, 86.73 (dq, *J* = 173.2, 3.2 Hz), 61.26, 39.61 (qd, *J* = 28.8, 22.8 Hz), 30.16 (d, *J* = 20.7 Hz), 29.67 (d, *J* = 4.1 Hz), 14.45; **^{19}F NMR** (376 MHz, Chloroform-*d*) δ -64.04 (d, *J* = 7.4 Hz, 3F), -184.67 (q, *J* = 7.3 Hz, 1F); **ESI-HRMS** exact mass calculated for [$C_{15}H_{16}F_4O_4Na^+$]: 359.0877, found 359.0877.

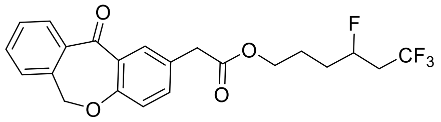

50

4,6,6,6-Tetrafluorohexyl-2-(11-oxo-6,11-dihydrodibenzo[b,e]oxepin-2-yl)acetate (**50**): The compound was purified by columnchromatography (petroleum ether/EtOAc = 60/1), resulting in a colorless liquid (41.6 mg) at 49% yield. **^{1}H NMR** (400 MHz, Chloroform-*d*) δ 8.12 (d, *J* = 2.4 Hz, 1H), 7.91 – 7.86 (m, 1H), 7.59 – 7.34 (m, 4H), 7.06 – 7.01 (m, 1H), 5.19 (s, 2H), 4.81 (dtt, *J* = 49.1, 7.7, 3.7 Hz, 1H), 4.21 – 4.10 (m, 2H), 3.64 (s, 2H), 2.61 – 2.20 (m, 2H), 1.91 – 1.60 (m, 4H). **^{13}C NMR** (101 MHz, Chloroform-*d*) δ 190.92, 171.43, 160.63, 140.51, 136.40, 135.68, 132.95, 132.52, 129.60, 129.41, 127.97, 127.85, 125.45 (dq, *J* = 3.6, 276.9 Hz), 125.30, 121.23, 87.27 (dq, *J* = 172.9, 3.2 Hz), 73.77, 64.23, 40.38, 39.50 (qd, *J* = 28.6, 22.9 Hz), 31.71 (d, *J* = 21.0 Hz), 24.16 (d, *J* = 4.0 Hz). **^{19}F NMR** (376 MHz, Chloroform-*d*) δ -64.07 (d, *J* = 7.5 Hz, 3F), -182.91 (q, *J* = 7.9 Hz, 1F). **ESI-HRMS** exact mass calculated for [$C_{22}H_{20}F_4O_4Na^+$]: 447.1190, found 447.1193.

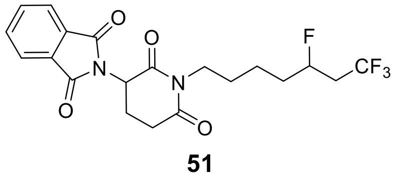

51

2-(2,6-Dioxo-1-(5,7,7,7-tetrafluoroheptyl)piperidin-3-yl)isoindoline-1,3-dione (**51**): The compound was purified by columnchromatography (petroleum ether/EtOAc = 30/1), resulting in a solid with color (64.2 mg) at 75% yield. **^{1}H NMR** (400 MHz, Chloroform-*d*) δ 7.86 – 7.77 (m, 2H), 7.76 – 7.67 (m, 2H), 5.02 – 4.93 (m, 1H), 4.86 – 4.63 (m, 1H), 3.81 – 3.74 (m, 2H), 2.97 – 2.86 (m, 1H), 2.80 – 2.66 (m, 2H), 2.54 – 2.16 (m, 2H), 2.14 – 2.03 (m, 1H), 1.79 – 1.30 (m, 6H). **^{13}C NMR** (101 MHz, Chloroform-*d*) δ 170.97, 168.73, 167.38, 134.44, 131.68, 129.77 – 121.37 (m), 123.64, 88.40 – 86.49 (m), 50.07, 40.08 – 39.84 (m), 39.70 – 38.40 (m), 34.39 (d, *J* = 22.3 Hz), 31.88, 27.29 (d, *J* = 7.5 Hz), 21.92, 21.61 (d, *J* = 4.2 Hz). **^{19}F NMR** (376 MHz, Chloroform-*d*) δ -64.04 – -64.13 (m, 3F), -181.90 (dq, *J* = 65.3, 7.4 Hz, 1F). **ESI-HRMS** exact mass calculated for [$C_{20}H_{20}F_4N_2O_4Na^+$]: 451.1251, found 451.1254.

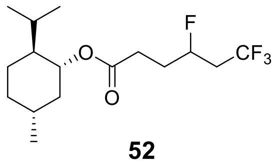

52

(1*R*,2*S*,5*R*)-2-Isopropyl-5-methylcyclohexyl 4,6,6,6-tetrafluorohexanoate (**52**): The compound was purified by columnchromatography (petroleum ether/EtOAc = 80/1), resulting in a colorless liquid (40.4 mg) at 62% yield. **^{1}H NMR** (400 MHz, Chloroform-*d*) δ 4.96 – 4.75 (m, 1H), 4.70 (td, *J* = 10.9, 4.4 Hz, 1H), 2.63 – 2.42 (m, 2H), 2.42 – 2.23 (m, 2H), 2.06 – 1.92 (m, 3H), 1.88 – 1.78 (m, 1H), 1.71 – 1.63 (m, 2H), 1.55 – 1.41 (m, 1H), 1.41 – 1.32 (m, 1H), 1.11 – 0.84 (m, 9H), 0.75 (d, *J* = 7.0 Hz, 3H). **^{13}C NMR** (101 MHz, Chloroform-*d*) δ 172.13, 172.11, 125.40 (dq, *J* = 3.7, 276.8 Hz), 87.81 – 85.90 (m), 74.80, 74.78, 47.15, 41.05, 41.02, 39.59 (qd, *J* = 28.8, 22.8 Hz), 34.37, 31.54, 30.43 (d, *J* = 21.0 Hz), 29.88 – 29.65 (m), 26.50, 26.48, 23.60, 23.57, 21.12, 20.86, 16.43, 16.41; **^{19}F NMR** (376 MHz, Chloroform-*d*) δ -64.14 – -64.18 (m, 3F), -184.41 (q, *J* = 7.8 Hz, 1F); **ESI-HRMS** exact mass calculated for [$C_{16}H_{26}F_4O_2Na^+$]: 349.1761, found 349.1770.

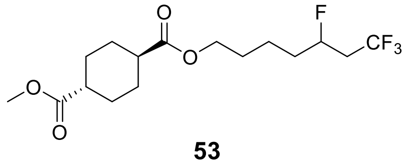

53

1-Methyl-4-(5,7,7,7-tetrafluoroheptyl) (1*r*,4*r*)-cyclohexane-1,4-dicarboxylate (**53**): The compound was purified by columnchromatography (petroleum ether/EtOAc = 60/1), resulting in a solid with color (22.8 mg) at 32% yield. **^{1}H NMR** (400 MHz, Chloroform-*d*) δ 4.78 (dtt, *J* = 48.9, 8.3, 3.5 Hz, 1H), 4.06 (t, *J* = 6.4 Hz, 2H), 3.65 (s, 3H), 2.60 – 2.43 (m, 1H), 2.40 – 2.19 (m, 3H), 2.08 – 1.97 (m, 4H), 1.81 – 1.49 (m, 6H), 1.46 – 1.39 (m, 4H). **^{13}C NMR** (101 MHz, Chloroform-*d*) δ 175.98, 175.55, 125.50 (dq, *J* = 3.6, 276.8 Hz), 87.46 (dq, *J* = 172.5, 3.2 Hz), 63.93, 51.69, 42.61, 42.50, 39.49 (qd, *J* = 28.5, 23.1 Hz), 34.66 (q, *J* = 20.9 Hz), 28.32, 28.14, 28.12, 21.31 (d, *J* = 4.1 Hz). **^{19}F NMR** (376 MHz, Chloroform-*d*) δ -64.14 (d, *J* = 7.5 Hz, 3F), -182.45 (q, *J* = 7.8 Hz, 1F). **ESI-HRMS** exact mass calculated for [$C_{16}H_{24}F_4O_4Na^+$]: 379.1503, found 379.1512.

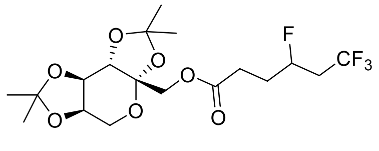

54

((3a*S*,5a*R*,8a*R*,8b*S*)-2,2,7,7-Tetramethyltetrahydro-3a*H*-bis([1,3]dioxolo)[4,5-b:4',5'-d]pyran-3a-yl)methyl 4,6,6,6-tetrafluorohexanoate (**54**): The compound was purified by columnchromatography (petroleum ether/EtOAc = 10/1), resulting in a colorless liquid (38.7 mg) at 45% yield. **^{1}H NMR** (400 MHz, Chloroform-*d*) δ 4.95 – 4.75 (m, 1H), 4.58 (dd, *J* = 7.9, 2.6 Hz, 1H), 4.42 (dd, *J* = 11.6, 2.0 Hz, 1H),4.28 – 4.25 (m, 1H), 4.23 – 4.20 (m, 1H), 4.05 – 4.00 (m, 1H), 3.91 – 3.85 (m, 1H), 3.77 – 3.71 (m, 1H), 2.62 – 2.22 (m, 4H), 2.11 – 1.92 (m, 2H), 1.51 (s, 3H), 1.45 (s, 3H), 1.37 (s, 3H), 1.32 (s, 3H). **^{13}C NMR** (101 MHz, Chloroform-*d*) δ 171.85, 125.33 (dq, *J* = 3.4, 276.8 Hz), 109.22, 108.86, 108.84, 101.53, 86.63 (dq, *J* = 173.0, 3.2 Hz), 70.83, 70.71, 70.13, 65.70, 65.65, 61.35, 40.10 – 38.86 (m), 30.08 (d, *J* = 20.9 Hz), 29.21 – 29.08 (m), 26.50, 25.95, 25.23, 24.11. **^{19}F NMR** (376 MHz, Chloroform-*d*) δ -64.06 – -64.12 (m, 3F), -184.85 (q, *J* = 7.9 Hz, 1F); **ESI-HRMS** exact mass calculated for [$C_{18}H_{26}F_4O_7Na^+$]: 453.1507, found 453.1514.

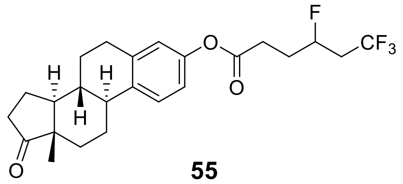

55

(8*R*,9*S*,13*S*,14*S*)-13-Methyl-17-oxo-7, 8, 9, 11, 12, 13, 14, 15, 16, 17-decahydro-6H-cyclopenta[a]phen-anthren-3-yl 5,7,7,7-tetra-fluoroheptanoate (**55**): The compound was purified by columnchromatography (petroleum ether/EtOAc = 60/1), resulting in a white solid (44.2 mg) at 50% yield. **^{1}H NMR** (400 MHz, Chloroform-*d*) δ 7.29 (d, *J* = 8.5 Hz, 1H), 6.85 (d, *J* = 2.5 Hz, 1H), 6.81 (s, 1H), 5.06 – 4.84 (m, 1H), 2.94 – 2.86 (m, 2H), 2.79 – 2.72 (m, 2H), 2.66 – 2.21 (m, 5H), 2.20 – 1.93 (m, 6H), 1.69 – 1.38 (m, 6H), 0.90 (s, 3H). **^{13}C NMR** (101 MHz, Chloroform-*d*) δ 171.43, 148.45, 138.20, 137.66, 126.55, 125.31 (dq, *J* = 3.7, 276.8 Hz), 121.53, 118.69, 86.73 (dq, *J* = 173.2, 3.3 Hz), 50.49, 48.02, 44.23, 39.52 (qd, *J* = 28.9, 22.7 Hz), 38.06, 35.94, 31.63, 30.21 (d, *J* = 20.9 Hz), 29.55, 29.51, 29.49, 26.40, 25.83, 21.67, 13.90. **^{19}F NMR** (376 MHz, Chloroform-*d*) δ -64.03 (d, *J* = 7.6 Hz, 3F), -184.61 (q, *J* = 7.6 Hz, 1F). **ESI-HRMS** exact mass calculated for [$C_{24}H_{28}F_4O_3Na^+$]: 463.1867, found 463.1862.

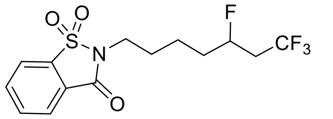

56

2-(5,7,7,7-Tetrafluoroheptyl)benzo[d]isothiazol-3(2H)-one 1,1-dioxide (**56**): The compound was purified by columnchromatography (petroleum ether/EtOAc = 30/1), resulting in a liquid with color (38.8 mg) at 55% yield. **^{1}H NMR** (400 MHz, Chloroform-*d*) δ 8.07 – 8.03 (m, 1H), 7.94 – 7.90 (m, 1H), 7.89 – 7.79 (m, 2H), 4.81 (dtt, *J* = 48.8, 8.2, 3.5 Hz, 1H), 3.79 (t, *J* = 7.3 Hz, 2H), 2.62 – 2.20 (m, 2H), 1.96 – 1.87 (m, 2H), 1.84 – 1.45 (m, 4H). **^{13}C NMR** (101 MHz, Chloroform-*d*) δ 159.12, 137.79, 134.90, 134.48, 127.46, 125.53 (dq, *J* = 3.4, 276.8 Hz), 125.30, 121.05, 87.44 (dq, *J* = 172.6, 3.2 Hz), 39.46 (qd, *J* = 28.5, 22.9 Hz), 39.04, 34.47 (d, *J* = 20.9 Hz), 28.07, 21.99 (d, *J* = 4.2 Hz); **^{19}F NMR** (376 MHz, Chloroform-*d*) δ -64.10 (d, *J* = 7.5 Hz, 3F), -182.30 (q, *J* = 7.3 Hz, 1F); **ESI-HRMS** exact mass calculated for [$C_{14}H_{15}F_4NO_3SNa^+$]: 376.0601, found 376.0604.

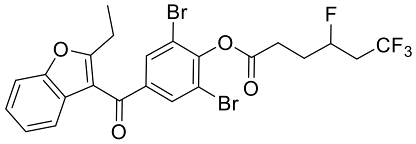

57

2,6-Dibromo-4-(2-ethylbenzofuran-3-carbonyl)phenyl 4,6,6,6-tetrafluorohexanoate (**57**): The compound was purified by columnchromatography (petroleum ether/EtOAc = 50/1), resulting in a liquid with color (53.2 mg) at 45% yield. **^{1}H NMR** (400 MHz, Chloroform-*d*) δ 8.03 (s, 2H), 7.51 (d, *J* = 8.1 Hz, 1H), 7.41 (d, *J* = 7.7 Hz, 1H), 7.36 – 7.22 (m, 2H), 5.10 – 4.90 (m, 1H), 3.01 – 2.87 (m, 4H), 2.69 – 2.32 (m, 2H), 2.28 – 2.11 (m, 2H), 1.37 (t, *J* = 7.5 Hz, 3H). **^{13}C NMR** (101 MHz, Chloroform-*d*) δ 187.84, 168.58, 167.52, 153.82, 149.35, 139.22, 133.19, 126.34, 125.28 (dq, *J* = 3.8, 276.9 Hz), 124.95, 124.14, 121.12, 118.20, 115.31, 111.34, 86.45 (dq, *J* = 173.6, 3.2 Hz), 39.58 (qd, *J* = 28.9, 22.7 Hz), 30.02 (d, *J* = 21.0 Hz), 29.19 (d, *J* = 4.3 Hz), 22.20, 12.32. **^{19}F NMR** (376 MHz, Chloroform-*d*) δ -63.95 (d, *J* = 7.3 Hz, 3F), -184.91 (q, *J* = 7.4 Hz, 1F); **ESI-HRMS** exact mass calculated for [$C_{23}H_{18}Br_2F_4O_4Na^+$]: 614.9400, found 614.9404.

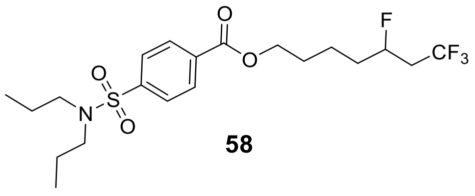

58

5,7,7,7-Tetrafluoroheptyl 4-(N,N-dipropylsulfamoyl)benzoate (**58**): The compound was purified by columnchromatography (petroleum ether/EtOAc = 10/1), resulting in a colorless liquid (54.6 mg) at 60% yield. **^{1}H NMR** (400 MHz, Chloroform-*d*) δ 8.14 – 7.78 (m, 4H), 4.77 (dtt, *J* = 49.1, 8.2, 3.4 Hz, 1H), 4.31 (t, *J* = 6.4 Hz, 2H), 3.04 (t, *J* = 8.0 Hz, 4H), 2.57 – 2.18 (m, 2H), 1.83 – 1.43 (m, 10H), 0.80 (t, *J* = 7.4 Hz, 6H). **^{13}C NMR** (101 MHz, Chloroform-*d*) δ 165.14, 144.19, 133.55, 130.13, 126.95, 125.45 (dq, *J* = 3.0, 276.8 Hz), 87.39 (dq, *J* = 172.4, 3.1 Hz), 65.11, 49.90, 39.25 (qd, *J* = 28.4, 22.7 Hz), 34.47 (d, *J* = 20.7 Hz), 28.18, 21.88, 21.28 (d, *J* = 4.1 Hz), 11.04. **^{19}F NMR**

(376 MHz, Chloroform-*d*) δ -64.14 (d, *J* = 7.1 Hz, 3F), -182.74 (q, *J* = 7.4 Hz, 1F). **ESI-HRMS** exact mass calculated for [$C_{20}H_{29}F_4NO_4SNa^+$]: 478.1646, found 478.1653.

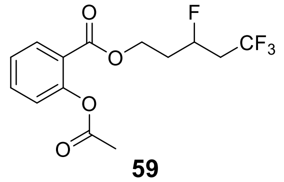

59

3,5,5,5-Tetrafluoropentyl 2-acetoxybenzoate (**59**): The compound was purified by columnchromatography (petroleum ether/EtOAc = 60/1), resulting in a colorless liquid (31.5 mg) at 49% yield. **^{1}H NMR** (400 MHz, Chloroform-*d*) δ 7.98 (dd, *J* = 7.8, 1.6 Hz, 1H), 7.63 – 7.52 (m, 1H), 7.35 – 7.29 (m, 1H), 7.15 – 7.05 (m, 1H), 5.12 – 4.90 (m, 1H), 4.54 – 4.34 (m, 2H), 2.68 – 2.28 (m, 5H), 2.18 – 1.99 (m, 2H). **^{13}C NMR** (101 MHz, Chloroform-*d*) δ 169.74, 164.28, 150.84, 134.20, 131.62, 126.18, 125.30 (dq, *J* = 3.4, 276.8 Hz), 123.97, 123.08, 84.84 (dq, *J* = 173.3, 3.3 Hz), 60.39 (d, *J* = 4.7 Hz), 39.47 (qd, *J* = 28.8, 22.5 Hz), 34.22 (d, *J* = 20.8 Hz), 21.04. **^{19}F NMR** (376 MHz, Chloroform-*d*) δ -63.97 (d, *J* = 7.4 Hz, 3F), -184.90 (q, *J* = 7.4 Hz, 1F). **ESI-HRMS** exact mass calculated for [$C_{14}H_{14}F_4O_4Na^+$]: 345.0720, found 345.0726.

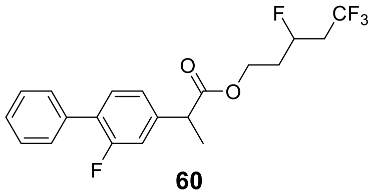

60

3,5,5,5-Tetrafluoropentyl 2-(2-fluoro-[1,1'-biphenyl]-4-yl)propanoate (**60**): The compound was purified by columnchromatography (petroleum ether/EtOAc = 80/1), resulting in a colorless liquid (45.3 mg) at 59% yield. **^{1}H NMR** (400 MHz, Chloroform-*d*) δ 7.56 (d, *J* = 7.3 Hz, 2H), 7.50 – 7.35 (m, 4H), 7.20 – 7.10 (m, 2H), 4.96 – 4.73 (m, 1H), 4.36 – 4.20 (m, 2H), 3.79 (q, *J* = 7.1 Hz, 1H), 2.61 – 2.16 (m, 2H), 2.08 – 1.85 (m, 2H), 1.60 – 1.52 (m, 3H). **^{13}C NMR** (101 MHz, Chloroform-*d*) δ 173.77, 159.82 (d, *J* = 248.5 Hz), 141.68 (d, *J* = 4.0 Hz), 141.60 (d, *J* = 4.0 Hz), 135.50, 131.02 (d, *J* = 3.8 Hz), 129.03 (d, *J* = 2.9 Hz), 128.56, 128.11 (d, *J* = 13.5 Hz), 127.81, 125.29 (dq, *J* = 3.5, 277.0 Hz), 123.57 (d, *J* = 3.3 Hz), 85.74 – 83.75 (m), 60.30 – 60.10 (m), 45.12 – 45.00 (m), 39.35 (qd, *J* = 28.7, 22.8 Hz), 34.04 (dd, *J* = 21.0, 5.1 Hz), 18.25. **^{19}F NMR** (376 MHz, Chloroform-*d*) δ -64.08 – -64.14 (m, 3F), -117.43 – -117.49 (m, 1F), -184.34 – -184.68 (m, 1F). **ESI-HRMS** exact mass calculated for [$C_{20}H_{19}F_5O_2H^+$]: 387.1378, found 387.1385.

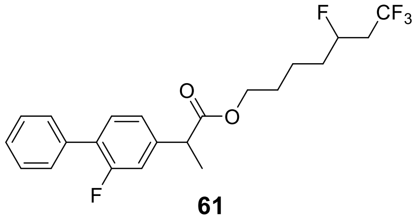

61

5,7,7,7-Tetrafluoroheptyl 2-(2-fluoro-[1,1'-biphenyl]-4-yl)propanoate (**61**): The compound was purified by columnchromatography (petroleum ether/EtOAc = 80/1), resulting in a colorless liquid (57.9 mg) at 70% yield. **^{1}H NMR** (400 MHz, Chloroform-*d*) δ 7.58 – 7.50 (m, 2H), 7.48 – 7.34 (m, 4H), 7.19 – 7.10 (m, 2H), 4.77 (dtt, *J* = 48.9, 7.6, 3.2 Hz, 1H), 4.13 (t, *J* = 6.4 Hz, 2H), 3.77 (q, *J* = 7.1 Hz, 1H), 2.58 – 2.15 (m, 2H), 1.78 – 1.57 (m, 4H), 1.55 (d, *J* = 7.2 Hz, 3H), 1.53 – 1.34 (m, 2H). **^{13}C NMR** (101 MHz, Chloroform-*d*) δ 174.07, 161.01, 158.55, 141.98 (d, *J* = 7.7 Hz), 135.54, 130.90 (d, *J* = 3.9 Hz), 129.02 (d, *J* = 3.0 Hz), 128.56, 127.93 (d, *J* = 13.5 Hz), 127.79, 125.51 (dq, *J* = 3.5, 276.9 Hz), 123.64 (d, *J* = 3.3 Hz), 87.44 (dq, *J* = 172.7, 3.3 Hz), 64.57, 45.17 (d, *J* = 1.2 Hz), 39.41 (qd, *J* = 28.5, 23.0 Hz), 34.57 (d, *J* = 20.8 Hz), 28.21, 21.22 (d, *J* = 4.1 Hz), 18.38. **^{19}F NMR** (376 MHz, Chloroform-*d*) δ -64.09 (d, *J* = 7.7 Hz, 3F), -117.66 (s, 1F), -182.41 (q, *J* = 7.7 Hz, 1F). **ESI-HRMS** exact mass calculated for [$C_{22}H_{23}F_5O_2H^+$]: 415.1691, found 415.1688.

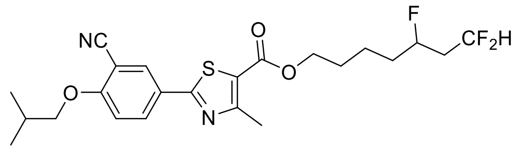

63

5,7,7-Trifluoroheptyl 2-(3-cyano-4-isobutoxyphenyl)-4-methylthiazole-5-carboxylate (**63**): The compound was purified by columnchromatography (petroleum ether/EtOAc = 50/1), resulting in a white solid (64.6 mg) at 69% yield. **^{1}H NMR** (400 MHz, Chloroform-*d*) δ 8.15 – 8.12 (m, 1H), 8.08 – 8.03 (m, 1H), 6.99 (d, *J* = 8.9 Hz, 1H), 5.98 (tdd, *J* = 56.6, 6.7, 2.8 Hz, 1H), 4.84 – 4.61 (m, 1H), 4.29 (t, *J* = 6.4 Hz, 2H), 3.88 (d, *J* = 6.5 Hz, 2H), 2.73 (s, 3H), 2.29 – 1.94 (m, 3H), 1.85 – 1.48 (m, 6H), 1.07 (d, *J* = 6.7 Hz, 6H). **^{13}C NMR** (101 MHz, Chloroform-*d*) δ 167.27, 162.55, 162.04, 161.29, 132.61, 132.08, 126.00, 121.70, 115.44, 115.10 (dt, *J* = 4.9, 233.8 Hz), 112.70, 102.99, 88.92 – 88.02 (m), 75.75, 64.97, 40.04 (q, *J* = 21.3 Hz), 34.74 (d, *J* = 20.5 Hz), 28.38, 28.23, 21.52 (d, *J* = 4.3 Hz), 19.11, 17.55. **^{19}F NMR** (376 MHz, Chloroform-*d*) δ -116.06 – -118.24 (m, 2F), -183.71 (s, 1F). **ESI-HRMS** exact mass calculated for [$C_{23}H_{27}F_3N_2O_3SNa^+$]: 491.1587, found 491.1592.

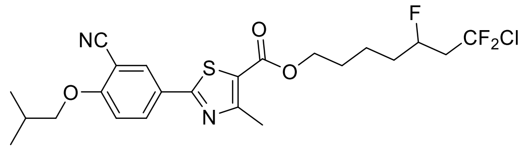

64

7-Chloro-5,7,7-trifluoroheptyl 2-(3-cyano-4-isobutoxyphenyl)-4-methylthiazole-5-carboxylate (**64**): The compound was purified by columnchromatography (petroleum ether/EtOAc = 50/1), resulting in a white solid (59.2 mg) at 59% yield. **^{1}H NMR** (400 MHz, Chloroform-*d*) δ 8.14 – 8.11 (m, 1H), 8.07 – 8.02 (m, 1H), 6.98 (d, *J* = 8.9 Hz, 1H), 4.87 (dtt, *J* = 49.2, 8.0, 3.4 Hz, 1H), 4.29 (t, *J* = 6.4 Hz, 2H), 3.87 (d, *J* = 6.5 Hz, 2H), 2.73 (d, 3H), 2.82 – 2.40 (m, 2H), 2.23 – 2.11 (m, 1H), 1.86 – 1.45 (m, 6H), 1.07 (d, *J* = 6.7 Hz, 6H). **^{13}C NMR** (101 MHz, Chloroform-*d*) δ 167.25, 162.54, 162.00 161.28, 132.59, 132.03, 127.76 (dt, *J* = 2.7, 292.6 Hz), 125.97, 121.66, 115.42, 112.69, 102.97, 88.21 (dt, *J* = 173.3, 2.9 Hz), 75.74, 64.89, 47.45 – 46.60 (m), 34.63 (d, *J* = 20.9 Hz), 28.32, 28.21, 21.37 (d, *J* = 4.2 Hz), 19.09, 17.53. **^{19}F NMR** (376 MHz, Chloroform-*d*) δ -48.57 – -50.31 (m, 2F), -181.86 (t, *J* = 9.0 Hz, 1F). **ESI-HRMS** exact mass calculated for [$C_{23}H_{26}ClF_3N_2O_3SNa^+$]: 525.1197, found 525.1199.

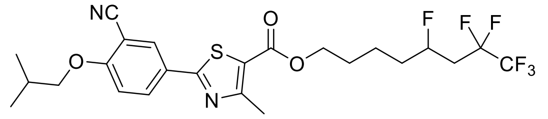

65

5,7,7,8,8,8-Hexafluorooctyl 2-(3-cyano-4-isobutoxyphenyl)-4-methylthiazole-5-carboxylate (**65**): The compound was purified by columnchromatography (petroleum ether/EtOAc = 50/1), resulting in a white solid (72.3 mg) at 68% yield. **^{1}H NMR** (400 MHz, Chloroform-*d*) δ 8.15 (d, *J* = 2.2 Hz, 1H), 8.09 – 8.04 (m, 1H), 7.02 – 6.97 (m, 1H), 4.93 (dtt, *J* = 48.8, 8.0, 3.4 Hz, 1H), 4.31 (t, *J* = 6.4 Hz, 2H), 3.89 (d, *J* = 6.6 Hz, 2H), 2.75 (s, 3H), 2.59 – 2.12 (m, 3H), 1.93 – 1.54 (m, 6H), 1.08 (d, *J* = 6.7 Hz, 6H). **^{13}C NMR** (101 MHz, Chloroform-*d*) δ 167.35, 162.61, 162.08, 161.39, 132.64, 132.14, 126.05, 121.71, 120.80 – 117.10 (m), 115.46, 114.85 – 114.25 (m), 112.72, 103.07, 86.70 (dt, *J* = 173.2, 3.0 Hz), 75.81, 64.91, 36.45 (dt, *J* = 23.2, 21.4 Hz), 35.16 (d, *J* = 20.9 Hz), 28.36, 28.27, 21.44 (d, *J* = 4.1 Hz), 19.13, 17.58. **^{19}F NMR** (376 MHz, Chloroform-*d*) δ -85.79 – -85.85 (m, 3F), -116.95 – -117.02 (m, 2F), -180.70 – -180.78 (m, 1F). **ESI-HRMS** exact mass calculated for [$C_{24}H_{26}F_6N_2O_3SNa^+$]: 559.1461, found 559.1466.

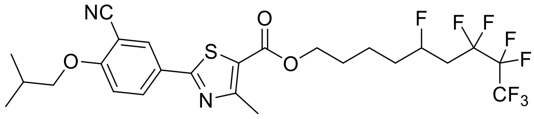

66

5,7,7,8,8,9,9,9-Octafluorononyl 2-(3-cyano-4-isobutoxyphenyl)-4-methylthiazole-5-carboxylate (**66**): The compound was purified by columnchromatography (petroleum ether/EtOAc = 50/1), resulting in a white solid (83.2 mg) at 71% yield. ^{1}**H NMR** (400 MHz, Chloroform-*d*) δ 8.17 (d, *J* = 2.0 Hz, 1H), 8.11 – 8.06 (m, 1H), 7.00 (d, *J* = 8.9 Hz, 1H), 4.96 (dtt, *J* = 48.9, 7.9, 3.4 Hz, 1H), 4.32 (t, *J* = 6.4 Hz, 2H), 3.90 (d, *J* = 6.5 Hz, 2H), 2.76 (s, 3H), 2.66 – 2.13 (m, 3H), 1.93 – 1.53 (m, 6H), 1.09 (d, *J* = 6.7 Hz, 6H). ^{13}**C NMR** (101 MHz, Chloroform-*d*) δ 167.18, 162.50, 161.94, 161.22, 132.53, 131.92, 125.91, 122.46 – 105.35 (m, $CF_3CF_2CF_2$), 121.62, 115.37, 112.64, 102.89, 86.58 (dt, *J* = 173.6, 2.7 Hz), 75.70, 64.84, 36.18 (q, *J* = 21.9 Hz), 35.11 (d, *J* = 20.9 Hz), 28.27, 28.18, 21.36 (d, *J* = 4.1 Hz), 18.99, 17.44. ^{19}**F NMR** (376 MHz, Chloroform-*d*) δ -80.39 (t, *J* = 11.3 Hz, 3F), -114.00 – -114.24 (m, 2F), -127.80 – -127.92 (m, 2F), -180.48 – -180.57 (m, 1F). **ESI-HRMS** exact mass calculated for [$C_{25}H_{26}F_8N_2O_3SNa^+$]: 609.1429, found 609.1439.

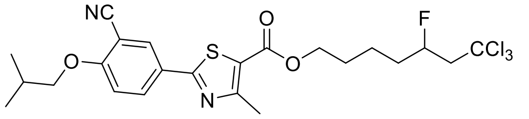

67

7,7,7-Trichloro-5-fluoroheptyl-2-(3-cyano-4-isobutoxyphenyl)-4-methylthiazole-5-carboxylate (**67**): The compound was purified by columnchromatography (petroleum ether/EtOAc = 50/1), resulting in a white solid (57.7 mg) at 54% yield. ^{1}**H NMR** (400 MHz, Chloroform-*d*) δ 8.16 – 8.13 (m, 1H), 8.09 – 8.04 (m, 1H), 6.99 (d, *J* = 8.9 Hz, 1H), 4.34 – 4.24 (m, 3H), 3.88 (d, *J* = 6.5 Hz, 2H), 3.33 – 3.07 (m, 2H), 2.76 – 2.73 (m, 3H), 2.25 – 2.13 (m, 1H), 2.08 – 1.57 (m, 6H), 1.07 (d, *J* = 6.7 Hz, 6H). ^{13}**C NMR** (101 MHz, Chloroform-*d*) δ 167.31, 162.57, 162.05, 161.34, 132.63, 132.12, 126.02, 121.72, 115.45, 112.71, 103.04, 96.89, 77.55 – 75.72 (m), 64.91, 62.28, 57.46, 38.52, 28.24, 28.04, 22.71, 19.15, 17.61. ^{19}**F NMR** (376 MHz, Chloroform-*d*) δ -178.46 (s, 1F). **ESI-HRMS** exact mass calculated for [$C_{23}H_{26}Cl_3FN_2O_3SH^+$]: 535.0787, found 535.0788.

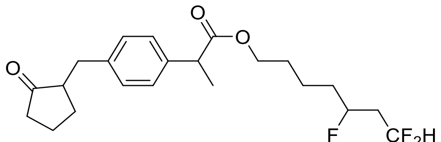

68

2-(4-(8,10,10-Trifluoro-3-oxodecan-2-yl)benzyl)cyclopentan-1-one (**68**): The compound was purified by columnchromatography (petroleum ether/EtOAc = 80/1), resulting in a colorless liquid (34.1 mg) at 45% yield. ^{1}**H NMR** (400 MHz, Chloroform-*d*) δ 7.23 – 7.09 (m, 4H), 5.95 (tdd, *J* = 56.5, 6.8, 2.8 Hz, 1H), 4.70 – 4.58 (m, 1H), 4.07 (t, *J* = 6.3 Hz, 2H), 3.68 (q, *J* = 7.1 Hz, 1H), 3.16 – 3.08 (m, 1H), 2.56 – 2.44 (m, 1H), 2.38 – 2.27 (m, 2H), 2.16 – 2.04 (m, 3H), 2.03 – 1.87 (m, 2H), 1.77 – 1.51 (m, 6H), 1.48 (d, *J* = 7.2 Hz, 3H), 1.44 – 1.28 (m, 2H). ^{13}**C NMR** (101 MHz, Chloroform-*d*) δ 220.30, 174.74, 139.00, 138.50, 129.21, 127.61, 115.13 (dt, *J* = 4.9, 238.6 Hz), 89.94 – 88.08 (m), 64.34, 51.07, 45.27, 40.40 – 39.60 (m), 38.29, 35.29, 34.69 (d, *J* = 20.5 Hz), 29.35, 28.26, 21.29 (d, *J* = 4.5 Hz), 20.65, 18.53. ^{19}**F NMR** (376 MHz, Chloroform-*d*) δ -116.00 – -118.41 (m, 2F), -183.54 – -183.61 (m, 1F). **ESI-HRMS** exact mass calculated for [$C_{22}H_{29}F_3O_3Na^+$]: 421.1961, found 421.1972.

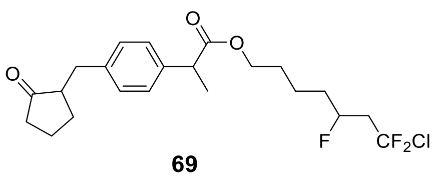

69

2-(4-(10-Chloro-8,10,10-trifluoro-3-oxodecan-2-yl)benzyl)cyclopentan-1-one (**69**): The compound was purified by columnchromatography (petroleum ether/EtOAc = 80/1), resulting in a

colorless liquid (47.1 mg) at 57% yield. **^{1}H NMR** (400 MHz, Chloroform-*d*) δ 7.23 – 7.09 (m, 4H), 4.78 (dtt, *J* = 49.2, 7.3, 3.1 Hz, 1H), 4.07 (t, *J* = 6.3 Hz, 2H), 3.68 (q, *J* = 7.1 Hz, 1H), 3.16 – 3.06 (m, 1H), 2.75 – 2.59 (m, 1H), 2.54 – 2.27 (m, 4H), 2.16 – 2.01 (m, 2H), 2.00 – 1.89 (m, 1H), 1.79 – 1.31 (m, 11H). **^{13}C NMR** (101 MHz, Chloroform-*d*) δ 220.27, 174.73, 139.01, 138.49, 129.21, 127.80 (dt, *J* = 2.8, 292.4 Hz), 127.60, 88.23 (dt, *J* = 173.3, 3.0 Hz), 64.27, 51.06, 47.40 – 46.60 (m), 45.26, 38.28, 35.30, 34.59 (d, *J* = 21.0 Hz), 29.35, 28.20, 21.16 (d, *J* = 4.3 Hz), 20.64, 18.53. **^{19}F NMR** (376 MHz, Chloroform-*d*) δ -48.50 – -50.44 (m, 2F), -181.68 – -181.76 (m, 1F). **ESI-HRMS** exact mass calculated for [$C_{22}H_{28}ClF_3O_3Na^+$]: 455.1571, found 455.1572.

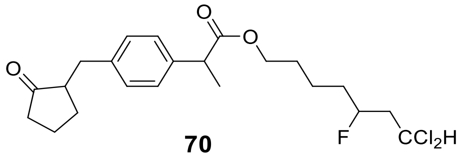

70

2-(4-(10,10-Dichloro-8-fluoro-3-oxodecan-2-yl)benzyl)cyclopentan-1-one (**70**): The compound was purified by columnchromatography (petroleum ether/EtOAc = 80/1), resulting in a colorless liquid (44.6 mg) at 54% yield. **^{1}H NMR** (400 MHz, Chloroform-*d*) δ 7.23 – 7.09 (m, 4H), 5.93 – 5.81 (m, 1H), 4.76 – 4.56 (m, 1H), 4.07 (t, *J* = 6.4 Hz, 2H), 3.68 (q, *J* = 7.1 Hz, 1H), 3.17 – 3.08 (m, 1H), 2.54 – 2.45 (m, 2H), 2.37 – 2.20 (m, 3H), 2.15 – 2.04 (m, 2H), 2.00 – 1.90 (m, 1H), 1.80 – 1.51 (m, 6H), 1.48 (d, *J* = 7.2 Hz, 3H), 1.45 – 1.27 (m, 2H). **^{13}C NMR** (101 MHz, Chloroform-*d*) δ 220.25, 174.73, 138.99, 138.49, 129.22, 127.60, 90.54 (d, *J* = 169.7 Hz), 69.69 (d, *J* = 4.5 Hz), 64.33, 51.07, 49.27 (d, *J* = 20.8 Hz), 45.26, 38.29, 35.32, 34.40 – 34.08 (m), 29.35, 28.29, 21.35 (d, *J* = 4.5 Hz), 20.66, 18.54. **^{19}F NMR** (376 MHz, Chloroform-*d*) δ -185.17 – -185.27 (m, 1F). **ESI-HRMS** exact mass calculated for [$C_{22}H_{29}Cl_2FO_3Na^+$]: 453.1370, found 453.1374.

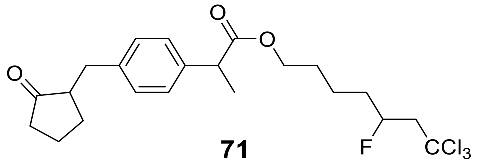

71

2-(4-(10,10,10-Trichloro-8-fluoro-3-oxodecan-2-yl)benzyl)cyclopentan-1-one (**71**): The compound was purified by columnchromatography (petroleum ether/EtOAc = 80/1), resulting in a colorless liquid (41.6 mg) at 46% yield. **^{1}H NMR** (400 MHz, Chloroform-*d*) δ 7.23 – 7.09 (m, 4H), 4.98 – 4.78 (m, 1H), 4.08 (t, *J* = 6.4 Hz, 2H), 3.68 (q, *J* = 7.1 Hz, 1H), 3.17 – 3.04 (m, 2H), 2.89 – 2.78 (m, 1H), 2.55 – 2.45 (m, 1H), 2.38 – 2.28 (m, 2H), 2.16 – 2.02 (m, 2H), 2.01 – 1.90 (m, 1H), 1.79 – 1.68 (m, 2H), 1.65 – 1.50 (m, 4H), 1.48 (d, *J* = 7.2 Hz, 3H), 1.46 – 1.30 (m, 2H). **^{13}C NMR** (101 MHz, Chloroform-*d*) δ 220.26, 174.73, 139.00, 138.49, 129.22, 127.61, 96.63, 90.29 (d, *J* = 174.4 Hz), 64.32, 59.56 (d, *J* = 22.5 Hz), 51.08, 45.27, 38.29, 35.32, 35.03 (d, *J* = 21.3 Hz), 29.36, 28.24, 21.21 (d, *J* = 4.5 Hz), 20.66, 18.56. **^{19}F NMR** (376 MHz, Chloroform-*d*) δ -178.32 – -178.36 (m, 1F). **ESI-HRMS** exact mass calculated for [$C_{22}H_{28}Cl_3FO_3Na^+$]: 487.0980, found 487.0988.

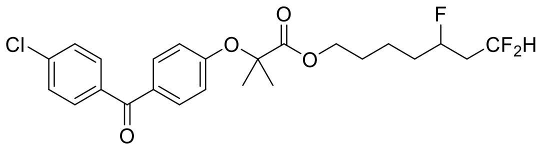

72

5,7,7-Trifluoroheptyl 2-(4-(4-chlorobenzoyl)phenoxy)-2-methylpropanoate (**72**): The compound was purified by columnchromatography (petroleum ether/EtOAc = 60/1), resulting in a colorless liquid (28.1 mg) at 30% yield. **^{1}H NMR** (400 MHz, DMSO-d_6) δ 7.77 – 7.58 (m, 6H), 6.90 (d, *J* = 8.7 Hz, 2H), 6.18 – 6.06 (m, 1H), 4.68 – 4.54 (m, 1H), 4.13 (t, *J* = 6.3 Hz, 2H), 2.21 – 1.98 (m, 2H), 1.65 – 1.44 (m, 10H), 1.37 – 1.17 (m, 2H). **^{13}C NMR** (101 MHz, Chloroform-*d*) δ 194.29, 173.87, 159.78, 138.53, 136.46, 132.16, 131.28, 130.49, 128.67, 117.21, 115.08 (dt, *J* = 4.9, 238.7 Hz), 89.86 – 87.96 (m), 79.52, 65.44, 40.35 – 39.60 (m), 34.68 (d, *J* = 20.4 Hz), 28.18, 25.56 (d, *J* = 4.3 Hz), 21.36 (d, *J* = 4.3 Hz). **^{19}F NMR** (376 MHz, Chloroform-*d*) δ -115.96 – -118.38 (m, 2F), -183.71 (s, 1F). **ESI-HRMS** exact mass calculated for [$C_{24}H_{26}ClF_3O_4H^+$]: 471.1544, found 471.1542.

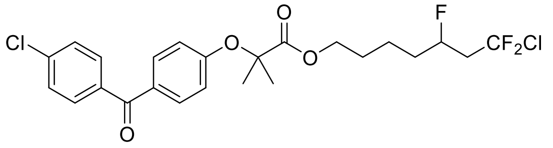

73

7-Chloro-5,7,7-trifluoroheptyl 2-(4-(4-chlorobenzoyl)phenoxy)-2-methylpropanoate (**73**): The compound was purified by columnchromatography (petroleum ether/EtOAc = 60/1), resulting in a colorless liquid (41.0 mg) at 41% yield. **^{1}H NMR** (400 MHz, Chloroform-*d*) δ 7.77 – 7.65 (m, 4H), 7.47 – 7.40 (m, 2H), 6.89 – 6.81 (m, 2H), 4.80 – 4.68 (m, 1H), 4.17 (t, *J* = 6.4 Hz, 2H), 2.71-2.60 (m, 1H), 2.53 – 2.30 (m, 1H), 1.67 (s, 6H), 1.68 – 1.26 (m, 6H). **^{13}C NMR** (101 MHz, Chloroform-*d*) δ 194.26, 173.84, 159.76, 138.51, 136.42, 132.13, 131.26, 130.48, 128.65, 127.72 (dt, *J* = 3.0, 292.5 Hz), 117.19, 88.17 (dt, *J* = 173.3, 3.0 Hz), 79.50, 65.35, 47.70 – 46.50 (m), 34.56 (d, *J* = 20.9 Hz), 28.12, 25.54 (d, *J* = 4.8 Hz), 21.22 (d, *J* = 4.1 Hz). **^{19}F NMR** (376 MHz, Chloroform-*d*) δ -48.63 – -50.33 (m, 2F), -181.92 – -182.00 (m, 1F). **ESI-HRMS** exact mass calculated for [$C_{24}H_{25}Cl_2F_3O_4H^+$]: 505.1155, found 505.1153.

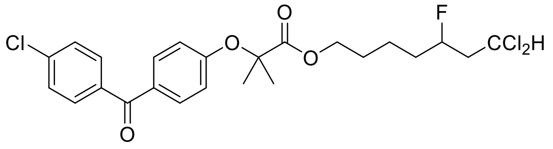

74

7,7-Dichloro-5-fluoroheptyl 2-(4-(4-chlorobenzoyl)phenoxy)-2-methylpropanoate (**74**): The compound was purified by columnchromatography (petroleum ether/EtOAc = 60/1), resulting in a colorless liquid (50.8 mg) at 51% yield. **^{1}H NMR** (400 MHz, DMSO-*d*$_6$) δ 7.77 – 7.58 (m, 6H), 6.90 (d, *J* = 8.8 Hz, 2H), 6.33 – 6.21 (m, 1H), 4.67 – 4.45 (m, 1H), 4.13 (t, *J* = 6.2 Hz, 2H), 2.47 – 2.27 (m, 2H), 1.54 (m, 10H), 1.26 (m, 2H). **^{13}C NMR** (101 MHz, DMSO-*d*$_6$) δ 193.18, 172.77, 159.23, 137.07, 136.23, 131.93, 131.18, 129.57, 128.61, 117.05, 90.68 (d, *J* = 168.6 Hz), 79.11, 71.05 (d, *J* = 4.4 Hz), 65.04, 47.80 (d, *J* = 20.6 Hz), 33.26 (d, *J* = 19.8 Hz), 27.56, 25.11, 20.70 (d, *J* = 4.4 Hz). **^{19}F NMR** (376 MHz, Chloroform-*d*) δ -178.15 (s, 1F). **ESI-HRMS** exact mass calculated for [$C_{24}H_{26}Cl_3FO_4H^+$]: 503.0953, found 503.0953.

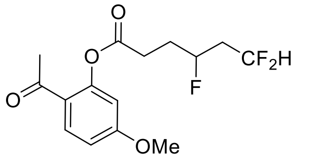

75

2-Acetyl-5-methoxyphenyl 4,6,6-trifluorohexanoate (**75**): The compound was purified by columnchromatography (petroleum ether/EtOAc = 80/1), resulting in a colorless liquid (19.8 mg) at 31% yield. **^{1}H NMR** (400 MHz, Chloroform-*d*) δ 7.83 (d, *J* = 8.8 Hz, 1H), 6.86 – 6.80 (m, 1H), 6.60 (d, *J* = 2.5 Hz, 1H), 6.07 – 5.96 (m, 1H), 4.99 – 4.79 (m, 1H), 3.86 (s, 3H), 2.90 – 2.75 (m, 2H), 2.50 (s, 3H), 2.36 – 2.07 (m, 4H). **^{13}C NMR** (101 MHz, Chloroform-*d*) δ 195.85, 171.37, 163.97, 151.39, 132.84, 122.78, 115.02 (dt, *J* = 5.0, 238.9 Hz), 111.82, 109.53, 89.31 – 87.42 (m), 55.89, 40.45 – 39.75 (m), 30.14 (d, *J* = 20.7 Hz), 29.78 (d, *J* = 4.4 Hz), 28.90. **^{19}F NMR** (376 MHz, Chloroform-*d*) δ -115.89 – -118.23 (m, 2F), -185.72 – -185.75 (m, 1F). **ESI-HRMS** exact mass calculated for [$C_{15}H_{17}F_3O_4Na^+$]: 341.0971, found 341.0988.

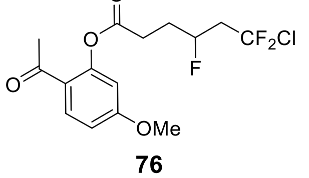

76

2-Acetyl-5-methoxyphenyl 6-chloro-4,6,6-trifluorohexanoate (**76**): The compound was purified by columnchromatography (petroleum ether/EtOAc = 80/1), resulting in a colorless liquid (36.2 mg) at 51% yield. **^{1}H NMR** (400 MHz, DMSO-d_6) δ 7.93 (d, *J* = 8.8 Hz, 1H), 6.98 – 6.93 (m, 1H), 6.78 (d, *J* = 2.4 Hz, 1H), 5.11 – 4.91 (m, 1H), 3.82 (s, 3H), 3.05 – 2.87 (m, 2H), 2.76 (t, *J* = 7.5 Hz, 2H), 2.45 (s, 3H), 2.13 – 1.96 (m, 2H). **^{13}C NMR** (101 MHz, Chloroform-*d*) δ 195.82, 171.26, 163.97, 151.36, 132.86, 127.67 (dt, *J* = 2.6, 293.4 Hz), 122.69, 111.82, 109.52, 87.62 (dt, *J* = 173.5, 3.1 Hz), 55.87, 47.09 (td, *J* = 24.3, 22.2 Hz), 30.06 (d, *J* = 20.9 Hz), 29.61 (d, *J* = 4.2 Hz), 28.85. **^{19}F NMR** (376 MHz, Chloroform-*d*) δ -48.56 – -50.43 (m, 2F), -183.95 – -184.03 (m, 1F). **ESI-HRMS** exact mass calculated for [$C_{15}H_{16}ClF_3O_4Na^+$]: 375.0581, found 375.0587.

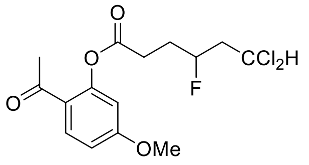

77

2-Acetyl-5-methoxyphenyl 6,6-dichloro-4-fluorohexanoate (**77**): The compound was purified by columnchromatography (petroleum ether/EtOAc = 80/1), resulting in a colorless liquid (30.1 mg) at 43% yield. **^{1}H NMR** (400 MHz, Chloroform-*d*) δ 7.83 (d, *J* = 8.8 Hz, 1H), 6.87 – 6.79 (m, 1H), 6.60 (d, *J* = 2.5 Hz, 1H), 5.96 – 5.87 (m, 1H), 5.00 – 4.79 (m, 1H), 3.85 (s, 3H), 2.90 – 2.75 (m, 2H), 2.70 – 2.57 (m, 1H), 2.50 (s, 3H), 2.49 – 2.35 (m, 1H), 2.25 – 2.05 (m, 2H). **^{13}C NMR** (101 MHz, Chloroform-*d*) δ 195.80, 171.32, 163.94, 151.37, 132.83, 122.73, 111.78, 109.52, 89.91 (d, *J* = 170.4 Hz), 69.46 (d, *J* = 4.5 Hz), 55.87, 49.22 (d, *J* = 20.5 Hz), 29.79, 29.71 (d, *J* = 25.5 Hz), 28.88. **^{19}F NMR** (376 MHz, Chloroform-*d*) δ -187.26 (s, 1F). **ESI-HRMS** exact mass calculated for [$C_{15}H_{17}Cl_2FO_4Na^+$]: 373.0380, found 373.0384.

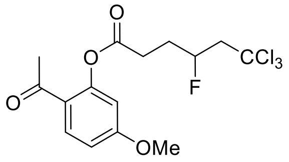

78

2-Acetyl-5-methoxyphenyl 6,6,6-trichloro-4-fluorohexanoate (**78**): The compound was purified by columnchromatography (petroleum ether/EtOAc = 80/1), resulting in a colorless liquid (40.8 mg) at 53% yield. **^{1}H NMR** (400 MHz, Chloroform-*d*) δ 7.83 (d, *J* = 8.8 Hz, 1H), 6.88 – 6.78 (m, 1H), 6.61 (d, *J* = 2.5 Hz, 1H), 5.22 – 5.02 (m, 1H), 3.85 (s, 3H), 3.29 – 3.18 (m, 1H), 3.05 – 2.96 (m, 1H), 2.92 – 2.79 (m, 2H), 2.50 (s, 3H), 2.31 – 2.14 (m, 2H). **^{13}C NMR** (101 MHz, Chloroform-*d*) δ 195.79, 171.27, 163.96, 151.37, 132.84, 122.71, 111.81, 109.52, 96.35, 89.64 (d, *J* = 174.7 Hz), 59.54 (d, *J* = 22.2 Hz), 55.87, 30.46 (d, *J* = 21.4 Hz), 29.70 (d, *J* = 4.4 Hz), 28.88. **^{19}F NMR** (376 MHz, Chloroform-*d*) δ -180.74 (s, 1F). **ESI-HRMS** exact mass calculated for [$C_{15}H_{16}Cl_3FO_4Na^+$]: 406.9990, found 407.0002.

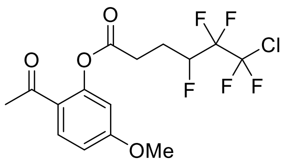

79

2-Acetyl-5-methoxyphenyl 7-chloro-4,6,6,7,7-pentafluoroheptanoate (**79**): The compound was purified by columnchromatography (petroleum ether/EtOAc = 80/1), resulting in a colorless liquid (33.7 mg) at 42% yield. **^{1}H NMR** (400 MHz, Chloroform-*d*) δ 7.83 (d, *J* = 8.8 Hz, 1H), 6.88 – 6.78 (m, 1H), 6.61 (d, *J* = 2.5 Hz, 1H), 5.20 – 5.00 (m, 1H), 3.86 (s, 3H), 2.94 – 2.78 (m, 2H), 2.69 – 2.53 (m, 1H), 2.50 (s, 3H), 2.47 – 2.28 (m, 1H), 2.27 – 2.11 (m, 2H). **^{13}C NMR** (101 MHz, Chloroform-*d*) δ 195.80, 171.24, 163.97, 151.36, 132.85, 123.45 (tt, *J* = 298.9, 37.1 Hz), 122.68, 118.99 – 113.10 (m), 111.81, 109.51, 86.40 (dt, *J* = 173.6, 2.7 Hz), 55.86, 36.70 – 35.95 (m), 30.54 (d, *J* = 20.9 Hz), 29.65 (d, *J* = 4.3 Hz), 28.82. **^{19}F NMR** (376 MHz, Chloroform-*d*) δ -71.68 – -71.72

(m, 2F), -112.80 – -113.10 (m, 2F), -182.64 – -182.76 (m, 1F). **ESI-HRMS** exact mass calculated for [$C_{16}H_{16}ClF_5O_4Na^+$]: 425.0549, found 425.0551.

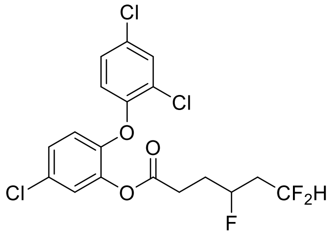

80

5-Chloro-2-(2,4-dichlorophenoxy)phenyl 4,6,6-trifluorohexanoate (**80**): The compound was purified by columnchromatography (petroleum ether/EtOAc = 80/1), resulting in a colorless liquid (28.5 mg) at 32% yield. **^{1}H NMR** (400 MHz, DMSO-d_6) δ 7.79 – 7.73 (m, 1H), 7.54 – 7.48 (m, 1H), 7.44 – 7.34 (m, 2H), 7.12 (d, *J* = 8.8 Hz, 1H), 6.97 (d, *J* = 8.8 Hz, 1H), 6.21 – 6.11 (m, 1H), 4.89 – 4.67 (m, 1H), 2.64 (t, *J* = 7.5 Hz, 2H), 2.32 – 2.06 (m, 2H), 2.02 – 1.83 (m, 2H). **^{13}C NMR** (101 MHz, DMSO-d_6) δ 170.14, 150.65, 146.20, 141.46, 130.08, 128.80, 128.49, 128.27, 127.38, 124.62, 124.55, 121.19, 120.45, 115.78 (dt, *J* = 4.5, 237.3 Hz), 88.14 (dt, *J* = 167.5, 6.8 Hz), 38.94 – 38.24 (m), 29.49 (d, *J* = 20.5 Hz), 28.65 (d, *J* = 4.6 Hz). **^{19}F NMR** (376 MHz, DMSO-d_6) δ -115.67 – -115.73 (m, 2F), -183.53 (t, *J* = 3.5 Hz, 1F). **ESI-HRMS** exact mass calculated for [$C_{18}H_{14}Cl_3F_3O_3Na^+$]: 462.9853, found 462.9852.

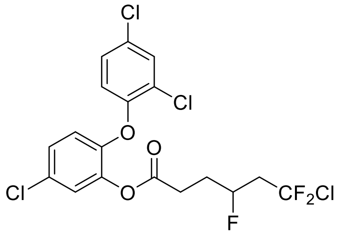

81

5-Chloro-2-(2,4-dichlorophenoxy)phenyl 6-chloro-4,6,6-trifluorohexanoate (**81**): The compound was purified by columnchromatography (petroleum ether/EtOAc = 80/1), resulting in a colorless liquid (55.8 mg) at 59% yield. **^{1}H NMR** (400 MHz, DMSO-d_6) δ 7.76 – 7.70 (m, 1H), 7.52 – 7.46 (m, 1H), 7.43 – 7.32 (m, 2H), 7.08 (d, *J* = 8.8 Hz, 1H), 6.96 (d, *J* = 8.8 Hz, 1H), 5.01 – 4.76 (m, 1H), 2.98 – 2.78 (m, 2H), 2.66 (t, *J* = 7.4 Hz, 2H), 2.06 – 1.83 (m, 2H). **^{13}C NMR** (101 MHz, DMSO-d_6) δ 170.27, 150.72, 146.39, 141.54, 131.49 – 125.60 (m), 130.21, 128.92, 128.71, 128.39, 127.53, 124.85, 124.63, 121.16, 120.67, 87.79 (dt, *J* = 171.6, 3.3 Hz), 45.38 (td, *J* = 23.2, 21.0 Hz), 29.26 (d, *J* = 20.6 Hz), 28.58 (d, *J* = 4.6 Hz). **^{19}F NMR** (376 MHz, DMSO-d_6) δ -46.48 – -48.87 (m, 2F), -183.61 (t, *J* = 9.9 Hz, 1F). **ESI-HRMS** exact mass calculated for [$C_{18}H_{13}Cl_4F_3O_3H^+$]: 474.9644, found 474.9652.

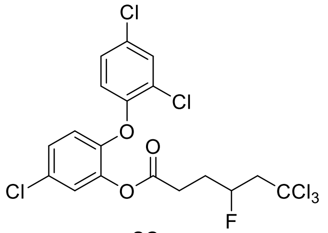

82

5-Chloro-2-(2,4-dichlorophenoxy)phenyl 6,6,6-trichloro-4-fluorohexanoate (**82**): The compound was purified by columnchromatography (petroleum ether/EtOAc = 80/1), resulting in a colorless liquid (50.3 mg) at 50% yield. **^{1}H NMR** (400 MHz, Chloroform-*d*) δ 7.49 – 7.43 (m, 1H), 7.23 – 7.14 (m, 3H), 6.92 – 6.80 (m, 2H), 5.11 – 4.92 (m, 1H), 3.23 – 3.13 (m, 1H), 2.97 – 2.86 (m, 1H), 2.73 (t, *J* = 7.1 Hz, 2H), 2.14 – 2.00 (m, 2H). **^{13}C NMR** (101 MHz, Chloroform-*d*) δ 170.10, 150.92, 146.67, 141.59, 130.56, 129.74, 129.36, 128.31, 127.25, 126.02, 124.41, 120.51, 120.17, 96.12, 89.30 (d, *J* = 175.3 Hz), 59.41 (d, *J* = 22.4 Hz), 30.52 (d, *J* = 21.4 Hz), 29.18 (d, *J* = 4.4 Hz).

^{19}**F NMR** (376 MHz, Chloroform-*d*) δ -180.68 (s, 1F). **ESI-HRMS** exact mass calculated for [$C_{18}H_{13}Cl_6FO_3Na^+$]: 528.8872, found 528.8871.

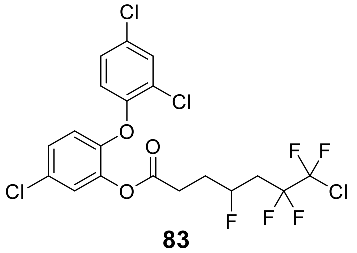

83

5-Chloro-2-(2,4-dichlorophenoxy)phenyl 7-chloro-4,6,6,7,7-pentafluoroheptanoate (**83**): The compound was purified by columnchromatography (petroleum ether/EtOAc = 80/1), resulting in a colorless liquid (68.5 mg) at 65% yield. ^{1}**H NMR** (400 MHz, DMSO-*d*$_6$) δ 7.77 – 7.71 (m, 1H), 7.53 – 7.47 (m, 1H), 7.44 – 7.33 (m, 2H), 7.09 (d, *J* = 8.8 Hz, 1H), 6.98 (d, *J* = 8.8 Hz, 1H), 5.09 – 4.86 (m, 1H), 2.79 – 2.53 (m, 4H), 2.13 – 1.88 (m, 2H). ^{13}**C NMR** (101 MHz, DMSO-*d*$_6$) δ 170.11, 150.61, 146.28, 141.44, 130.06, 128.76, 128.56, 128.24, 127.35, 126.50 – 119.66 (m), 124.73, 124.51, 121.02, 120.54, 119.35 – 113.48 (m), 87.46 – 85.55 (m), 34.69 (q, *J* = 21.0 Hz), 29.56 (d, *J* = 20.4 Hz), 28.54 (d, *J* = 4.4 Hz). ^{19}**F NMR** (376 MHz, DMSO-*d*$_6$) δ -71.28 – -71.34 (m, 2F), -110.53 – -113.46 (m, 2F), -182.25 – -182.40 (m, 1F). **ESI-HRMS** exact mass calculated for [$C_{19}H_{13}Cl_4F_5O_3H^+$]: 524.9612, found 524.9628.

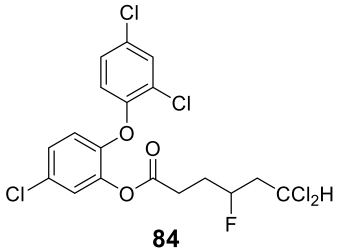

84

5-Chloro-2-(2,4-dichlorophenoxy)phenyl 6,6-dichloro-4-fluorohexanoate (**84**): The compound was purified by columnchromatography (petroleum ether/EtOAc = 80/1), resulting in a colorless liquid (36.8 mg) at 39% yield. ^{1}**H NMR** (400 MHz, DMSO-*d*$_6$) δ 7.79 – 7.73 (m, 1H), 7.53 – 7.47 (m, 1H), 7.42.7.44 (m, 2H), 7.10 (d, *J* = 8.8 Hz, 1H), 6.98 (d, *J* = 8.8 Hz, 1H), 6.33 – 6.27 (m, 1H), 4.86 – 4.63 (m, 1H), 2.65 (t, *J* = 7.4 Hz, 2H), 2.61 – 2.39 (m, 2H), 2.04 – 1.82 (m, 2H). ^{13}**C NMR** (101 MHz, DMSO-*d*$_6$) δ 170.13, 150.62, 146.27, 141.41, 130.07, 128.80, 128.53, 128.21, 127.36, 124.72, 124.49, 121.06, 120.57, 89.78 (d, *J* = 169.2 Hz), 70.87 (d, *J* = 4.4 Hz), 47.56 (d, *J* = 20.2 Hz), 29.04 (d, *J* = 20.4 Hz), 28.63 (d, *J* = 4.6 Hz). ^{19}**F NMR** (376 MHz, DMSO-*d*$_6$) δ -184.75 (s, 1F). **ESI-HRMS** exact mass calculated for [$C_{18}H_{14}Cl_5FO_3Na^+$]: 494.9262, found 494.9261.

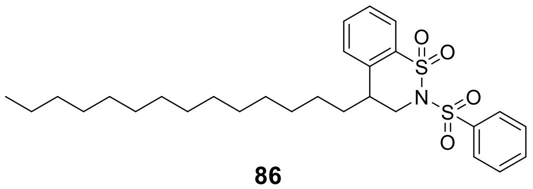

86

2-**(phenylsulfonyl)-4-Tetradecyl-3,4-dihydro-2H-benzo[e][1,2]thiazine 1,1-dioxide** (**86**): The compound was purified by columnchromatography (petroleum ether/EtOAc = 60/1), resulting in a colorless liquid (27.0 mg) at 26% yield. ^{1}**H NMR** (400 MHz, Chloroform-*d*) δ 7.99 – 7.94 (m, 2H), 7.75 – 7.70 (m, 1H), 7.63 – 7.57 (m, 1H), 7.52 – 7.46 (m, 3H), 7.37 – 7.31 (m, 1H), 7.31 – 7.27 (m, 1H), 4.52 – 4.28 (m, 2H), 3.24 – 3.15 (m, 1H), 1.79 – 1.70 (m, 2H), 1.49 – 1.36 (m, 2H), 1.30 – 1.21 (m, 22H), 0.88 (t, *J* = 6.7 Hz, 3H). ^{13}**C NMR** (101 MHz, Chloroform-*d*) δ 139.26, 139.16, 137.21, 134.02, 132.94, 129.05, 129.00, 128.22, 127.32, 123.74, 50.02, 37.17, 34.24, 31.97, 29.75, 29.74, 29.71, 29.70, 29.68, 29.63, 29.62, 29.48, 29.41, 26.51, 22.74, 14.18. **ESI-HRMS** exact mass calculated for [$C_{28}H_{41}NO_4S_2Na^+$]: 542.2369; found: 542.2381.

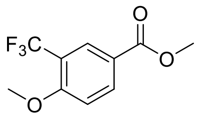

103

3-Methyl 4-methoxy-3-(trifluoromethyl)benzoate (**103**): The compound was purified by columnchromatography (petroleum ether/EtOAc = 80/1), resulting in a solid with color (10.3 mg) at 22% yield. **^{1}H NMR** (400 MHz, Chloroform-*d*) δ 8.2 – 8.25 (m, 1H), 8.21 – 8.17 (m, 1H), 7.03 (d, *J* = 8.7 Hz, 1H), 3.97 (s, 3H), 3.91 (s, 3H). **^{13}C NMR** (101 MHz, Chloroform-*d*) δ 165.92, 161.12 – 161.02 (m), 135.28, 129.16 (q, *J* = 5.3 Hz), 123.25 (q, *J* = 272.5 Hz), 122.29, 118.89 (q, *J* = 31.6 Hz), 111.66, 56.35, 52.34. **^{19}F NMR** (376 MHz, Chloroform-*d*) δ -62.90 (s, 3F). **ESI-HRMS** exact mass calculated for [$C_{10}H_9F_3O_3H^+$]: 235.0577; found: 235.0577.

Copies of ^{1}H NMR, ^{13}C NMR and ^{19}F NMR spectra

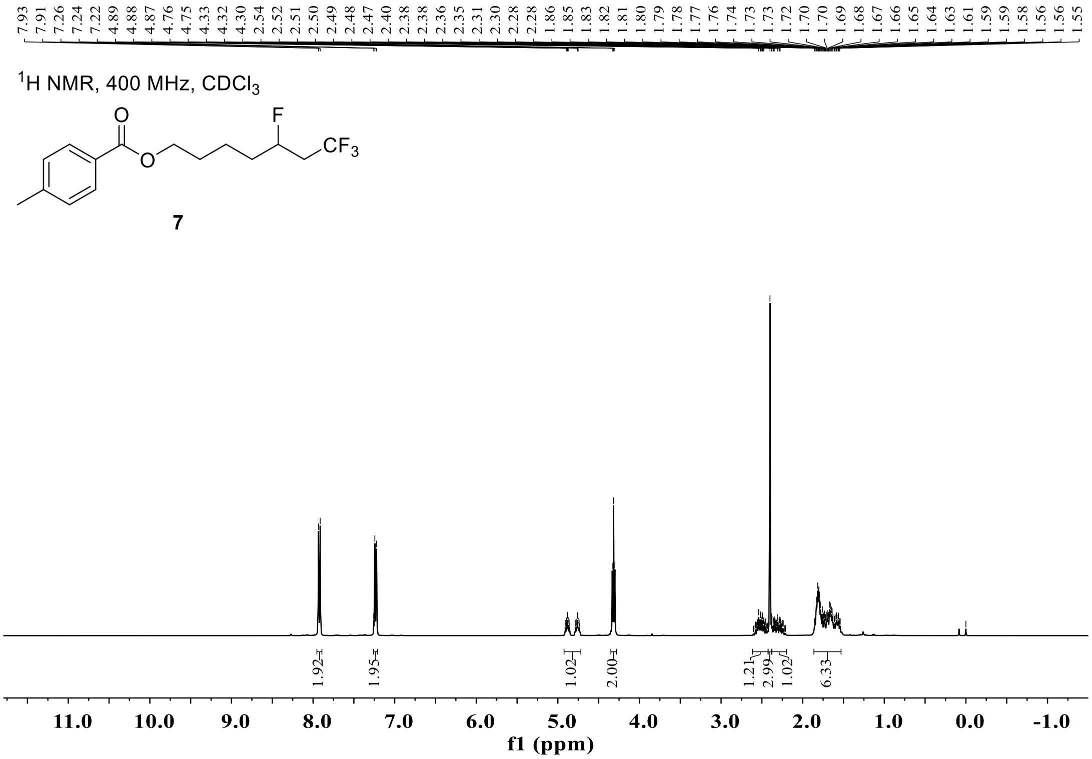

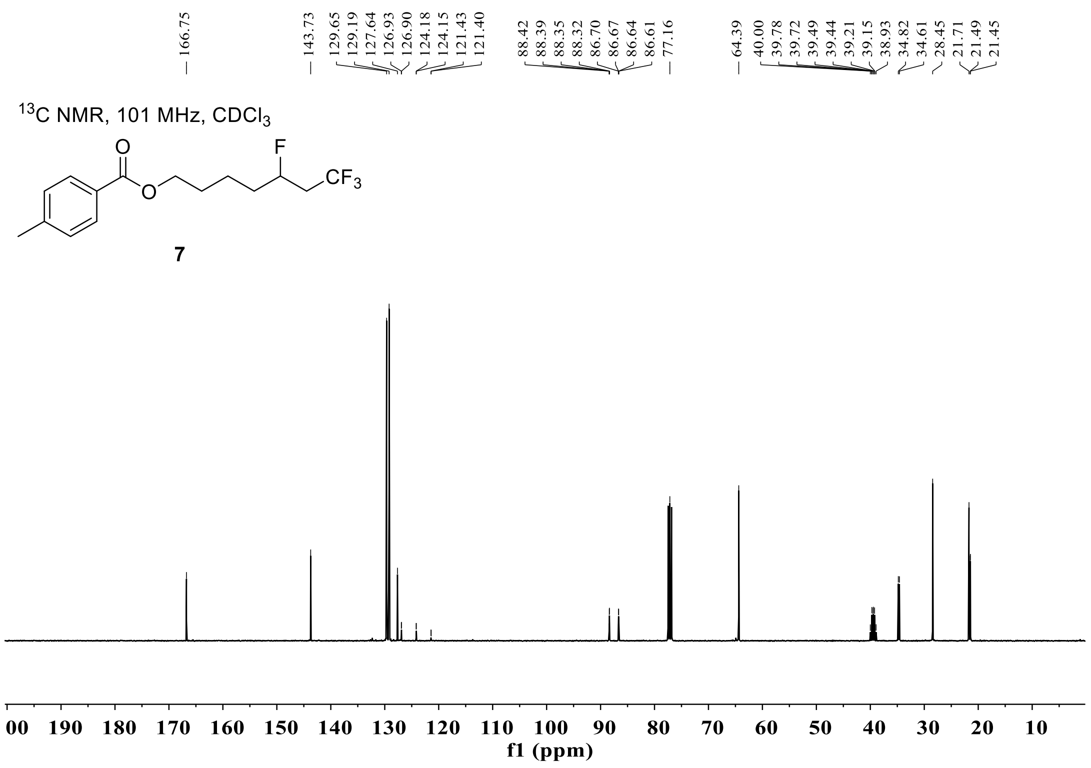

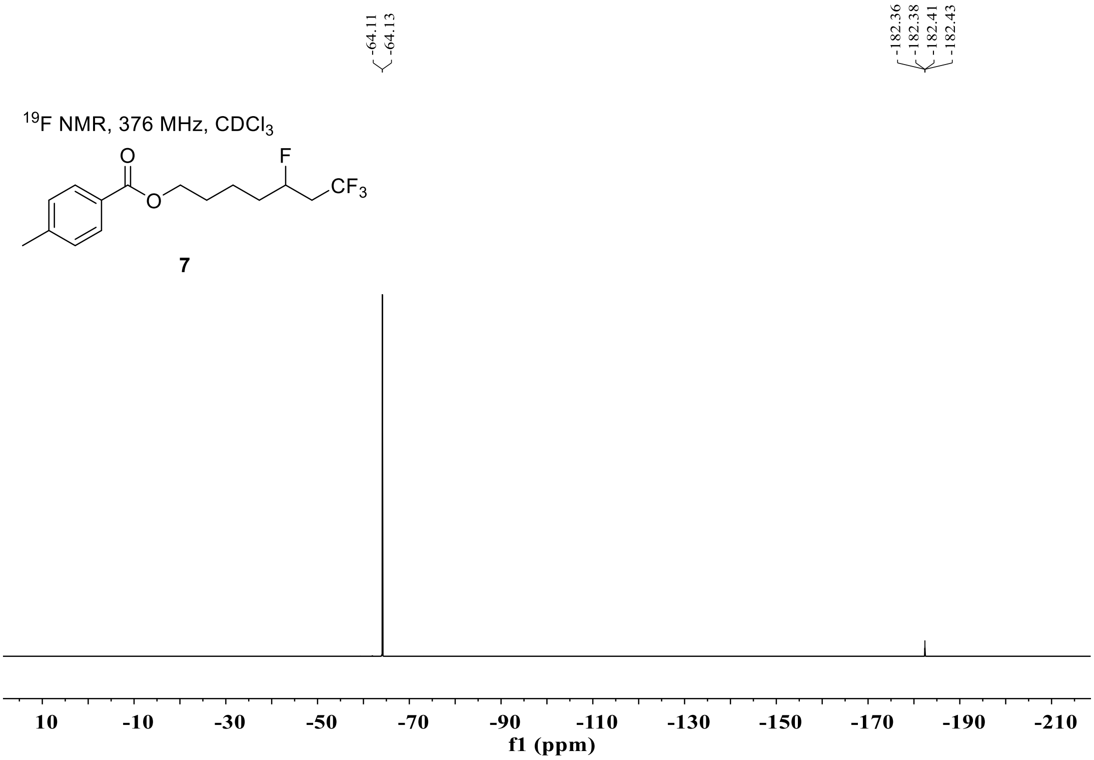

-64.11
-64.13
-182.36
-182.38
-182.41
-182.43
^{19}F NMR, 376 MHz, CDCl$_3$
O
O
F
CF$_3$
7
10 -10 -30 -50 -70 -90 -110 -130 -150 -170 -190 -210
f1 (ppm)

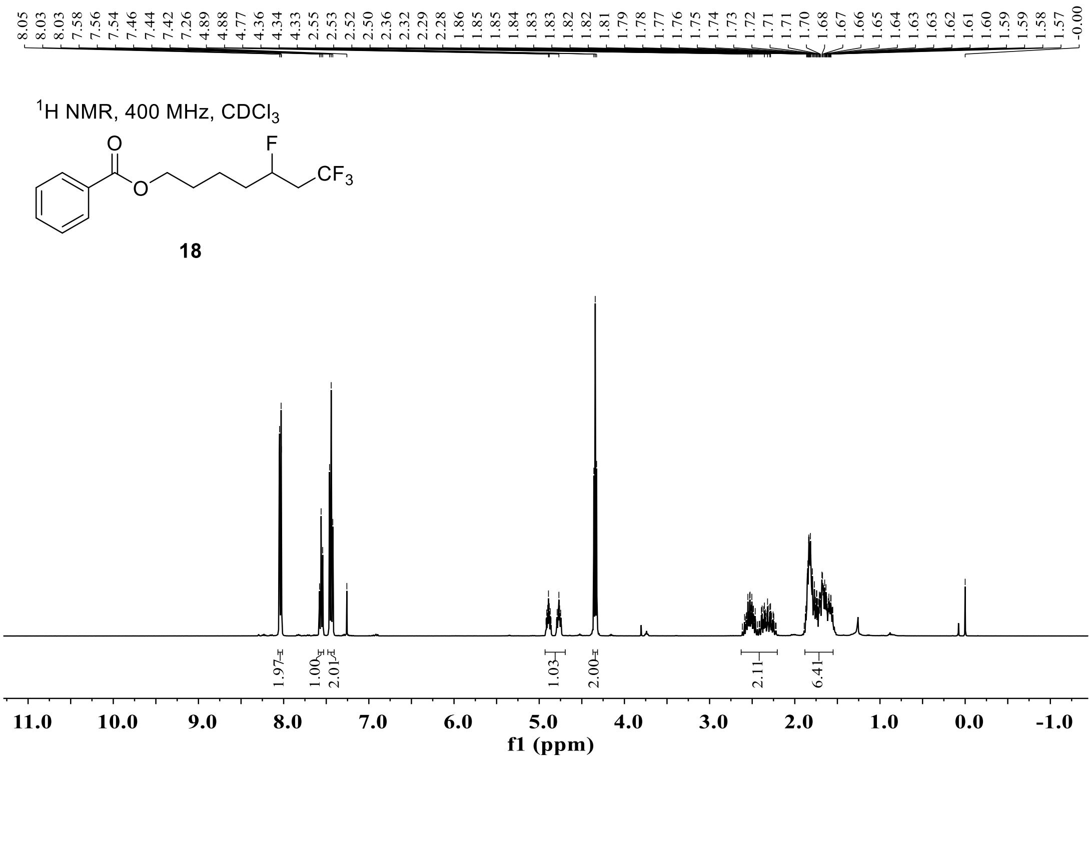

8.05 8.03 8.03 7.58 7.56 7.54 7.46 7.44 7.42 7.26 4.89 4.88 4.77 4.36 4.34 4.33 2.55 2.53 2.52 2.50 2.36 2.32 2.29 2.28 1.86 1.85 1.85 1.84 1.83 1.83 1.82 1.82 1.81 1.79 1.78 1.77 1.76 1.75 1.74 1.73 1.72 1.71 1.71 1.70 1.68 1.67 1.66 1.65 1.64 1.63 1.63 1.62 1.61 1.60 1.59 1.59 1.58 1.57 -0.00
^{1}H NMR, 400 MHz, CDCl$_3$
O
O
F
CF$_3$
18
1.97
1.00
2.01
1.03
2.00
2.11
6.41
11.0 10.0 9.0 8.0 7.0 6.0 5.0 4.0 3.0 2.0 1.0 0.0 -1.0
f1 (ppm)

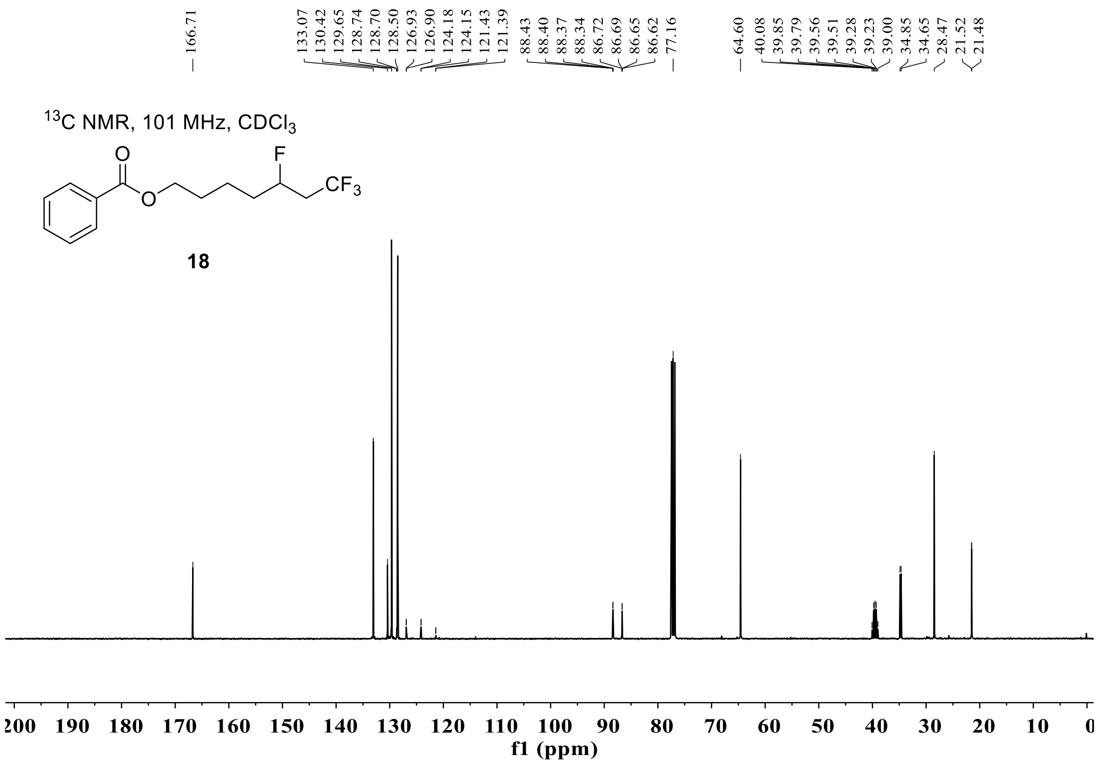
166.71
133.07
130.42
129.65
128.74
128.70
128.50
126.93
126.90
124.18
124.15
121.43
121.39
88.43
88.40
88.37
88.34
86.72
86.69
86.65
86.62
77.16
64.60
40.08
39.85
39.79
39.56
39.51
39.28
39.23
39.00
34.85
34.65
28.47
21.52
21.48
^{13}C NMR, 101 MHz, CDCl$_3$
O
F
O
CF$_3$
18
200 190 180 170 160 150 140 130 120 110 100 90 80 70 60 50 40 30 20 10 0
f1 (ppm)

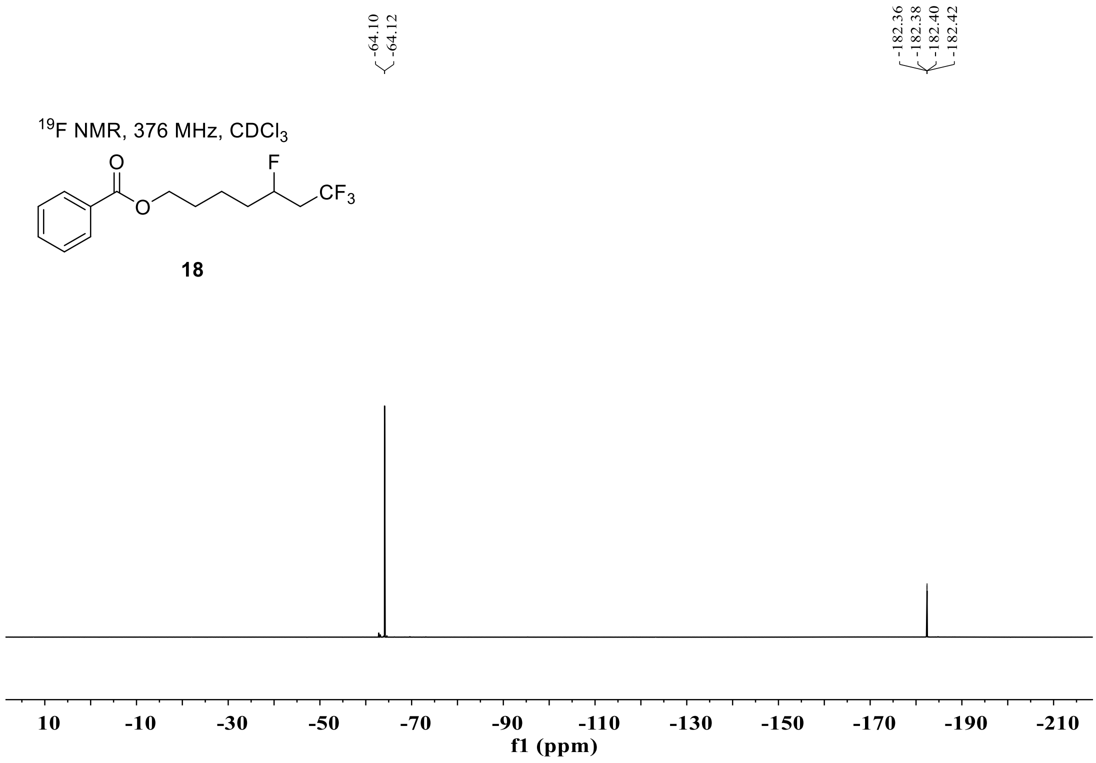
-64.10
-64.12
-182.36
-182.38
-182.40
-182.42
^{19}F NMR, 376 MHz, CDCl$_3$
O
F
O
CF$_3$
18
10 -10 -30 -50 -70 -90 -110 -130 -150 -170 -190 -210
f1 (ppm)

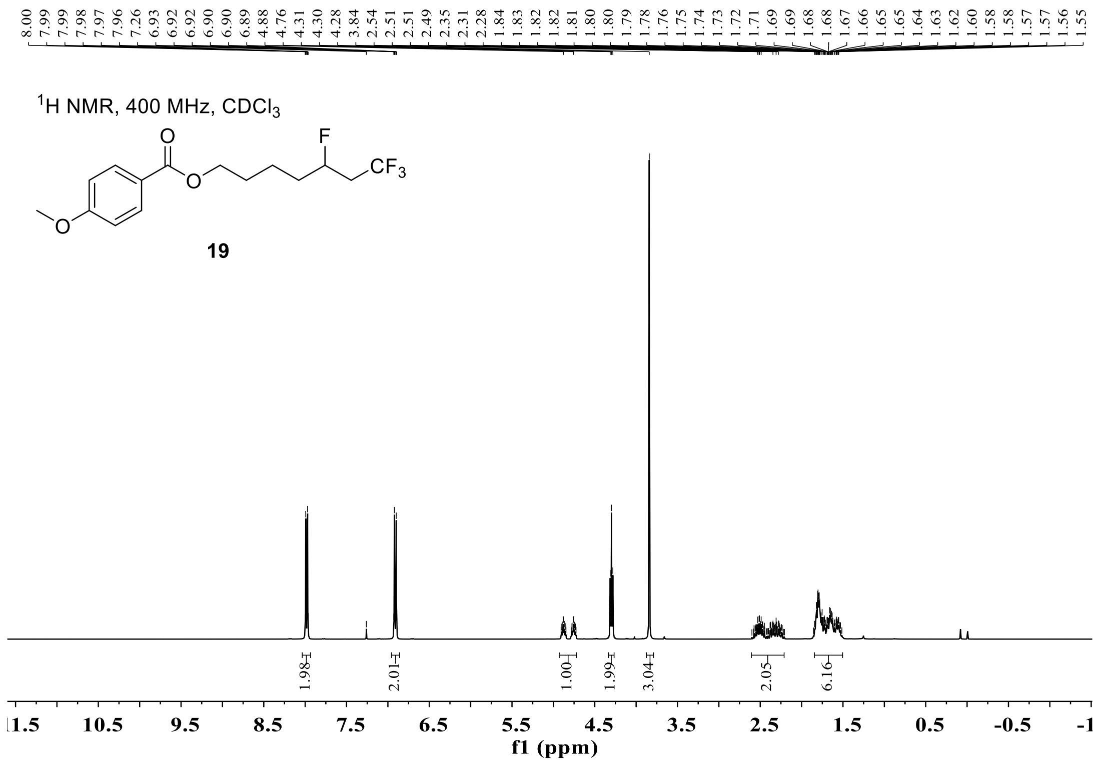

8.00
7.99
7.99
7.98
7.97
7.96
7.26
6.93
6.92
6.92
6.90
6.90
6.89
4.88
4.76
4.31
4.30
4.28
3.84
2.54
2.51
2.51
2.49
2.35
2.31
2.28
1.84
1.83
1.82
1.82
1.81
1.80
1.80
1.79
1.78
1.76
1.75
1.74
1.73
1.72
1.71
1.69
1.69
1.68
1.68
1.67
1.66
1.65
1.65
1.64
1.63
1.62
1.60
1.58
1.57
1.57
1.56
1.55
^{1}H NMR, 400 MHz, CDCl$_3$
O
F
O
CF$_3$
O
19
1.98
2.01
1.00
1.99
3.04
2.05
6.16
11.5
10.5
9.5
8.5
7.5
6.5
5.5
4.5
3.5
2.5
1.5
0.5
-0.5
-1
f1 (ppm)

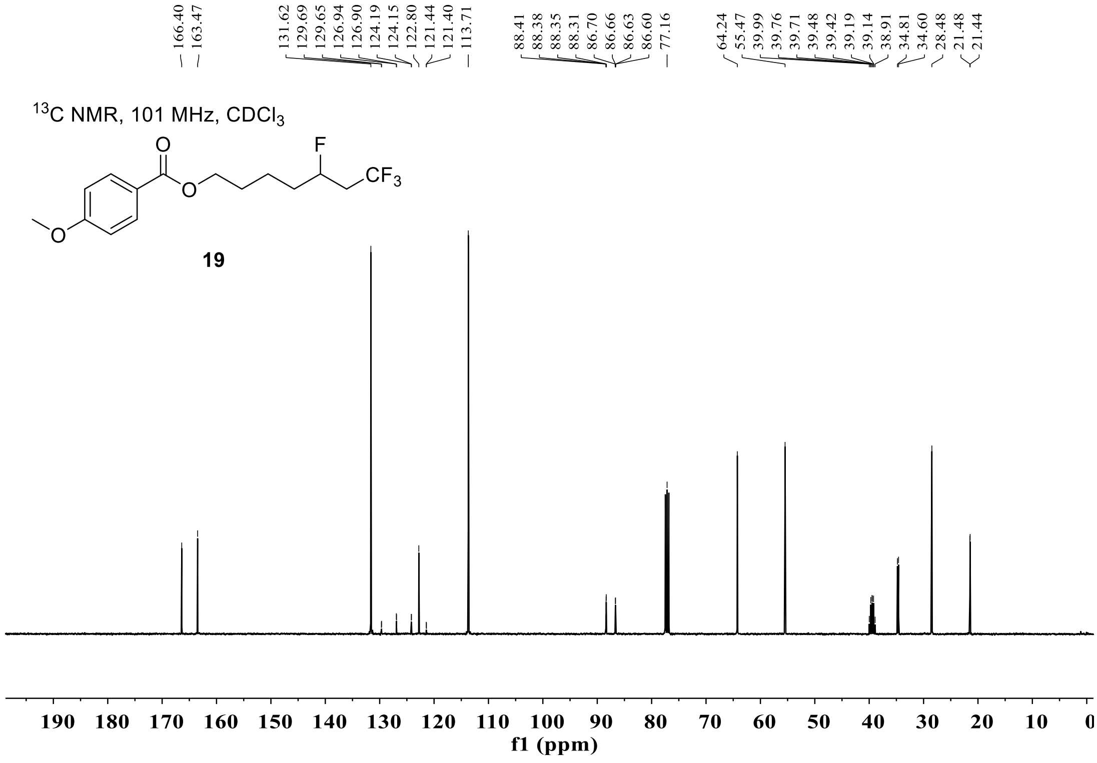

166.40
163.47
131.62
129.69
129.65
126.94
126.90
124.19
124.15
122.80
121.44
121.40
113.71
88.41
88.38
88.35
88.31
86.70
86.66
86.63
86.60
77.16
64.24
55.47
39.99
39.76
39.71
39.48
39.42
39.19
39.14
38.91
34.81
34.60
28.48
21.48
21.44
^{13}C NMR, 101 MHz, CDCl$_3$
O
F
O
CF$_3$
O
19
190
180
170
160
150
140
130
120
110
100
90
80
70
60
50
40
30
20
10
0
f1 (ppm)

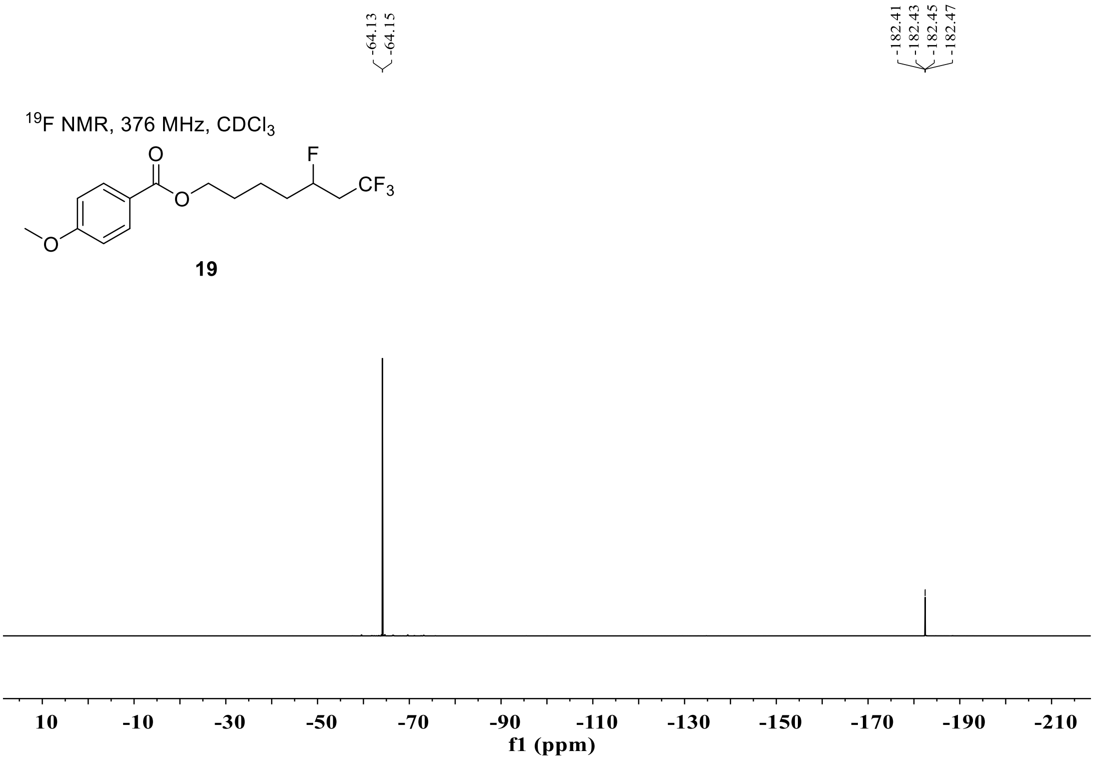
-64.13
-64.15
-182.41
-182.43
-182.45
-182.47
19F NMR, 376 MHz, CDCl3
19
10 -10 -30 -50 -70 -90 -110 -130 -150 -170 -190 -210
f1 (ppm)

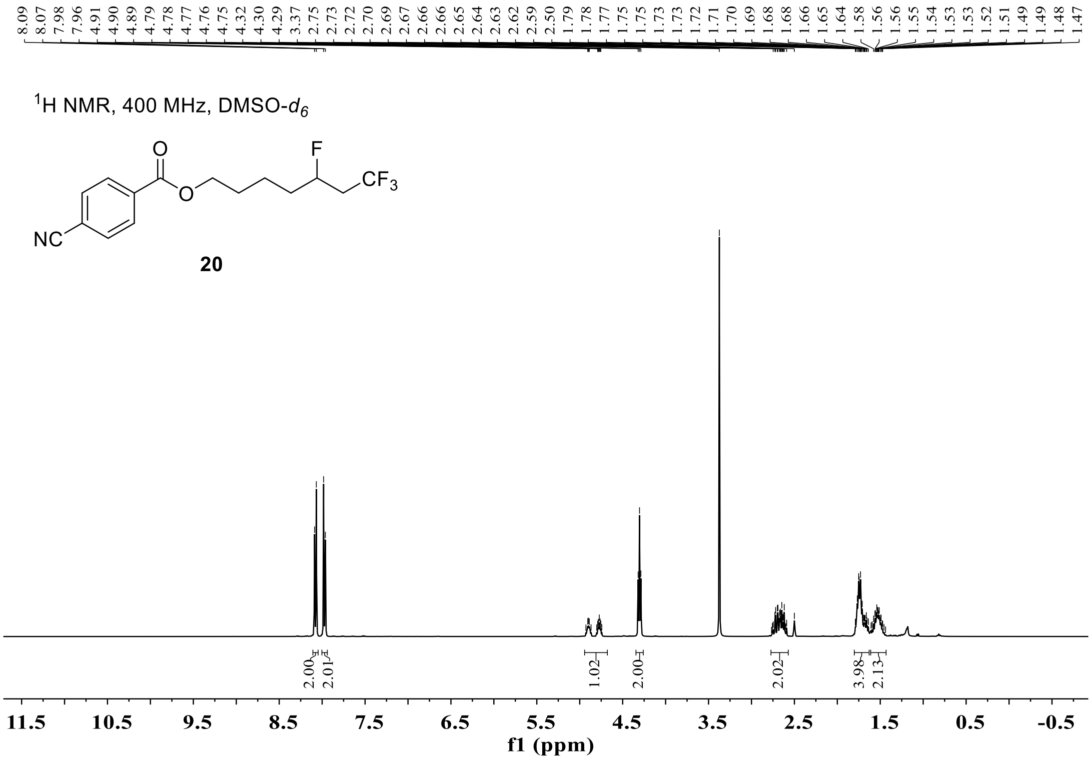
8.09 8.07 7.98 7.96 4.91 4.90 4.89 4.79 4.78 4.77 4.76 4.75 4.32 4.30 4.29 3.37 2.75 2.73 2.72 2.70 2.69 2.67 2.66 2.66 2.65 2.64 2.63 2.62 2.59 2.50 1.79 1.78 1.77 1.75 1.75 1.73 1.73 1.72 1.71 1.70 1.69 1.68 1.68 1.66 1.65 1.64 1.58 1.56 1.55 1.54 1.53 1.53 1.52 1.51 1.49 1.49 1.48 1.47
1H NMR, 400 MHz, DMSO-d6
NC
20
2.00
2.01
1.02
2.00
2.02
3.98
2.13
11.5 10.5 9.5 8.5 7.5 6.5 5.5 4.5 3.5 2.5 1.5 0.5 -0.5
f1 (ppm)

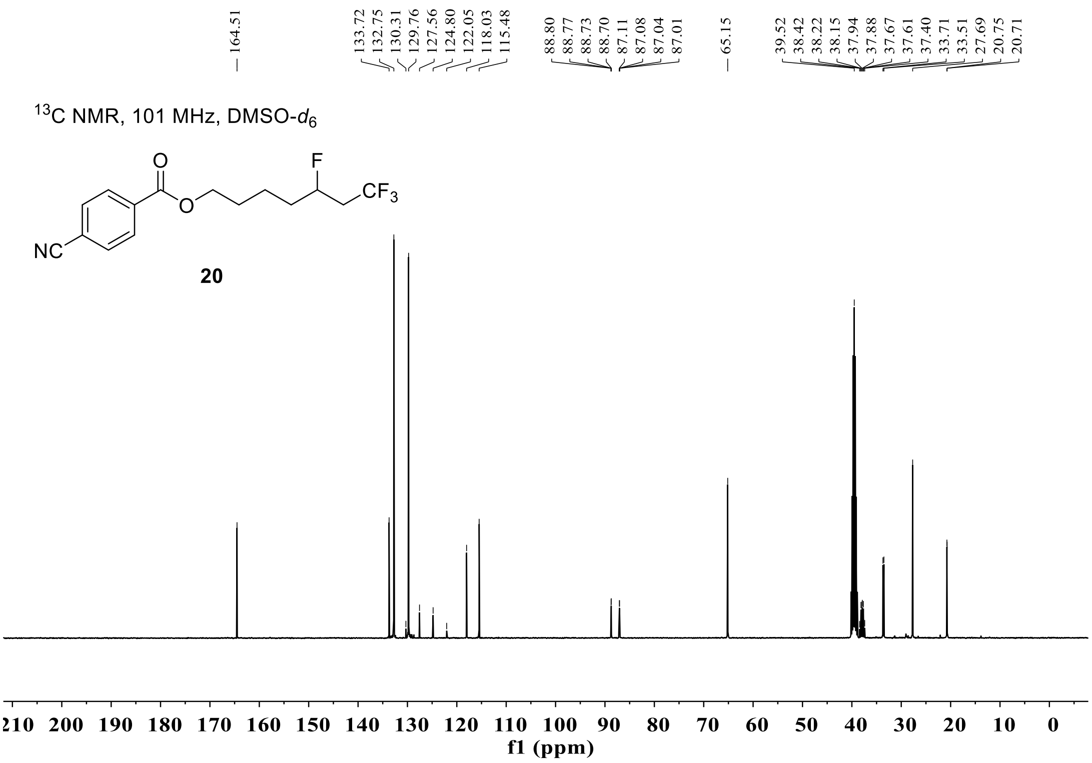
164.51
133.72
132.75
130.31
129.76
127.56
124.80
122.05
118.03
115.48
88.80
88.77
88.73
88.70
87.11
87.08
87.04
87.01
65.15
39.52
38.42
38.22
38.15
37.94
37.88
37.67
37.61
37.40
33.71
33.51
27.69
20.75
20.71
13C NMR, 101 MHz, DMSO-d6
O
F
O
CF3
NC
20
210 200 190 180 170 160 150 140 130 120 110 100 90 80 70 60 50 40 30 20 10 0
f1 (ppm)

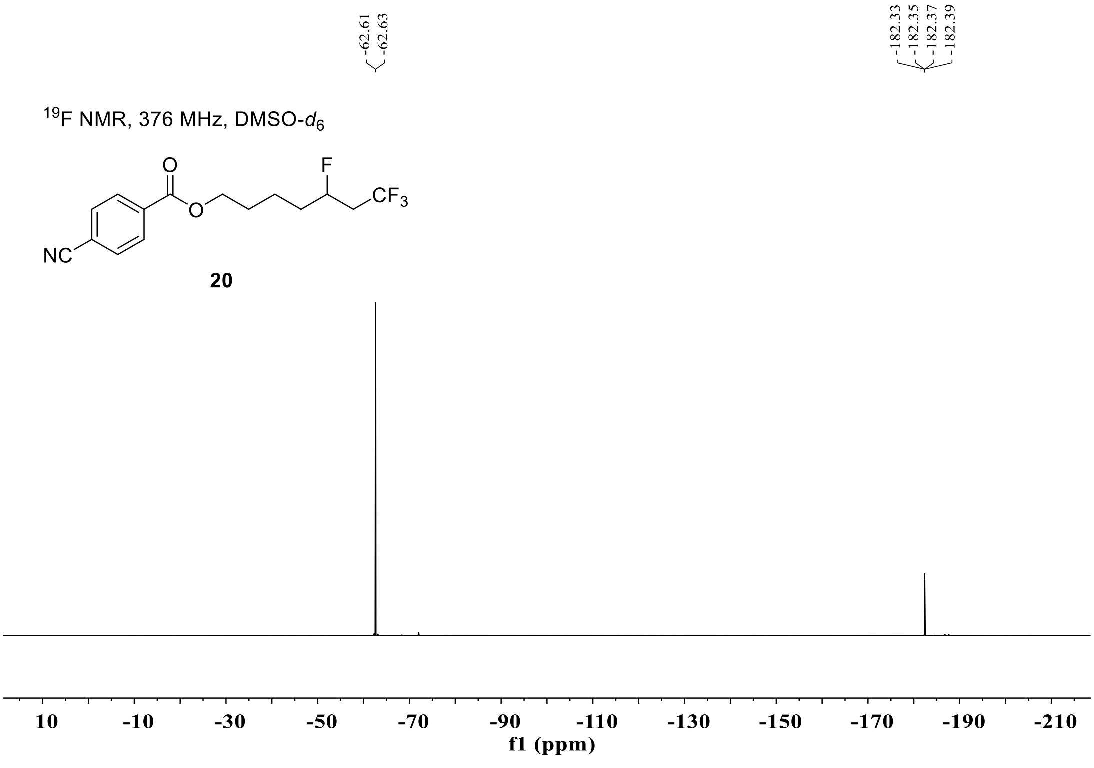
-62.61
-62.63
-182.33
-182.35
-182.37
-182.39
19F NMR, 376 MHz, DMSO-d6
O
F
O
CF3
NC
20
10 -10 -30 -50 -70 -90 -110 -130 -150 -170 -190 -210
f1 (ppm)

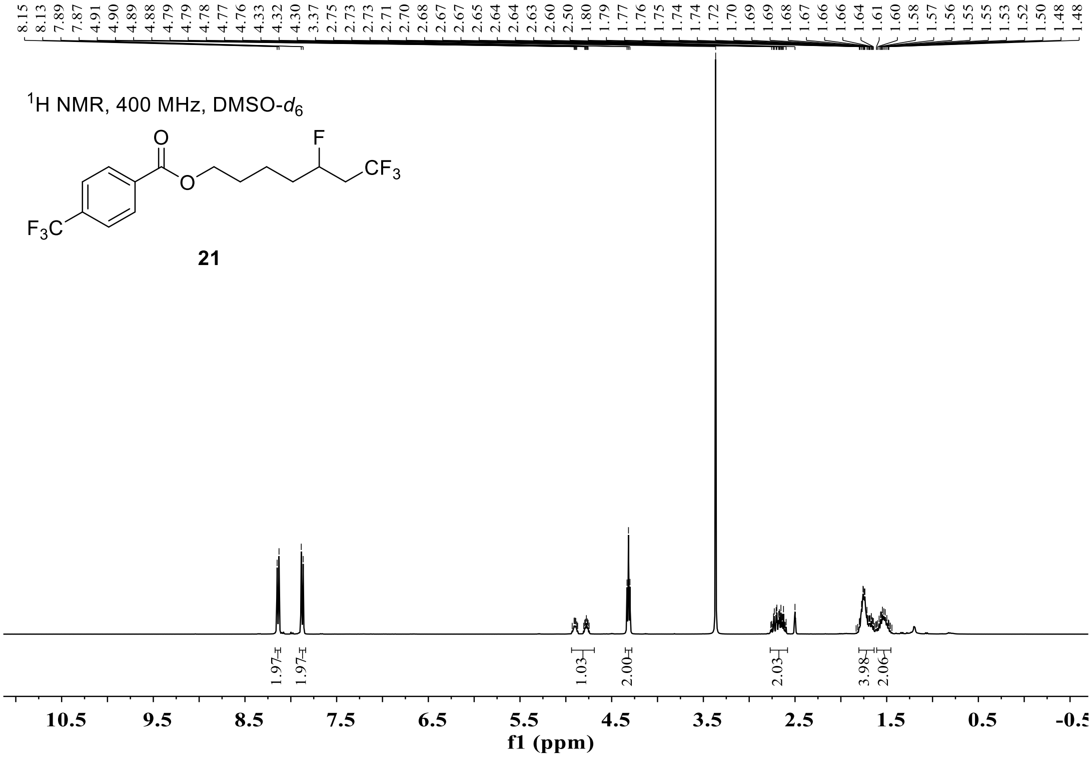

1H NMR, 400 MHz, DMSO-d6
21
f1 (ppm)

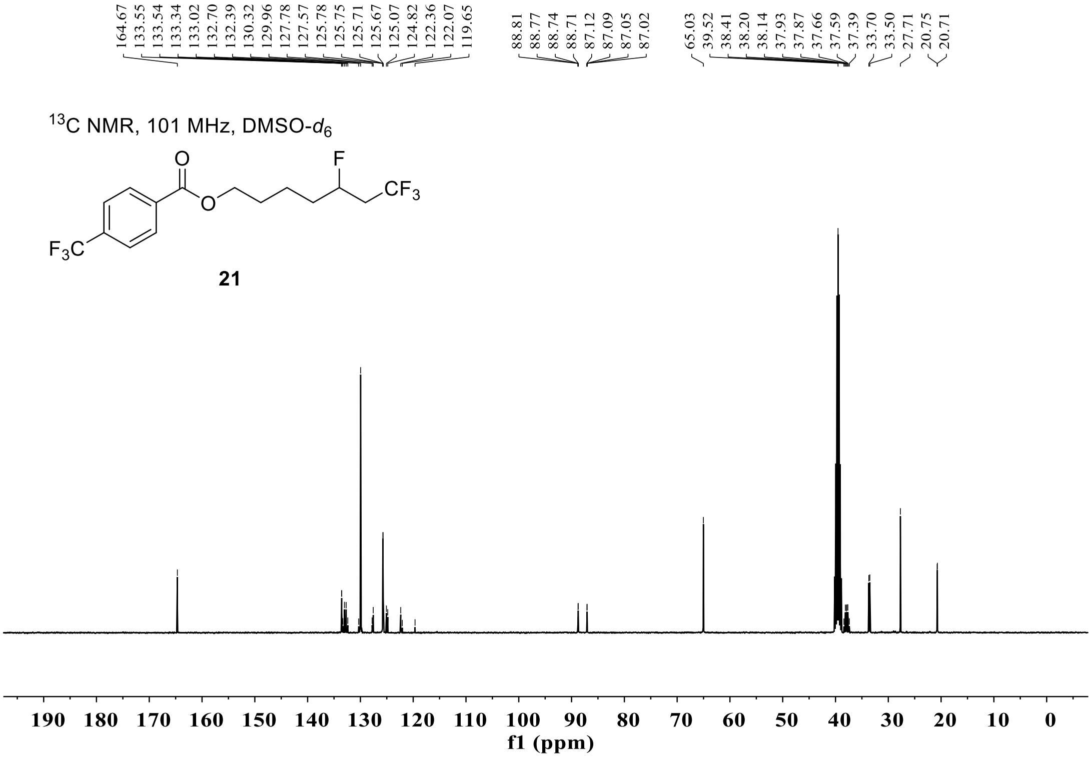

13C NMR, 101 MHz, DMSO-d6
21
f1 (ppm)

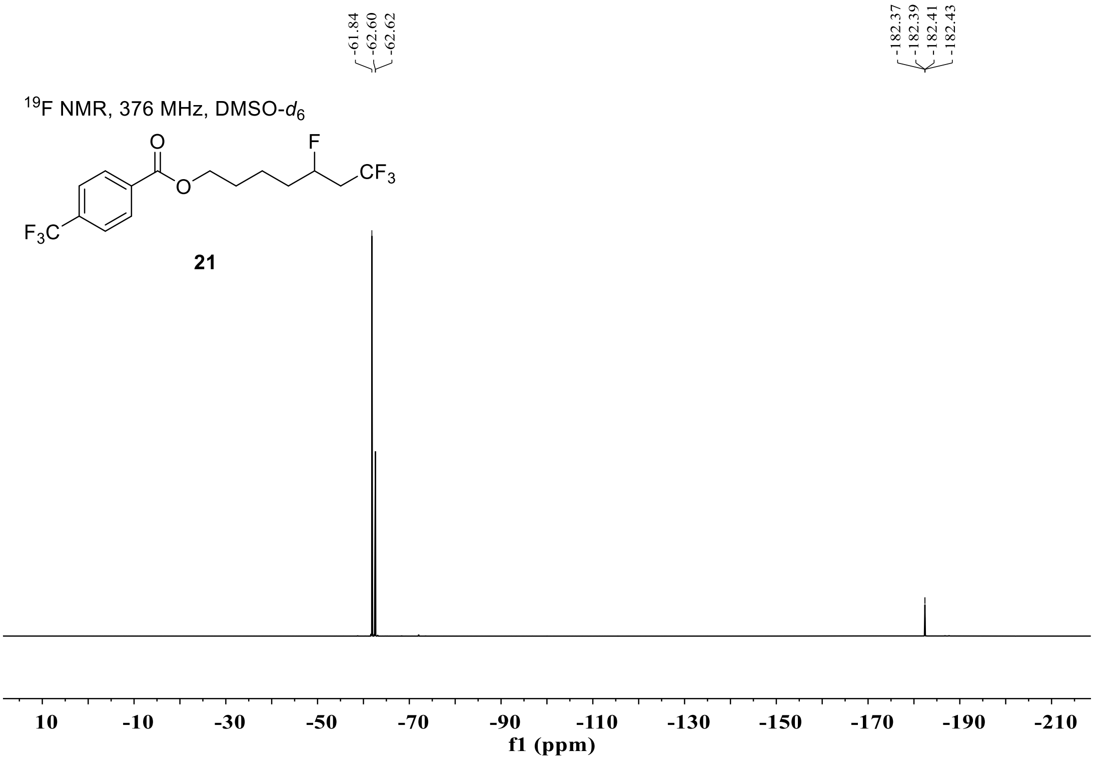

-61.84
-62.60
-62.62
-182.37
-182.39
-182.41
-182.43
^{19}F NMR, 376 MHz, DMSO-d_6
21
10 -10 -30 -50 -70 -90 -110 -130 -150 -170 -190 -210
f1 (ppm)

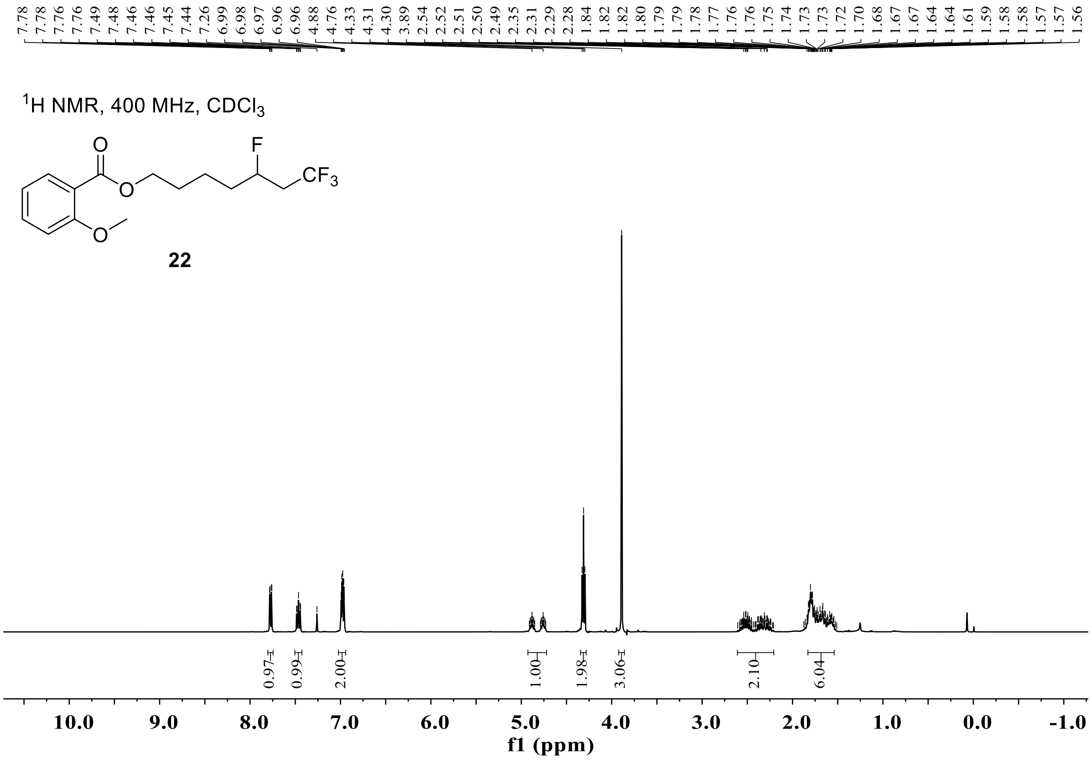

7.78 7.78 7.76 7.76 7.49 7.48 7.46 7.46 7.45 7.44 7.26 6.99 6.98 6.97 6.96 6.96 4.88 4.76 4.33 4.31 4.30 3.89 2.54 2.52 2.51 2.50 2.49 2.35 2.31 2.29 2.28 1.84 1.82 1.80 1.79 1.79 1.78 1.77 1.76 1.76 1.75 1.74 1.73 1.73 1.72 1.70 1.68 1.67 1.67 1.64 1.61 1.59 1.58 1.58 1.57 1.56
^{1}H NMR, 400 MHz, CDCl$_3$
22
0.97 0.99 2.00 1.00 1.98 3.06 2.10 6.04
10.0 9.0 8.0 7.0 6.0 5.0 4.0 3.0 2.0 1.0 0.0 -1.0
f1 (ppm)

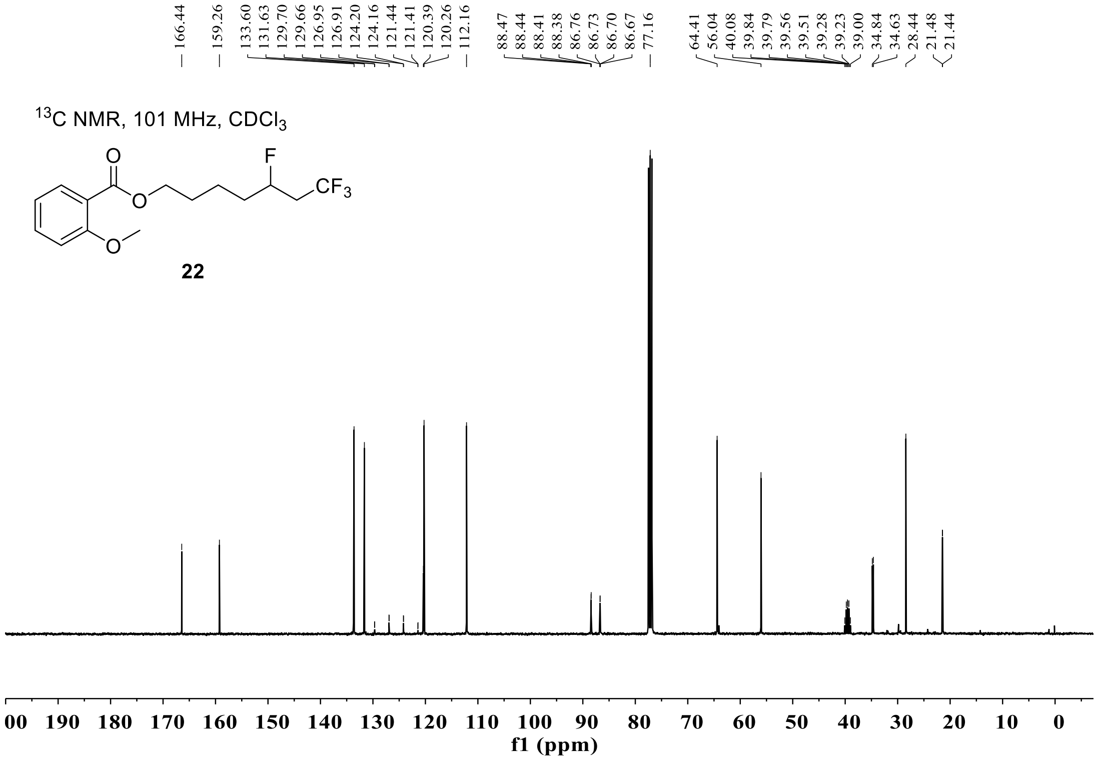
166.44
159.26
133.60
131.63
129.70
129.66
126.95
126.91
124.20
124.16
121.44
121.41
120.39
120.26
112.16
88.47
88.44
88.41
88.38
86.76
86.73
86.70
86.67
77.16
64.41
56.04
40.08
39.84
39.79
39.56
39.51
39.28
39.23
39.00
34.84
34.63
28.44
21.48
21.44
^{13}C NMR, 101 MHz, CDCl$_3$
O
F
O
CF$_3$
O
22
00 190 180 170 160 150 140 130 120 110 100 90 80 70 60 50 40 30 20 10 0
f1 (ppm)

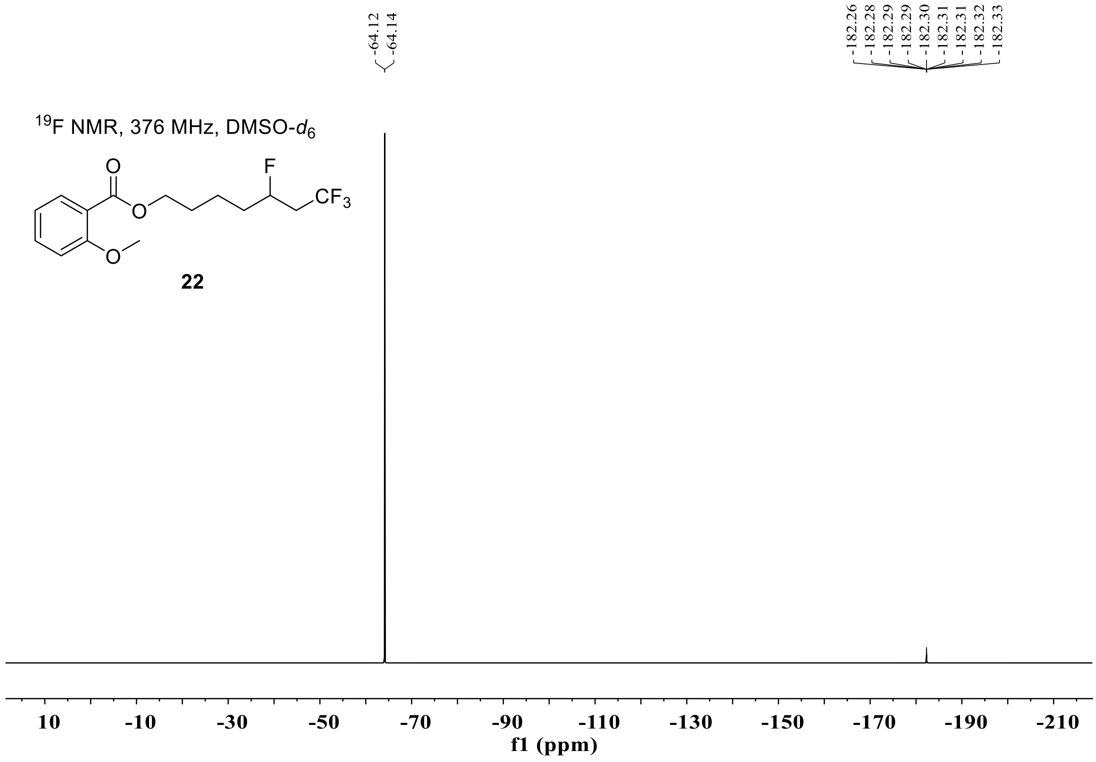
-64.12
-64.14
-182.26
-182.28
-182.29
-182.29
-182.30
-182.31
-182.31
-182.32
-182.33
^{19}F NMR, 376 MHz, DMSO-d_6
O
F
O
CF$_3$
O
22
10 -10 -30 -50 -70 -90 -110 -130 -150 -170 -190 -210
f1 (ppm)

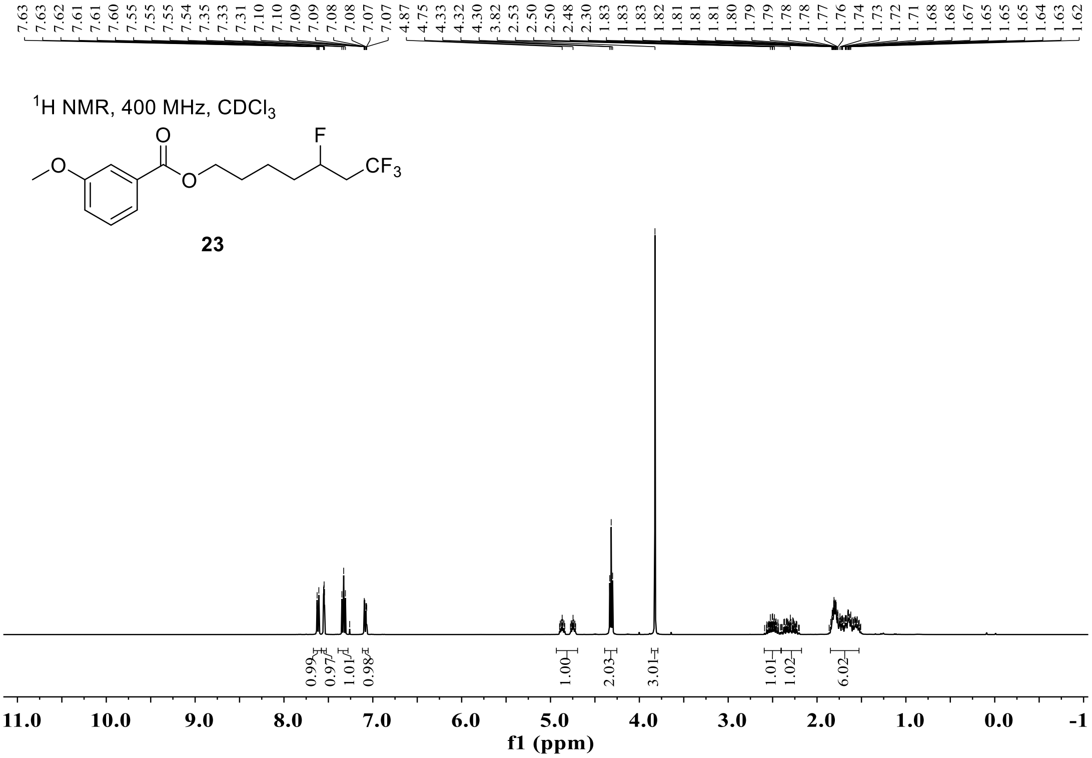
7.63 7.63 7.62 7.61 7.61 7.60 7.55 7.55 7.55 7.54 7.35 7.33 7.31 7.10 7.10 7.09 7.09 7.08 7.08 7.07 7.07 4.87 4.75 4.33 4.32 4.30 3.82 2.53 2.50 2.50 2.48 2.30 1.83 1.83 1.83 1.82 1.81 1.81 1.81 1.80 1.79 1.79 1.78 1.78 1.77 1.76 1.74 1.73 1.72 1.71 1.68 1.68 1.67 1.65 1.65 1.65 1.64 1.63 1.62
1H NMR, 400 MHz, CDCl3
O
F
O
O
CF3
23
0.99 0.97 1.01 0.98 1.00 2.03 3.01 1.01 1.02 6.02
11.0 10.0 9.0 8.0 7.0 6.0 5.0 4.0 3.0 2.0 1.0 0.0 -1
f1 (ppm)

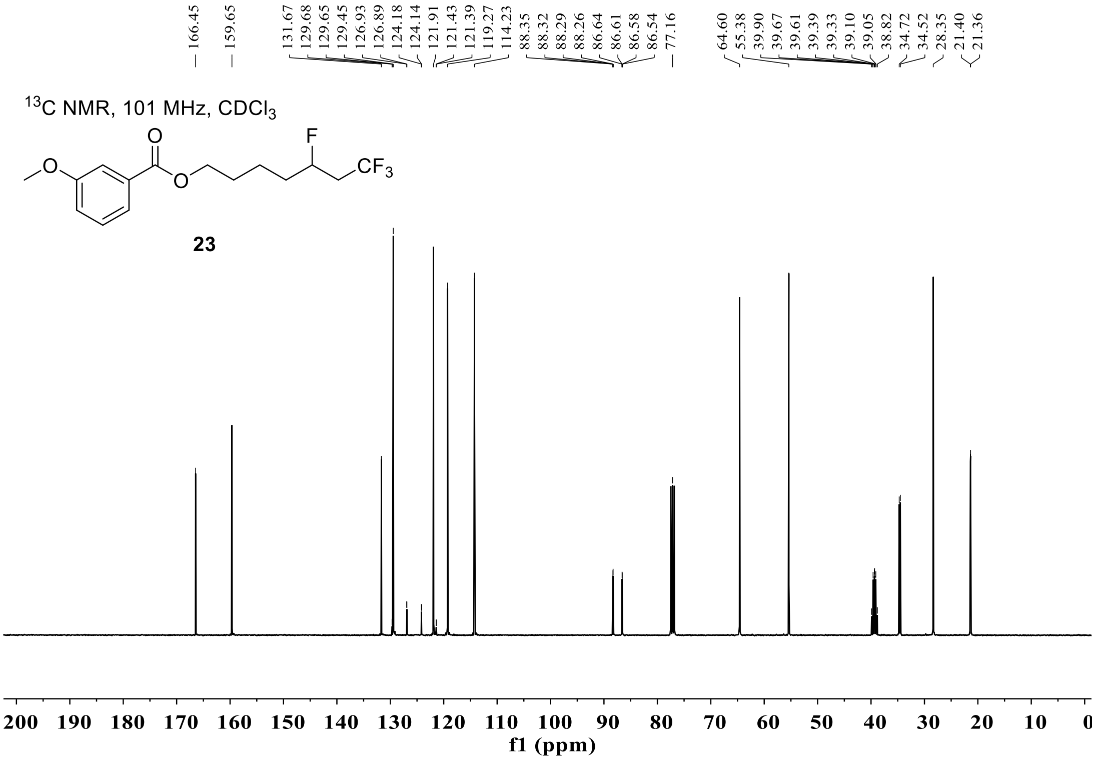
166.45 159.65 131.67 129.68 129.65 129.45 126.93 126.89 124.18 124.14 121.91 121.43 121.39 119.27 114.23 88.35 88.32 88.29 88.26 86.64 86.61 86.58 86.54 77.16 64.60 55.38 39.90 39.67 39.61 39.39 39.33 39.10 39.05 38.82 34.72 34.52 28.35 21.40 21.36
13C NMR, 101 MHz, CDCl3
O
F
O
O
CF3
23
200 190 180 170 160 150 140 130 120 110 100 90 80 70 60 50 40 30 20 10 0
f1 (ppm)

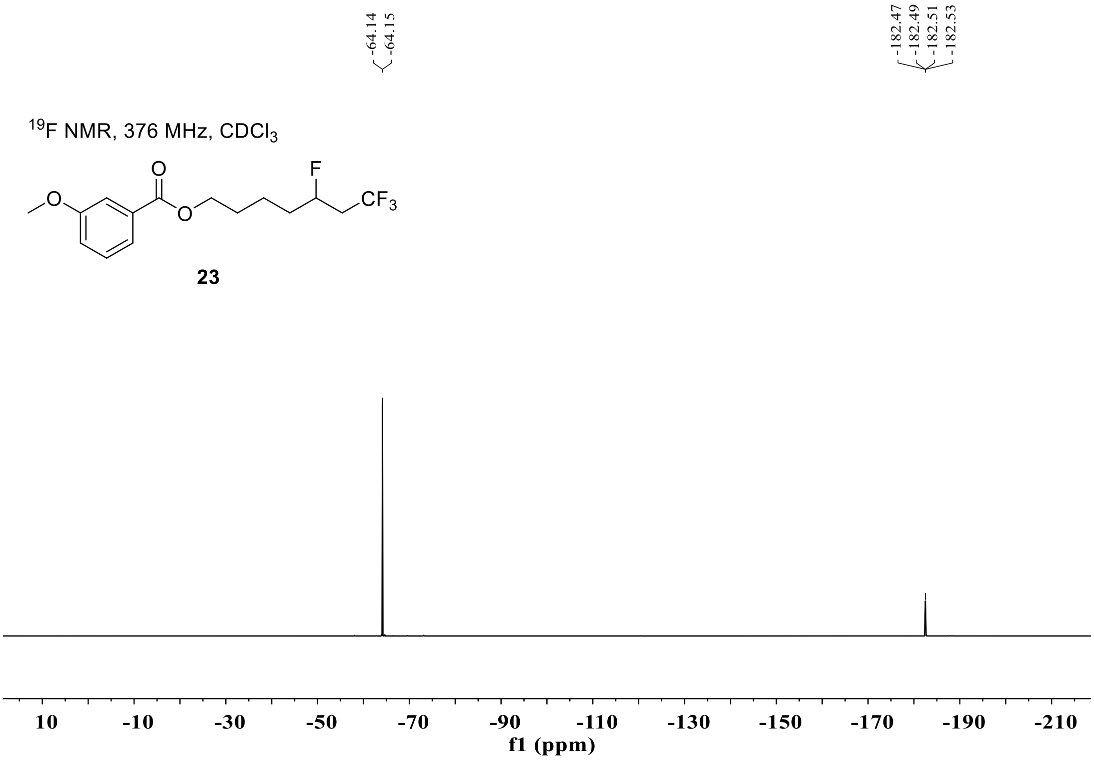
-64.14
-64.15
-182.47
-182.49
-182.51
-182.53
19F NMR, 376 MHz, CDCl3
O
F
O
O
CF3
23
10 -10 -30 -50 -70 -90 -110 -130 -150 -170 -190 -210
f1 (ppm)

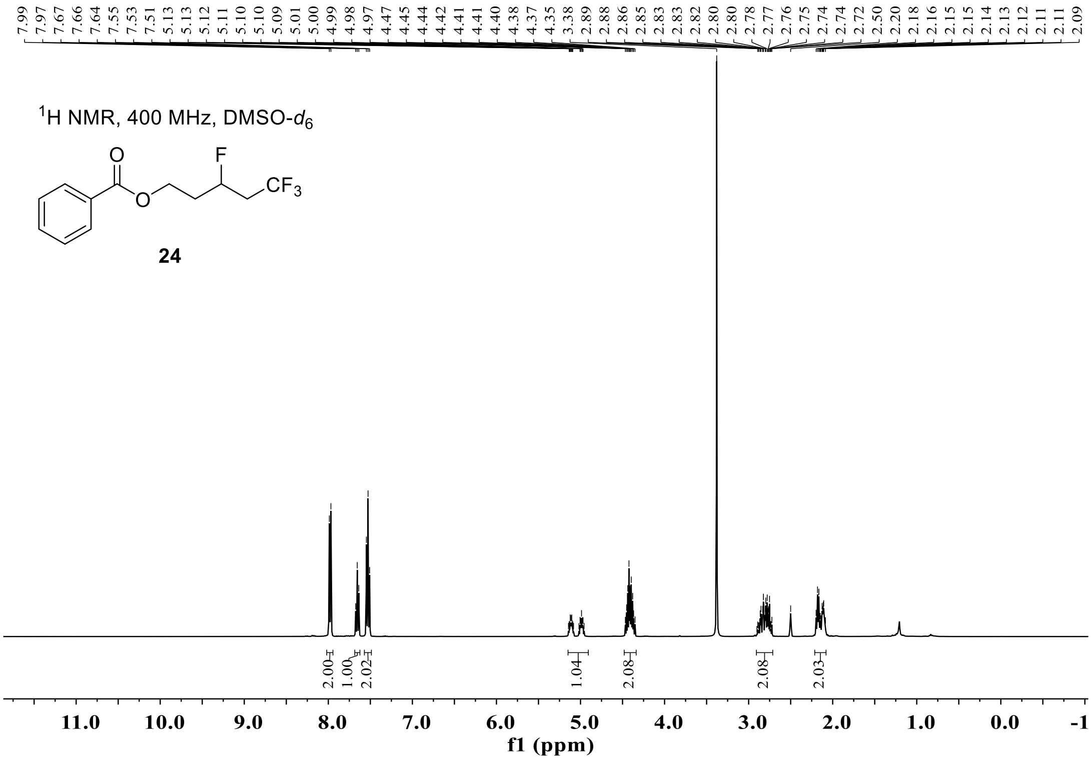
7.99 7.97 7.67 7.66 7.64 7.55 7.53 7.51 5.13 5.13 5.12 5.11 5.10 5.10 5.09 5.01 5.00 4.99 4.98 4.97 4.47 4.45 4.44 4.42 4.41 4.41 4.40 4.38 4.37 4.35 3.38 2.89 2.88 2.86 2.85 2.83 2.82 2.80 2.78 2.77 2.76 2.75 2.74 2.74 2.72 2.50 2.20 2.18 2.16 2.15 2.15 2.14 2.13 2.12 2.11 2.11 2.09
1H NMR, 400 MHz, DMSO-d6
O
F
O
CF3
24
2.00
1.00
2.02
1.04
2.08
2.08
2.03
11.0 10.0 9.0 8.0 7.0 6.0 5.0 4.0 3.0 2.0 1.0 0.0 -1
f1 (ppm)

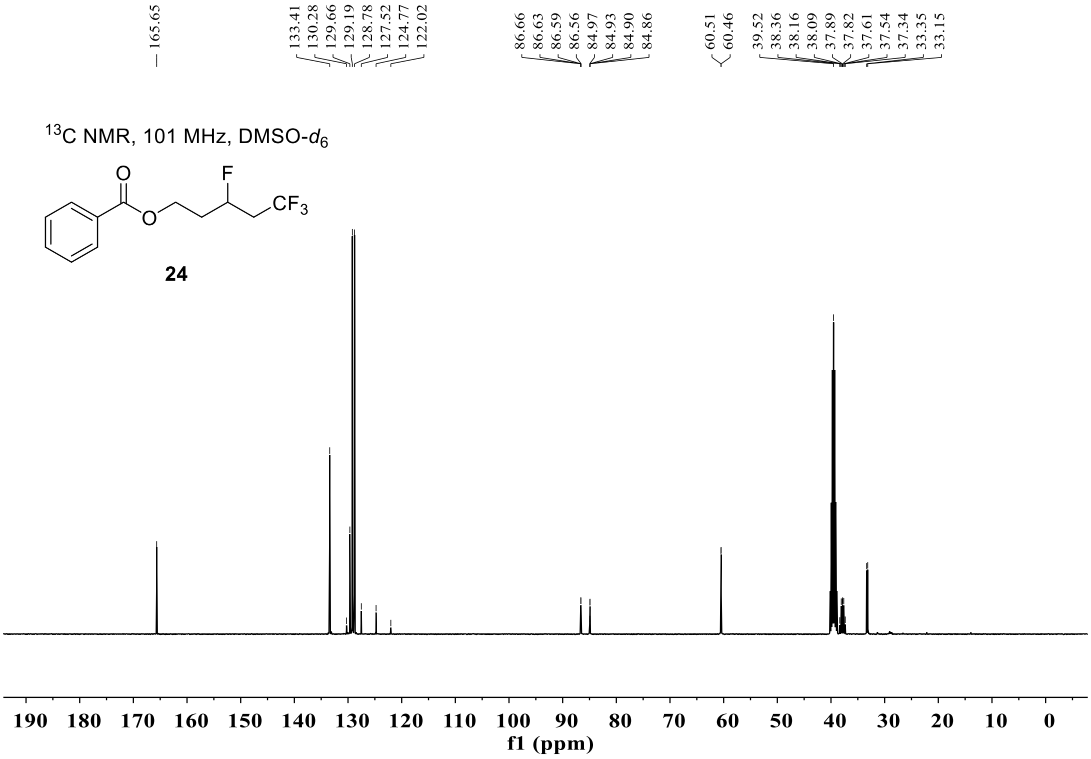
165.65
133.41
130.28
129.66
129.19
128.78
127.52
124.77
122.02
86.66
86.63
86.59
86.56
84.97
84.93
84.90
84.86
60.51
60.46
39.52
38.36
38.16
38.09
37.89
37.82
37.61
37.54
37.34
33.35
33.15
^{13}C NMR, 101 MHz, DMSO-d_6
O
F
O
CF_3
24
190 180 170 160 150 140 130 120 110 100 90 80 70 60 50 40 30 20 10 0
f1 (ppm)

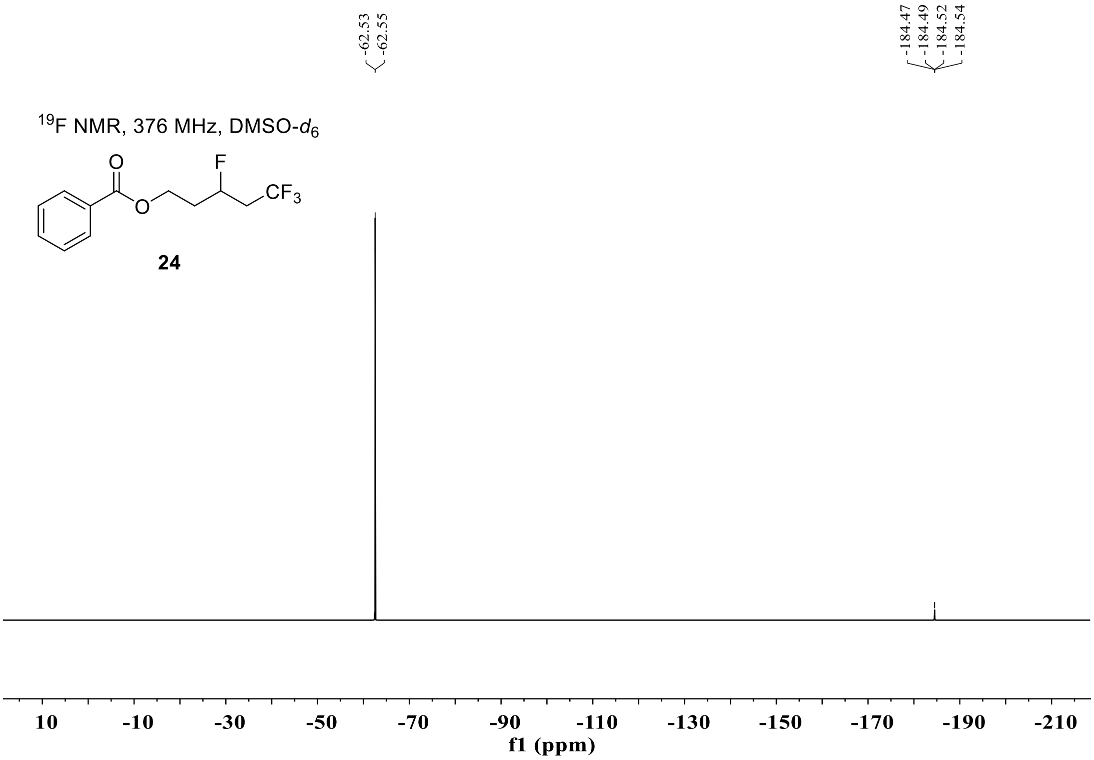
-62.53
-62.55
-184.47
-184.49
-184.52
-184.54
^{19}F NMR, 376 MHz, DMSO-d_6
O
F
O
CF_3
24
10 -10 -30 -50 -70 -90 -110 -130 -150 -170 -190 -210
f1 (ppm)

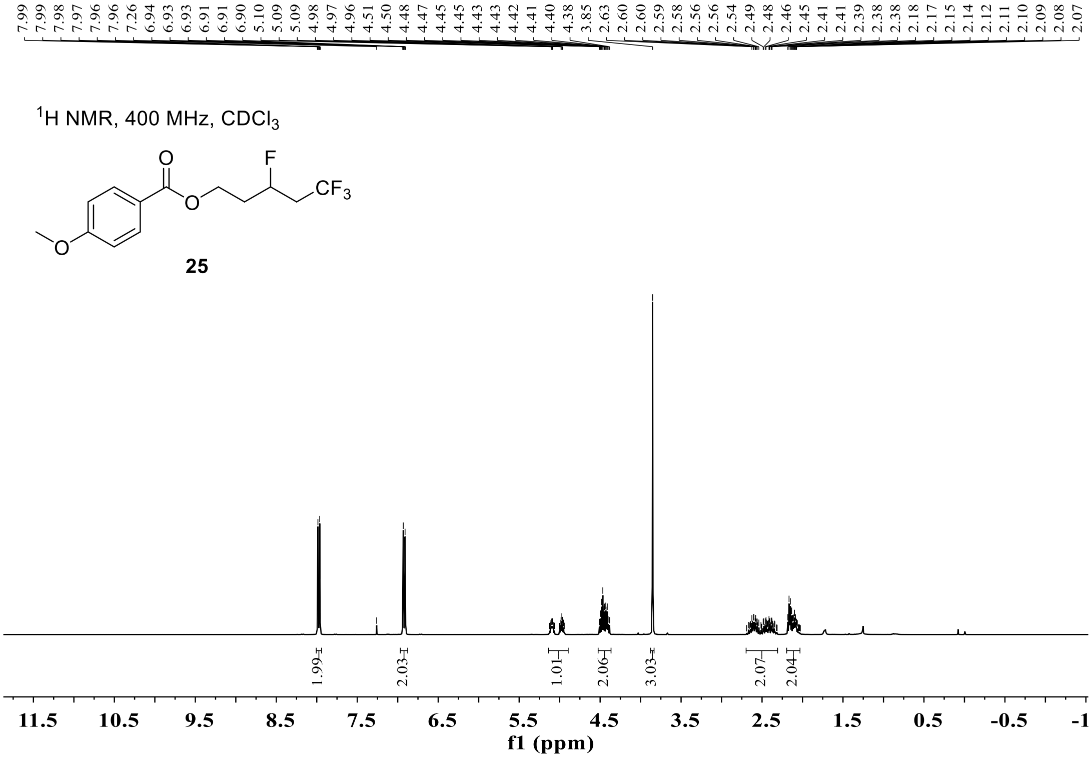

^{1}H NMR, 400 MHz, CDCl$_3$
25
f1 (ppm)

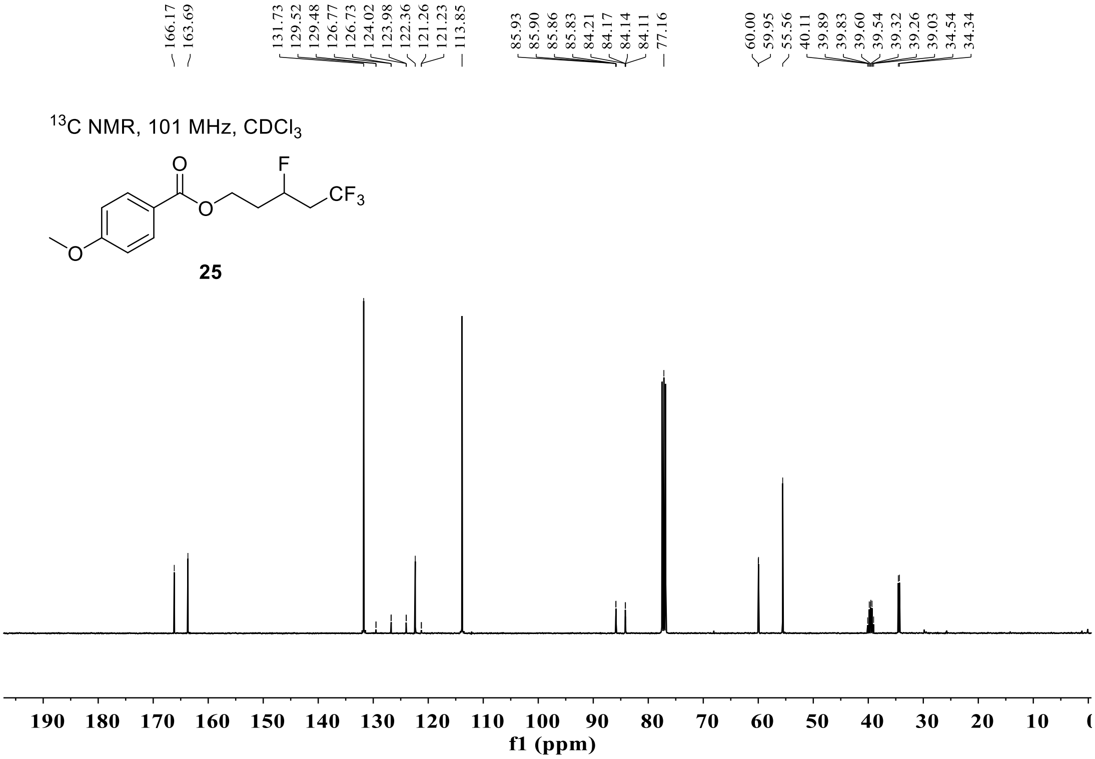

^{13}C NMR, 101 MHz, CDCl$_3$
25
f1 (ppm)

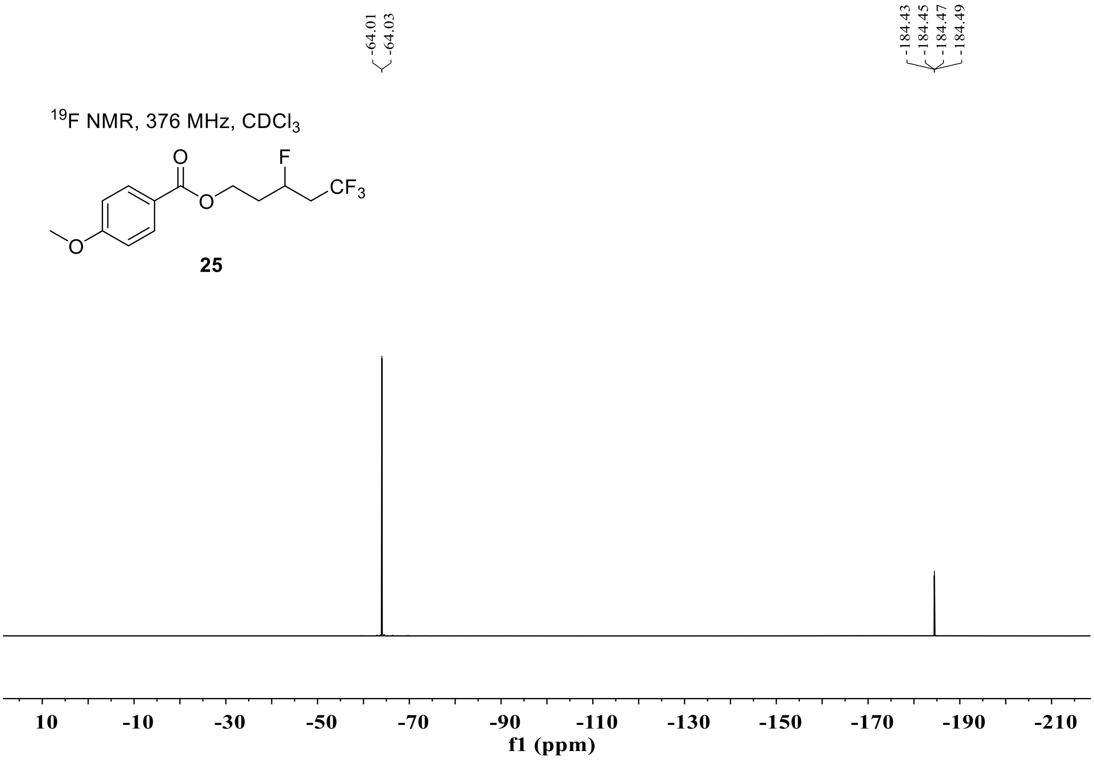
-64.01
-64.03
-184.43
-184.45
-184.47
-184.49
19F NMR, 376 MHz, CDCl3
O
F
O
CF3
O
25
10
-10
-30
-50
-70
-90
-110
-130
-150
-170
-190
-210
f1 (ppm)

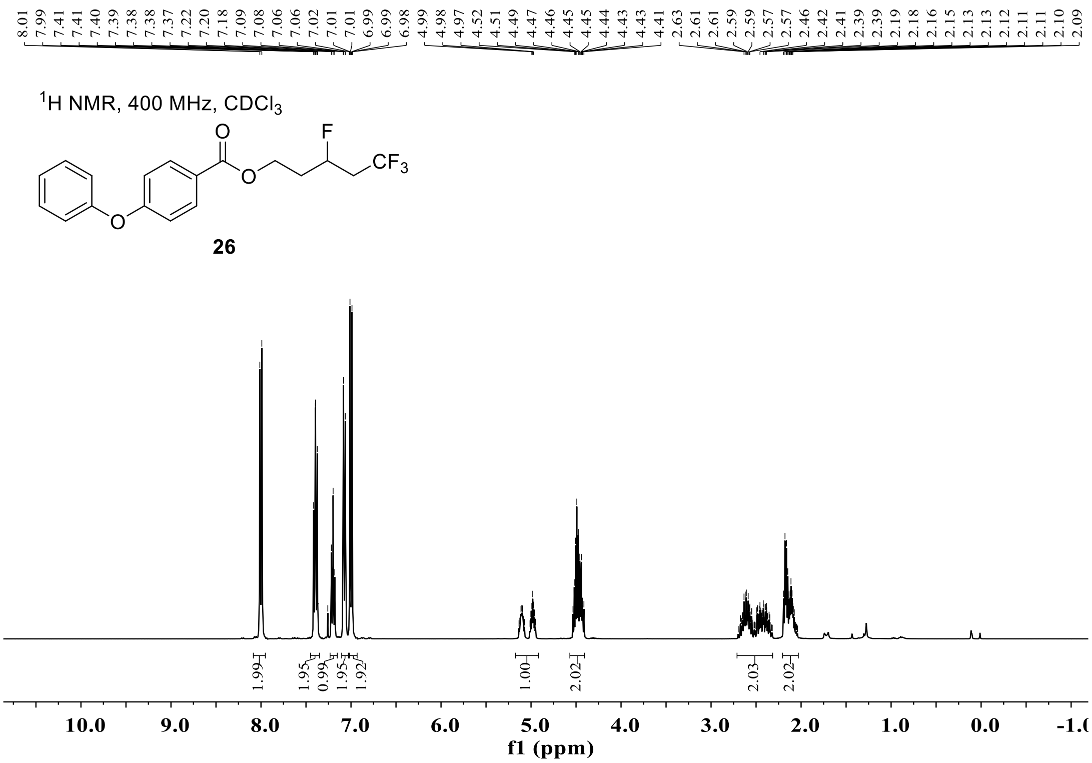
1H NMR, 400 MHz, CDCl3
O
F
O
CF3
O
26
1.99
1.95
0.99
1.95
1.92
1.00
2.02
2.03
2.02
10.0
9.0
8.0
7.0
6.0
5.0
4.0
3.0
2.0
1.0
0.0
f1 (ppm)

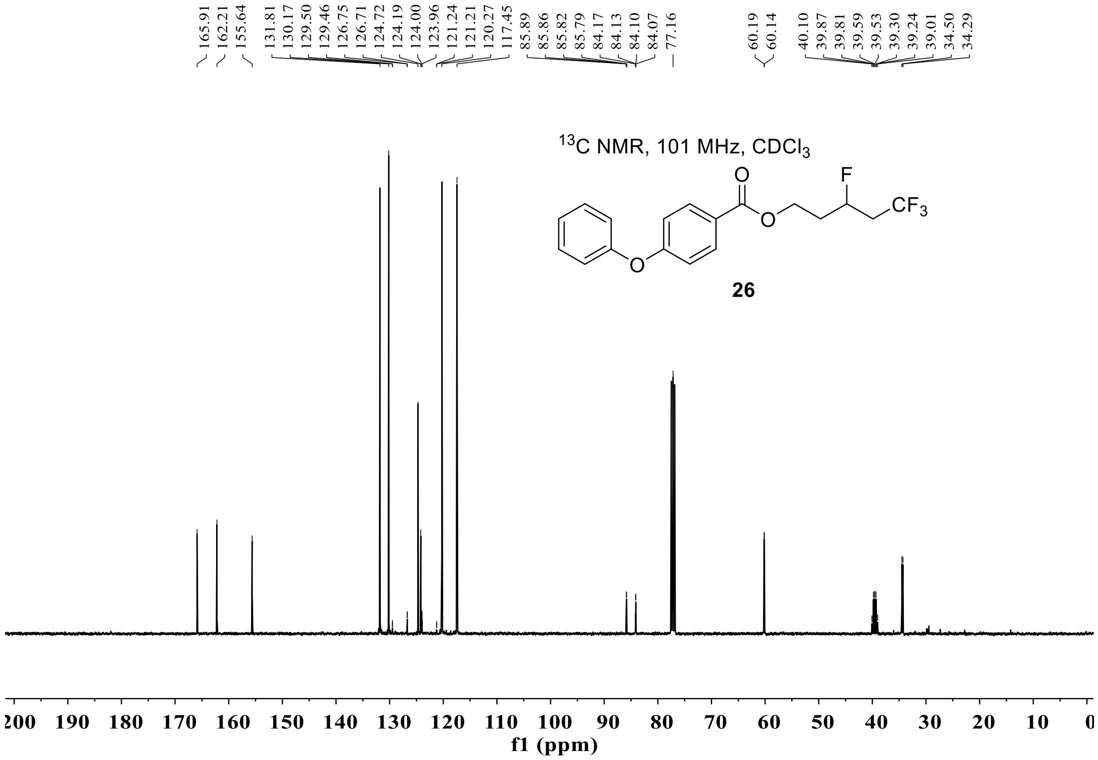
165.91
162.21
155.64
131.81
130.17
129.50
129.46
126.75
126.71
124.72
124.19
124.00
123.96
121.24
121.21
120.27
117.45
85.89
85.86
85.82
85.79
84.17
84.13
84.10
84.07
77.16
60.19
60.14
40.10
39.87
39.81
39.59
39.53
39.30
39.24
39.01
34.50
34.29
^{13}C NMR, 101 MHz, $CDCl_3$
O
F
CF_3
O
O
26
200 190 180 170 160 150 140 130 120 110 100 90 80 70 60 50 40 30 20 10 0
f1 (ppm)

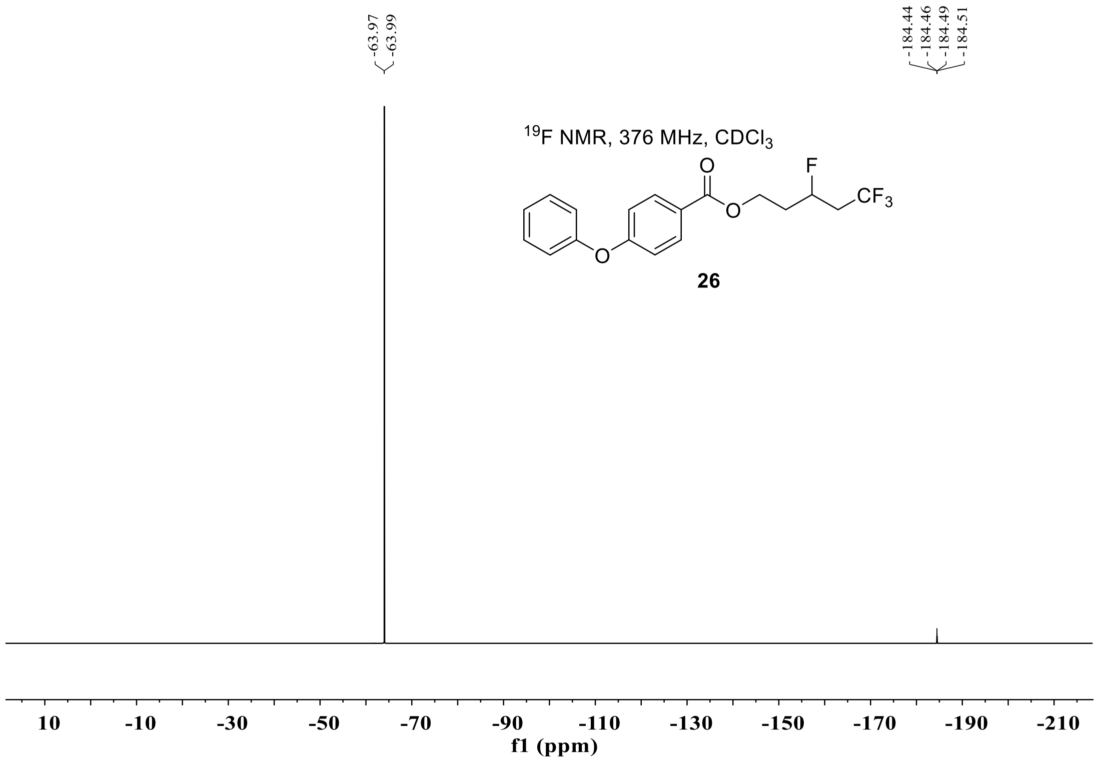
-63.97
-63.99
-184.44
-184.46
-184.49
-184.51
^{19}F NMR, 376 MHz, $CDCl_3$
O
F
CF_3
O
O
26
10 -10 -30 -50 -70 -90 -110 -130 -150 -170 -190 -210
f1 (ppm)

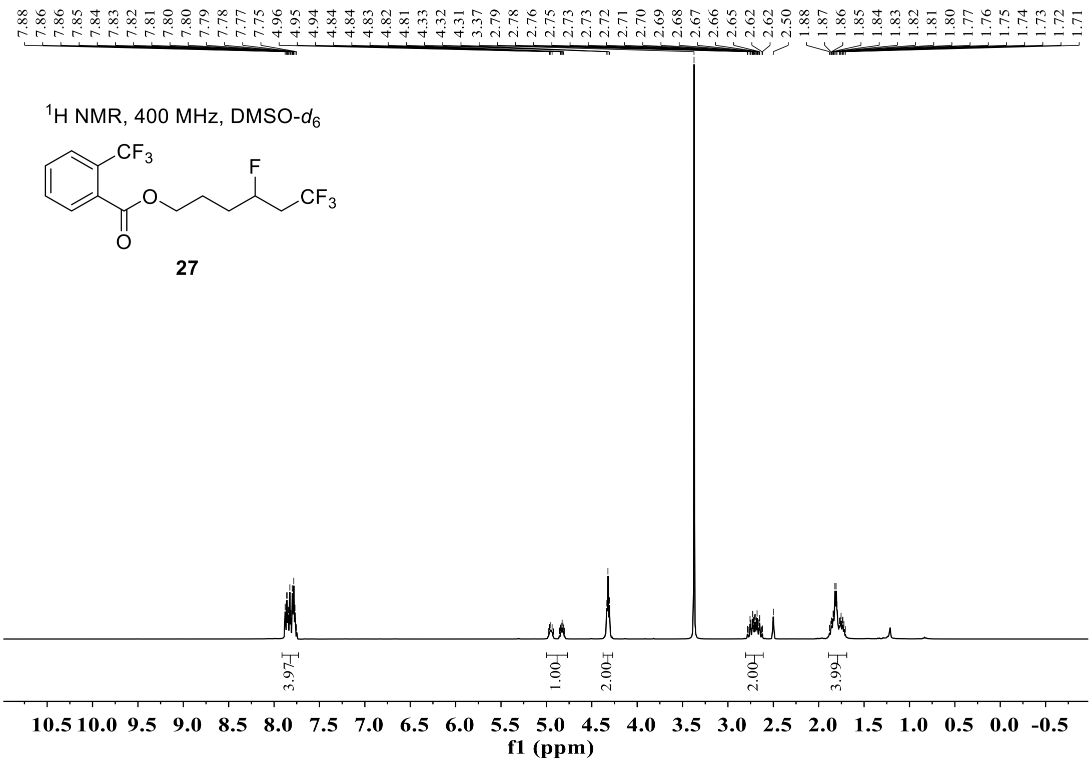
1H NMR, 400 MHz, DMSO-d6
CF3
F
O
CF3
O
27
3.97
1.00
2.00
2.00
3.99
f1 (ppm)

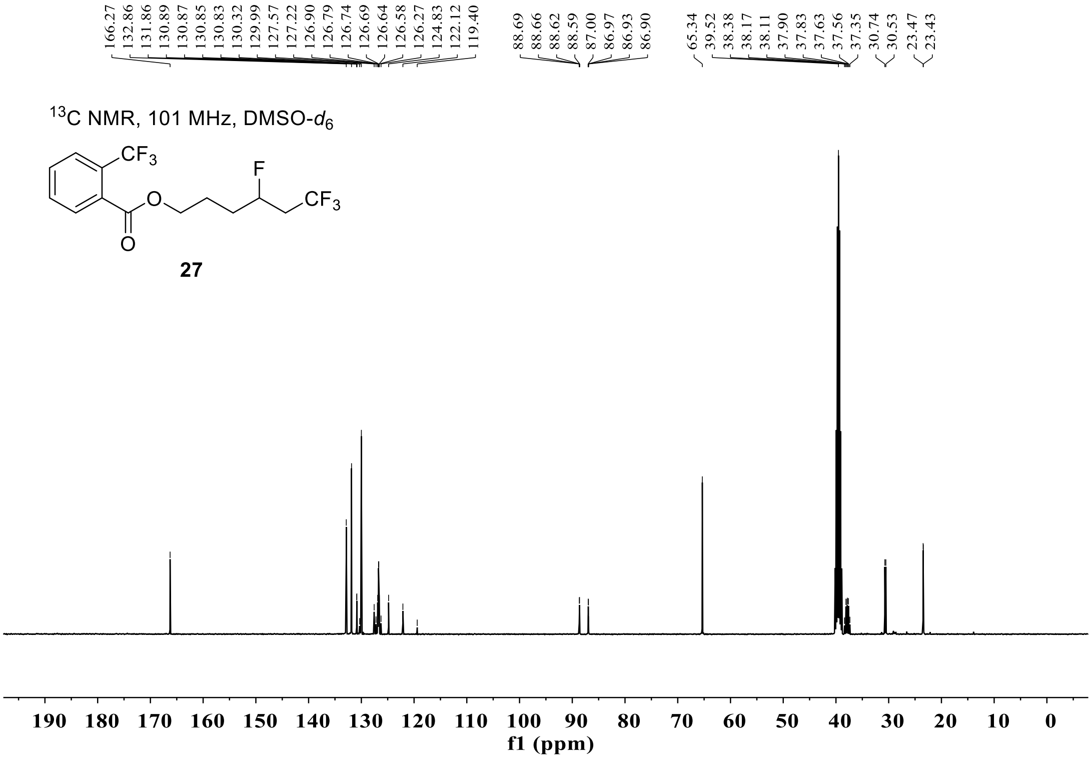
13C NMR, 101 MHz, DMSO-d6
CF3
F
O
CF3
O
27
f1 (ppm)

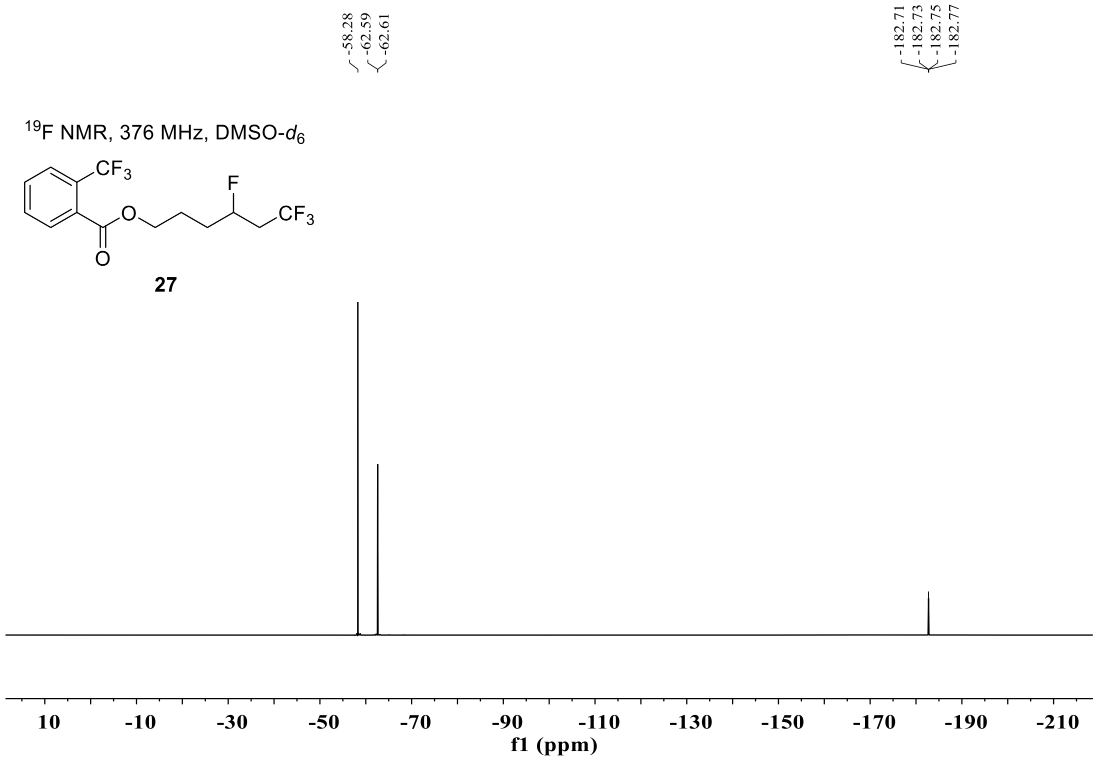
-58.28
-62.59
-62.61
-182.71
-182.73
-182.75
-182.77
19F NMR, 376 MHz, DMSO-d6
CF3
F
O
CF3
O
27
10 -10 -30 -50 -70 -90 -110 -130 -150 -170 -190 -210
f1 (ppm)

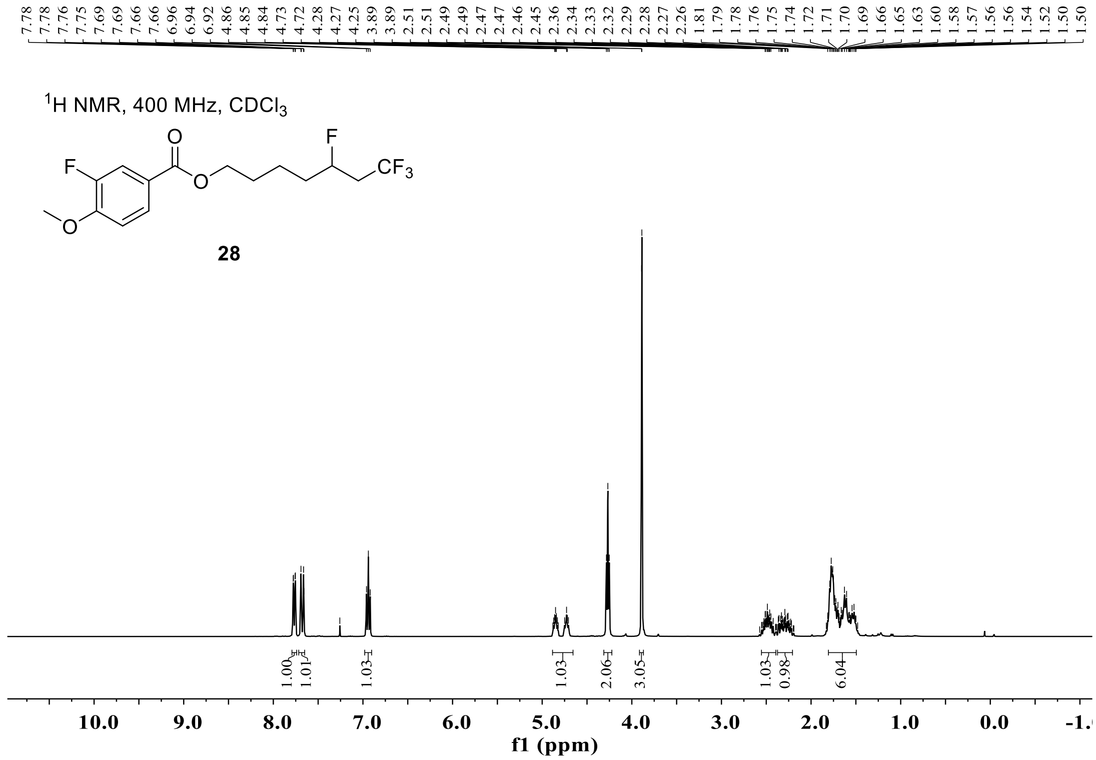
7.78 7.78 7.76 7.75 7.69 7.69 7.66 7.66 6.96 6.94 6.92 4.86 4.85 4.84 4.73 4.72 4.28 4.27 4.25 3.89 3.89 2.51 2.51 2.49 2.47 2.47 2.46 2.45 2.36 2.34 2.33 2.32 2.29 2.28 2.27 2.26 1.81 1.79 1.78 1.76 1.75 1.74 1.72 1.71 1.70 1.69 1.66 1.65 1.63 1.60 1.58 1.57 1.56 1.54 1.52 1.50 1.50
1H NMR, 400 MHz, CDCl3
O
F
F
O
CF3
O
28
1.00
1.01
1.03
1.03
2.06
3.05
1.03
0.98
6.04
10.0 9.0 8.0 7.0 6.0 5.0 4.0 3.0 2.0 1.0 0.0
f1 (ppm)

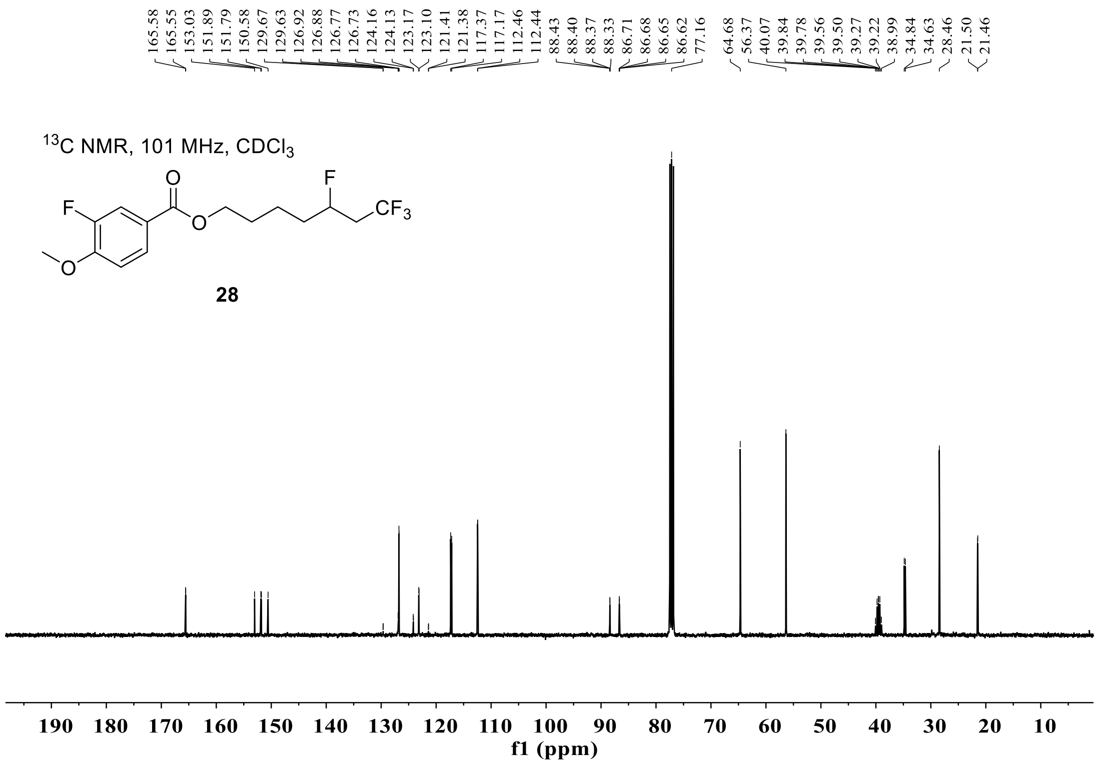
165.58
165.55
153.03
151.89
151.79
150.58
129.67
129.63
126.92
126.88
126.77
126.73
124.16
124.13
123.17
123.10
121.41
121.38
117.37
117.17
112.46
112.44
88.43
88.40
88.37
88.33
86.71
86.68
86.65
86.62
77.16
64.68
56.37
40.07
39.84
39.78
39.56
39.50
39.27
39.22
38.99
34.84
34.63
28.46
21.50
21.46
^{13}C NMR, 101 MHz, CDCl$_3$
O
F
F
CF$_3$
O
O
28
190 180 170 160 150 140 130 120 110 100 90 80 70 60 50 40 30 20 10
f1 (ppm)

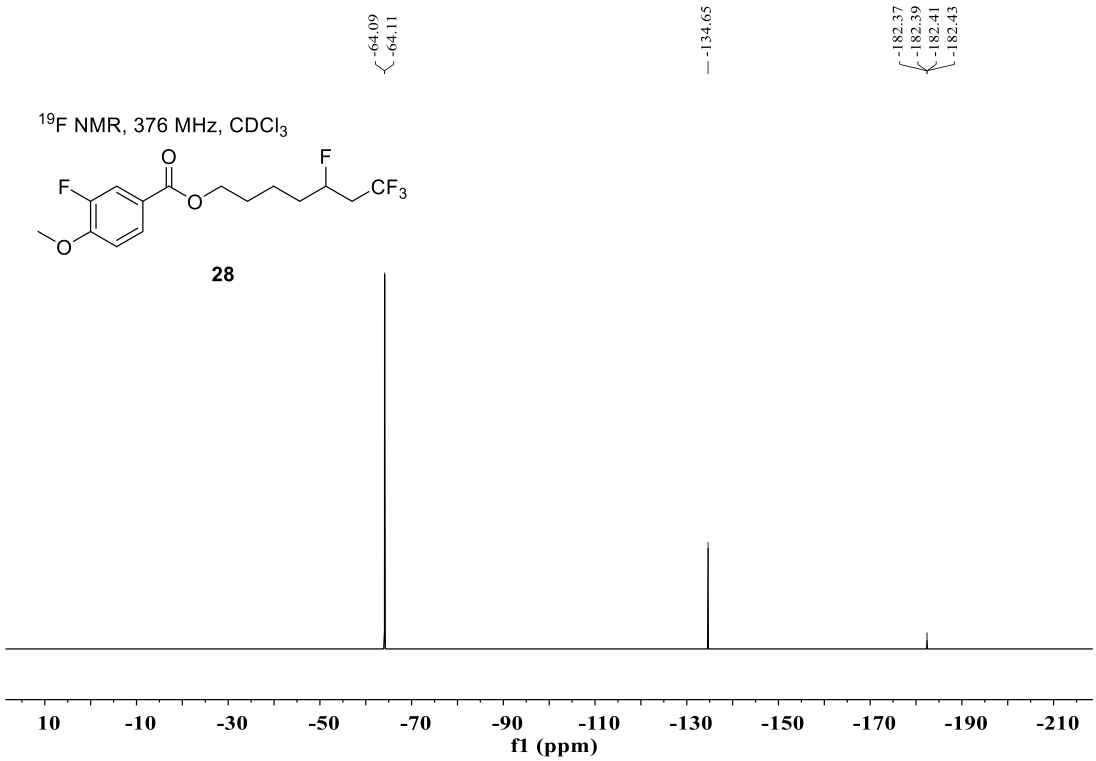
-64.09
-64.11
-134.65
-182.37
-182.39
-182.41
-182.43
^{19}F NMR, 376 MHz, CDCl$_3$
O
F
F
CF$_3$
O
O
28
10 -10 -30 -50 -70 -90 -110 -130 -150 -170 -190 -210
f1 (ppm)

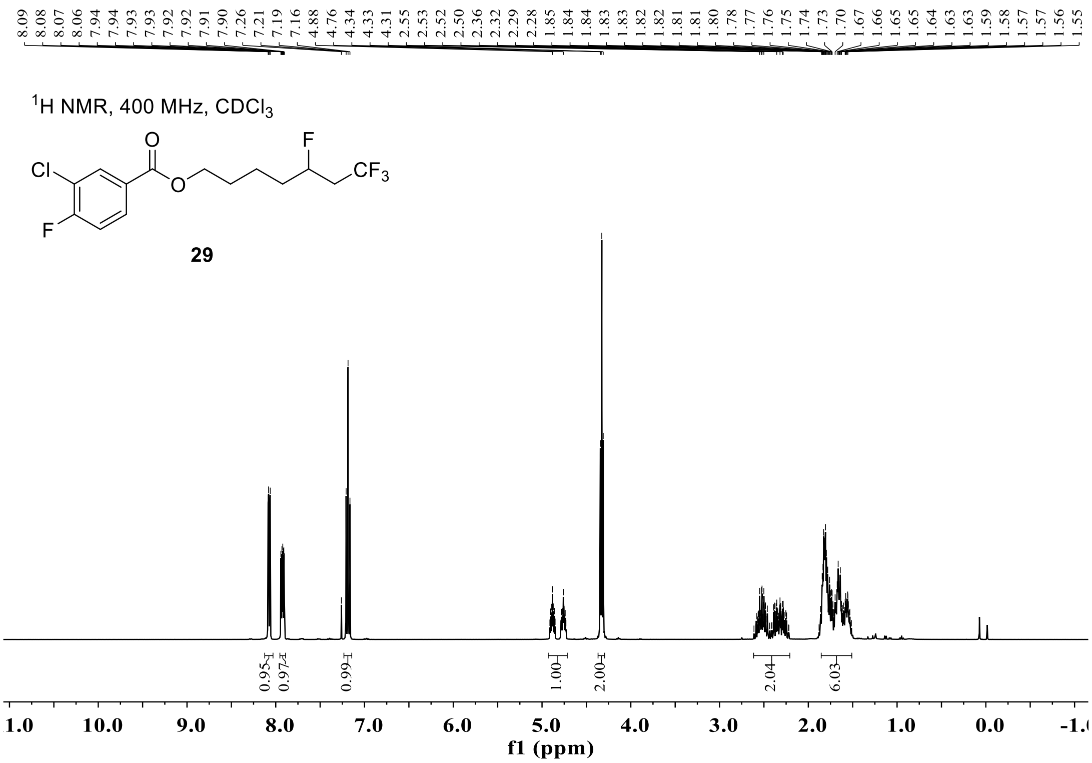
8.09 8.08 8.07 8.06 7.94 7.94 7.93 7.93 7.92 7.92 7.91 7.90 7.26 7.21 7.19 7.16 4.88 4.76 4.34 4.33 4.31 2.55 2.53 2.52 2.50 2.36 2.32 2.29 2.28 1.85 1.84 1.84 1.83 1.83 1.82 1.82 1.81 1.81 1.80 1.78 1.77 1.76 1.75 1.74 1.73 1.70 1.67 1.66 1.65 1.65 1.64 1.63 1.63 1.59 1.58 1.57 1.57 1.56 1.55
^{1}H NMR, 400 MHz, CDCl$_3$
O
F
Cl
O
CF$_3$
F
29
0.95
0.97
0.99
1.00
2.00
2.04
6.03
1.0 10.0 9.0 8.0 7.0 6.0 5.0 4.0 3.0 2.0 1.0 0.0
f1 (ppm)

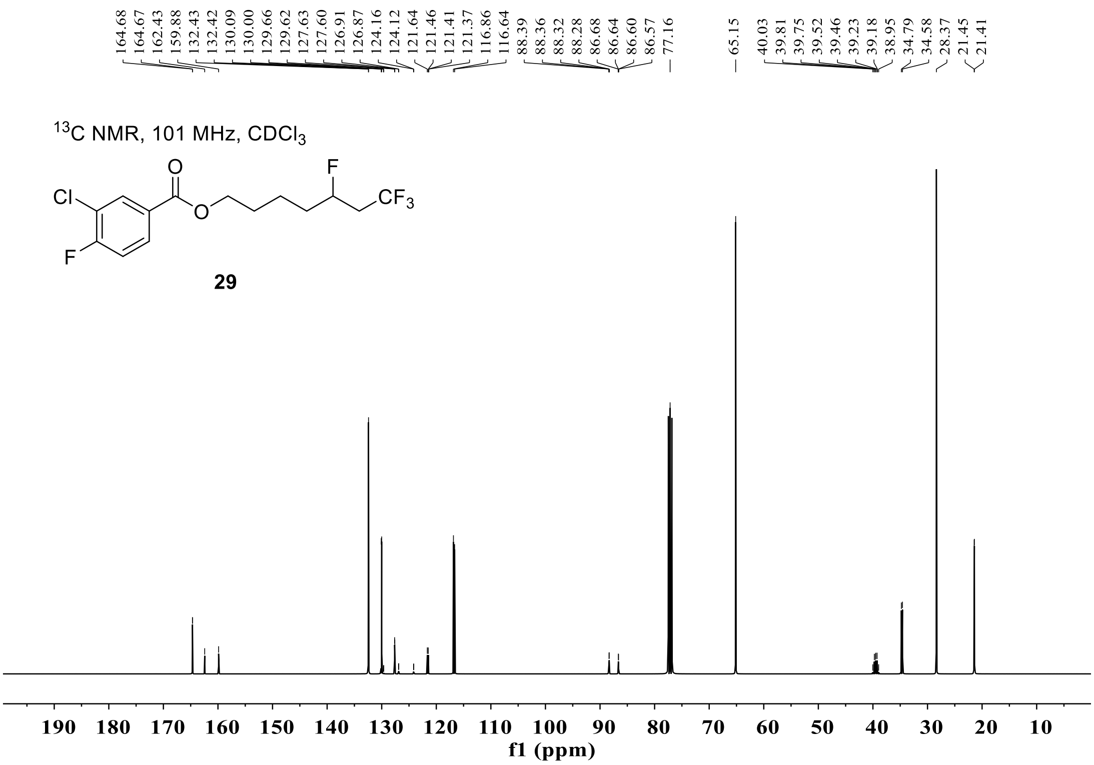
164.68 164.67 162.43 159.88 132.43 132.42 130.09 130.00 129.66 129.62 127.63 127.60 126.91 126.87 124.16 124.12 121.64 121.46 121.41 121.37 116.86 116.64 88.39 88.36 88.32 88.28 86.68 86.64 86.60 86.57 77.16
65.15
40.03 39.81 39.75 39.52 39.46 39.23 39.18 38.95 34.79 34.58 28.37 21.45 21.41
^{13}C NMR, 101 MHz, CDCl$_3$
O
F
Cl
O
CF$_3$
F
29
190 180 170 160 150 140 130 120 110 100 90 80 70 60 50 40 30 20 10
f1 (ppm)

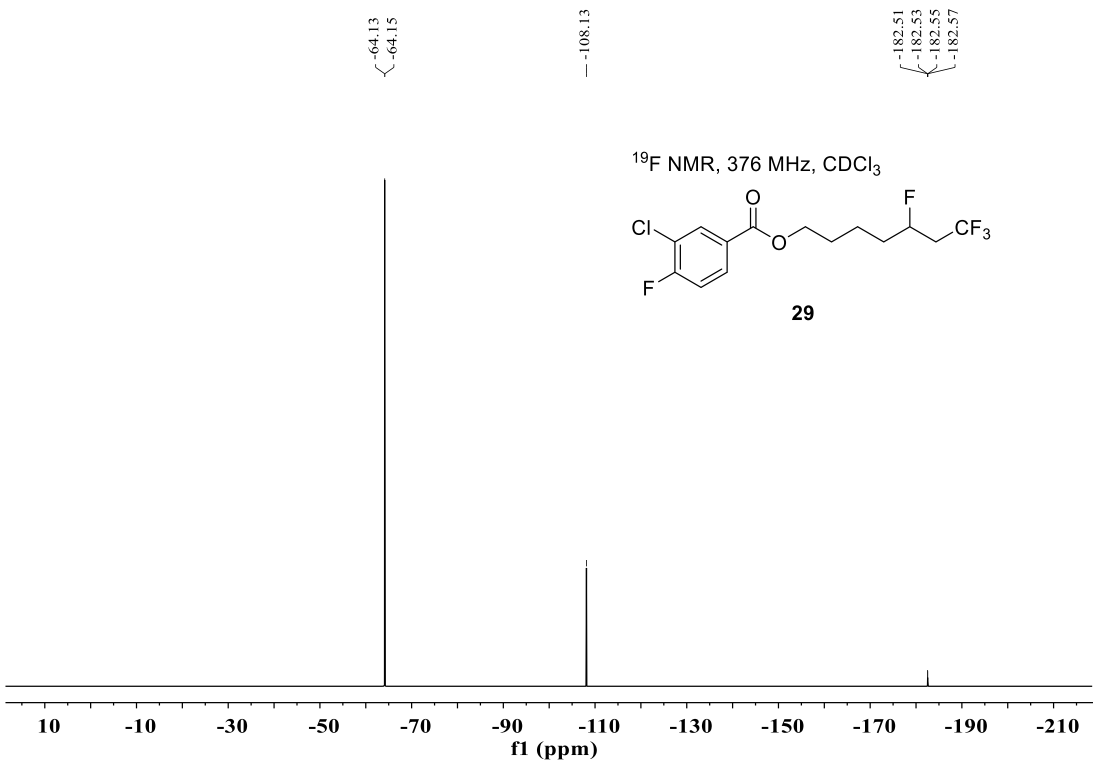
-64.13
-64.15
-108.13
-182.51
-182.53
-182.55
-182.57
19F NMR, 376 MHz, CDCl3
29
10
-10
-30
-50
-70
-90
-110
-130
-150
-170
-190
-210
f1 (ppm)

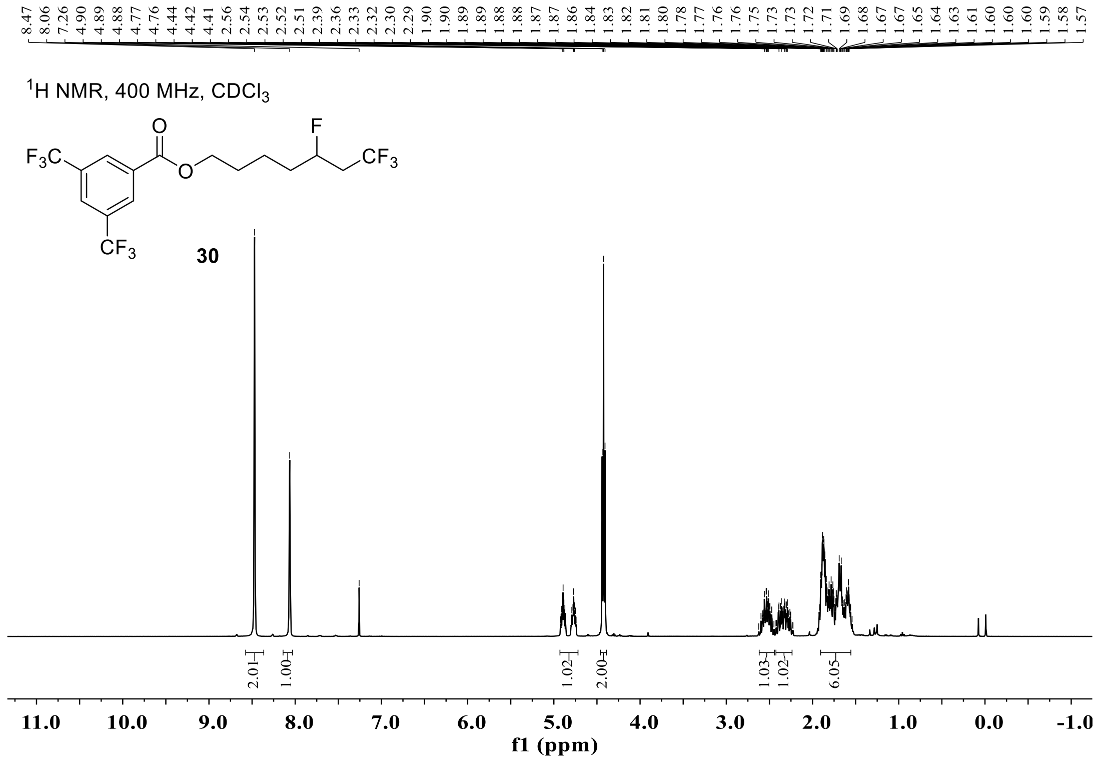
1H NMR, 400 MHz, CDCl3
30
2.01
1.00
1.02
2.00
1.03
1.02
6.05
11.0
10.0
9.0
8.0
7.0
6.0
5.0
4.0
3.0
2.0
1.0
0.0
-1.0
f1 (ppm)

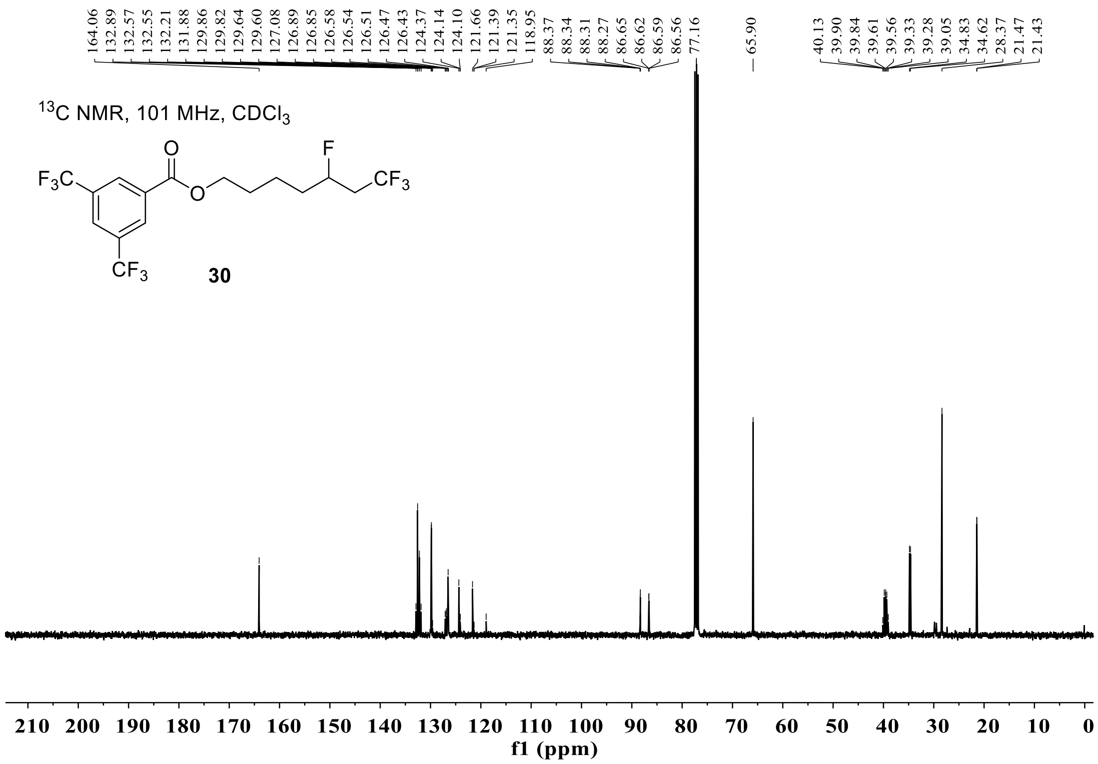
164.06
132.89
132.57
132.55
132.21
131.88
129.86
129.82
129.64
129.60
127.08
126.89
126.85
126.58
126.54
126.51
126.47
126.43
124.37
124.14
124.10
121.66
121.39
121.35
118.95
88.37
88.34
88.31
88.27
86.65
86.62
86.59
86.56
77.16
65.90
40.13
39.90
39.84
39.61
39.56
39.33
39.28
39.05
34.83
34.62
28.37
21.47
21.43
^{13}C NMR, 101 MHz, CDCl$_3$
O
F
F$_3$C
O
CF$_3$
CF$_3$
30
210 200 190 180 170 160 150 140 130 120 110 100 90 80 70 60 50 40 30 20 10 0
f1 (ppm)

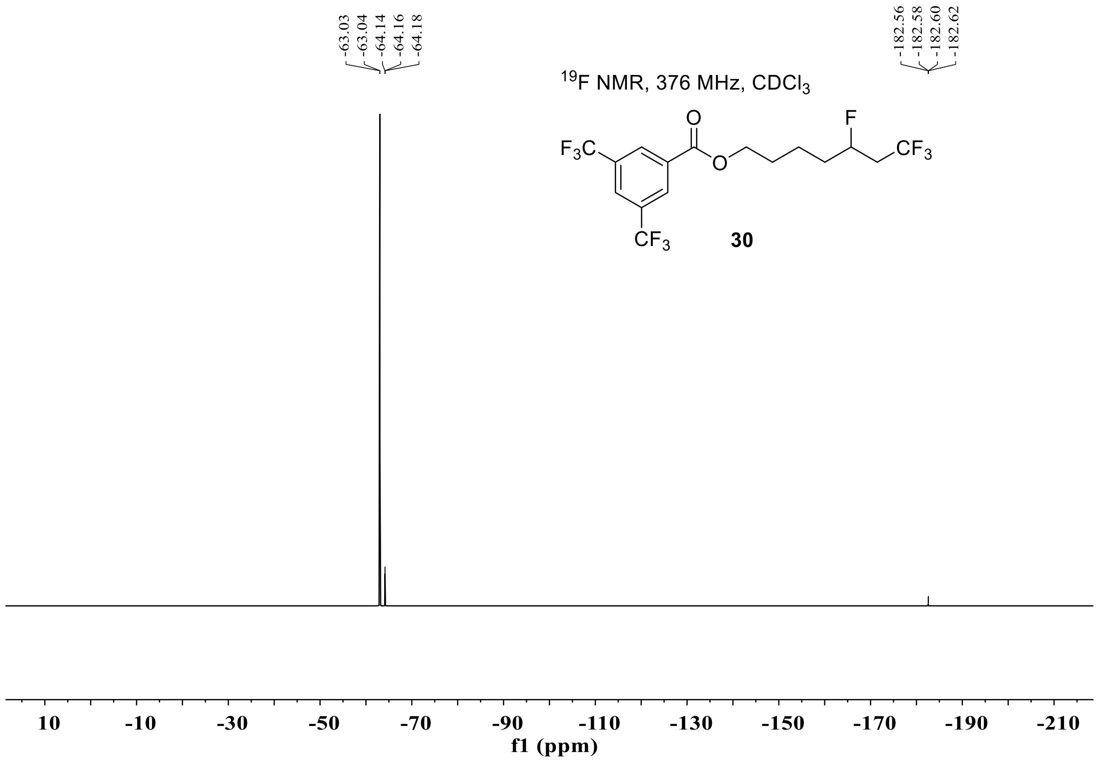
-63.03
-63.04
-64.14
-64.16
-64.18
-182.56
-182.58
-182.60
-182.62
^{19}F NMR, 376 MHz, CDCl$_3$
O
F
F$_3$C
O
CF$_3$
CF$_3$
30
10 -10 -30 -50 -70 -90 -110 -130 -150 -170 -190 -210
f1 (ppm)

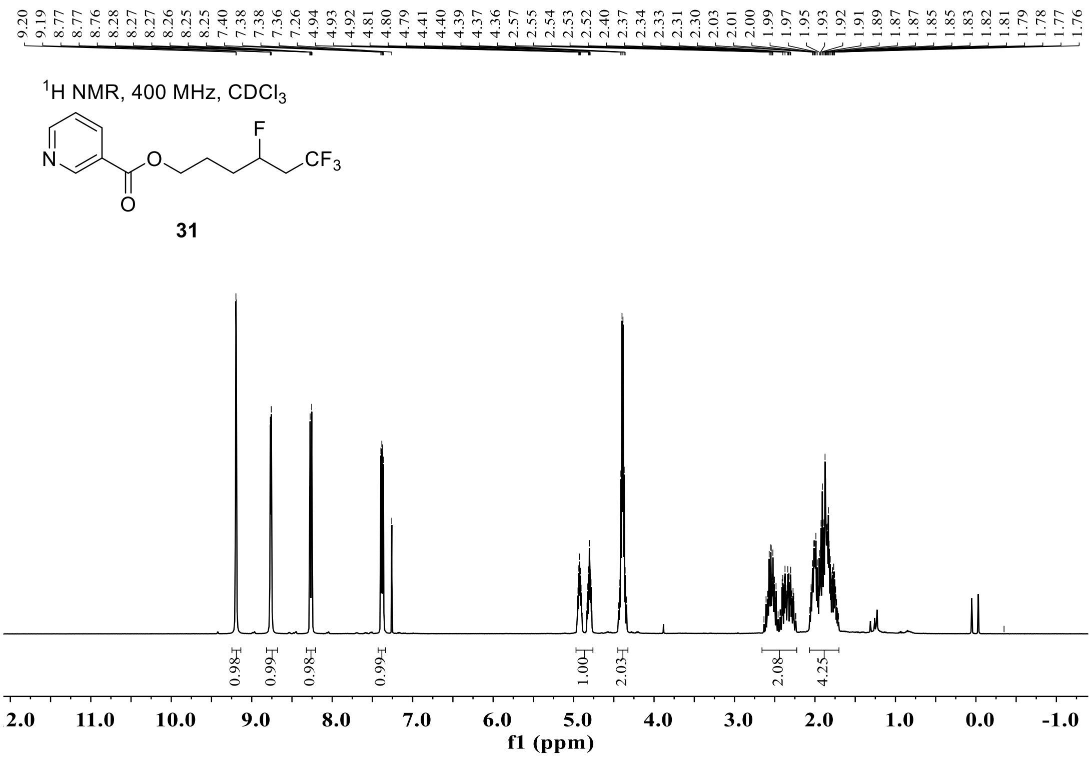
9.20 9.19 8.77 8.77 8.76 8.28 8.27 8.27 8.26 8.25 8.25 7.40 7.38 7.38 7.36 7.26 4.94 4.93 4.92 4.81 4.80 4.79 4.41 4.40 4.39 4.37 4.36 2.57 2.55 2.54 2.53 2.52 2.40 2.37 2.34 2.33 2.31 2.30 2.03 2.01 2.00 1.99 1.97 1.95 1.93 1.92 1.91 1.89 1.87 1.87 1.85 1.85 1.83 1.82 1.81 1.79 1.78 1.77 1.76
^{1}H NMR, 400 MHz, $CDCl_3$
F
N
O
CF_3
O
31
0.98 0.99 0.98 0.99 1.00 2.03 2.08 4.25
2.0 11.0 10.0 9.0 8.0 7.0 6.0 5.0 4.0 3.0 2.0 1.0 0.0 -1.0
f1 (ppm)

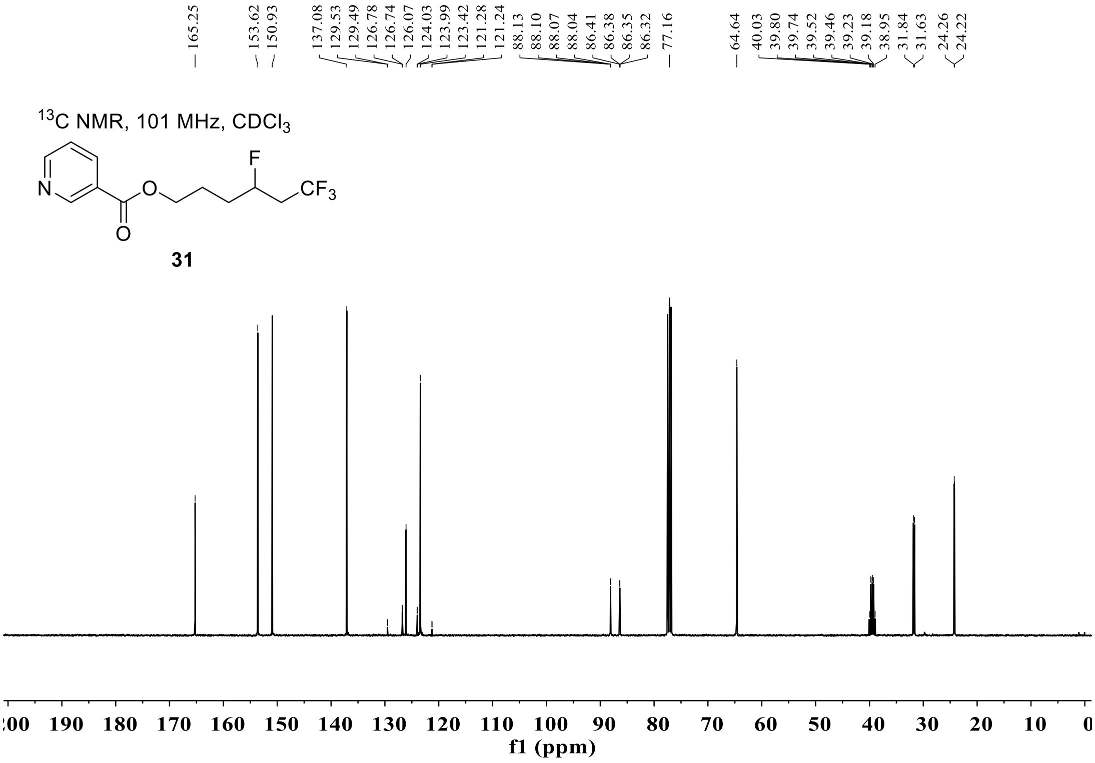
165.25
153.62 150.93
137.08 129.53 129.49 126.78 126.74 126.07 124.03 123.99 123.42 121.28 121.24
88.13 88.10 88.07 88.04 86.41 86.38 86.35 86.32
77.16
64.64
40.03 39.80 39.74 39.52 39.46 39.23 39.18 38.95
31.84 31.63
24.26 24.22
^{13}C NMR, 101 MHz, $CDCl_3$
F
N
O
CF_3
O
31
200 190 180 170 160 150 140 130 120 110 100 90 80 70 60 50 40 30 20 10 0
f1 (ppm)

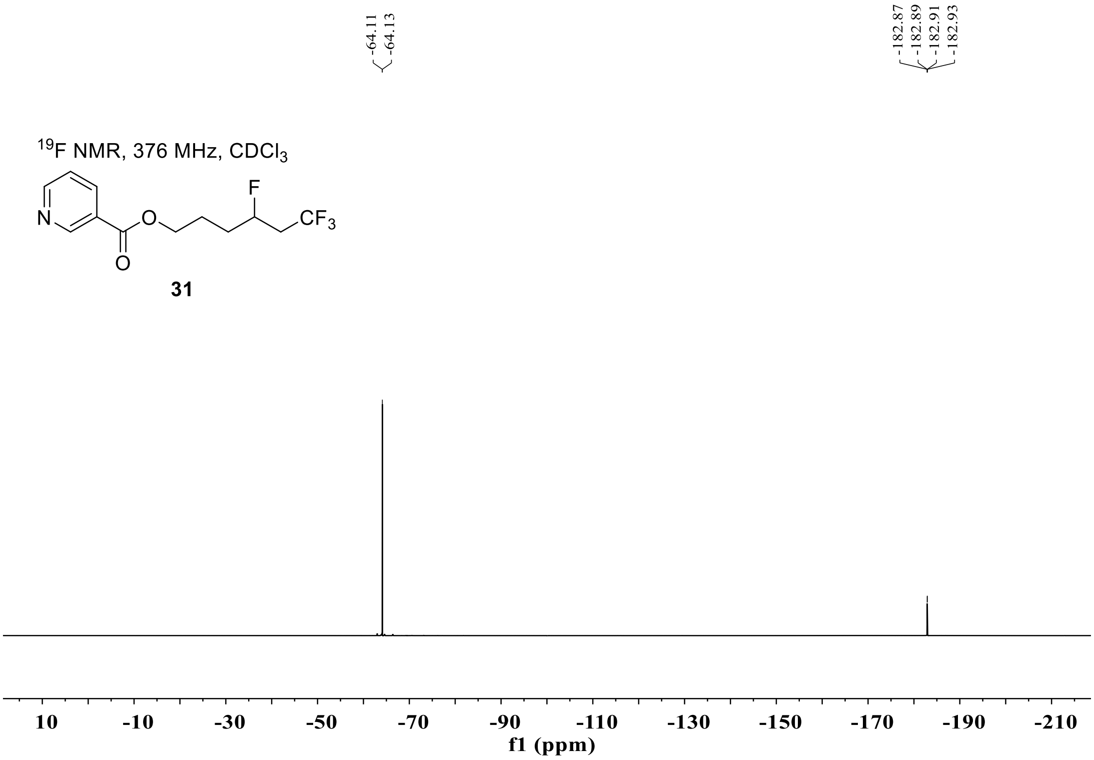

-64.11
-64.13
-182.87
-182.89
-182.91
-182.93
^{19}F NMR, 376 MHz, CDCl$_3$
F
N
O
CF$_3$
O
31
10 -10 -30 -50 -70 -90 -110 -130 -150 -170 -190 -210
f1 (ppm)

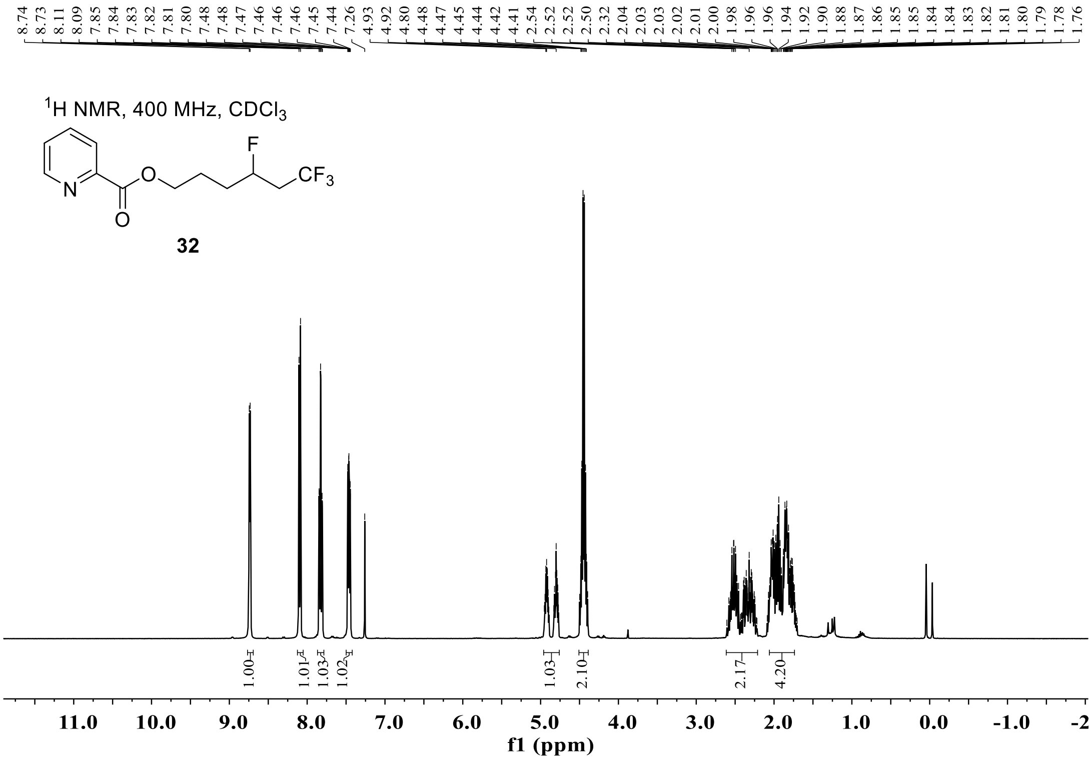

8.74 8.73 8.11 8.09 7.85 7.84 7.83 7.82 7.81 7.80 7.48 7.48 7.47 7.46 7.46 7.45 7.44 7.26 4.93 4.92 4.80 4.48 4.47 4.45 4.44 4.42 4.41 2.54 2.52 2.52 2.50 2.32 2.04 2.03 2.03 2.02 2.01 2.00 1.98 1.96 1.96 1.94 1.92 1.90 1.88 1.87 1.86 1.85 1.85 1.84 1.84 1.83 1.82 1.81 1.80 1.79 1.78 1.76
^{1}H NMR, 400 MHz, CDCl$_3$
F
N
O
CF$_3$
O
32
1.00
1.01
1.03
1.02
1.03
2.10
2.17
4.20
11.0 10.0 9.0 8.0 7.0 6.0 5.0 4.0 3.0 2.0 1.0 0.0 -1.0 -2
f1 (ppm)

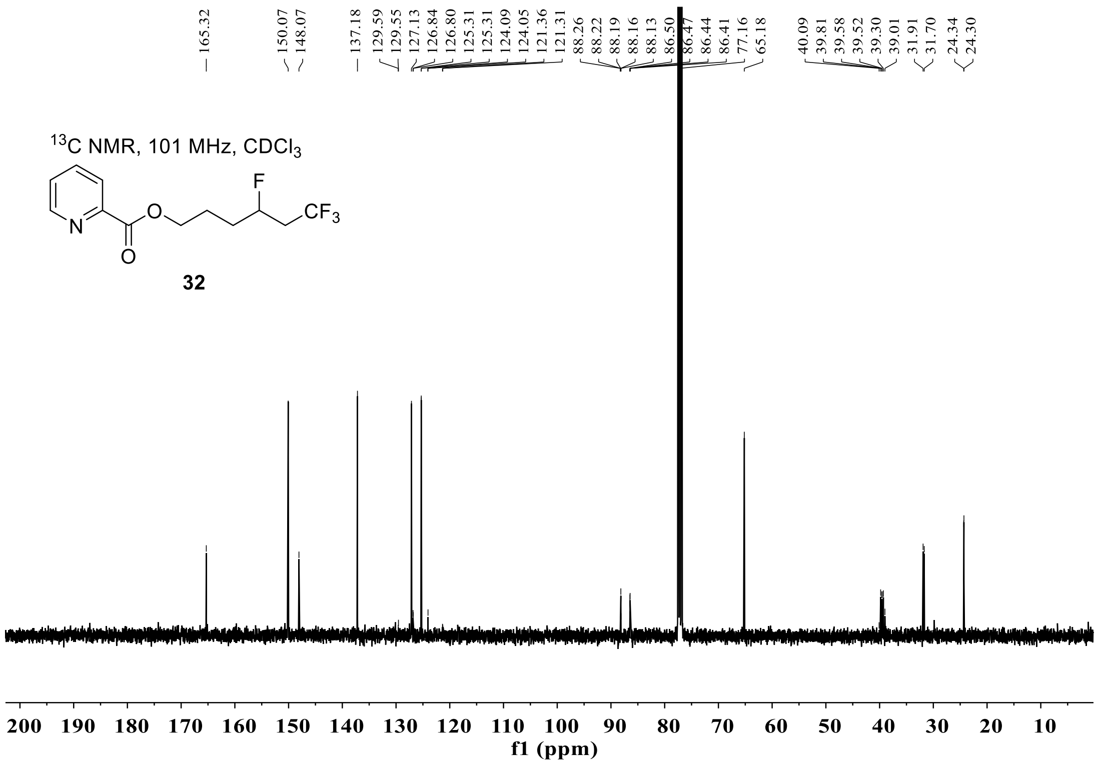

13C NMR, 101 MHz, CDCl3
F
CF3
N
O
O
32
f1 (ppm)

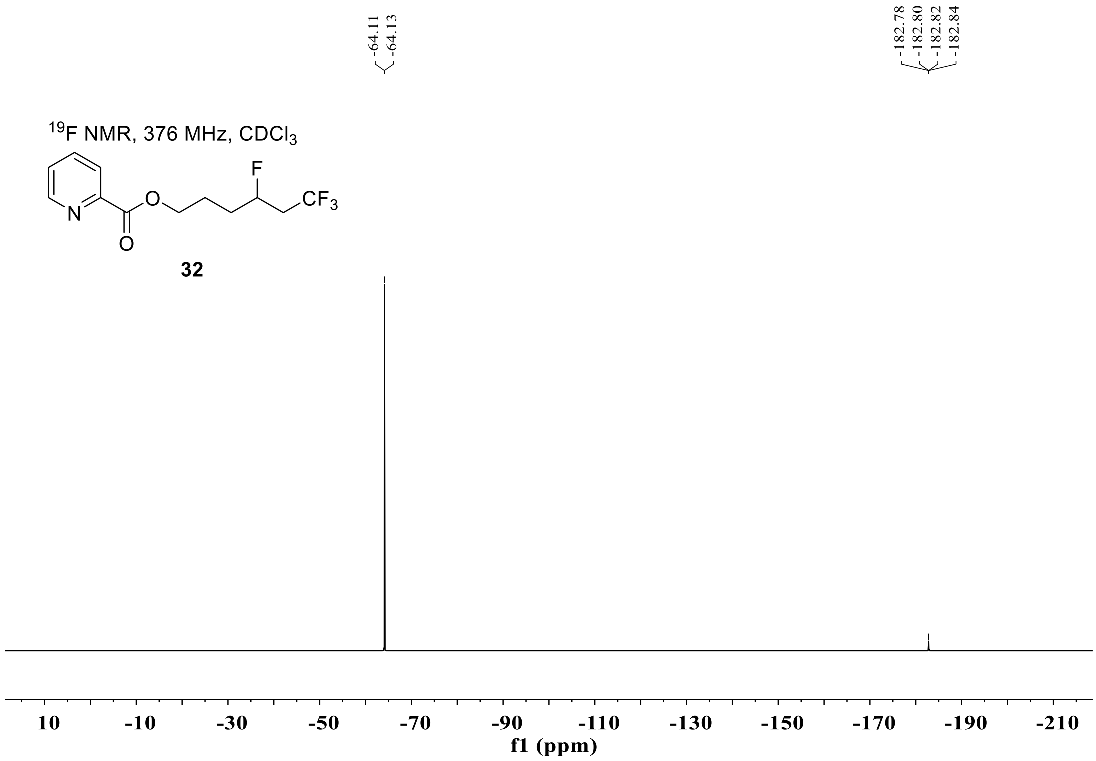

19F NMR, 376 MHz, CDCl3
F
CF3
N
O
O
32
-64.11
-64.13
-182.78
-182.80
-182.82
-182.84
f1 (ppm)

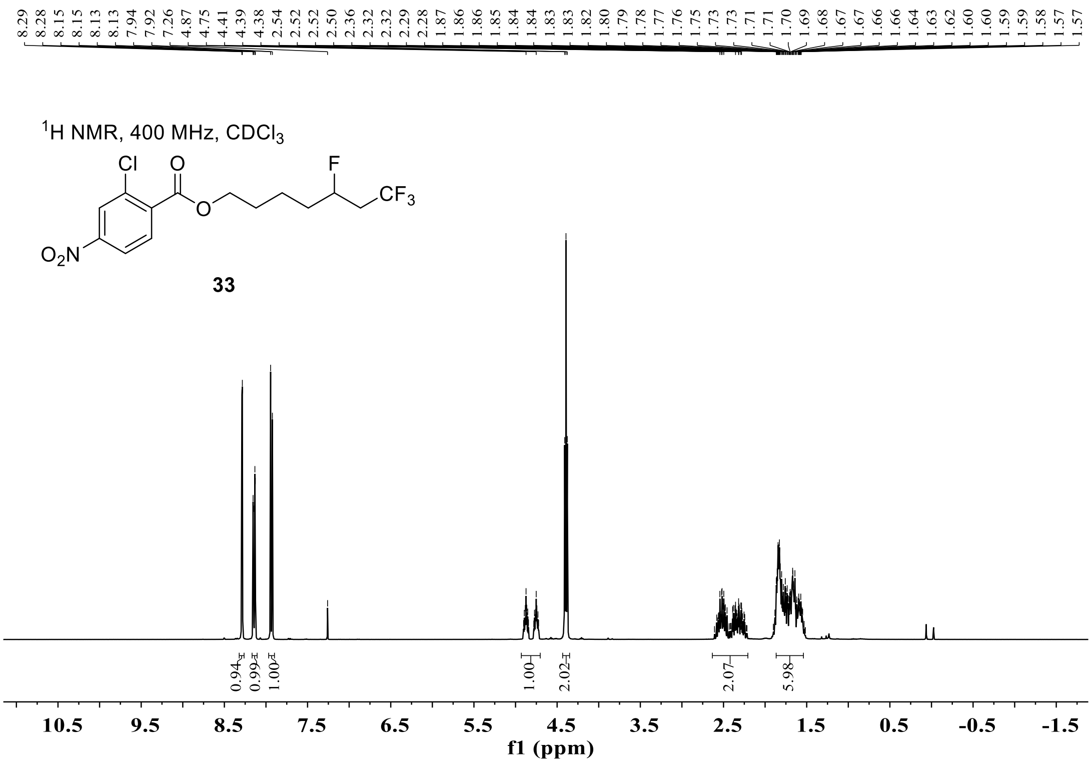

^{1}H NMR, 400 MHz, CDCl$_3$
33
f1 (ppm)

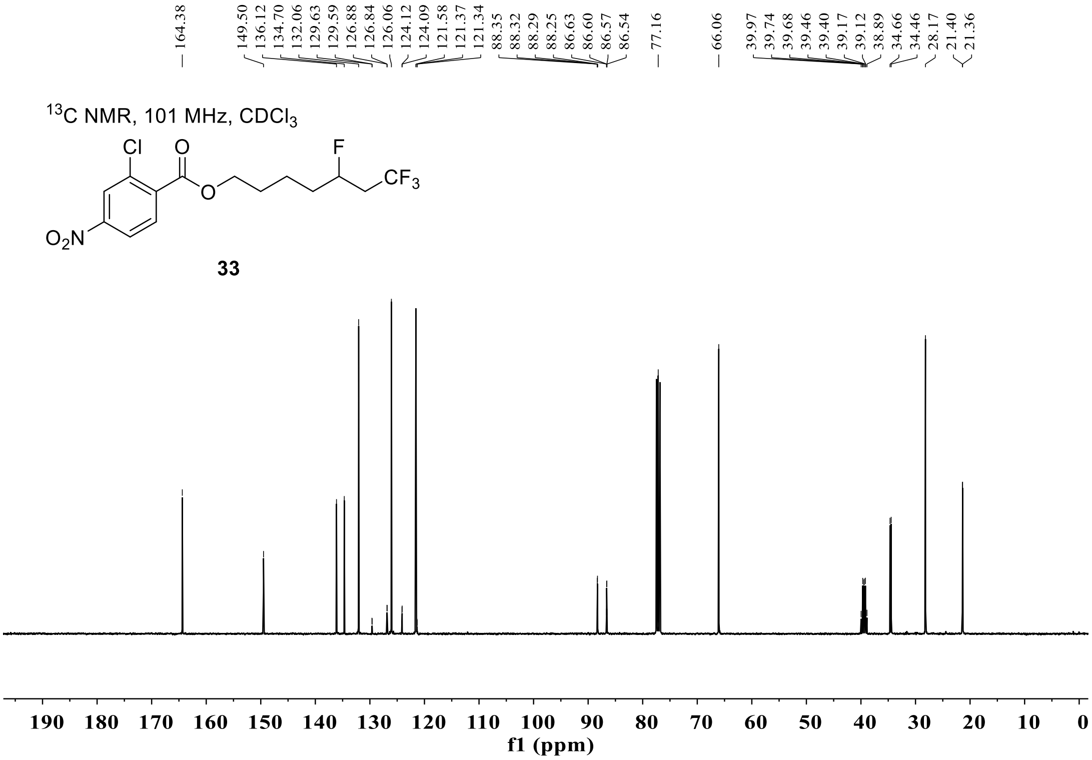

^{13}C NMR, 101 MHz, CDCl$_3$
33
f1 (ppm)

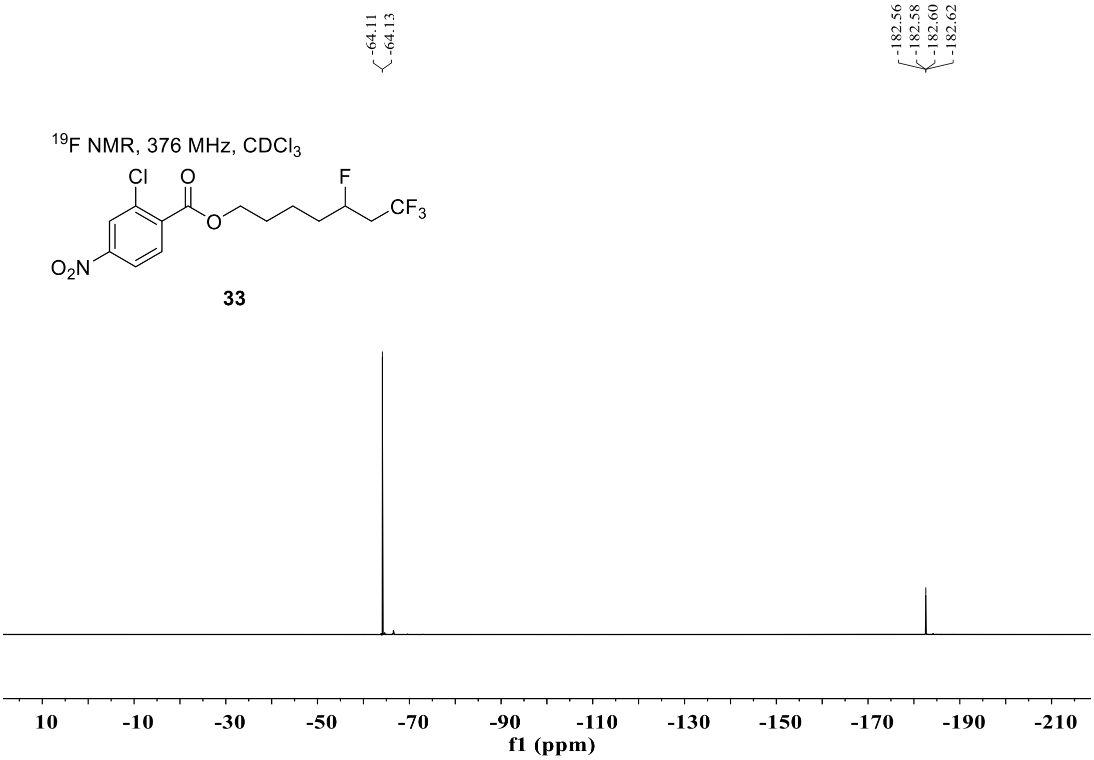
-64.11
-64.13
-182.56
-182.58
-182.60
-182.62
19F NMR, 376 MHz, CDCl3
Cl
O
F
O
CF3
O2N
33
10 -10 -30 -50 -70 -90 -110 -130 -150 -170 -190 -210
f1 (ppm)

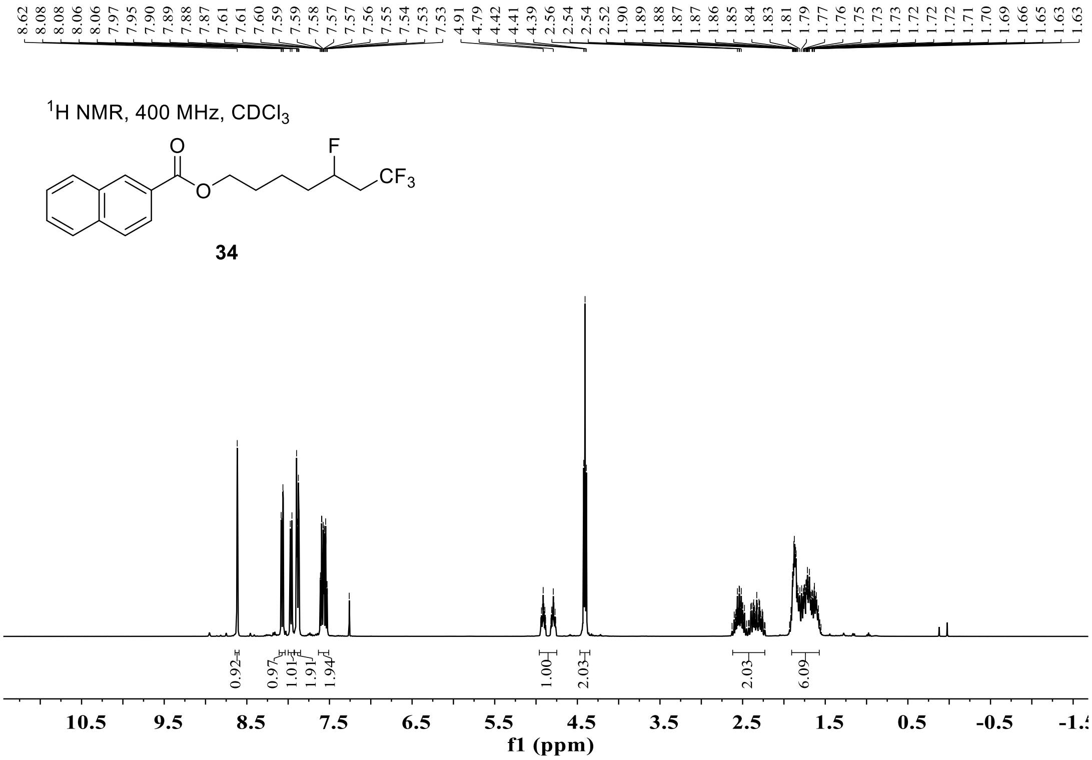
8.62 8.08 8.08 8.06 8.06 7.97 7.95 7.90 7.89 7.88 7.87 7.61 7.61 7.60 7.59 7.59 7.58 7.57 7.57 7.56 7.55 7.54 7.53 7.53 4.91 4.79 4.42 4.41 4.39 2.56 2.54 2.54 2.52 1.90 1.89 1.88 1.87 1.87 1.86 1.85 1.84 1.83 1.81 1.79 1.77 1.76 1.75 1.73 1.73 1.72 1.72 1.72 1.71 1.70 1.69 1.66 1.65 1.63 1.63
1H NMR, 400 MHz, CDCl3
O
F
O
CF3
34
0.92
0.97
1.01
1.91
1.94
1.00
2.03
2.03
6.09
10.5 9.5 8.5 7.5 6.5 5.5 4.5 3.5 2.5 1.5 0.5 -0.5
f1 (ppm)

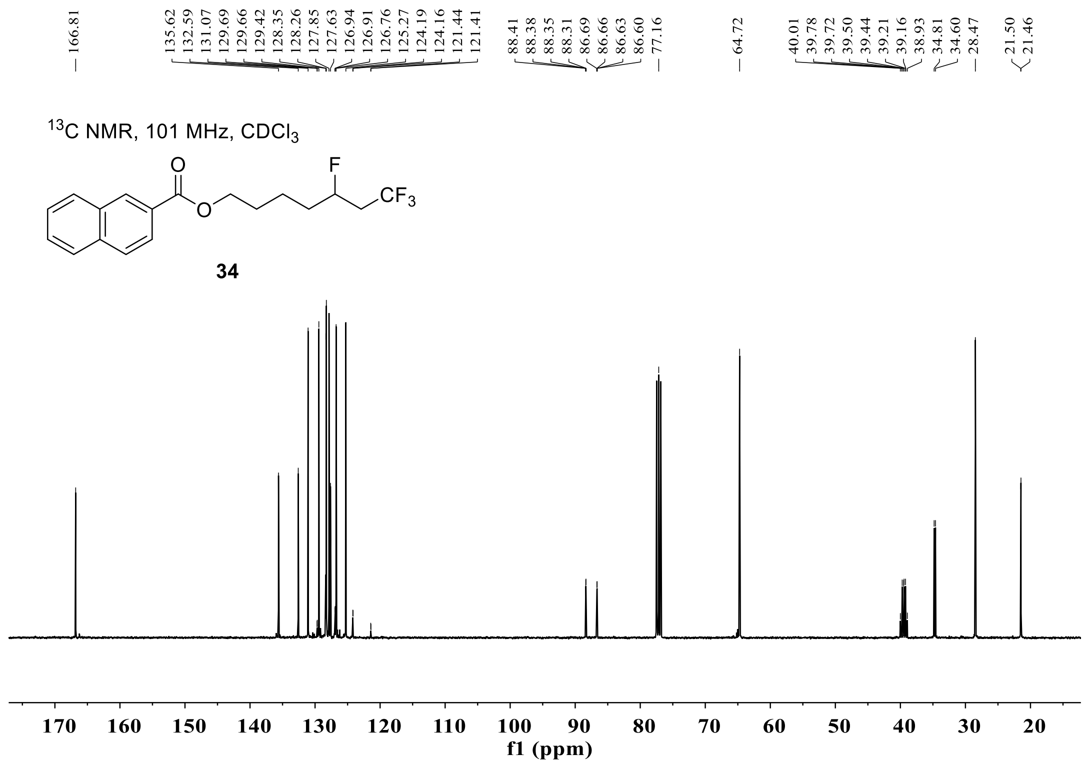
166.81
135.62
132.59
131.07
129.69
129.66
129.42
128.35
128.26
127.85
127.63
126.94
126.91
126.76
125.27
124.19
124.16
121.44
121.41
88.41
88.38
88.35
88.31
86.69
86.66
86.63
86.60
77.16
64.72
40.01
39.78
39.72
39.50
39.44
39.21
39.16
38.93
34.81
34.60
28.47
21.50
21.46
13C NMR, 101 MHz, CDCl3
O
F
O
CF3
34
170 160 150 140 130 120 110 100 90 80 70 60 50 40 30 20
f1 (ppm)

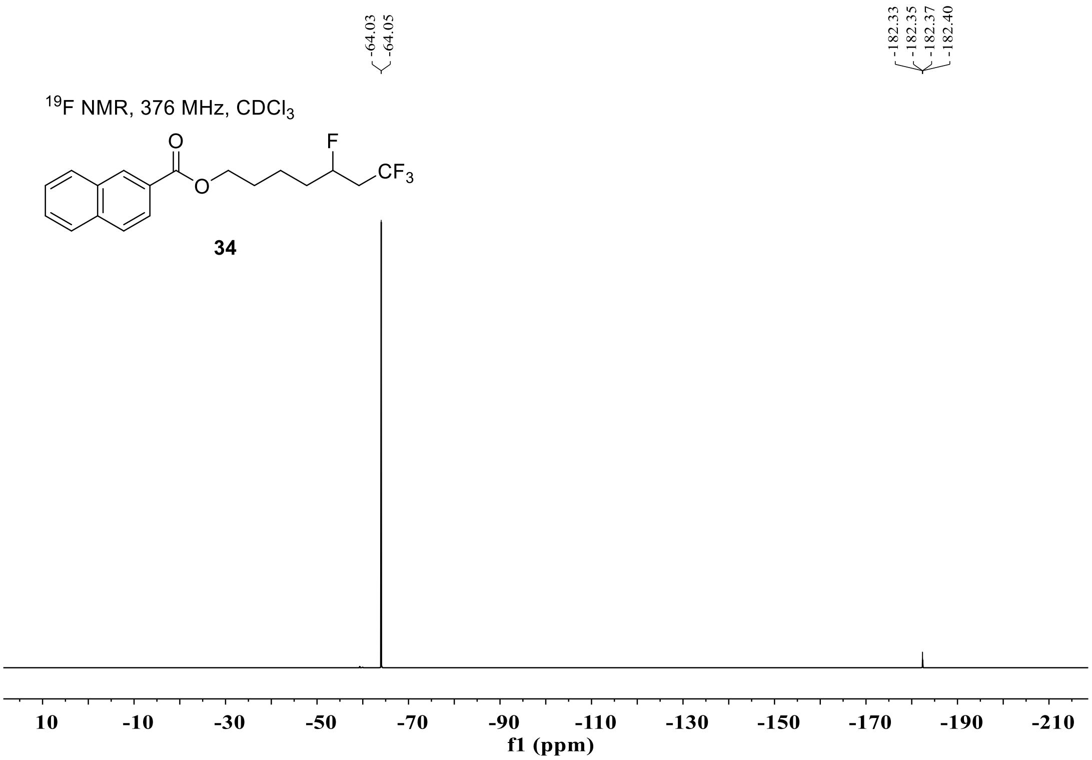
-64.03
-64.05
-182.33
-182.35
-182.37
-182.40
19F NMR, 376 MHz, CDCl3
O
F
O
CF3
34
10 -10 -30 -50 -70 -90 -110 -130 -150 -170 -190 -210
f1 (ppm)

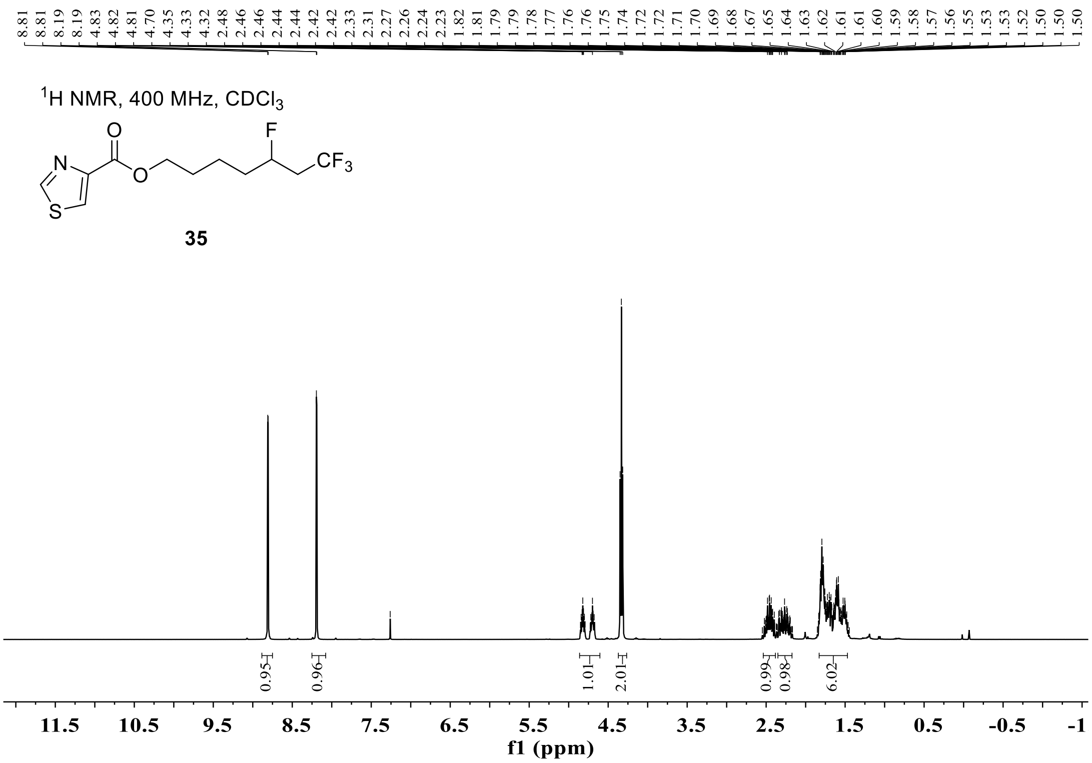
8.81 8.81 8.19 8.19 4.83 4.82 4.81 4.70 4.35 4.33 4.32 2.48 2.46 2.46 2.44 2.44 2.42 2.42 2.33 2.31 2.27 2.26 2.24 2.23 1.82 1.81 1.79 1.79 1.78 1.77 1.76 1.76 1.75 1.74 1.72 1.72 1.71 1.70 1.69 1.68 1.67 1.65 1.64 1.63 1.62 1.61 1.61 1.60 1.59 1.58 1.57 1.56 1.55 1.53 1.53 1.52 1.50 1.50 1.50
^{1}H NMR, 400 MHz, CDCl$_3$
O
F
N
O
CF$_3$
S
35
0.95
0.96
1.01
2.01
0.99
0.98
6.02
11.5 10.5 9.5 8.5 7.5 6.5 5.5 4.5 3.5 2.5 1.5 0.5 -0.5 -1
f1 (ppm)

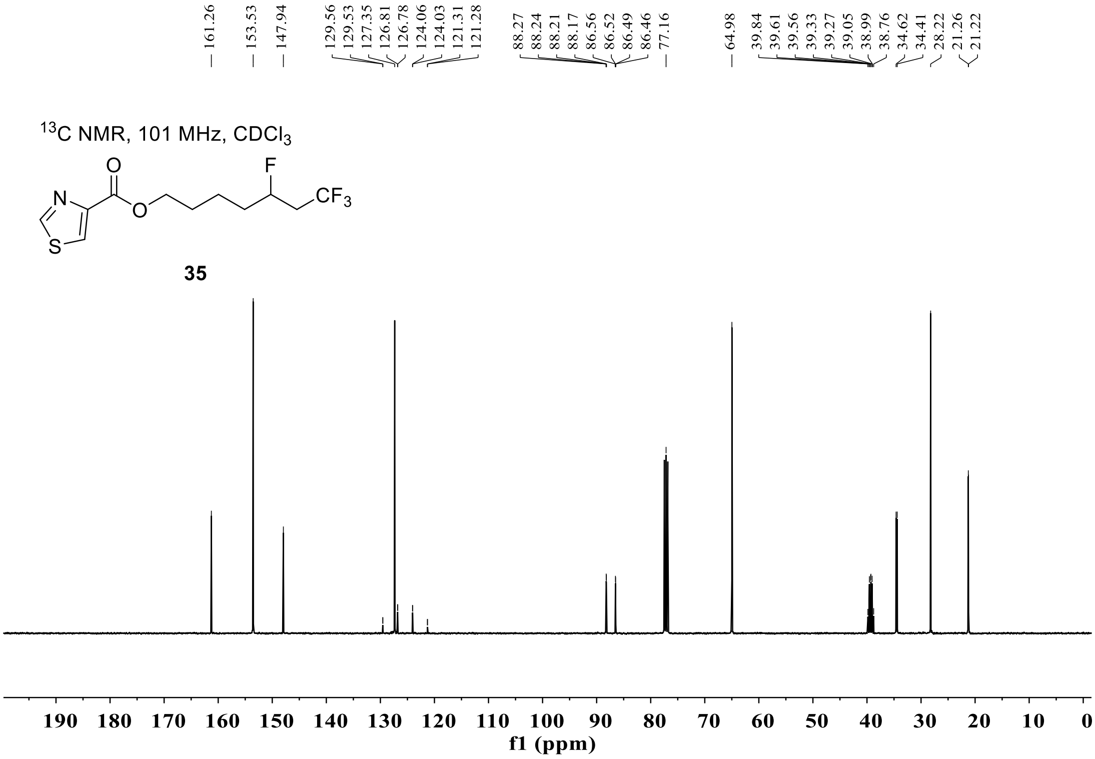
161.26
153.53
147.94
129.56 129.53 127.35 126.81 126.78 124.06 124.03 121.31 121.28
88.27 88.24 88.21 88.17 86.56 86.52 86.49 86.46 77.16
64.98
39.84 39.61 39.56 39.33 39.27 39.05 38.99 38.76 34.62 34.41 28.22 21.26 21.22
^{13}C NMR, 101 MHz, CDCl$_3$
O
F
N
O
CF$_3$
S
35
190 180 170 160 150 140 130 120 110 100 90 80 70 60 50 40 30 20 10 0
f1 (ppm)

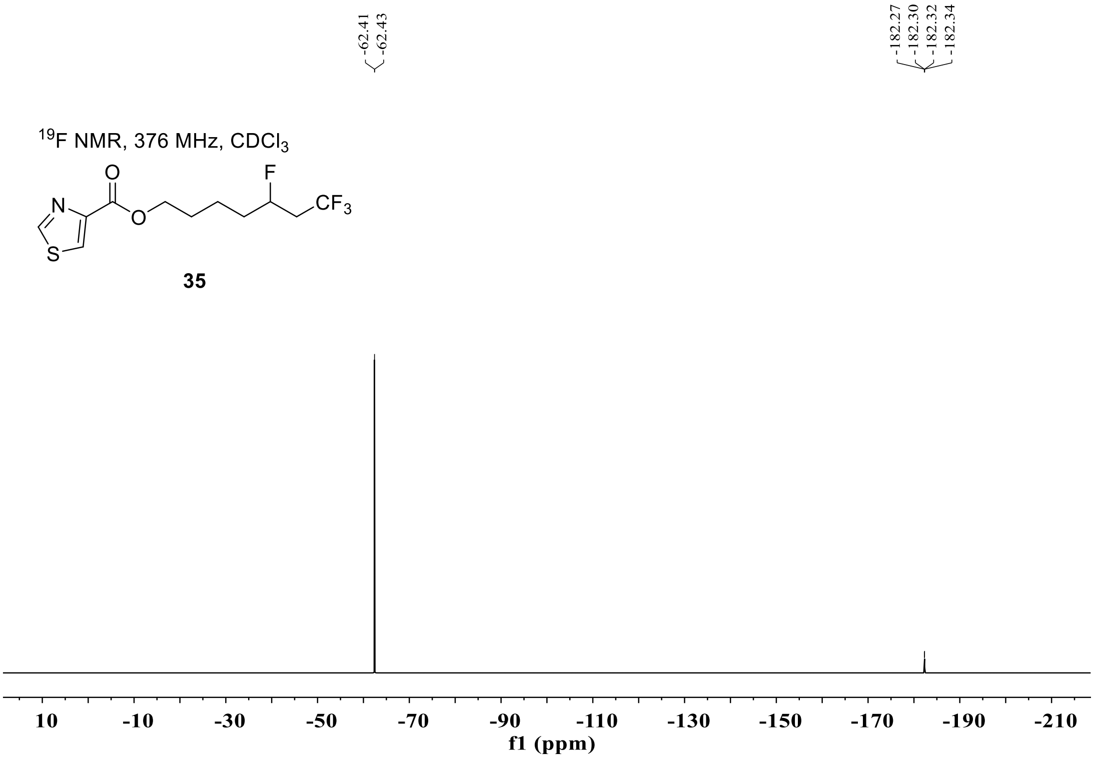
-62.41
-62.43
-182.27
-182.30
-182.32
-182.34
19F NMR, 376 MHz, CDCl3
35
10 -10 -30 -50 -70 -90 -110 -130 -150 -170 -190 -210
f1 (ppm)

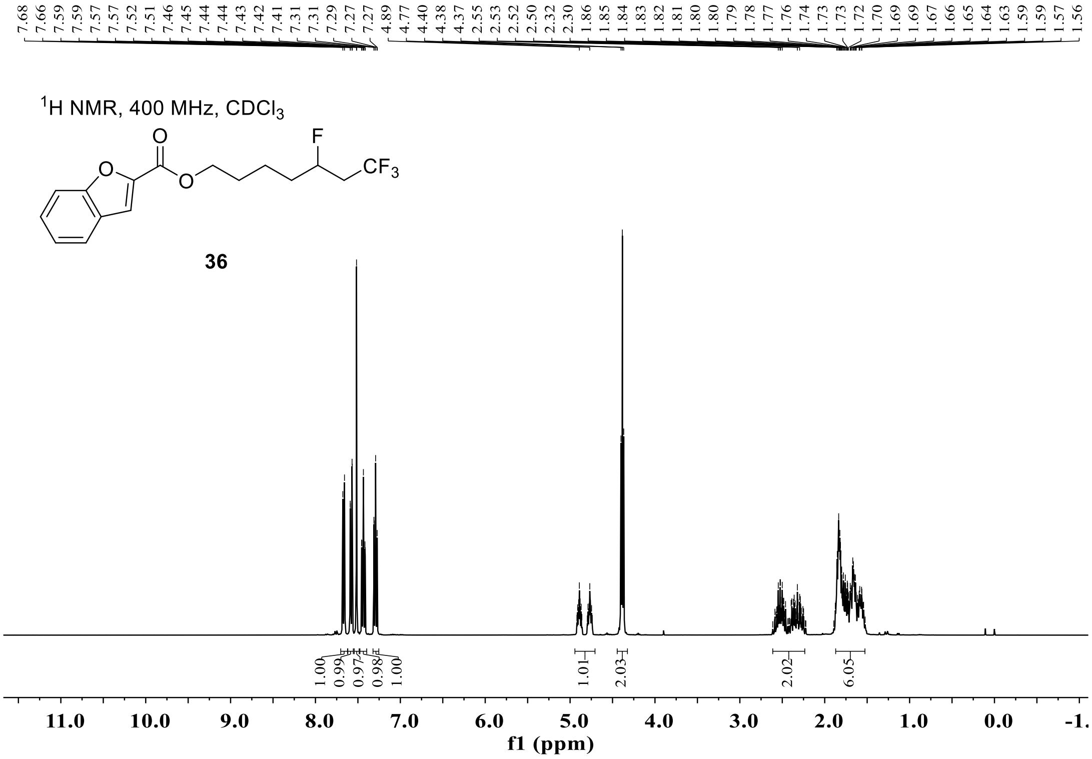
7.68 7.66 7.59 7.59 7.57 7.57 7.52 7.51 7.46 7.45 7.44 7.44 7.43 7.42 7.41 7.31 7.31 7.29 7.27 7.27 4.89 4.77 4.40 4.38 4.37 2.55 2.53 2.52 2.50 2.32 2.30 1.86 1.85 1.84 1.83 1.82 1.81 1.80 1.80 1.79 1.78 1.77 1.76 1.74 1.73 1.73 1.72 1.70 1.69 1.67 1.66 1.65 1.64 1.63 1.59 1.59 1.57 1.56
1H NMR, 400 MHz, CDCl3
36
1.00 0.99 0.97 0.98 1.00 1.01 2.03 2.02 6.05
11.0 10.0 9.0 8.0 7.0 6.0 5.0 4.0 3.0 2.0 1.0 0.0 -1.
f1 (ppm)

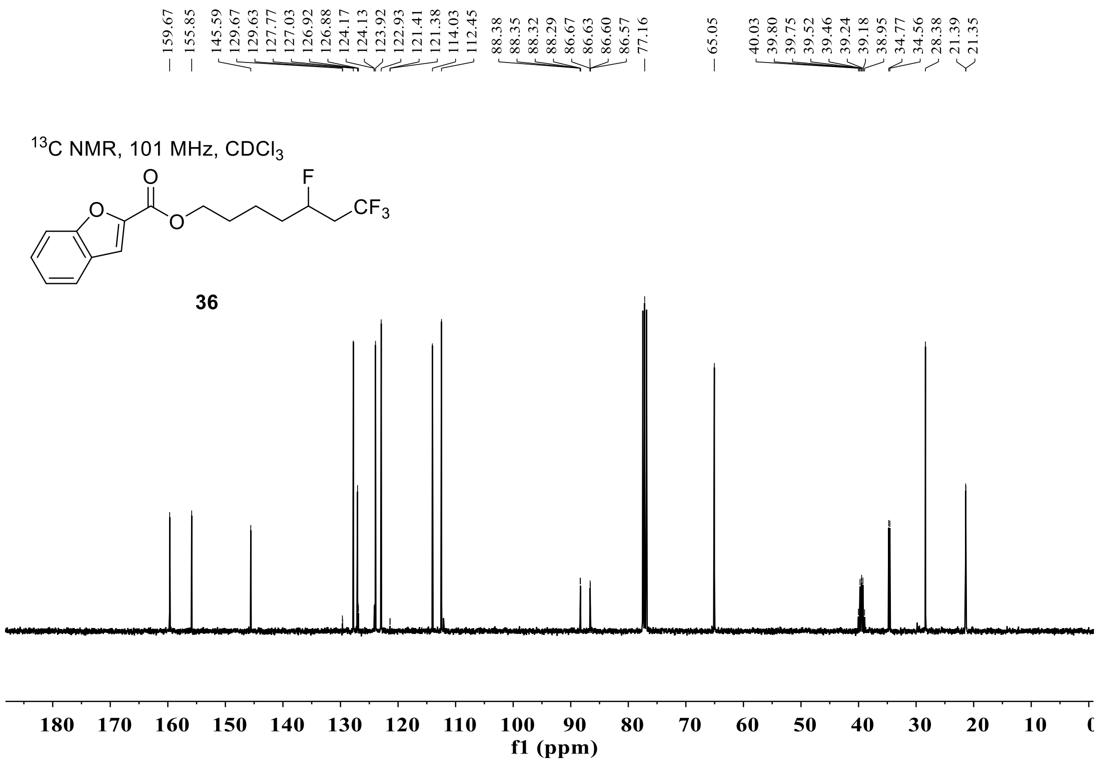
159.67
155.85
145.59
129.67
129.63
127.77
127.03
126.92
126.88
124.17
124.13
123.92
122.93
121.41
121.38
114.03
112.45
88.38
88.35
88.32
88.29
86.67
86.63
86.60
86.57
77.16
65.05
40.03
39.80
39.75
39.52
39.46
39.24
39.18
38.95
34.77
34.56
28.38
21.39
21.35
13C NMR, 101 MHz, CDCl3
36
f1 (ppm)

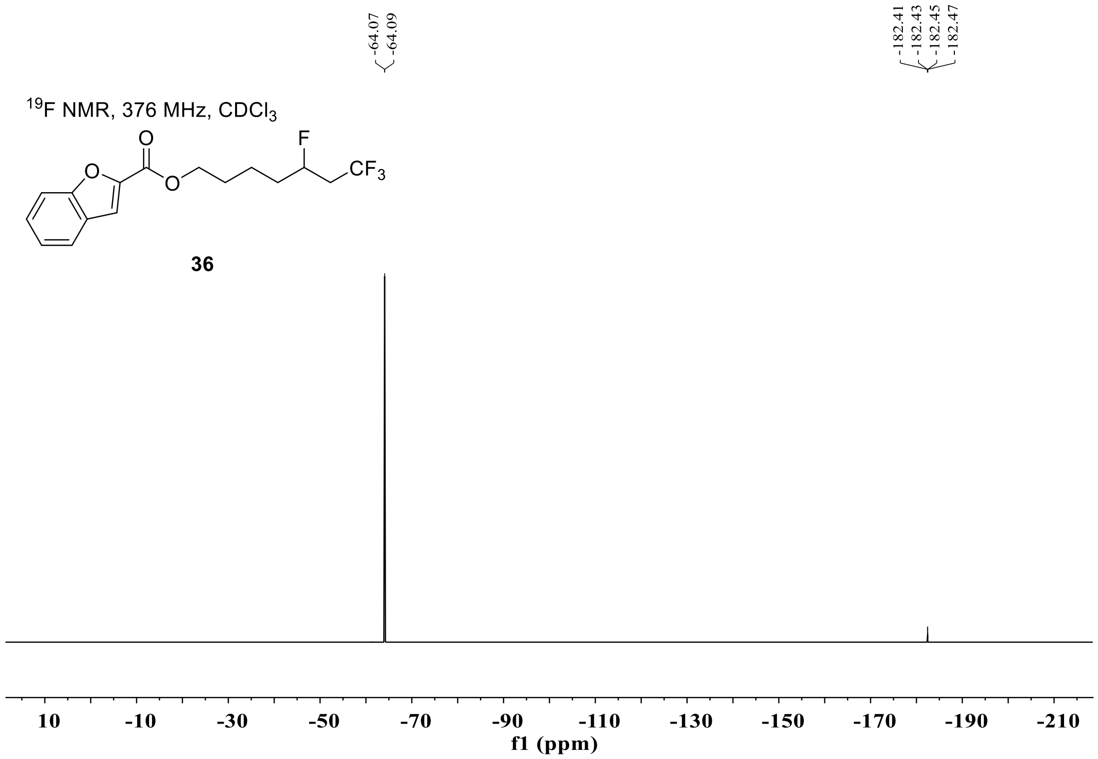
-64.07
-64.09
-182.41
-182.43
-182.45
-182.47
19F NMR, 376 MHz, CDCl3
36
f1 (ppm)

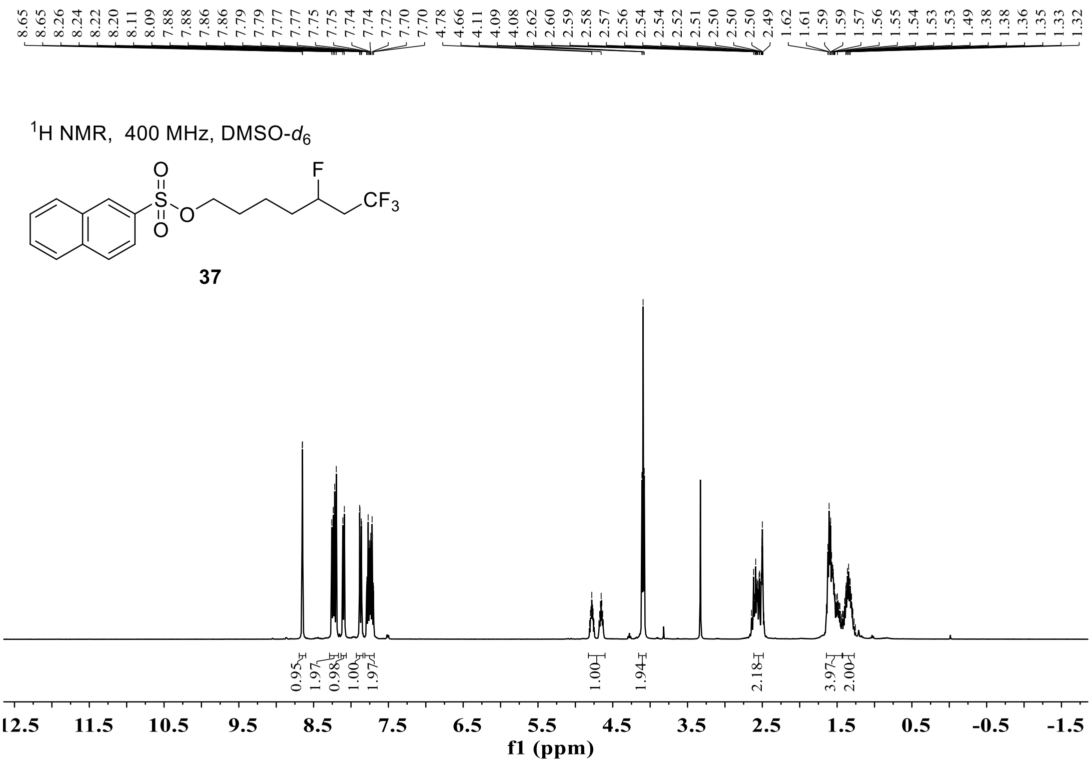
8.65 8.65 8.26 8.24 8.22 8.20 8.11 8.09 7.88 7.88 7.86 7.86 7.79 7.79 7.77 7.77 7.75 7.75 7.74 7.74 7.72 7.70 7.70 4.78 4.66 4.11 4.09 4.08 2.62 2.60 2.59 2.58 2.57 2.56 2.54 2.54 2.52 2.51 2.50 2.50 2.50 2.49 1.62 1.61 1.59 1.59 1.57 1.56 1.55 1.54 1.53 1.53 1.49 1.38 1.38 1.36 1.35 1.33 1.32
^{1}H NMR, 400 MHz, DMSO-d_6
37
0.95 1.97 0.98 1.00 1.97 1.00 1.94 2.18 3.97 2.00
12.5 11.5 10.5 9.5 8.5 7.5 6.5 5.5 4.5 3.5 2.5 1.5 0.5 -0.5 -1.5
f1 (ppm)

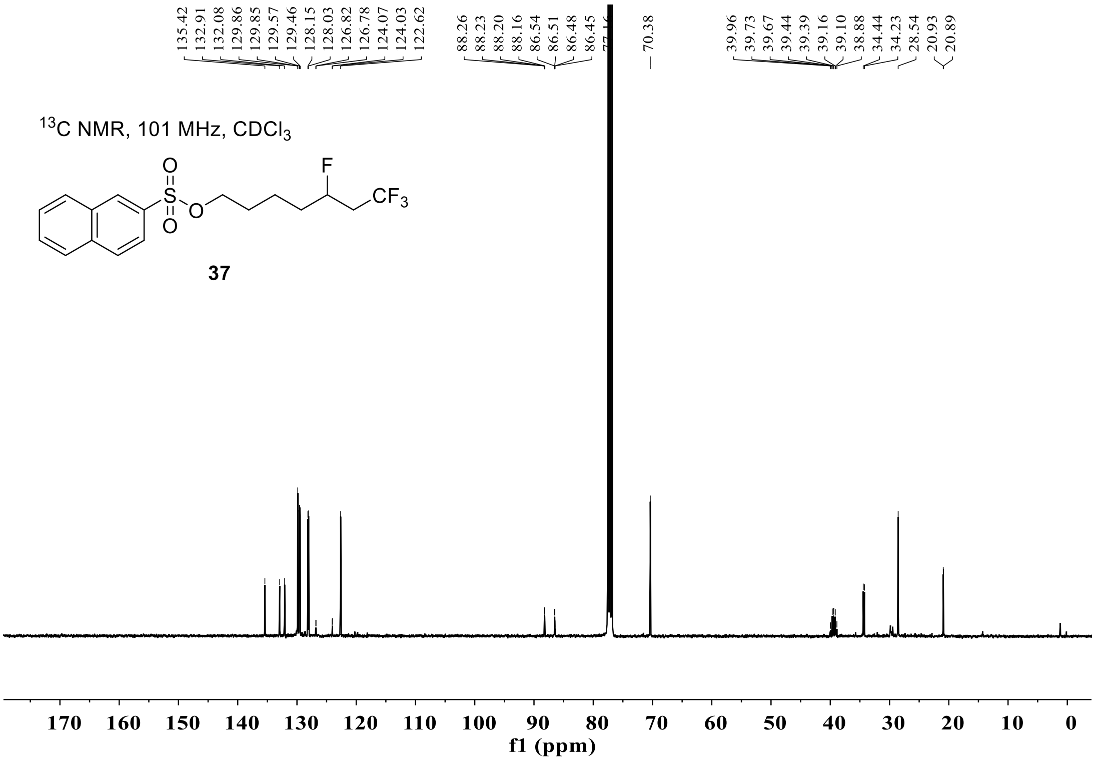
135.42 132.91 132.08 129.86 129.85 129.57 129.46 128.15 128.03 126.82 126.78 124.07 124.03 122.62 88.26 88.23 88.20 88.16 86.54 86.51 86.48 86.45 70.38 39.96 39.73 39.67 39.44 39.39 39.16 39.10 38.88 34.44 34.23 28.54 20.93 20.89
^{13}C NMR, 101 MHz, $CDCl_3$
37
170 160 150 140 130 120 110 100 90 80 70 60 50 40 30 20 10 0
f1 (ppm)

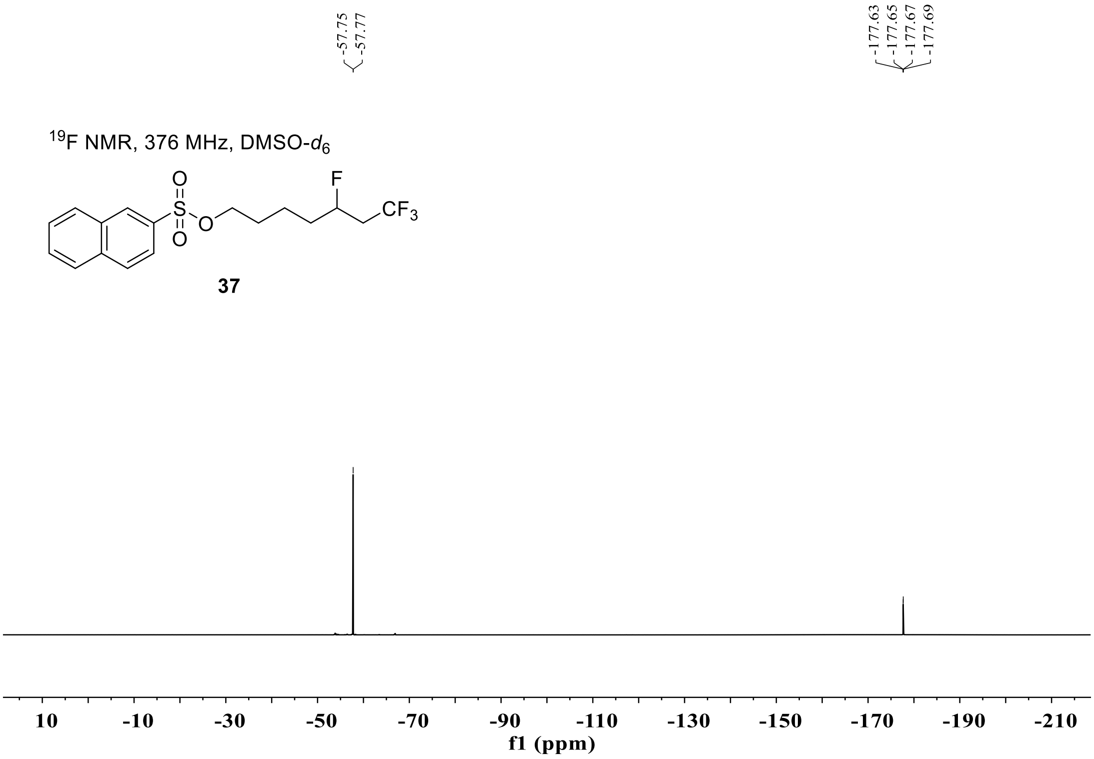
-57.75
-57.77
-177.63
-177.65
-177.67
-177.69
19F NMR, 376 MHz, DMSO-d6
O
S
O
O
F
CF3
37
10 -10 -30 -50 -70 -90 -110 -130 -150 -170 -190 -210
f1 (ppm)

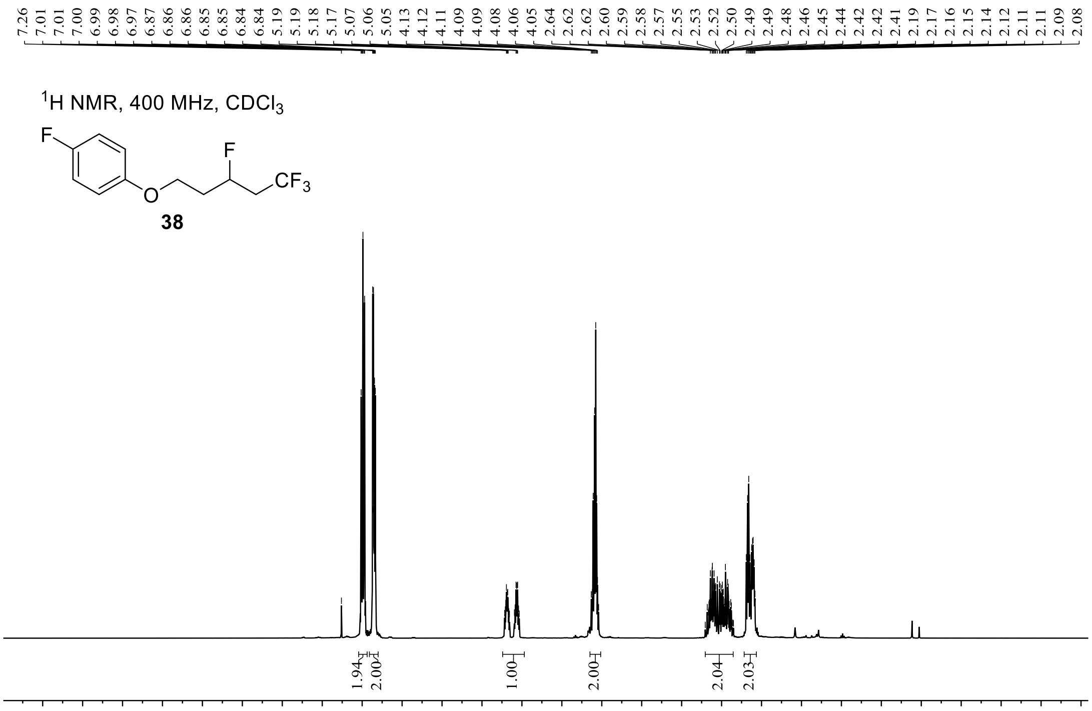
7.26 7.01 7.01 7.00 6.99 6.98 6.97 6.87 6.86 6.86 6.85 6.85 6.84 6.84 5.19 5.19 5.18 5.17 5.07 5.06 5.05 4.13 4.12 4.11 4.09 4.09 4.08 4.06 4.05 2.64 2.62 2.62 2.60 2.59 2.58 2.57 2.55 2.53 2.52 2.50 2.49 2.49 2.48 2.46 2.45 2.44 2.42 2.42 2.41 2.19 2.17 2.16 2.15 2.14 2.12 2.11 2.11 2.09 2.08
1H NMR, 400 MHz, CDCl3
F
O
F
CF3
38
1.94
2.00
1.00
2.00
2.04
2.03
11.0 10.0 9.0 8.0 7.0 6.0 5.0 4.0 3.0 2.0 1.0 0.0 -1.0 -2.
f1 (ppm)

^{13}C NMR, 101 MHz, $CDCl_3$

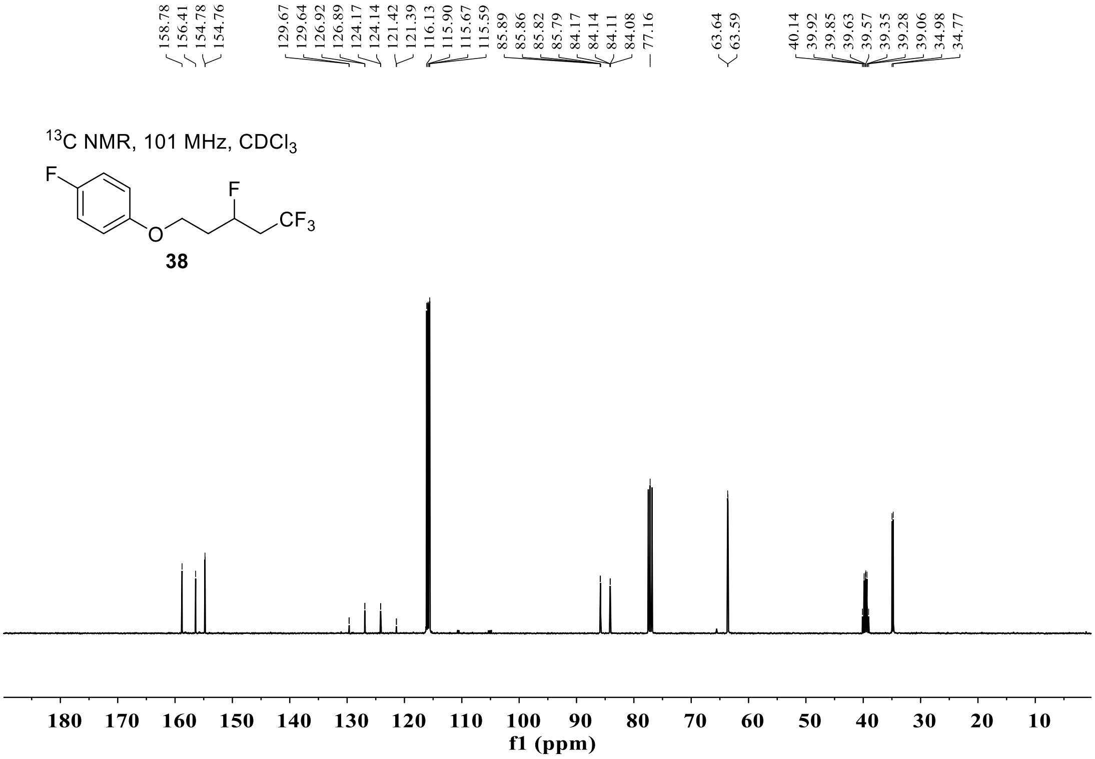

^{19}F NMR, 376 MHz, $CDCl_3$

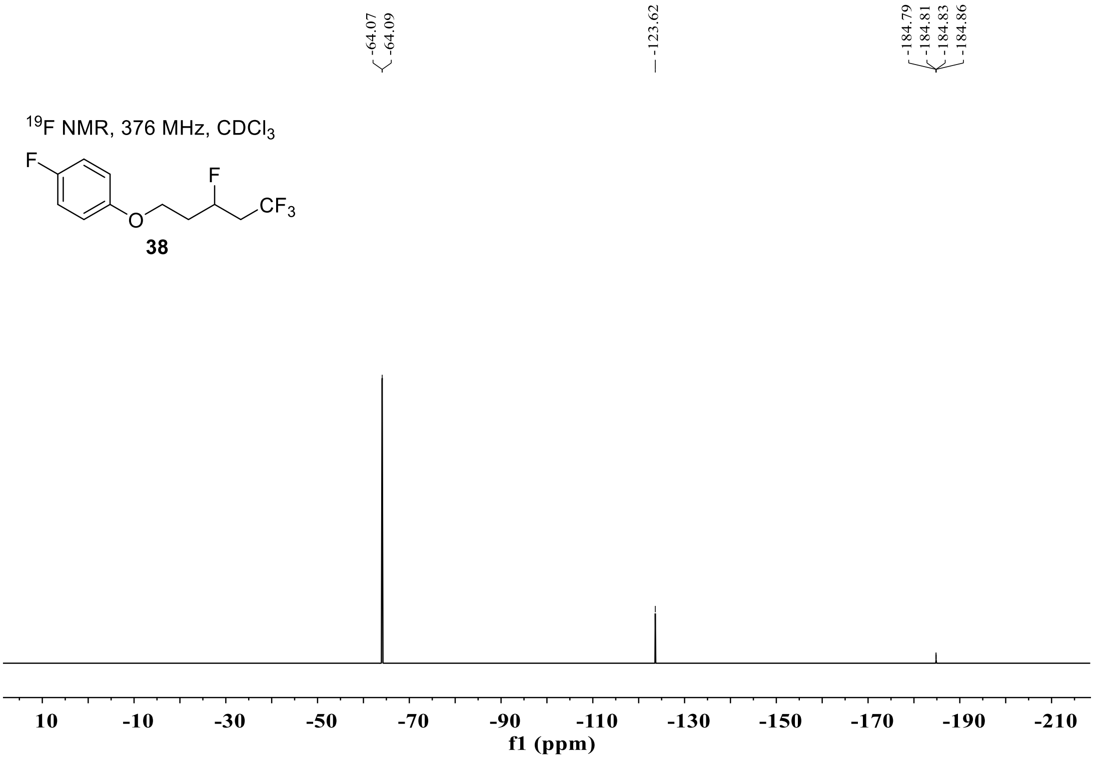

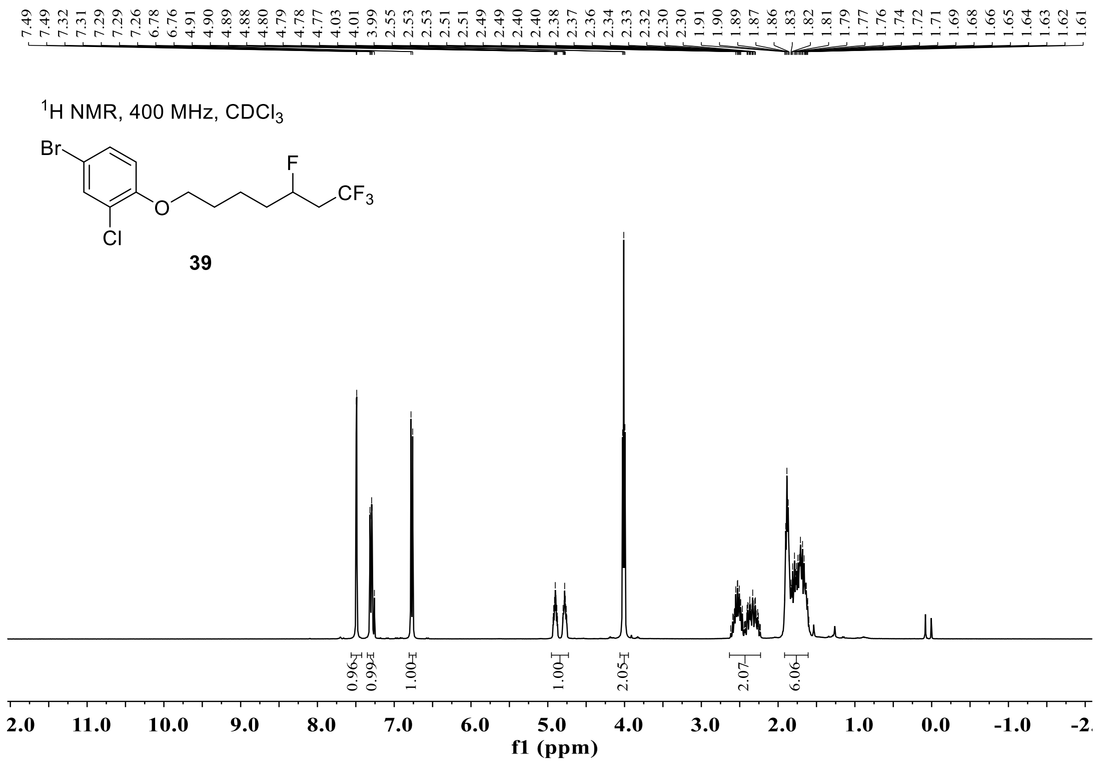

7.49 7.49 7.32 7.31 7.29 7.29 7.26 6.78 6.76 4.91 4.90 4.89 4.88 4.80 4.79 4.78 4.77 4.03 4.01 3.99 2.55 2.53 2.53 2.51 2.51 2.49 2.49 2.40 2.40 2.38 2.37 2.36 2.34 2.33 2.32 2.30 2.30 1.91 1.90 1.89 1.87 1.86 1.83 1.82 1.81 1.79 1.77 1.76 1.74 1.72 1.71 1.69 1.68 1.66 1.65 1.64 1.63 1.62 1.61
1H NMR, 400 MHz, CDCl3
Br
F
O
CF3
Cl
39
0.96
0.99
1.00
1.00
2.05
2.07
6.06
2.0 11.0 10.0 9.0 8.0 7.0 6.0 5.0 4.0 3.0 2.0 1.0 0.0 -1.0 -2.
f1 (ppm)

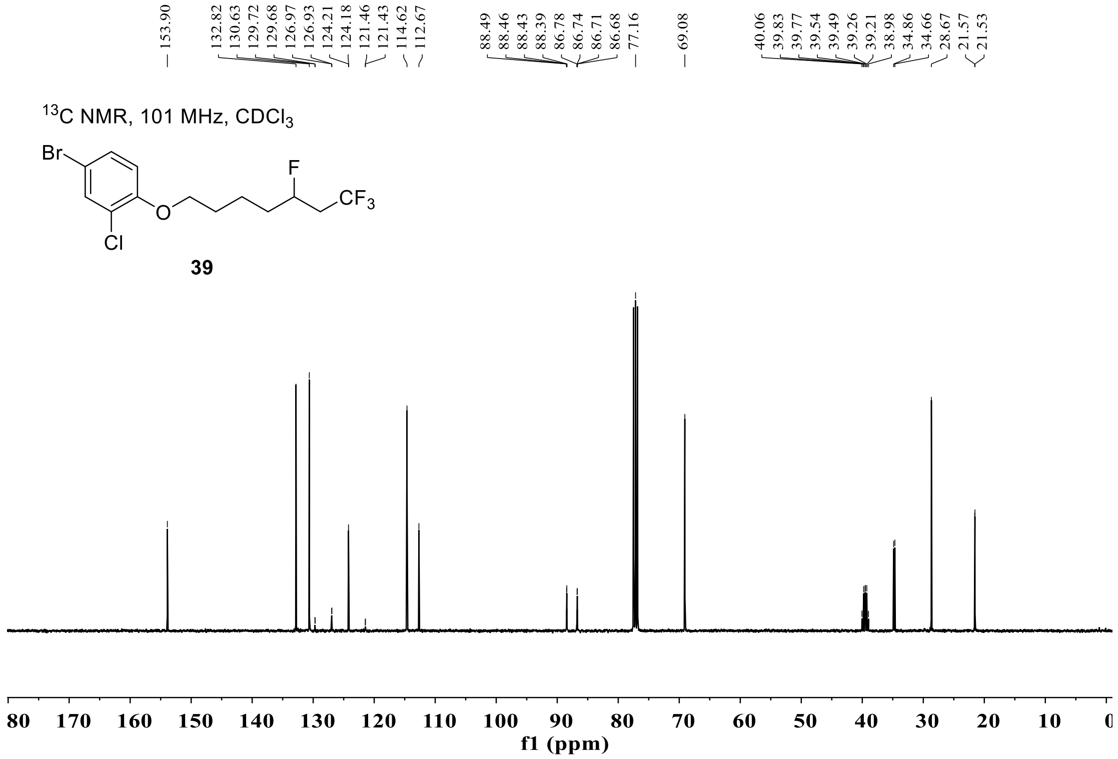

153.90 132.82 130.63 129.72 129.68 126.97 126.93 124.21 124.18 121.46 121.43 114.62 112.67 88.49 88.46 88.43 88.39 86.78 86.74 86.71 86.68 77.16 69.08 40.06 39.83 39.77 39.54 39.49 39.26 39.21 38.98 34.86 34.66 28.67 21.57 21.53
13C NMR, 101 MHz, CDCl3
Br
F
O
CF3
Cl
39
80 170 160 150 140 130 120 110 100 90 80 70 60 50 40 30 20 10 0
f1 (ppm)

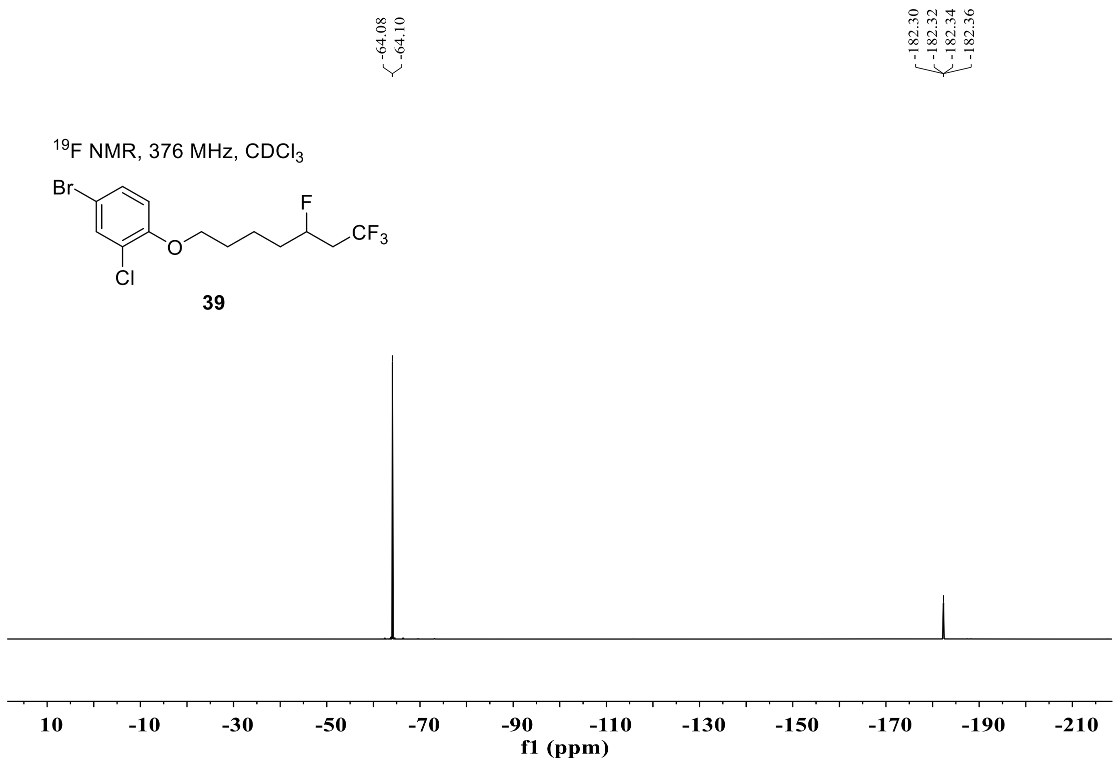

-64.08
-64.10
-182.30
-182.32
-182.34
-182.36
19F NMR, 376 MHz, CDCl3
Br
F
O
CF3
Cl
39
10 -10 -30 -50 -70 -90 -110 -130 -150 -170 -190 -210
f1 (ppm)

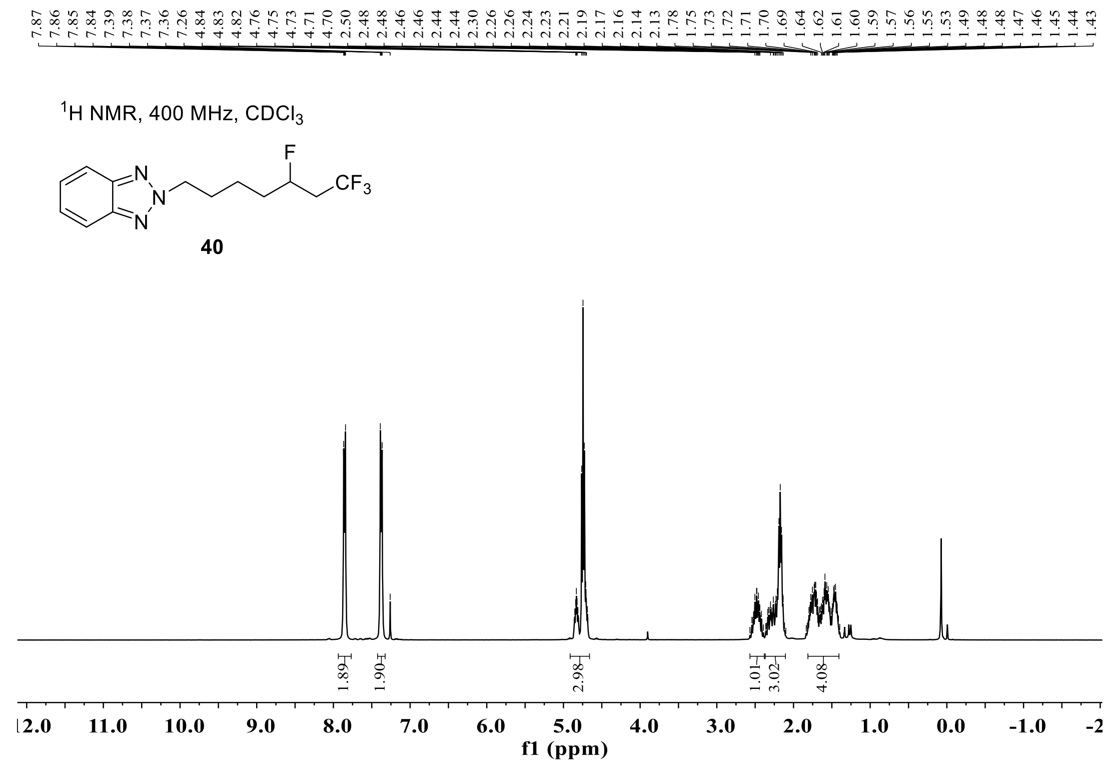

7.87 7.86 7.85 7.84 7.39 7.38 7.37 7.36 7.26 4.84 4.83 4.82 4.76 4.75 4.73 4.71 4.70 2.50 2.48 2.48 2.46 2.46 2.44 2.44 2.30 2.26 2.26 2.24 2.23 2.21 2.19 2.17 2.16 2.14 2.13 1.78 1.75 1.73 1.72 1.71 1.70 1.69 1.64 1.62 1.61 1.60 1.59 1.57 1.56 1.55 1.53 1.49 1.48 1.48 1.47 1.46 1.45 1.44 1.43
1H NMR, 400 MHz, CDCl3
N
N
N
F
CF3
40
1.89
1.90
2.98
1.01
3.02
4.08
12.0 11.0 10.0 9.0 8.0 7.0 6.0 5.0 4.0 3.0 2.0 1.0 0.0 -1.0 -2
f1 (ppm)

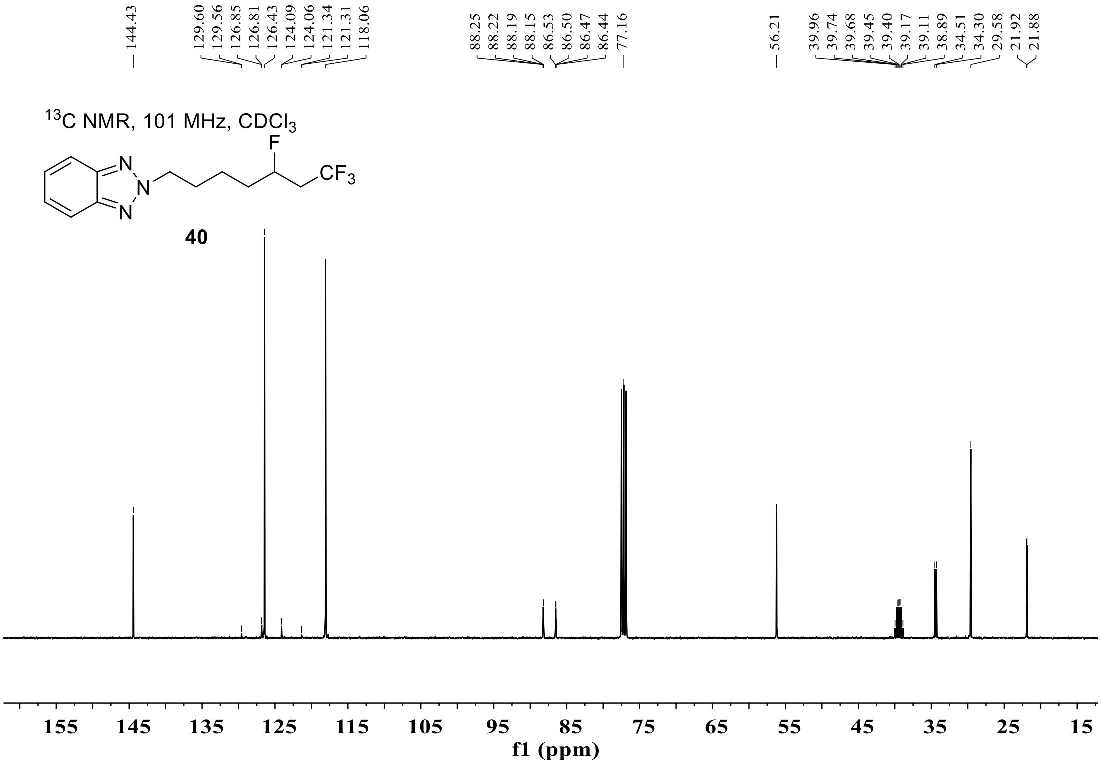
144.43
129.60
129.56
126.85
126.81
126.43
124.09
124.06
121.34
121.31
118.06
88.25
88.22
88.19
88.15
86.53
86.50
86.47
86.44
77.16
56.21
39.96
39.74
39.68
39.45
39.40
39.17
39.11
38.89
34.51
34.30
29.58
21.92
21.88
13C NMR, 101 MHz, CDCl3
F
N
N
N
CF3
40
155 145 135 125 115 105 95 85 75 65 55 45 35 25 15
f1 (ppm)

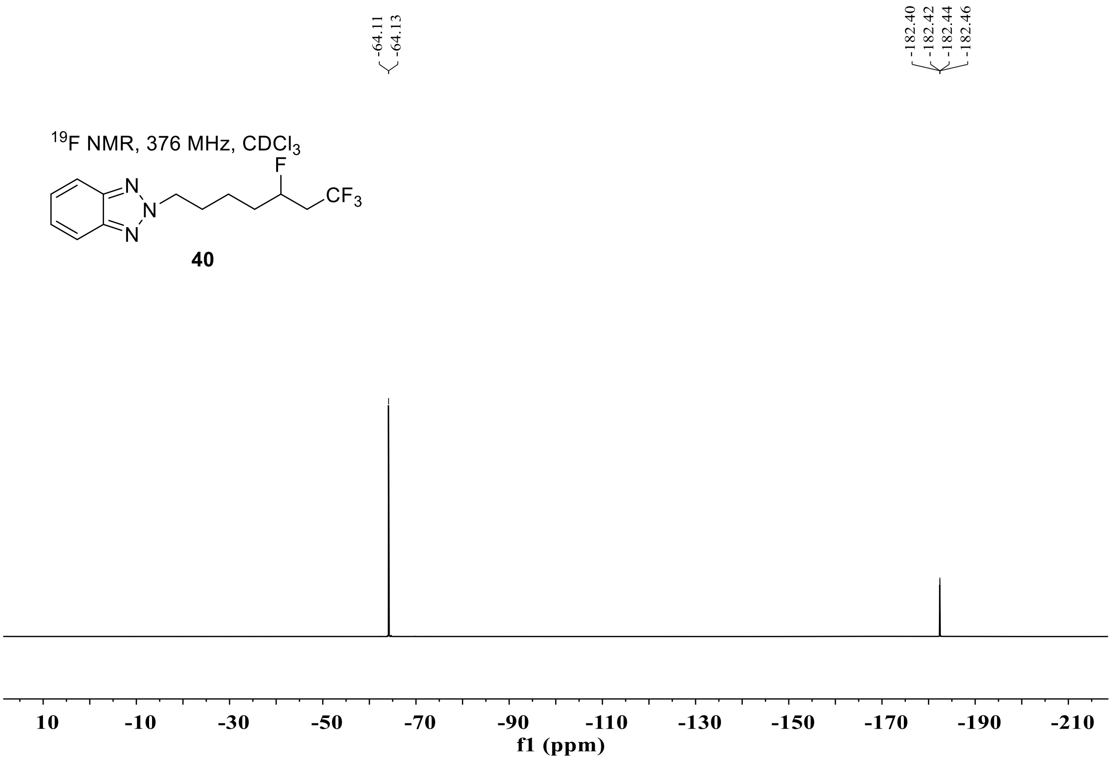
-64.11
-64.13
-182.40
-182.42
-182.44
-182.46
19F NMR, 376 MHz, CDCl3
F
N
N
N
CF3
40
10 -10 -30 -50 -70 -90 -110 -130 -150 -170 -190 -210
f1 (ppm)

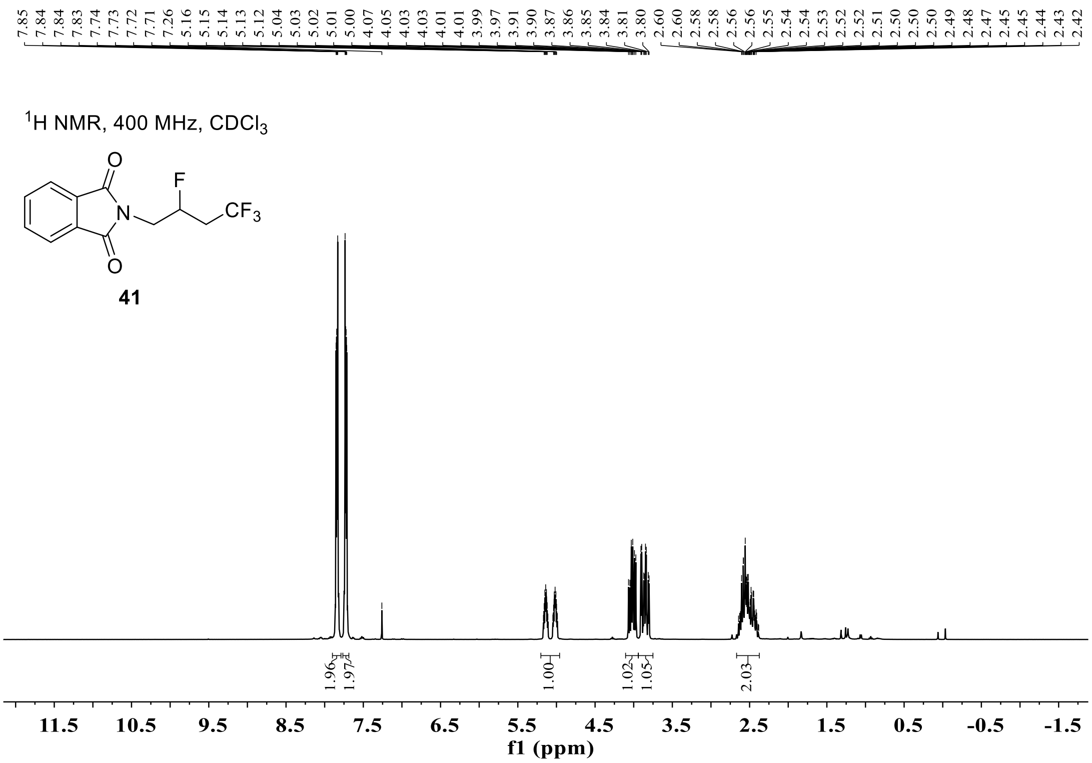
7.85 7.84 7.84 7.83 7.74 7.73 7.72 7.71 7.26 5.16 5.15 5.14 5.13 5.12 5.04 5.03 5.02 5.01 5.00 4.07 4.05 4.03 4.03 4.01 4.01 3.99 3.97 3.91 3.90 3.87 3.86 3.85 3.84 3.81 3.80 2.60 2.58 2.58 2.56 2.56 2.55 2.54 2.54 2.53 2.52 2.52 2.51 2.50 2.50 2.49 2.48 2.47 2.45 2.45 2.44 2.43 2.42
^{1}H NMR, 400 MHz, $CDCl_3$
O
F
N
CF_3
O
41
1.96
1.97
1.00
1.02
1.05
2.03
11.5 10.5 9.5 8.5 7.5 6.5 5.5 4.5 3.5 2.5 1.5 0.5 -0.5 -1.5
f1 (ppm)

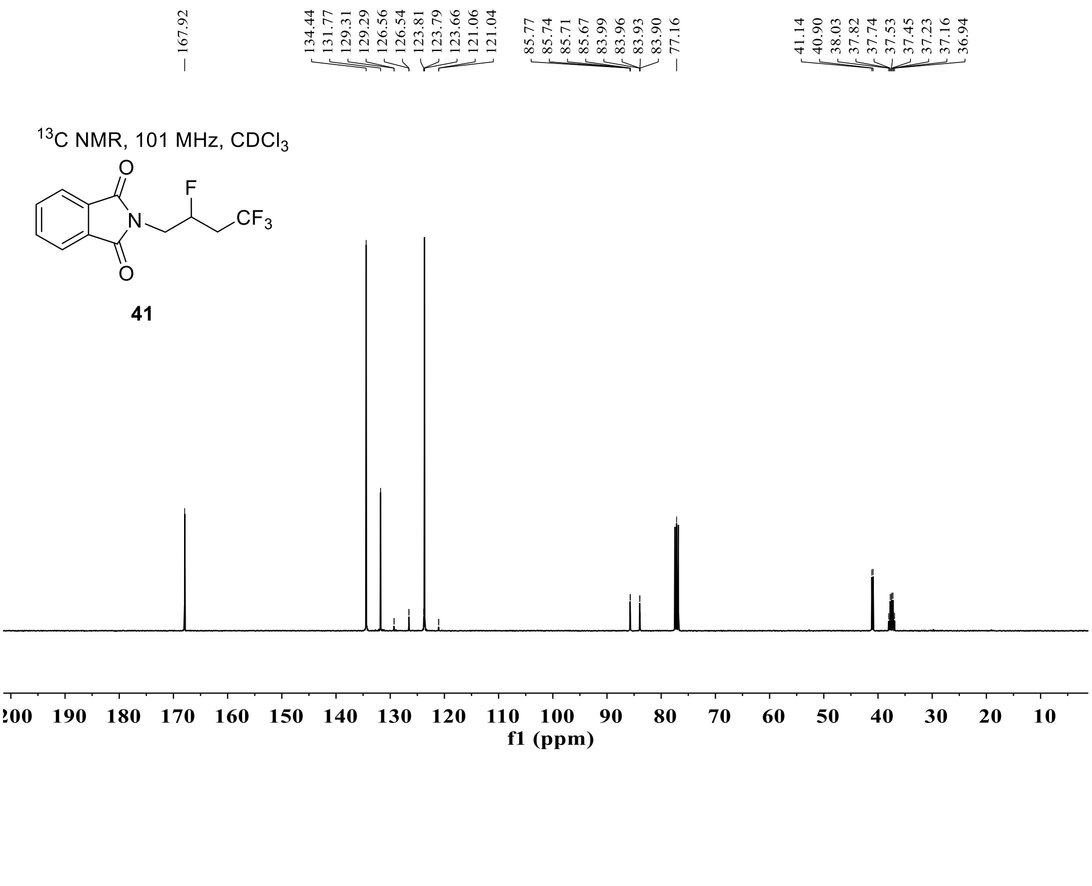
167.92
134.44 131.77 129.31 129.29 126.56 126.54 123.81 123.79 123.66 121.06 121.04
85.77 85.74 85.71 85.67 83.99 83.96 83.93 83.90 77.16
41.14 40.90 38.03 37.82 37.74 37.53 37.45 37.23 37.16 36.94
^{13}C NMR, 101 MHz, $CDCl_3$
O
F
N
CF_3
O
41
200 190 180 170 160 150 140 130 120 110 100 90 80 70 60 50 40 30 20 10
f1 (ppm)

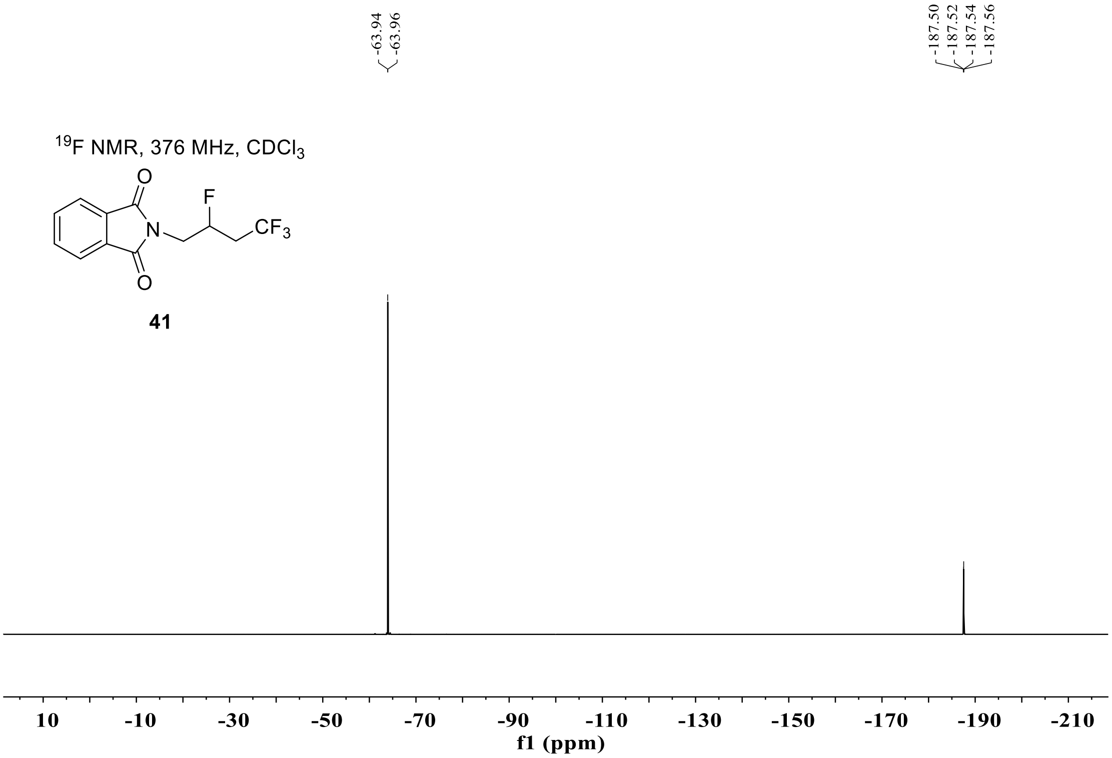
-63.94
-63.96
-187.50
-187.52
-187.54
-187.56
19F NMR, 376 MHz, CDCl3
O
F
N
CF3
O
41
10 -10 -30 -50 -70 -90 -110 -130 -150 -170 -190 -210
f1 (ppm)

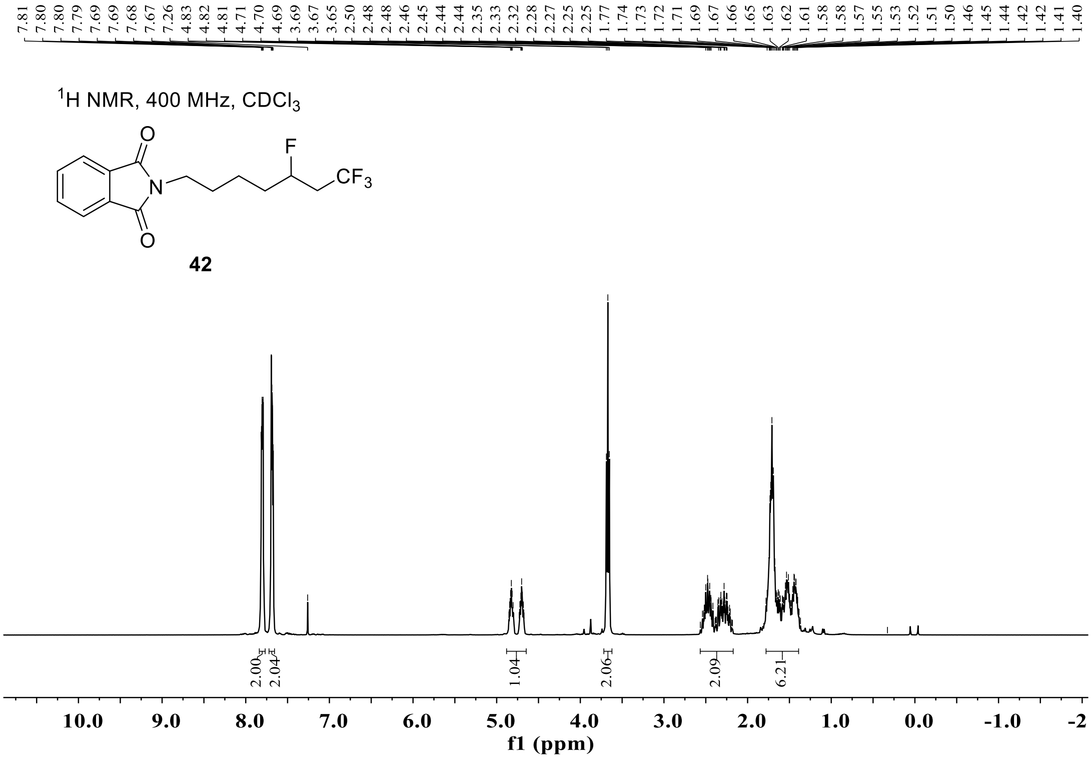
7.81 7.80 7.80 7.79 7.69 7.69 7.68 7.67 7.26 4.83 4.82 4.81 4.71 4.70 4.69 3.69 3.67 3.65 2.50 2.48 2.48 2.46 2.45 2.44 2.44 2.35 2.33 2.32 2.28 2.27 2.25 2.25 1.77 1.74 1.73 1.72 1.71 1.69 1.67 1.66 1.65 1.63 1.62 1.61 1.58 1.58 1.57 1.55 1.53 1.52 1.51 1.50 1.46 1.45 1.44 1.42 1.42 1.41 1.40
1H NMR, 400 MHz, CDCl3
O
F
N
CF3
O
42
2.00
2.04
1.04
2.06
2.09
6.21
10.0 9.0 8.0 7.0 6.0 5.0 4.0 3.0 2.0 1.0 0.0 -1.0 -2
f1 (ppm)

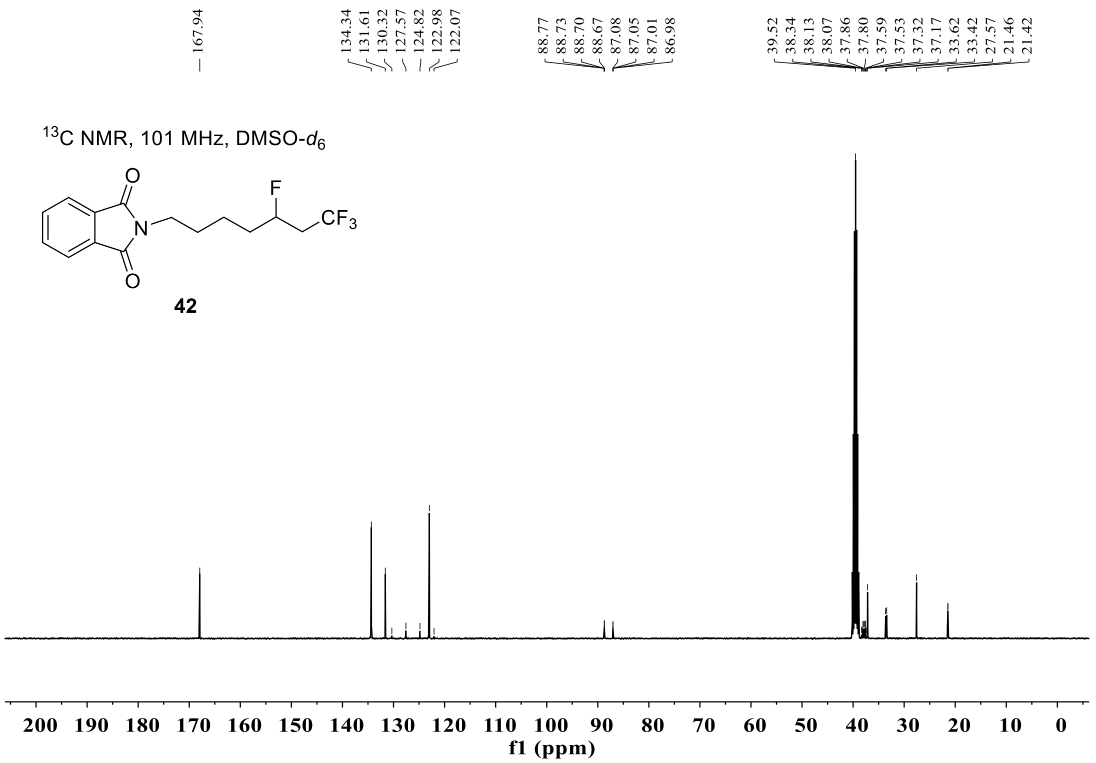
— 167.94
134.34
131.61
130.32
127.57
124.82
122.98
122.07
88.77
88.73
88.70
88.67
87.08
87.05
87.01
86.98
39.52
38.34
38.13
38.07
37.86
37.80
37.59
37.53
37.32
37.17
33.62
33.42
27.57
21.46
21.42
13C NMR, 101 MHz, DMSO-d6
O
F
N
CF3
O
42
200 190 180 170 160 150 140 130 120 110 100 90 80 70 60 50 40 30 20 10 0
f1 (ppm)

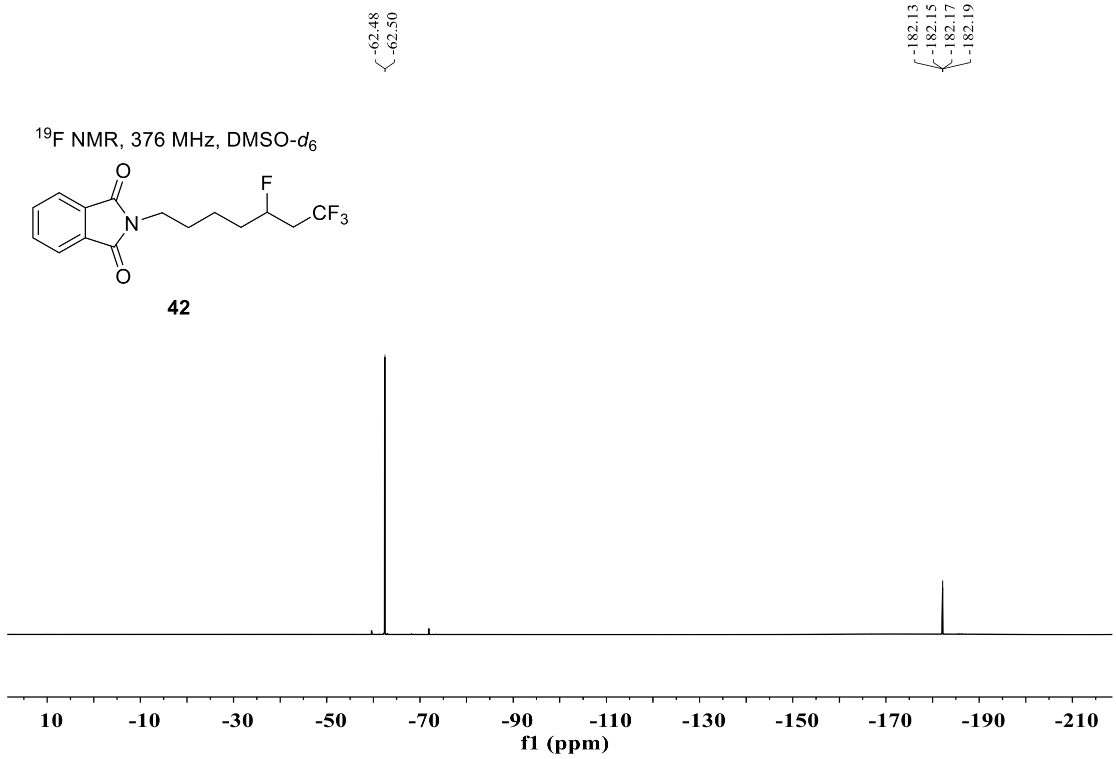
-62.48
-62.50
-182.13
-182.15
-182.17
-182.19
19F NMR, 376 MHz, DMSO-d6
O
F
N
CF3
O
42
10 -10 -30 -50 -70 -90 -110 -130 -150 -170 -190 -210
f1 (ppm)

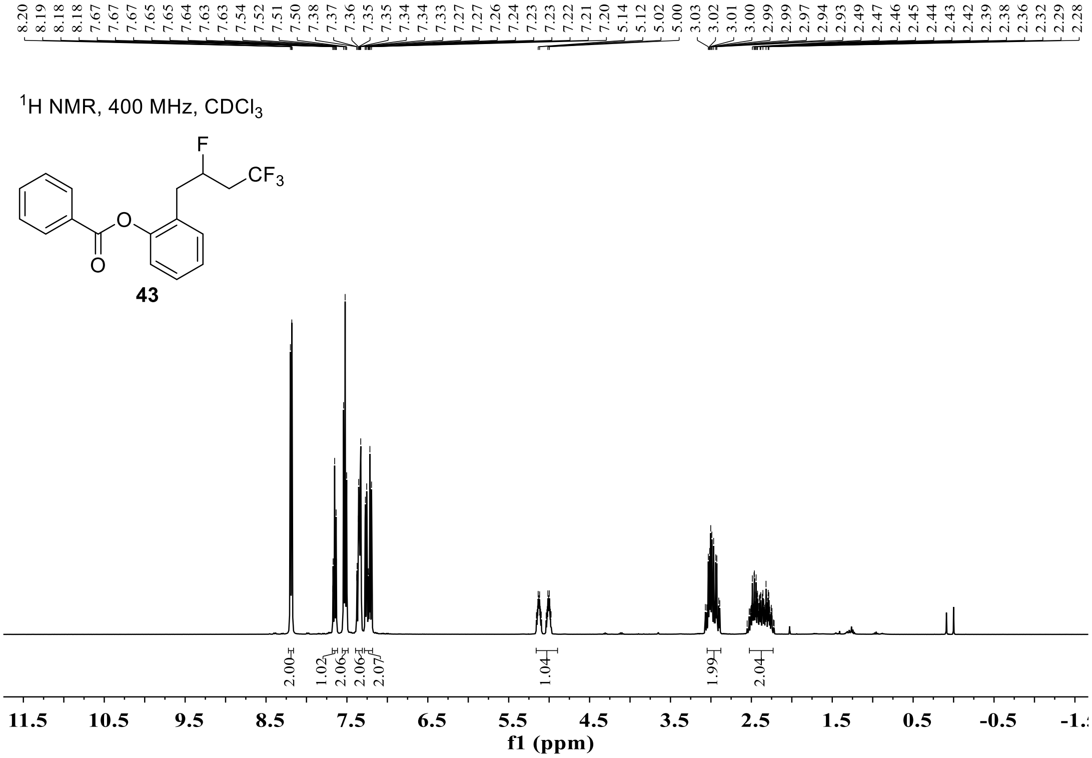
8.20 8.19 8.18 8.18 7.67 7.67 7.65 7.65 7.64 7.63 7.63 7.54 7.52 7.51 7.50 7.38 7.37 7.36 7.35 7.35 7.34 7.34 7.33 7.27 7.27 7.26 7.24 7.23 7.23 7.22 7.21 7.20 5.14 5.12 5.02 5.00 3.03 3.02 3.01 3.00 2.99 2.99 2.97 2.94 2.93 2.49 2.47 2.46 2.45 2.44 2.43 2.42 2.39 2.38 2.36 2.32 2.29 2.28
^{1}H NMR, 400 MHz, $CDCl_3$
F
CF_3
O
O
43
2.00 1.02 2.06 2.06 2.07 1.04 1.99 2.04
11.5 10.5 9.5 8.5 7.5 6.5 5.5 4.5 3.5 2.5 1.5 0.5 -0.5
f1 (ppm)

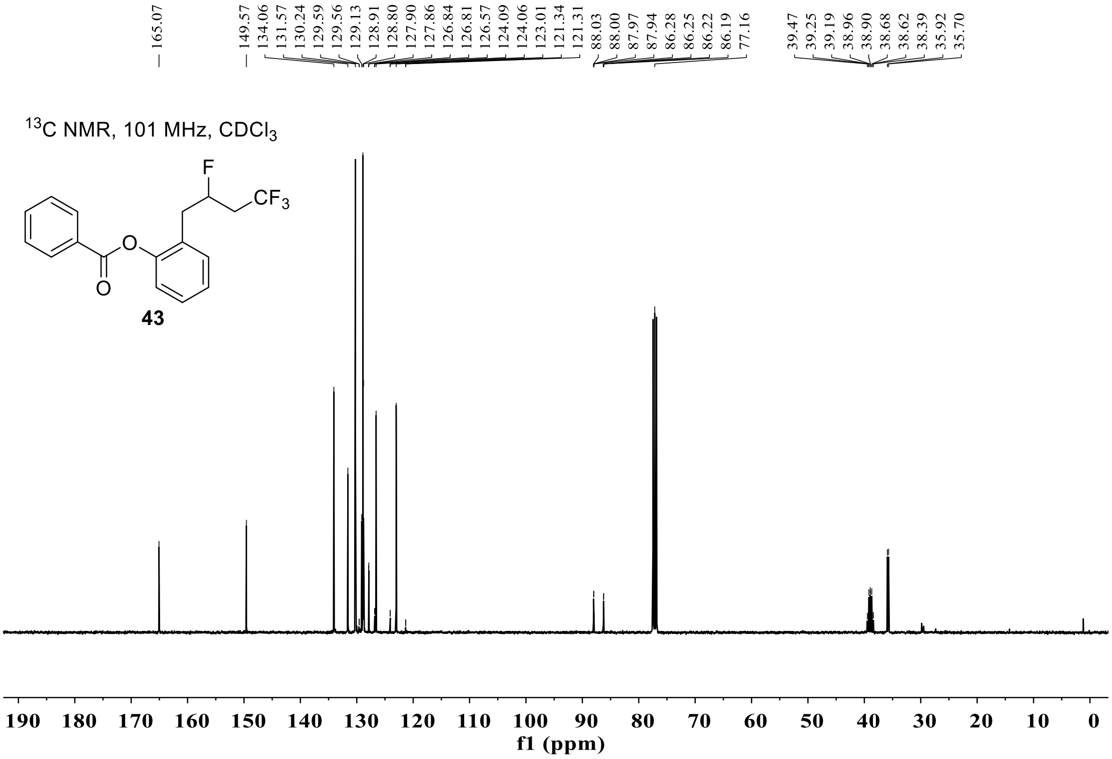
165.07
149.57 134.06 131.57 130.24 129.59 129.56 129.13 128.91 128.80 127.90 127.86 126.84 126.81 126.57 124.09 124.06 123.01 121.34 121.31 88.03 88.00 87.97 87.94 86.28 86.25 86.22 86.19 77.16
39.47 39.25 39.19 38.96 38.90 38.68 38.62 38.39 35.92 35.70
^{13}C NMR, 101 MHz, $CDCl_3$
F
CF_3
O
O
43
190 180 170 160 150 140 130 120 110 100 90 80 70 60 50 40 30 20 10 0
f1 (ppm)

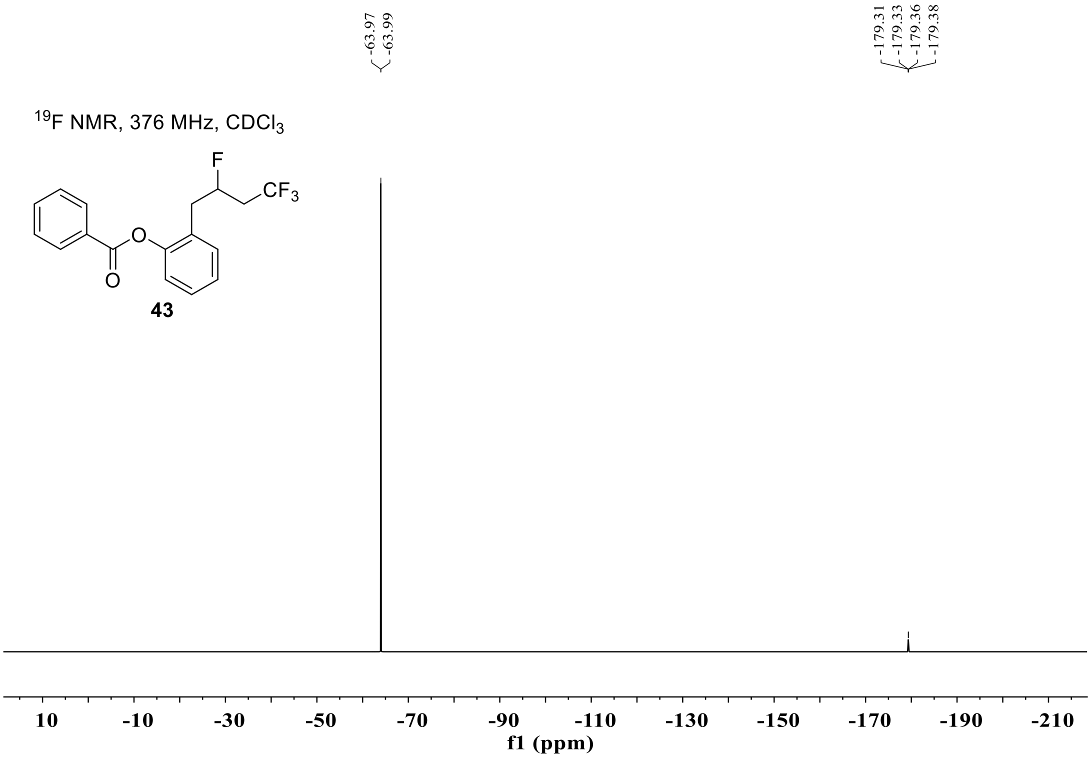
-63.97
-63.99
-179.31
-179.33
-179.36
-179.38
19F NMR, 376 MHz, CDCl3
F
CF3
O
O
43
10 -10 -30 -50 -70 -90 -110 -130 -150 -170 -190 -210
f1 (ppm)

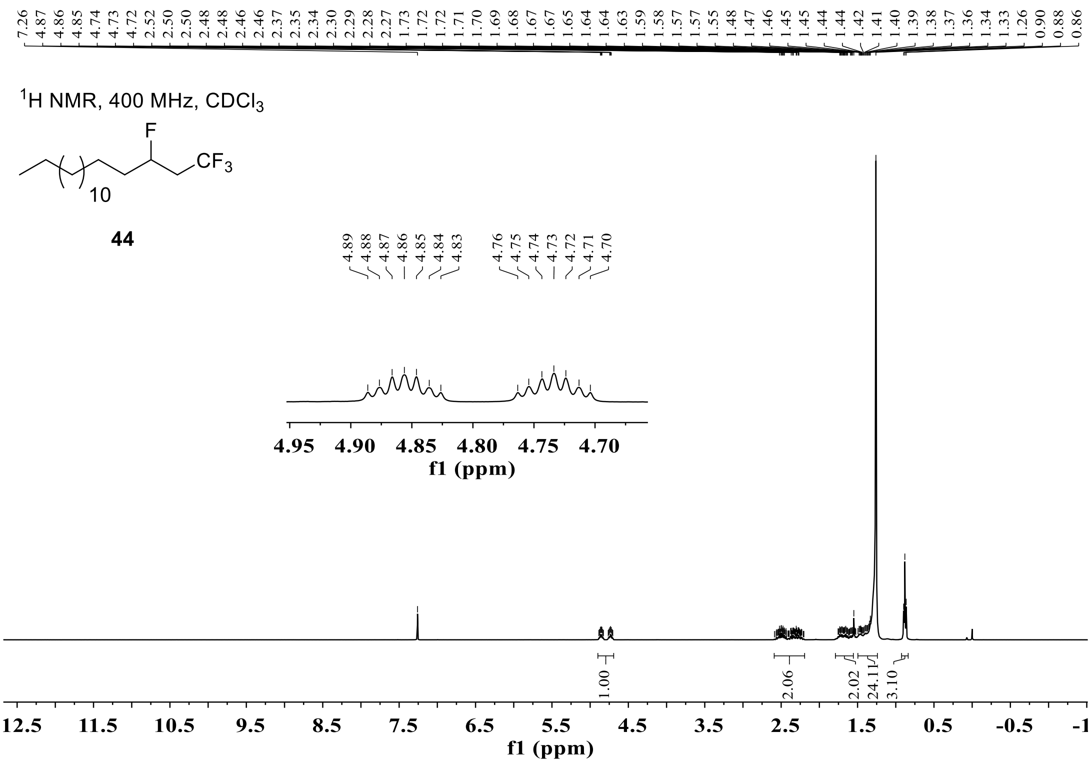
7.26 4.87 4.86 4.85 4.74 4.73 4.72 2.52 2.50 2.50 2.48 2.48 2.46 2.46 2.37 2.35 2.34 2.30 2.29 2.28 2.27 1.73 1.72 1.72 1.71 1.70 1.69 1.68 1.67 1.67 1.65 1.64 1.64 1.63 1.59 1.58 1.57 1.57 1.55 1.48 1.47 1.46 1.45 1.45 1.44 1.42 1.41 1.40 1.39 1.38 1.37 1.36 1.34 1.33 1.26 0.90 0.88 0.86
1H NMR, 400 MHz, CDCl3
F
CF3
10
44
4.89 4.88 4.87 4.86 4.85 4.84 4.83
4.76 4.75 4.74 4.73 4.72 4.71 4.70
4.95 4.90 4.85 4.80 4.75 4.70
f1 (ppm)
1.00
2.06
2.02
24.11
3.10
12.5 11.5 10.5 9.5 8.5 7.5 6.5 5.5 4.5 3.5 2.5 1.5 0.5 -0.5 -1
f1 (ppm)

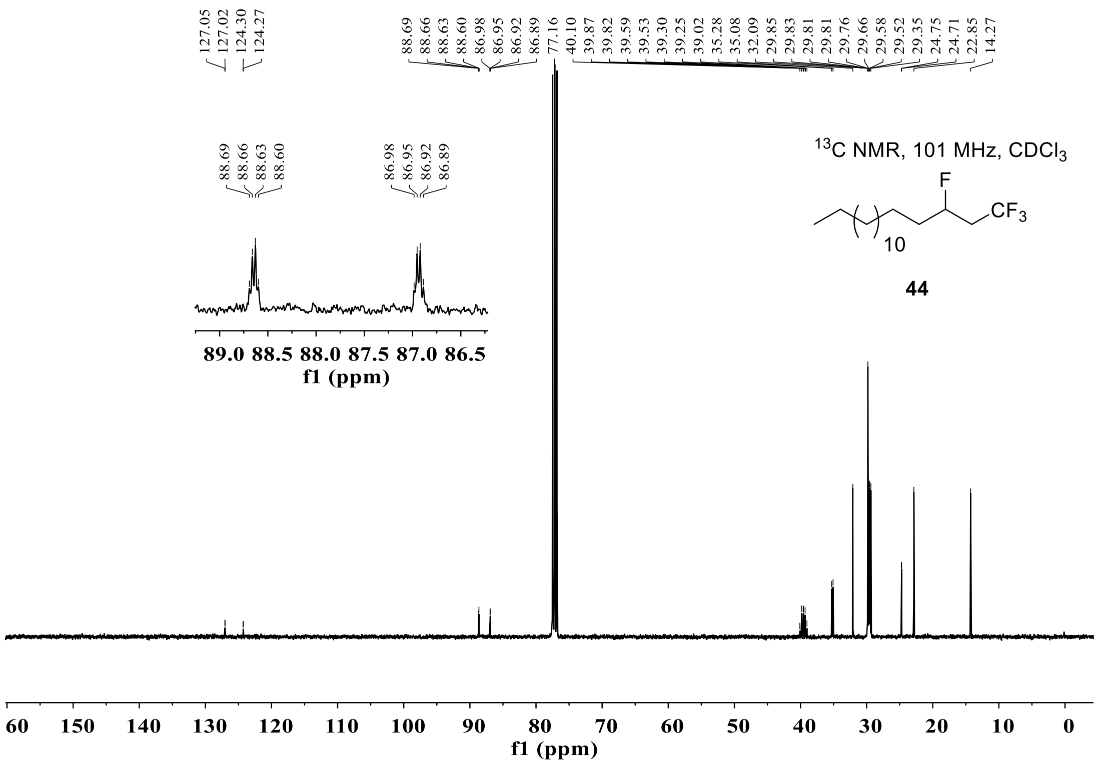

127.05
127.02
124.30
124.27
88.69
88.66
88.63
88.60
86.98
86.95
86.92
86.89
77.16
40.10
39.87
39.82
39.59
39.53
39.30
39.25
39.02
35.28
35.08
32.09
29.85
29.83
29.81
29.81
29.76
29.66
29.58
29.52
29.35
24.75
24.71
22.85
14.27
^{13}C NMR, 101 MHz, CDCl$_3$
F
CF$_3$
10
44
89.0 88.5 88.0 87.5 87.0 86.5
f1 (ppm)
60 150 140 130 120 110 100 90 80 70 60 50 40 30 20 10 0
f1 (ppm)

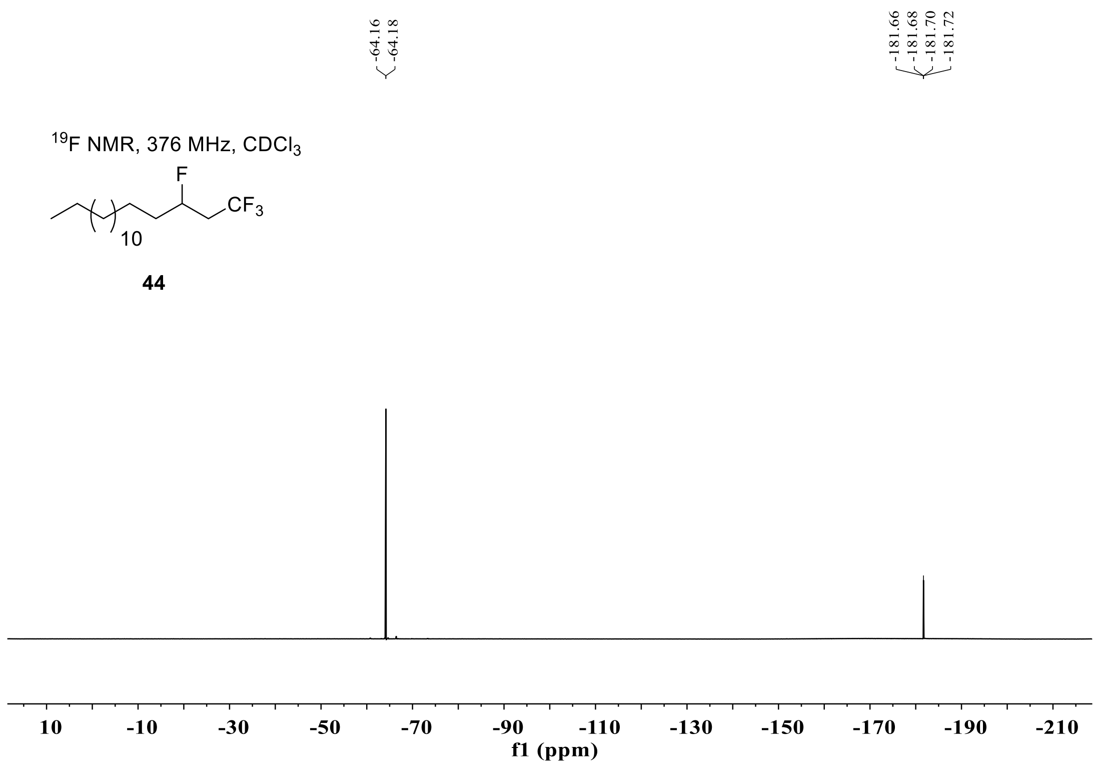

-64.16
-64.18
-181.66
-181.68
-181.70
-181.72
^{19}F NMR, 376 MHz, CDCl$_3$
F
CF$_3$
10
44
10 -10 -30 -50 -70 -90 -110 -130 -150 -170 -190 -210
f1 (ppm)

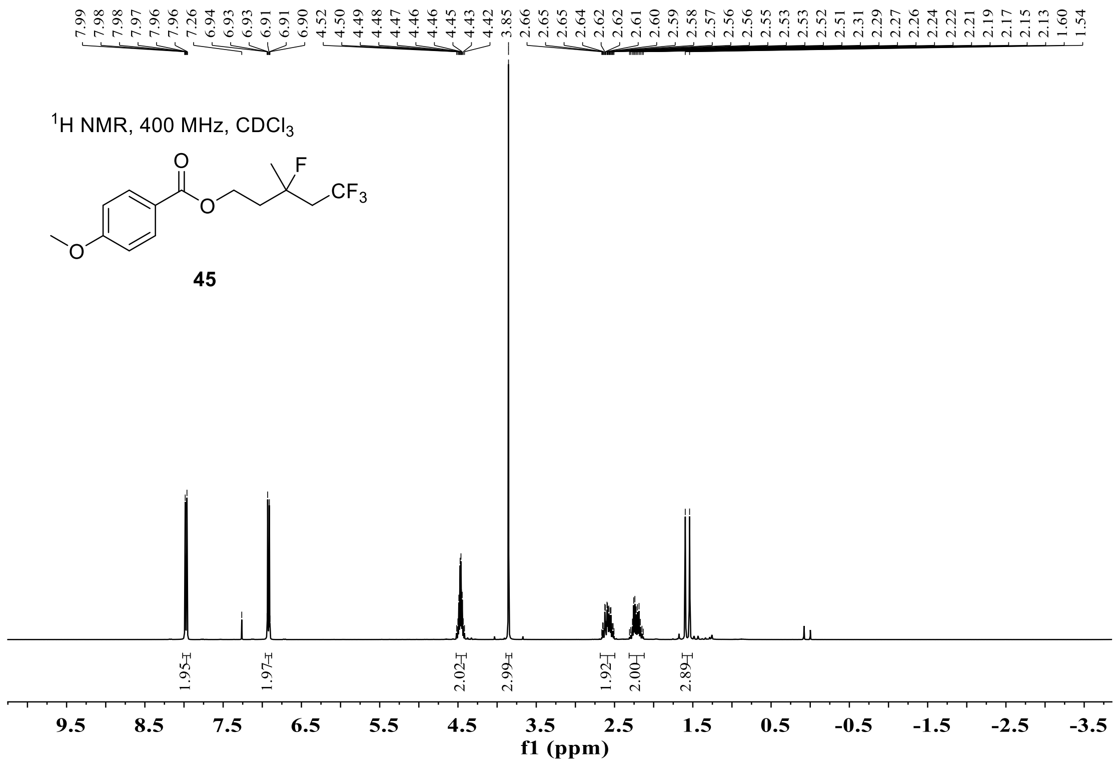

^{1}H NMR, 400 MHz, $CDCl_3$
45
f1 (ppm)

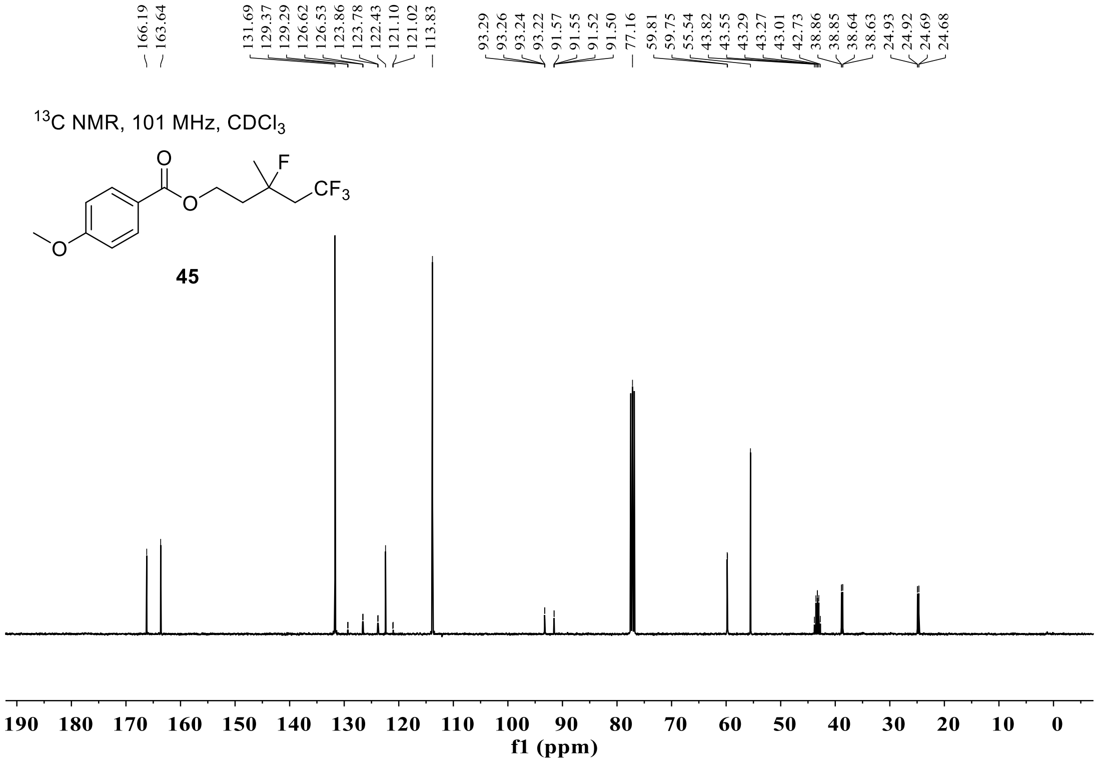

^{13}C NMR, 101 MHz, $CDCl_3$
45
f1 (ppm)

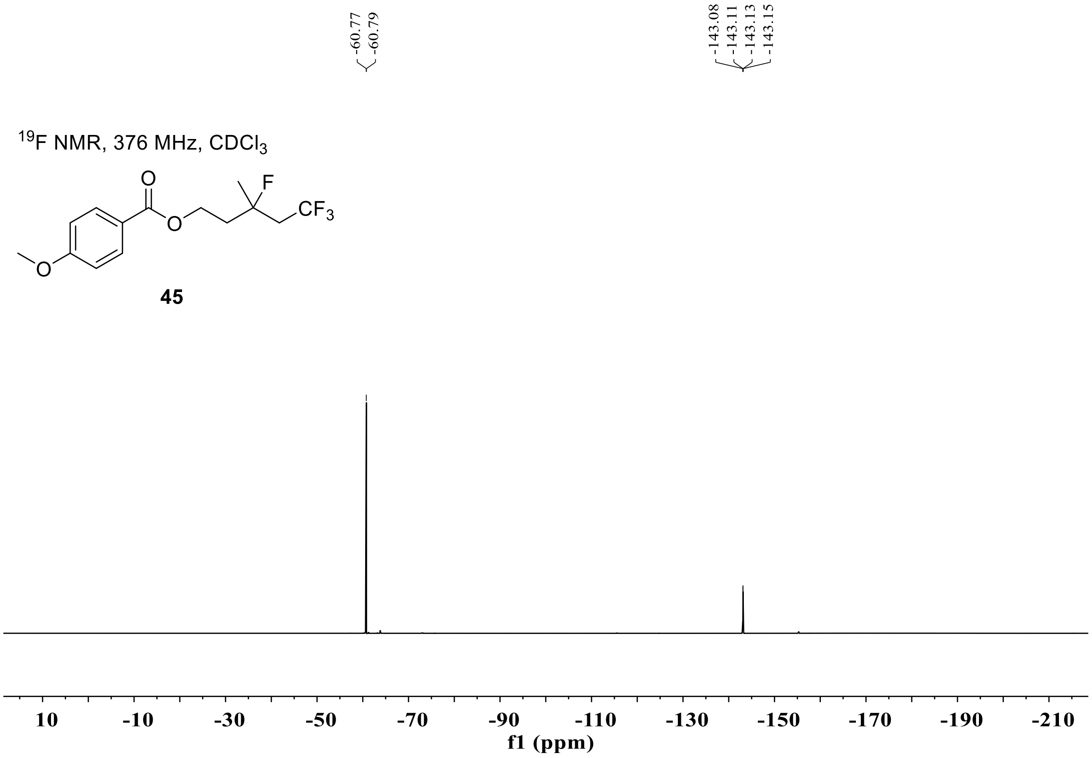

^{19}F NMR, 376 MHz, $CDCl_3$

45

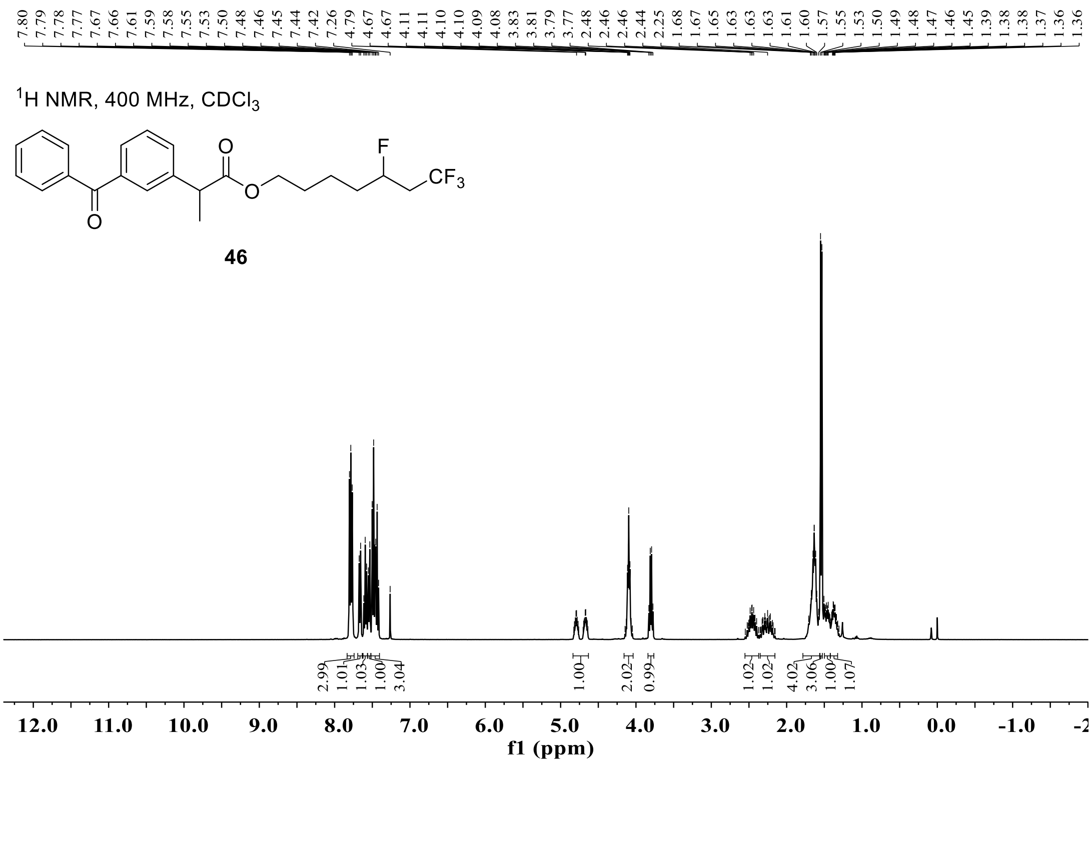

^{1}H NMR, 400 MHz, $CDCl_3$

46

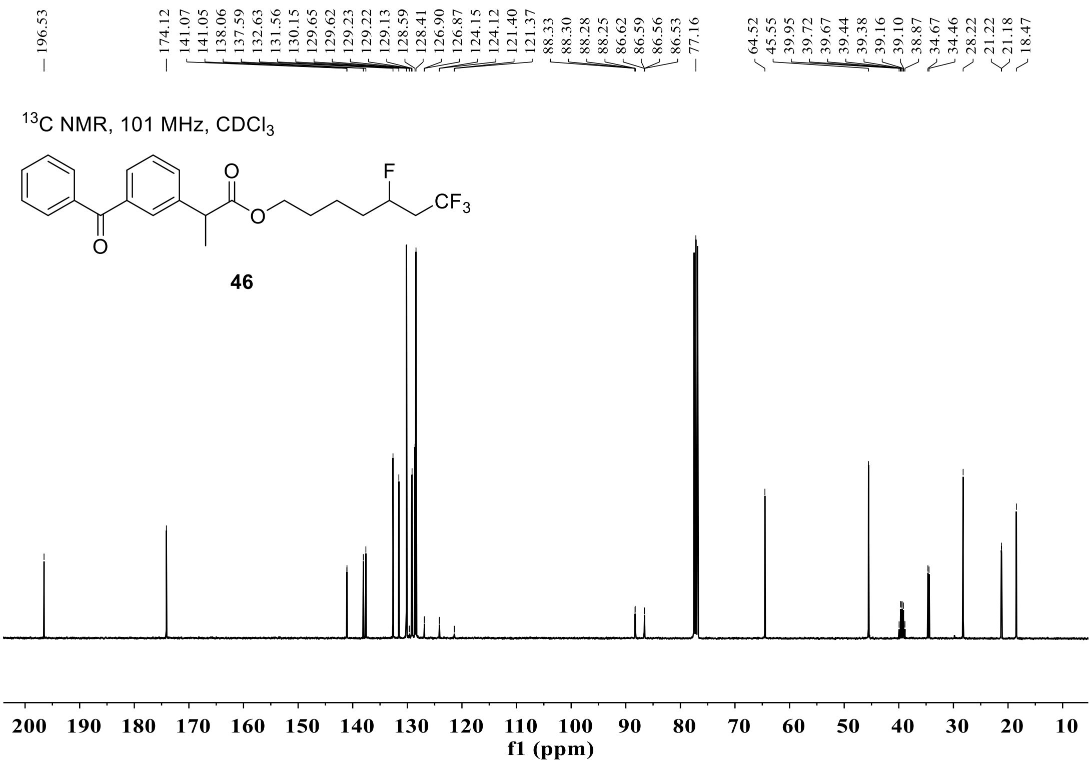
196.53
174.12
141.07
141.05
138.06
137.59
132.63
131.56
130.15
129.65
129.62
129.23
129.22
129.13
128.59
128.41
126.90
126.87
124.15
124.12
121.40
121.37
88.33
88.30
88.28
88.25
86.62
86.59
86.56
86.53
77.16
64.52
45.55
39.95
39.72
39.67
39.44
39.38
39.16
39.10
38.87
34.67
34.46
28.22
21.22
21.18
18.47
^{13}C NMR, 101 MHz, CDCl$_3$
O
F
CF$_3$
O
O
46
200 190 180 170 160 150 140 130 120 110 100 90 80 70 60 50 40 30 20 10
f1 (ppm)

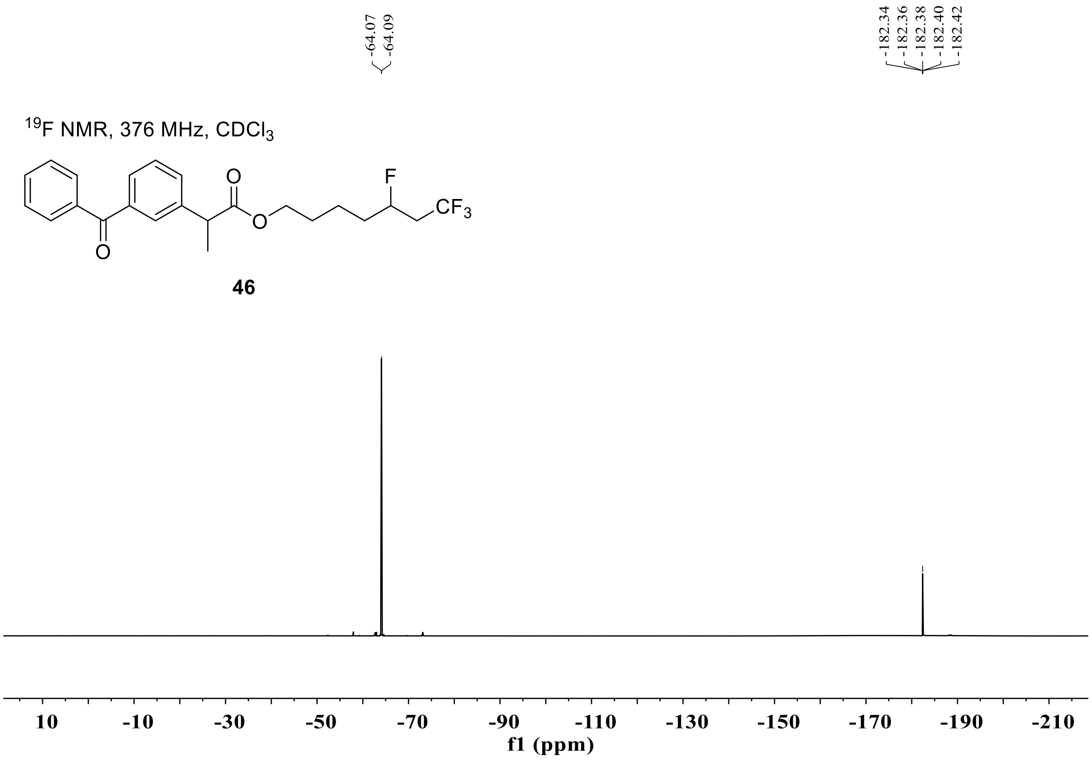
-64.07
-64.09
-182.34
-182.36
-182.38
-182.40
-182.42
^{19}F NMR, 376 MHz, CDCl$_3$
O
F
CF$_3$
O
O
46
10 -10 -30 -50 -70 -90 -110 -130 -150 -170 -190 -210
f1 (ppm)

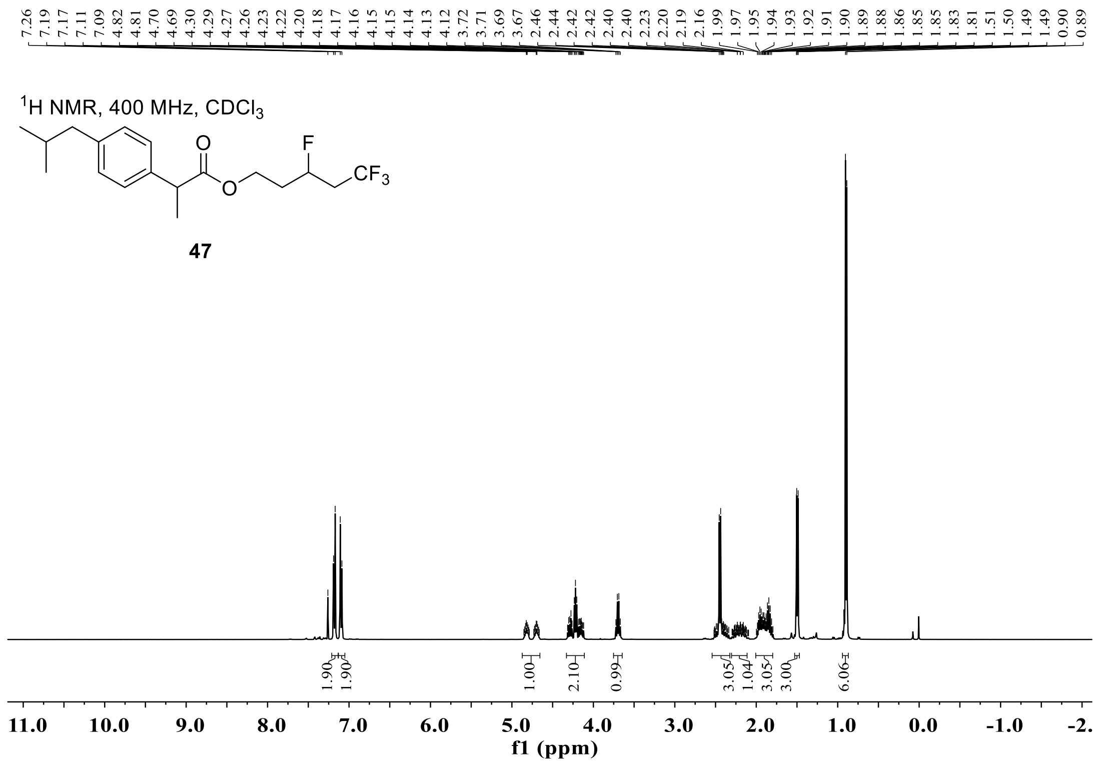
7.26 7.19 7.17 7.11 7.09 4.82 4.81 4.70 4.69 4.30 4.29 4.27 4.26 4.23 4.22 4.20 4.18 4.17 4.16 4.15 4.15 4.14 4.13 4.12 3.72 3.71 3.69 3.67 2.46 2.44 2.42 2.42 2.40 2.40 2.23 2.20 2.19 2.16 1.99 1.97 1.95 1.94 1.93 1.92 1.91 1.90 1.89 1.88 1.86 1.85 1.85 1.83 1.81 1.51 1.50 1.49 1.49 0.90 0.89
1H NMR, 400 MHz, CDCl3
O
F
O
CF3
47
1.90 1.90 1.00 2.10 0.99 3.05 1.04 3.05 3.00 6.06
11.0 10.0 9.0 8.0 7.0 6.0 5.0 4.0 3.0 2.0 1.0 0.0 -1.0 -2.
f1 (ppm)

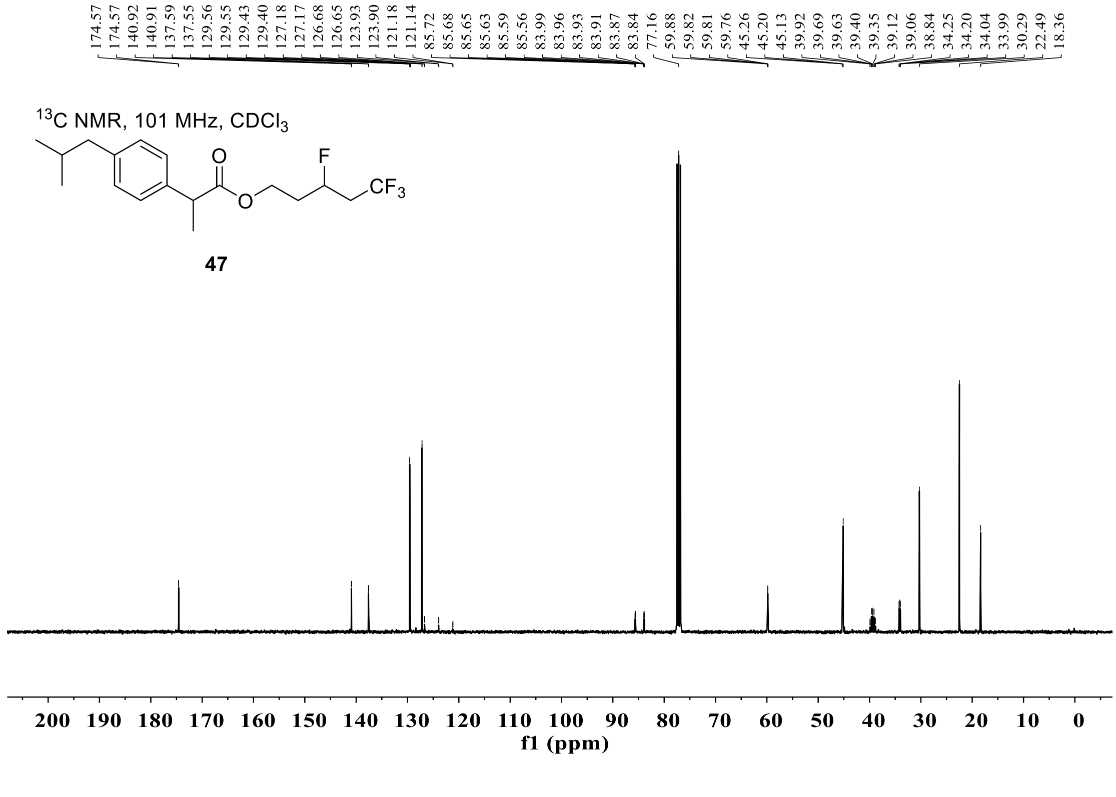
174.57 174.57 140.92 140.91 137.59 137.55 129.56 129.55 129.43 129.40 127.18 127.17 126.68 126.65 123.93 123.90 121.18 121.14 85.72 85.68 85.65 85.63 85.59 85.56 83.99 83.96 83.93 83.91 83.87 83.84 77.16 59.88 59.82 59.81 59.76 45.26 45.20 45.13 39.92 39.69 39.63 39.40 39.35 39.12 39.06 38.84 34.25 34.20 34.04 33.99 30.29 22.49 18.36
13C NMR, 101 MHz, CDCl3
O
F
O
CF3
47
200 190 180 170 160 150 140 130 120 110 100 90 80 70 60 50 40 30 20 10 0
f1 (ppm)

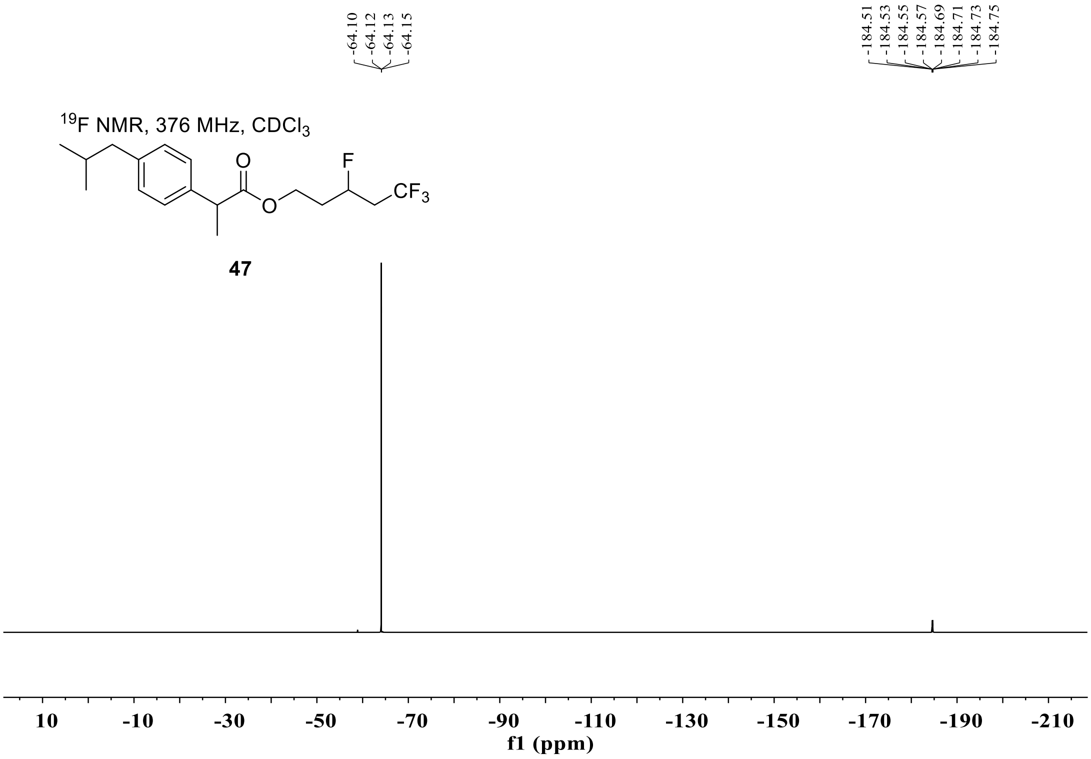

-64.10
-64.12
-64.13
-64.15
-184.51
-184.53
-184.55
-184.57
-184.69
-184.71
-184.73
-184.75
^{19}F NMR, 376 MHz, CDCl$_3$
O
F
CF$_3$
47
f1 (ppm)

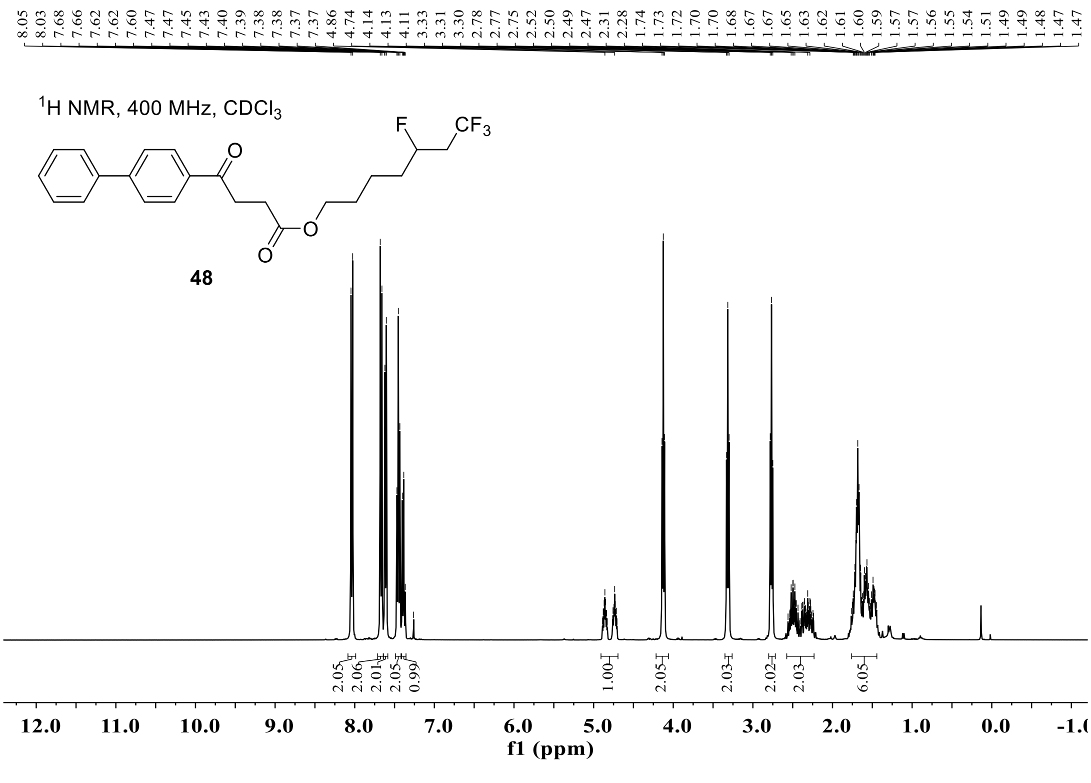

^{1}H NMR, 400 MHz, CDCl$_3$
F
CF$_3$
O
48
f1 (ppm)

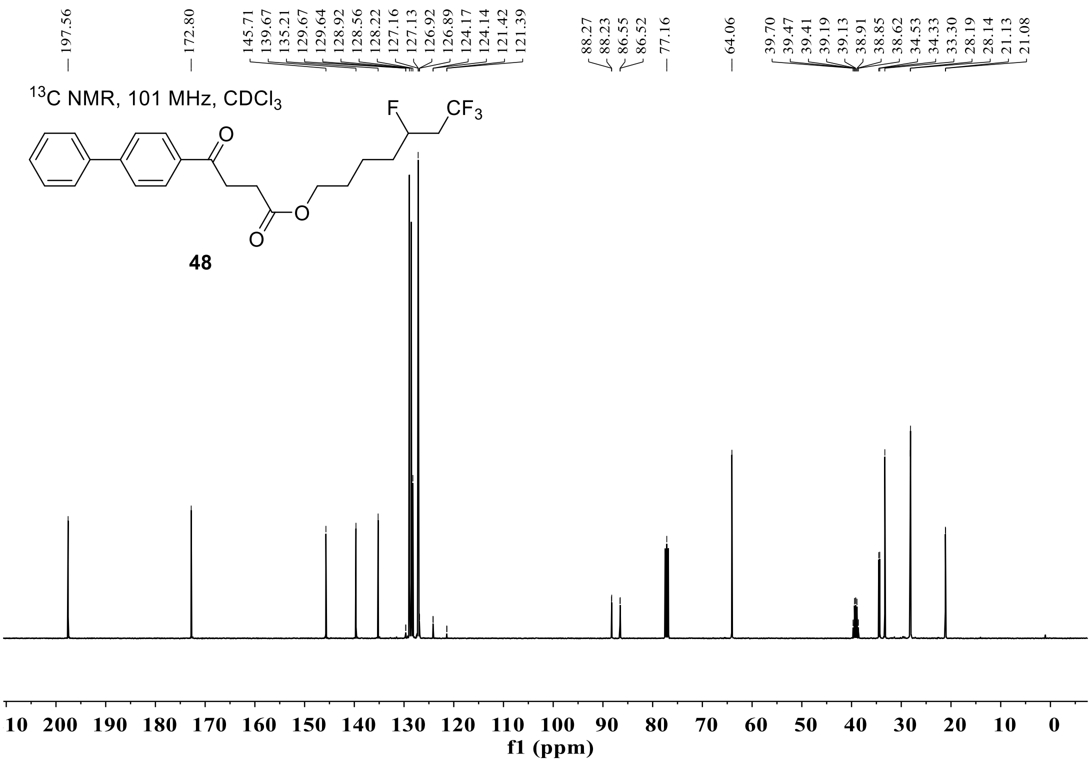

— 197.56
— 172.80
145.71
139.67
135.21
129.67
129.64
128.92
128.56
128.22
127.16
127.13
126.92
126.89
124.17
124.14
121.42
121.39
88.27
88.23
86.55
86.52
— 77.16
— 64.06
39.70
39.47
39.41
39.19
39.13
38.91
38.85
38.62
34.53
34.33
33.30
28.19
28.14
21.13
21.08
^{13}C NMR, 101 MHz, CDCl$_3$
F
CF$_3$
O
O
O
48
10 200 190 180 170 160 150 140 130 120 110 100 90 80 70 60 50 40 30 20 10 0
f1 (ppm)

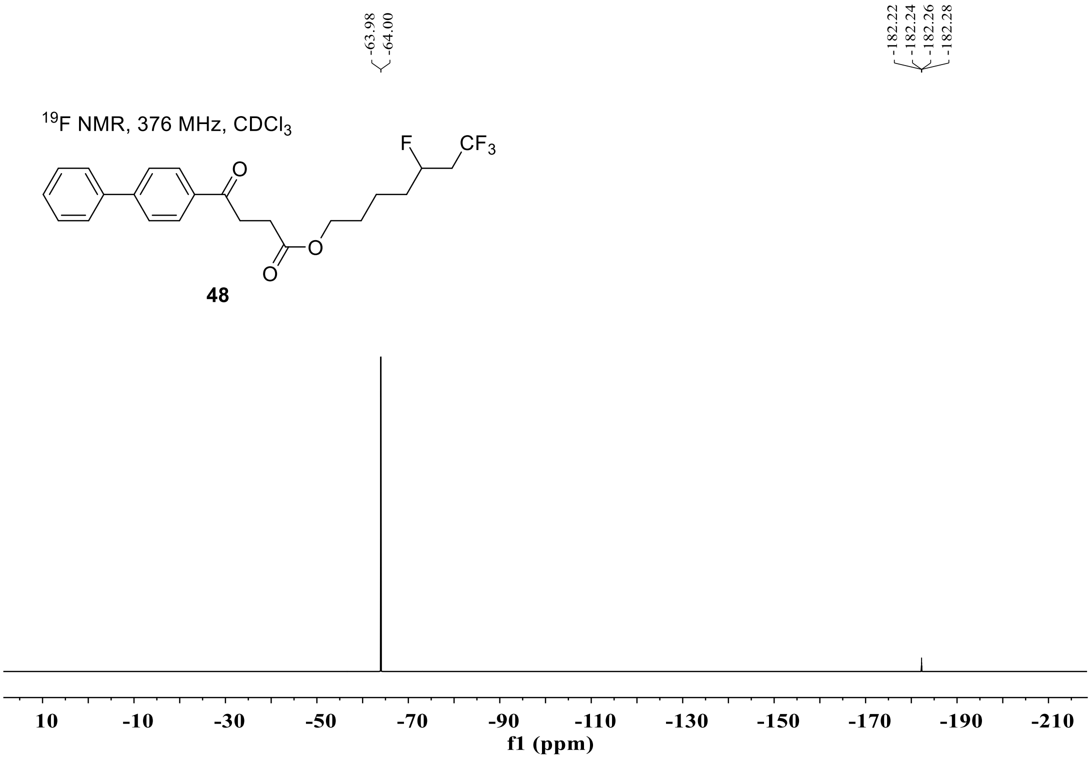

-63.98
-64.00
-182.22
-182.24
-182.26
-182.28
^{19}F NMR, 376 MHz, CDCl$_3$
F
CF$_3$
O
O
O
48
10 -10 -30 -50 -70 -90 -110 -130 -150 -170 -190 -210
f1 (ppm)

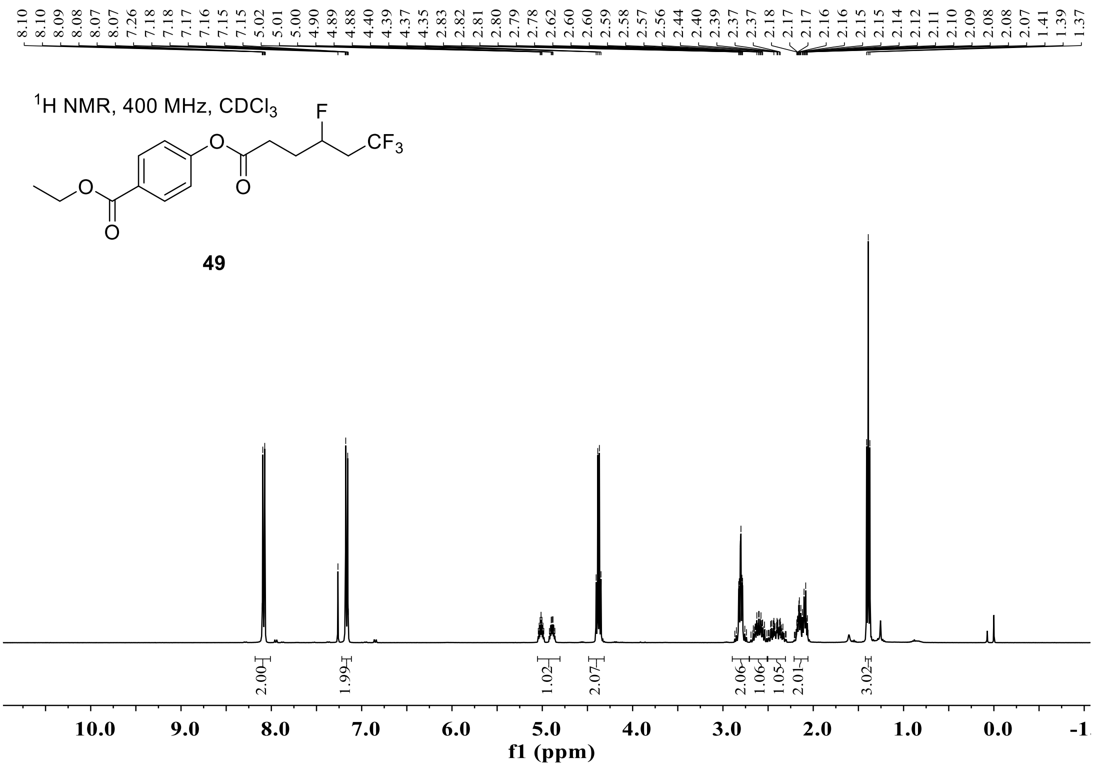
8.10 8.10 8.09 8.08 8.07 8.07 7.26 7.18 7.18 7.17 7.16 7.15 7.15 5.02 5.01 5.00 4.90 4.89 4.88 4.40 4.39 4.37 4.35 2.83 2.82 2.81 2.80 2.79 2.78 2.62 2.60 2.60 2.59 2.58 2.57 2.56 2.44 2.40 2.39 2.37 2.37 2.18 2.17 2.17 2.16 2.16 2.15 2.15 2.14 2.12 2.11 2.10 2.09 2.08 2.07 1.41 1.39 1.37
^{1}H NMR, 400 MHz, CDCl$_3$
49
2.00 1.99 1.02 2.07 2.06 1.06 1.05 2.01 3.02
f1 (ppm)

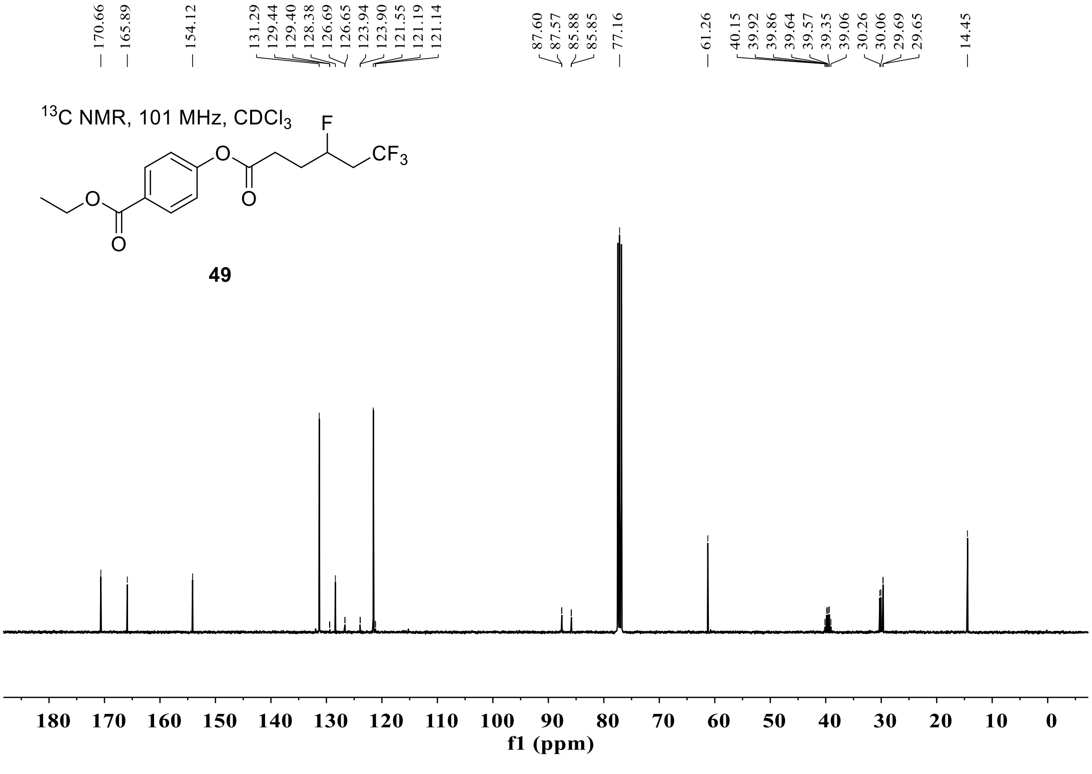
170.66 165.89 154.12 131.29 129.44 129.40 128.38 126.69 126.65 123.94 123.90 121.55 121.19 121.14 87.60 87.57 85.88 85.85 77.16 61.26 40.15 39.92 39.86 39.64 39.57 39.35 39.06 30.26 30.06 29.69 29.65 14.45
^{13}C NMR, 101 MHz, CDCl$_3$
49
f1 (ppm)

^{19}F NMR, 376 MHz, $CDCl_3$

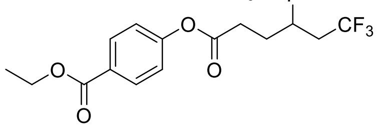

49

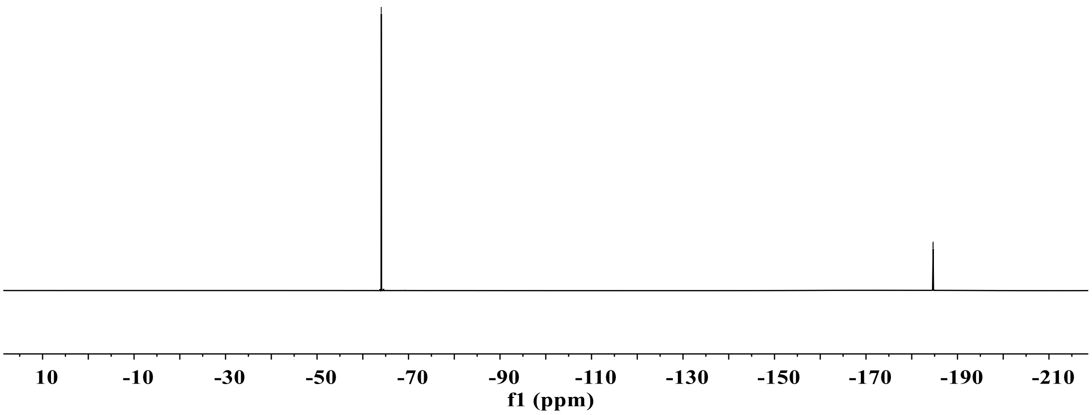

^{1}H NMR, 400 MHz, $CDCl_3$

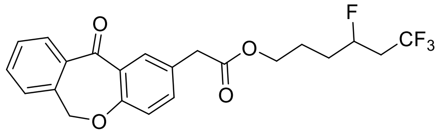

50

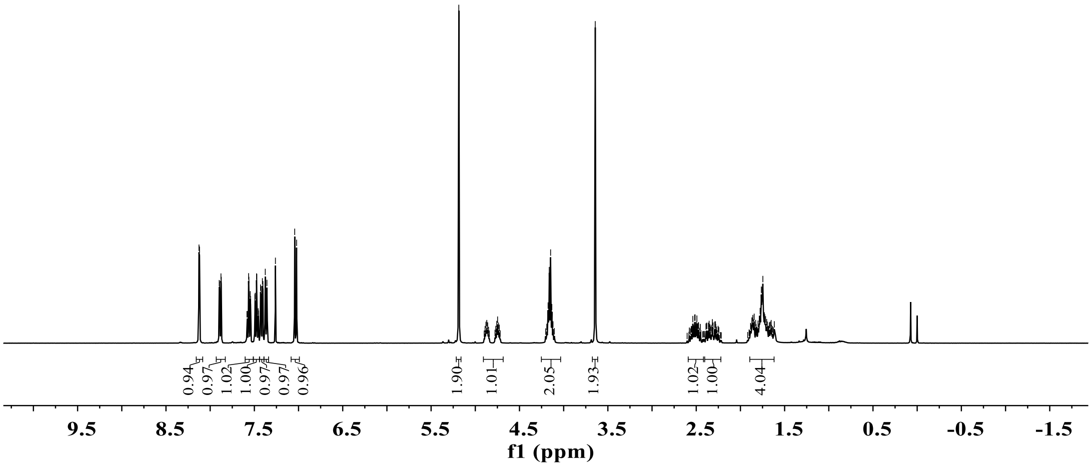

^{13}C NMR, 101 MHz, $CDCl_3$

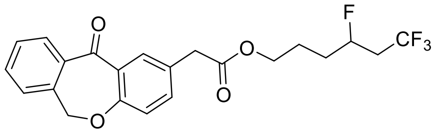

50

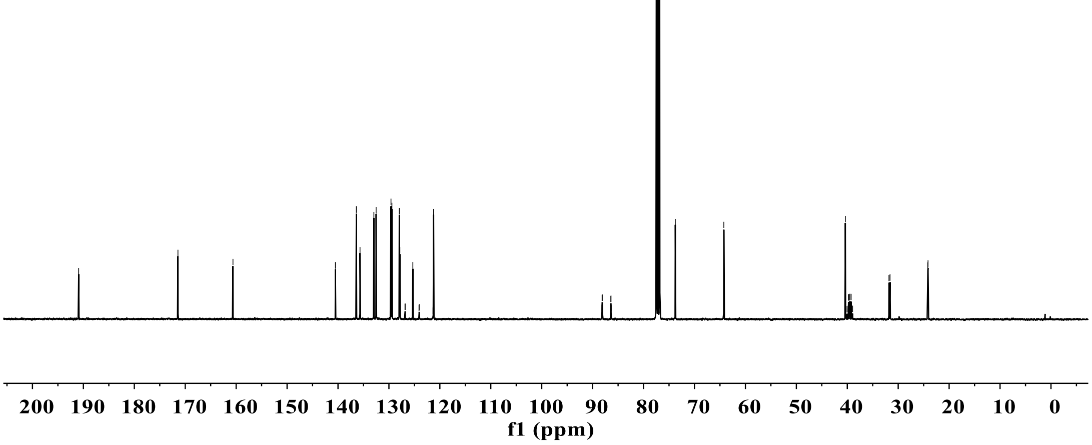

^{19}F NMR, 376 MHz, $CDCl_3$

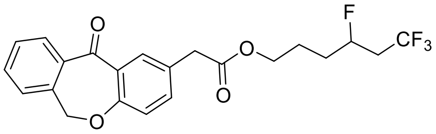

50

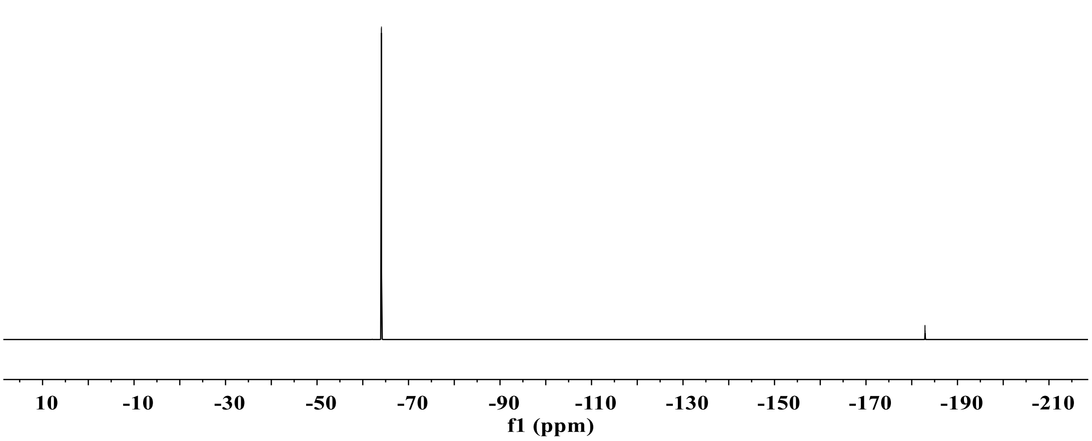

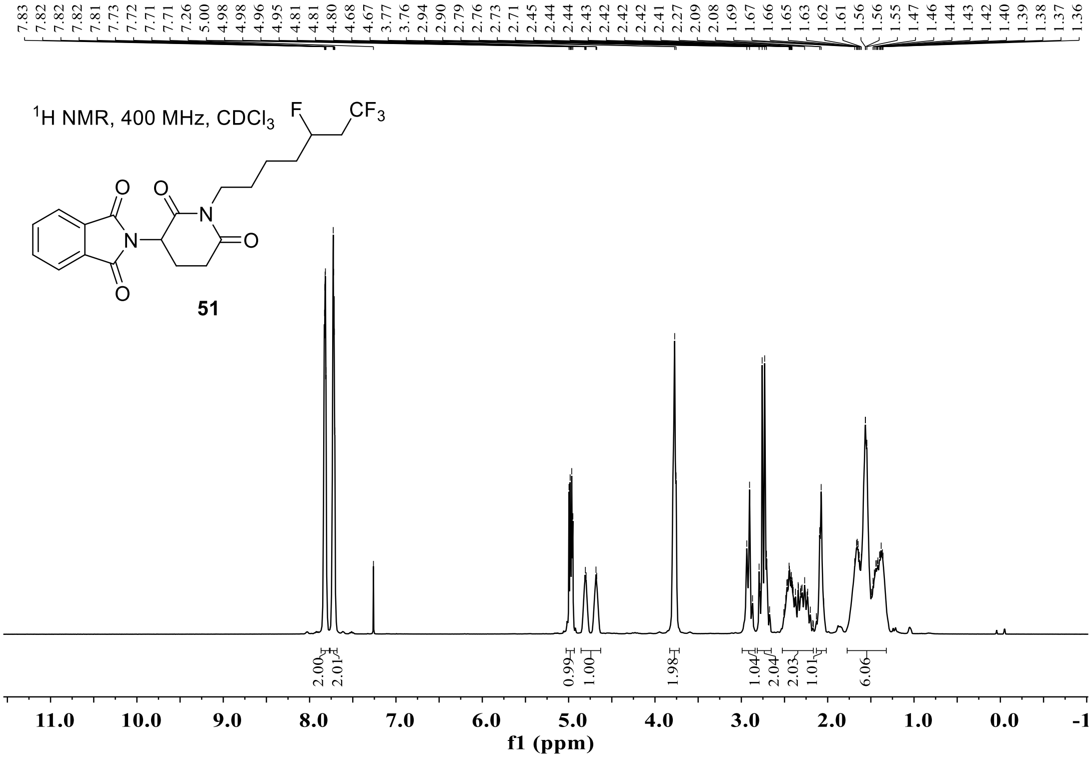

7.83 7.82 7.82 7.82 7.81 7.73 7.72 7.71 7.71 7.26 5.00 4.98 4.98 4.96 4.95 4.81 4.81 4.80 4.68 4.67 3.77 3.76 2.94 2.90 2.79 2.76 2.73 2.71 2.45 2.44 2.44 2.43 2.42 2.42 2.42 2.41 2.27 2.09 2.08 1.69 1.67 1.66 1.65 1.63 1.62 1.61 1.56 1.56 1.55 1.47 1.46 1.44 1.43 1.42 1.40 1.39 1.38 1.37 1.36
^{1}H NMR, 400 MHz, $CDCl_3$
51
2.00 2.01 0.99 1.00 1.98 1.04 2.04 2.03 1.01 6.06
11.0 10.0 9.0 8.0 7.0 6.0 5.0 4.0 3.0 2.0 1.0 0.0 -1
f1 (ppm)

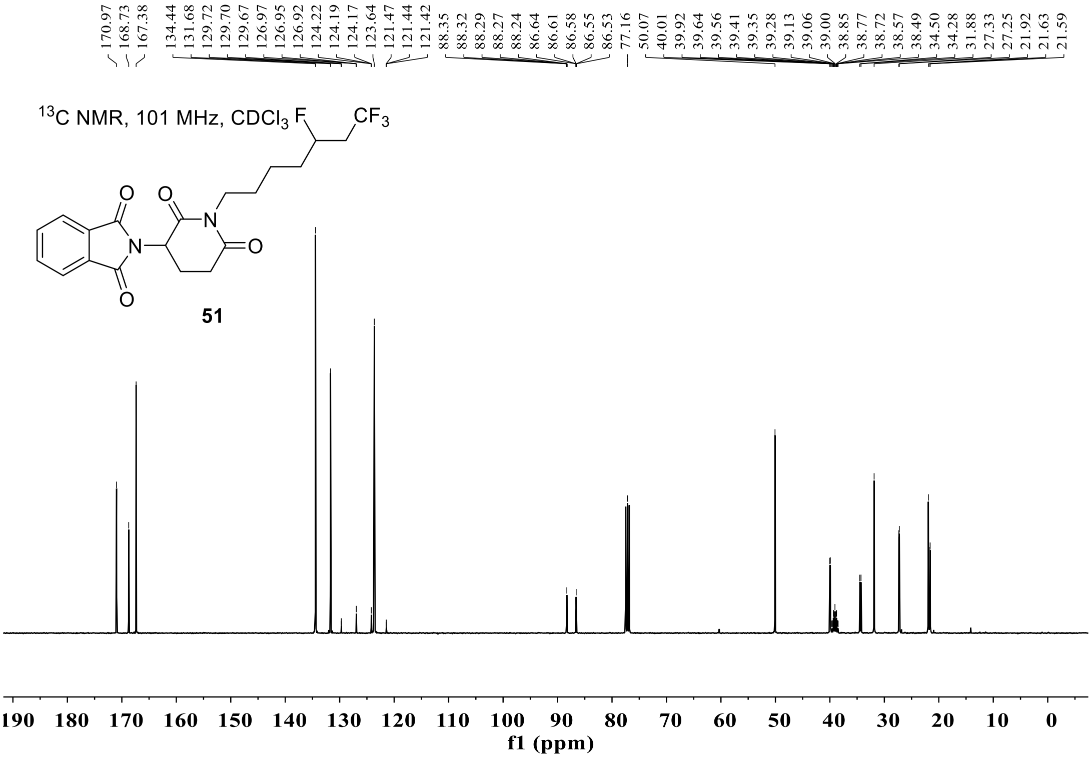

170.97 168.73 167.38 134.44 131.68 129.72 129.70 129.67 126.97 126.95 126.92 124.22 124.19 124.17 123.64 121.47 121.44 121.42 88.35 88.32 88.29 88.27 88.24 86.64 86.61 86.58 86.55 86.53 77.16 50.07 40.01 39.92 39.64 39.56 39.41 39.35 39.28 39.13 39.06 39.00 38.85 38.77 38.72 38.57 38.49 34.50 34.28 31.88 27.33 27.25 21.92 21.63 21.59
^{13}C NMR, 101 MHz, $CDCl_3$
51
190 180 170 160 150 140 130 120 110 100 90 80 70 60 50 40 30 20 10 0
f1 (ppm)

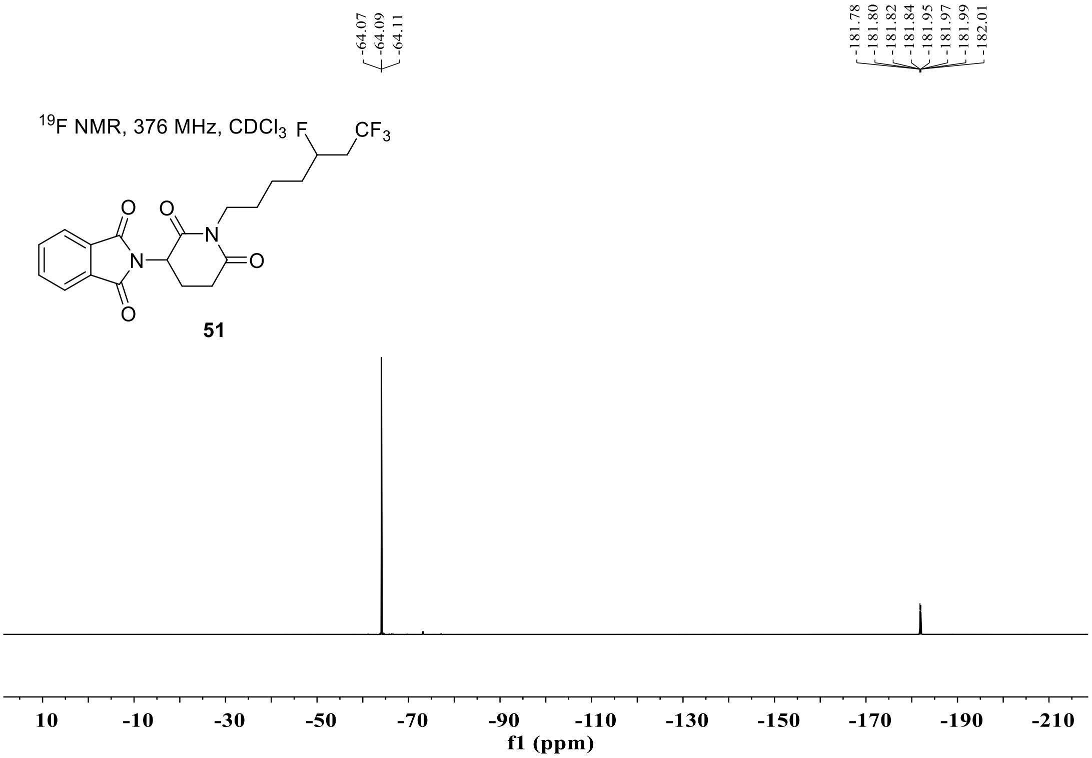

-64.07
-64.09
-64.11
-181.78
-181.80
-181.82
-181.84
-181.95
-181.97
-181.99
-182.01
19F NMR, 376 MHz, CDCl3
F
CF3
O
N
51
10 -10 -30 -50 -70 -90 -110 -130 -150 -170 -190 -210
f1 (ppm)

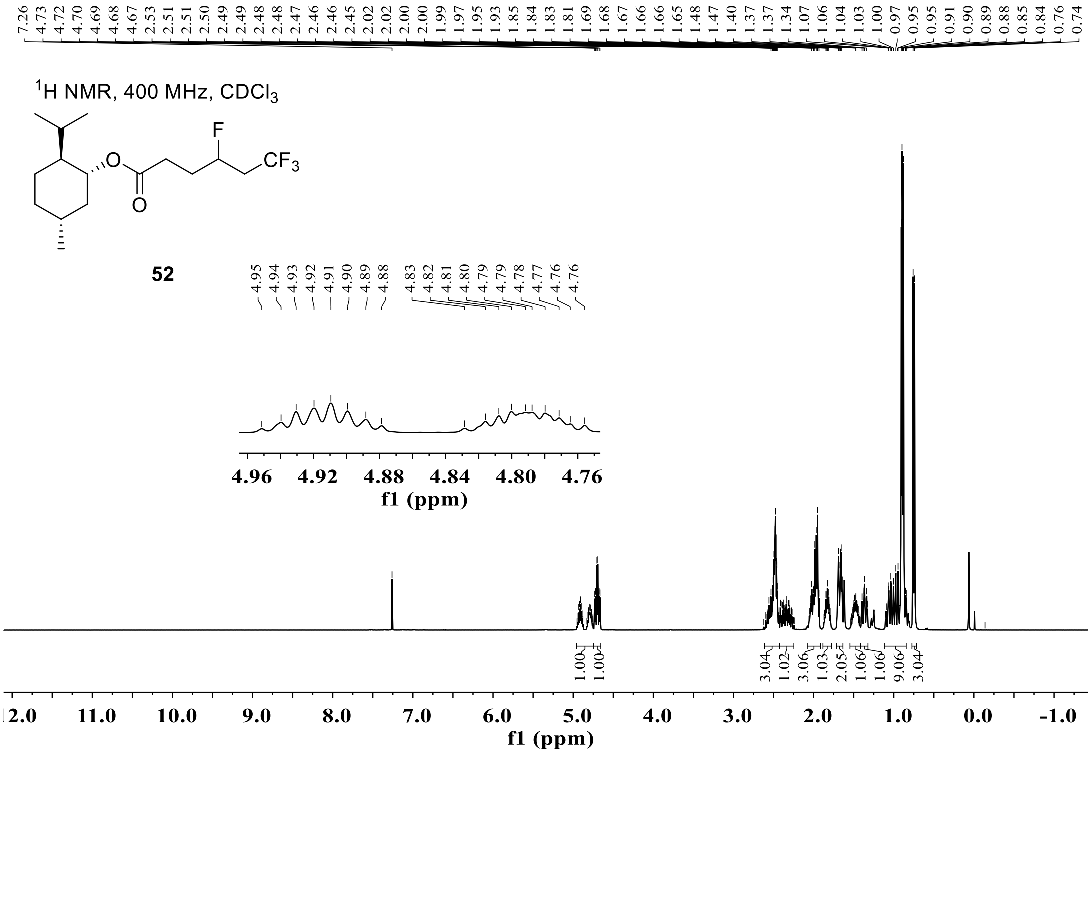

7.26 4.73 4.72 4.70 4.69 4.68 4.67 2.53 2.51 2.51 2.50 2.49 2.49 2.48 2.48 2.47 2.46 2.46 2.45 2.02 2.02 2.00 1.99 1.97 1.95 1.93 1.85 1.84 1.83 1.81 1.69 1.68 1.67 1.66 1.66 1.65 1.48 1.47 1.40 1.37 1.37 1.34 1.07 1.06 1.04 1.03 1.00 0.97 0.95 0.95 0.91 0.90 0.89 0.88 0.85 0.84 0.76 0.74
1H NMR, 400 MHz, CDCl3
F
O
CF3
52
4.95 4.94 4.93 4.92 4.91 4.90 4.89 4.88 4.83 4.82 4.81 4.80 4.79 4.79 4.78 4.77 4.76 4.76
4.96 4.92 4.88 4.84 4.80 4.76
f1 (ppm)
1.00 1.00 3.04 1.02 3.06 1.03 2.05 1.06 1.06 9.06 3.04
2.0 11.0 10.0 9.0 8.0 7.0 6.0 5.0 4.0 3.0 2.0 1.0 0.0 -1.0
f1 (ppm)

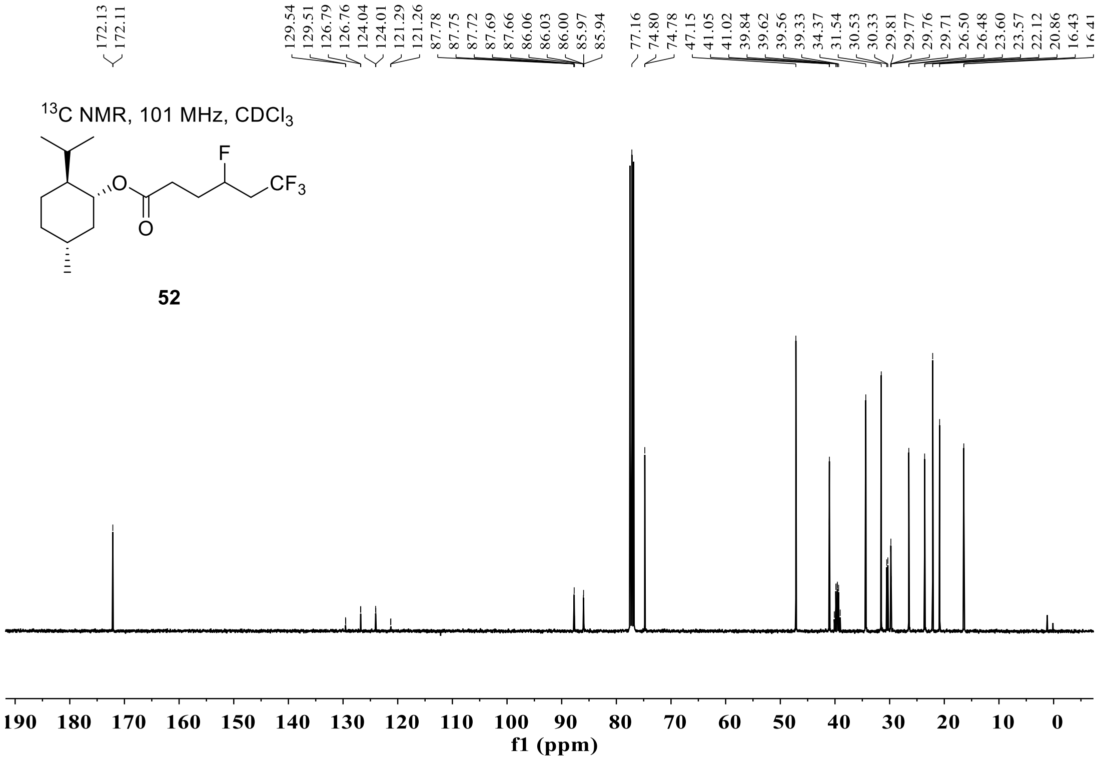
172.13
172.11
129.54
129.51
126.79
126.76
124.04
124.01
121.29
121.26
87.78
87.75
87.72
87.69
87.66
86.06
86.03
86.00
85.97
85.94
77.16
74.80
74.78
47.15
41.05
41.02
39.84
39.62
39.56
39.33
34.37
31.54
30.53
30.33
29.81
29.77
29.76
29.71
26.50
26.48
23.60
23.57
22.12
20.86
16.43
16.41
13C NMR, 101 MHz, CDCl3
F
O
CF3
O
52
190 180 170 160 150 140 130 120 110 100 90 80 70 60 50 40 30 20 10 0
f1 (ppm)

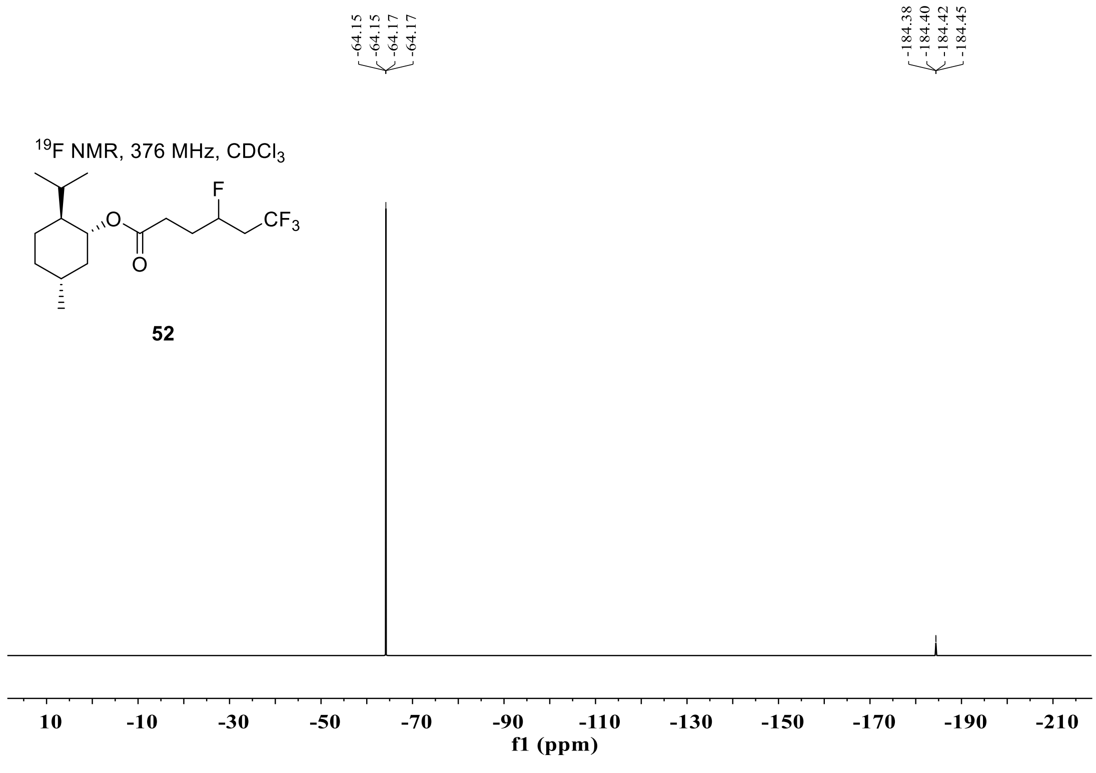
-64.15
-64.15
-64.17
-64.17
-184.38
-184.40
-184.42
-184.45
19F NMR, 376 MHz, CDCl3
F
O
CF3
O
52
10 -10 -30 -50 -70 -90 -110 -130 -150 -170 -190 -210
f1 (ppm)

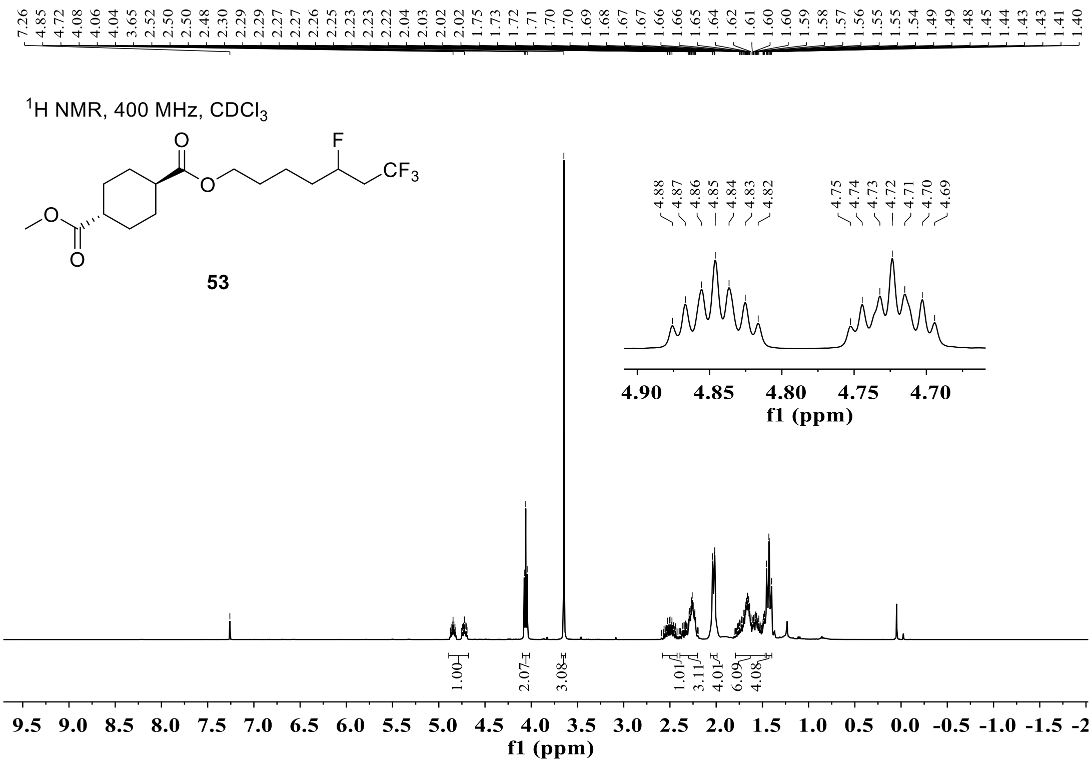
7.26 4.85 4.72 4.08 4.06 4.04 3.65 2.52 2.50 2.50 2.48 2.30 2.29 2.29 2.27 2.27 2.26 2.25 2.23 2.23 2.22 2.04 2.03 2.02 2.02 1.75 1.73 1.72 1.71 1.70 1.70 1.69 1.68 1.67 1.67 1.66 1.66 1.65 1.64 1.62 1.61 1.60 1.60 1.59 1.58 1.57 1.56 1.55 1.55 1.54 1.49 1.49 1.48 1.45 1.44 1.43 1.43 1.41 1.40
¹H NMR, 400 MHz, CDCl₃
53
4.88 4.87 4.86 4.85 4.84 4.83 4.82
4.75 4.74 4.73 4.72 4.71 4.70 4.69
4.90 4.85 4.80 4.75 4.70
f1 (ppm)
1.00 2.07 3.08 1.01 3.11 4.01 6.09 4.08
9.5 9.0 8.5 8.0 7.5 7.0 6.5 6.0 5.5 5.0 4.5 4.0 3.5 3.0 2.5 2.0 1.5 1.0 0.5 0.0 -0.5 -1.0 -1.5 -2
f1 (ppm)

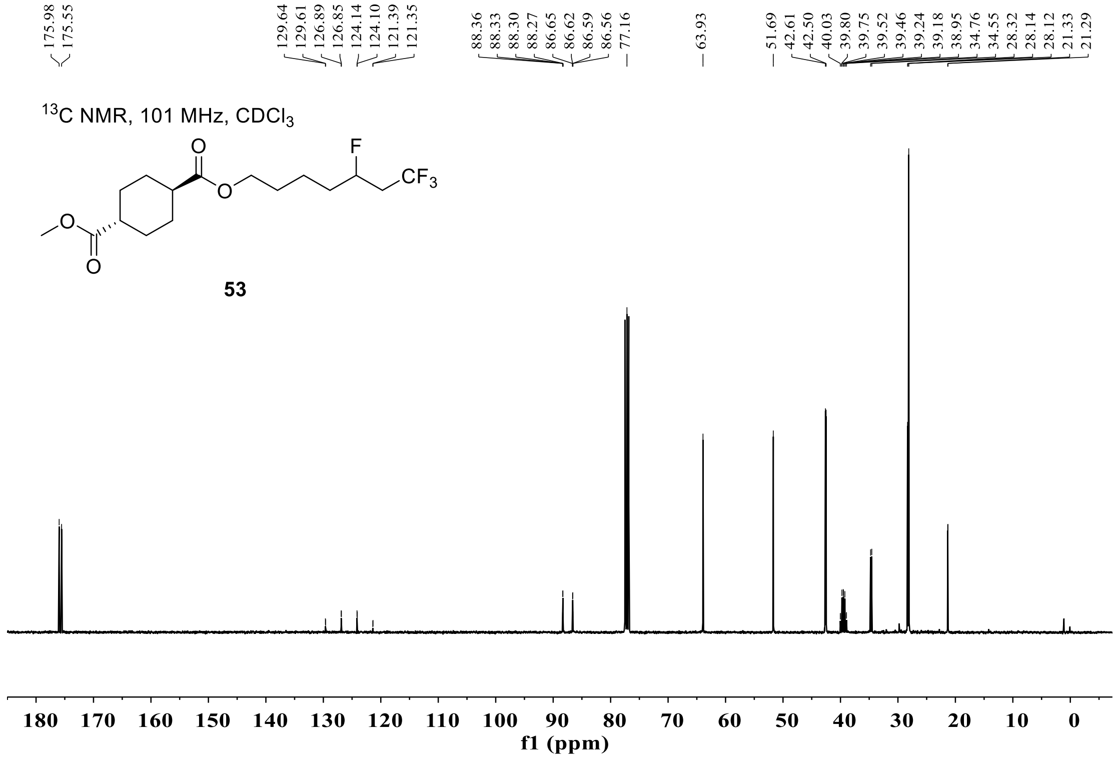
175.98 175.55 129.64 129.61 126.89 126.85 124.14 124.10 121.39 121.35 88.36 88.33 88.30 88.27 86.65 86.62 86.59 86.56 77.16 63.93 51.69 42.61 42.50 40.03 39.80 39.75 39.52 39.46 39.24 39.18 38.95 34.76 34.55 28.32 28.14 28.12 21.33 21.29
¹³C NMR, 101 MHz, CDCl₃
53
180 170 160 150 140 130 120 110 100 90 80 70 60 50 40 30 20 10 0
f1 (ppm)

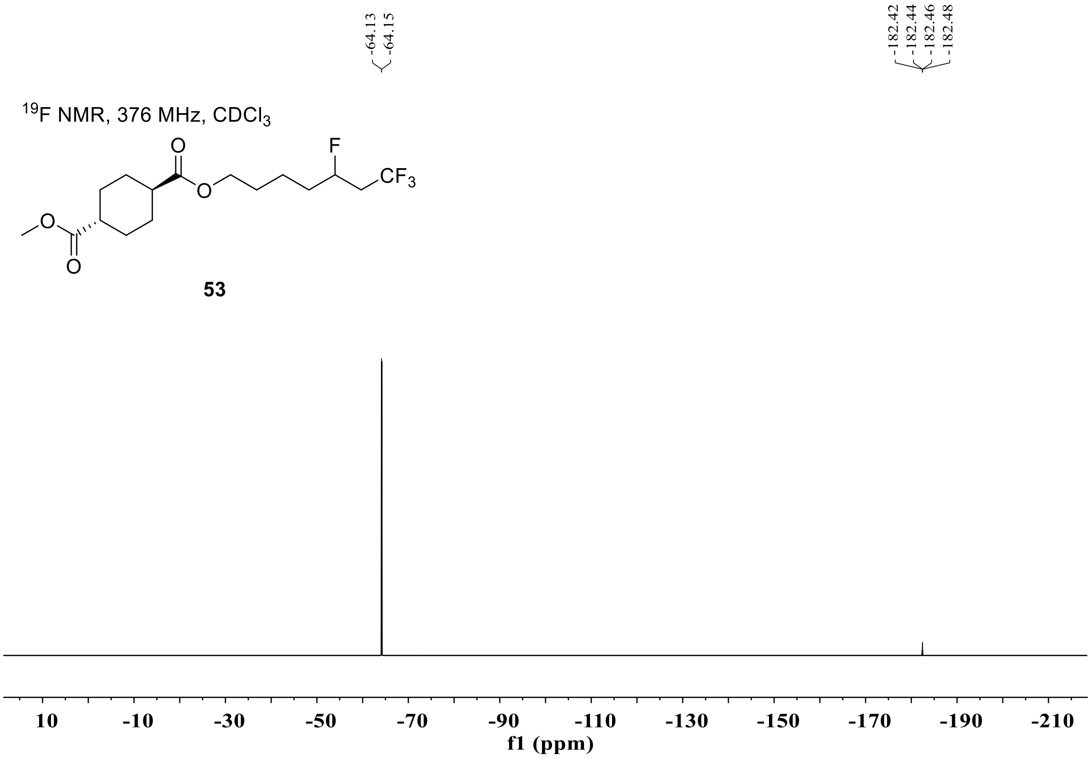
-64.13
-64.15
-182.42
-182.44
-182.46
-182.48
19F NMR, 376 MHz, CDCl3
53
10 -10 -30 -50 -70 -90 -110 -130 -150 -170 -190 -210
f1 (ppm)

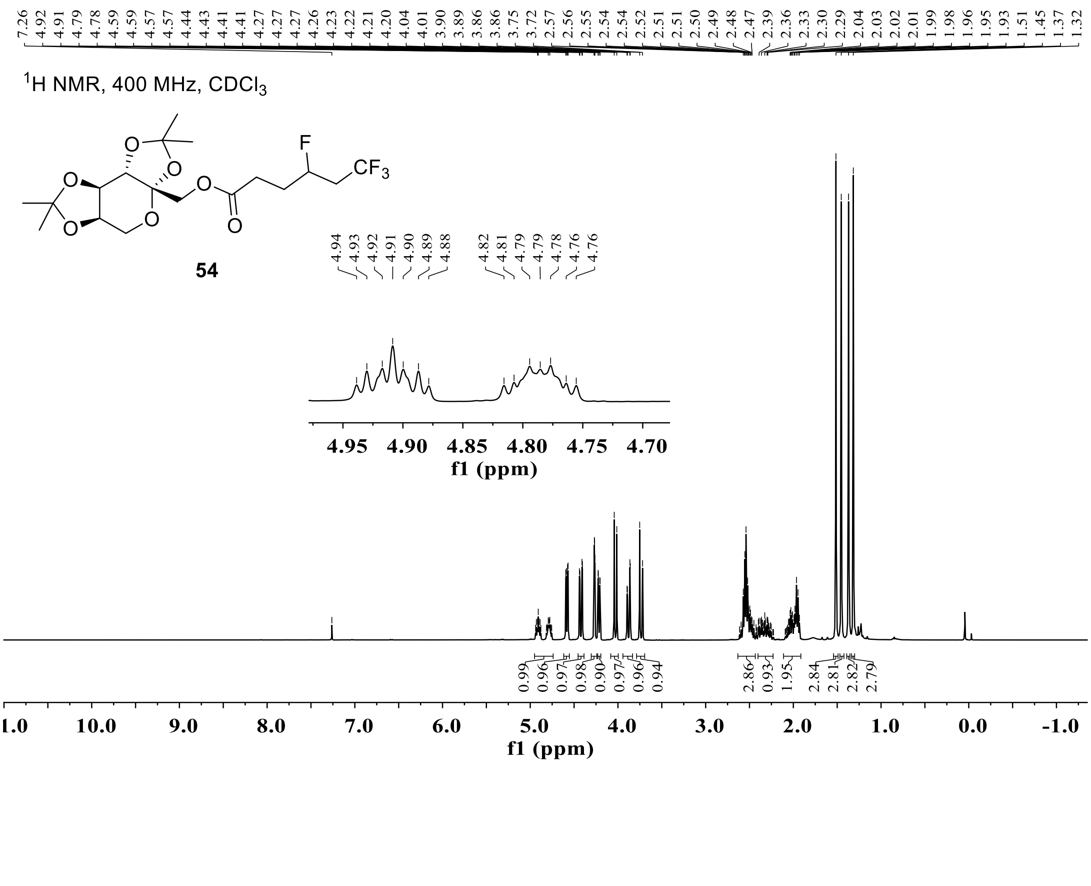
1H NMR, 400 MHz, CDCl3
54
4.95 4.90 4.85 4.80 4.75 4.70
f1 (ppm)
11.0 10.0 9.0 8.0 7.0 6.0 5.0 4.0 3.0 2.0 1.0 0.0 -1.0
f1 (ppm)

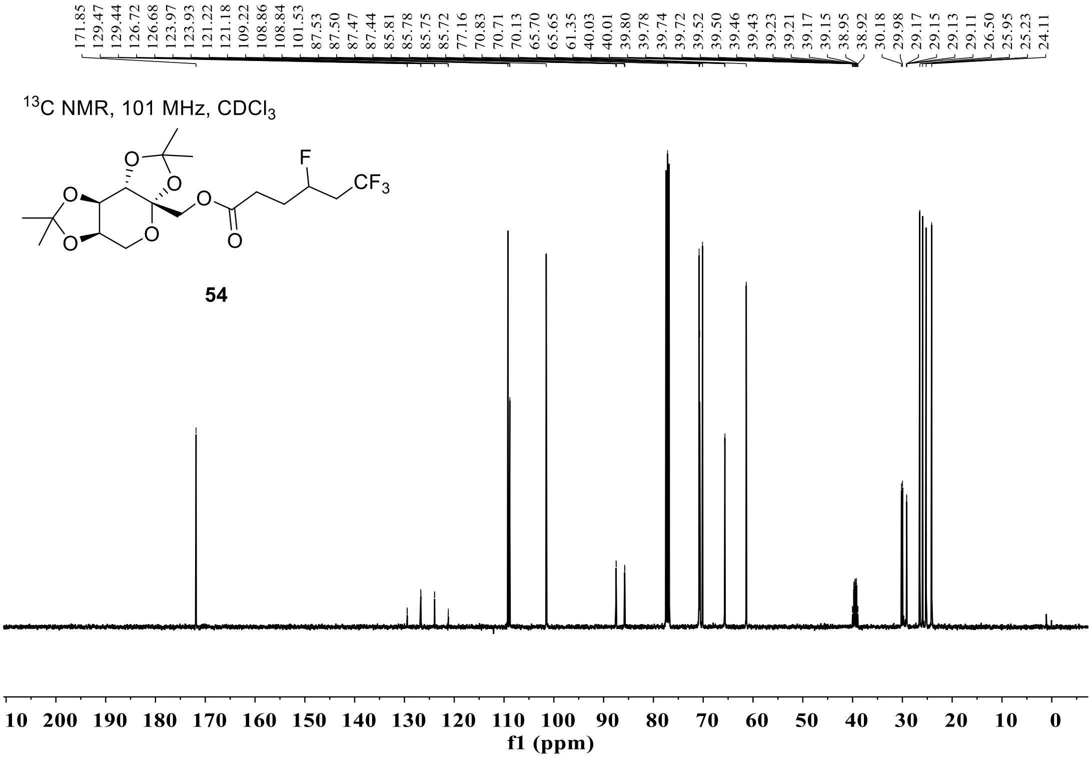
171.85
129.47
129.44
126.72
126.68
123.97
123.93
121.22
121.18
109.22
108.86
108.84
101.53
87.53
87.50
87.47
87.44
85.81
85.78
85.75
85.72
77.16
70.83
70.71
70.13
65.70
65.65
61.35
40.03
40.01
39.80
39.78
39.74
39.72
39.52
39.50
39.46
39.43
39.23
39.21
39.17
39.15
38.95
38.92
30.18
29.98
29.17
29.15
29.13
29.11
26.50
25.95
25.23
24.11
^{13}C NMR, 101 MHz, CDCl$_3$
F
CF$_3$
54
10 200 190 180 170 160 150 140 130 120 110 100 90 80 70 60 50 40 30 20 10 0
f1 (ppm)

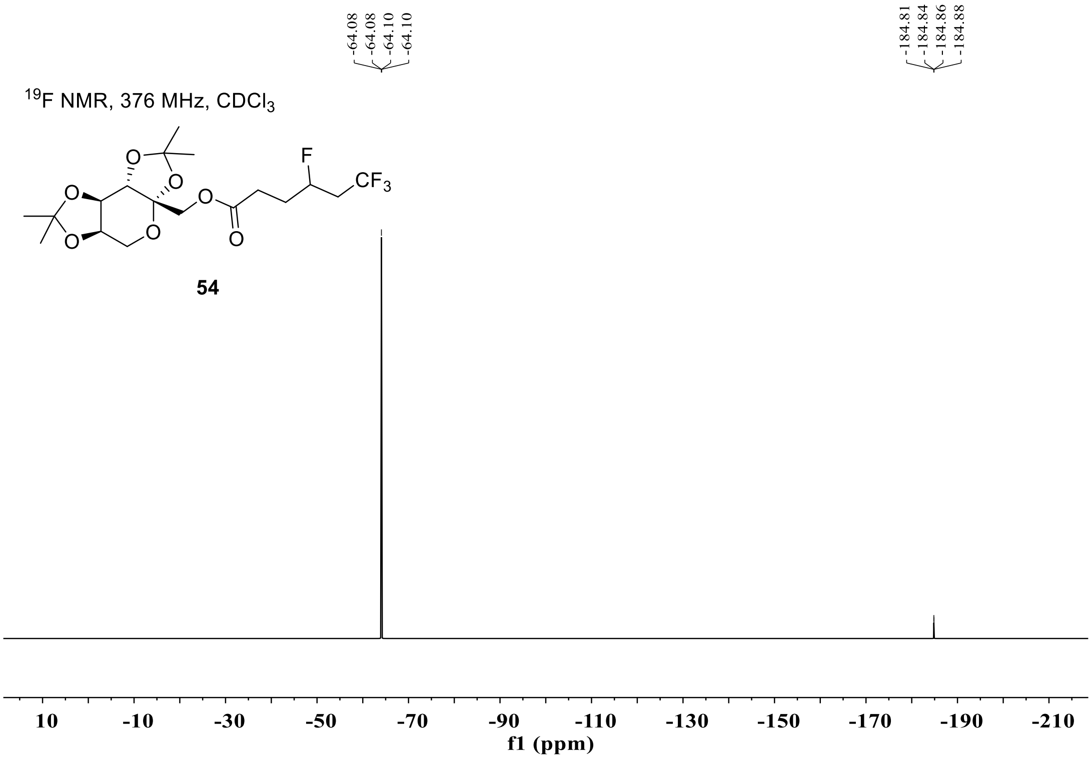
-64.08
-64.08
-64.10
-64.10
-184.81
-184.84
-184.86
-184.88
^{19}F NMR, 376 MHz, CDCl$_3$
F
CF$_3$
54
10 -10 -30 -50 -70 -90 -110 -130 -150 -170 -190 -210
f1 (ppm)

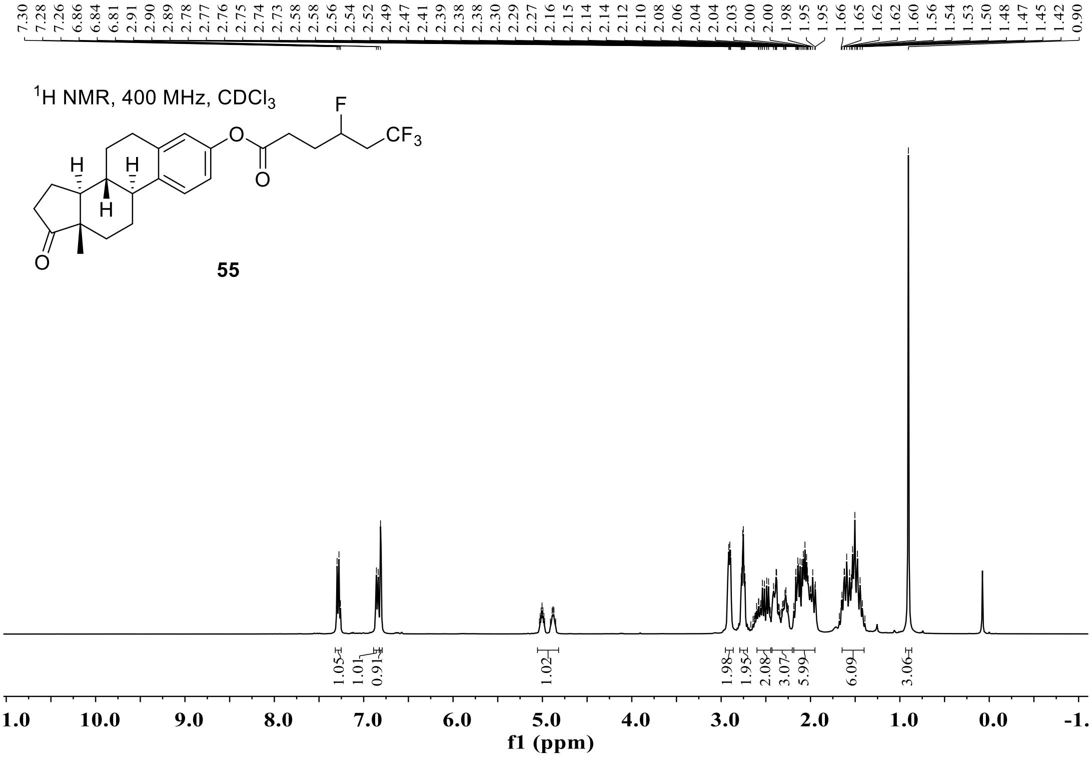
7.30 7.28 7.26 6.86 6.84 6.81 2.91 2.90 2.89 2.78 2.77 2.76 2.75 2.74 2.73 2.58 2.58 2.56 2.54 2.52 2.49 2.47 2.41 2.39 2.38 2.38 2.30 2.29 2.27 2.16 2.15 2.14 2.14 2.12 2.10 2.08 2.06 2.04 2.04 2.03 2.00 2.00 1.98 1.95 1.95 1.66 1.65 1.62 1.60 1.56 1.54 1.53 1.50 1.48 1.47 1.45 1.42 0.90
1H NMR, 400 MHz, CDCl3
F
CF3
O
H
55
1.05 1.01 0.91 1.02 1.98 1.95 2.08 3.07 5.99 6.09 3.06
1.0 10.0 9.0 8.0 7.0 6.0 5.0 4.0 3.0 2.0 1.0 0.0 -1.
f1 (ppm)

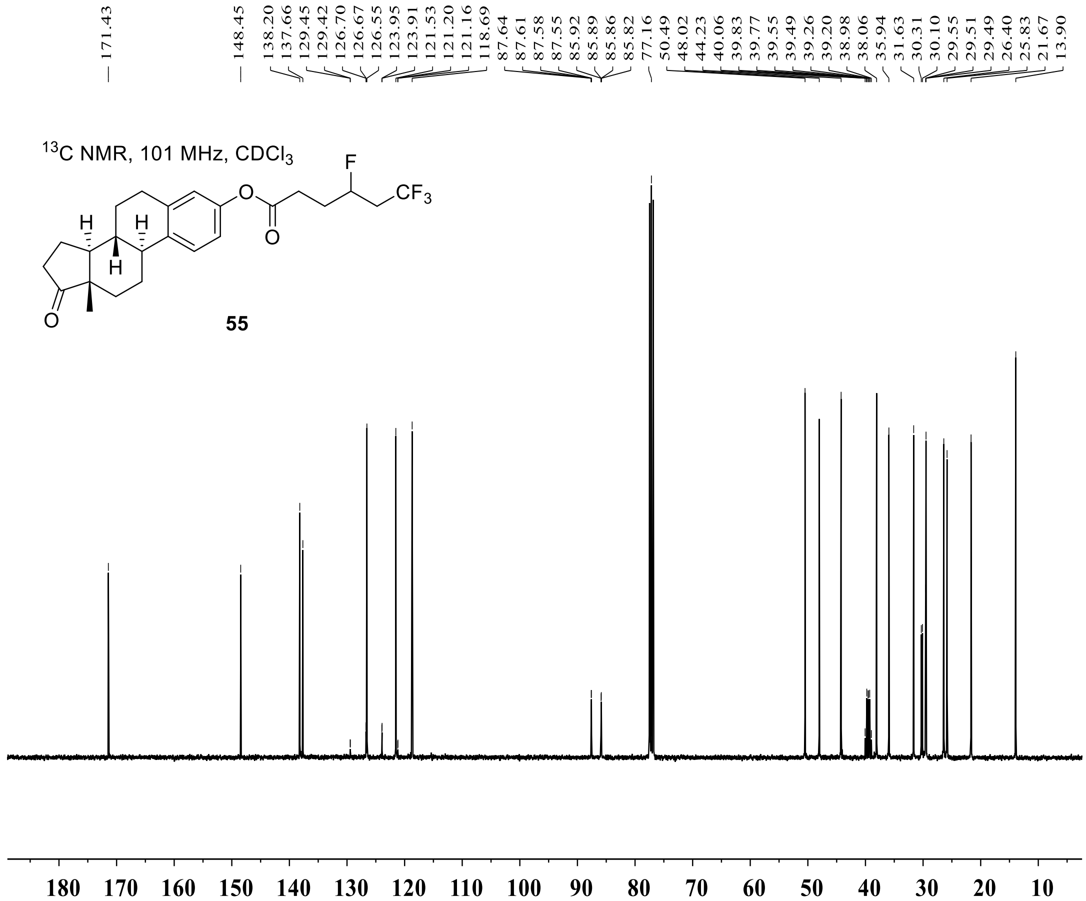
171.43 148.45 138.20 137.66 129.45 129.42 126.70 126.67 126.55 123.95 123.91 121.53 121.20 121.16 118.69 87.64 87.61 87.58 87.55 85.92 85.89 85.86 85.82 77.16 50.49 48.02 44.23 40.06 39.83 39.77 39.55 39.49 39.26 39.20 38.98 38.06 35.94 31.63 30.31 30.10 29.55 29.51 29.49 26.40 25.83 21.67 13.90
13C NMR, 101 MHz, CDCl3
F
CF3
O
H
55
180 170 160 150 140 130 120 110 100 90 80 70 60 50 40 30 20 10
f1 (ppm)

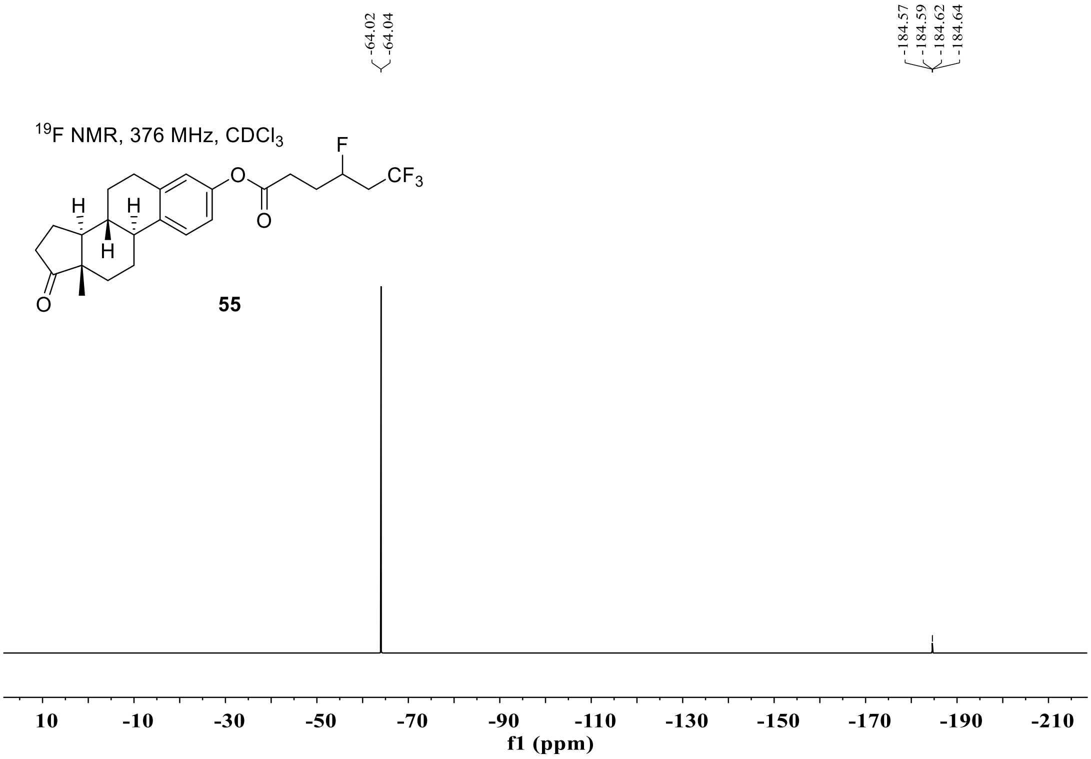
-64.02
-64.04
-184.57
-184.59
-184.62
-184.64
^{19}F NMR, 376 MHz, CDCl$_3$
F
O
CF$_3$
H
H
H
O
O
55
10
-10
-30
-50
-70
-90
-110
-130
-150
-170
-190
-210
f1 (ppm)

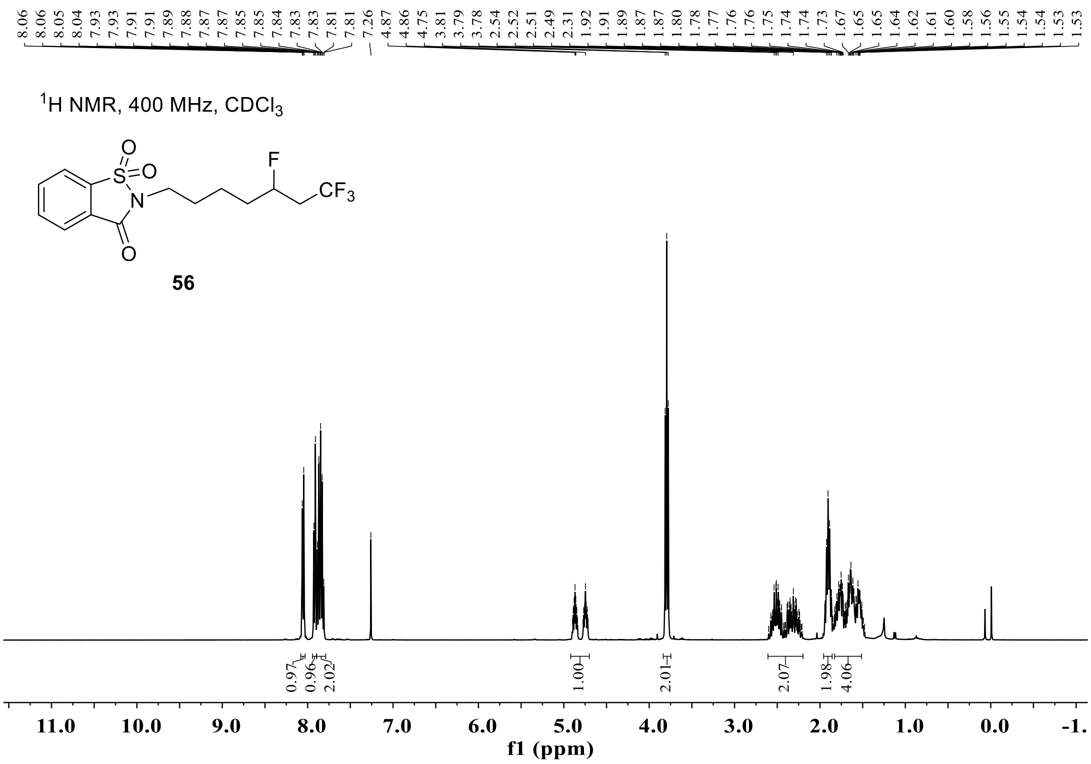
8.06 8.06 8.05 8.04 7.93 7.93 7.91 7.91 7.89 7.88 7.87 7.87 7.85 7.85 7.84 7.83 7.83 7.81 7.81 7.26 4.87 4.86 4.75 3.81 3.79 3.78 2.54 2.52 2.51 2.49 2.31 1.92 1.91 1.89 1.87 1.87 1.80 1.78 1.77 1.76 1.76 1.75 1.74 1.74 1.73 1.67 1.65 1.65 1.64 1.62 1.61 1.60 1.58 1.56 1.55 1.54 1.54 1.53 1.53
^{1}H NMR, 400 MHz, CDCl$_3$
O
S=O
N
O
F
CF$_3$
56
0.97
0.96
2.02
1.00
2.01
2.07
1.98
4.06
11.0
10.0
9.0
8.0
7.0
6.0
5.0
4.0
3.0
2.0
1.0
0.0
-1.
f1 (ppm)

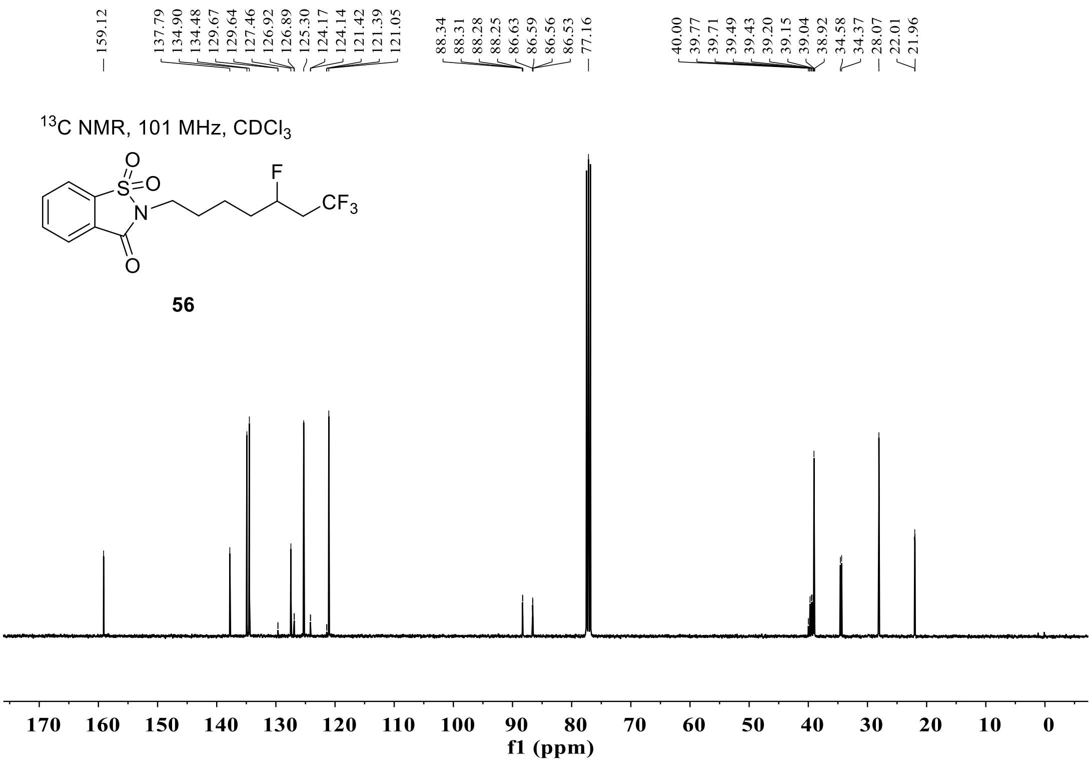
159.12
137.79
134.90
134.48
129.67
129.64
127.46
126.92
126.89
125.30
124.17
124.14
121.42
121.39
121.05
88.34
88.31
88.28
88.25
86.63
86.59
86.56
86.53
77.16
40.00
39.77
39.71
39.49
39.43
39.20
39.15
39.04
38.92
34.58
34.37
28.07
22.01
21.96
13C NMR, 101 MHz, CDCl3
56
170 160 150 140 130 120 110 100 90 80 70 60 50 40 30 20 10 0
f1 (ppm)

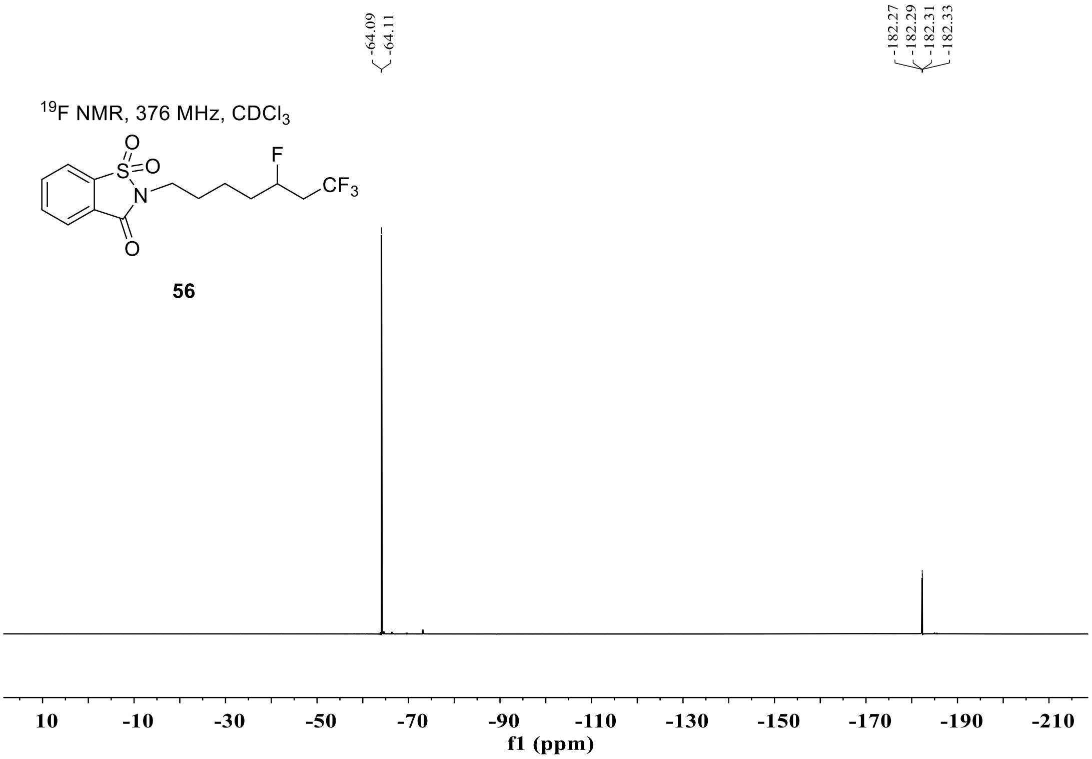
-64.09
-64.11
-182.27
-182.29
-182.31
-182.33
19F NMR, 376 MHz, CDCl3
56
10 -10 -30 -50 -70 -90 -110 -130 -150 -170 -190 -210
f1 (ppm)

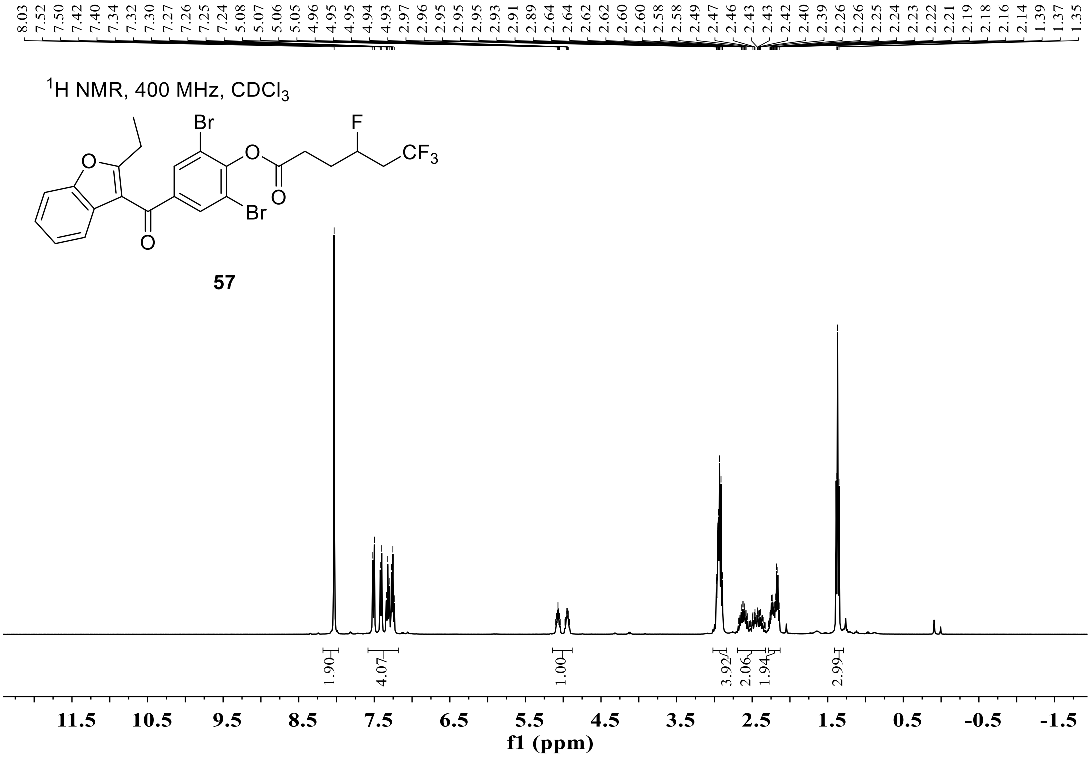

8.03 7.52 7.50 7.42 7.40 7.34 7.32 7.30 7.27 7.26 7.25 7.24 5.08 5.07 5.06 5.05 4.96 4.95 4.95 4.94 4.93 2.97 2.96 2.95 2.95 2.95 2.93 2.91 2.89 2.64 2.64 2.62 2.62 2.60 2.60 2.58 2.58 2.49 2.47 2.46 2.43 2.43 2.42 2.40 2.39 2.26 2.26 2.25 2.24 2.23 2.22 2.21 2.19 2.18 2.16 2.14 1.39 1.37 1.35
^{1}H NMR, 400 MHz, CDCl$_3$
Br
F
O
CF$_3$
O
Br
O
57
1.90
4.07
1.00
3.92
2.06
1.94
2.99
11.5 10.5 9.5 8.5 7.5 6.5 5.5 4.5 3.5 2.5 1.5 0.5 -0.5 -1.5
f1 (ppm)

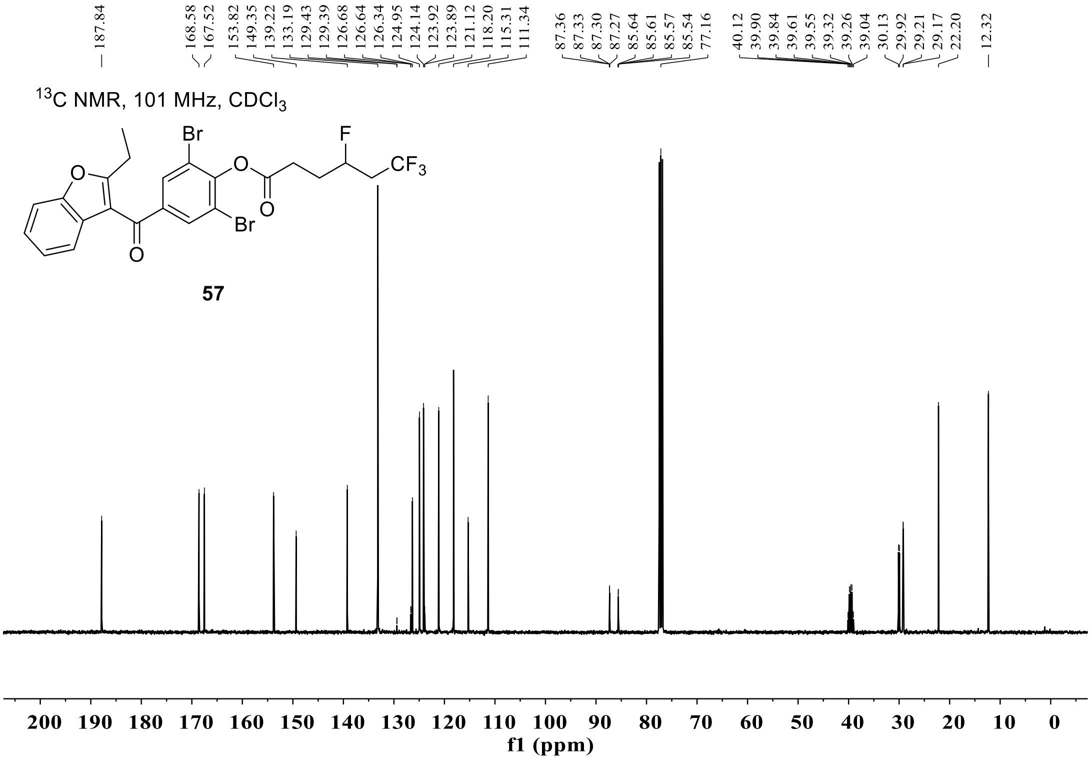

187.84
168.58 167.52
153.82 149.35 139.22 133.19 129.43 129.39 126.68 126.64 126.34 124.95 124.14 123.92 123.89 121.12 118.20 115.31 111.34
87.36 87.33 87.30 87.27 85.64 85.61 85.57 85.54 77.16
40.12 39.90 39.84 39.61 39.55 39.32 39.26 39.04
30.13 29.92 29.21 29.17 22.20
12.32
^{13}C NMR, 101 MHz, CDCl$_3$
Br
F
O
CF$_3$
O
Br
O
57
200 190 180 170 160 150 140 130 120 110 100 90 80 70 60 50 40 30 20 10 0
f1 (ppm)

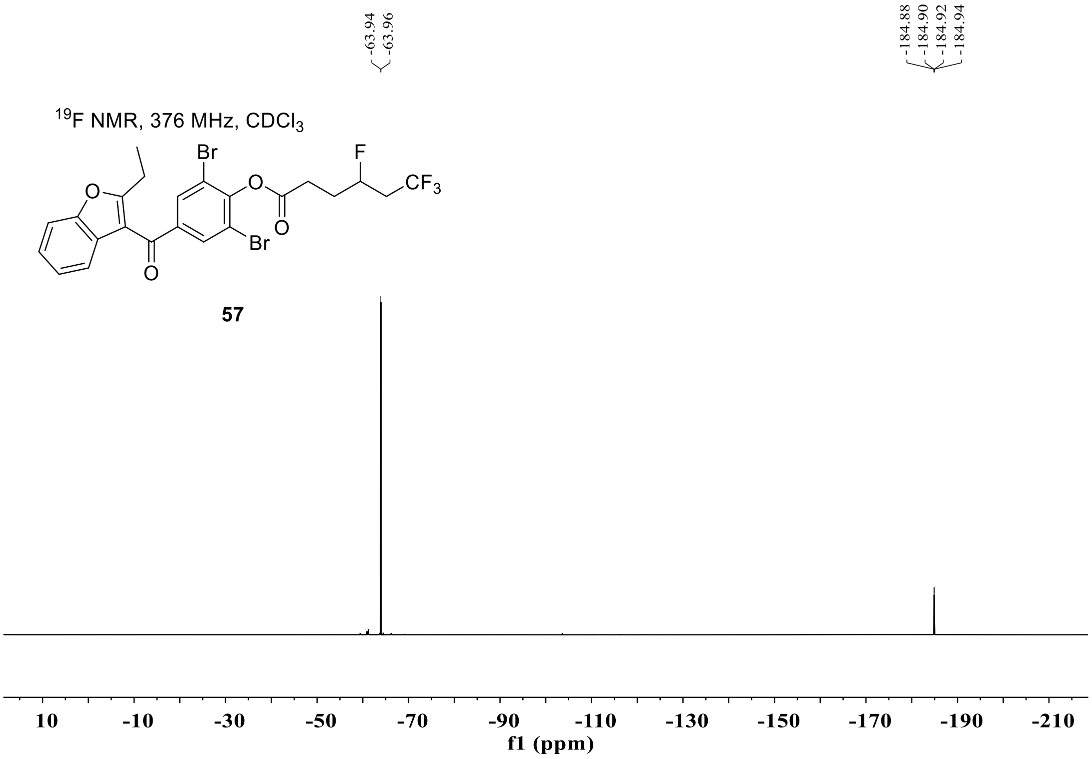

-63.94
-63.96
-184.88
-184.90
-184.92
-184.94
19F NMR, 376 MHz, CDCl3
Br
O
F
CF3
O
O
Br
O
57
10 -10 -30 -50 -70 -90 -110 -130 -150 -170 -190 -210
f1 (ppm)

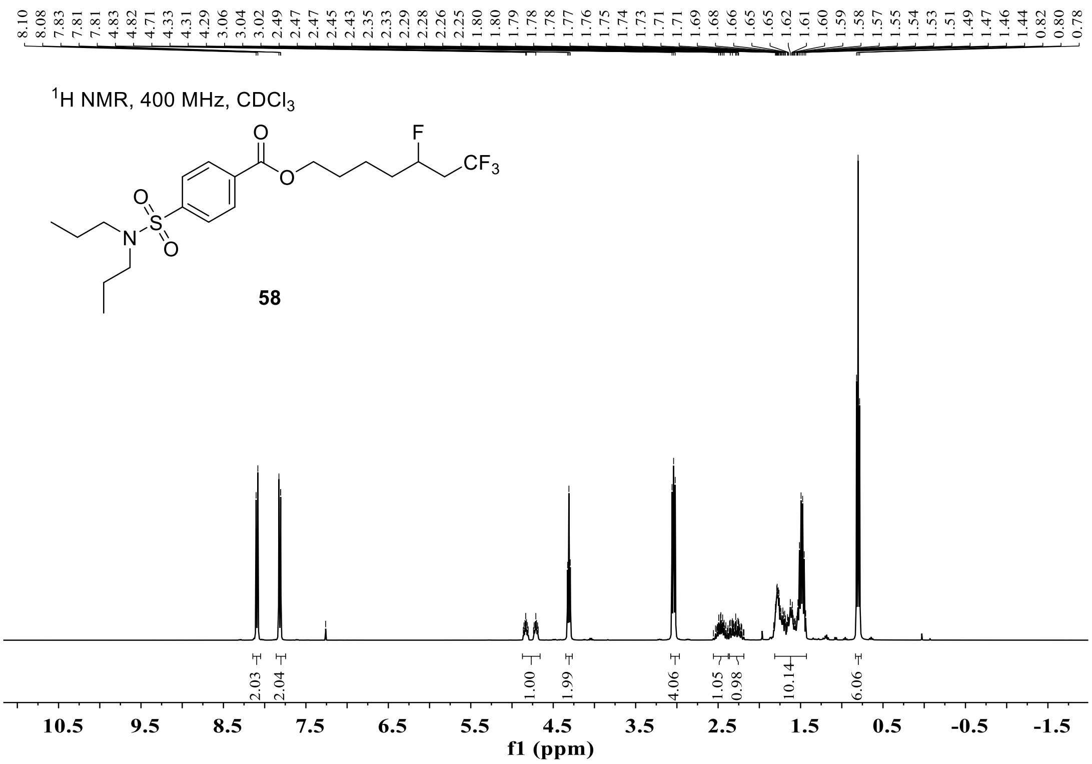

8.10 8.08 7.83 7.81 4.83 4.82 4.71 4.33 4.31 4.29 3.06 3.04 3.02 2.49 2.47 2.45 2.43 2.35 2.33 2.29 2.28 2.26 2.25 1.80 1.80 1.79 1.78 1.78 1.77 1.76 1.75 1.74 1.73 1.71 1.71 1.69 1.68 1.66 1.65 1.65 1.62 1.61 1.60 1.59 1.58 1.57 1.55 1.54 1.53 1.51 1.49 1.47 1.46 1.44 0.82 0.80 0.78
1H NMR, 400 MHz, CDCl3
O
F
CF3
O
O
S
N
O
58
2.03 2.04 1.00 1.99 4.06 1.05 0.98 10.14 6.06
10.5 9.5 8.5 7.5 6.5 5.5 4.5 3.5 2.5 1.5 0.5 -0.5 -1.5
f1 (ppm)

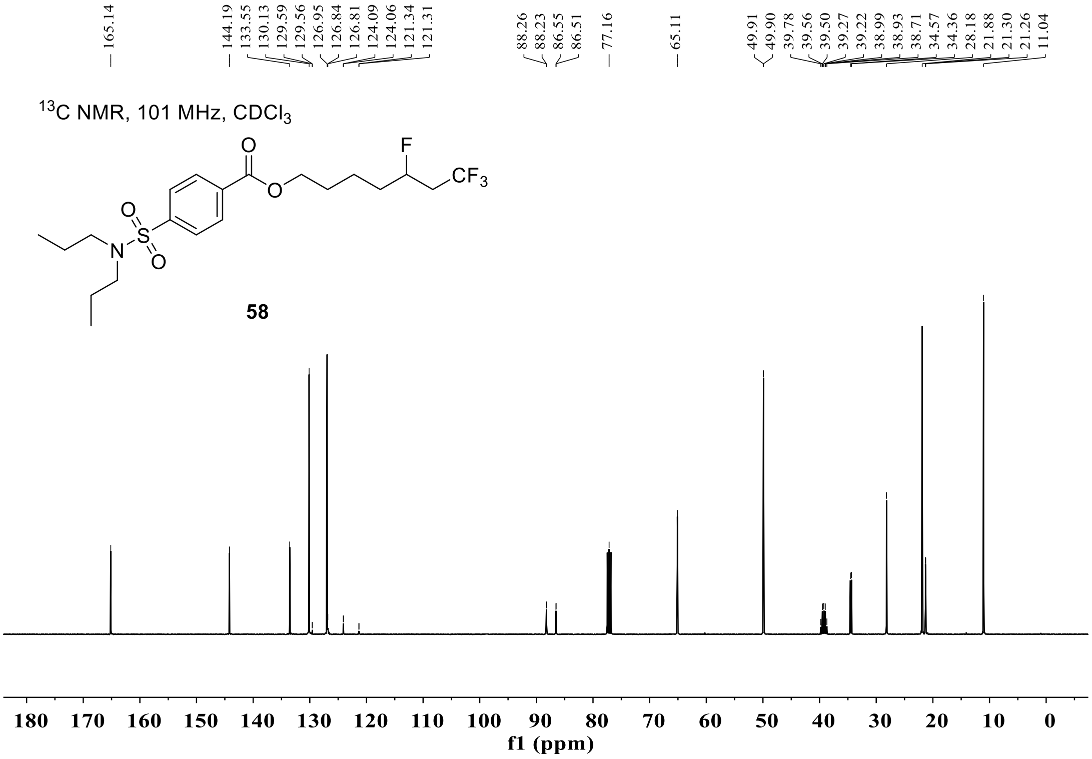

165.14
144.19
133.55
130.13
129.59
129.56
126.95
126.84
126.81
124.09
124.06
121.34
121.31
88.26
88.23
86.55
86.51
77.16
65.11
49.91
49.90
39.78
39.56
39.50
39.27
39.22
38.99
38.93
38.71
34.57
34.36
28.18
21.88
21.30
21.26
11.04
^{13}C NMR, 101 MHz, CDCl$_3$
O
F
O
CF$_3$
O
S
N
O
58
180 170 160 150 140 130 120 110 100 90 80 70 60 50 40 30 20 10 0
f1 (ppm)

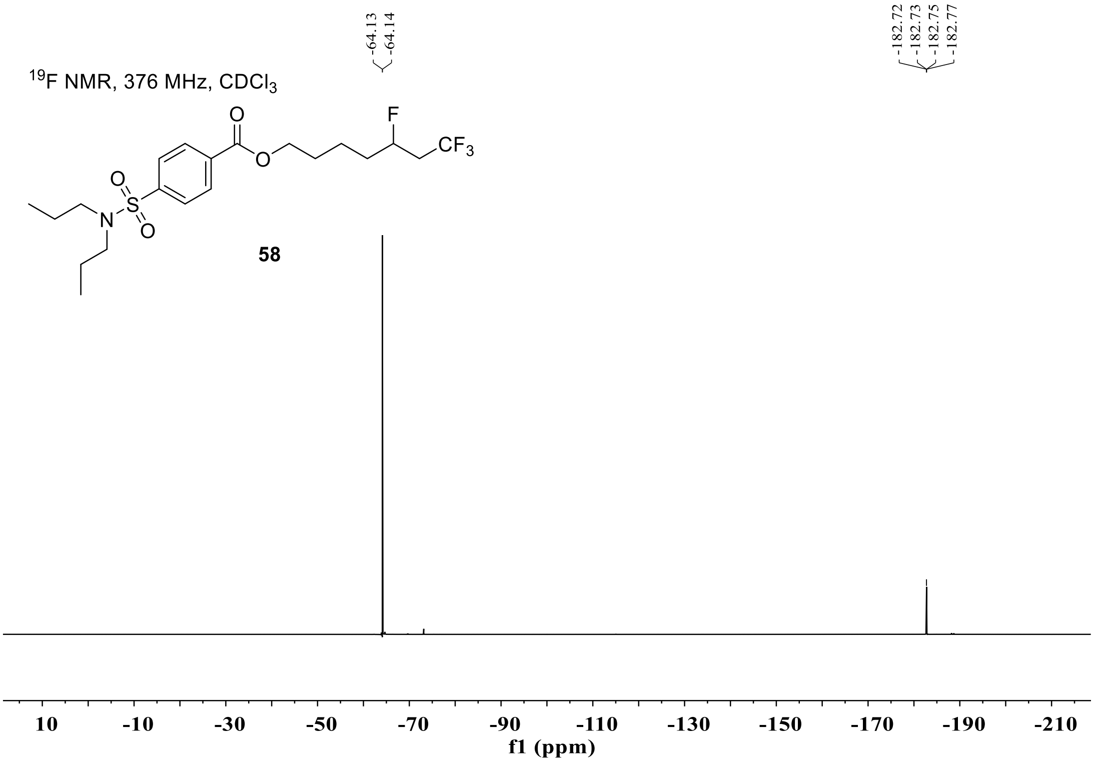

-64.13
-64.14
-182.72
-182.73
-182.75
-182.77
^{19}F NMR, 376 MHz, CDCl$_3$
O
F
O
CF$_3$
O
S
N
O
58
10 -10 -30 -50 -70 -90 -110 -130 -150 -170 -190 -210
f1 (ppm)

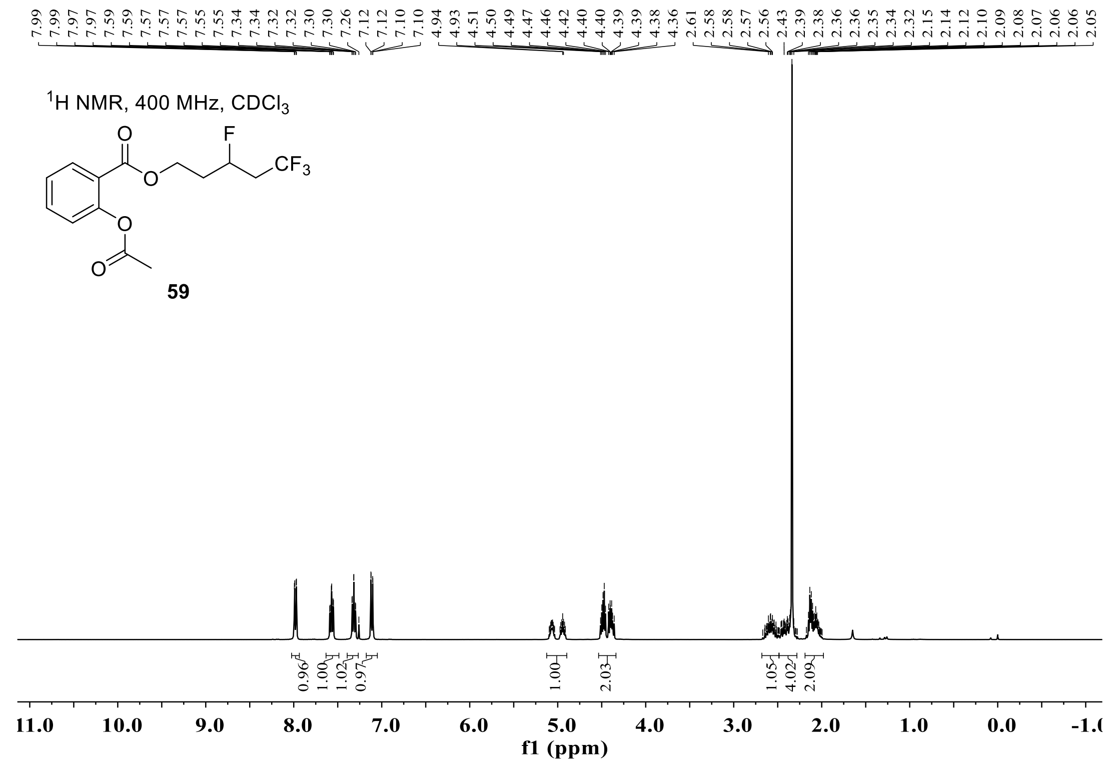
7.99 7.99 7.97 7.97 7.59 7.59 7.57 7.57 7.57 7.55 7.55 7.34 7.34 7.32 7.32 7.30 7.30 7.26 7.12 7.12 7.10 7.10 4.94 4.93 4.51 4.50 4.49 4.47 4.46 4.42 4.40 4.40 4.39 4.39 4.38 4.36 2.61 2.58 2.58 2.57 2.56 2.43 2.39 2.38 2.36 2.36 2.35 2.34 2.32 2.15 2.14 2.12 2.10 2.09 2.08 2.07 2.06 2.06 2.05
1H NMR, 400 MHz, CDCl3
O
F
O
CF3
O
O
59
0.96
1.00
1.02
0.97
1.00
2.03
1.05
4.02
2.09
11.0 10.0 9.0 8.0 7.0 6.0 5.0 4.0 3.0 2.0 1.0 0.0
f1 (ppm)

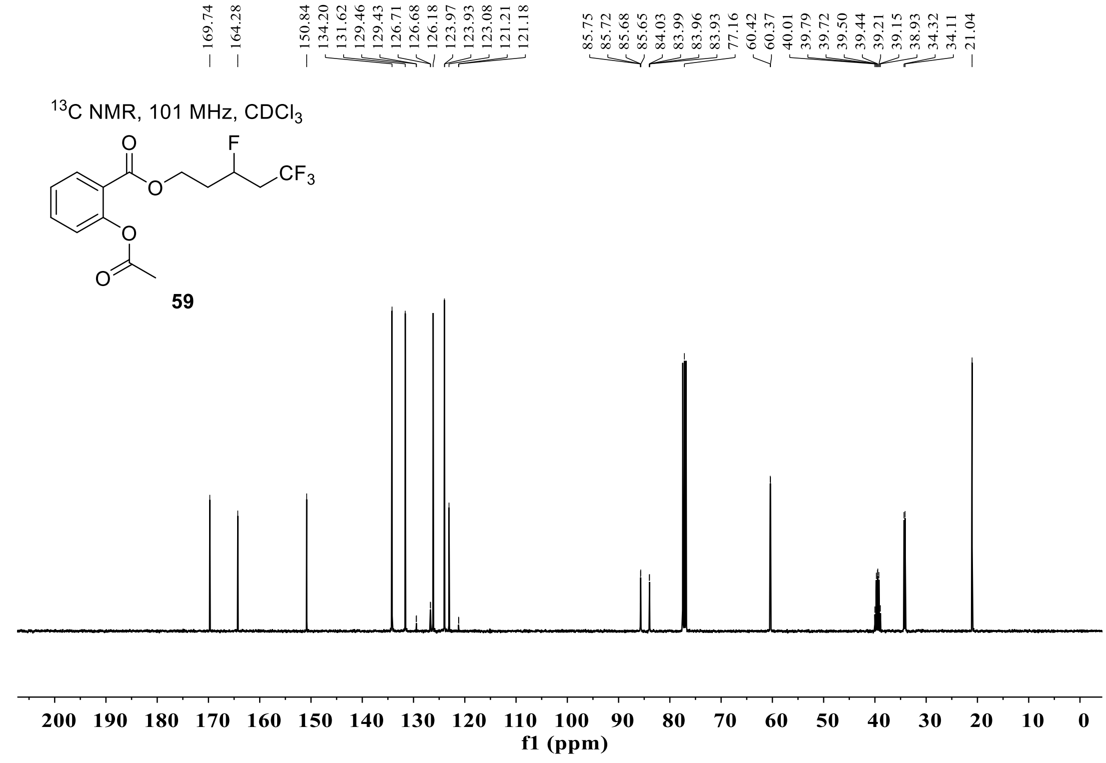
169.74 164.28 150.84 134.20 131.62 129.46 129.43 126.71 126.68 126.18 123.97 123.93 123.08 121.21 121.18 85.75 85.72 85.68 85.65 84.03 83.99 83.96 83.93 77.16 60.42 60.37 40.01 39.79 39.72 39.50 39.44 39.21 39.15 38.93 34.32 34.11 21.04
13C NMR, 101 MHz, CDCl3
O
F
O
CF3
O
O
59
200 190 180 170 160 150 140 130 120 110 100 90 80 70 60 50 40 30 20 10 0
f1 (ppm)

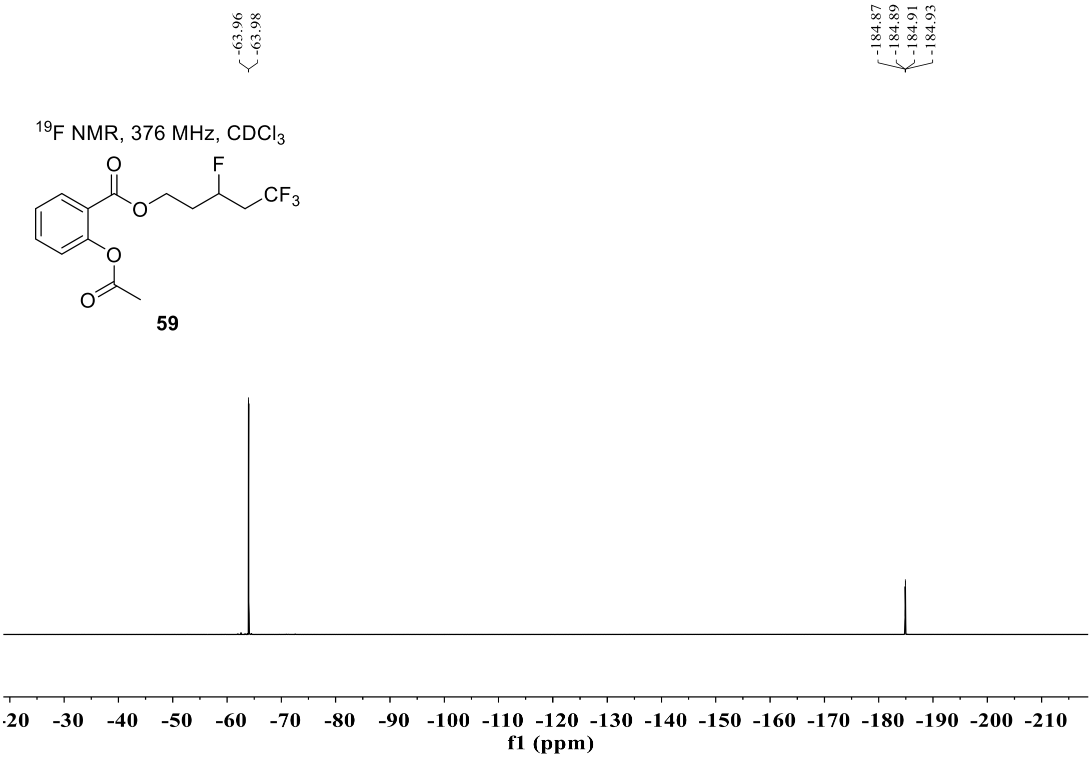
-63.96
-63.98
-184.87
-184.89
-184.91
-184.93
19F NMR, 376 MHz, CDCl3
O
F
O
CF3
O
O
59
-20 -30 -40 -50 -60 -70 -80 -90 -100 -110 -120 -130 -140 -150 -160 -170 -180 -190 -200 -210
f1 (ppm)

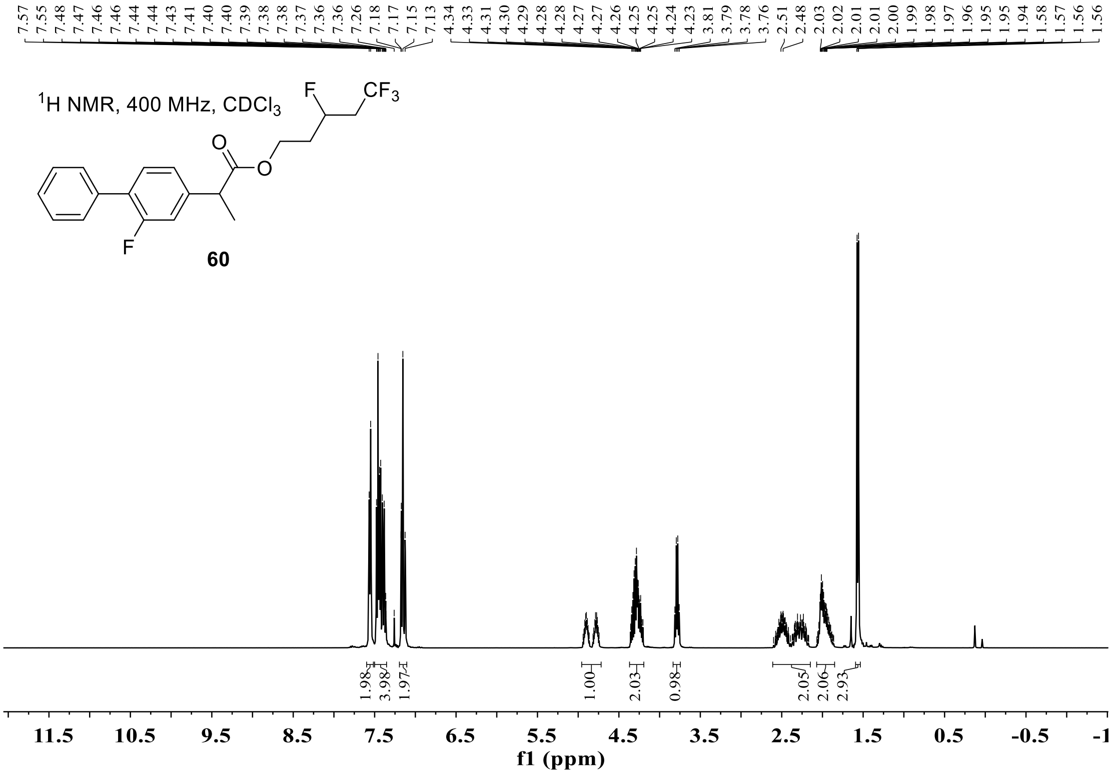
7.57 7.55 7.48 7.47 7.46 7.46 7.44 7.44 7.43 7.41 7.40 7.40 7.39 7.38 7.38 7.37 7.36 7.36 7.26 7.18 7.17 7.15 7.13 4.34 4.33 4.31 4.30 4.29 4.28 4.28 4.27 4.27 4.26 4.25 4.25 4.24 4.23 3.81 3.79 3.78 3.76 2.51 2.48 2.03 2.02 2.01 2.01 2.00 1.99 1.98 1.97 1.96 1.95 1.95 1.94 1.58 1.57 1.56 1.56
1H NMR, 400 MHz, CDCl3
F
CF3
O
O
F
60
1.98
3.98
1.97
1.00
2.03
0.98
2.05
2.06
2.93
11.5 10.5 9.5 8.5 7.5 6.5 5.5 4.5 3.5 2.5 1.5 0.5 -0.5 -1
f1 (ppm)

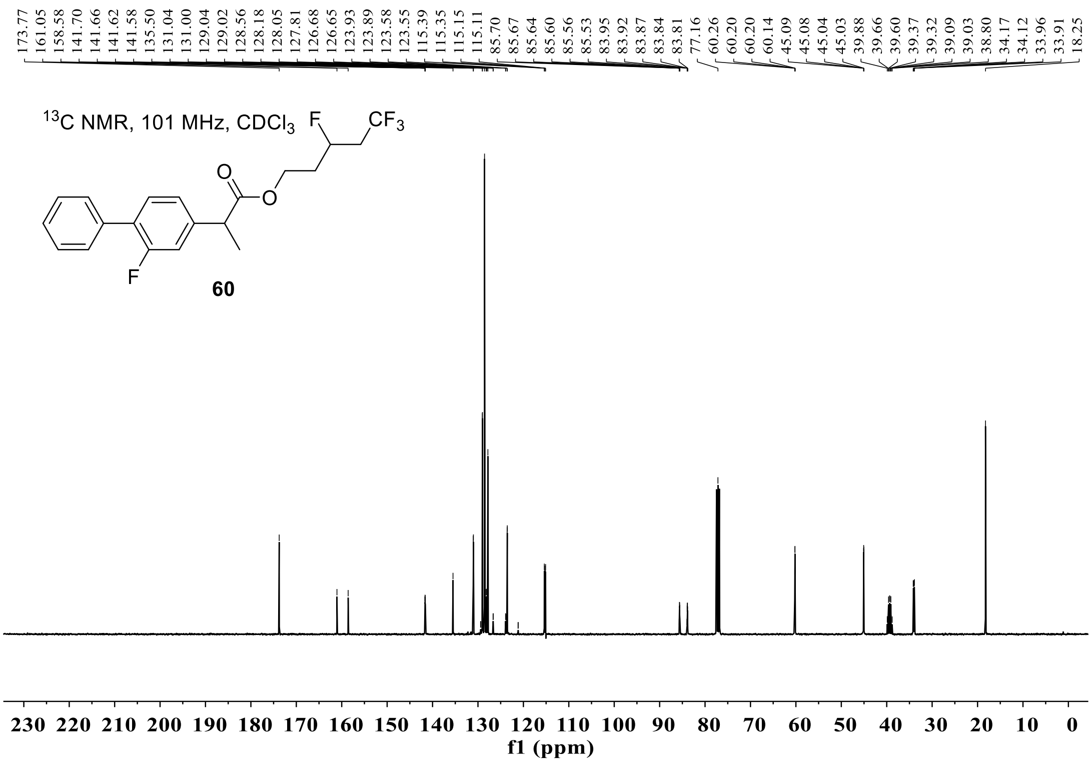
173.77 161.05 158.58 141.70 141.66 141.62 141.58 135.50 131.04 131.00 129.04 129.02 128.56 128.18 128.05 127.81 126.68 126.65 123.93 123.89 123.58 123.55 115.39 115.35 115.15 115.11 85.70 85.67 85.64 85.60 85.56 85.53 83.95 83.92 83.87 83.84 83.81 77.16 60.26 60.20 60.20 60.14 45.09 45.08 45.04 45.03 39.88 39.66 39.60 39.37 39.32 39.09 39.03 38.80 34.17 34.12 33.96 33.91 18.25
^{13}C NMR, 101 MHz, CDCl$_3$
F
CF$_3$
O
O
F
60
230 220 210 200 190 180 170 160 150 140 130 120 110 100 90 80 70 60 50 40 30 20 10 0
f1 (ppm)

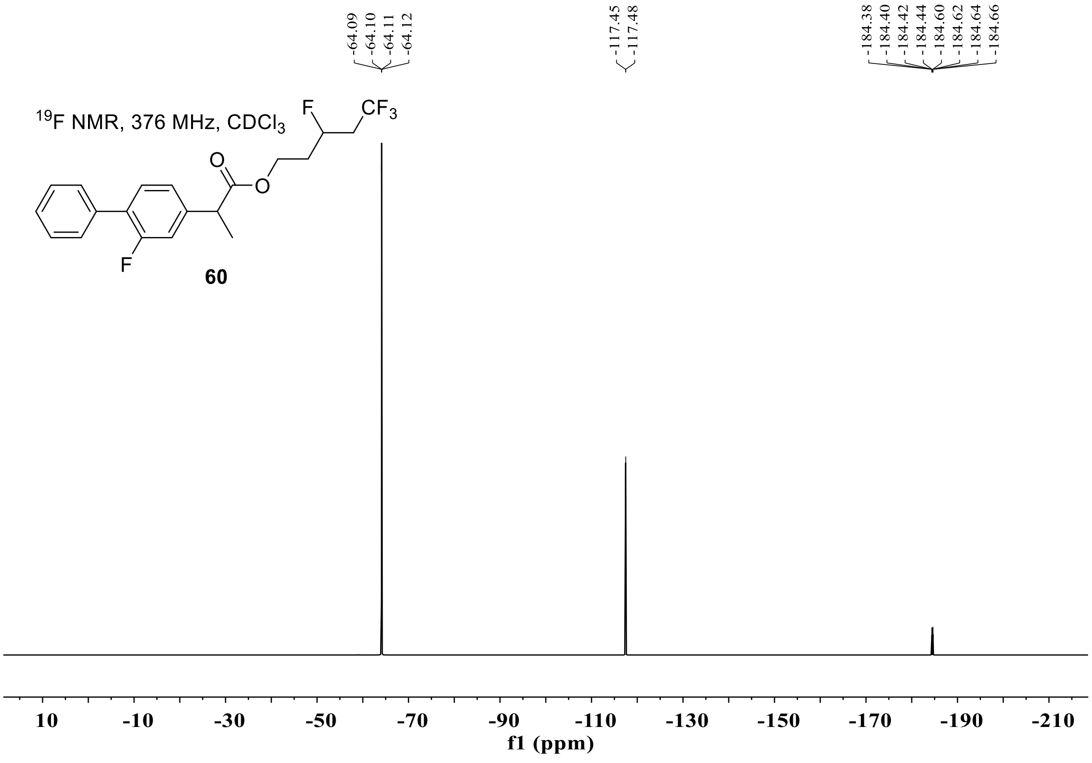
-64.09 -64.10 -64.11 -64.12
-117.45 -117.48
-184.38 -184.40 -184.42 -184.44 -184.60 -184.62 -184.64 -184.66
^{19}F NMR, 376 MHz, CDCl$_3$
F
CF$_3$
O
O
F
60
10 -10 -30 -50 -70 -90 -110 -130 -150 -170 -190 -210
f1 (ppm)

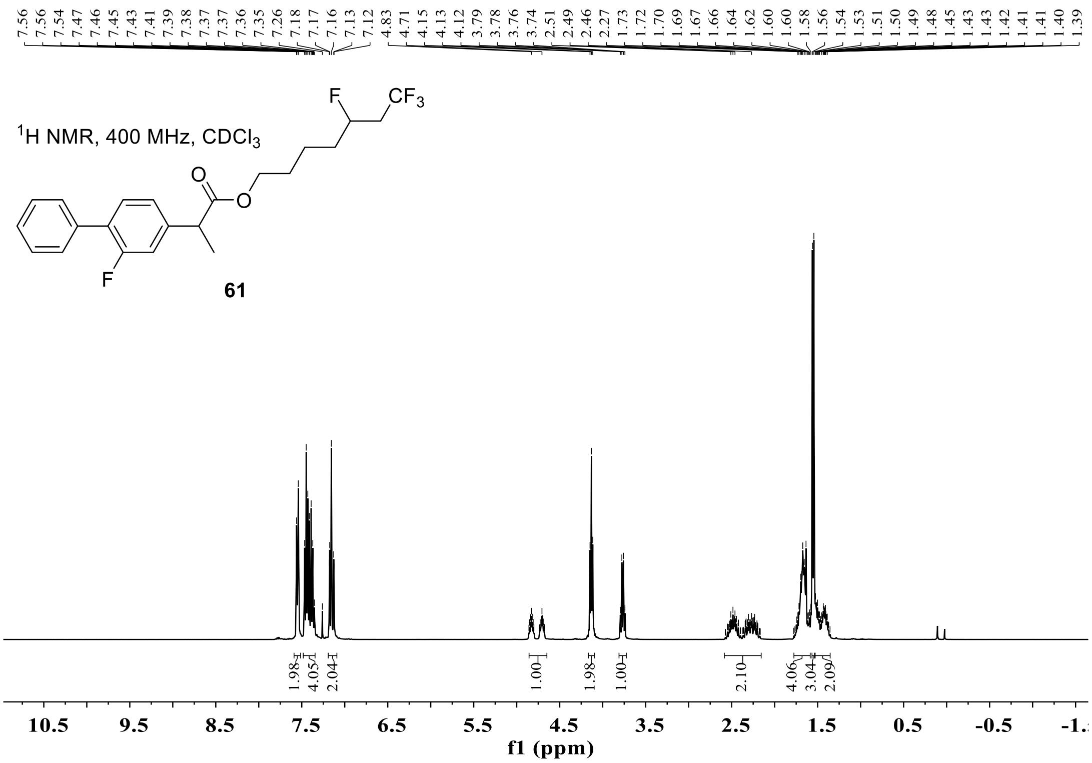

7.56 7.56 7.54 7.47 7.46 7.45 7.43 7.41 7.39 7.38 7.37 7.37 7.36 7.35 7.26 7.18 7.17 7.16 7.13 7.12 4.83 4.71 4.15 4.13 4.12 3.79 3.78 3.76 3.74 2.51 2.49 2.46 2.27 1.73 1.72 1.70 1.69 1.67 1.66 1.64 1.62 1.60 1.58 1.56 1.54 1.53 1.51 1.50 1.49 1.48 1.45 1.43 1.43 1.42 1.41 1.41 1.40 1.39
¹H NMR, 400 MHz, CDCl₃
F
CF₃
O
O
F
61
1.98
4.05
2.04
1.00
1.98
1.00
2.10
4.06
3.04
2.09
10.5 9.5 8.5 7.5 6.5 5.5 4.5 3.5 2.5 1.5 0.5 -0.5
f1 (ppm)

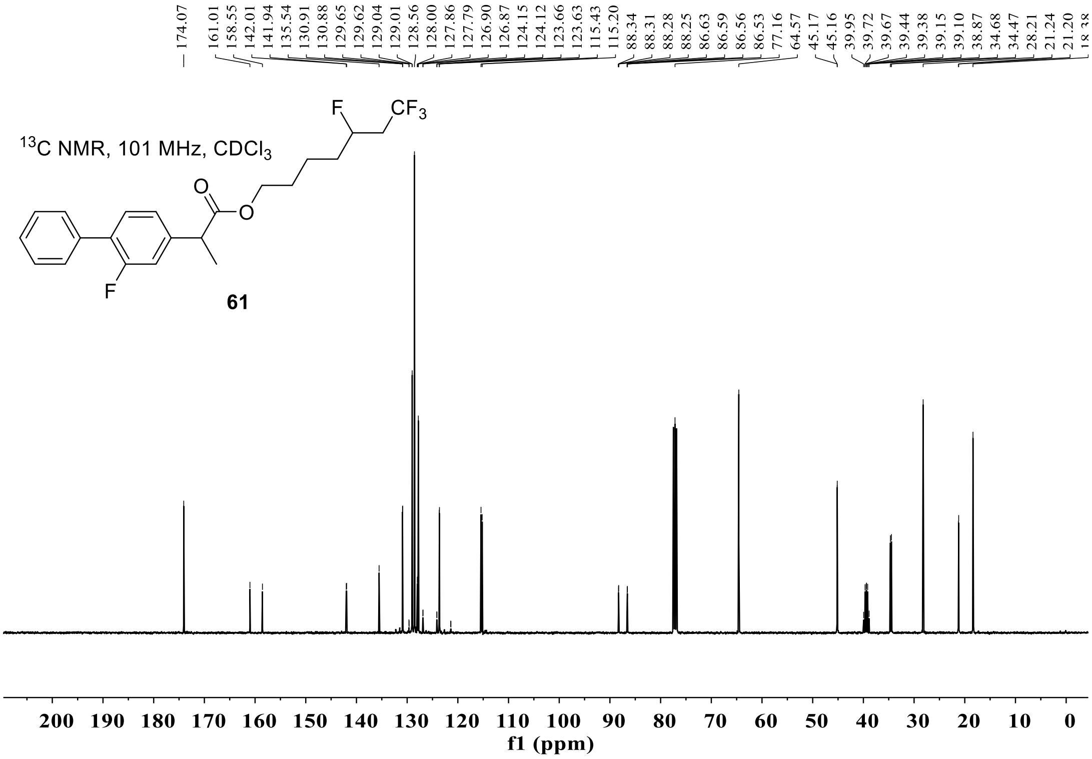

174.07 161.01 158.55 142.01 141.94 135.54 130.91 130.88 129.65 129.62 129.04 129.01 128.56 128.00 127.86 127.79 126.90 126.87 124.15 124.12 123.66 123.63 115.43 115.20 88.34 88.31 88.28 88.25 86.63 86.59 86.56 86.53 77.16 64.57 45.17 45.16 39.95 39.72 39.67 39.44 39.38 39.15 39.10 38.87 34.68 34.47 28.21 21.24 21.20
¹³C NMR, 101 MHz, CDCl₃
F
CF₃
O
O
F
61
200 190 180 170 160 150 140 130 120 110 100 90 80 70 60 50 40 30 20 10 0
f1 (ppm)

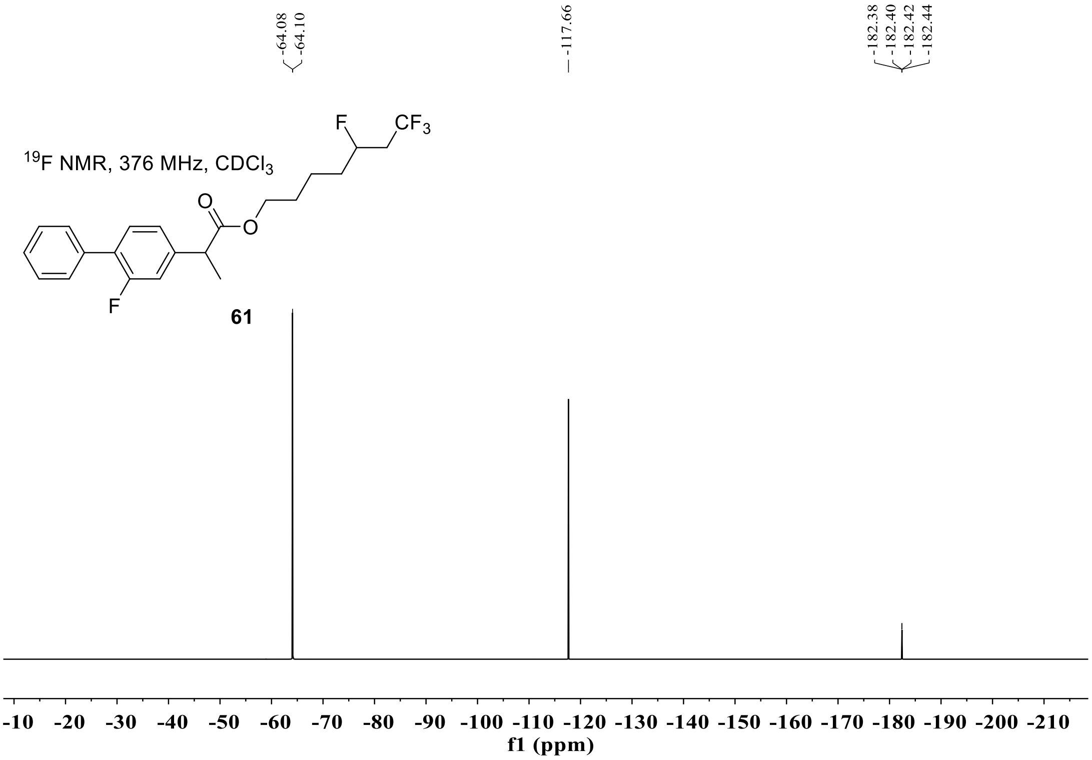
-64.08
-64.10
-117.66
-182.38
-182.40
-182.42
-182.44
19F NMR, 376 MHz, CDCl3
F
CF3
O
O
F
61
-10 -20 -30 -40 -50 -60 -70 -80 -90 -100 -110 -120 -130 -140 -150 -160 -170 -180 -190 -200 -210
f1 (ppm)

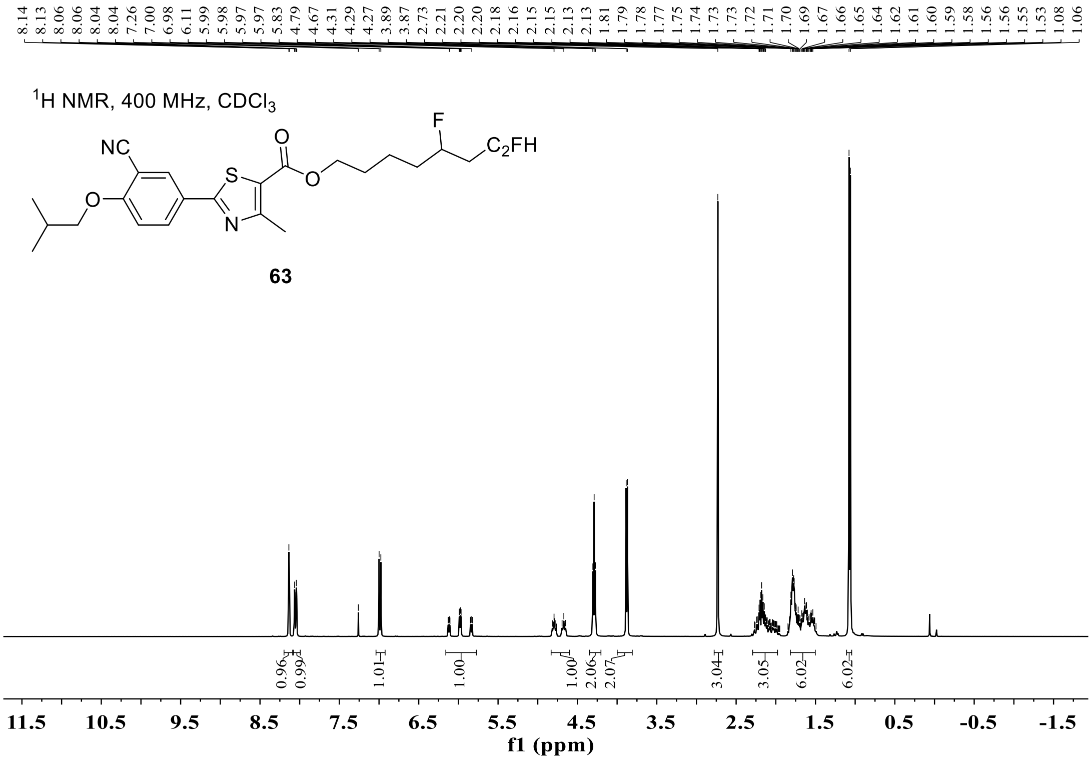
8.14 8.13 8.06 8.06 8.04 8.04 7.26 7.00 6.98 6.11 5.99 5.98 5.97 5.97 5.83 4.79 4.67 4.31 4.29 4.27 3.89 3.87 2.73 2.21 2.20 2.20 2.18 2.16 2.15 2.15 2.13 2.13 1.81 1.79 1.78 1.77 1.75 1.74 1.73 1.73 1.72 1.71 1.70 1.69 1.67 1.66 1.65 1.64 1.62 1.61 1.60 1.59 1.58 1.56 1.55 1.53 1.08 1.06
1H NMR, 400 MHz, CDCl3
NC
O
S
N
O
O
F
C2FH
63
0.96
0.99
1.01
1.00
1.00
2.06
2.07
3.04
3.05
6.02
6.02
11.5 10.5 9.5 8.5 7.5 6.5 5.5 4.5 3.5 2.5 1.5 0.5 -0.5 -1.5
f1 (ppm)

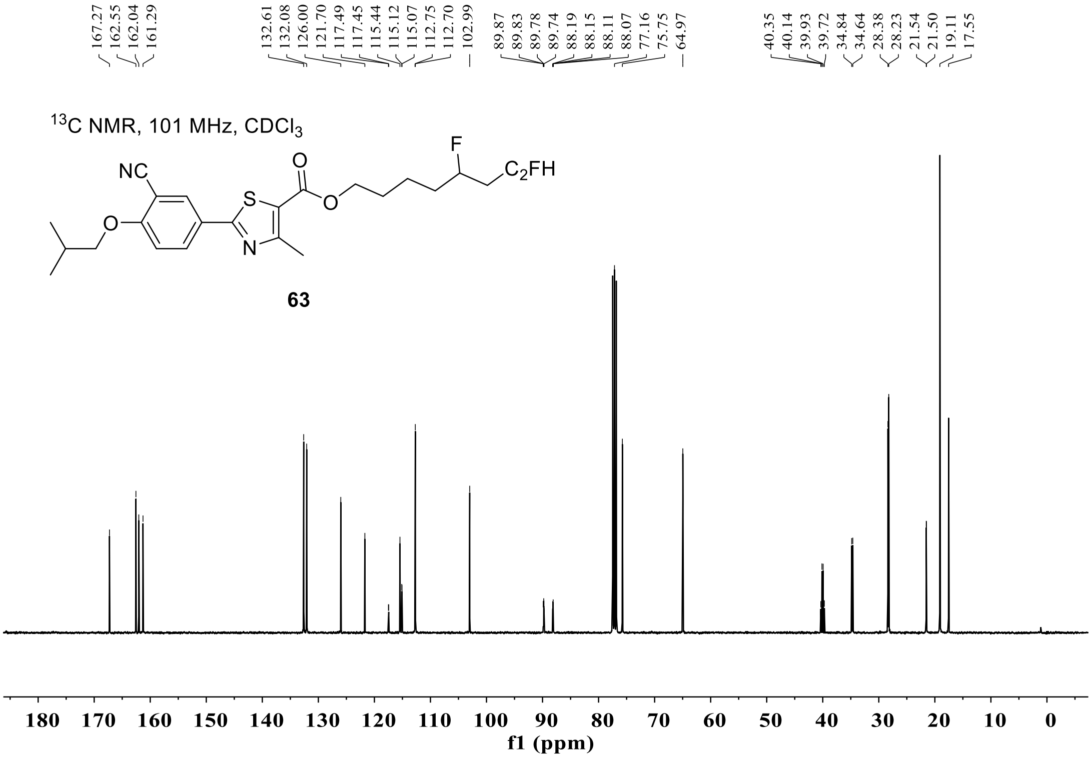
167.27
162.55
162.04
161.29
132.61
132.08
126.00
121.70
117.49
117.45
115.44
115.12
115.07
112.75
112.70
102.99
89.87
89.83
89.78
89.74
88.19
88.15
88.11
88.07
77.16
75.75
64.97
40.35
40.14
39.93
39.72
34.84
34.64
28.38
28.23
21.54
21.50
19.11
17.55
^{13}C NMR, 101 MHz, $CDCl_3$
NC
O
S
N
O
F
C_2FH
63
f1 (ppm)

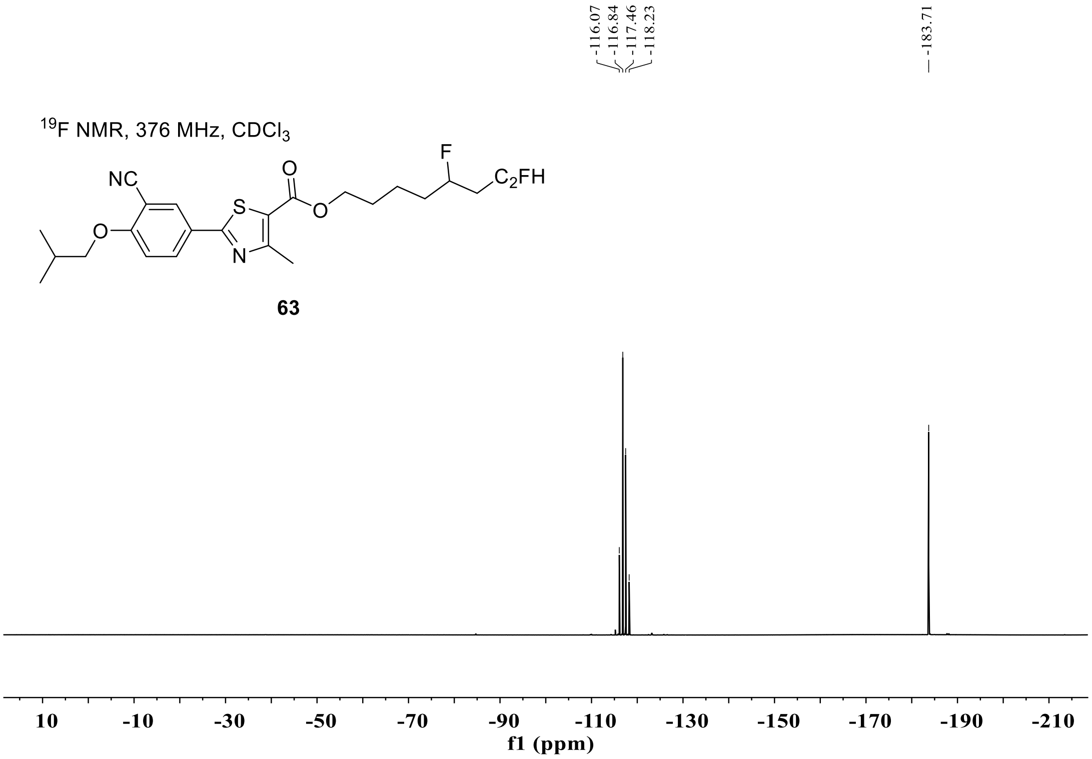
-116.07
-116.84
-117.46
-118.23
-183.71
^{19}F NMR, 376 MHz, $CDCl_3$
NC
O
S
N
O
F
C_2FH
63
f1 (ppm)

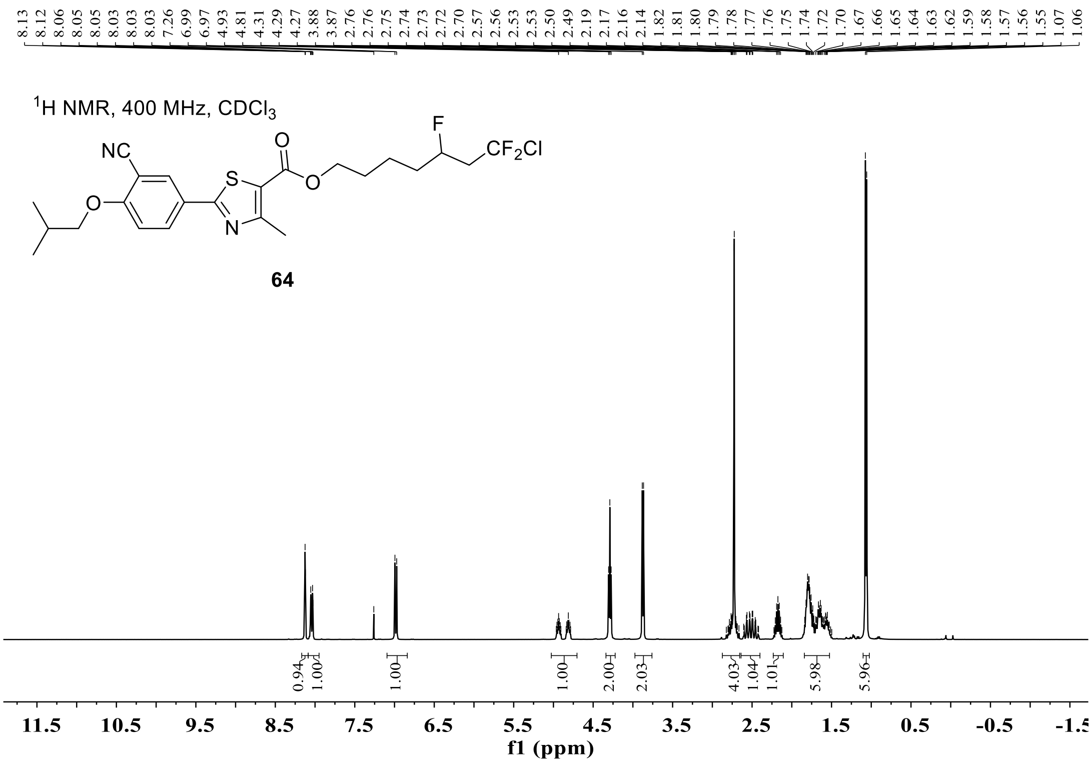

8.13 8.12 8.06 8.05 8.05 8.03 8.03 8.03 7.26 6.99 6.97 4.93 4.81 4.31 4.29 4.27 3.88 3.87 2.76 2.76 2.75 2.74 2.73 2.72 2.70 2.57 2.56 2.53 2.53 2.50 2.49 2.19 2.17 2.16 2.14 1.82 1.81 1.80 1.79 1.78 1.77 1.76 1.75 1.74 1.72 1.70 1.67 1.66 1.65 1.64 1.63 1.62 1.59 1.58 1.57 1.56 1.55 1.07 1.06
1H NMR, 400 MHz, CDCl3
NC
O
S
N
O
F
CF2Cl
64
0.94 1.00 1.00 1.00 2.00 2.03 4.03 1.04 1.01 5.98 5.96
11.5 10.5 9.5 8.5 7.5 6.5 5.5 4.5 3.5 2.5 1.5 0.5 -0.5
f1 (ppm)

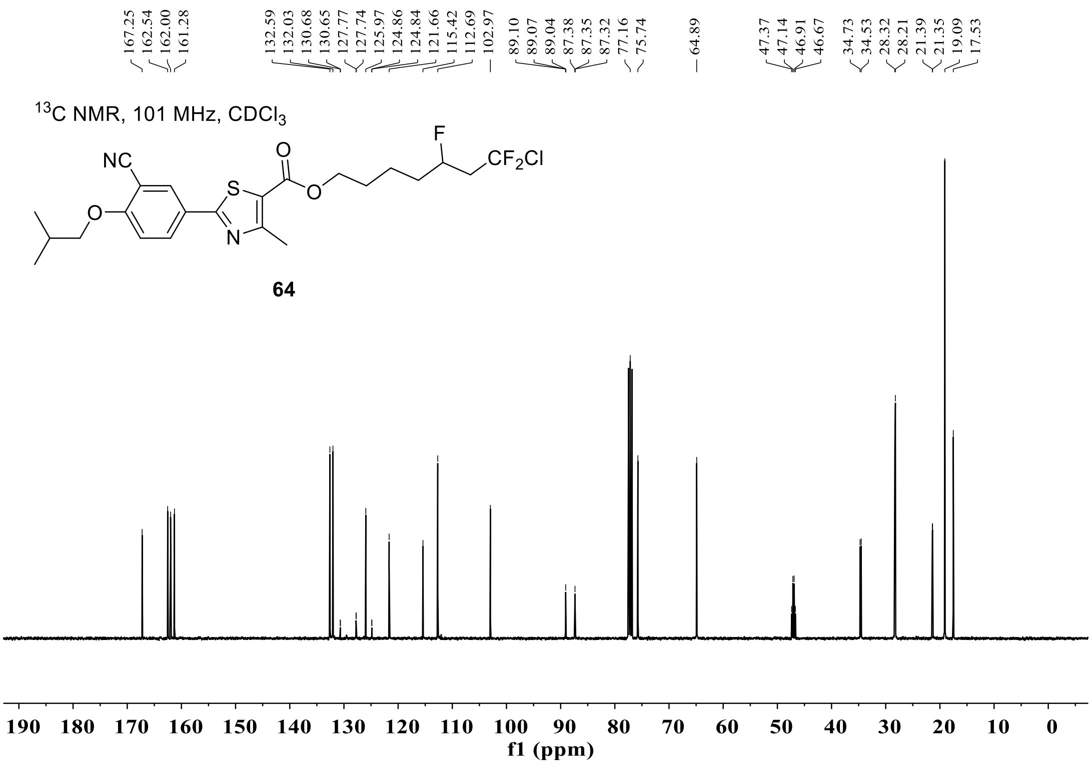

167.25 162.54 162.00 161.28 132.59 132.03 130.68 130.65 127.77 127.74 125.97 124.86 124.84 121.66 115.42 112.69 102.97 89.10 89.07 89.04 87.38 87.35 87.32 77.16 75.74 64.89 47.37 47.14 46.91 46.67 34.73 34.53 28.32 28.21 21.39 21.35 19.09 17.53
13C NMR, 101 MHz, CDCl3
NC
O
S
N
O
F
CF2Cl
64
190 180 170 160 150 140 130 120 110 100 90 80 70 60 50 40 30 20 10 0
f1 (ppm)

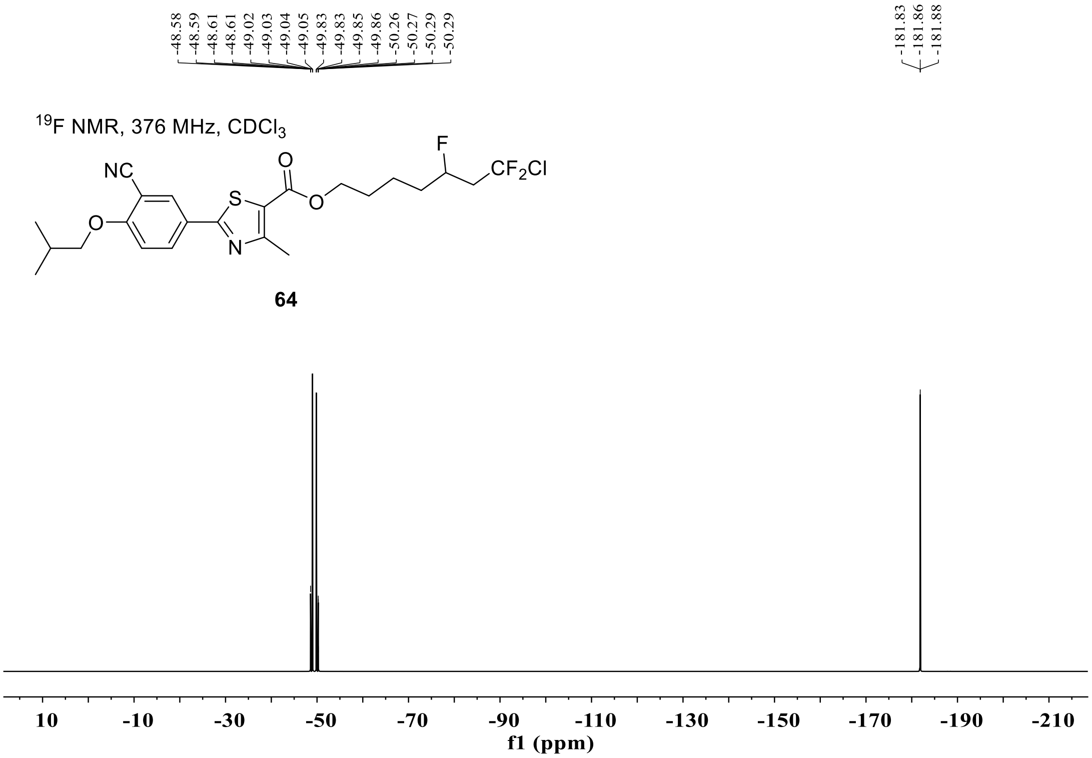

-48.58 -48.59 -48.61 -48.61 -49.02 -49.03 -49.04 -49.05 -49.83 -49.83 -49.85 -49.86 -50.26 -50.27 -50.29 -50.29
-181.83 -181.86 -181.88
19F NMR, 376 MHz, CDCl3
NC
O
F
CF2Cl
S
O
O
N
64
10 -10 -30 -50 -70 -90 -110 -130 -150 -170 -190 -210
f1 (ppm)

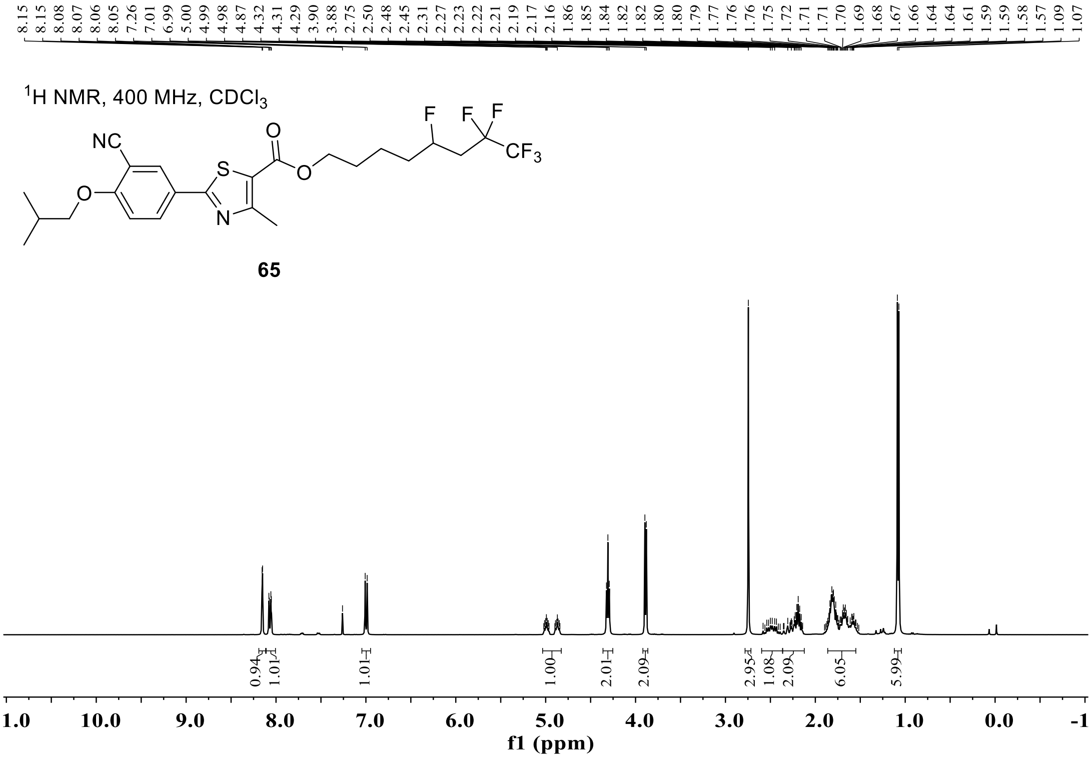

8.15 8.15 8.08 8.07 8.06 8.05 7.26 7.01 6.99 5.00 4.99 4.98 4.87 4.32 4.31 4.29 3.90 3.88 2.75 2.50 2.48 2.45 2.31 2.27 2.23 2.22 2.21 2.19 2.17 2.16 1.86 1.85 1.84 1.82 1.82 1.80 1.79 1.77 1.76 1.76 1.75 1.72 1.71 1.71 1.70 1.69 1.68 1.67 1.66 1.64 1.64 1.61 1.59 1.58 1.57 1.09 1.07
1H NMR, 400 MHz, CDCl3
NC
O
F F F
CF3
S
O
O
N
65
0.94 1.01 1.01 1.00 2.01 2.09 2.95 1.08 2.09 6.05 5.99
1.0 10.0 9.0 8.0 7.0 6.0 5.0 4.0 3.0 2.0 1.0 0.0 -1
f1 (ppm)

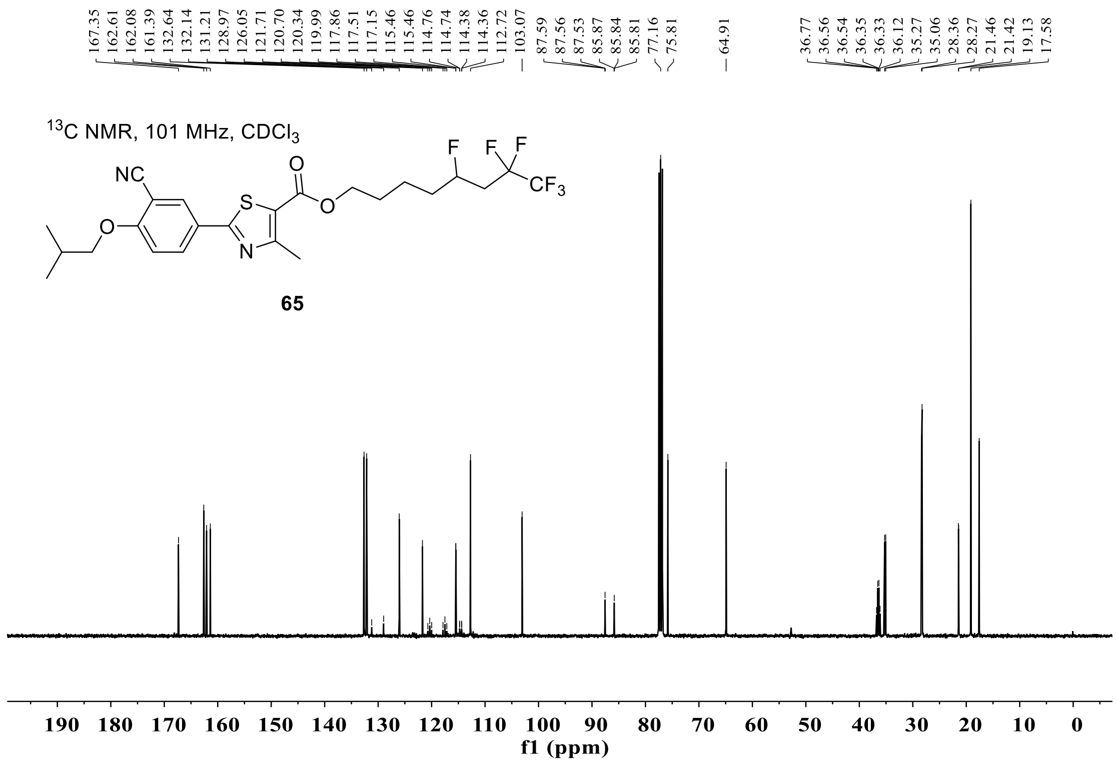
167.35
162.61
162.08
161.39
132.64
132.14
131.21
128.97
126.05
121.71
120.70
120.34
119.99
117.86
117.51
117.15
115.46
114.76
114.74
114.38
114.36
112.72
103.07
87.59
87.56
87.53
85.87
85.84
85.81
77.16
75.81
64.91
36.77
36.56
36.54
36.35
36.33
36.12
35.27
35.06
28.36
28.27
21.46
21.42
19.13
17.58
^{13}C NMR, 101 MHz, $CDCl_3$
NC
O
S
N
O
F
F
F
CF_3
65
190 180 170 160 150 140 130 120 110 100 90 80 70 60 50 40 30 20 10 0
f1 (ppm)

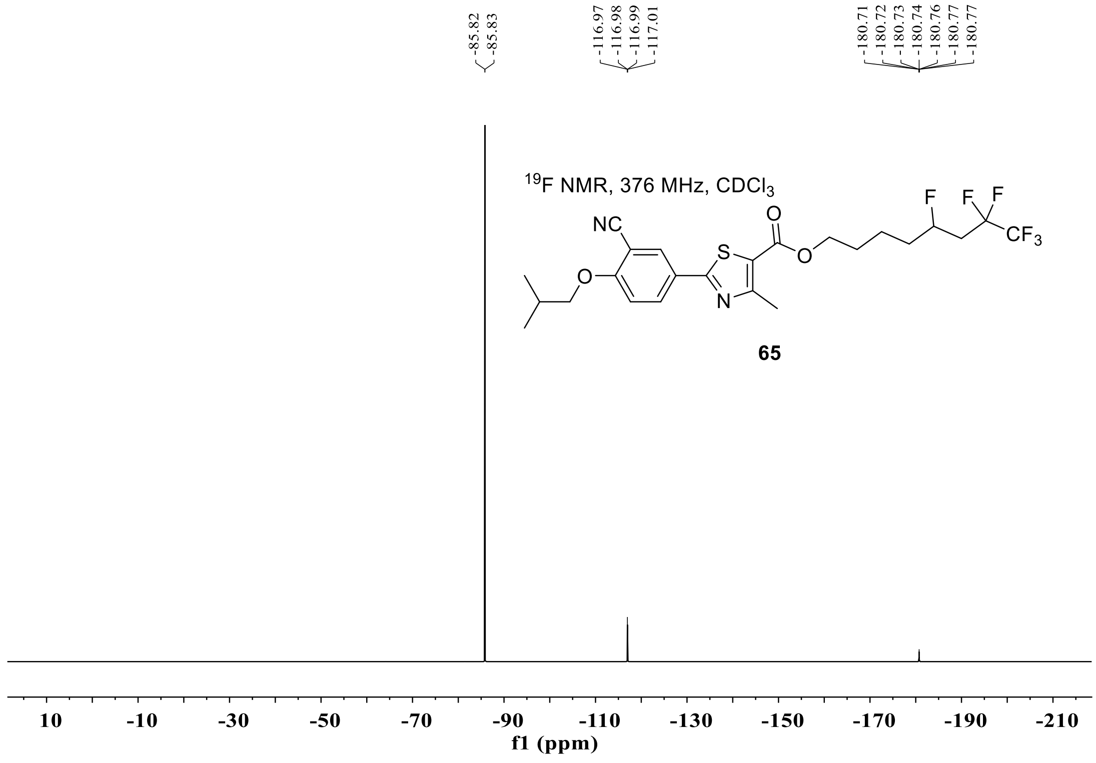
-85.82
-85.83
-116.97
-116.98
-116.99
-117.01
-180.71
-180.72
-180.73
-180.74
-180.76
-180.77
-180.77
^{19}F NMR, 376 MHz, $CDCl_3$
NC
O
S
N
O
F
F
F
CF_3
65
10 -10 -30 -50 -70 -90 -110 -130 -150 -170 -190 -210
f1 (ppm)

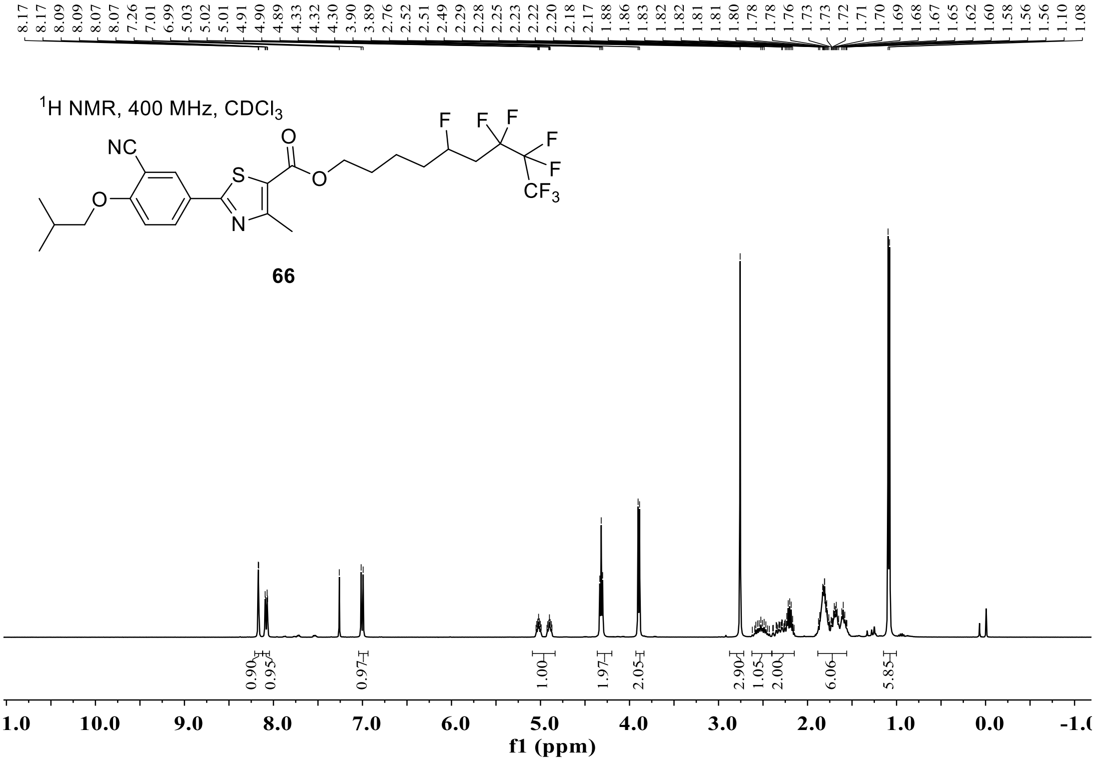

8.17 8.17 8.09 8.09 8.07 8.07 7.26 7.01 6.99 5.03 5.02 5.01 4.91 4.90 4.89 4.33 4.32 4.30 3.90 3.89 2.76 2.52 2.51 2.49 2.29 2.28 2.25 2.23 2.22 2.20 2.18 2.17 1.88 1.86 1.83 1.82 1.81 1.81 1.80 1.78 1.76 1.73 1.73 1.72 1.71 1.70 1.69 1.68 1.67 1.65 1.62 1.60 1.58 1.56 1.56 1.10 1.08
^{1}H NMR, 400 MHz, CDCl$_3$
NC
O
S
N
O
O
F
F
F
F
F
CF$_3$
66
0.90 0.95 0.97 1.00 1.97 2.05 2.90 1.05 2.00 6.06 5.85
1.0 10.0 9.0 8.0 7.0 6.0 5.0 4.0 3.0 2.0 1.0 0.0 -1.(
f1 (ppm)

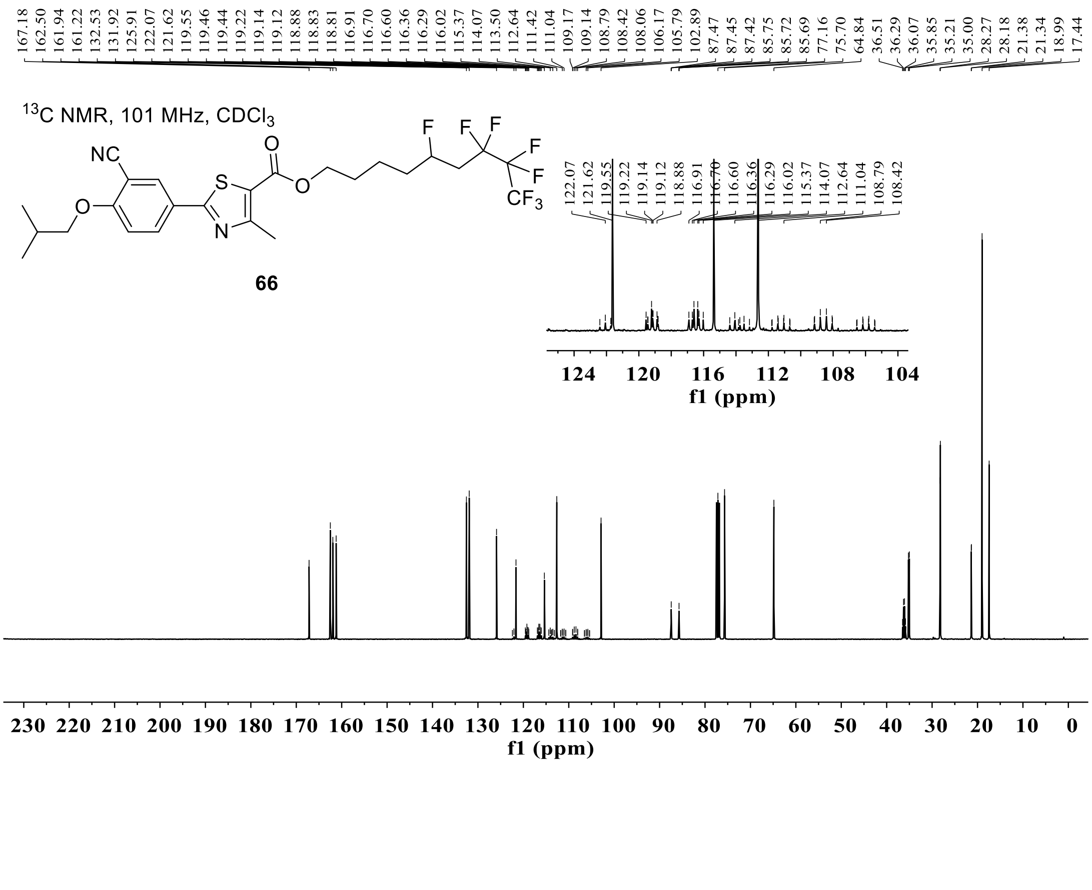

167.18 162.50 161.94 161.22 132.53 131.92 125.91 122.07 121.62 119.55 119.46 119.44 119.22 119.14 119.12 118.88 118.83 118.81 116.91 116.70 116.60 116.36 116.29 116.02 115.37 114.07 113.50 112.64 111.42 111.04 109.17 109.14 108.79 108.42 108.06 106.17 105.79 102.89 87.47 87.45 87.42 85.75 85.72 85.69 77.16 75.70 64.84 36.51 36.29 36.07 35.85 35.21 35.00 28.27 28.18 21.38 21.34 18.99 17.44
^{13}C NMR, 101 MHz, CDCl$_3$
NC
O
S
N
O
O
F
F
F
F
F
CF$_3$
66
122.07 121.62 119.55 119.22 119.14 119.12 118.88 116.91 116.70 116.60 116.36 116.29 116.02 115.37 114.07 112.64 111.04 108.79 108.42
124 120 116 112 108 104
f1 (ppm)
230 220 210 200 190 180 170 160 150 140 130 120 110 100 90 80 70 60 50 40 30 20 10 0
f1 (ppm)

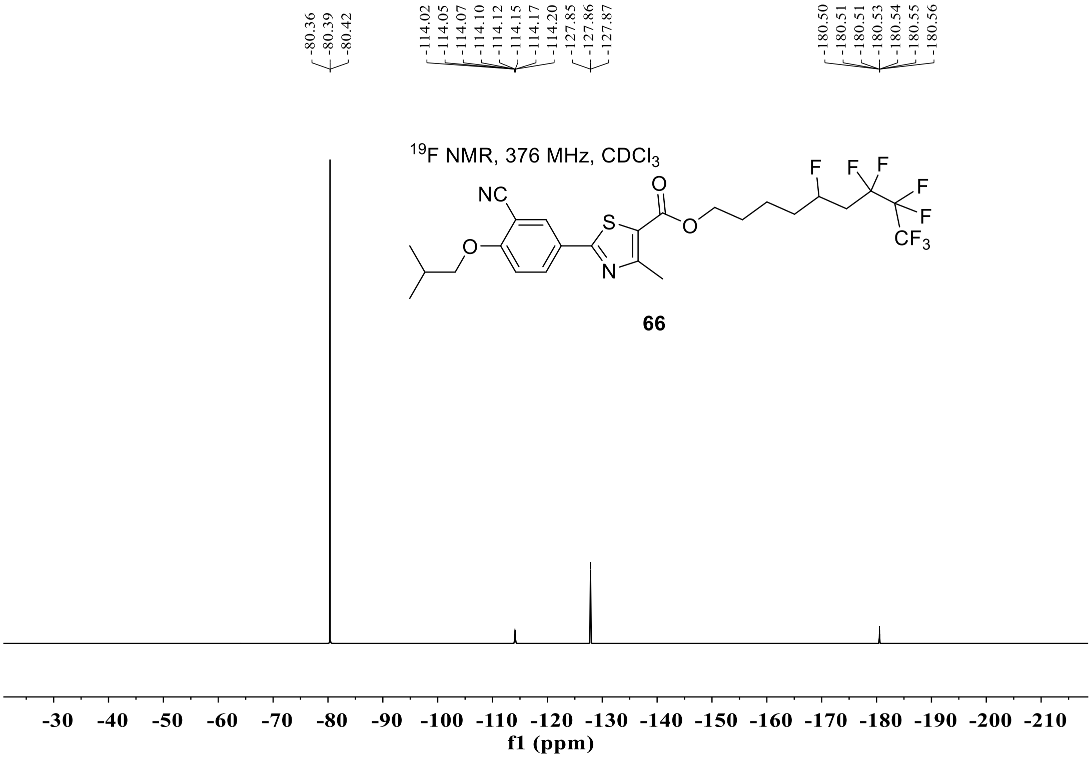

^{19}F NMR, 376 MHz, $CDCl_3$
NC
O
S
N
F
CF_3
66
f1 (ppm)

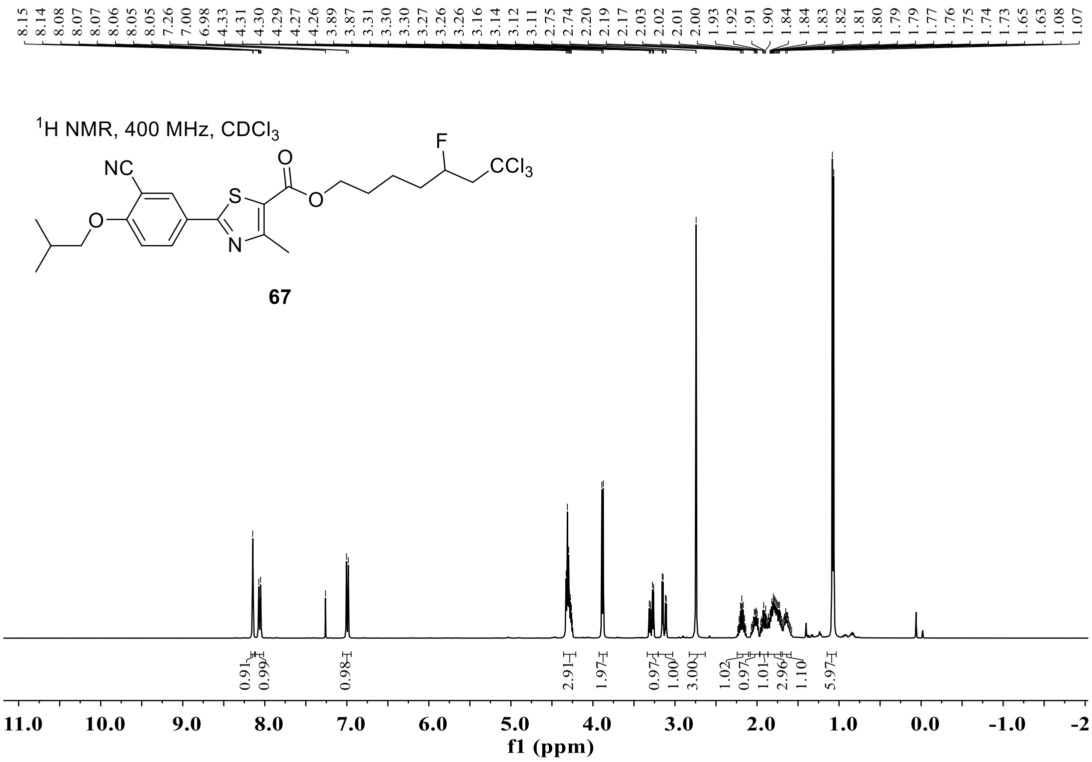

^{1}H NMR, 400 MHz, $CDCl_3$
NC
O
S
N
F
CCl_3
67
f1 (ppm)

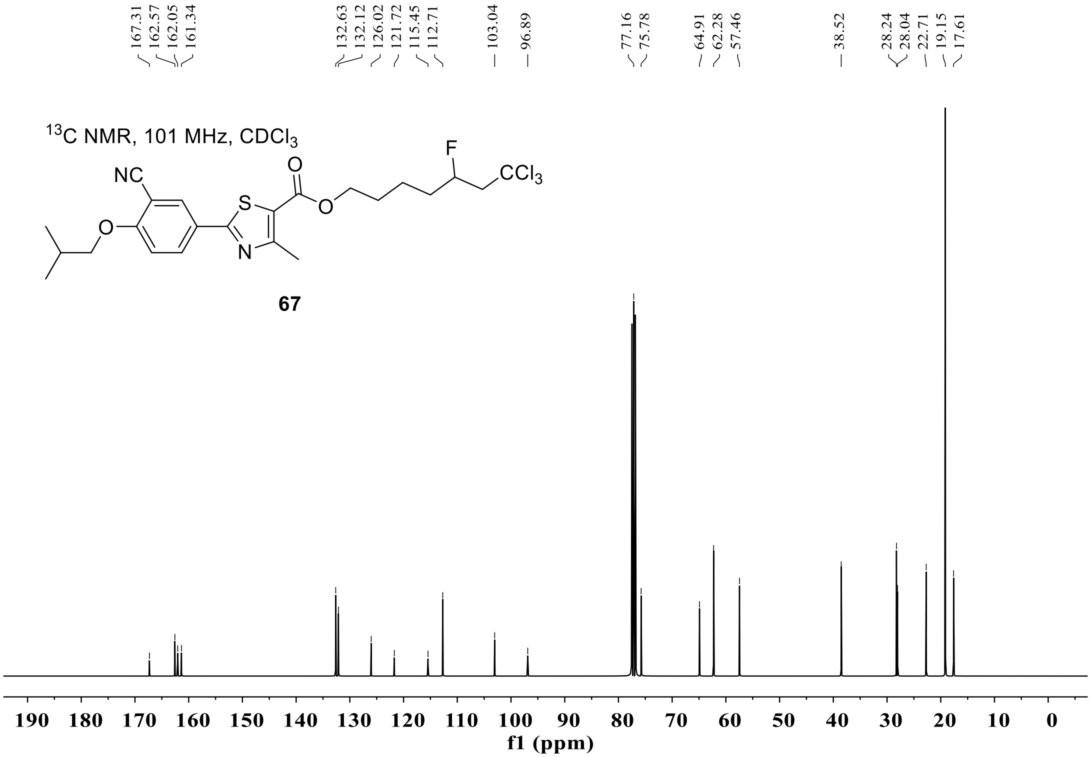

167.31
162.57
162.05
161.34
132.63
132.12
126.02
121.72
115.45
112.71
103.04
96.89
77.16
75.78
64.91
62.28
57.46
38.52
28.24
28.04
22.71
19.15
17.61
13C NMR, 101 MHz, CDCl3
NC
O
S
N
O
F
CCl3
67
190 180 170 160 150 140 130 120 110 100 90 80 70 60 50 40 30 20 10 0
f1 (ppm)

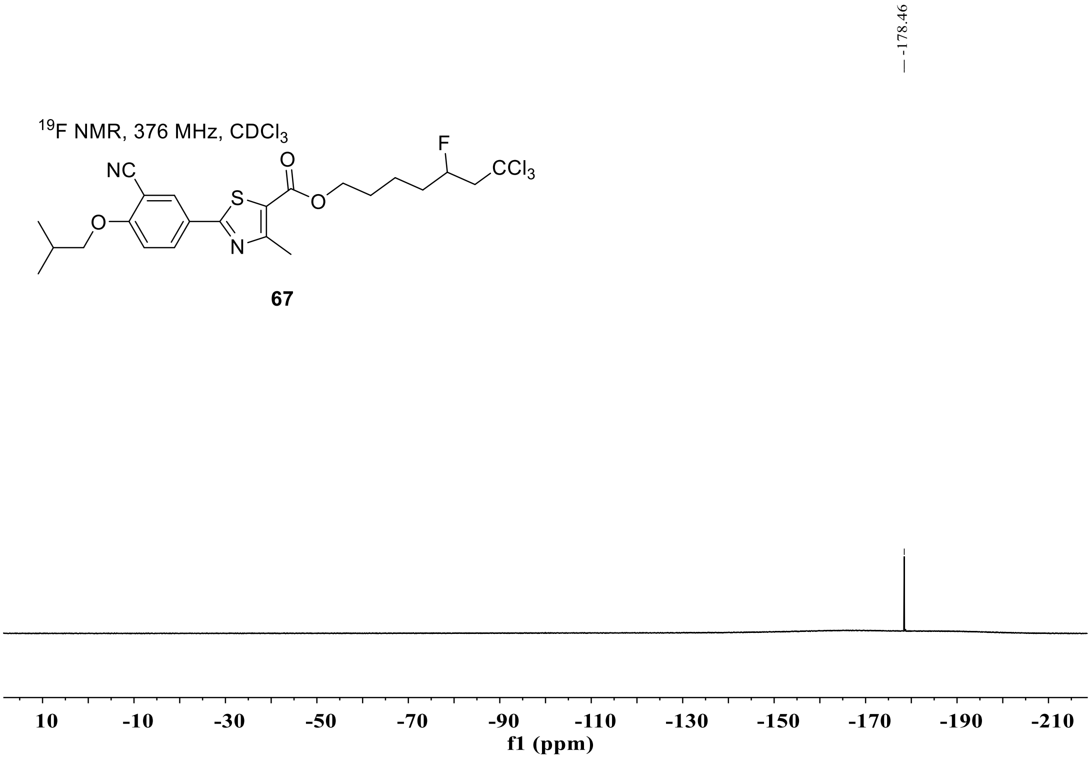

-178.46
19F NMR, 376 MHz, CDCl3
NC
O
S
N
O
F
CCl3
67
10 -10 -30 -50 -70 -90 -110 -130 -150 -170 -190 -210
f1 (ppm)

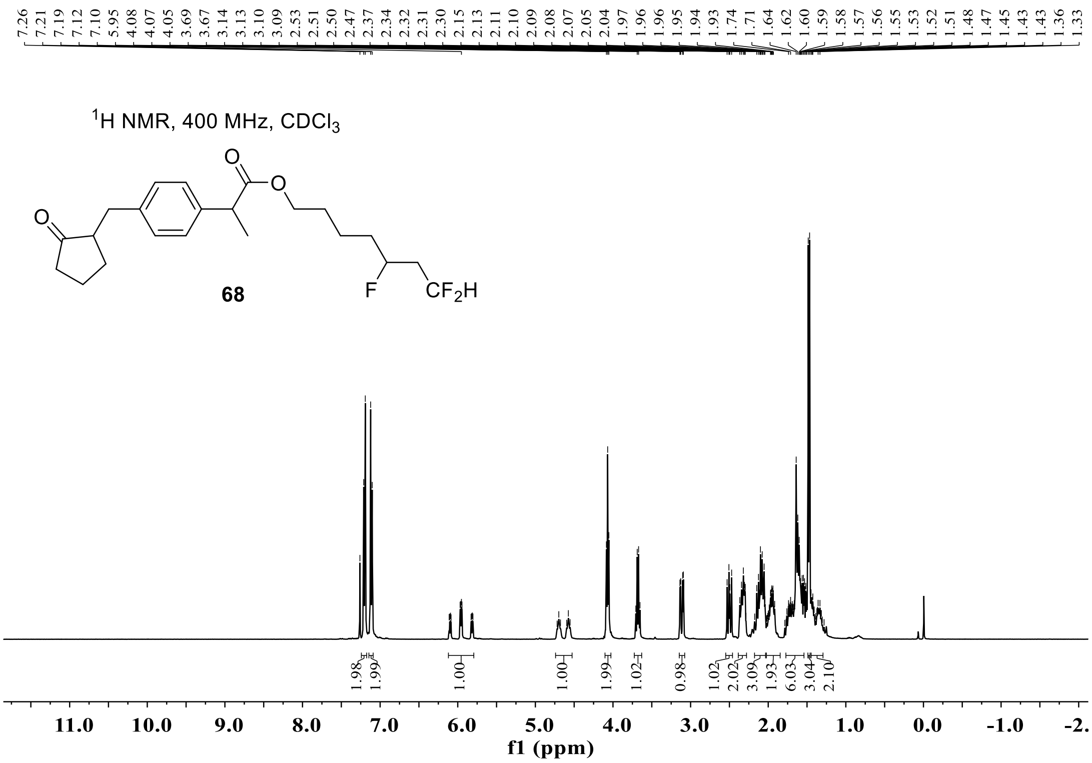
7.26 7.21 7.19 7.12 7.10 5.95 4.08 4.07 4.05 3.69 3.67 3.14 3.13 3.10 3.09 2.53 2.51 2.50 2.47 2.37 2.34 2.32 2.31 2.30 2.15 2.13 2.11 2.10 2.09 2.08 2.07 2.05 2.04 1.97 1.96 1.96 1.95 1.94 1.93 1.74 1.71 1.64 1.62 1.60 1.59 1.58 1.57 1.56 1.55 1.53 1.52 1.51 1.48 1.47 1.45 1.43 1.43 1.36 1.33
1H NMR, 400 MHz, CDCl3
O
O
O
68
F
CF2H
1.98 1.99 1.00 1.00 1.99 1.02 0.98 1.02 2.02 3.09 1.93 6.03 3.04 2.10
11.0 10.0 9.0 8.0 7.0 6.0 5.0 4.0 3.0 2.0 1.0 0.0 -1.0 -2.
f1 (ppm)

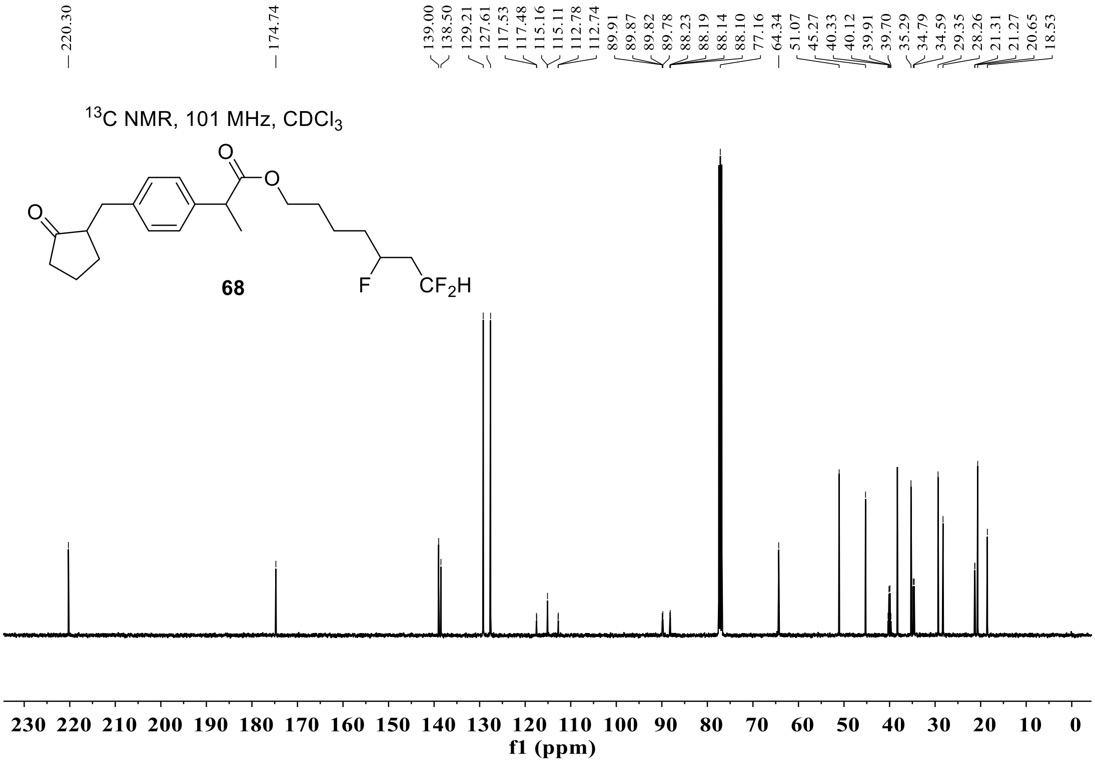
220.30 174.74 139.00 138.50 129.21 127.61 117.53 117.48 115.16 115.11 112.78 112.74 89.91 89.87 89.82 89.78 88.23 88.19 88.14 88.10 77.16 64.34 51.07 45.27 40.33 40.12 39.91 39.70 35.29 34.79 34.59 29.35 28.26 21.31 21.27 20.65 18.53
13C NMR, 101 MHz, CDCl3
O
O
O
68
F
CF2H
230 220 210 200 190 180 170 160 150 140 130 120 110 100 90 80 70 60 50 40 30 20 10 0
f1 (ppm)

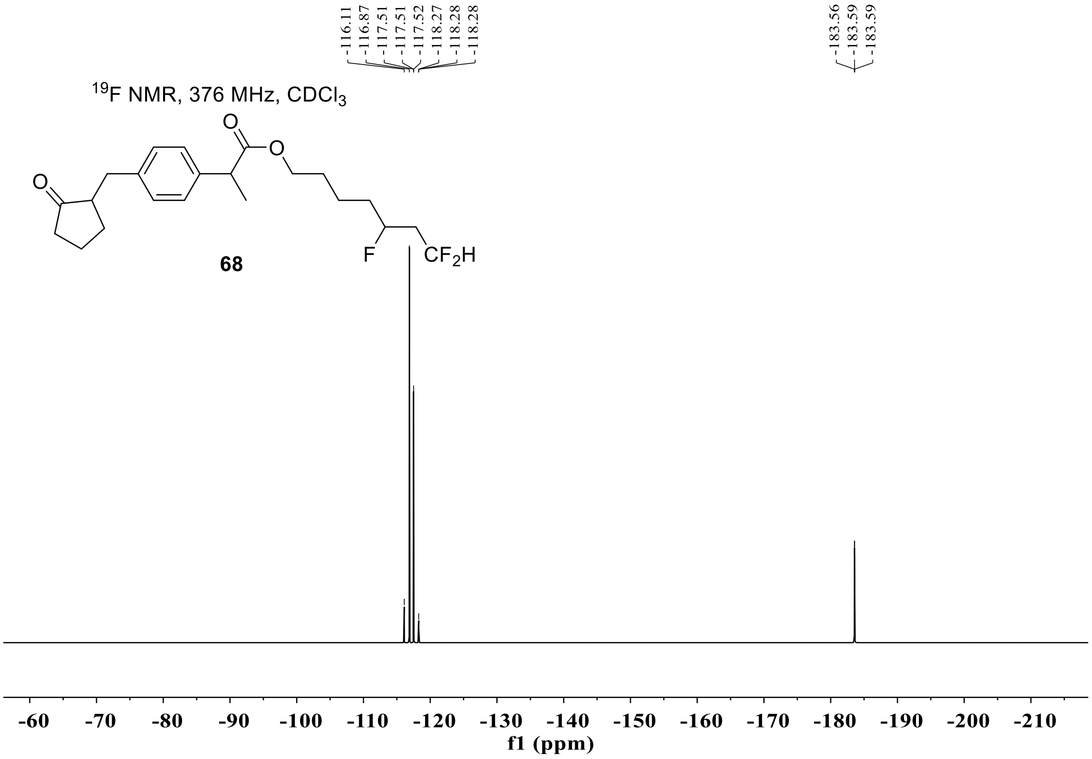
-116.11
-116.87
-117.51
-117.51
-117.52
-118.27
-118.28
-118.28
-183.56
-183.59
-183.59
19F NMR, 376 MHz, CDCl3
68
F
CF2H
-60 -70 -80 -90 -100 -110 -120 -130 -140 -150 -160 -170 -180 -190 -200 -210
f1 (ppm)

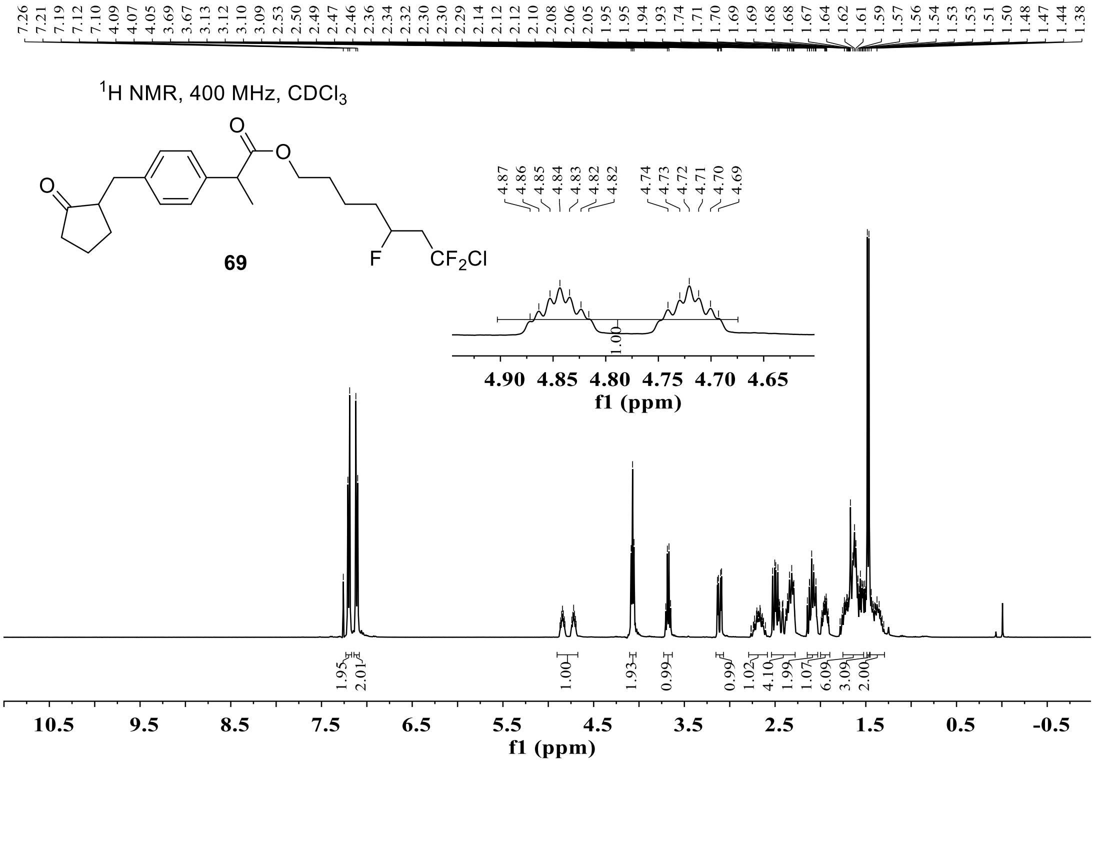
7.26 7.21 7.19 7.12 7.10 4.09 4.07 4.05 3.69 3.67 3.13 3.12 3.10 3.09 2.53 2.50 2.49 2.47 2.46 2.36 2.34 2.32 2.30 2.30 2.29 2.14 2.12 2.12 2.10 2.08 2.06 2.05 1.95 1.95 1.94 1.93 1.74 1.71 1.70 1.69 1.69 1.68 1.68 1.67 1.64 1.62 1.61 1.59 1.57 1.56 1.54 1.53 1.53 1.51 1.50 1.48 1.47 1.44 1.38
1H NMR, 400 MHz, CDCl3
69
F
CF2Cl
4.87 4.86 4.85 4.84 4.83 4.82 4.82 4.74 4.73 4.72 4.71 4.70 4.69
1.00
4.90 4.85 4.80 4.75 4.70 4.65
f1 (ppm)
1.95 2.01 1.00 1.93 0.99 0.99 1.02 4.10 1.99 1.07 6.09 3.09 2.00
10.5 9.5 8.5 7.5 6.5 5.5 4.5 3.5 2.5 1.5 0.5 -0.5
f1 (ppm)

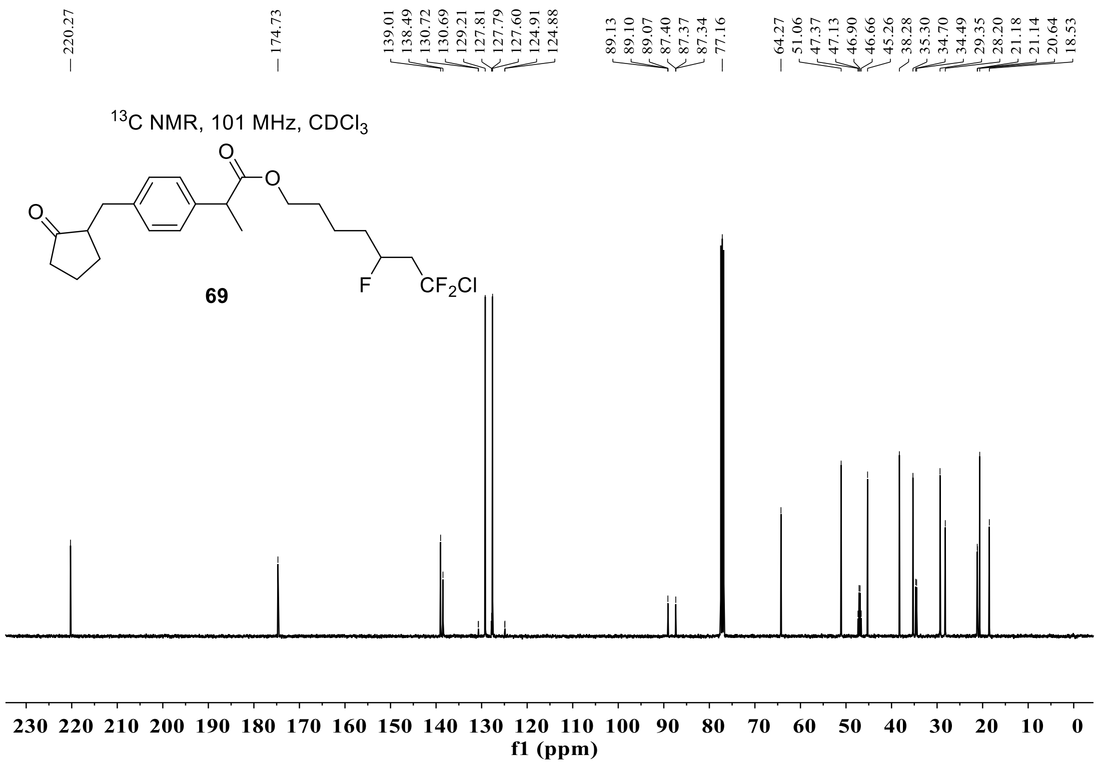
220.27
174.73
139.01
138.49
130.72
130.69
129.21
127.81
127.79
127.60
124.91
124.88
89.13
89.10
89.07
87.40
87.37
87.34
77.16
64.27
51.06
47.37
47.13
46.90
46.66
45.26
38.28
35.30
34.70
34.49
29.35
28.20
21.18
21.14
20.64
18.53
13C NMR, 101 MHz, CDCl3
O
O
O
F
CF2Cl
69
230 220 210 200 190 180 170 160 150 140 130 120 110 100 90 80 70 60 50 40 30 20 10 0
f1 (ppm)

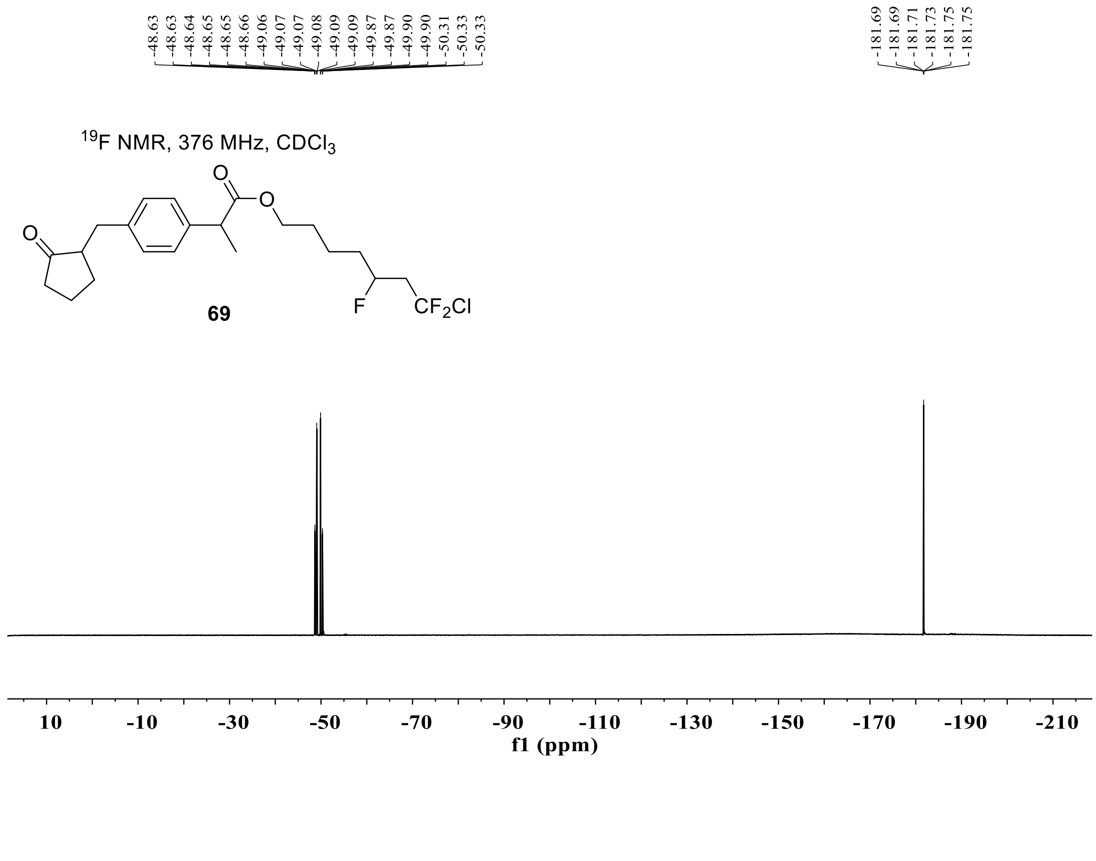
-48.63
-48.63
-48.64
-48.65
-48.65
-48.66
-49.06
-49.07
-49.07
-49.08
-49.09
-49.09
-49.87
-49.87
-49.90
-49.90
-50.31
-50.33
-50.33
-181.69
-181.69
-181.71
-181.73
-181.75
-181.75
19F NMR, 376 MHz, CDCl3
O
O
O
F
CF2Cl
69
10 -10 -30 -50 -70 -90 -110 -130 -150 -170 -190 -210
f1 (ppm)

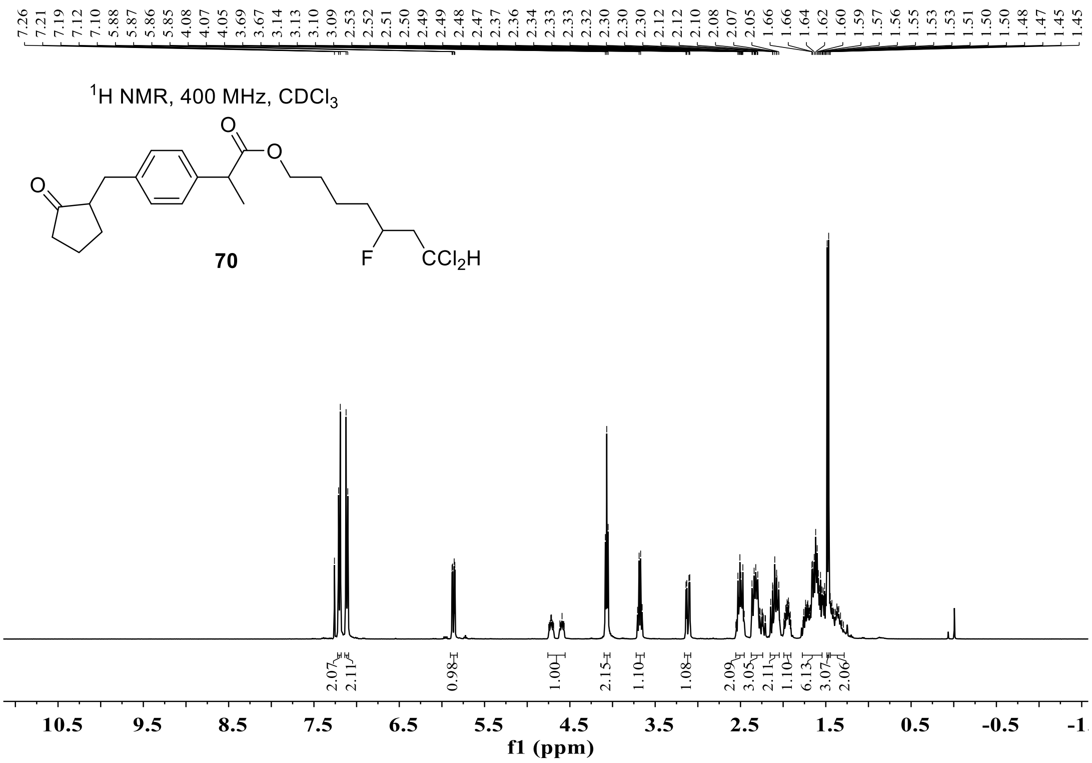
^{1}H NMR, 400 MHz, CDCl$_3$
70
F
CCl$_2$H
f1 (ppm)

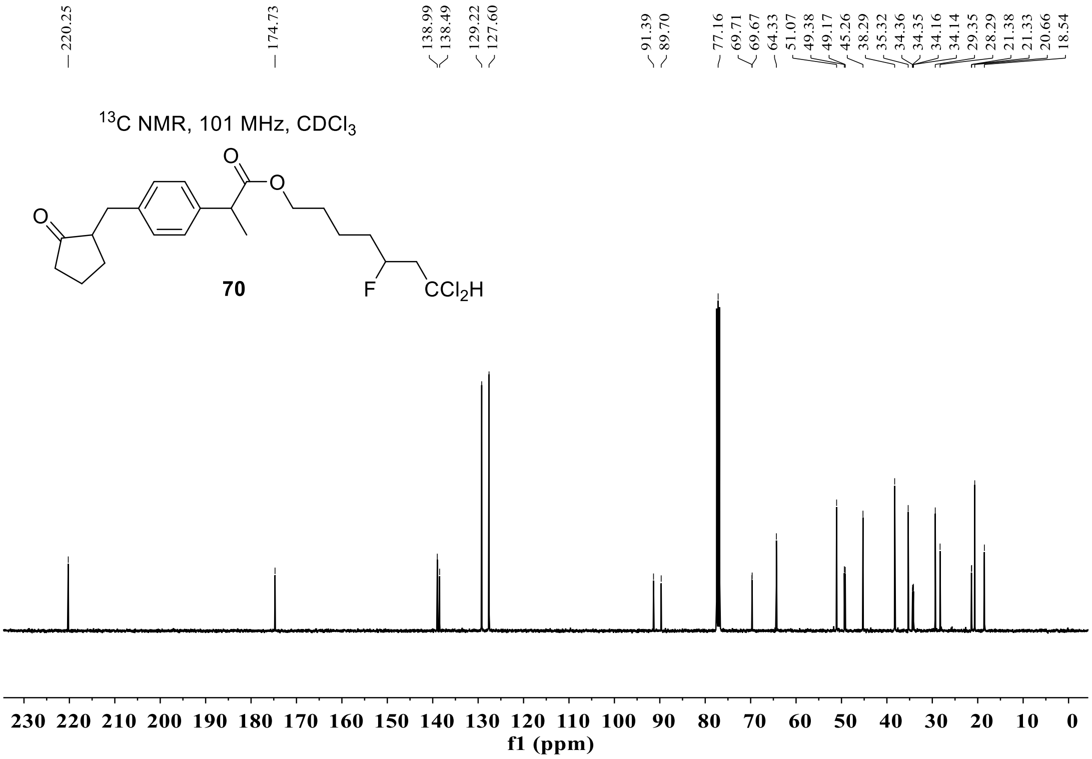
^{13}C NMR, 101 MHz, CDCl$_3$
70
F
CCl$_2$H
f1 (ppm)

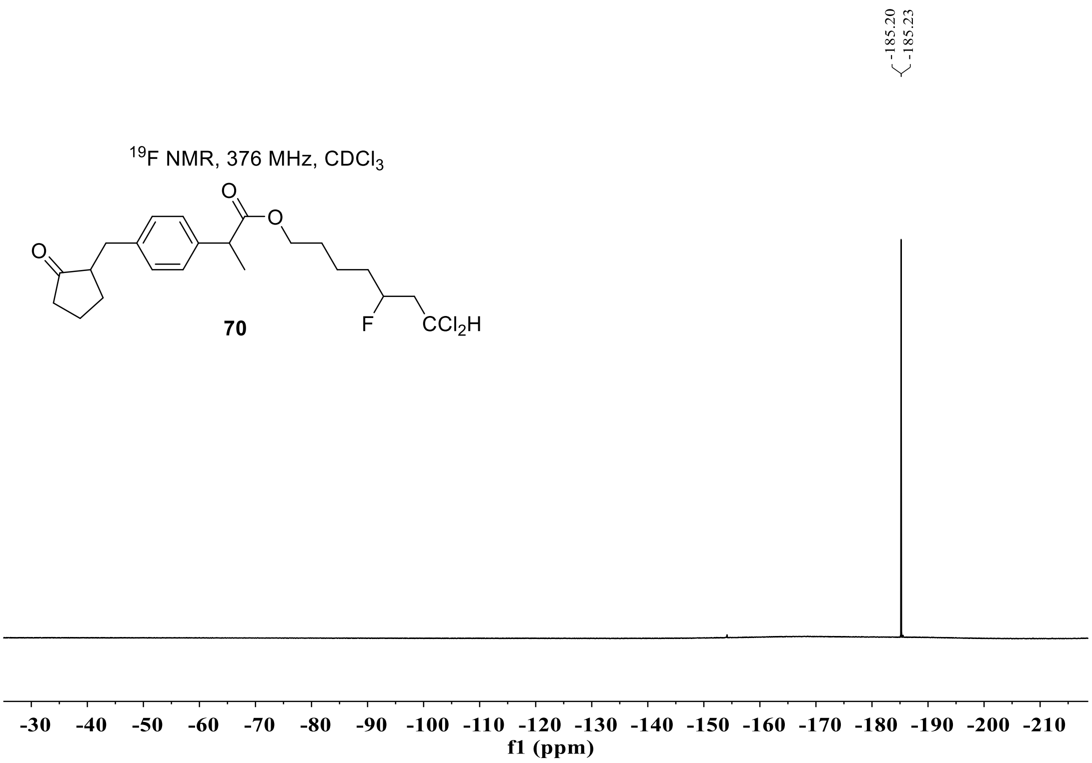
-185.20
-185.23
^{19}F NMR, 376 MHz, $CDCl_3$
70
F
CCl_2H
O
-30 -40 -50 -60 -70 -80 -90 -100 -110 -120 -130 -140 -150 -160 -170 -180 -190 -200 -210
f1 (ppm)

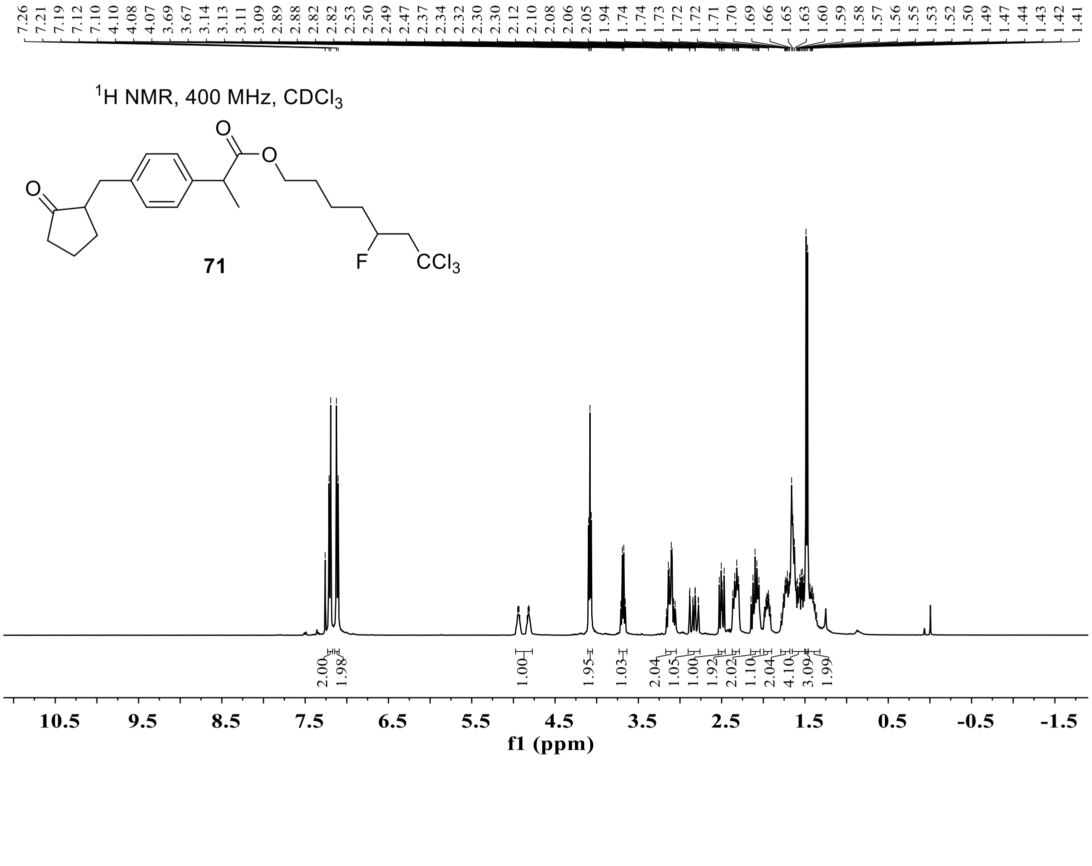
7.26 7.21 7.19 7.12 7.10 4.10 4.08 4.07 3.69 3.67 3.14 3.13 3.11 3.09 2.89 2.88 2.82 2.82 2.53 2.50 2.49 2.47 2.37 2.34 2.32 2.30 2.30 2.12 2.10 2.08 2.06 2.05 1.94 1.74 1.74 1.73 1.72 1.72 1.71 1.70 1.69 1.66 1.65 1.63 1.60 1.59 1.58 1.57 1.56 1.55 1.53 1.52 1.50 1.49 1.47 1.44 1.43 1.42 1.41
^{1}H NMR, 400 MHz, $CDCl_3$
71
F
CCl_3
O
2.00 1.98 1.00 1.95 1.03 2.04 1.05 1.00 1.92 2.02 1.10 2.04 4.10 3.09 1.99
10.5 9.5 8.5 7.5 6.5 5.5 4.5 3.5 2.5 1.5 0.5 -0.5 -1.5
f1 (ppm)

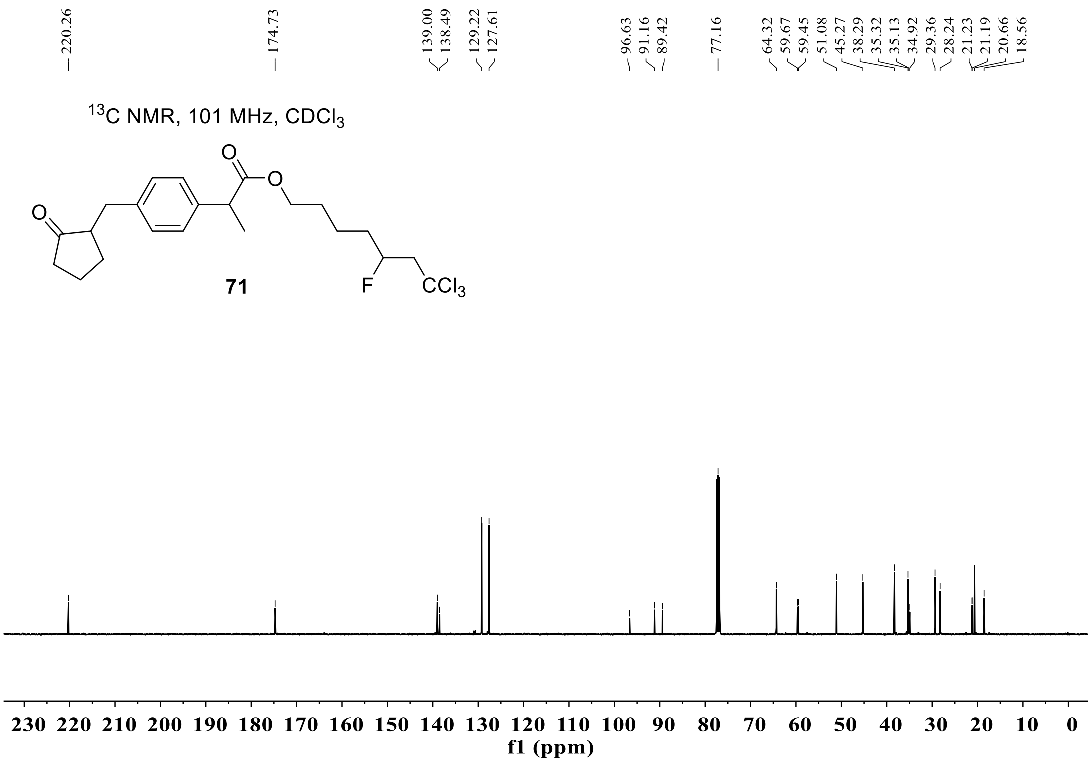
220.26
174.73
139.00
138.49
129.22
127.61
96.63
91.16
89.42
77.16
64.32
59.67
59.45
51.08
45.27
38.29
35.32
35.13
34.92
29.36
28.24
21.23
21.19
20.66
18.56
^{13}C NMR, 101 MHz, CDCl$_3$
O
O
O
O
F
CCl$_3$
71
230 220 210 200 190 180 170 160 150 140 130 120 110 100 90 80 70 60 50 40 30 20 10 0
f1 (ppm)

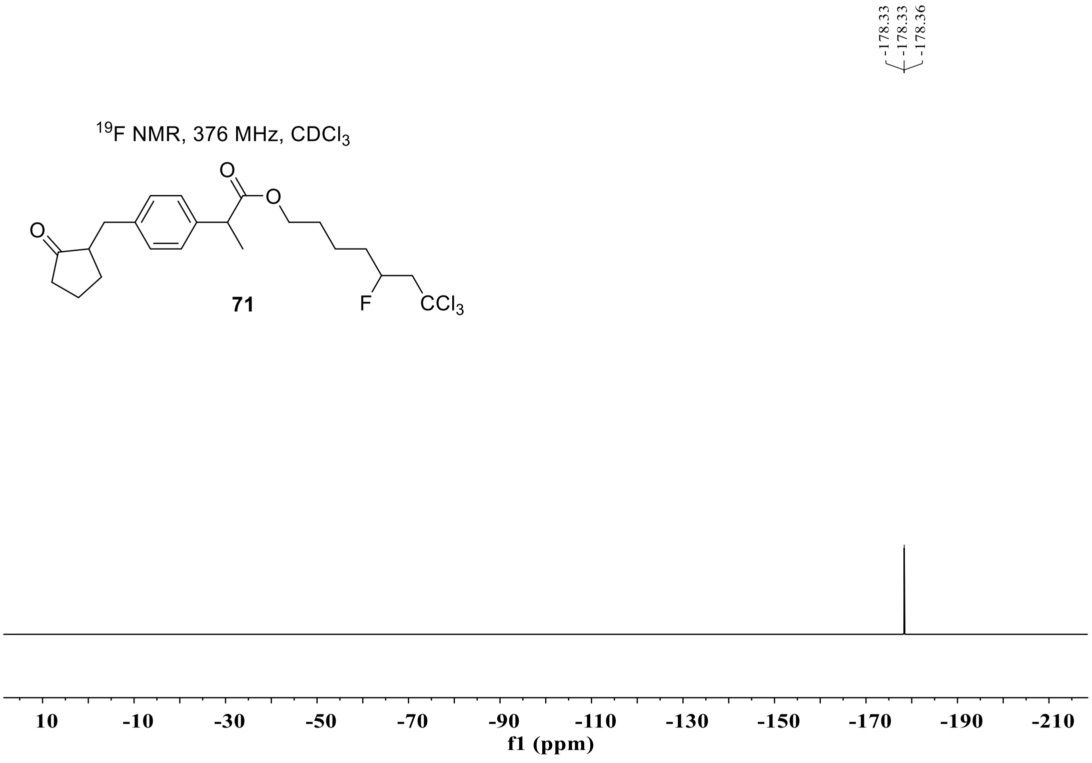
-178.33
-178.33
-178.36
^{19}F NMR, 376 MHz, CDCl$_3$
O
O
O
O
F
CCl$_3$
71
10 -10 -30 -50 -70 -90 -110 -130 -150 -170 -190 -210
f1 (ppm)

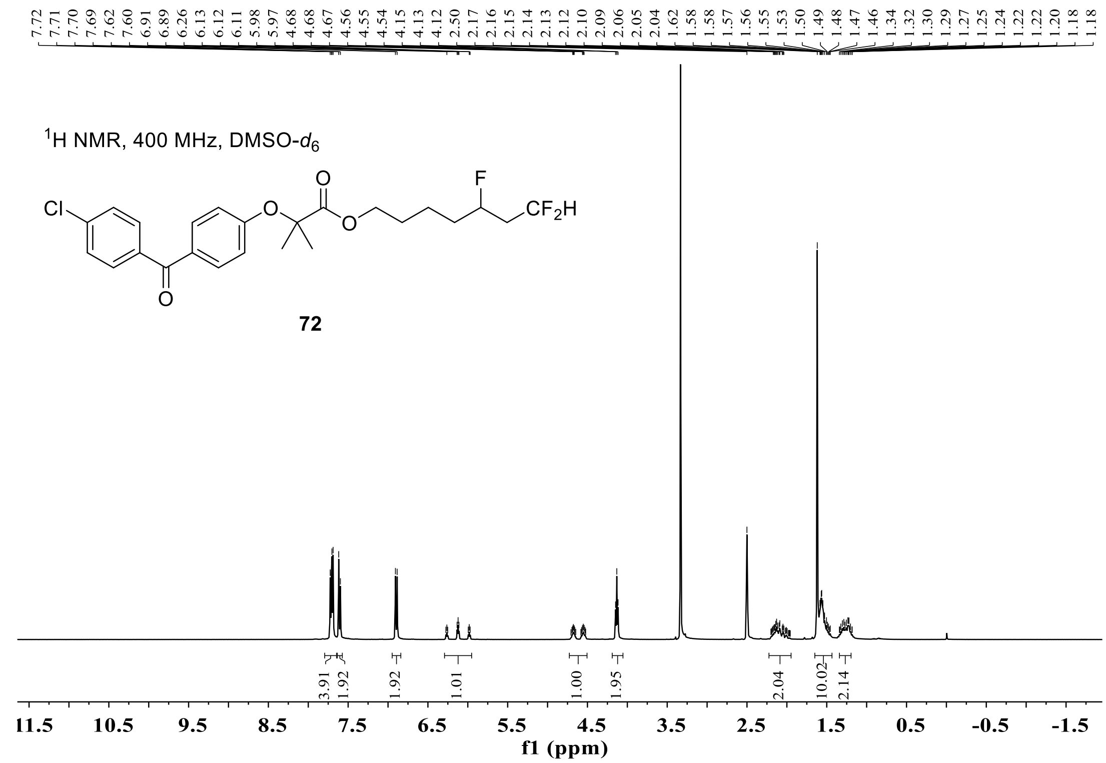
7.72 7.71 7.70 7.69 7.62 7.60 6.91 6.89 6.26 6.13 6.12 6.11 5.98 5.97 4.68 4.67 4.56 4.55 4.54 4.15 4.13 4.12 2.50 2.17 2.16 2.15 2.14 2.13 2.12 2.10 2.09 2.06 2.05 2.04 1.62 1.58 1.57 1.56 1.55 1.53 1.50 1.49 1.48 1.47 1.46 1.34 1.32 1.30 1.29 1.27 1.25 1.24 1.22 1.20 1.18 1.18
1H NMR, 400 MHz, DMSO-d6
Cl
O
O
O
O
F
CF2H
72
3.91 1.92 1.92 1.01 1.00 1.95 2.04 10.02 2.14
11.5 10.5 9.5 8.5 7.5 6.5 5.5 4.5 3.5 2.5 1.5 0.5 -0.5 -1.5
f1 (ppm)

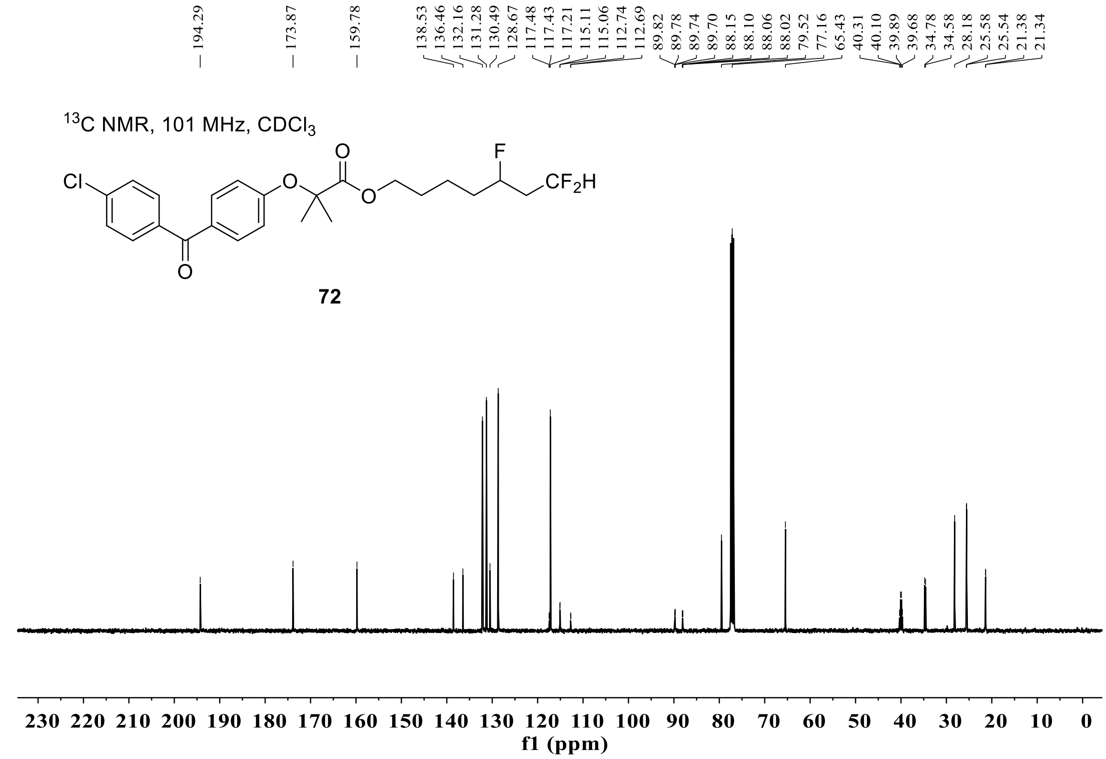
194.29 173.87 159.78 138.53 136.46 132.16 131.28 130.49 128.67 117.48 117.43 117.21 115.11 115.06 112.74 112.69 89.82 89.78 89.74 89.70 88.15 88.10 88.06 88.02 79.52 77.16 65.43 40.31 40.10 39.89 39.68 34.78 34.58 28.18 25.58 25.54 21.38 21.34
13C NMR, 101 MHz, CDCl3
Cl
O
O
O
O
F
CF2H
72
230 220 210 200 190 180 170 160 150 140 130 120 110 100 90 80 70 60 50 40 30 20 10 0
f1 (ppm)

^{19}F NMR, 376 MHz, $CDCl_3$

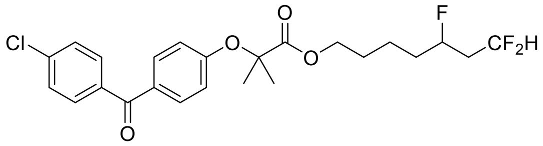

72

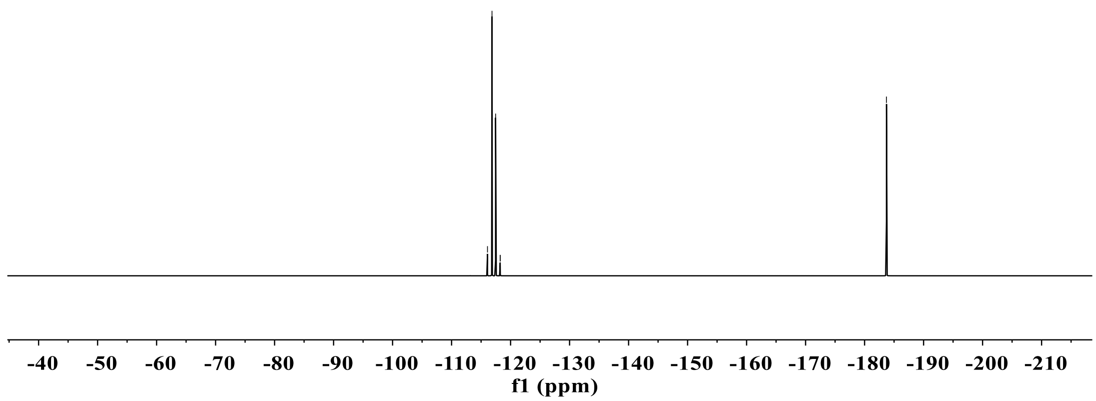

^{1}H NMR, 400 MHz, $CDCl_3$

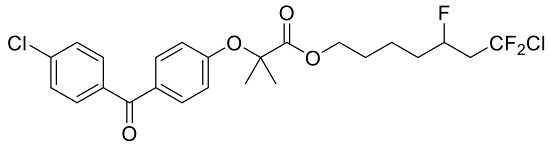

73

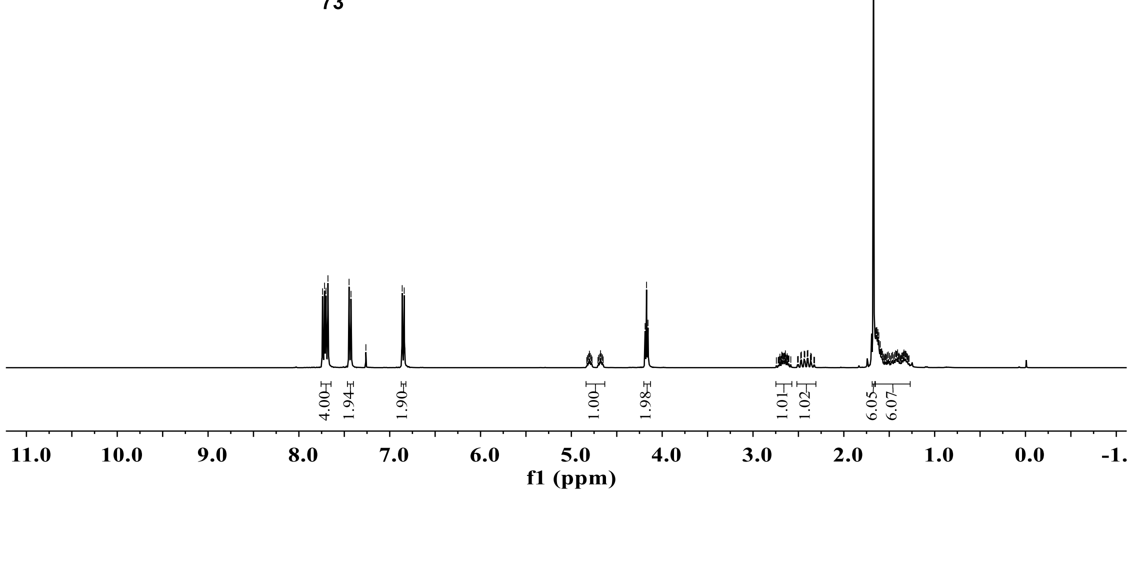

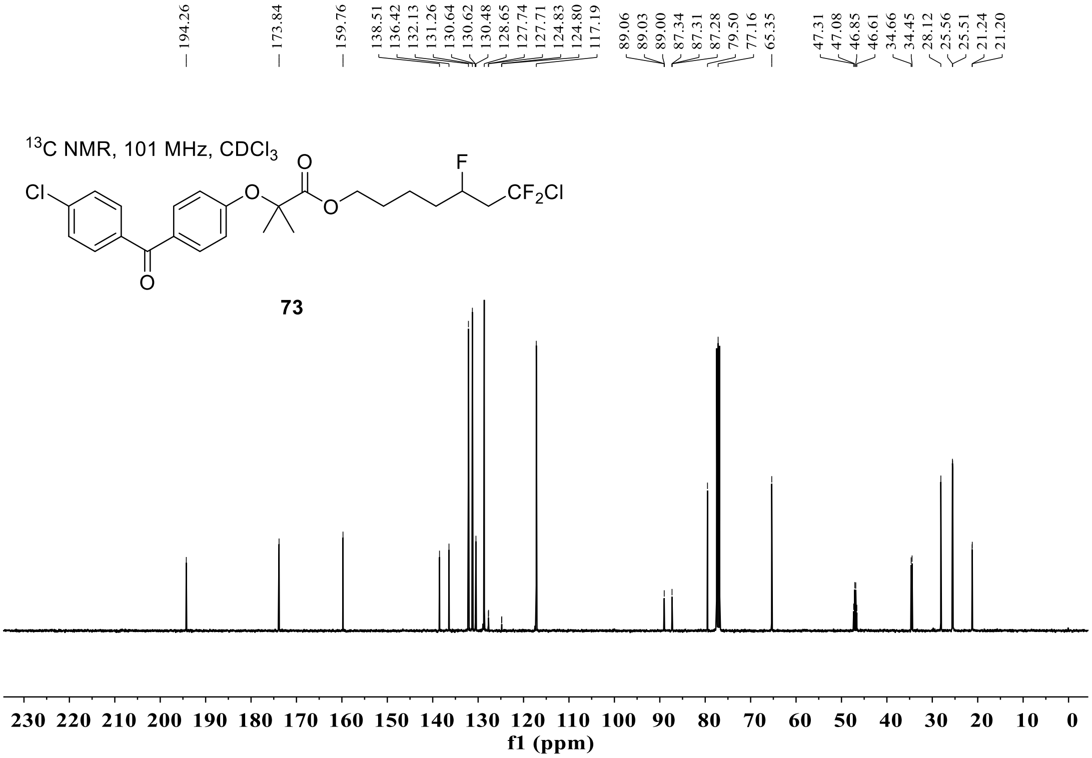
194.26
173.84
159.76
138.51 136.42 132.13 131.26 130.64 130.62 130.48 128.65 127.74 127.71 124.83 124.80 117.19
89.06 89.03 89.00 87.34 87.31 87.28 79.50 77.16 65.35
47.31 47.08 46.85 46.61 34.66 34.45 28.12 25.56 25.51 21.24 21.20
^{13}C NMR, 101 MHz, CDCl$_3$
Cl
O
O
O
O
F
CF$_2$Cl
73
230 220 210 200 190 180 170 160 150 140 130 120 110 100 90 80 70 60 50 40 30 20 10 0
f1 (ppm)

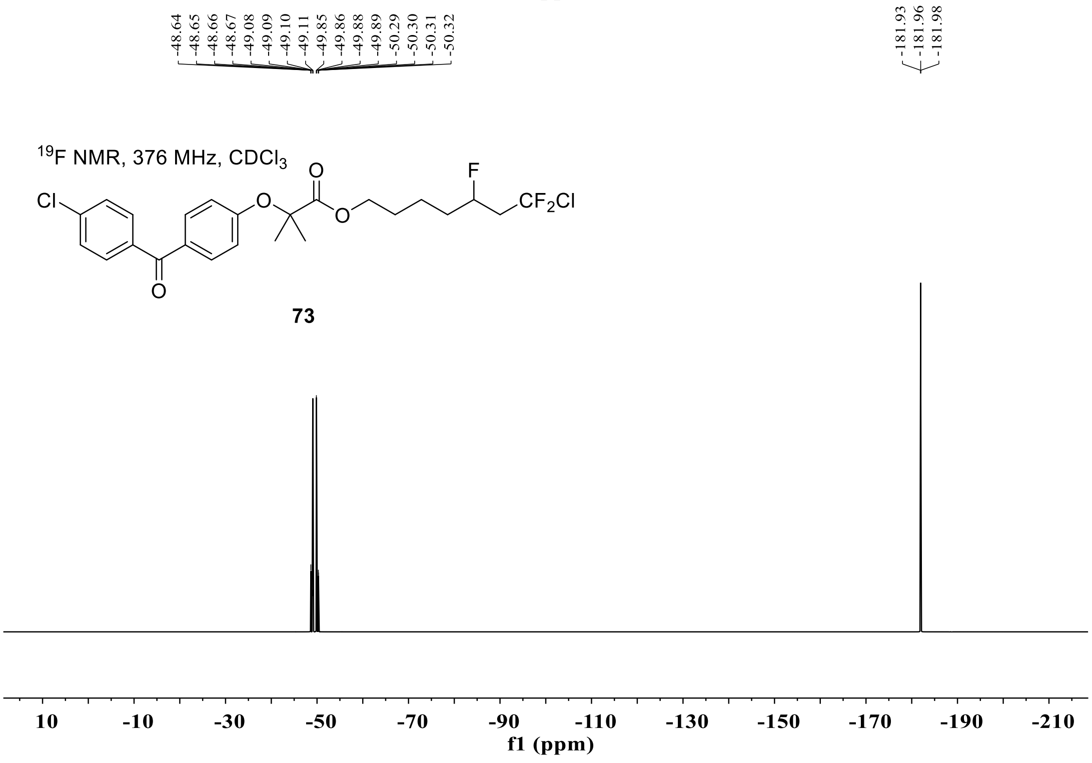
-48.64 -48.65 -48.66 -48.67 -49.08 -49.09 -49.10 -49.11 -49.85 -49.86 -49.88 -49.89 -50.29 -50.30 -50.31 -50.32
-181.93 -181.96 -181.98
^{19}F NMR, 376 MHz, CDCl$_3$
Cl
O
O
O
O
F
CF$_2$Cl
73
10 -10 -30 -50 -70 -90 -110 -130 -150 -170 -190 -210
f1 (ppm)

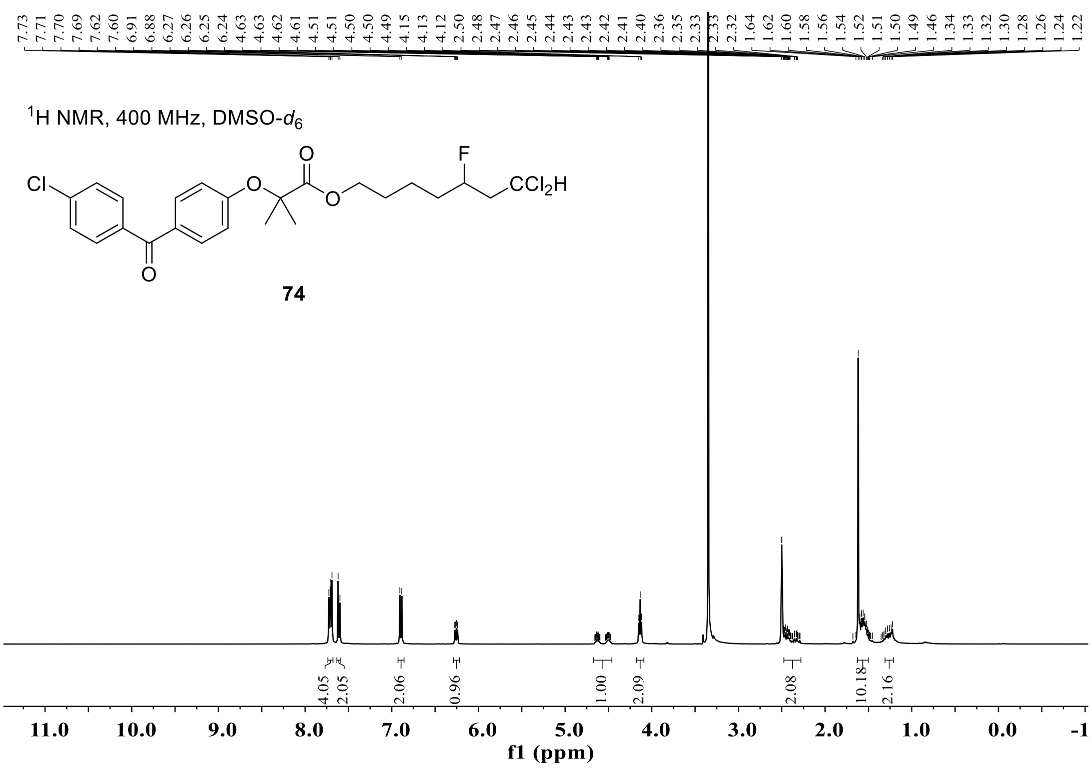

7.73
7.71
7.70
7.69
7.62
7.60
6.91
6.88
6.27
6.26
6.25
6.24
4.63
4.63
4.62
4.61
4.51
4.51
4.50
4.50
4.49
4.15
4.13
4.12
2.50
2.48
2.47
2.46
2.45
2.44
2.43
2.43
2.42
2.41
2.40
2.36
2.35
2.33
2.33
2.32
1.64
1.62
1.60
1.58
1.56
1.54
1.52
1.51
1.50
1.49
1.46
1.34
1.33
1.32
1.30
1.28
1.26
1.24
1.22
^{1}H NMR, 400 MHz, DMSO-d_6
Cl
O
O
O
F
CCl_2H
O
O
74
4.05
2.05
2.06
0.96
1.00
2.09
2.08
10.18
2.16
11.0 10.0 9.0 8.0 7.0 6.0 5.0 4.0 3.0 2.0 1.0 0.0 -1
f1 (ppm)

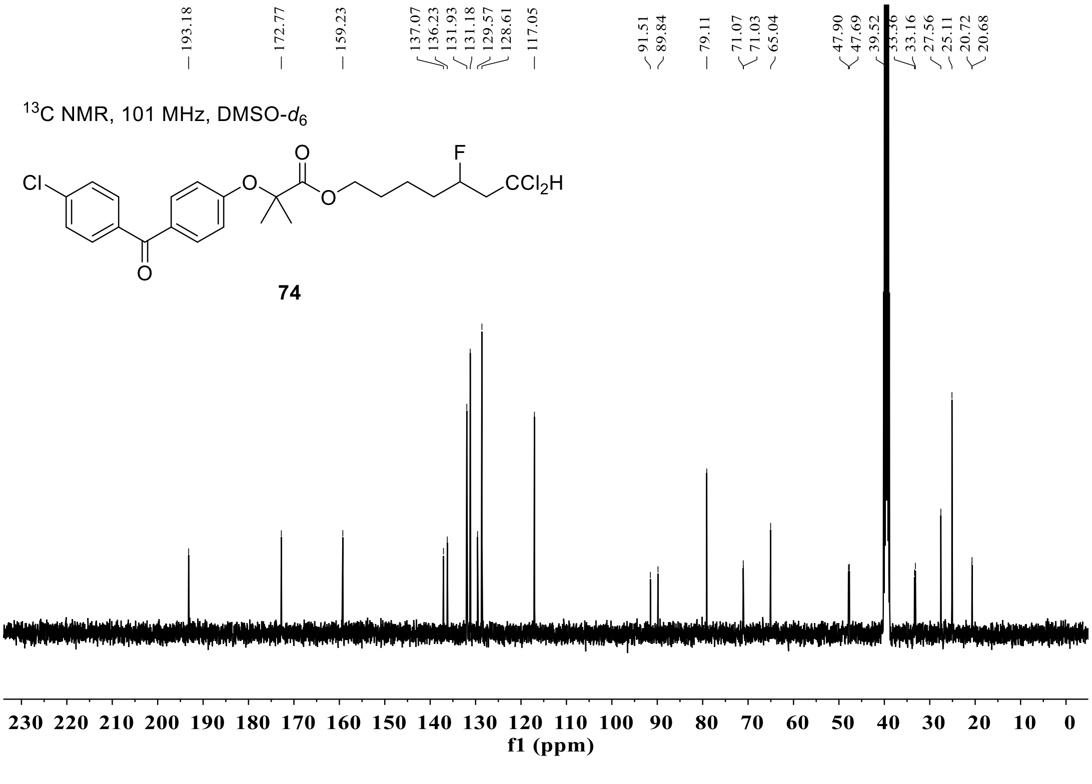

193.18
172.77
159.23
137.07
136.23
131.93
131.18
129.57
128.61
117.05
91.51
89.84
79.11
71.07
71.03
65.04
47.90
47.69
39.52
33.36
33.16
27.56
25.11
20.72
20.68
^{13}C NMR, 101 MHz, DMSO-d_6
Cl
O
O
O
F
CCl_2H
O
O
74
230 220 210 200 190 180 170 160 150 140 130 120 110 100 90 80 70 60 50 40 30 20 10 0
f1 (ppm)

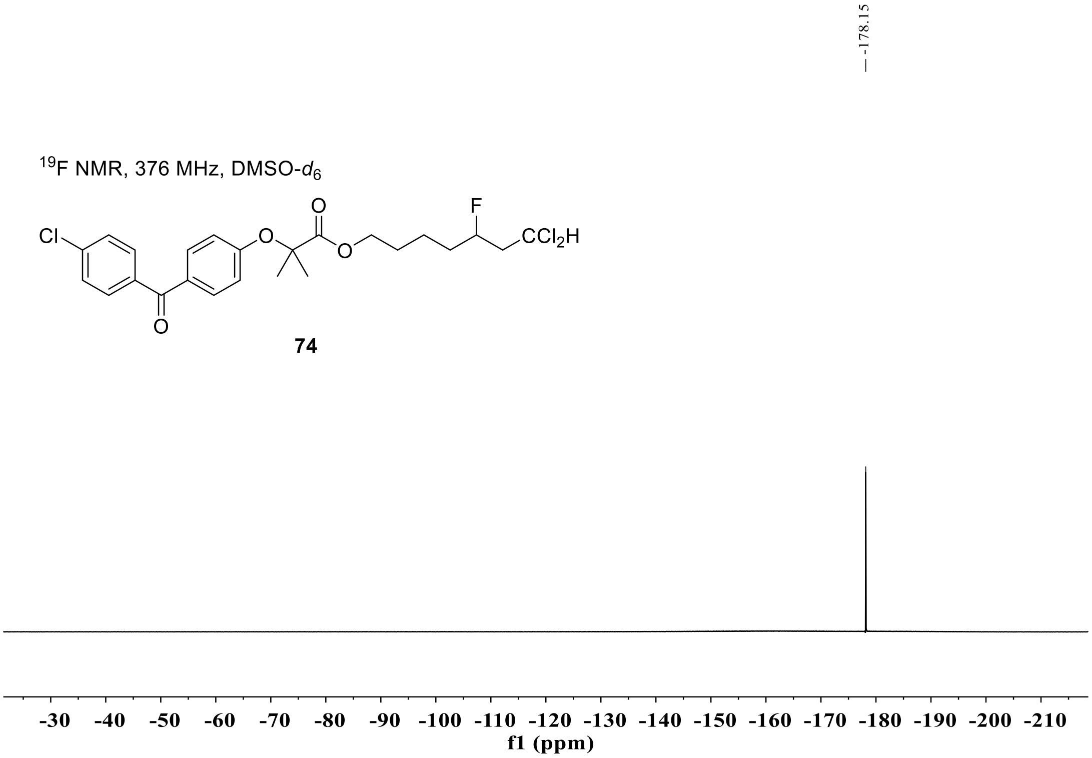
-178.15
19F NMR, 376 MHz, DMSO-d6
Cl
O
O
F
CCl2H
O
O
74
-30 -40 -50 -60 -70 -80 -90 -100 -110 -120 -130 -140 -150 -160 -170 -180 -190 -200 -210
f1 (ppm)

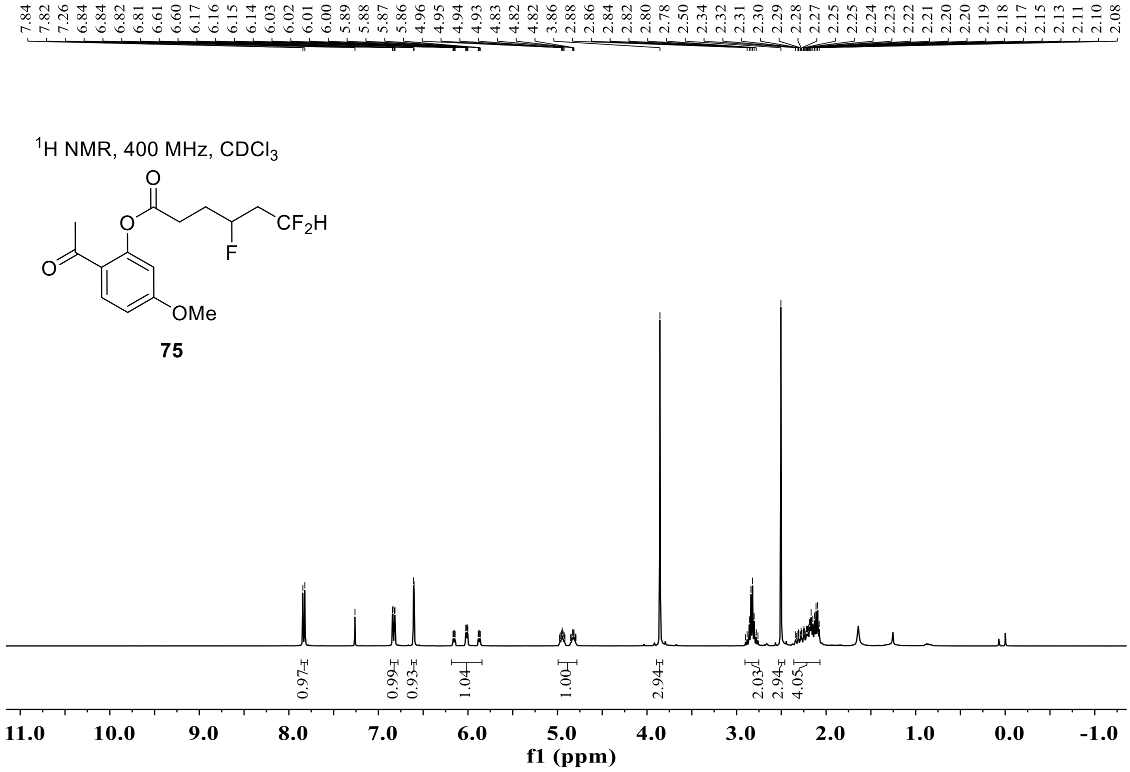
7.84 7.82 7.26 6.84 6.84 6.82 6.81 6.61 6.60 6.17 6.16 6.15 6.14 6.03 6.02 6.01 6.00 5.89 5.88 5.87 5.86 4.96 4.95 4.94 4.93 4.83 4.82 4.82 3.86 2.88 2.86 2.84 2.82 2.80 2.78 2.50 2.34 2.32 2.31 2.30 2.29 2.28 2.27 2.25 2.25 2.24 2.23 2.22 2.21 2.20 2.20 2.19 2.18 2.17 2.15 2.13 2.11 2.10 2.08
1H NMR, 400 MHz, CDCl3
O
O
CF2H
F
O
OMe
75
0.97 0.99 0.93 1.04 1.00 2.94 2.03 2.94 4.05
11.0 10.0 9.0 8.0 7.0 6.0 5.0 4.0 3.0 2.0 1.0 0.0 -1.0
f1 (ppm)

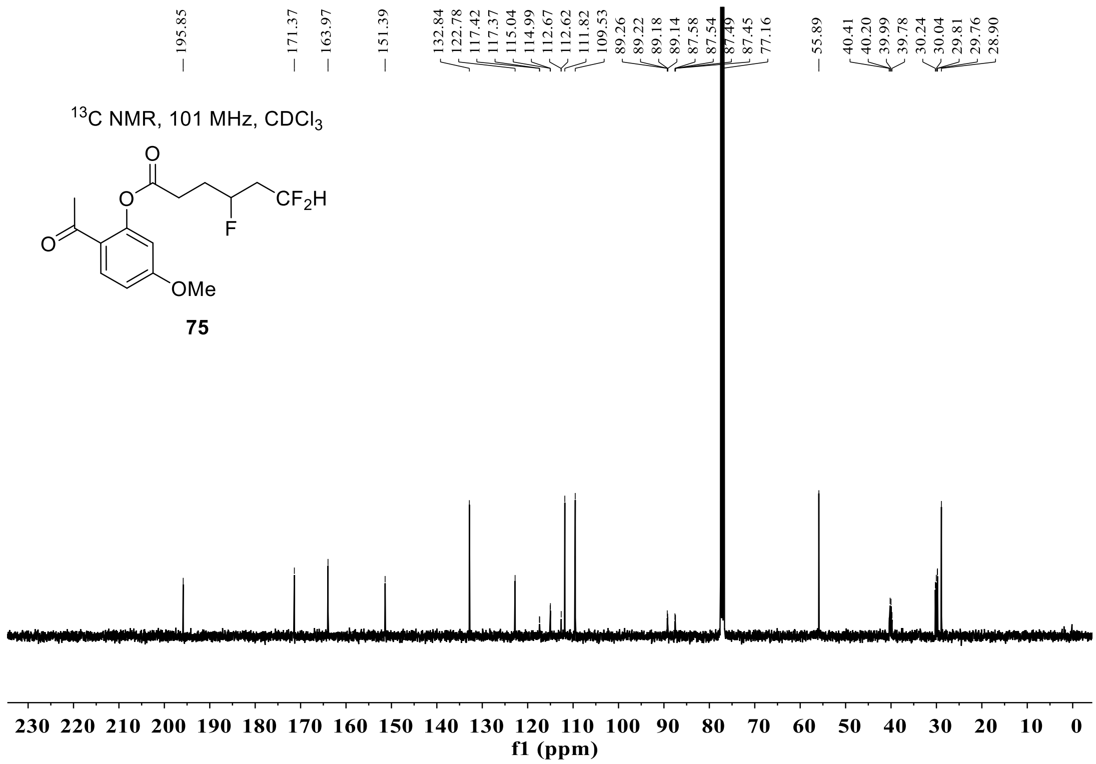

195.85
171.37
163.97
151.39
132.84
122.78
117.42
117.37
115.04
114.99
112.67
112.62
111.82
109.53
89.26
89.22
89.18
89.14
87.58
87.54
87.49
87.45
77.16
55.89
40.41
40.20
39.99
39.78
30.24
30.04
29.81
29.76
28.90
^{13}C NMR, 101 MHz, CDCl$_3$
O
O
CF$_2$H
F
O
OMe
75
230 220 210 200 190 180 170 160 150 140 130 120 110 100 90 80 70 60 50 40 30 20 10 0
f1 (ppm)

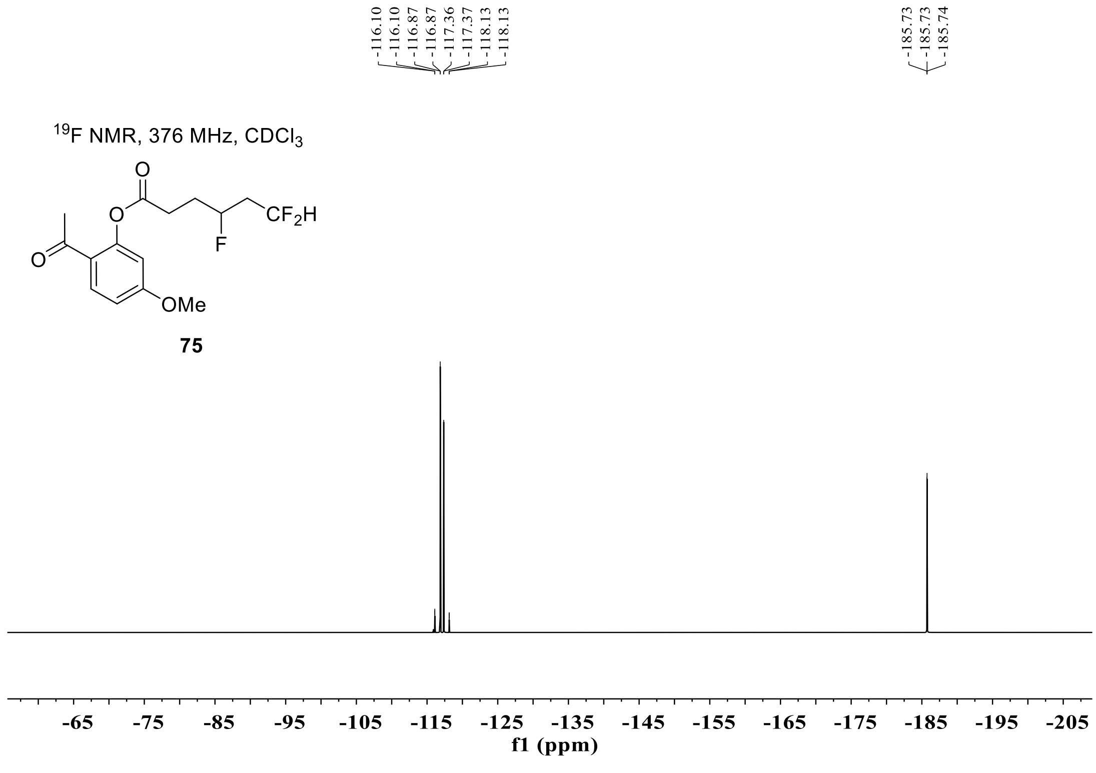

-116.10
-116.10
-116.87
-116.87
-117.36
-117.37
-118.13
-118.13
-185.73
-185.73
-185.74
^{19}F NMR, 376 MHz, CDCl$_3$
O
O
CF$_2$H
F
O
OMe
75
-65 -75 -85 -95 -105 -115 -125 -135 -145 -155 -165 -175 -185 -195 -205
f1 (ppm)

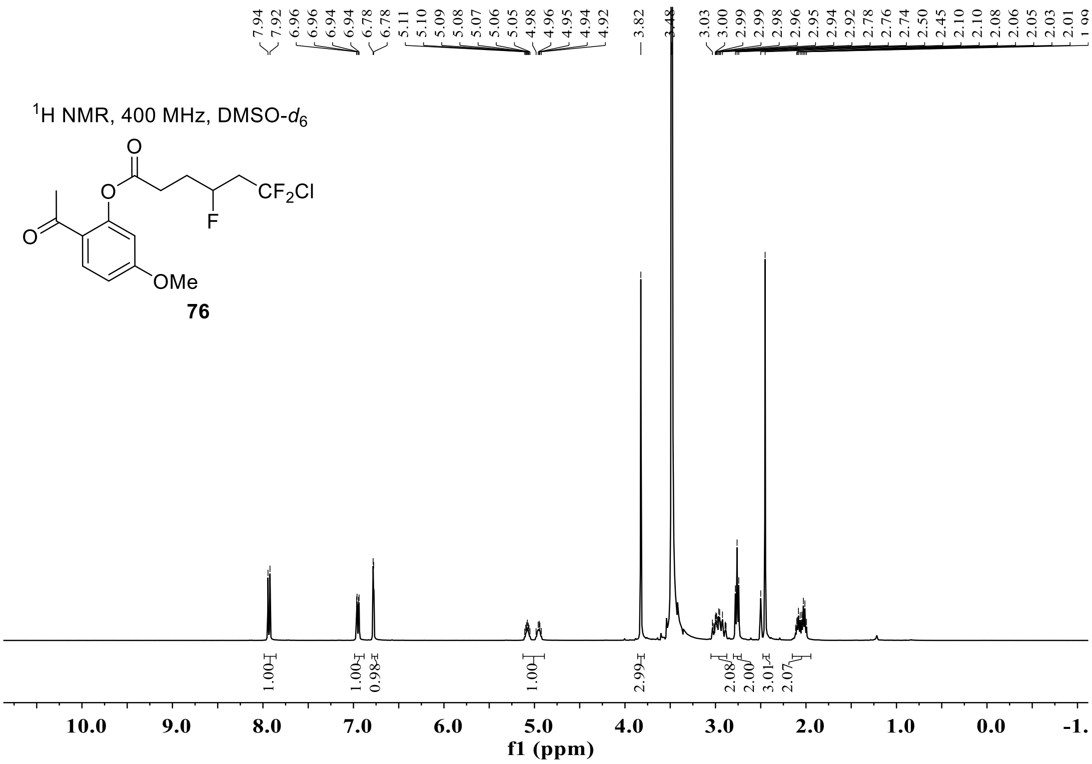
7.94 7.92 6.96 6.96 6.94 6.94 6.78 6.78 5.11 5.10 5.09 5.08 5.07 5.06 5.05 4.98 4.96 4.95 4.94 4.92 3.82 3.48 3.03 3.00 2.99 2.99 2.98 2.96 2.95 2.94 2.92 2.78 2.76 2.74 2.50 2.45 2.10 2.08 2.06 2.05 2.03 2.01
^{1}H NMR, 400 MHz, DMSO-d_6
O
O
CF$_2$Cl
F
O
OMe
76
1.00 1.00 0.98 1.00 2.99 2.08 2.00 3.01 2.07
10.0 9.0 8.0 7.0 6.0 5.0 4.0 3.0 2.0 1.0 0.0 -1.
f1 (ppm)

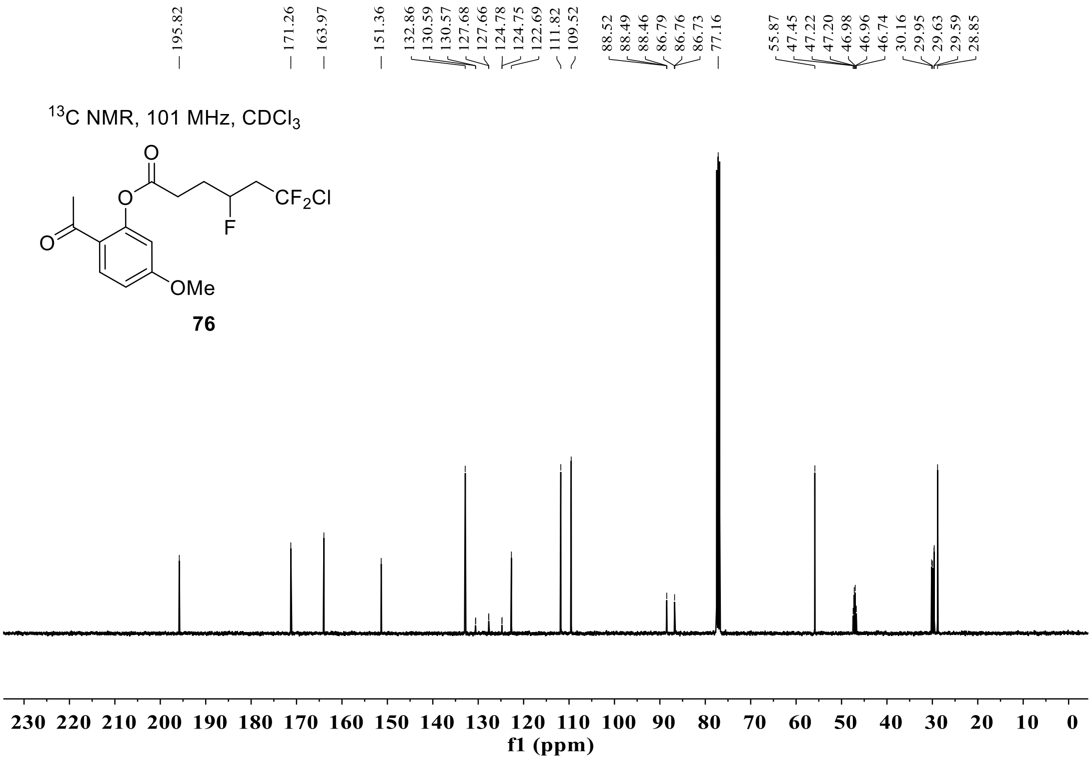
195.82 171.26 163.97 151.36 132.86 130.59 130.57 127.68 127.66 124.78 124.75 122.69 111.82 109.52 88.52 88.49 88.46 86.79 86.76 86.73 77.16 55.87 47.45 47.22 47.20 46.98 46.96 46.74 30.16 29.95 29.63 29.59 28.85
^{13}C NMR, 101 MHz, CDCl$_3$
O
O
CF$_2$Cl
F
O
OMe
76
230 220 210 200 190 180 170 160 150 140 130 120 110 100 90 80 70 60 50 40 30 20 10 0
f1 (ppm)

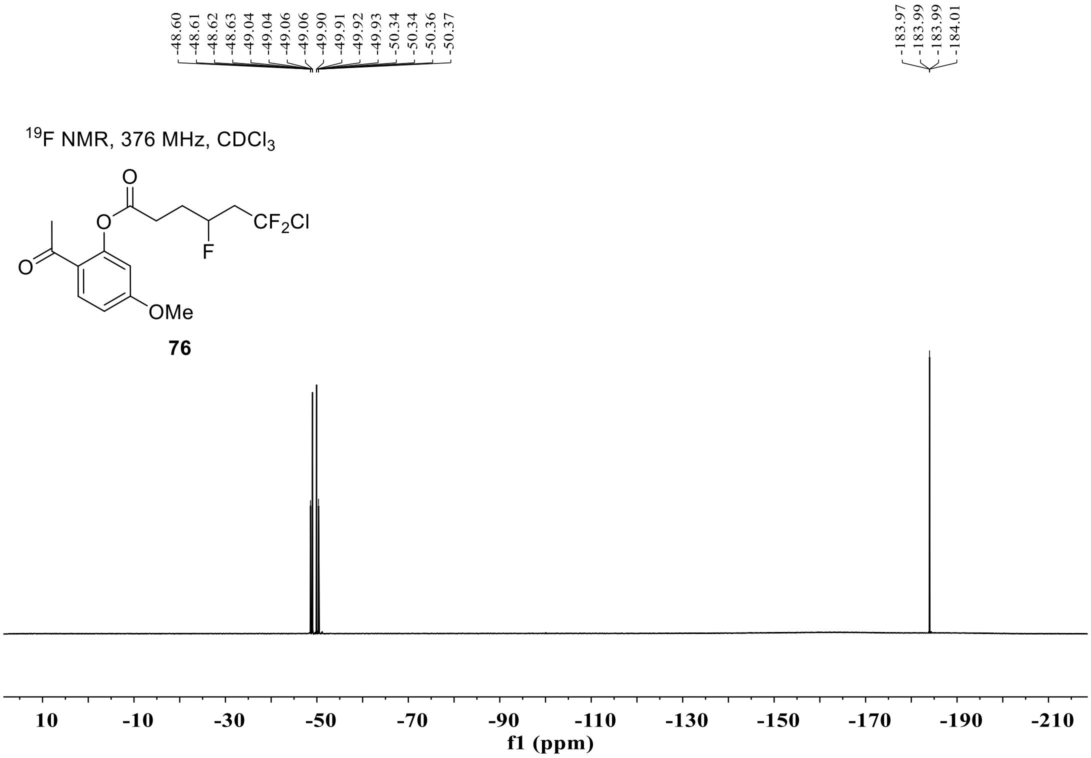
-48.60 -48.61 -48.62 -48.63 -49.04 -49.04 -49.06 -49.06 -49.90 -49.91 -49.92 -49.93 -50.34 -50.34 -50.36 -50.37
-183.97 -183.99 -183.99 -184.01
^{19}F NMR, 376 MHz, $CDCl_3$
O
O
CF_2Cl
F
O
OMe
76
10 -10 -30 -50 -70 -90 -110 -130 -150 -170 -190 -210
f1 (ppm)

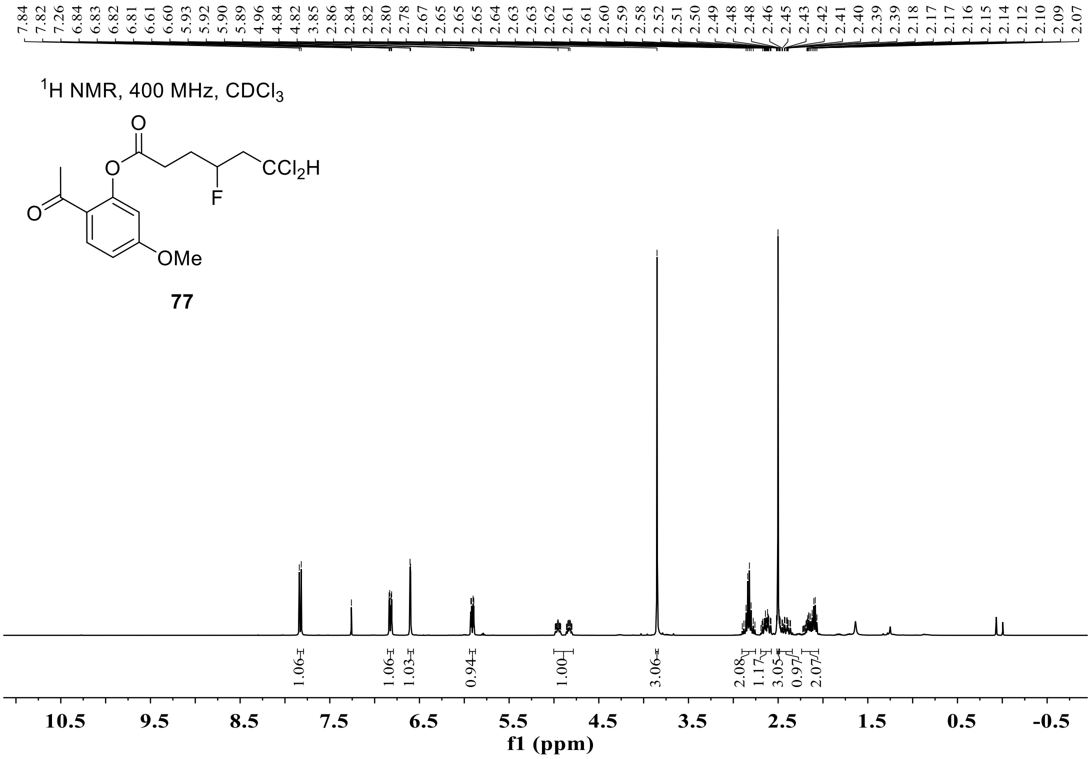
7.84 7.82 7.26 6.84 6.83 6.82 6.81 6.61 6.60 5.93 5.92 5.90 5.89 4.96 4.84 4.82 3.85 2.86 2.84 2.82 2.80 2.78 2.67 2.65 2.65 2.64 2.63 2.62 2.61 2.60 2.59 2.58 2.52 2.51 2.50 2.49 2.48 2.48 2.46 2.45 2.43 2.42 2.41 2.40 2.39 2.18 2.17 2.17 2.16 2.15 2.14 2.12 2.10 2.09 2.07
^{1}H NMR, 400 MHz, $CDCl_3$
O
O
CCl_2H
F
O
OMe
77
1.06 1.06 1.03 0.94 1.00 3.06 2.08 1.17 3.05 0.97 2.07
10.5 9.5 8.5 7.5 6.5 5.5 4.5 3.5 2.5 1.5 0.5 -0.5
f1 (ppm)

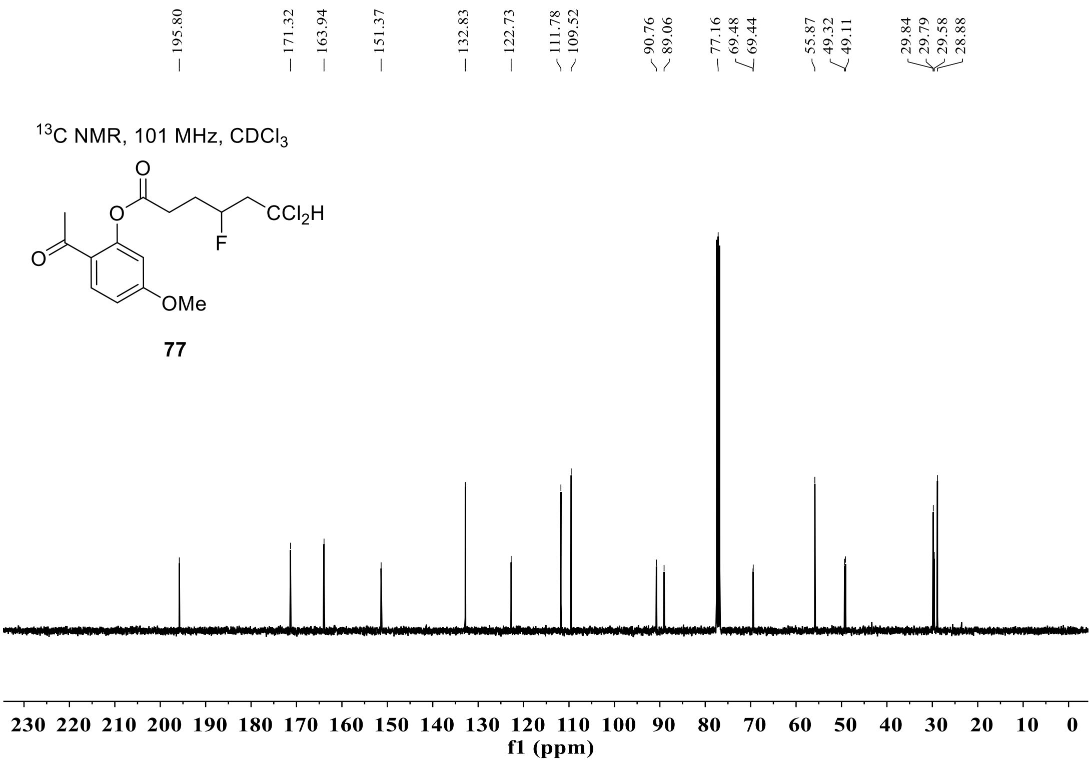
195.80
171.32
163.94
151.37
132.83
122.73
111.78
109.52
90.76
89.06
77.16
69.48
69.44
55.87
49.32
49.11
29.84
29.79
29.58
28.88
^{13}C NMR, 101 MHz, $CDCl_3$
O
O
CCl_2H
F
O
OMe
77
230 220 210 200 190 180 170 160 150 140 130 120 110 100 90 80 70 60 50 40 30 20 10 0
f1 (ppm)

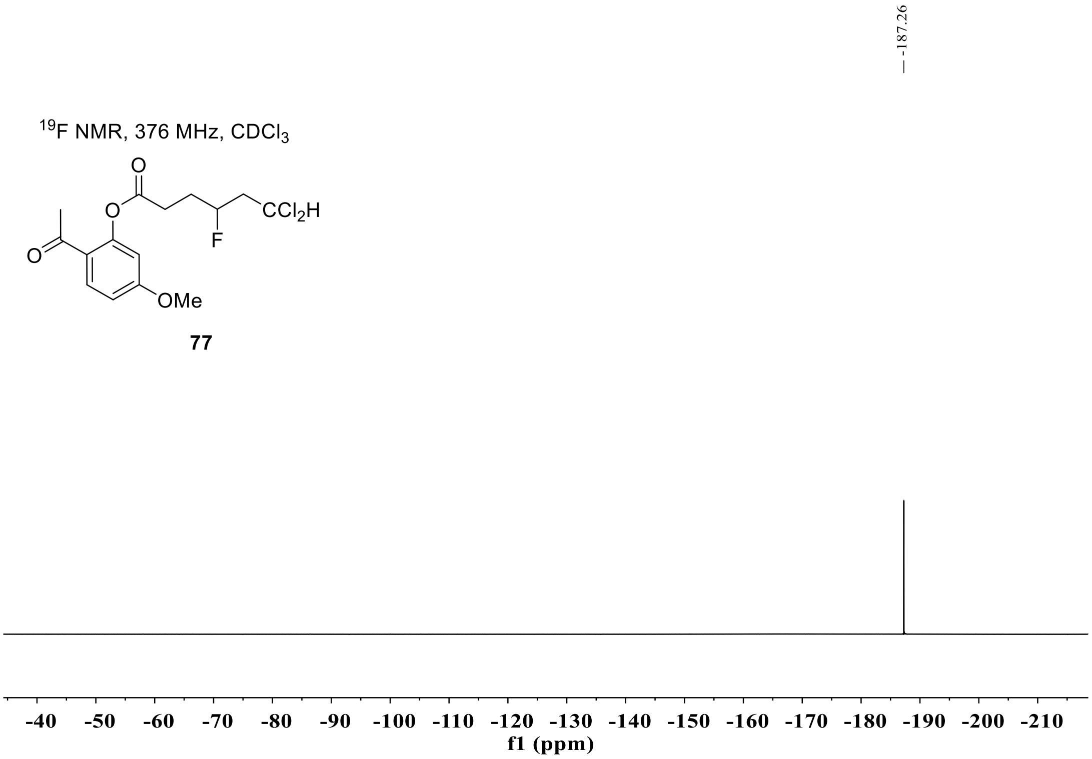
-187.26
^{19}F NMR, 376 MHz, $CDCl_3$
O
O
CCl_2H
F
O
OMe
77
-40 -50 -60 -70 -80 -90 -100 -110 -120 -130 -140 -150 -160 -170 -180 -190 -200 -210
f1 (ppm)

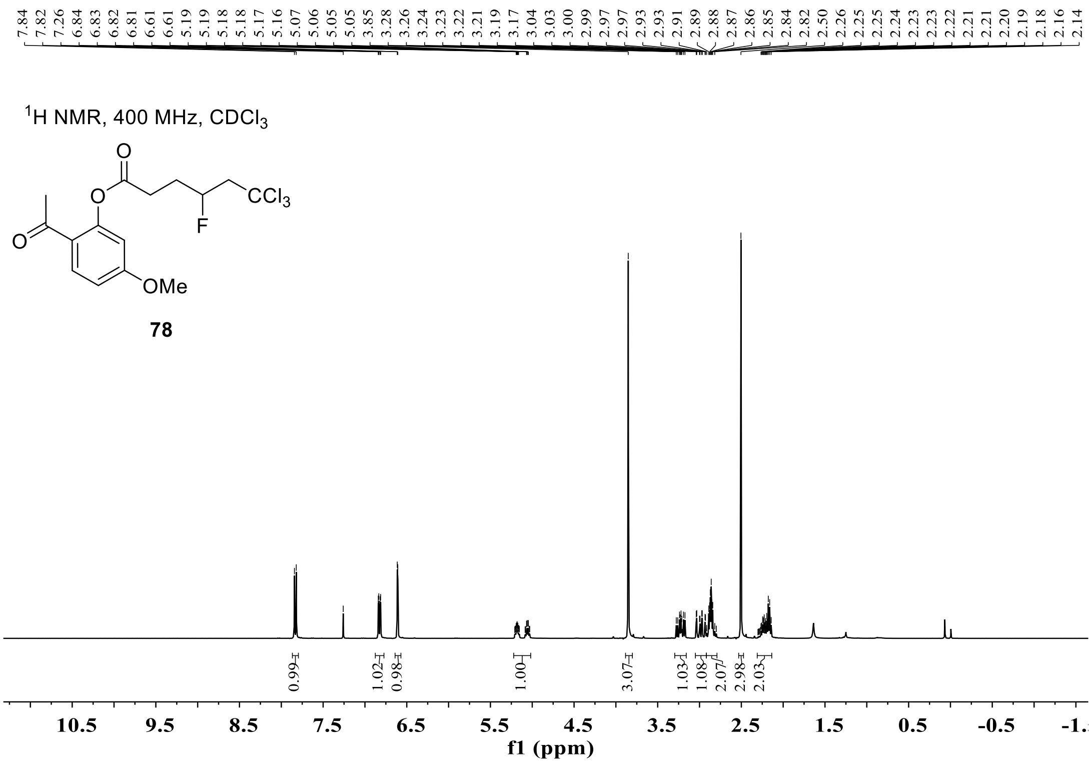
7.84 7.82 7.26 6.84 6.83 6.82 6.81 6.61 6.61 5.19 5.19 5.18 5.18 5.17 5.16 5.07 5.06 5.05 5.05 3.85 3.28 3.26 3.24 3.23 3.22 3.21 3.19 3.17 3.04 3.03 3.00 2.99 2.97 2.97 2.93 2.93 2.91 2.89 2.88 2.87 2.86 2.85 2.84 2.82 2.50 2.26 2.25 2.25 2.24 2.23 2.23 2.22 2.21 2.21 2.20 2.19 2.18 2.16 2.14
^{1}H NMR, 400 MHz, CDCl$_3$
O
O
CCl$_3$
F
O
OMe
78
0.99 1.02 0.98 1.00 3.07 1.03 1.08 2.07 2.98 2.03
10.5 9.5 8.5 7.5 6.5 5.5 4.5 3.5 2.5 1.5 0.5 -0.5
f1 (ppm)

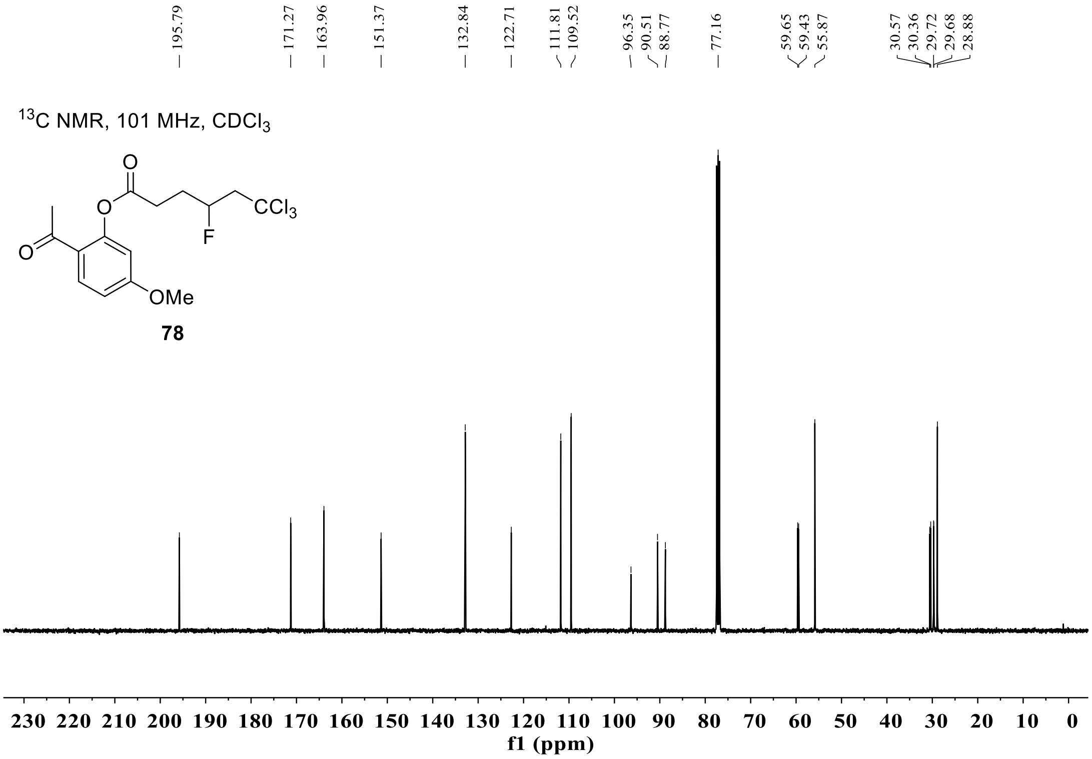
195.79 171.27 163.96 151.37 132.84 122.71 111.81 109.52 96.35 90.51 88.77 77.16 59.65 59.43 55.87 30.57 30.36 29.72 29.68 28.88
^{13}C NMR, 101 MHz, CDCl$_3$
O
O
CCl$_3$
F
O
OMe
78
230 220 210 200 190 180 170 160 150 140 130 120 110 100 90 80 70 60 50 40 30 20 10 0
f1 (ppm)

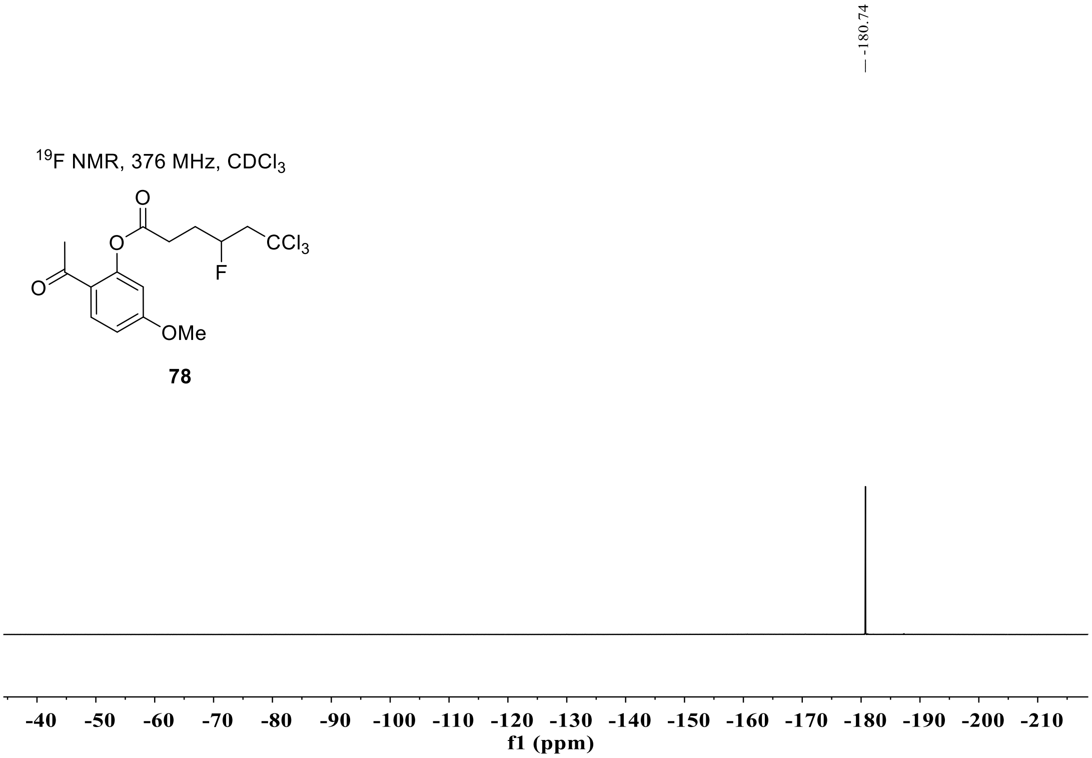
-180.74
19F NMR, 376 MHz, CDCl3
O
O
CCl3
F
O
OMe
78
-40 -50 -60 -70 -80 -90 -100 -110 -120 -130 -140 -150 -160 -170 -180 -190 -200 -210
f1 (ppm)

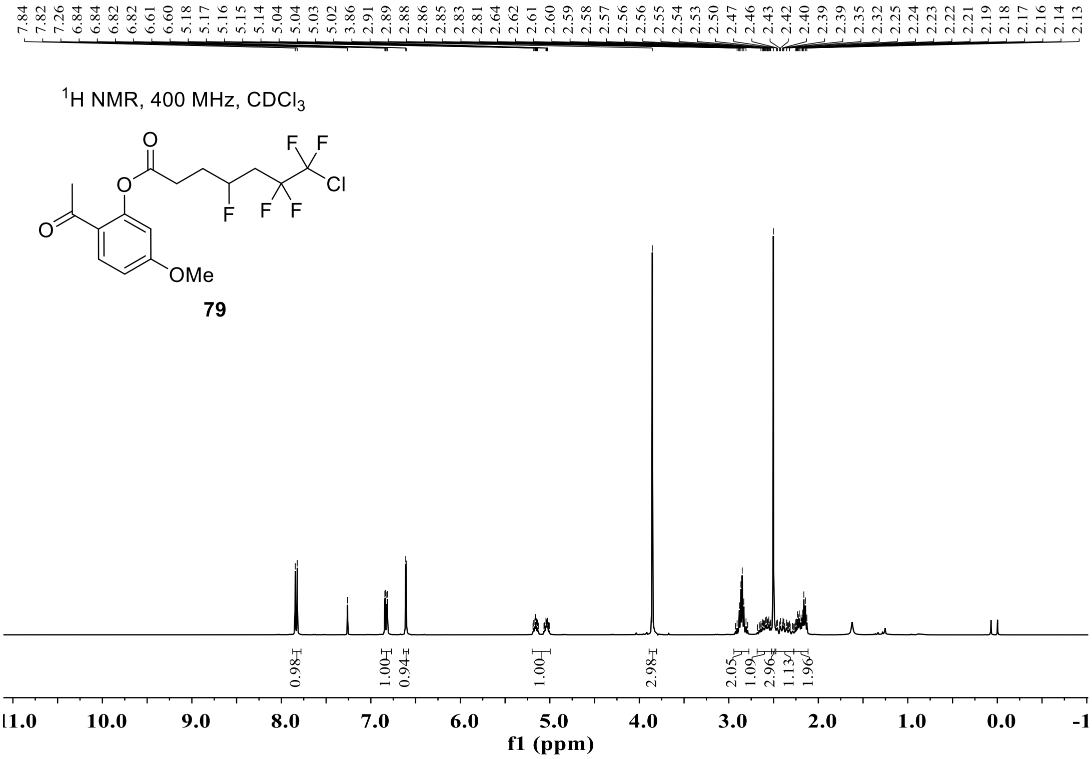
7.84 7.82 7.26 6.84 6.84 6.82 6.82 6.61 6.60 5.18 5.17 5.16 5.15 5.14 5.04 5.04 5.03 5.02 3.86 2.91 2.89 2.88 2.86 2.85 2.83 2.81 2.64 2.62 2.61 2.60 2.59 2.58 2.57 2.56 2.55 2.54 2.53 2.50 2.47 2.46 2.43 2.42 2.40 2.39 2.35 2.32 2.25 2.24 2.23 2.22 2.21 2.19 2.18 2.17 2.16 2.14 2.13
1H NMR, 400 MHz, CDCl3
F F
O
O
Cl
F
F F
O
OMe
79
0.98 1.00 0.94 1.00 2.98 2.05 1.09 2.96 1.13 1.96
11.0 10.0 9.0 8.0 7.0 6.0 5.0 4.0 3.0 2.0 1.0 0.0 -1
f1 (ppm)

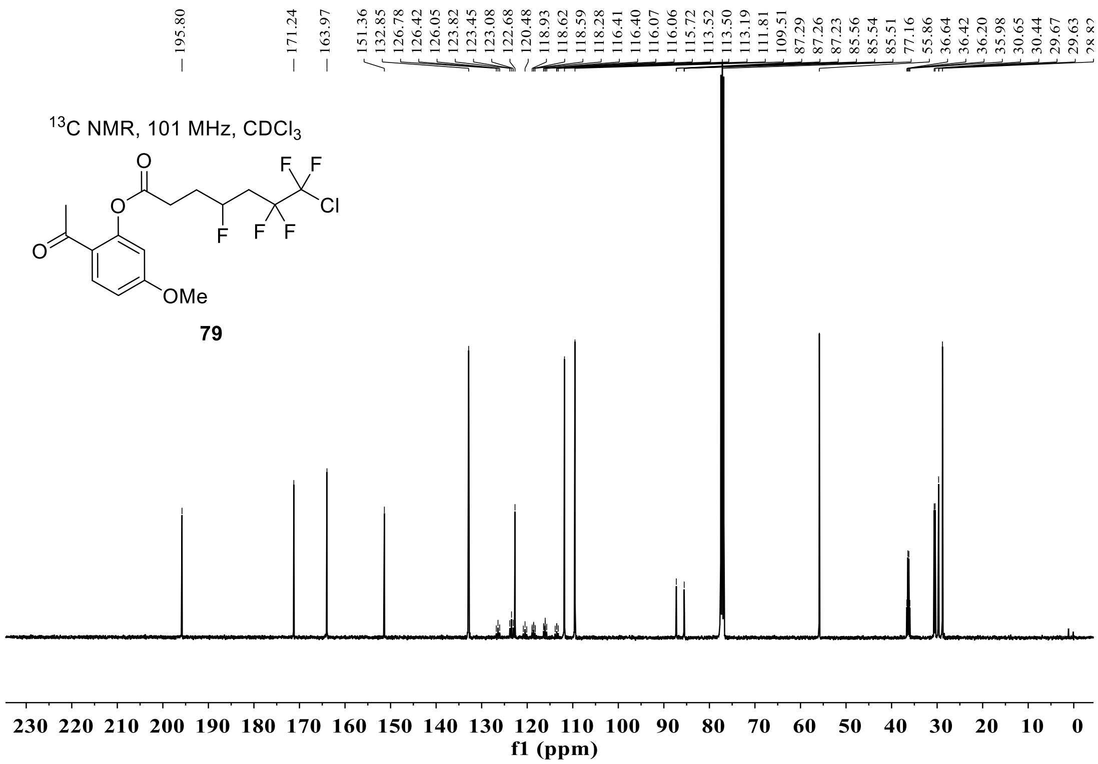
195.80
171.24
163.97
151.36
132.85
126.78
126.42
126.05
123.82
123.45
123.08
122.68
120.48
118.93
118.62
118.59
118.28
116.41
116.40
116.07
116.06
115.72
113.52
113.50
113.19
111.81
109.51
87.29
87.26
87.23
85.56
85.54
85.51
77.16
55.86
36.64
36.42
36.20
35.98
30.65
30.44
29.67
29.63
13C NMR, 101 MHz, CDCl3
O
F F
O
Cl
O
F F F
OMe
79
230 220 210 200 190 180 170 160 150 140 130 120 110 100 90 80 70 60 50 40 30 20 10 0
f1 (ppm)

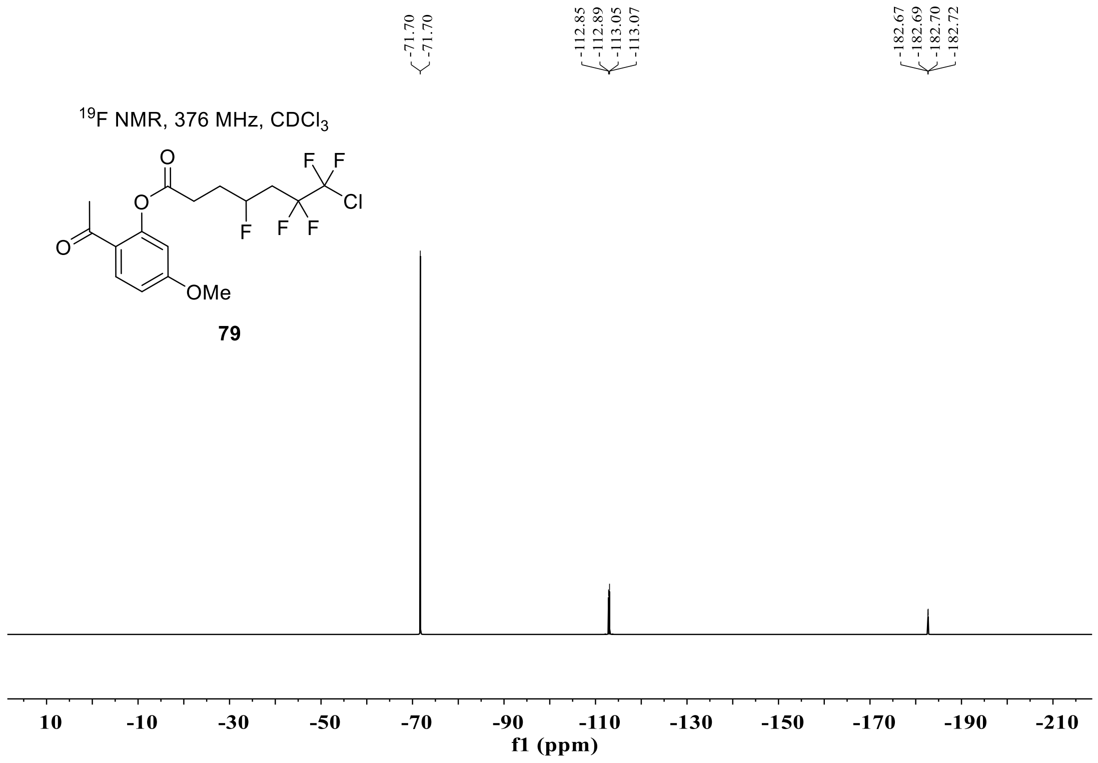
-71.70
-71.70
-112.85
-112.89
-113.05
-113.07
-182.67
-182.69
-182.70
-182.72
19F NMR, 376 MHz, CDCl3
O
F F
O
Cl
O
F F F
OMe
79
10 -10 -30 -50 -70 -90 -110 -130 -150 -170 -190 -210
f1 (ppm)

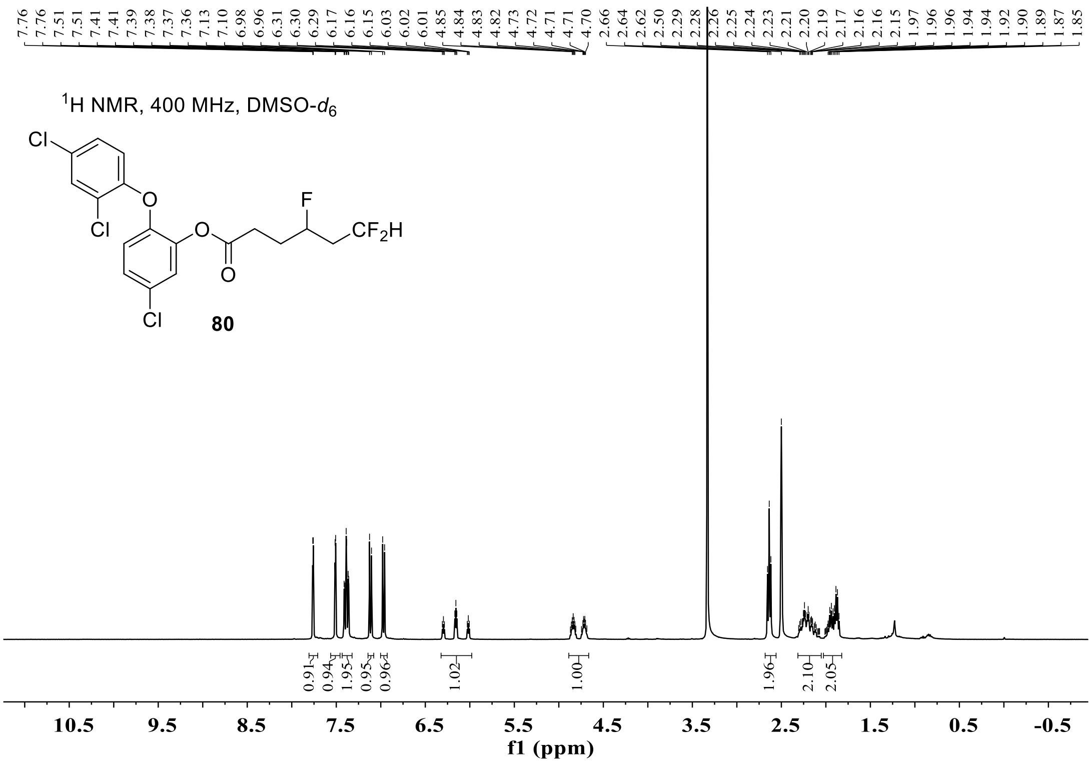
7.76 7.76 7.51 7.51 7.41 7.41 7.39 7.38 7.37 7.36 7.13 7.10 6.98 6.96 6.31 6.30 6.29 6.17 6.16 6.15 6.03 6.02 6.01 4.85 4.84 4.83 4.82 4.73 4.72 4.71 4.71 4.70 2.66 2.64 2.62 2.50 2.29 2.28 2.26 2.25 2.24 2.23 2.21 2.20 2.19 2.17 2.16 2.16 2.15 1.97 1.96 1.96 1.94 1.94 1.92 1.90 1.89 1.87 1.85
1H NMR, 400 MHz, DMSO-d6
Cl
O
Cl
O
F
CF2H
O
Cl
80
0.91 0.94 1.95 0.95 0.96 1.02 1.00 1.96 2.10 2.05
10.5 9.5 8.5 7.5 6.5 5.5 4.5 3.5 2.5 1.5 0.5 -0.5
f1 (ppm)

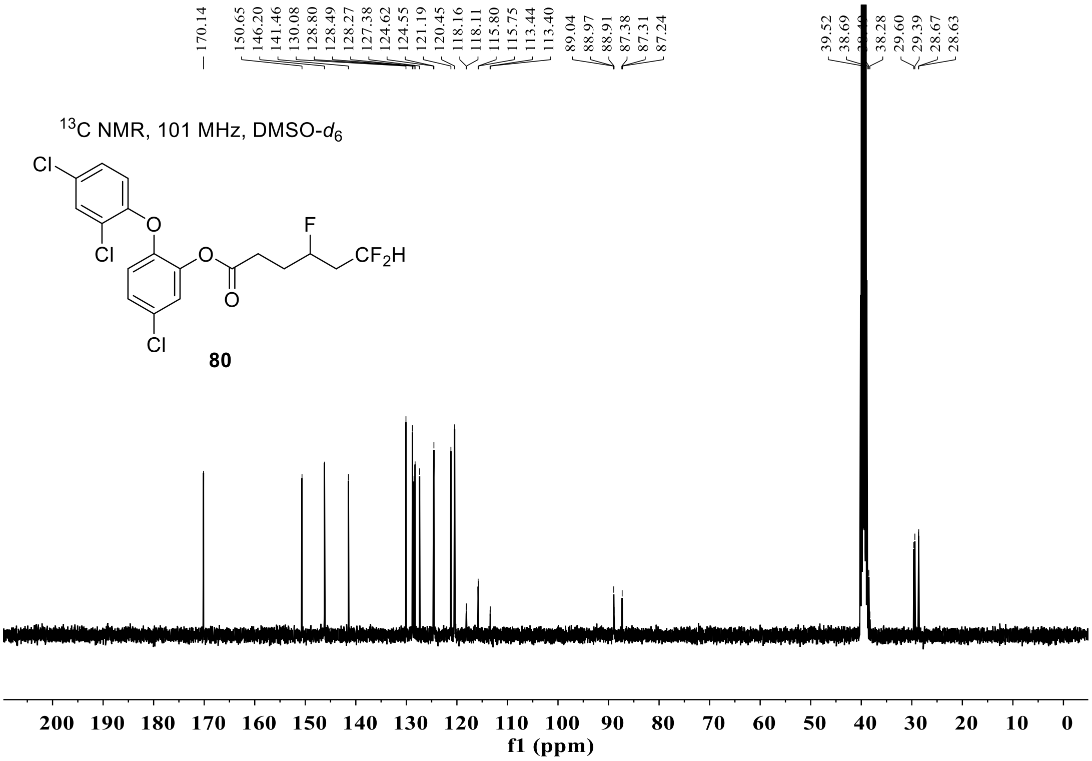
170.14
150.65 146.20 141.46 130.08 128.80 128.49 128.27 127.38 124.62 124.55 121.19 120.45 118.16 118.11 115.80 115.75 113.44 113.40
89.04 88.97 88.91 87.38 87.31 87.24
39.52 38.69 38.28 29.60 29.39 28.67 28.63
13C NMR, 101 MHz, DMSO-d6
Cl
O
Cl
O
F
CF2H
O
Cl
80
200 190 180 170 160 150 140 130 120 110 100 90 80 70 60 50 40 30 20 10 0
f1 (ppm)

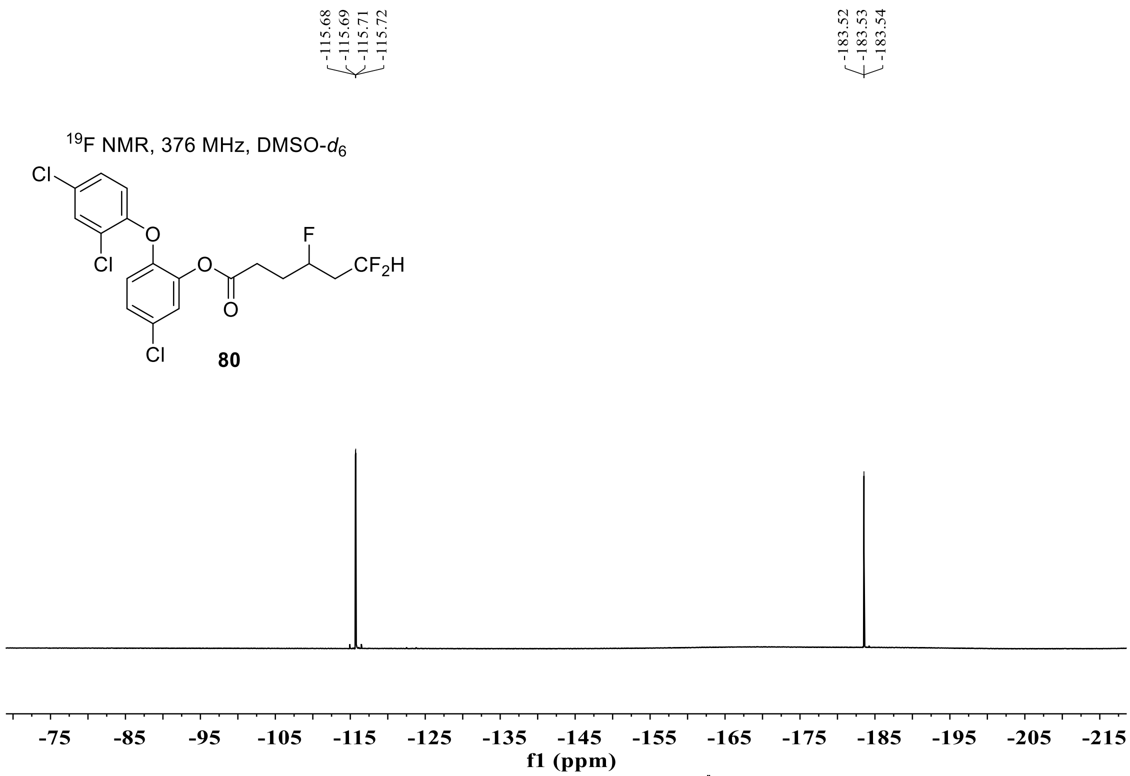
-115.68
-115.69
-115.71
-115.72
-183.52
-183.53
-183.54
19F NMR, 376 MHz, DMSO-d6
80
-75 -85 -95 -105 -115 -125 -135 -145 -155 -165 -175 -185 -195 -205 -215
f1 (ppm)

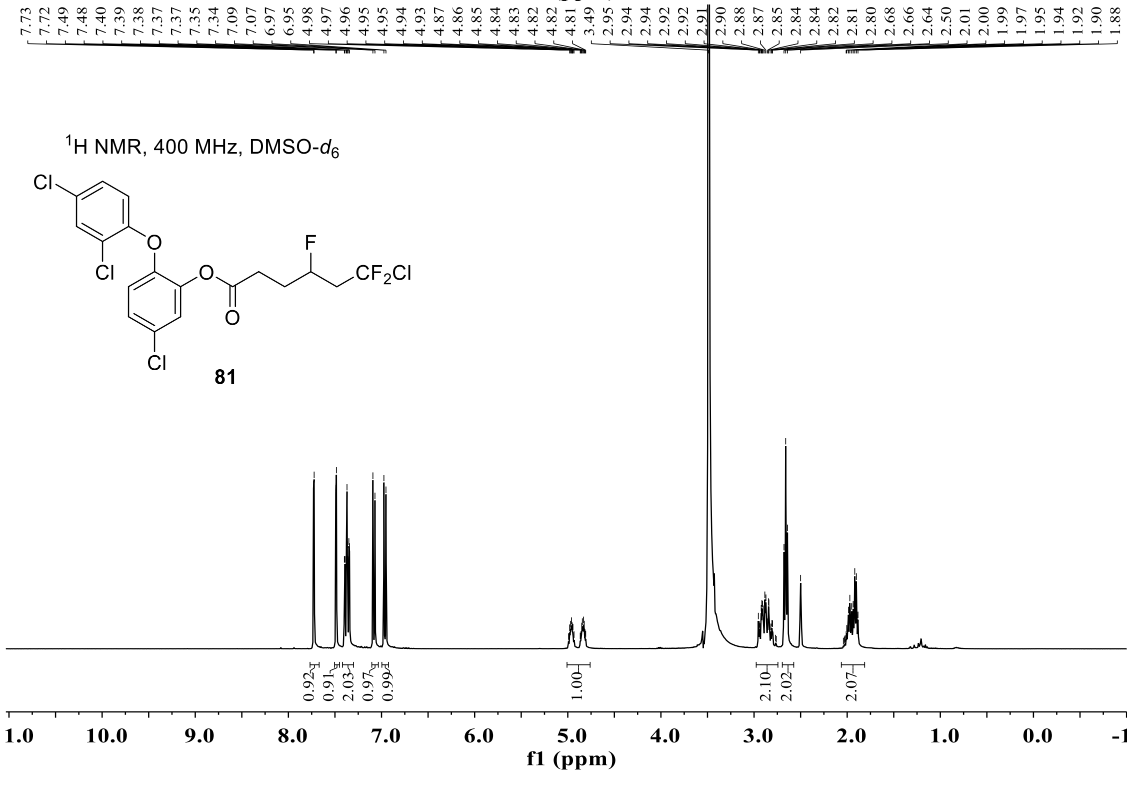
7.73 7.72 7.49 7.48 7.40 7.39 7.38 7.37 7.37 7.35 7.34 7.09 7.07 6.97 6.95 4.98 4.97 4.96 4.95 4.95 4.94 4.93 4.87 4.86 4.85 4.84 4.83 4.82 4.82 4.81 3.49 2.95 2.94 2.94 2.92 2.92 2.91 2.90 2.88 2.87 2.85 2.84 2.84 2.82 2.81 2.80 2.68 2.66 2.64 2.50 2.01 2.00 1.99 1.97 1.95 1.94 1.92 1.90 1.88
1H NMR, 400 MHz, DMSO-d6
81
0.92
0.91
2.03
0.97
0.99
1.00
2.10
2.02
2.07
1.0 10.0 9.0 8.0 7.0 6.0 5.0 4.0 3.0 2.0 1.0 0.0 -1
f1 (ppm)

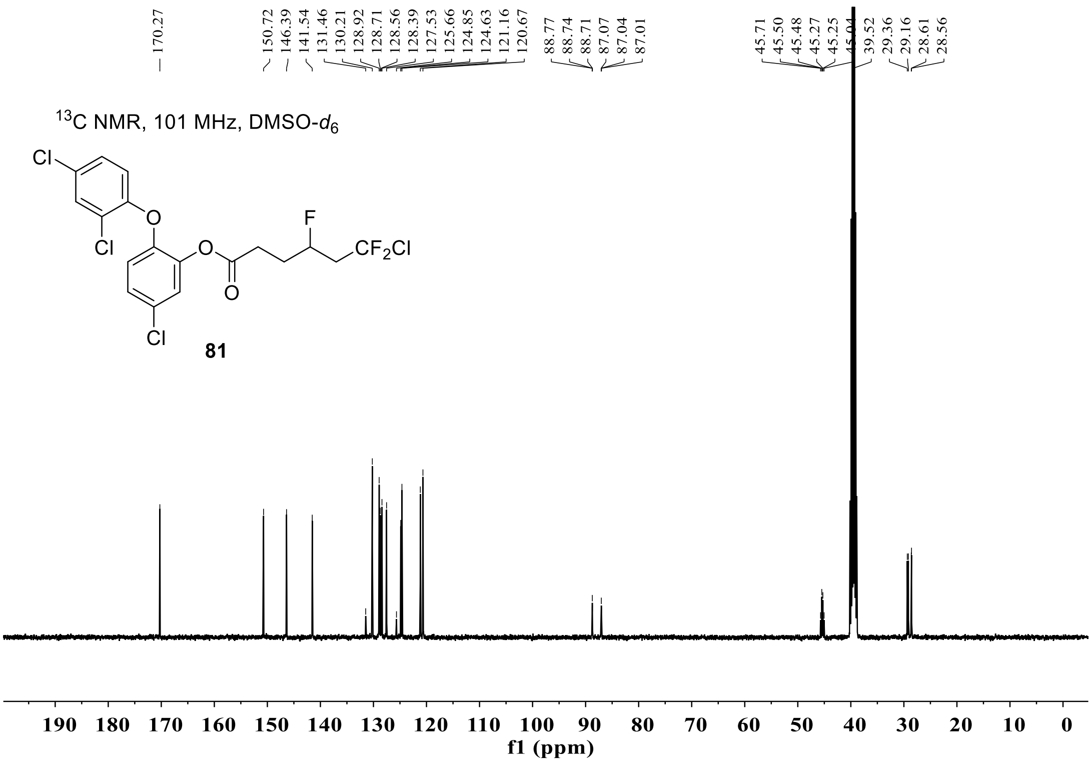

170.27
150.72
146.39
141.54
131.46
130.21
128.92
128.71
128.56
128.39
127.53
125.66
124.85
124.63
121.16
120.67
88.77
88.74
88.71
87.07
87.04
87.01
45.71
45.50
45.48
45.27
45.25
39.52
29.36
29.16
28.61
28.56
13C NMR, 101 MHz, DMSO-d6
Cl
O
Cl
O
O
F
CF2Cl
Cl
81
190 180 170 160 150 140 130 120 110 100 90 80 70 60 50 40 30 20 10 0
f1 (ppm)

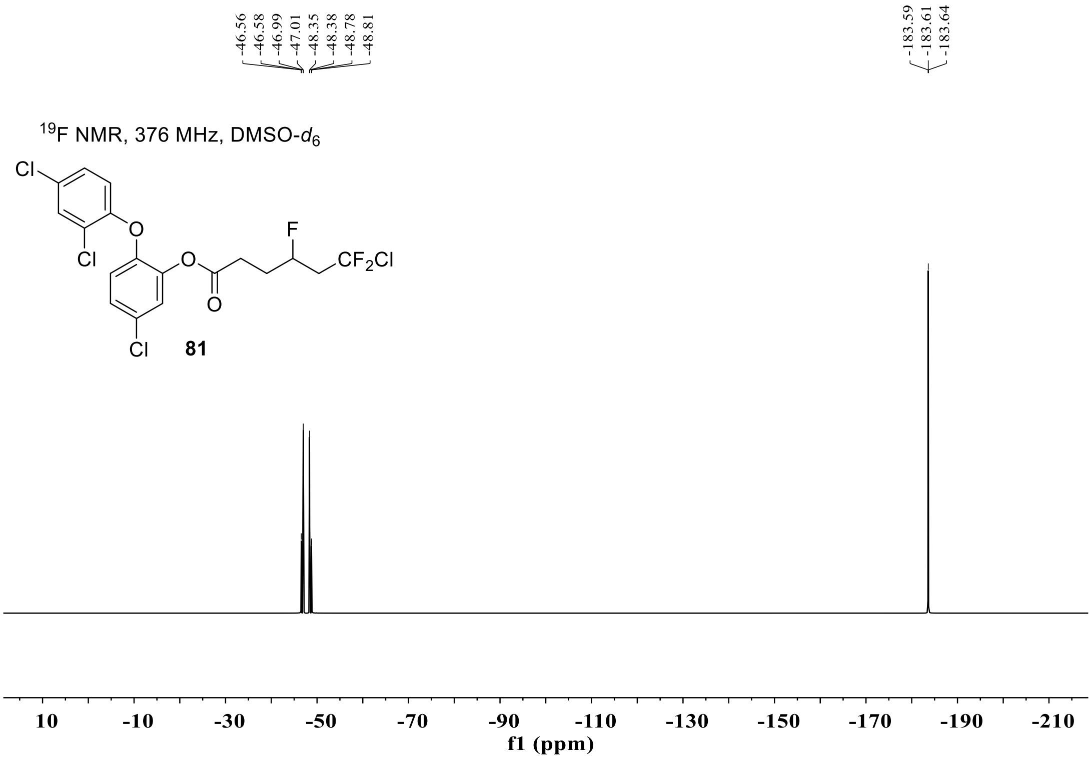

-46.56
-46.58
-46.99
-47.01
-48.35
-48.38
-48.78
-48.81
-183.59
-183.61
-183.64
19F NMR, 376 MHz, DMSO-d6
Cl
O
Cl
O
O
F
CF2Cl
Cl
81
10 -10 -30 -50 -70 -90 -110 -130 -150 -170 -190 -210
f1 (ppm)

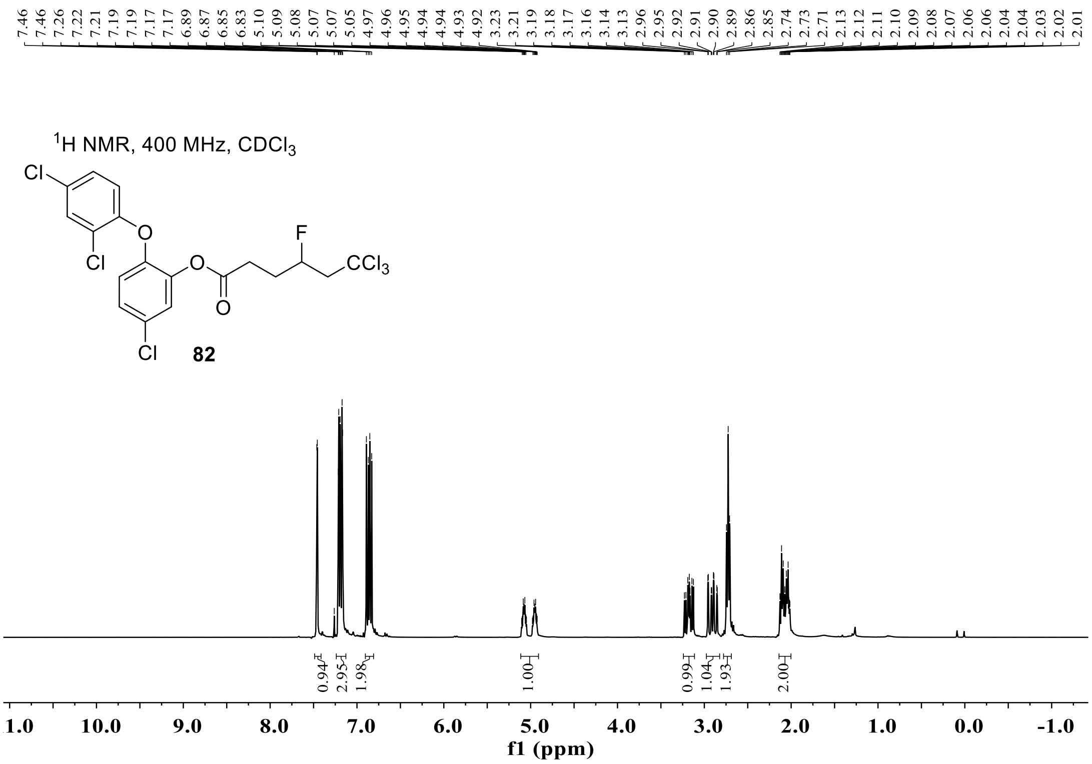
7.46 7.46 7.26 7.22 7.21 7.19 7.19 7.17 7.17 6.89 6.87 6.85 6.83 5.10 5.09 5.08 5.07 5.07 5.05 4.97 4.96 4.95 4.94 4.94 4.93 4.92 3.23 3.21 3.19 3.18 3.17 3.16 3.14 3.13 2.96 2.95 2.92 2.91 2.90 2.89 2.86 2.85 2.74 2.73 2.71 2.13 2.12 2.11 2.10 2.09 2.08 2.07 2.06 2.06 2.04 2.04 2.03 2.02 2.01
^{1}H NMR, 400 MHz, CDCl$_3$
Cl
O
Cl
O
F
CCl$_3$
O
Cl
82
0.94
2.95
1.98
1.00
0.99
1.04
1.93
2.00
1.0 10.0 9.0 8.0 7.0 6.0 5.0 4.0 3.0 2.0 1.0 0.0 -1.0
f1 (ppm)

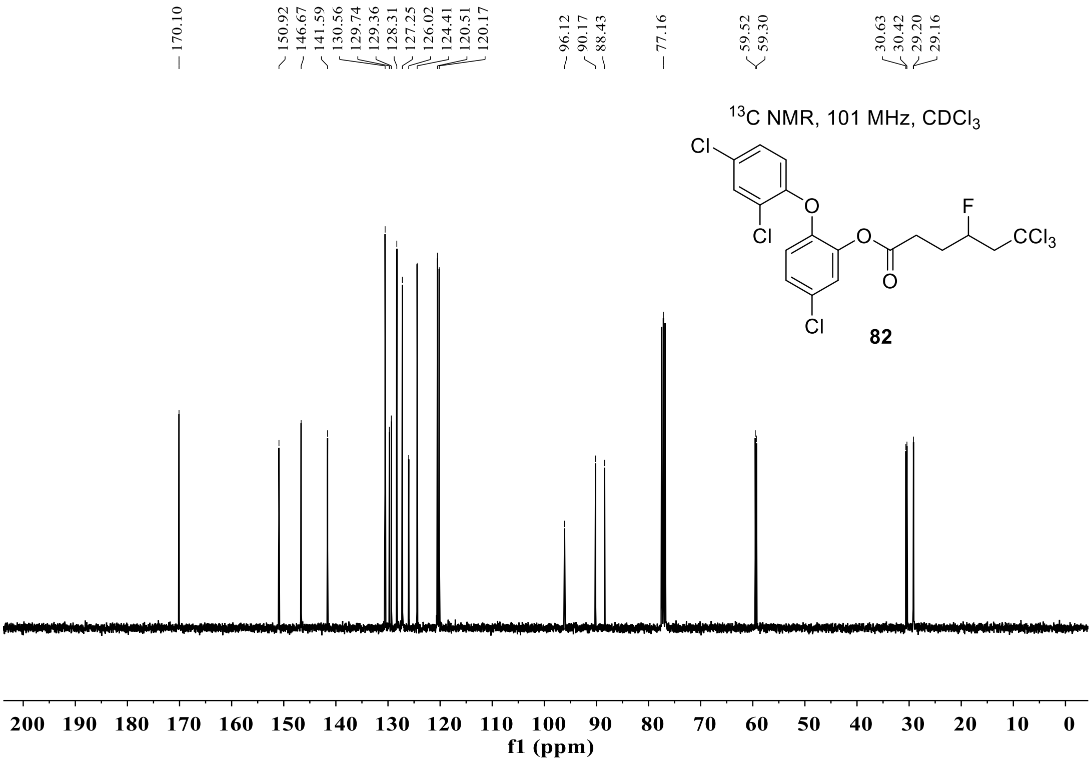
170.10
150.92 146.67 141.59 130.56 129.74 129.36 128.31 127.25 126.02 124.41 120.51 120.17
96.12 90.17 88.43
77.16
59.52 59.30
30.63 30.42 29.20 29.16
^{13}C NMR, 101 MHz, CDCl$_3$
Cl
O
Cl
O
F
CCl$_3$
O
Cl
82
200 190 180 170 160 150 140 130 120 110 100 90 80 70 60 50 40 30 20 10 0
f1 (ppm)

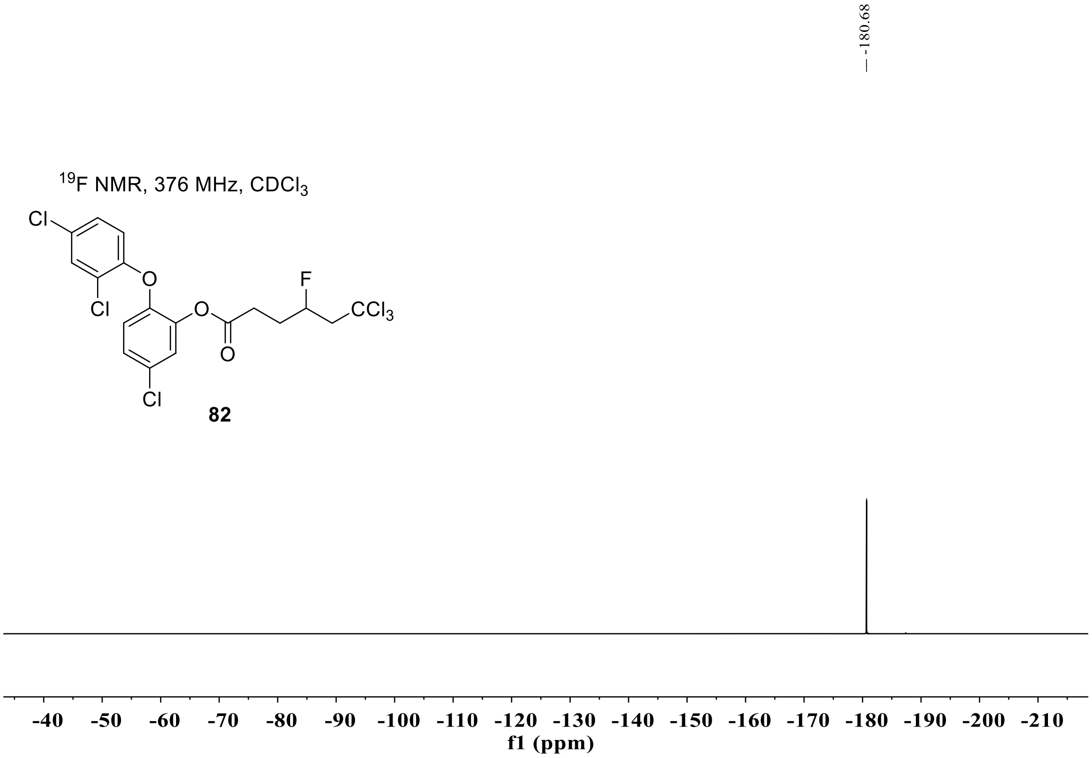

-180.68
^{19}F NMR, 376 MHz, CDCl$_3$
82
f1 (ppm)

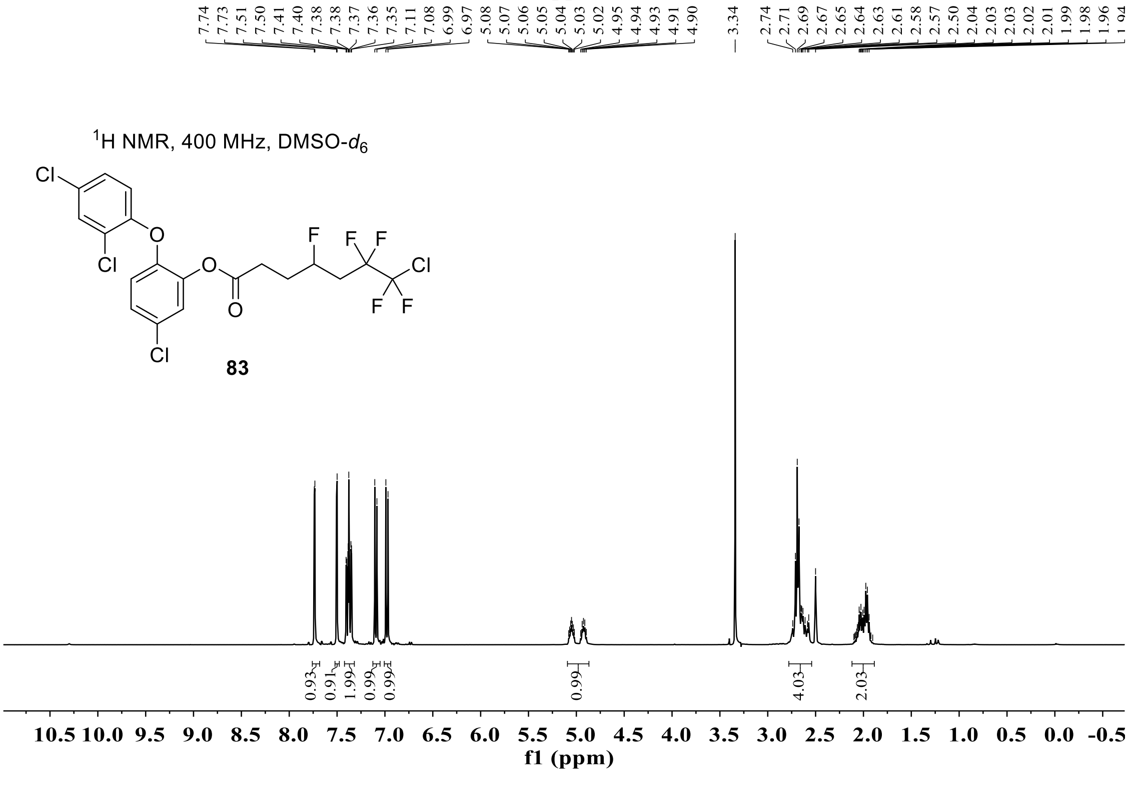

^{1}H NMR, 400 MHz, DMSO-d_6
83
f1 (ppm)

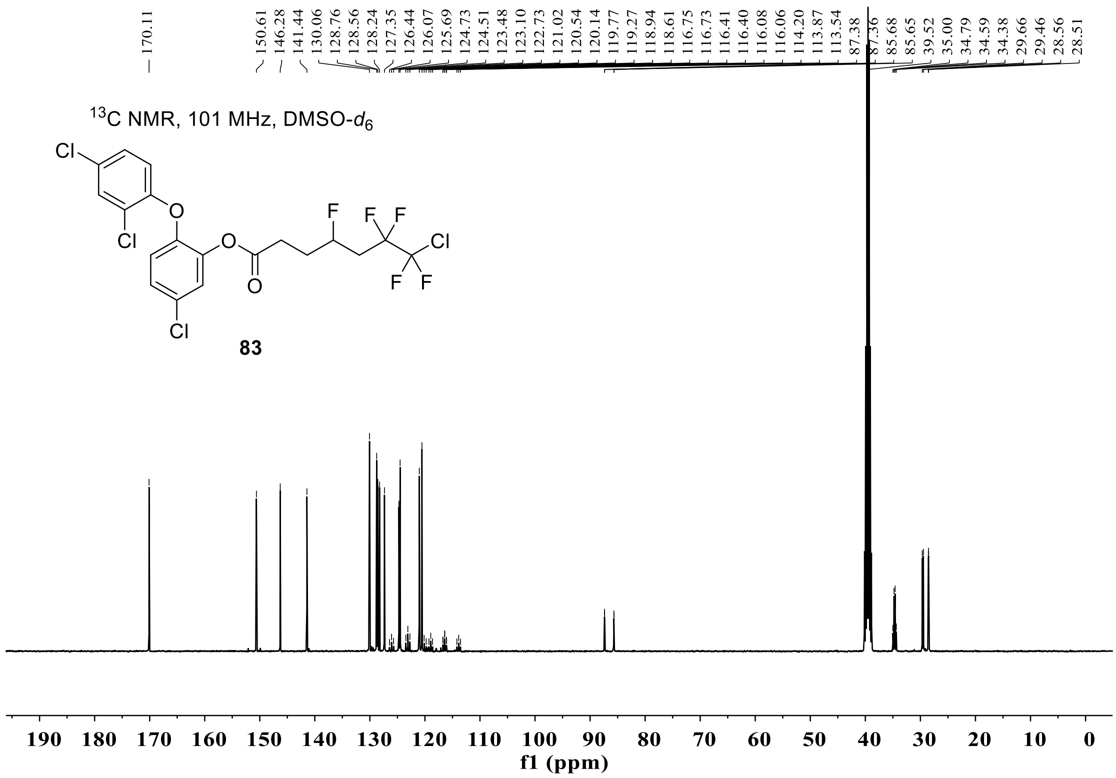
170.11
150.61
146.28
141.44
130.06
128.76
128.56
128.24
127.35
126.44
126.07
125.69
124.73
124.51
123.48
123.10
122.73
121.02
120.54
120.14
119.77
119.27
118.94
118.61
116.75
116.73
116.41
116.40
116.08
116.06
114.20
113.87
113.54
87.38
87.36
85.68
85.65
39.52
35.00
34.79
34.59
34.38
29.66
29.46
28.56
28.51
^{13}C NMR, 101 MHz, DMSO-d_6
Cl
O
Cl
O
F
F
F
Cl
O
F
F
Cl
83
190 180 170 160 150 140 130 120 110 100 90 80 70 60 50 40 30 20 10 0
f1 (ppm)

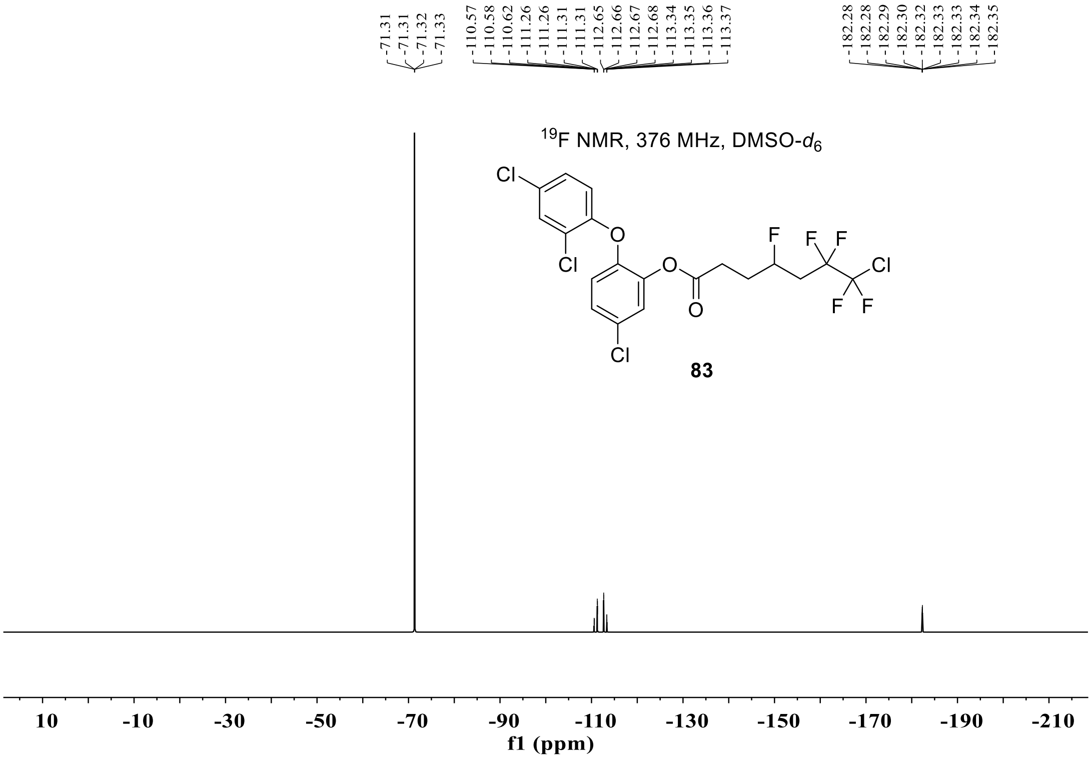
-71.31
-71.31
-71.32
-71.33
-110.57
-110.58
-110.62
-111.26
-111.26
-111.31
-111.31
-112.65
-112.66
-112.67
-112.68
-113.34
-113.35
-113.36
-113.37
-182.28
-182.28
-182.29
-182.30
-182.32
-182.33
-182.33
-182.34
-182.35
^{19}F NMR, 376 MHz, DMSO-d_6
Cl
O
Cl
O
F
F
F
Cl
O
F
F
Cl
83
10 -10 -30 -50 -70 -90 -110 -130 -150 -170 -190 -210
f1 (ppm)

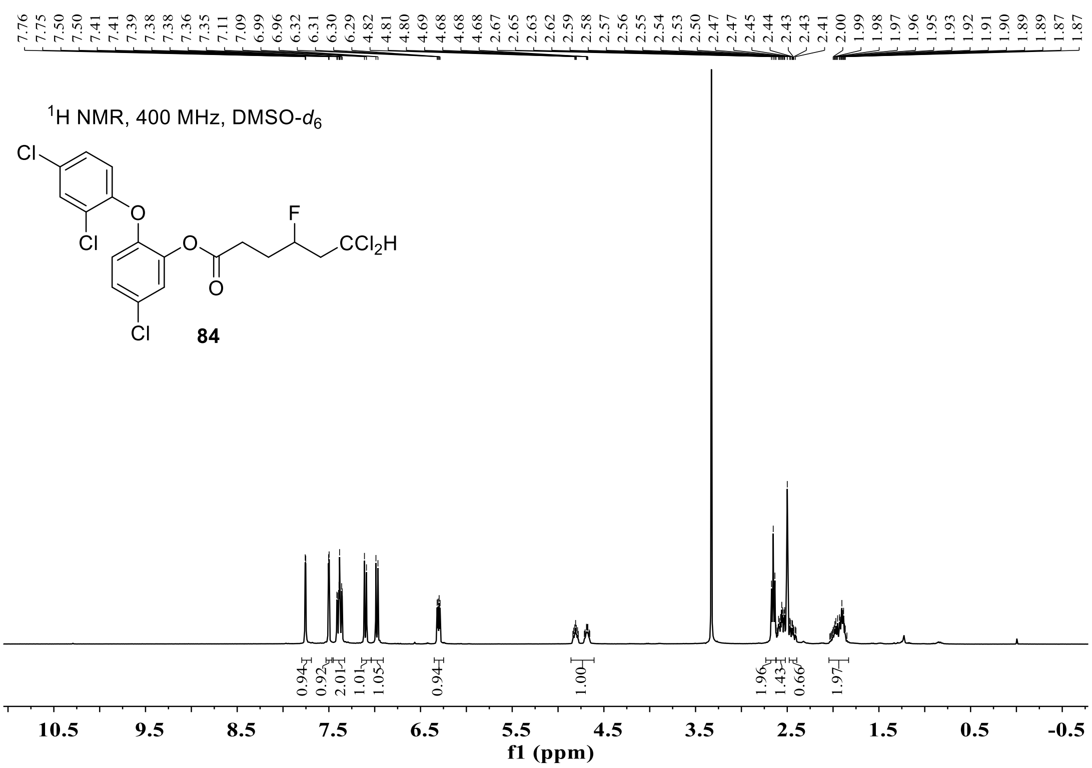
1H NMR, 400 MHz, DMSO-d6
Cl
O
Cl
O
F
CCl2H
O
Cl
84
10.5
9.5
8.5
7.5
6.5
5.5
4.5
3.5
2.5
1.5
0.5
-0.5
f1 (ppm)

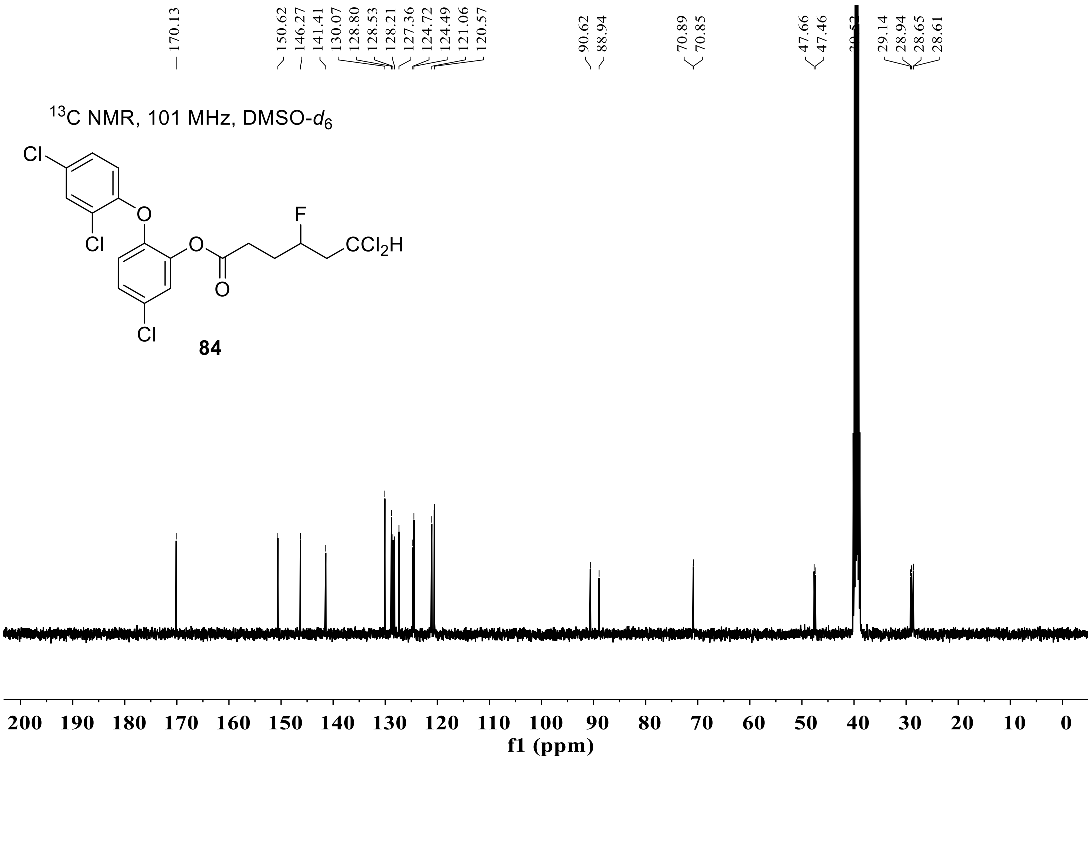
13C NMR, 101 MHz, DMSO-d6
Cl
O
Cl
O
F
CCl2H
O
Cl
84
200 190 180 170 160 150 140 130 120 110 100 90 80 70 60 50 40 30 20 10 0
f1 (ppm)

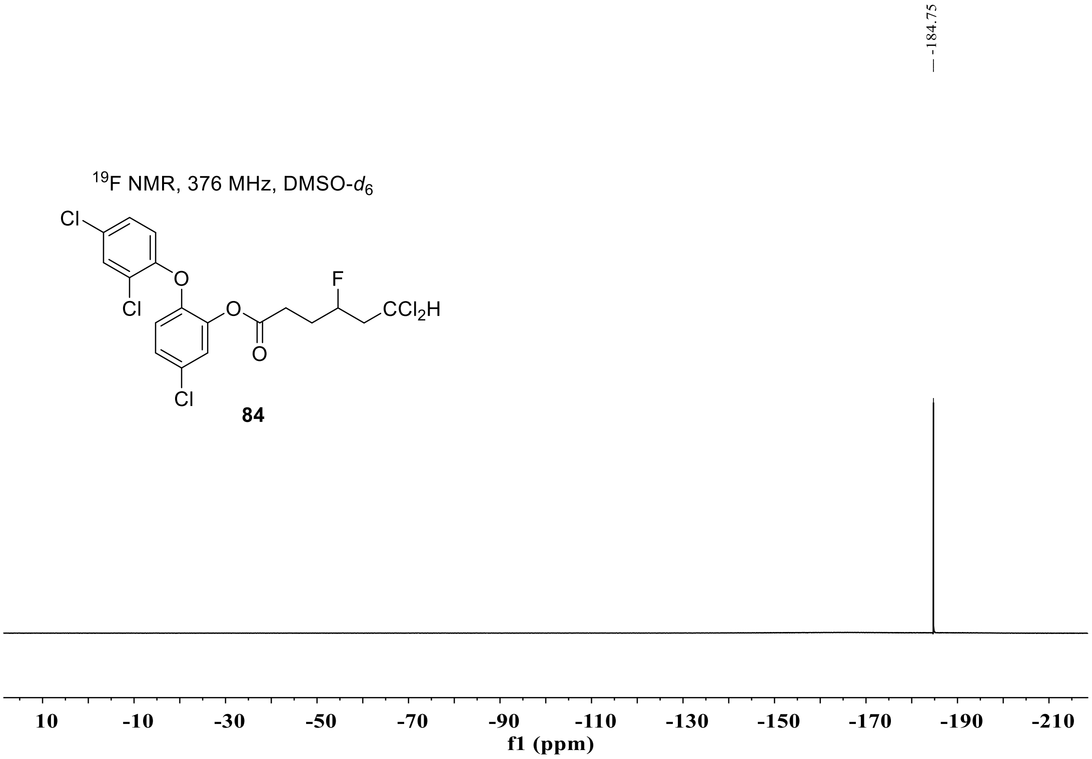

-184.75
^{19}F NMR, 376 MHz, DMSO-d_6
84
10 -10 -30 -50 -70 -90 -110 -130 -150 -170 -190 -210
f1 (ppm)

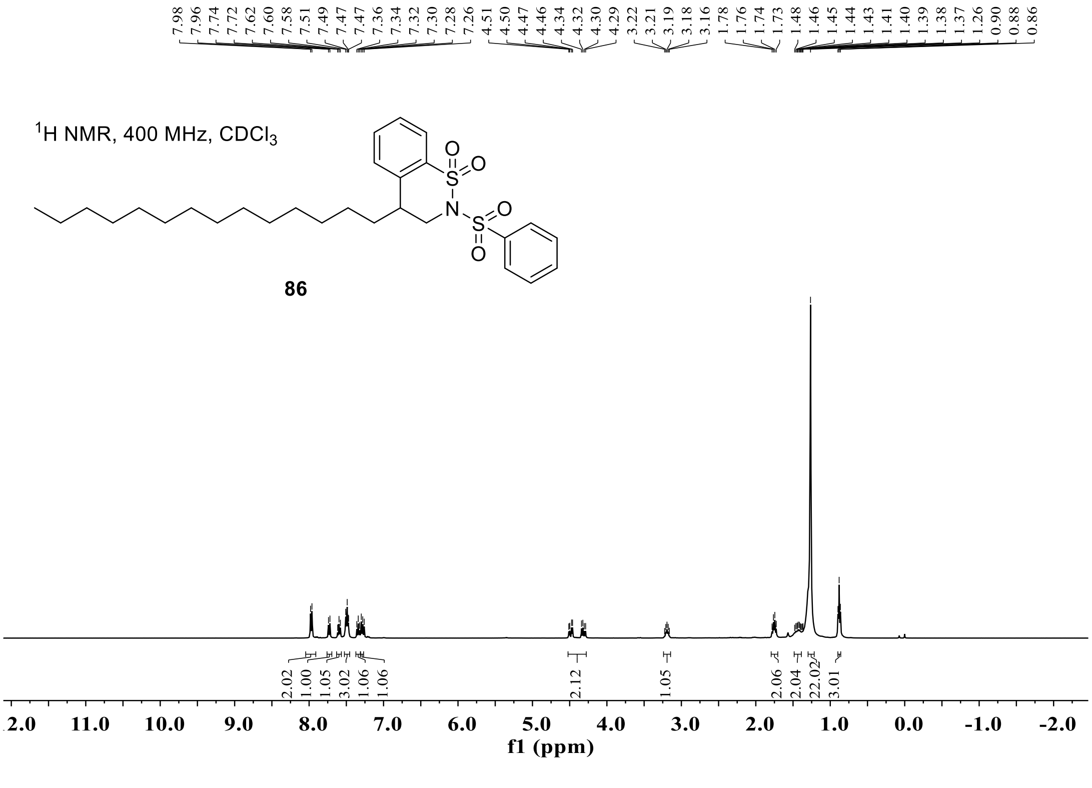

7.98 7.96 7.74 7.72 7.62 7.60 7.58 7.51 7.49 7.47 7.47 7.36 7.34 7.32 7.30 7.28 7.26 4.51 4.50 4.47 4.46 4.34 4.32 4.30 4.29 3.22 3.21 3.19 3.18 3.16 1.78 1.76 1.74 1.73 1.48 1.46 1.45 1.44 1.43 1.41 1.40 1.39 1.38 1.37 1.26 0.90 0.88 0.86
^{1}H NMR, 400 MHz, $CDCl_3$
86
2.02 1.00 1.05 3.02 1.06 1.06 2.12 1.05 2.06 2.04 22.02 3.01
2.0 11.0 10.0 9.0 8.0 7.0 6.0 5.0 4.0 3.0 2.0 1.0 0.0 -1.0 -2.0
f1 (ppm)

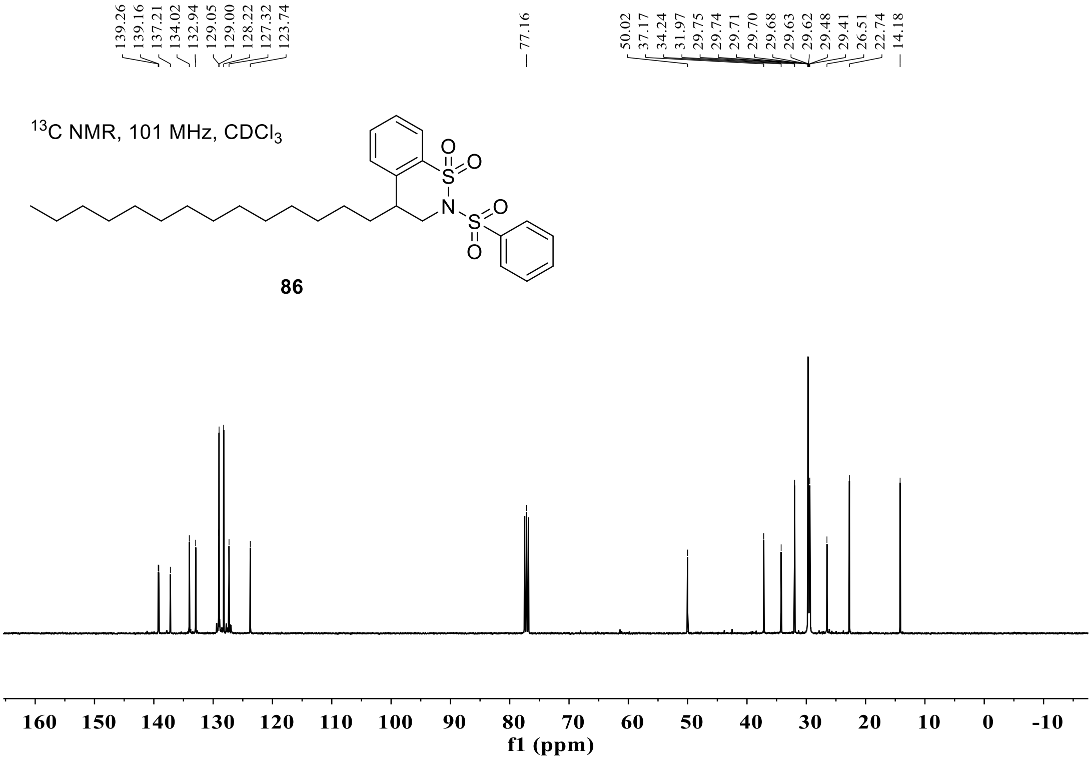
139.26
139.16
137.21
134.02
132.94
129.05
129.00
128.22
127.32
123.74
77.16
50.02
37.17
34.24
31.97
29.75
29.74
29.71
29.70
29.68
29.63
29.62
29.48
29.41
26.51
22.74
14.18
^{13}C NMR, 101 MHz, $CDCl_3$
O
S
O
N
S
O
O
86
160 150 140 130 120 110 100 90 80 70 60 50 40 30 20 10 0 -10
f1 (ppm)

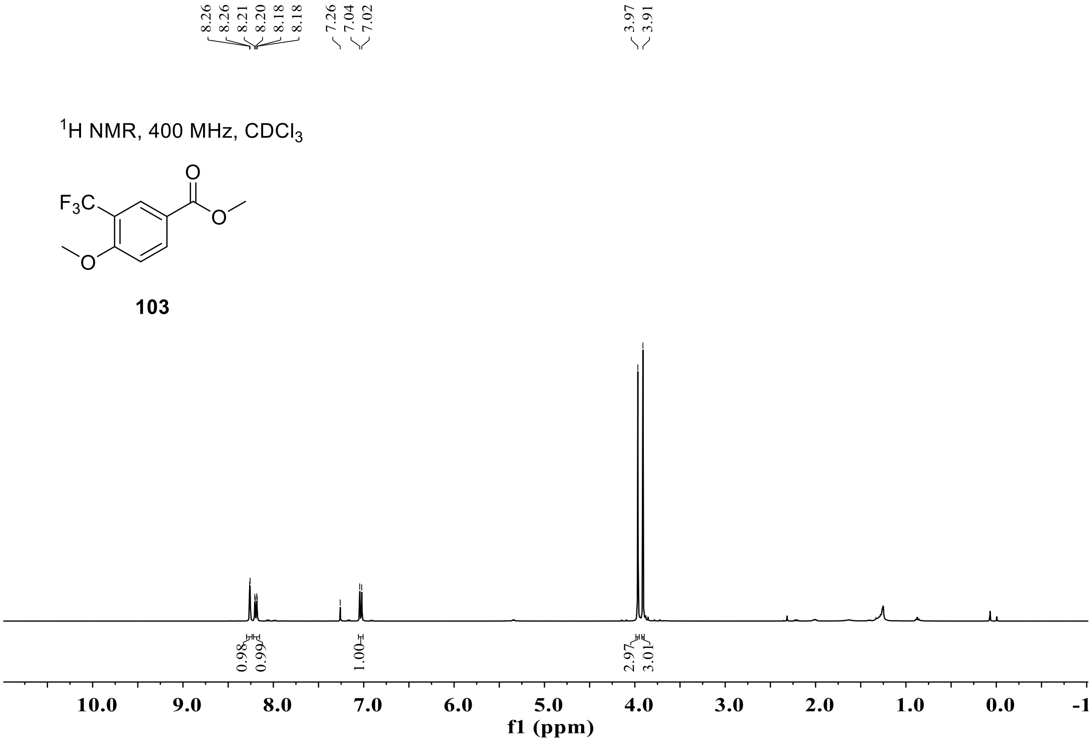
8.26
8.26
8.21
8.20
8.18
8.18
7.26
7.04
7.02
3.97
3.91
^{1}H NMR, 400 MHz, $CDCl_3$
O
F_3C
O
O
103
0.98
0.99
1.00
2.97
3.01
10.0 9.0 8.0 7.0 6.0 5.0 4.0 3.0 2.0 1.0 0.0 -1
f1 (ppm)

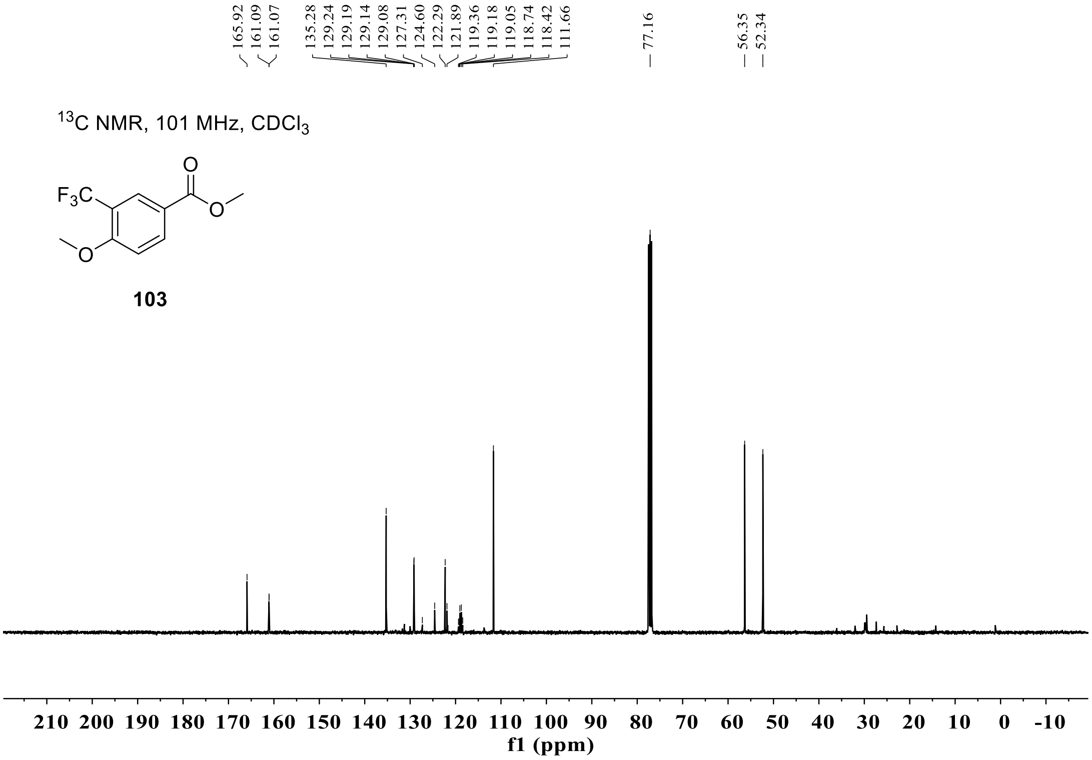
165.92
161.09
161.07
135.28
129.24
129.19
129.14
129.08
127.31
124.60
122.29
121.89
119.36
119.18
119.05
118.74
118.42
111.66
77.16
56.35
52.34
^{13}C NMR, 101 MHz, $CDCl_3$
F_3C
O
O
O
103
210 200 190 180 170 160 150 140 130 120 110 100 90 80 70 60 50 40 30 20 10 0 -10
f1 (ppm)

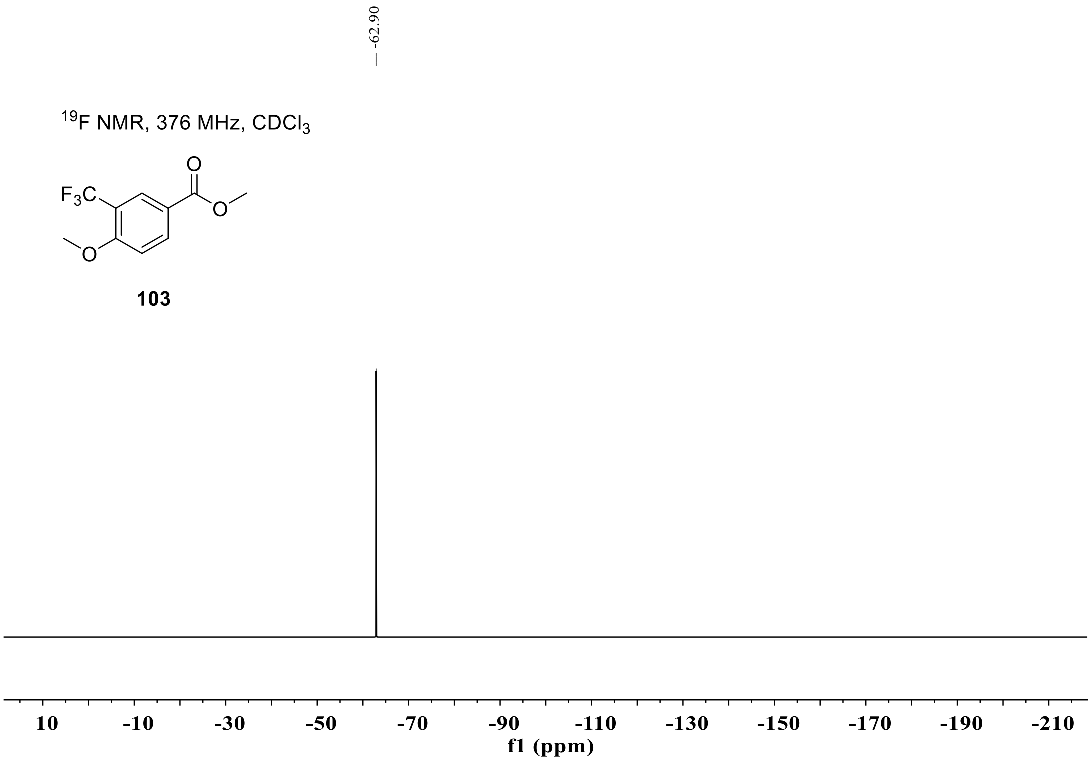
-62.90
^{19}F NMR, 376 MHz, $CDCl_3$
F_3C
O
O
O
103
10 -10 -30 -50 -70 -90 -110 -130 -150 -170 -190 -210
f1 (ppm)

4. Supplementary References

1. Ouyang, Y., Xu, X.-H., Qing, F.-L. Hydrotrifluoromethylthiolation of Unactivated Alkenes and Alkynes with Trifluoromethanesulfonic Anhydride through Deoxygenative Reduction and Photoredox Radical Processes. *Angew. Chem. Int. Ed.* **58**, 18508-18512 (2019).

2. Zhang, Y., Torker, S., Sigrist, M., Bregovic, N., Dydio, P. Binuclear Pd(I)-Pd(I) Catalysis Assisted by Iodide Ligands for Selective Hydroformylation of Alkenes and Alkynes. *J. Am. Chem. Soc.* **142**, 18251-18265 (2020).

3. Min S. J., Won S. K., Ye H. S., Ha J. J., Eun J. K. et al. Chemoselective Activities of Fe(III) Catalysts in the Hydrofunctionalization of Allenes. *Org. Lett.* **14**, 6262-6265 (2012).

4. Pirzer A. S., Alvarez E. M., Friedrich H., Heinrich M. R. Radical Carbofluorination of Alkenes with Arylhydrazines and Selectfluor: Additives, Mechanistic Pathways, and Polar Effects. *Chem. Eur. J.* **25**, 2786-2792 (2019).

5. Frisch, M. J., Trucks, G. W., Schlegel, H. B., Scuseria, G. E., Robb, M. A., Cheeseman, J. R., Scalmani, G., Barone, V., Petersson, G. A., Nakatsuji, H., Li, X., Caricato, M., Marenich, A. V., Bloino, J., Janesko, B. G., Gomperts, R., Mennucci, B., Hratchian, H. P., Ortiz, J. V., Izmaylov, A. F., Sonnenberg, J. L., Williams-Young, D., Ding, F., Lipparini, F., Egidi, F., Goings, J., Peng, B., Petrone, A., Henderson, T., Ranasinghe, D., Zakrzewski, V. G., Gao, J., Rega, N., Zheng, G., Liang, W., Hada, M., Ehara, M., Toyota, K., Fukuda, R., Hasegawa, J., Ishida, M., Nakajima, T., Honda, Y., Kitao, O., Nakai, H., Vreven, T., Throssell, K., Montgomery Jr. J. A., Peralta, J. E., Ogliaro, F., Bearpark, M. J., Heyd, J. J., Brothers, E. N., Kudin, K. N., Staroverov, V. N., Keith, T. A., Kobayashi ,R., Normand, J., Raghavachari, K., Rendell, A. P., Burant, J. C., Iyengar, S. S., Tomasi, J., Cossi, M., Millam, J. M., Klene, M., Adamo, C., Cammi, R., Ochterski, J. W., Martin, R. L., Morokuma,K., Farkas,O., Foresman, J. B., Fox, D. J., Gaussian16, Gaussian, Inc., Wallingford, CT, 2016.

6. Furche, F., Ahlrichs, R. Adiabatic time-dependent density functional methods for excited state properties. *J. Chem. Phys.* **117**, 7433-7447 (2002).

7. Giovanni S., Michael J. F., Benedetta M., Jacopo T., Cammi, R. et al. Geometries and properties of excited states in the gas phase and in solution: Theory and application of a time-dependent density functional theory polarizable continuum model. *J. Chem. Phys.* **124**, 094107 (2006).

8. Bauernschmitt, R., Ahlrichs R. Treatment of electronic excitations within the adiabatic approximation of time dependent density functional theory. *Chem. Phys. Lett.* **256**, 454-464 (1996).

9. Adamo, C., Jacquemin, D. The calculations of excited-state properties with Time-Dependent Density Functional Theory. *Chem. Soc. Rev.* **42**, 845-856, (2013).
